# The testes transcriptome of the New World Screwworm, *Cochliomyia hominivorax*

**DOI:** 10.1016/j.dib.2016.11.026

**Published:** 2016-11-19

**Authors:** Kylie G. Bendele, Felix D. Guerrero, Connor Cameron, Adalberto A. Perez de Leon

**Affiliations:** a United States Department of Agriculture, Agricultural Research Service, Knipling-Bushland U. S. Livestock Insects Research Laboratory, Kerrville, USA; b Veterinary Pest Genomics Center, United States Department of Agriculture, Agricultural Research Service, Kerrville, USA; c National Center for Genome Resources, Santa Fe, NM, USA

**Keywords:** New World Screwworm, Transcriptome, Testes, RNA-seq, *de novo* assembly

## Abstract

The New World Screwworm (NWS), *Cochliomyia hominivorax*, is a pest insect that is endemic to subtropical and tropical regions of the Western Hemisphere. The female lays eggs in open wounds or orifices of warm-blooded animals. Upon hatching, the resulting larvae feed upon the host׳s living tissues, which can become infected and death can occur. The sterile insect technique was developed to eradicate this pest from North America and new female conditional-lethal strains that generate only male individuals are being developed for use in the eradication program. To facilitate the identification of useful transcripts and gene promoters for these new strains, we used an Illumina Hi-Seq protocol to sequence the testes transcriptome of NWS. We report the assembly of 4149 transcripts (≥200 nt) from testes dissected from NWS males obtained from the J06 strain used in the screwworm production plant in Pacora, Panama. Functional annotation resulted in 2060, 2031, 558, and 325 transcripts with assigned BlastX, Gene Ontology, Enzyme Codes, and KEGG pathway information, respectively. In the Gene Ontology annotations, 6% and 3% of the transcripts in the Biological Process Ontology were noted as Developmental Process and Reproduction, respectively. This data set will serve as a resource to facilitate studies of sex determination in the NWS and the development of recombinant vectors that can be used to create new male-only strains of NWS.

**Specifications**

**Table:** 

Subject Area	Biology
More specific subject area	Insect genomics
Type of data	Transcriptome sequences and associated annotations
How data was acquired	2×54 paired-end read RNAseq of RNA isolated from testes dissected from NWS males
Data format	Raw FASTQ and processed FASTA sequence files
Experimental factors	Testes dissected from 100 male individuals into RNALater
Experimental features	Assembled transcriptome of testes tissue dissected from adult male NWS
Data source location	Strain of NWS used in the screwworm production plant in Pacora, Panama
Data accessibility	Data is with this article and also available at the National Center for BIotechnology Information (NCBI) Short Read Archive (SRA) through the direct link http://trace.ncbi.nlm.nih.gov/Traces/sra_sub/sub.cgi?subid=707563 or through SRA accession number SRP076734. The *C. hominivorax* testes transcriptome shotgun assembly project has been deposited at DDBJ/EMBL/GenBank under the accession GEVJ00000000. The version described in this paper is the first version, GEVJ01000000. The overall BioProject ID is PRJNA324578 and the BioSample accession is SAMN05213682.

**Value of Data**

• Testes-specific transcript sequences to supplement the available NWS transcriptomes previously reported for embryos, larvae, adult male, and adult female [1].
• Resource for investigations of sex-specific gene expression and sex determination pathways.
• Provides candidate protein coding regions and gene promoters for the development of recombinant vectors that can be used to create new male-only strains of NWS.

## 1 Data

Testes were dissected out of male NWS from Pacora, Panama. Following RNA isolation, 1 lane of 2×54 paired end RNAseq reads were obtained, *de novo* assembled and annotated. The raw reads are accessible at NCBI׳s SRA through the direct link http://trace.ncbi.nlm.nih.gov/Traces/sra_sub/sub.cgi?subid=707563 or through SRA accession number SRP076734. The assembled transcriptome shotgun assembly project has been deposited at DDBJ/EMBL/GenBank under the accession GEVJ00000000. The version described in this paper is the first version, GEVJ01000000. The overall BioProject ID is PRJNA324578 and the BioSample accession is SAMN05213682.

## 2 Experimental design, materials and methods

### 2.1 Experimental design, materials and methods

#### 2.1.1 Testes tissue

One hundred male NWS adults were obtained from the Pacora, Panama screwworm production plant colony and testes dissected out and placed directly into a 1.5 ml microcentrifuge tube containing 100 μl of RNA*later* at room temperature (Ambion Inc., Austin, TX, USA). The tissues were shipped on dry ice to the USDA-ARS laboratory for RNA isolation.

#### 2.1.2 RNA isolation

RNA was extracted using the ToTALLY RNA Isolation Kit, following the manufacturer׳s protocols (Ambion). A disposable pellet pestle (Kontes Inc., Vineland, NY, USA) was used to grind the tissue in the microcentrifuge tube, using a final volume of 700 μl of the ToTALLY RNA kit׳s Denaturation Buffer. The optional LiCl precipitation step to selectively precipitate RNA was incorporated into our protocol and at this point 45 μg of nucleic acids resulted. Agarose gel electrophoresis determined the integrity of the nucleic acid sample was good, but DNA was still present. Following DNAse treatment with the Turbo DNA-*free* kit and protocols (Ambion), 40 μg of DNA-free RNA was obtained.

##### 2.1.2.1 Sequencing and bioinformatics

Sequencing was performed at the National Center for Genome Resources (Santa,Fe, NM, USA) using the standard Illumina RNAseq library preparation protocol and a single lane of the RNAseq 2×54 paired-end approach. A total of 72750822 raw reads were produced and quality preassessment performed by the Illumina pipeline and the contaminant filtering pipeline developed at National Center for Genome Resources. *De novo* assembly of the transcriptome used an iterative k-mer strategy in ABySS [2] followed by gap closure and additional assembly using overlap methods in both MIRA [3] and Cap3 [4]. The assembled contigs were screened at submission to the NCBI Transcriptome Shotgun Assembly (TSA) database using the NCBI foreign contamination screen protocol. Supplementary File 1 contains the FastA sequences of the final assembled dataset of 4149 entries ≥200 nt.

**Fig. 1:**
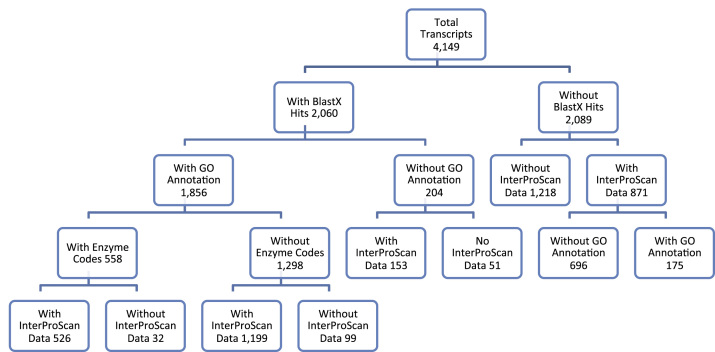
Summary of the annotation process for the assembled NWS testes transcripts. Flowchart summarizes the results of the various annotation steps, including BlastX, GO, InterProScan, Enzyme Codes, and KEGG pathway. The number in each box indicates the number of transcripts associated with the respective annotation tool and presence or absence of information.

**Fig. 2:**
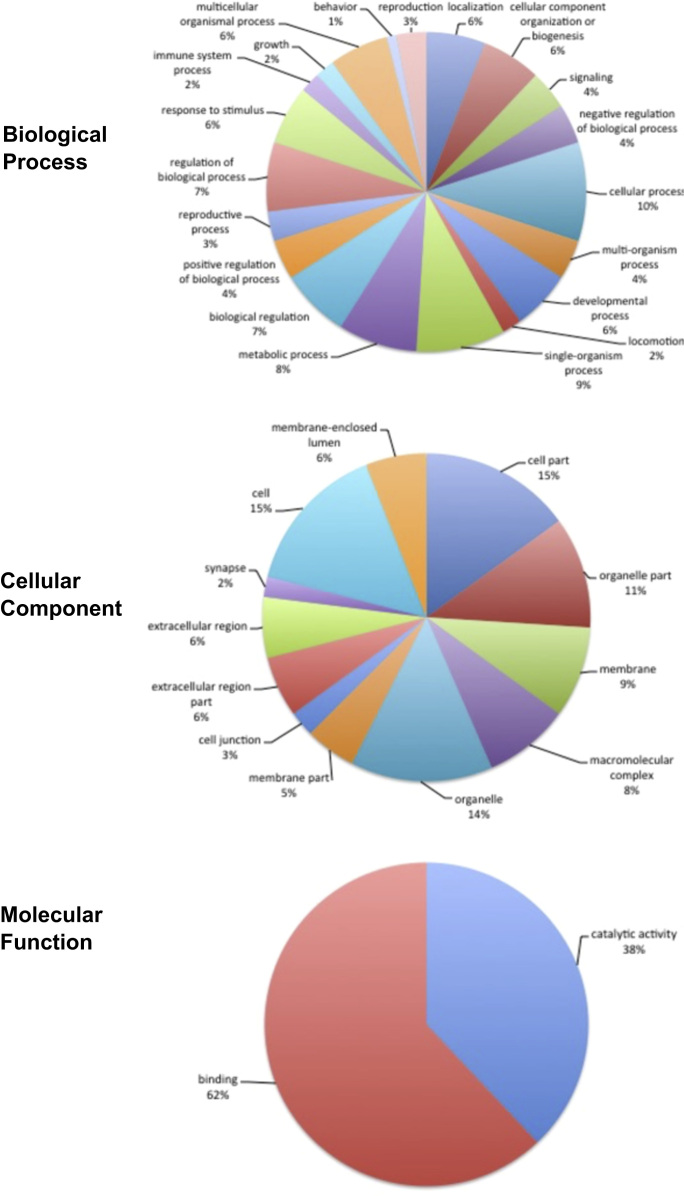
Gene Ontology classifications of assembled dataset contigs. All NWS assembled contigs were annotated with Blast2GO mapping and level 2 GO terms shown for Biological Process, Cellular Component, and Molecular Function ontologies. The percentage of annotated transcripts with each indicated GO term is shown.

The assembled transcripts were screened using Blast2GO PRO version 1.4 plugin CloudBlastX [5], [6], [7] on the CLC Genomics Workbench version 8.0.1 (http://www.clcbio.com, CLC Inc, Aarhu, Denmark) against the UniProtKB/Swiss-Prot database using 1.0E-10 e-Value cutoff. The transcripts with CloudBlast hits were mapped using Blast2GO PRO Mapping and GO Annotation performed using Blast2GO PRO Annotation [8]. KEGG pathway maps were determined using Blast2GO Basic version 3.1.3. Statistics from the different steps of annotation of the transcripts can be found in Table 1 and a hierarchy summary in Fig. 1. Fig. 2 shows the functional annotation of *C. hominivorax* testes transcripts for Gene Ontology Level 2 terms for biological process, cellular component and molecular function. Transcript information including CloudBlastX hits, GO terms, InterProScan, Enzyme Codes and KEGG pathway data can be found in Supplementary Table 1.

## Conflict of Interest

The authors declare there is no conflict of interest on any work in this paper.

## Figures and Tables

**Table 1:** Table of transcriptome assembly statistics.

Number of transcripts	4149
Total size of transcripts	2,675,766 nt
Longest transcript	10,293 nt
Shortest transcript	200 nt
Number of transcripts >1K nt	716
Number of transcripts >10K nt	1
Average transcript length	644 nt
Transcripts with InterProScan	2596
Transcripts with BlastX hits	2060
Transcripts with GO Annotation	2031
Transcripts with Enzyme Codes	558
Transcripts with KEGG Pathways	325

## Appendix A Supplementary material

Supplementary file 1. FastA sequences of the final assembled dataset contigs.

.

Supplementary Table 1. Functional annotation information for the NWS testes transcripts.
