# Supplementary material for: The testes transcriptome of the New World Screwworm, Cochliomyia hominivorax
Source: Data Brief. 2016 Nov 19;9:1141–6. doi: 10.1016/j.dib.2016.11.026 (PMC5133534; doi:10.1016/j.dib.2016.11.026)
Supplement: Supplementary file 1 — FastA sequences of the final assembled dataset contigs. [file mmc1.docx]

>BPA_3

CATCTATTGAAGCGTTAGGAACTTTGGAAGCAATACGGGCACATTTACCTATTTCGAAACCTACAAAACCTAAATGGCGTCCAGCTGGTAGACTAAGAGTAGATCGTCGAGTAAAACCATCTGAAAGAGTGTTTACAATGGATTTCGGTCGTGTTTTGATACCCTCTAAAAGAACACAAAAGAAAAAAGGTTGGAAAAAGCTTGATGTTATGTCTCCAATTGCCGAAGATAAAACGATAACTGATGATGACTTTATGCAATTTTCCCCAAGTCTCCAATTGTCGAAGATAAAACGATAACTGATGATGACTTTATGCAATTTTCCCCATTAAAACCTCACGAAGGTCAAAAGCGTTTGCAATATTTAAAAGAGCAAGCAGCAAGACGTGTAGCGGAAACTATTAGAGCTGCTGAAGAAGCACAAGCCGCAATTTCTTTTCAAACTCCTCCCCGTCGCCAAGCAGTTAGTCCTGGACGTACTCCCCAGACACCGGAAATGACAACTGCAAAATTTAGTCCTATTCAACTGTTAAGTCCAGCTAGTCCAAGAACTGAAGAAACTAGGCGTGAAAAAGTAATAACAAAAATGGGTCCTAGTGGTGGTACCGAAACTATAAGGGAAGAAATTACTGAGACTTCAACAATTGAAAACGGAGAAGTTCATACATCAACAAGGCAAGTTACAACCAAAACAGATGAATATGGTAATATAACATGCCACATTCAAGAAGGTAAAGGTTTATCAGGGGAAGAAGAAGCATTAGCTGATATAGAACCGCCAGAATTTTTGGATACATCTGATATACCACCTCCAAGGGATCCTTCTATACCCACACCCGCTGAAATAATCGATAGAATTCGAATGCAAGTACAGCAAAAGCTAAATGTTGCCTTACCAGATATACCACAAAGTCCTGTTCCCGTATCTCCAGGTCAGATAGAATTACCTGAAGATTTAACAGACGAGGAATTGTTTGATATTGAAGCGCCTGAGTTCGAGAGAACACCACCAAAAGCATCGCCAAAATTAAAGGCATATACACCAATAGAAAAAGACCAACGAATGTATGCTTGGGGAGAAGTGCCTGTACCAACTCCGCCACCGCGTCAACCATCACCTCCACGAATACCAACACCAAAACCTGTTACACCACCTCCCAAAATCCCAAGTCCTAA

>BPA_5

CAGGGACCAAAGGTCTACCATCAATTTCATGGCCAAGTAAATATTTGTGATCGGAACTGGGAGAAACTGTTATGGTTTTATTACCTGTTTTAATACATTCATCTTCATAGAAGTAAACCATATAATCATTTGTATGATCGAATTCTATTAGGTCACTTATTGTGGGTGTTCCTCTGGGAACGGGCCACTTGACAGGTGGGTACAGTGCATCTAAATTTAATTCACATCCATGTAGATAAAGTTTTGCAAGAGCTTCGAGCAGATGGAAAACATTGTCGTTTACATTTCGTCTAAGAAGGCCAATATAAATACCATCAGGATTACAACTTCTCATGATACTTTGCAGAAGAGCATGTGGTGCTATTTCAACAGATAGAGAGTTTTTGGGCAAGTGTGTTGCGGCCTCTTCGAATAGGACACTGTTGAGAAGATTGTGTGCTTGATATTCAGTACAACAGAATTTGCAATCATCTATCAACCATTCAGTAGACGGATAGGTTGTACTTATCCATTTTTCGGAACGGACTTTTGGATTCGGAATGATTTCTTTCAGATACTTTGAAAGTTCAGGGCCAAACTTCTGAATATATCTGCTATGGTATGCGATATTTGAGCAAGGTACTTCCTTGGCAAAAATTTTTTTAGCTGTTAATGTTTCTACAAATTTGGCGACATCATTCTCTGGTCCCGAGATTGTACATGATTCACAACTATTGTGACAAGCTACTTCAATGTTTGGTGGTAATATTGATTTAATATCTTTGTAGCCTAATCCAACAGCTGCCATAGAACCACGAATATTTTCGACATCTAAGCTAACTTTACCACGGTAATAGGAGGCGAGTATAGCTTGCTCTAACGTAAAGCAACCATCGGCATATGCACATGCGACTTCTCCAGCAGAATGTCCAATTATATAATCTGGTTTCATTCCAATAGCCTGCAGCATATCAACTAAGGCAATTTGTGTGGCAGTAATGCATACAAAACAGTTTACAATATTTTGGAAAGTATCTGGATTATCGGATGTTAATACGTTTATAAGATCTACACCTTTTGACTTTAGAATTGAGTGACATTTCATAATTGAATTAGAACATATTTTGATTTTCATAAGGTCTTTACCCATTCCAAGCCATTGAGAGCCCATTCCTGAAAATACCCATACAATTGGATATCTAATACCTTCATTGTACTGAACTGACCTGGCCAAGCTTATTGGCTTAGAGTCATTGTTAGGTTTTAAAATGTTATACCCTCTATATACCATACCCTTAATACCTCCTTTTTGTGACGAACGCATTAATGTTAAATATTCCAAGTTAAATGGGCGTGCTTCTATGGTATTAAACGTTTTCTCAATATTACTTTCGATACGACTGGACCATGTCATTAGATAAGGCAAATCATTTGATTTCCCATACGAAGACGCATGTTGTTCAATTGGATTTTGTTTTAGAAGAGCATGGGCATTAGCACCACCAAAACCAAATGAATTCACACATATATATGGCTGTGTCAATTCGGTTTTTTCTGTTACTACAATCATTCTTCCTTCCTGAATTCCATTAATTCTTTTTCGAAGTGTGGTTATATTAATATTTGGCGGAATACAGTTATTATTAAACGATAATAAGACTTTAGTTACAGAAACAATGCCTGAAGATGCCTCCGAATGACCGCAGTTTGATTTAACTGAACCAATCAAAAGTGGACTGTTACGATGCTTGCATATCGATTCATCAATCGCTTTACACTCTTCAGGATCTCCGACTACAGTTCCTGTACTATGGGCTTCCATATATCCCAATTTATTTAAATCAAAATTGAGGTCTTTGTAGAACTTTGAGAGCAATTTCATTTGCATGTGACCCGAAGGATAGGTTAATCCTTCGGCCTTAAATCCATCACAATTCGTAGAGGAATGTATCAATGAAGCATAAATCCGTTTAGCATCGCATTTTCTTTGAAGAAAAATGCAACATATGGCTTCAGAACGAACATATCCATTGGCATCTTCATCAAATGGCCTACAATTTCCTGTCATTGAAAGAACTCCAAGCCTAGCAAATTTAGCTGTCACGAATGGACACGTCATCGCATTGGTACCACCAACAATTGCACTATCTACAATTCCATTTTTCATATGTTCATACGCAAGATTCAGTGCATACATGGAACTGGAACATGCAGTGTCACAAACCATAGATGGTCCATTAAGTCCCATTGTAAATGAGATACGATTTGCAAGAAGTGATTTACTTGATCCCAGCAACCCGAAGCCGCTATCTATACGATTTGCAAGAAGCGATTTACTTGATCCCAGCAACCCGAAGCCGCTATCTTGCTTATCACTATACATAAGATGTGCATCCGATTCAGATGAGCAGACCGCTATATATACTCCAGTATTAGTGTTGCGTAAATTCGTAGGATTTACACCTGCGTCCATGACAGCCTTATAAGACTCTTCAAGTAAAATTCGCATTTGGAAATCCATACTATCGGCTTGGTTCTGATGAATACCAAAGAAAGTAGCATCAA

>BPA_19

GCAAGACCATGGTCAAAGTTGATGGCAAGACCTTCACTTTCTTGGACAAAAAATGCGAACGCTCTTACTTGATGAAGCGTAATCCCCGTAAGGTTACCTGGACTGTCTTGTACCGTCGCAAGCACCGTAAGGGTATTGAGGAAGAGGCCACCAAGAAGCGTACCCGTAGAACACAAAAGTTCCAACGCGCTATTGTTGGTGCTTCCTTGGCCGAAATTATGGCTAAGCGTAACATGAAACCTGAAGTTCGTCGTGCCCAACGCGAACAAGCCATCAAGCAAGCCAAGGAACAAAAACGTGCCGCCAAAGCTGCCAAGAAGGCCACTCAACCCGCCCAACCTAAAGCCAAGGCTGCTCCCAAACAAAAAGCTGCTAAGG

>BPA_31

GTTACTGCAAAAACAAACCGTATCCTAAATCTCGTTTCTGCCGTGGTGTACCAGACCCAAAGATACGTATTTTCGATTTAGGAAGGAAGAAAGCTACTGTAGAAGATTTTCCACTATGCGTCCATTTAGTATCTGATGAATATGAACAACTTAGTAGCGAAGCTTTGGAAGCCGGACGTATATGTTGTAATAAGTATTTAGTTAAATACTGTGGAAAGGATCAATTCCACATAAGAATGCGTCTTCATCCATTCCACGTCATTCGTATCAACAAAATGTTGTCCTGTGCCGGAGCTGATAGGCTCCAAACTGGAATGCGTGGTGCTTTCGGTAAGCCACAAGGTACAGTTGCAAGAGTTCGTATTGGTCAGCCAATTATGTCTGTTCGCTCTAGCGACAGATTCAAAGCTCAAGTTGTTGAAGCTCTACGTCGGGCTAAGTTCAAGTTTCCCGGGCGCCAAAAGATTTATGTTTCAAAGAAATGGGGATTTACTAAATACGATCGTGAACGTTACGAAGAACTGCGCAGCGAAAATCGATTAGAACCAGACGGTTGCAACGTCAAATACAGACCAGAACATGGGCCCATGTCGGTATGGGAGAAAGTTCAGCGT

>BPA_32

TCACCAAAACTACAACTTACAAACTAGAACCCGATGGAAGTATCTCAAGTAAATCATTGCTGCAAAAAAGACATCGTTATGTCTATAACTCGGAAAAAGTACCCGATGATATTCAAAAAAAGACATCGTTATGTCTATAACTCGGATAAAGTACCCGATGATATTCCCATCGATGAACTAAGAAATTCTCCCACTGGTACCATAACAAGAACCTATATTGACAGTGATGGAAGCAAAGTAACACAAACGATTGGAGTTAGTAAACCTAAGCCTTCCACTGTGATAGATCCAGATGATTTAACGGGAGATTGGTTTAATAAACCTTTCCCAGCATGGCCAGAGGAAACAGCACCTGCCACCAATACAAGGCATAGAACTATTACTAGAACATATACAAGACCAAATGGAGAGAGGAGAACAGTACAAACTTGGAACGAAAGTCCTGTACATCGTCTAACACCACATTTGAATAATAGACCTGCAACGAGAACTTATTCTAGACCCACCAATGTGGAACCTTTTGAGATTAACAGCAAACCTAAATTTCCTAATATAGGAGATATCGATTGGCCCACAATTGAGGATCCTGAAGATGAAGATGGCTGGGAAGTAGTAGAGGAACCAGAAGTTGAAGCTACCACCAAGAAATCACCTCAAGCTTGGACCCCGAAAACCTGGGAATGGCCAGAATTTCCTACTCGACCCACCACAGCGGC

>BPA_34

GTTGTCGTTGTCGTTGTCGTTGTTGCTATTACCCGTTAATGTTTGTTGTTGAAATTCATTTAAAATACGACTTTGTGGTATATCTGTTTCTACCCTACTACCAACAATATCGTTGGTATTAGCACATCGTTCTTGATCAAAGAATATATCAATTATAATGCGTACACCACTTGATGTTTTAGCAATTGTGGTCTTACTTTGGTAGGATGTTTCAATTGGAGTCTCTTGACAAATGTTGGCCTCCTCTACACTATCAATTGACAGTAAACGATTCTTCGATTGCTGTTGTTTTTGCTGCAGATGCCCCACCACAGGTTGAGAATTATAGTTGTTGCTGTTGTTGTGGCAGAGACCATTGCATAAGTTTTTATTTAGTTCTTCATTAAAATCACTTAATATATTTGGCCTAATGGTGGTGGCTGTATTAATATCAGCAGAATTTCCTACATCATCACTGACTCTGTTACAATTCTCAACCACAAGAAAACGCTGAGTTAAATCTTTAGCATTGATTTTATTACAATTGTTGTTACTGTTGGTGTTGCTGTTGCTGTTGCTATTAGAGCTTCTAATACTAATATTGCTTTTGCGTTTTTGGTTGTTCTCATTGTTTTGTTGCAATAACAAAGATGATGTTGACGTTGACGACAACATTTTGGTGGTTATCGTATTTATACGGTTTCTATTTTGGCAACACATACATGCTGAACAACTGTTACAGCAATTGATTGTATTTTGATTATGTTCATCGATAGTTGGTGATGATTTATGTCCAATTTGGTCAACAAATTGCTTAGCATTCAGATGAGATCCATAATTATTTAAACCACAATATGATGATTTGGTAGCAGCCAATAAATGTTGCGAATGATGATGATGATGATGTTGTGGTCTTTTGGCAGCGAATGATGATGAAGATGATGTTGTGGTCTTTTGGCAGTATTTAATTTATATTTGATTTTATGATTAATATTATTTGTTTCTTCTTCCTTTGATTGATTATTA

>BPA_35

TTATTCATATACATGCGTTGTAATTTACGAACCATGGGAAATATAACTTTTGAATTAACTACATCGTCTACAGATATGGTTAATCGTTCATTATTTCGTGAATCAAAATCACCTTTGCCAATTTCGGTTGTACGGATATGGTTAATCGTTCATTATTTCCTGAATCAAAATCACCTTTGCCAATTTCGGTTGTGACGTCCTTTTGCGGCAACACCGAATGTATGCGTTCAGTGCATCGTTTACATTGATTCATAGTAAGAGGATACTGCGATACTGAGGGTTGTTCCAGCAAACCACAAGCAGTGTTATTATCAACCGTGTTATCAATGTCTGCATTTCCCATTTGCTTTAGAGTCTCATCAATGACTTCGTGAAGCTTACATTGAGACGTTACCATTTTACGTAACTCCTGAGAACTTTTGCGACTTACTACCGTTTTAGATGATTTTTGACTTTTAGCACTATCTTCTGAGCCATTACCAAGACCACACAAATTTGAATCATTTTCATATAAATAACGATGATCTAACAATTGTTTACCGATGTAGTTTGATATTTTCGGGAAGAATTAGTAGTTAAAACTGGGGCAGATGGCTTGGGATTAGTTGGTAACTGTTTTATTGAAAATTCATTGTCACCGTTACGCTGTCTGTAGGAATCGCTACCATAATGTGATGCTACACCATTTGGATAATATAACATTCGATCGGCATCCCCACCACTTGTTTGAGCAAGATTAAAACTTCTTTGCTTAGCGTTAAGATTTGCTTCTAATTCTTGTACATTTTGGTGGGCTTTCGTATACTCTTTGGAATGTTGAGCCACAAGCTGCTCATTTAAATAACTCTGTTGAGTTTTTTGTTGTAATTTTGGATCCTTCACACGTCCCATA

>BPA_37

CAACAACAACAACAGCAGCAGCAAACATTGTCTGCTCCCCTGTCGCCAACAACAACCTCCTCCCAGCCATATGATGAAAATGATTCCCAGCATGAGTTTAGCAGCAGTTGTCTGAAGGAAAATGCATTTGGTATGAGTGATAAAAGTAGTACAACCAATAGCCTACCGAATAGTCCTATACATAGTAGCAACAACAATAATACATCGCTGTTGAATAACAACAACAATACCAGCCTTTGTAATCACAACATACCCAATAGTAACAACAACAACAACAATAATAATTCTATCATCAGAAAGAATAATAATAACAACAACAACAGCAGCAATAACAATCCCAATAATAATAACAACAATACATGTAGTGCCAATCGTAATGTTGTCGATGATGACTCTTGTTTACGCATGGATACCGATAGACCATTCGGTGAAAAGGAGCCCGATCCAGATAATATTAAAATGTTTGTTGGCCAAGTGCCAAAATCTATGGATGAGGCACAACTACGCGAAATGTTTGAAGAATATGGTCCAGTGCATTCAATAAATGTATTGAGGGATAAAGCAACCGGAATTAGTAAAGGTTGTTGTTTTGTGACATTTTATACCCGCCGCGCTGCCTTAAAAGCTCAAGATGCCCTGCACAATGTTAAAACCTTAAATGGGATGTATCATCCTATACAAATGAAACCCGCCGATAGTGAAAATCGTAATGAACGGAAGCTCTTTGTTGGTATGCTAAATAAGAAATTGAACGAGAACGACGTACGTAAATTATTTGAAGTGCACGGTGCGATCGAAGAGTGTACAGTGTTGCGTGATCAAAACGGTCAAAGTAAAGGGTGTGCATTTGTGACTTTTGCCACAAAACACGCAGCGATATCAGCAATTAAAGTAACTTTAAATCAAAATAAAACTATGGAAGGCTGCACTTCACCATTAGTTGTGAAATTCGCTGATACCCAAAAGGAAAAGGAACAAAAGAAAATTCAACAATACCAAGCGAATCTATGGAATTTGGCGACGAATATTAATATACCTTTGGGTCAAACAGCGACAACGGTTTCGACGCCCATGCTACCCAATCCACCCCAACAGGCTAGTCCTGTTTTGGGAGCAGATGCTATAACGCCAGCATCATTACAATTGTTGCAACAATTGCAGGCTGTTGGTTTACAACAACAACTATGGCAAGGTTTGGGTGCACAAACAAATACTGCAGATACAGCAGCTGCTGCAGCCGCTGGTCTTCTACCTCCTATGACTGTACAAAATTTAGCTGCTTTGGCAGCCATGACACAATCCTCATTGACAAGTGCTGCCACTAATCCGGGCAGTGCCCAGTTGACAAATACAGCCGCCTTATTGTGGTCTGATTCAAGTTCTTTAGCATCGGCCATATTAGGAAAAACTGCCAATACAGGGTCTGATTCAAGTTCTTTAGCATCGGCCTATATGTCAACAGCCGGTCTATCACAATTTGGTAGTACCGCCTTAACTTCATCACCTTTAGCCAGTGTTGCCTTAAGTAATGCAGCTGCTGCAGCAGCAGGAAAACAAATTGAGGGTCCTGAAGGCTGTAATCTATTCATTTATCATTTGCCACAAGAATTTACTGACACAGATTTGGCATCAACTTTC

>BPA_39

CATATTAACACTTGTTAAGGTTTGATTTGTTTGAGTGCTCATAACAGATAGGCGGCCAACTGTTTCTTCGGTCGTGGTAGAACATAAAGTAGTTGTCGTTGTATTCAAATGATCTCTAACCGAGTCTACTGTTATGTCACCCATTGAACTGGTAATAGTTTCATTTGCTCGATTAGGAGAATTGCCATCGCTAACACCATTTGTTAGGCCAGCAGCATGATCA

>BPA_86

ATCGATTTTAACTTTGCTGGTGGCATTTTCAGTAGCGCTTGATGGTGGCCCAACACGATTCTTAATCTCAGCCGCCATTGTCATGAAGGCCTGCTCCACATTGGTGGCGCTTTTGGCTGAAGTTTCAAGGAATGGAATGCCTAATTGATTTGCATATTCCGCGGCTGTGGTATGATCTACCACCTTTTTGGTGGTTAAATCACTTTTGTTGCCCACCAATAATTTATTGACATTTTCACAGGCATATCGTTCAATTTCCTCAAGCCATTGCTTAACATTATTAAAGGATTCTTGATCCGTACAATCATAAACAACAATAATACCATGAGCACCTCTGTAATAAGATGAGGTTATGGTACGGAAACGCTCTTGACCAGCAGTATCCCAAATTTGCAATTTTATGGTTTTACCA

>BPA_111

AGTAAAAAATTCCAAGAGATTTTGTTGGACAAGAGATCGACTCATTGTGTTAACAAAATCAGCAACAACAGCAACTAAACGTTTTTAAATTTTATTTGTGTATCTTTGTCTCGTCGTAAGTTAATTAAAAAACCAACAAATTCAATATGAGCTGGCAAGATTATGTTGATAACCAATTGATGGCCTCGCAATGCGTTACCAAAGCCTGTATCGCTGGTCATGATGGCAACGTTTGGGCTTCTTCCAAAGGTTTTGAGGTTACAAAAGAAGAATTGTCAAAATTGATCTCTGGATTTGATAATCAAGATCTTCTTACCAGCAATGGCGTAACATTGGCCGGCCAAAGATATATCTATTTGTCCGGTACAGACCGTGTTGTCCGTGCCAAGTTTGGACGCAGCGGCGTGCATTGCATGAAAACCACACAAGCCGT

>BPA_113

TTTAAAACAAAAAGAATTCGATAAAATTTACTTCGAATTAATGGCTACTGAATATACACGATTGCTTGAGGAACATAAGCCGCAGATTATGGAGGATATATCAGATCAGATAAGAAGTTGGTTTAAAGAATTATGCCACAAATTCTGGAACGATTCAGATGTTATTAGACGAAACTATGGATTTGGATGAATTTAGGCAATATAAAATGAATGAAGCTTTATCTAAAGAGGAGAAAAAAAATTTGATGGAAAAACAGAAACTCCAAGAGAAGAAAGAGAAGGAAAAAATGAAAAAAGAAATGCAGAAAGAGAAGCTAAGACAACAAAAGATGAGTAATGCAGGAATATGTGATATTGGACAAGAATTGAGAGACAATAATAATGTGGGGAAAATTGAAGAGACTTTTTCACAATTTATAACCGACTGGAAATATATTGATGAATATCTAAATAAAAATAATGATCTTGTTAAAGAATGGGTCTCTGAAGATGAACTTGTTATTATTCATAAAGAACTACGAGGTCTTGTTGATGAATACATGAGAATTGAATATGAATTATTAAAAAAAGCATGGAGTCTTGATAATAAAAAGAAATATAAGCCAATAAAGCAAAAGGGAAAGAAAGTGAAAAAAATAAAAGTGAAGCCAACCAAAGATTTAACGGCAAATCGTACGAATGAATGTTTATATCAAGAGTTAAGAGAAGCGAAAATAATTGAAGATTATGCAATTAAAAGTTTCAGTGATTATGTAGGAGATTTTAATGTTTTGGCAGATGATTCTAGGGATGAGAGTCATATTGTTACTGTAAATGCGGCAAAAGCCGATATAAAATTCGCTATACAAGACTGTATGTTTGGTATGGGAAAATTTAATGTGGATAAGCCAAAGTCTTTATGTTTAATTGGACCGGAAAAAAGTGGAAAAAAGTTATTGTGTAATATTATTGCTTCTGAACTTGGTGCTATTTTTATGAACCTAAGTCCAGAGAAAACCTACAAGTTTAATGGAAATCTGCAATATTTAATAAATGTGGTAATTAAAGTGGCCAAAGCTTATCAACCTACAATTATTTTTATACAAGATGCTCACAGAGTCTTTTGGAAAAAAGTTCCAAAAGATCAAATAGATATAAAACCAACACTTT

>BPA_115

GAGTACGATACACATATTGTTGTTCACATTTGGCGGTAAAACGACTAGGTACAGTGAGTCCATTTCTTACGCCGCATGTTTCAGTGTCCCGAGAACTGCACACTTCAATTACAATACCTTGTTTAAATTTATCGGTATTAATAATAATAAACCAATCTCCATTAGCAGCTGCTTGTGGGGTTATAGTTTCAACATGATTACTGCATAAGTAATCTCTTACTGTGTTATTATTTAAATATTGTTGCATATTTGCTGGTGGCAACTCGAATGGTTCAAACAATTCTGTCATACCTGATGATTCAATTAAGTCATCAATTGCATGTGTTTCTGGGTAATCACGAATATTCTTACAGAATGGACTAACTAGTGGATCAAAATCTTCATCTAAA

>BPA_193

TCACCAGCATTGGCGGCTGAACCGGCAGCTCCAGCAGCAGCGGCACCACTCTTATTAGCCAATTCGCTAGCAATCTTTTTCAATTGTGTGATATCTTGGGGACCCAATTGTGTTAAAATACCTGGTAACATTTCTGAAATCGACTTGTTCTCGCCATGTCCAGTAATGGCAAAGGTGTTGGTGGGCAATGAAGCTTGAGCCTTAGGATTGTTAAAGTGGATGACAGTACCATCATGTTTTATAATGTTGACCTCCTCAATGCCGGGTATAGTGTTTACCGATAATTTTTTCAATGATGACTGCAATTTCTTATCGTCTGTAGCGGGGGTGGAGTGTACGATCTTCTTCTTACGGCGTGGTGTACCCTTACCACCAATACGCACTTGCGCTTGCAATTTTTTCAATTTTTCGGCATTCATATTTTTATTTGATTTTTTGCGGACGGTGCGTGAGAAAACACGTGCGCCAAAAGTAGAGTCAACTGTCAAAAAGAAATCGCC

>BPA_225

GTCAAATTGCCGAAGATCGTTTGTTGGACGAAATGAAAGGCAAATCTTCATTGGAAGAAAAGAAAAAGAAGGTTAAGGGTATTTTGATGCTTATGCAACCTTGTGACCATATCATTGAAATTGCCTTCCCATTGCGTCGTGATTCTGGTGATTTCGAATTGATTACCGGCTACCGTGCCCAACACTCTACCCATCGTATACCAACCAAGGGCG

>BPA_236

ACTCTCATGTGTGCCATTTTCAAGAATAATTTCTCCACTGTCGGCAAATCTAAGATTTACACCAGAAGAAGACGAATTTGTTTGTCCTATGCCAAGTGTACTGGCTGTCATTTTATGATGCGTCTCCAGTAAATTGCCATCTAAGCGCATTGTTGTGCTTTCTGGCGTATTTGATTCTGTTGTAGTAGCTGACTGAGTCTTCGAATTTGATGAGGCACATGAG

>BPA_281

GCAATATCATAAAAACAACTATATATAAACTATTTTACGAGTAGTGAAGATCAGTATGAACTTCTCCATGCCTTCTCTTTGAAACTAAATCTTGATTTCATTGTGTGCGGTTCAGAGTCGGTCTGTAGTGGACATCAGCTATATATTCTTTATGAAACTTCATATCGAGTTCATACACAAACACTTCATGATGATCAAAC

>BPA_286

CTAAAGTCTAAGATGGGATGGTTTAAAGCTTAATGAGGAGCTGGGGATGACTAGAAGTCAGAAACGCGTCCTTTACGTTCCCAATCACCATAGCGAGTTGGCTCGGGACCAGCGGGACCACCAATTTCACCAGTGTGGGGATTAACATTGTTGGGCCATGGTCTCAAGGGTTCAACTTCTTGGAAGGGATGGCGAGAGAATTCATC

>BPA_298

GAAATCCAAGCGCAAGGTTGAAGGTGACTTGAAGCTCACCCAAGAAGCCGTTGCCGATTTGGAACGCAACAAGAAGGAATTGGAACAAACCATCCAACGCAAGGACAAGGAATTGGGTGCCCTCACCGGCAAATTGGACGATGAACAAGTCGTCGTCAACAAGAACAACCGTCAAATCAAGGAATTGCAAGCCCGCATCGAAGAATTGGAAGAAGAAATCGAAGCTGAACGTCAAGCCCGCGCCAAGGCCGAGAAACAACGTGCTGATTTGGCCCGCGAATTGGAGGAATTGGGT

>BPA_304

AATTTTTAGGTGATATACTTAAAATAAAACCAAAATCAATATGTATAATATGGCCATCGGAGTGCAGCAAAATATTGCCATTATGCCTATCTTTAACTTGCAACAGATACGATATTAAACAATATGCTGCACAACTTTGTACGAAATTCTTTTGAGCCACACGGAACGCATCACTATCTGGAGCACCATATTCATCAATGAAATAATCCCTTAAAGATTTATTGGAATTCTTTTTG

>BPA_305

CGCATATTTTCCTGAAGAAAACTAAAAATGCCAAAAGCTGTTTGTGTTATCAATGGTGATGCTAAGGGAACCGTATTCTTTGAGCAGACTGATGAAAATTCTCCAGTAAAAGTAACTGGTGAAATTACTGGTTTGTCTAAAGGTTTGCATGGTTTCCATGTTCACGAATTTGGTGACAACACTAATGGCTGCACCTCAGCTGGACCACATTTCAATCCAGGTGGCAAGGAACATGGTGCCCCTACTGATGAAAACAGACATGTAGGCGATTTGGGTAACATTGAGGCCAGCGGTAATGGCCCCACTAAGGTTGATATCTCCGATAAACAAATAAGTTTGTTTGGAGCGAACAGCATTTTGGGACGTACTGTTGTTGTCCATGCTGATCCCGATGATTTGGGTAAGGGTGGTCACGAATTGAGCAAATCAACTGGTAATGCTGGTGCTCGCATTGGTTGCGGTGTAATTGGCATTGCTAAAGCTTAAATCAATTGATTAACA

>BPA_310

TCCCACCTCGAACAAATTGGTGGCTAAAAATGAAAAAATTGACTTGGATGTTGGCACCGGTTTGTATCGCACTGTTACCGGTTTGAAGAAGAAATATCCCCACTTGAAGGTTTTGTTGAGTGTGGGCGGTGATAAGGATGAAGTTGACAAGGATAATAACAAATATTTGACTTTGTTGGAGTCCAGCAATGCCCGCATTCCTTTCATTAACAGTGCTCACTCTTTGGTTAAAACTTATGGTTTTGATGGTCTTGATATTGCCTGGGAATTCCCTAAGAATAAACCAAAGAAGGTTCATAATAGTGTTGGTAAATTCTGGAAGGGTTTCAAGAAAATCTTTACTGGCGACTTTGTTATTGATGAGAAGGCTGAGGAACATAAGGAACAATTTACTGCTTTGATCAGAGAATTGAAGAATGCTTTGCGCCCCGATGGTTATCTGTTGGGTGTTTCTGTGTTGCCCAATGTAAACTCTTCATTGTTCTACGATGTTCCCGCTATTGTAAATAATTTGGATTACGTCAATCTTATGGCCTTCGATTTCCAAACCCCAGATCGCAACCCTGAGGTAGCTGATTTCCCTGCACCCATTTATGAATTGAATGAACGTAACCCCGAATTTAATGTCAATTATCAAGTCCAATATTGGTTGAACAATCATTGTCCCGCCACCAAAATCAATGTAGGTGTTGCTGCTTATGGTCGTGCCTGGAAAATGACCAAGGATTCTGGTCTCACTGGTCTACCACCAGTATCAGATACCGATAAGGCTGCTCCTGCTGGCGTACAAACCCAACGATAAGGCTGCTCCTGCTGGTGTACAAACCCAAATTGAAGGTCTCTTGAGCTGGCCTGAAGTATGTGCTAAATTACCCAATCCCGCCAATCAACACTTGAAGGGTGCTGATTCTCC

>BPA_314

GTAAATAGCGTTGACGAGCTGCTGGACCCGATTTAGGTTTAGAAGAAGATTTGTCTTCCATTTTCCGTTCGTCCTTAGAGGCAAAGTTATTTACAATTGAACTTAAGCCCGCTGTTTTGCTTTCTTGATAAATTTCTCTCTTAATCTCCTTGCGCTTCTCTTCCACTGTATTTTCAGTGTATGTAACAAATTCACTCTTAGATTTCTCCAATACATTAGTGCTGGCTTGCGTAGTCCCAGATGAGATTTGTATTTTCTTTGGCTCATTTTGCTTTCTGTCAACTTCATCTTGTTCCTCATCACCGGCATTGGCCTTGGCTATAGAACGTTTTGAATTACGATGCATTAAACGACGCAAGAAACCTGTTTCTGTAGTTTCCATGGGCTGAGAACTTTTATCAAACACTTCGGGCTTCTTAGAGGTCTCAGCCTTCCTATTACCTTCCTCGTTTTGTGGATTATCTTTGGATTCAAACATGGCGCTGGCTTTAGCAGTCAAATTACGTAAGCCAAATATGTTAGTAGTAGATGATGATGTTGAAGTAATATTCGAAGTGTTCGTTGATTGTGTTGTATTCGCATGGGAAATTTTCGTAGTTTTGGTTATGGACTCCTTGGTGGCAGTGTGTGGAGGTTCATCCATAAGTTTAGCAGCATTAACGCCCGGTGGGAGAGAACGAGTTTTAGCTTTGGTATCCAAATCTTTCACTTTCGATATTTGTTTGTTAACAAATTTGTTTATATCTTCATTGGCCTCTGGTATTGAAGTCTCCAGGGTCCTTCTATGTTGCCGGCTAGGCCCTCCCTTTTTCTTTGTCGGCCTTATGGCCATTTTGTGTTTTGCAGCCGAATGTGACAATCTTTGCCAATCGGATGAAAACAAATCCACTTCATCTTTAGTATCATTAAATGAAGAATCACGATGTCTTAAAATATCCGATGACACTGATATACTCGACATGGTCGATGATCTTGAGTATCTTGTTACTGTGGTACGTTCCTGTACAATTTCTGTTGTAGTTGAATTATTTGCCGTAGATGTTGAGGTGGCCAAAAAATCGTCACCATCATTACTGTTCATACTCAATAAACTCAGGGAGGATACTTCACTGTGTTTTTCACCCACAATCGATTTATTACTCGCTGAAGCTCCTGTTGTAGCATGATGCTGTGAGTGTGCTGCCCGTCTGTGTGGTGAGGCTGGGCTACGTGGTAAACCCAAATCTTCATCTGAAGATGTGGTGAGGCTGGGCTGCGTGGTAAACCCAAATCTTCATCTGAAGCATCTGGACGATTGCGGCGTTTACGTAAGACATCAGCAAGCTCGGCCTTTAAAACTATAGACAAACTGCTGGCGGTTGCTGATTCAGAGAAAACACTATCATGCGACTGGGAGAGACCATCGCGAGAATAATCACTGTCAACCAAACGACCCTCACTTAAACTACGTTGACTGTCTGACATTTCTCCTCCACTGGCACTGCCACCATTTTTAACACGTTCCGTTGCTGATGAATTATGACCACCACCAGTGCCACTGTCATTGCCACTACCACTATCTAAATCCTGTGATGAATCACCTACACTACGTAATTTTTGACGTTCTGTAAAGAAACTACGATTTATGGCGCCTGGAAAAACGGCCACACCCATTGATTGCCGTTGACTCAAAAGTGAAGTTTTGGTGGTTCCCCCAGAACCCGTTGCAGTCGCAGCTAACGATGATGATGTCGTTGATGAATGTGCATGATGATGGTGATATGTTGCTGATGTTTGTGTTGTTGTACCAAACATATCTTCGGCTTCTTGTTGTTGATGTCTCTCAAAATCGTATTCTTTGGATTGTTGCTGATGTTTTGGATTCTTATGTTTATGTTTAGATTTTGTTTTAGTTTCGGCATTCAATTGATATTCATGACTTTGATGTTTCTTCCCACCACCCAAGAGAGAACTGTATGCGGATTTGGATTTTGGCAAAGATTGTGTCAAATAGGCGTCCTGTAATTGTTGATGTGTTAGGGGTGAGGAAGGCGATGATGCCGATGCCACTGATGTGATTGATGAAAGTGTAGCACTACGTAAATTCTTTTCACTATTACTACTAGCCGTAGTACCTGTTATAGTAGAGGTGGGCGTATCACCGGCAGCTGTTGTTGCTGAGGTTATGGTGCGTCTACGAAAAATGCGTCGCAGATTACGTAGCGGATGAAATCTTCGTCTTTTGGTTTGTTCGGTATTGGGTGGTGTGGCAGTTGGTGTCAAACCTGCACTGGTTCCACCACTG

>BPA_370

GATAATAAAGCAACAACAACTGACAATAAAGAAACTTCAGCAGCTTCAGCAGCATCGTCACCATCGGAAGATAAACAAAATTTACAAAATTTCAGATGTCCAAAATGTGAATGTAACAATAAAACAATAATTGAGGATTCCGATGTGAAGGCAGAAGTTGTTACAAAAGAGTTACCAGATACTCCAGCTCCCATTGAAACCGATAGTGGCAAAAAGGAATCTTGTAATCT

>BPA_455

CACAGAATGGCAAAAAAGAATTTTGTTCGGAAACCTGCATAGCAGAATTCCGCAAAGCTTATAGTAAAGGAGCCTGTGTGCAGTGTGACAATGTTATACGGGAAGGAGCTCCCAATAAGGAATTCTGCTCGTTAATGTGTATGAATAAGCACCAAAAGAAAAATGGCTCTACACGTTCCCTCAACAATGGCACACGTATGGACGGTTCGAATGGTGGCAAAAATTTAG

>BPA_459

TTAGTGAATCTTGTGGGTATTTTTGAATTGTAATATATTTCTTGGGGTTCTTCCATGGAATTGAAAACATAATCAAAACTTGGTTCATTTTCTTGAGAATTAACAAAAGAGTTTTGATTCATTATCATTCTATCCGAGAATGGAGGAGAAGCCACTCCCACTTTAAACATCATCATATTTCCAAATTTCATGAGCTGATTTAAATTAGCAAAAGGATTTAAATTTCCATACGACTGGTGAGTACG

>BPA_496

CCTTGGTAGCCTCCTCATGTAAATCTTTAATTAATGCTAAACGCTCCTTTTCTATTCGCATTGAAAAAGCTTCCAATAATGTCATGGGATCATCTAGAGGAGGTCTTTCGCACAGACTTACATCTCGAGTATCTTGGTTTAAAGTTATATTAAATGAAAAATCATTAAAGTTTTCCCGACCATCAATAGTATCCTGTATAATGCCAGGACAATAAGCATCTTTAACTAAATTCTCGATTTCAACTTTACGTAAGGCCGAGCGTTGTGTGTCTTGAACTTGAGATTTATCTGAGCTATCAAATTGTACACGACCACACCTTTTGGCATTGGCGCATTCTTTTGTTGAAGGAGTTTCGGGTCGTAATTGCGTTTTATCTCCATAGAAAGACGGGTCGTAATTGCGTTTTATCTCCATGGAAAGTACTATGACCTAAAGTTGTTACATTGGGACACTGTGCGGTACAGAGATTTCTTAAATTATACAACGCTGATTCCTGGGGACATCCTTTATATAGCATACTTGAGTCGAAGACCTGTTCCAACGTTTGTTCGGTTGGTAATGTTAAAGTTGCTGTTGATTTCTCTGGCGTTACGAATGATGAAATAAAATCATATGTAGAACTTAATTTTTCAGCTAAACAGCGAAATAAACTTGATTTAGGTTTATCGCAAGGTGAACAGGTTGTTGACAACGGCTGCGAAACTTCTTGTGGTTTTAAAGCCGAAAGTAATTCTTGGCACTGAGTTATATTTTTTGTGTTTGGTACTAAATAAGTAGAGGTAAAATTGTTGGGTTGGTCACTATAAGTATTGTATCTATAACTTTCTTGACAGGTAATATCTTCTGGTGGAATTCTCCCAGATTGCATAAGTCTATTGTCTACTTGTCTCTGGGGATAGGCGGAACGGGGTGTTAAGTCTGGTGCACTTATATCCATATCACATTGATATTGAGTTTGGTTTGTTAGATCTACATAACAATTGTCGGCGGGCTCCGTTTGTAGTTTTCCATATGAGTTTGGTTCATTAAACAAAGACATATTATGATAATAAGACTCTGATGTTGGTGTTTGAGATTTATTTGGTGTTTGCGTTTTTACTGGACTAGGACATATTCTACTAGGTAAAGGCAAATAATCTTCTTCTATATTATATGTGTCATCGTAATTTTTAGAAACACCAAAATTTCTGCATTCAGCAGGAATCTGGCCTTGTCTACTTGCATTTATGGAATGATAATCTTTGGTAAAGTCTCTACAATGTATCATATTTGGATCTGGTGGGGGTAATGGAGGTGGAGAGGGAATTGTTTGCGTTTTATTTAAAATAGAAATATTTTTATTTGCCATAGATCTTTCCTGCTGTATAGATTCTTCAGTTGCATAAGGGTCAATGGTATCGTATGATTTTTGAAAACTCTTTTGAATAGATGGTTCATATAGTGGTGTCCTTGGTGTCTTACTGAAATTTTGAAAGTTGAAAGATTGTGGTGAGGTTTGTCTATAGCAACTGTTGTCTTGAGTAATATTAGAAAAATTCTTAGGTCTTGATATTTGATCGCTAAAGGGTTCCAAAAAGCTATCGGTAGGTTTTTTAAAATACGAAAGATCTGAGGAAGGACCAGTAGTTGTCGTTGTAGTCCTTATAGTTTTAGTTATACTGATTGGAGTATTCAATGAAGATTCGCCTAATGGGCACACCGGTGTTGCCGTTGTCTGGTTTACCGATGTTGCAGGATCTGTAGATTTATTTAATATTTGCTGTACAGGAGAAATTGAAGGAGAAACAGATCTTATAGGACTGCTGCTTACGGATTGAGGTCTTGGACATATTCGTGGCACACGTTGAATCGGAGAATGAAGTGTAGAATATTGCACGGGAGTGGGACATGAAGAGGCTCGATGTTGTTTAGGTGTCGGCAAATTTTTACGACGAAATGTTAAATCCCTCTGCAAATCCAAGGGACGTCCAGTCCTGCCGGTGGGA

>BPA_497

TTTTTGCAATCACTGAACATATAGTGTATTAAGCACTAAGACGTGATGATTATTTTTAGCCAAAGTAACAACATAACCTTCCGATGTAATTTTCAAACCACAACAACGCGAAACCTTGACATGAGGACATTCAAATTCTGATTGTAAAATTCCATCACGGGAGTAGCAAGCAACGTGGAAACGGTTGCCATGAGAATCTCCAATCAATATGTCACCTGCATTTGATATATCGATTCCATTGGGGAAGCAAGTAACCTTTTCATTACCAATACGATATTGGAATGTGCCGTCCTCATGGAAAACTGCAACACAATGGCCTTTAAAATCGCAAACGTAAAAATCGTTATCTCTGATTGCGATATCGGAAGGTTCACGCATGTAATCGCTGCAATCAAACCAACGTACTAATTCGCCATCTTCTGATATAACAAATACAGTGGGTGAAACACTATCGACAGCAACAATATGACCTTTAGATGTTACAGCCAAGCCAGCCACAATATCTATGTATCTGATGGCTATTTTGCGCATAAAGTGACCACTCTTGGAAAATATTTGCATACGAGAACGTTCATTTCCACGATCGCATACTACAAATTTACCATTAGTATGCATTACAGCAACTTTACGGGGATACCATAATTGACCCTCCTCTTTTCCTGGTACTCCAAAGTAAAATTTCAAATTGCCTGATTTATCAAAAATCTCAATGCGATGATTGTTTGTGTCAGCCACAATAATTTCCTCATCTACACCCAAACAAAAGCCATGTGGCGAATTAAACTGTCCCTTAGATGTACCCAATGATCCAAACTTGCAACGTATTTGCATTGGTGTGGCCTTATTGCGACACCAGTTATTATACTGGTTGTATTGGGTGGTGGTGGTGGGGCATGGAAACTTGTAATACTCATGTCATCAACGGAACCACCACCATTGCCACCAGGACACAATGGCGGTAAGGTGGGTAGACTTGGTGATGGCGCTGTGGGTAGGCAGTAATCGTTGTTATTTAAACCCAGTTTAGCTAGAGCTTGTAGATTATTAAAAGCACGTTGATCACCAGAAAGAAGATCGGCTAAAGTTATGTGTTGATTTTGAGAATTAGCCGTGGGAACAATCGACTGATTCGAAGGTACAGCAGCTGATCCAACTAATGCATCATTAAGAGTATCAGGTGATTCGAAGGTACAGCAACTGATCCAACTAATGCATCATTAAGAGTATCAGGTGTATTTTCAGTAAGACTGGCAAGACGAGATAAATTGTATTCGAGTATACTGTTGTATCCACCATTAGGTGGTCCTCCACCGACACCTCCATTTCCACCGAGAGTTCCATTTAAACCAGTAGCAAGACTGTTATTCAACGGATTATTCAATGAGTTTAGACCATTAGTAGGCCCGGTGCCATTTAGAGCATTTAAACCTTGAGGACCCATAATTGGTCCACCACTAAGACCATTGCCCAACTGA

>BPA_498

ACCAACTGAATTAACACAAACACAACTAAGTCATGAGTTTAATGAAAATGTTCAAACCGGAAATCAAACATTGAATACATCATCAAGTGCTCCTTGTGCTTTGGTCAATAGTAATAGCAGTCTGGAAGATAATTGTAACACCACAGTAATTGATCAGCATAATGTCACAACCATTGTAATTGAATCATCGTCATCATTAACACCACCTTCATCATCAGCAGCAGGACATTTTGTTGGCCTAGAAAATAATAAGGTTAATGATGCAACAGCTAACAACACAACATCAACAACAACCACATCAACATGCAACAGCTAACAACACAATATCAACAACAACCACATCAACACTAACATCAAATACGCAGGCTGTTTTATCAACTTCTCAGAGTCTAAATGTCATCATGGATAGTGCTGATATATTGCGT

>BPA_500

AGATCTTTACCTTTTTGGTCTTTATCTTTCTGCAACTTATCTTTAAGATCTTTATCTTTTTGTTCCTTATCTTTTAGATCCTTATCTTTATCTTTTAACTCTTTTTCTTTTAGCTCTTTATCTTTCGCATTCTTATCTTTCGTCTCCTTATCTTTATCCAACTGCTCTTTACTTTTGTCTTTTTCTTTCTTAACGTTTCCCTCTTCATCTTTTTGTTCTTTCTCTTTTAGTTTTTTATCTTTTTCTGCTTTTTCATCATGTCCTTCTTCGTCTTCCGATTTTTCCTTTTTGATTTTCGTTCGATTTTTAATATTTTTATTCATCTTATCTTTAGATTTATTTTTTCTATCACTTTTACCTTCATCTTCAGAACTCTCCATGTCATCTGCTTATATCACTTTTGCCCTCATCTTCAGAACTCTCCATGTCATCTGCTTTTTGTTTTGACTTTTTATCTCCTCCCTCTAAACTATCACTAGCACCTCCACCTTTTTTACCTTTACCAGAAGGTCCTCTTTTACTTTTGCTACCTTTACCTCCTTTTCCTCCTTTACCTTTGGCTTTTCCAGACTTTCCGGATTTCTTTTTATGTCCACCTCCCTCTTCACCGCTCGAAGAGGTTTCAATACCTTCTATTTCTTCGACTTCTCCCTCCCCCATAGCTTCCTCTTCTTCCACTATTTCTGCTTCACTAGATGATGTTCTCAGTTCCATTTCAAATACATCCGATCTTAGATCTTCATCTTCATAATCACTTATATCAGCTAAAGCTTTAAAGCATATATCTTCGGCCTCTTCAAAGCATATGTGATCATTAAGTGATATTGTTTCACCTTTCGAATCCAATTTCGAATCTTCAATAG

>BPA_521

GCAGCATTCGCACCCTCAACCAAAATTGTTTTGCCCTGACGTAATGCTGTGTGCAAGAAACAAATGGTATCCTTGACATAAGGACGTAACTTCTCAGCATATTCTTTGTAGCGCAATAGTTCAGAATCAACATCAACCTTAATGGAGGGAAACAAACGCAAATGCGTGGCAACTATAGATTTGAATTTTTCACTGAAGAGATTAAAATCACCTAATAGCTCAC

>BPA_538

TGTTGCACATGCTGTTGGTGCTGTTGATGTTGATTTGCGTTTGAAGTTTCTTGCTGTTGTTGTTGCTGCTGCTGTTGCTGCTGTTGTTGTGGACTTTGTTCCGATTCATAATCATTTGTTATAAGCGTCTTTTTAGGCTGTCTCTTACGTGGTGTTTTTGTTGTTTTTGTTACCACCAATTGTTGTTGATGTTTCTCCTCTTGGGCCACCTTATCCGCAGCTAAATTAAGCTGTTGTTGCTGTTGATGTTGCTGCTGTTGTTGTTGGCTTTGTTGTTGTTGCTGCTGTTGCTGAGCCTGTTGTTGCTGTTGTTGTTGCTGTTGCATTGCCAAAAATGTATTCGATATCATTGATGGTGTATTCCAACTCAGCGTAGTCGGTGTTATGGTAGAGGTGGTGGGTGAAGGTGAAGCACCACTTGATGATGAATTTTCACCATAATA

>BPA_595

CTCATCAAAACGTAAACTGCGACAATAATTCAAGTACATCGAAAATTCTGCGGGGAATCCCTTGCATAATACCTCTATGGGAGTCGACATTTTCTTTTCTGAAATTTTTTCATATTTTTGGGTTTTATTGTTGGCTTTCATTCCTTGCCAAGGCAAAACACCACGATTAAAATACATCATGACATAACCCAAAGACTCCATATCATCT

>BPA_596

GTGTACGCAATTTTGCTAGCAAAGGAAAAATGGGTTTACCTCGTGTCTTTTTCGATATGACCGCTGACGGTCAACCTTTGGGCCGTATTGTCATGGAGCTCCGTAGTGATGTTGTTCCAAAGACCGCTGAGAATTTCCGTGCTCTTTGCACCGGTGAAAAAGGTTTCGGTTACAAGGGCTCCTCTTTCCATCGTGTCATTCCCAATTTCATGTGTCAAGGTGGTGATTTCACAAATCACAATGGCACTGGCGGTAAATCCATCTATGGCGCCAAGTTCGCTGATGAGAACTTCACCTTGAAGCACACTGGTCCTGGTATTTTGTCCATGGCCAATGCTGGACCCAACACCAACGGTTCTCAATTCTTTATCTGTACCGTTAAGACCGCTTGGTTGGACAGCAAACACGTTGTTTTCGGCCAAGTAGTTGAAGGCATGGATGTTGTTAAACAAATTGAAGGTTATGGAACTCAATCCGGCAAGACAAACAAGAAGATTGTTATTGCTGATTGTGGTGCTCTGTAAGCGAAACGCTACCGTCA

>BPA_633

AAATGAAAGGAGAATAAATGCAAACTGTTACCACCAGGATTTTCAGGATGTTTTATAAACTGCTGTTGCCTACACCATTGTAGAACAGCATATAACTCCTTAAAATGTTCATTTTCTATTTGCACATGTAAACAAGACCAAGAACGCCACTTTCTGGTCATGCACTTAAGCAAGGCAACACCCATAATCTCTTTATTTTCATTCATAACCACTAAACT

>BPA_641

TTGCGTAGATTAAATACTACCTTGGGATTGGAAGCCTTGCCAGTTTCAAAATCATAATCATATTCCTTAACCTCATAATCAGTGGTATCGATATAATAGAATTTATTGGTTTTCTCATTCCAAGCCAAACCATTAGAGATACCTACATCCGATTTAATTACCTCAACCTTGCCACCCTTTTCATATTTGTACAATTCACCATAACGATGTTCAAATTCATCACCCACATAACGC

>BPA_657

CGGAAGTCGTTTCTGGTCCAACAACATTTGAGGGTGGTGGCACATAACGTTTTTTACGATTTTCTGGTCCTGTTGATCCCTGTCTCACCATACGTCTTCTAATACCCGCTCCACCAGCACCAGCTCCTGCCACTGGTGTTCTACCGCGACCACTACCACCCGATGGCTTGGTTAGCAAATCTTTTACTTCACTTTTACTTGCGAGTACTTCAC

>BPA_658

GAATCATCATGTCGACTGGGTTAATGACATTAAATCTTATTGTTTGGATATTCCCGGTGAATTTCCTTATCCCATTATAGCCGATCCTTCACGTCAATTGGCTGTTGAGTTTGGCATGTTAGATGAAGATCAAAGAAAAGATCCTGAAGTGGGCAAAACCATACGTGCTTTGTTTATCATAAGTCCCGATCATAAATTGCGTTTGTCCATGTACTATCCCATGTCTTGTGGTCG

>BPA_669

TTCAGCAGCGATGGGCTTTTTGTTGCCAATGGGAATGGCTACTTTGATCTTTTCGGTAACTTCAGCAACTTTGCTTCTGGCCAAATCCAAGTAAGTGTCAATAGATTGTTTGTTGTTTTCATAAACCTTGGGCAAGGTGAACAAGGAGACAAAGGCCAAAATAACCAATGTCATGCCATTGAACCAAGCTCCAATATAAGTCATAACCCACAAAATTACACCGAATTTAATGGAATCAACTAAATCCTCAACCAAGAATAATCTTCTCAATTCAGCAATGAAGCCATTTAAGTGGGCAACAGCAACACCAGCAATATTTTGAATTTTCTCCTGGGACAATGTTAAATCGAATTCCAAATATTCTTTGAAGGGATGACCATCTGAGGTCTTTTGTATGGC

>BPA_674

CTAAGTAGTATTTTAATCACAACATGGATCTTACTGGAAAGAATGTTGTATTCGTAGCTGGCTTAGGAGGAATTGGTTATGAGGCCTGCAAGCAATTAATGACCAGGAATCTTGCTTATTTGGTGATTTTGGATGTCATCGAGAATGCAAAGGCTGTACAAGCCTTACAAGTAATGAATGTTAAAACTAAAGTCATCTATATGAAATTTGATGTTACCGATAAAACTAACATACAAAACACGTTTAAACAAATTGTTAACACAATAAAATATATTGAAGTATTAGTAAACGGTGCTGGAGTAATAGCAGACCGCAATGTTGAATTTACTATCAATGTTAATTTAATTGGCCTTATTAATACTACATTGATTGCTCTTCCCTACATGGATAAAACCAATGGTGGAAGAGGCGGTGTAATACTGAACATTGCTTCCGTGTTGGGATTGGAACCATGTTCTCCAATTGCTATCTACAGCGCATCTAAGTTTGGTGTTATTGGATTCACTCGCTCCCTATCGGATCCTTATTACTACAATCGCACTGGTGTTGTAATAACTGCACTTTGTCCAGGTCTTACCGAAAGCCCAATGACAGCCAACCCAAAAATTAGTGATACTTTTGATTATTCCAAACCTTTAACAGATCAAATATTTTCTGCACCACGACAACCTGCTGCCAAGGCCGGTGAACATTTGGTCAAAATAATTGAAATGGCTCAAAATGGAACAATGTGGATTAGTGATAAAAATACAATGACAAAAGTTGAACCAAAACTTTTCTGGCAGCCTTAAAATGTAAATTTATAAAACATTAAA

>BPA_710

CGTTTTAGTAGTAGTGGTGGTCCAACAACGGGCCTAGCTAGCTGTAGTACTGGTAGCAGTAGTAGTGGTAGTGGTGCTAGTTGTGGTAGTAATAATGGTGGTGGTGCATCTGGGGGAGGAGGTGGAGGAGCATGTGCGTTGCCATTTGCAGGCGCTTCCACTAGTAGCGGAAAAGTTAGCAGTACATTGGGTCCAATTATAAATTTGCC

>BPA_730

TTTTGACCAATTGCCAATCTGTGGTTAATTTTGTAATACGTACTATTAGCTCTATTGAATGTCAGGCTATTTTTCTTTATAGCAAATGTGCTACAGCTGCTAAAGCTTTAAGTCGAGTAGAAATCTACTGCCCAGTTTTTGTAATTTTGGAACTCGACAATAAAGAAGAAGATATTAATTGTGTTGTGACACTATCGAAAAATTTAAACCTACGAAGA

>BPA_752

TAGGACTGTCGCAATTTGAAGTCATTATTTTGACCTTTGGACACATGACCACAAGAACTTGGACAGGAAAAATATGGTATAGGCCCCCAACAATATGTAAGACTGTTCCAAAATATTCGCGTTACTTGCTTCGGTTGTTGCAGCAGCAGCACCTTCATCGTCTGCCATCTTTATTTATTTTATTTAGCGTTTCTCTTTAAAA

>BPA_753

ACAAAAGCATCATATTGTTCAGCAAGTTTTGTAGTCAGTACAGCATCATATTTTTCACGCAATTCATCTTCACGCTCCTTTAGCATACGTTCACATATTAATTGCACCTGTTTAAAAGTAAATAATGCCTTTTCTGGATGACGGACCATGCTGGGTGGGCTTTCTGGACGTCTAGGACTATCTGGTCCCATTTCTGAACCACTGGATTCTGAGTCTTGCATACGTTCAATAGCGGCCGAGGTAAAAGGCAATTGTTTGCGTCTGTGCAATCTTTTGATTTCATCACGGATATTTTGAGACATCTTATCTGGTGACATTTTTGAGAGATTATTAGCCTCAGCAAACGGACTTGGTTGTGGTTCCTTGACGAAACGTGTTGAAGGTGATTGAGAATTTGAGCAACCAGGTACATTATCATGGCCAGCCGATGAACGTGATGGTGAACCGTTGACG

>BPA_756

GGTTGTACCTTAATTGATAAAATATTATTTTTAGCCGACTTATCCGGATTTGTGGGTGTATCCTCAGCTGGTTCATCTAATTTTAAACTTTCTACATCTAGTTTGGGATCTTCTTGGGGACCATTTCCCGAGGAACCATTTCCCGAAGCATCATTTACCTCATCAATATCAATATCGGCGGAATTTTTATTTGGTCTTGCTGTGGTGGGTTCTCTTTCTTTTTGTTGCTGCTTACCCAATTTAACTTCGCCTGATCCTGAGCCCATATCAAAGTCCATTTGTTGTTGGGTTAACTCCACAGATTCCAATGAACTGTCCTCCATTTTAACGGTCTTTGCATTATCCTTAAAATCTGTAGAATTAGCAATTCTGGTTTCCTCCGAAGAATCTTCAGCCGAATACATAAGTTGTATAACCTCGGGTGCTTTAAGTTTATCATTATTTTCAGTTGACGATTGCAATTCACCACTGCCTTCTGTGGTAGCAGAGGAAGCAGCAGAGATAGCTACTAATTCATCGATTAACATGCCACTACCACTGCCGAGATTAACATGCCGCTACCACTGCCGCTACCATAACCAGTATCATCTAAATGTTTAGTACTCTTGAAAGCTTCATAGGTGGCAGCTATAGCATGGCTGCCTCCTATGCTTCTAGGTTCATCACCTATGTCATCTGAATCATTAATTTGGAAATTGTTGTCTCTTAAACTGAAATCATCTTCTGGCAAAGAAGGATAAGCAGGATTGCTTTCTAAAGGTATAGCAGAATCCTCATCATCTAAATGTTGATGCAAAGCGGACTCGGTAGAGGGTTTGCTGTAATAATCCTGTTTAGCAGGAGCCTCTGTGGTAGATACCTCCTTGTTATTATCCAAATTGTTATTAGTTTCCACTTCTTTTAAAACACCAACCGATAAATCCTCAAAGGGAGAACCCTCTGTCGTTGAGACCCCTTTTAAAGCCAAATAATTCTCCGAAGTTGAGGTCAATCTGGGTCCATCTGTTGTCAAGATAATATGATAAATGTTATCACTACC

>BPA_758

TCTGTTGGAACCATTTCATGGCATCTTCCTTGGTAAGGCGGTGGGGGAAGCCAACCTTACCGGACTTTCGCTTTCTGTGAGCAACATTATAACCTGGACGGCCGAGTACAACGTAAAAATCCAAACCGTAGATACCGATTGAGGGATCGTATTTTATACCCAAATCAATGTGTTCTTGAATACCGAAACCAAAGTTACCGGTGGCAGAGAAATTGTCACGTCTCAACTCGTATTCACGTACTTTGAGACCGCGTTCTAAGATTTCTTCAGCCTTGGCACCACGTACGGTGCAGTGTACGGCAATCTTTTCATTACGACGAATGCCAAAAGAACGTACTGTGTAGCGAGCTTTGGAGAAGACGGGTTGTTGGCCAGTCAATTGTTCCAATACCTTAGCGGCACGCGTCAATCTATCACCAGATTCACCTACACAAATGTTCAAGCACAATTTTCTGATATGCAGATCACGCATAGGGTTCTTGGCGGGATCTCTTTTGATCTTCTTTCCAGCTTCTTTAGTAGCGGCC

>BPA_759

CAAAATCTTGATAGGCCACGAAATTATTCCAAACACTCTTGAGGCGTTTACCAAACGTTATCTCTAAACATTTTTGTAAGTTCAGTAGTTCACTTTCCAATGAATCTATTTCCTGTTTTAGACGTTCTTCTCGAGCCTTACTTTCATTTAACTGTTGTGTGGCAAATCTATTGGATTCTAATATTCTGTTATTTAATGCTTTAATTTGTTCATCTTCTTCCTTGCGTTTTTTTATTATTTCGGAAAATTCATTATCTTTTTCTAGTATTTCTTTACGTAGATCGCTTTGCTTTTGCAAAGCTTCATTGTTTAGCTCGCATATCTCATTTATAAGTTCATTTTTCTGATTTTCTAAAGTTTGTATCTTGCGTTGTTCCATGGATAGTGAAGCTTGCAGTTCCCTTTGTACTGTCATACTTTTGAACAGATCTGATTTTGTTTGTGCCAACACTGTGTTAAGACTTTCGAATTCTTGTTCCTTAAATTGCAGTTGTTGTTCGAGCAGAGCTTTATCAGCCTTAAGACTGATCACAGTTGTAGTTAAACTCTCACACGAAGATTTTGTGAGGTTAATATCTTCTAATGATCTTTTTAATTGACAATTCAGTTCATCATTACGTTTTTGTTCAACACGAAAGAGAGTATTGAAAACGTGAAGTTGGTTCTTCGCTTTTATAGCACCGCGTTTGAGATCAGCCACTTCATCCATTAAATGTTTTACTTCGTCACGTCTTTTACGCCAAGCATTTAACATAATTGTTGCAAATATTCTCATGCCAGCCATTTGAGGATCTCGTCCAGACATAGTTACATAATAATTATCTTGATTATACATTTTGTGCACGAAAGAGTTTTTGGTATATGCTACCATCATATTTTCACTACACCTGATCGATGTCAAAGATTTTTTGTCTTCACAGTTGTTGTTGTTATTATTCAGATGCATTGTAGATGTAGGAACTGAAAAATTCATTTGACGTCTAGCATGATTAAAGTCCATTTGTGAAATTGATTTTCTCTGGCGAAAAGCTAATATAAGTCCAGACTTTTGCCGTACGACGCACGATGTGGTACTAGTTTTACCTTCAAATATTCTTTGCTCTTTTAGCGTCTCAACTTTTATTATTGAAGGCGAAGGCAAGGACTCATTTGTTGTCAATGAAGTAGAGGATAAAGATGGTGTAAGTTCAAGAGCTCTGGGTATATGGGATTTTTGTTTTAAAGTATTTTTAGCTTTCTGCTGAGACAATAATGGTTTTAATTGCAACTTCCTTTGTGATCTTCCATGTGATCTTCGCAAATTGGTTTTGCTTTGAGATTTCACAGACTTAATTTTCCTATCAGCTATCTTTTGACTATTATAATTATTATTGCGACTATCGGTGGATCGATCAGTAAATTCTATAGTTGTTGATTCATTTCCCGTCTCATTGCAATGTTCTGATGGTATTTGAAGAGGCGGAATTTCTATAATCGCTTTAGGCA

>BPA_761

TTTCACTTTGTTTAATTCTTCCTCTTCTGTTGTTGCGGCTGCTGCTGTCTGTTGATTATTTATGTCTCTCTTAGATTTAGCGCTTAATCAGATATTGTCCACAGAGTCGGCATCATCATCAGAACTGATTTCATCACCAAATTCAATATCTTCATCCAAACCATCTTCCACAAAGGTGACTGTATCGTTGATACGCACAGATTCGGGGAATTCACCATAAGTTTTAAGATTTCTAGCTTCGTCTGATGTGTATTTCAGAATAACATCGGCCTTAGAGTCTTGATAGTCACGAAGACCAACCAAAATGATGTCGCCTTGATTGATCCACACCTTCTTTCTTAATTTCCCCCGAATATGACACAGACGTTTTACACCATCAAAGCACATTGCCTCCAAACGGCCATTTCCCAACATTTTTGTTACTTGTGCATATTCTTGCTGATCTTCTTTGAAAATCAATTCACGTTTCTCGAATTCATTTTCGTTCTTACCACGACGACGATTTTTGCCACCTTTTCCCTT

>BPA_766

TTATAGTAGCGGGCCAACCTGTGGATACGGGATTCAACCAAAATAAGACGGAATTTACCATCCTTATCCTTGCGGTTACGTTCCAAATGCTTGCGAATGGCTACAGCCTTCTTGATCATGTGGTATAAATCTTCAGGGATGTCGGGTTTAAGACCAACAGATTTCATGATACGCAAAATTTTGTTACCATTAACAAAACGTACTTGAGCAACGCCATGGGAATCACGAAGAATGATACCGATTTTGGAAGGGGTTAAACCTTTTTTGCCCAATTTCTTAA

>BPA_804

TTGTAGTGTATAATTGCGTACAACTAAAGGTGAAAATTCAACAATGGCTGTTAGTATATCAATAGAGGCCGCTTTTGTTTTCTGATCATTCATTACCAGTGTTATTTCTAGAGCTTGTAAAATACCCAGACACGTTAGTGTCTTGTAAAAGGAATCTTTACCTTGCGGCTGTAGATTTTGCGCATAATTACAAAATTCCTTTAGAAACAACACAATATCACGAC

>BPA_809

GCTTCTTCTTCGGGTGTCAAAGCCAACCCAACCTTACGTGTACATTCTTGCTTTACAATAATCTTTAAAATTTTATGACTCAATTCAGCCAAATTACGTTTGTATTCCATTATTTTCGCTGTAGCCGCCGTGTGTCTTTGTCGTAATTCAGATAAATCATTTTCCACTTTACGTAAATACAAAGCATGCATTTCAGTTTCATTTTCCTGACATTT

>BPA_815

GCACTGTGTTGAGCTCAATTTCACGATAATGCCATAGTCGATATTCGGCCGTTGGTGAAAATTCATCTAAAGGCTTTTCGTCTAGAGTTTTCATTTTTGTTTTCATATACCTTATCCAGCCATTTACCACGTCTTCTAATTGTTCTTGAGAATACGTTGGTATCAAATTGATTTTCTTAGTGTCTATTTGAATTCTGACTTTGGACTCTT

>BPA_825

TACGTTAAACGACATTTTTCCTTTAAACTAAATGAACTTCCACTTGATGCTTTAACTGTCAGTGAAGTTTTGCGACTGCACATTTTGTCTTCTGGGGCTACAGTGAAAAATAAGAATGCTGAAAAATGGCGTATCATGTACAGAAACGGTTATACTTCAACGGAGGATCCAGGTCTAGCCCTTCGAATACAATATCCTCATATATTAAGAGCCCTAAAACTGTACACTGT

>BPA_845

GAATCAAGTTGGGATTCGGCAAATCTATGCCCACCAATTGTGTTTGGATTGACGGTATTGGCGAAAAGATCTCGGAATCGCACTTGCAGTCTCAATTTTCGAGATATGGCGCGGTCAGTAAAGTTGTGGTGGATCGCCAAAGGCAATTATCATTGGTGTTGTACGAGCAGATACAATACGCACAGACAGCTGTCAAAGAAATGAGAGGTGCCACATTGAG

>BPA_852

CTGCCTTTTCGTATTTGGGAGGGTTGACTTGTTGGAGCTGATCTTTCTTGAAATCTTTGGTCTCACCGCCGGGCAAGCCAACGCTGACGATATCACCCTTGGTGGCCTTAATTTCACCAAGGAGATAACCTTCCTTCTCATCGGGAACCCAACAGTTCTTCTTAGAGTCATAAGGTTTCGATTGATCGATACGTCTTTGTTCCAAAGAAAC

>BPA_870

ATTTAAGAATAAAGATTTTTTACCTGGTTAAACACTTTATCGTTATCAAATCGCACATGCATATTTTGCTCAAAAGATGACCATTGCTGTTCTCATTTTCAAGACATAGATGTTGCTAAAAGTTTCTAAAATGTCAAAGGTCTGAATTGTCTATGACTCTGACGCAATTGTCTATCGAAGGTCCTTGCTCGCATCGTGGTTTGCTTCATCGCATGAAAATATTTTCACCTGAACAGG

>BPA_874

ATTCACTTGGCCAAGCGTGTCCACAACATTGGCTTCAAGAAACGTGCTCCCCGCGCTATCAAAGAAATTCGTAAATTCGCTGAGAAGGAAATGGGTACCACTGATGTCAGAATCGACACCCGTTTGAACAAACACATCTGGTCTAAGGGTATCAGATCTACTCCCTTCCGCGTACGTGTGCGTTTGGCTCGTCGTCGTAATGATGATGAAGATTCCCCTAACAAATTGTACACTTTGGTTACTTATGTTCCCGTACCTACATTCAA

>BPA_879

GCAGCTTAGTGCAGACTGTATTTATGTTGGGTGTTTTTACCGGAGCGGTTGTGTTGGGTGGTTTGGCTGATAAGGTGGGACGTAAAACTGTATTTTGTTGGTCAGCTTTATTGCAACTTATTATTGGTGTAGCAGTAGCTTTTATACCAGAGTACTTCTCCTTTATGTTTGCTCGCTATCTATTAGGTATTTTTGGATCGGC

>BPA_922

AACATAGTAGAAGATATGAAAGAAGATGCTGATGAAGATGATGAAGAATATCACGGACAAAGTGGTGATGAAAATGAAACAGATGCCACCGAAACATCTGAACAGAATGCCAGACATAAATCAATACAAAAGCAGCGAACAGCAAAACGTAGTAAAACTACAAAAGCAACAACATCCGGCAAAACAAGCAACGTTATAAGAAAACTAATATTAAAAACACCCAATAATGAAG

>BPA_934

CTGTTATCATTGCATTAAGTGTTTATCTGACCATAAGCTTGGCAAATGTCTTTGCTTCGGCAATTCTGATTGTGGGTACAGTTAAAGAAAGACATTTATTGTTAGCACCCTGGTTGATAAATGCTTATATTTTCATATTCTTCAATGTCTTAAATTTCATTTACATGATTTACGTCTCGATTGCTGCCAGCTTCCCAATAGGTTCGGTGATATTTGGAATCTTAACTGC

>BPA_935

ATTATCAACGCAATATTATTAGCGTGTGTAATTTTCGCAATACGATGTTGTTCAGCATCGCTGGCATTTGTTAAGACAATAACACGGAATTGTTTAATAAAATCTTCAGTCAATTCACCAGTATACGATTTAGTGCGGACGTAATTGTTTAATTCCGCTAATTTATTACATGAAGCTTCAGCACGATTTTTACCAACATCCGATTCGGAGAGATAAAATTGTGAAGAG

>BPA_940

CGCAAAAAAACAGCCACCGCTGTAGCTTATTGCAAACGCGGTCGCGGTTTGCTCCGCGTGAATGGCCGTCCCTTGGAACAAATTGAACCCAAAGTTTTGCAATACAAATTGCAAGAACCTTTGTTGTTGCTTGGCAAGGAAAAATTCGCTGGTGTAGATATCCGTGTCCGCGTTAGCGGTGGTGGTCATGTTGCTCAAATCTATGCCATCCGTCAAGCCATCTCAAAGGCCTTGATTGCATTCTACCAAAAGTATGTCGATGAAGCCTCCAAAAAAGAAATTAAAGACATTCTTATTCAGTACGACAGAACCTTGTTGGTTGGTGATCCACGTCGCTGCGAACCCAAGAAATTCGGTGGTCCAGGTGCCCGTGCTCGCTACCAAAAATCTTACCGTTAAAATTTTATATTCAACA

>BPA_961

TATGGTTTAGGTGCGGCTGATCAACATGCCGGCATGGGTATGGGTGACTTAAGTGGTTGGGGTGCAGCTCCTACAGTGCCAGGTCAGGCTGGTGCCTATCCGGGTACTGGTCTAGGAGCTTATGGTCATCCCCAGGCTGGACCGCCTACACAGCAGCGACAAACCTCTTCTCAGCCTAGTTATCGCCAGCATATGCCCGCCTATGGTCAAC

>BPA_963

TCTTCCCATAGTTTATCTTCTTGACCTTCTCGCTTTTCATTATCATCATGTTCATGATCTTCCTCCTCTTCTTCACCATTAGCATGTTCATGTACTTCATGATCTTCTTCCTCTCCATGTTGTTCATGGGCTTCGTTCTCTTCATCTTCTTTATCCTCCTCGTCTTCCTCATTTTTATCCTTTTTCTCATTATCATCTGTCTTTTTATCTGACTCCGACTT

>BPA_982

AGCGGTCATAAATTCTTCAATAAATTCATCATATTCTGGGCCAGTGACACGTTTGTGTCTCAAACCAATGTAAAGAGGATCATTCAAAATACTTTCGGTATTGGTGCCAACATCTAATGTAATTGGTAAACATTGATGGGGTTTGATGCCAGCCAAAGCAGTATACAGAGATAATTTACCCACAGGTATGCCCATACCATTGGCACCAAGATCACCCAAACCCAAAATACGTTCACCATCAGTAACAAC

>BPA_1008

CTTGGATGCGTGAACCACCGTCACTACTTGGGCGGGTCCAAATCAAAGTTACATTACCTGAGTCCCAGTCGATAACTTTAGGTTGTCCTGGTGGATCGGGAACAGTGAATTGATATTTAGCCGTAACAGGTTCTTCTAACTCCAAAGGATCGGATAGTCCATATTGGTTTTCAGCACGAACACGGAAATGATATTTATGATTTGGTTCCAAA

>BPA_1051

GTAATTAAGGGCAGCAGCAAGTAATCTATGAACCATAATATCTGCATAACGTCGTATGGGACTAGTAAAATGTGTATATATGGGTATAGACAAAGAAAAATGCCAAAAGTCAGCATCTTCTGTTTTGCCTTCGCTACAATAATAACGTGCTCTGGCCATTGGTTTTGTTAACAAAGTACTTAAGCAAGCATCGAAAGCTTTAGGATCACTA

>BPA_1080

CTGGTATATTAGCAAGTGGTGGGGGAGGAGGTGGTGGTGGTGTAGGTAGTGCTAGTGTTATAGGTTTAAGACCAAGTCATCATCATCACCATCTCGATAGTGGTAGCAGCAGTAGTAGTAGCTGTAATAGTGGAGGTAGTGGCAATAGTCCAACTAACACTTCAGATACAAACAATTGTGCCTGTTCCTTAAATGCCATGGTAATATGC

>BPA_1082

GTTTGCTACAACGTATTTGTTTGACCTTAATATTATTTATGATATTTGCAGTGGCCGAACGTACATTTAAACAAAGATTTCTTTATGCCAAACTATTTTCACATTTAACTTCTTCACGTCGTGCTAGAAAATCAAATTTACCCCATTTTAGATTGAATAAAGTAAGAAATATTAAAACCTGGTTAAGTGTTCGATCATATTTAAAGAAACGTG

>BPA_1087

ACCTCTTCGCCAGCTACCAAATAATCATAATAAATGATGATGATTGTATCGGAATTCTTAGTTTCAACACGTTGCACTTGTTTGGCTTGACGTATTTCCTCAAATTTGTCATTGTTTTGCTTGACGGATTTCCTCAAATTTGTCCGTATCGGCGGTAAAACCAGAAGGTAGAGATATTTCCATAATGGCCATATTCGATTCTTTAATATCGCTATTGGCTCCGGGTACAAAATCAGCACAAACATCCATTTGCAATAATCTGGGATGAGCAGAGGGGAGAATATTGTGTTTAATGTTGAAACTAGGTTTATCATCTTTAGTAGCCAAATTGTAACGATATGACAATTGCACCAAAGAGGAGCCTTGTCCATCAGCTTGGAGGGTTACTTGGCGTGAGGATTTGGGAAGCACATGAGTTTGCAAAATCAACGAATTCTCCTTATCCACCGAAAAACTACCTTTGGTGGTTTCTTTGCCCTCGGCATCTTGAGCCTCAAATTTAATATTCATTTTACCGTCGCCAGCTGCAATATATTTTTCAGCAAAACTTATGAGAGCTTCCAAACCTACCACAGTATCTTGTGTGGAATCAAAACCACCCATACTATTACGATTACCGATCAACCATCTAATTATGGGTAGCAATTTTCCAGCTGGTTCGGTGTACACATATGCTTGCAATATATAAGAAGTGATTTCAATATCGTTGTTAGTATCCTTTGAGGATTTTGTCCACCATTTAAGGCCATTCTCTTCTTGGGCTAA

>BPA_1089

TGTTTGGGTTTCGATAAAATCCAGGATTATATAGATCATAAAAATACATATATGGGAGCTATTATAGGCAGAGTAGCGAATCGGGTATCAGAAGGCAAATTTTGTCTGGATGATTTTGAATATAGTTTAACAAAAAATTTCAAGGACAAATATCAATTGAATGGTGGTTTTAATGGTTTTGATAGTATGATTTGGGATATAATTGAAGAGAAAGCTAATGGTATTACCTTGCAACATATAAATCCTGATGGCATGGAAGGTTATCCGGGACAATTGACAACAACTGTAAAGTTTACCTTAGATAATAATAACATGCTTGGAATTTGTTTAGAAGCGTTAGCAAATAAGAAGACTCCTGTTAATTTATCGAATTATCTGTTCTTAAATTTAGCTGGTCATAATGCCCGTAAAGAAGGGCTATTTGAACATAATATTTTGCTGAAATGTAATAAAATTGTTGAATGTGATCAAGACCAAATTCCCACGGGTTGTGTAATGCCTGTACGTCATACTCCATACGATTTAAGAAAATATGTTAATTTCGGTAAACGTCTGAAGAAATTCTTTAATCATACTATTAAGGGGTTTGATCAAAATTATTGTTTGGATAATTGTGATGGTTCAGTAAAGCCAGTGGCGAAAGTGATACATCCTTGTACGGGAAGATTTGTAGAGATTTCTTGTAACCAACCTACGGTACAGTTGTTAAC

>BPA_1102

CTGGTGGGAAGGTGAACTACAAGCTAAGGGACGTCGGAGACAAATTGGTTGGTTCCCTGCTACATTTGTGAAGGTATTGCAGGGTGGACGTAATAGTGGCCGCAATACACCTGTTTCGGGAAGTCGCATTGAAATGACAGAAACAATTTTGGATAAAGTTATTGCTCTGTACCCCTATAAGGCACAAAATGATGATGAATTATCCTTTGACAAAGATGACATTATTAGTGTATTGG

>BPA_1136

TGTTTATCCAAATGACTGCGTTGGGATAAAGCCAAAACGCTATAACGACTTTTCATTTTAGTTCGCAAACTTTCTAAATCTTTACGGCACTCTGATGAACGTTCCTGCCAATAATTTCTTTCTGGCATTGTGTATTCGCCTAACGAAGTAGCAGCACTTACAGTTGTTGCTTGCGCTCCTAAAACCATTACCCCAGAAGCAGGACTTCCCGATAAAC

>BPA_1161

CTTCTATTGATTCTAATTCACCGCGCAATAATCCGGGAATATCTTCAGCTGCTCCAATGCCGCTAGAACAATGCTTGCCTTCAACTTCTTCGGGTATACCATCAGCGGGATCAAATGCTCGTCCTGGTTCTATGGTGCGGGTACCCGAACCATCAAATGTGGTCTCAAATCGTCATAGCTCACATTCGCGTAATTCTTCCTGGGATTTACGTTTAG

>BPA_1237

CCAATAAACCAGCTCCAGCAGTTAATAATTGAGCGCGTGCAGCATCCCATCGGCTGAAACCGGAGCGTAATAAAATTGCAAAATCACCAACTTCATGAGGAATTTCATGCAACAATATGGCAAATGTAGCTAAGACACCATGACGAAATGAGACCAAAAAAGAACCAGCTACAGCTAAACCATGTGTAAAATTATCAATAGAATTTGCCATAAGATTTAGATAACCAGCTACTTTTTTTGGTTGTTCTTTATTTTTTTGTTCACGTTCACGTAAAAAGCAACCATTTGGTACATCTTCAATATCACAACCTTTGC

>BPA_1247

TGGTAGTGGTGGTGCTAAGAAAAAGAAAATAAGTGAAGTTGATCGTCTTATGGGTGATGAGGGTGCTGTAAATATGCTTAATTCATTGGAGAAACTAGAGGCCACATTGGGTTCCGGTGATGCTAAATCGACTAGGCCGATGATGAGAAGCCGTGCAGCGACCATATCTGAAAAGCTGCCACGTAAAGACACAACATCACCTAATCGAAC

>BPA_1250

GCGGTGTTATACGTCATGGAGAATGGGCCTGGTTGGCCAGGAATTCAGTGCTACAAATCGTTTCATTGCGCAATGGCCAAACTATTTCTAGTTATGAATTTTGTGAATCTGGTGGCTATGAAAGTTGTTGTATTAAATGCGTCGAAGAAATATATCCCAACAATCCTGAGCTCATGTTGCTGGCCGTAGTTTTAGAAAGTTTTCGTGGGCCAGGTGGTGGTGGTAGTTTTGTA

>BPA_1272

CGCTAATGTTGAATAGAAATAAATGCGACTCTTACGTTTGGTGCTGTGATCAAAACGCAATAATAAAGCAATAAAAATACCAGGTATAACAATATCACCCAAACCCAACATAGCAAAATTGGAGGCATTCAAACCATTGGTAAGTAAATCTTGTGGGAATACTAGTTTAATGGGTGCTTCGAAGCTTTTAGCAACTGTAACCATAACATTTGTG

>BPA_1280

CGCGAATTTAAATCCACATCAAAATCGTTATTATCGTTGTCATCCAATTGTATATCGTTTTCCTCCTGATGATGCTGATGTTGATGATGATTTCCTTCACTTCCTCCTTGTTGCTGTGGCAATTCTATTTCTTCCCCACTATTAGCTGCCTCCTGCGGATAAAGATGAGGATGATTTTGTTGTTGATGAGCATTCGCTTCTAATGGTTGTACTATGACAGCCGAAGCATCAATAGTATCAGCTATATCACTACGATTTAAATGAGCAGTCAGCTGCTGCTC

>BPA_1286

TGATCCGGGTCCTAATAGTACTTGTTTCTGTTGTGATTTCTGCATTTCCTCCATTTGCGCACGTTCTGTTTCGAAGACGACAGGTGCAAATAAAATAACCGAACTGGTAAAAAAAATCCATGAAGCATTACAAGTGAATTTATATAGTCCCTTAACACTGCTAATGGTGCCATCCACCAATAAGCTGGTAAAATCACGTACAGGTTGCGGGAACATTTCTGTTAAACCCAAAAGACGCTCACCCAGAGTTTCATCGGGTTCATCATCGTAGTTTTCTTCATCTTTGTCACAAGTAGTGGCTACAGTTGTTGGGGTGTTGTTCTTTCTTTCAGGTGTTTCGTCTTTACTACCACCCAATGAAGACATACCGCTATCTTTTTCGATAAACTCGATATCGGGATCGGAGTCTACCAAATCTTCAA

>BPA_1295

TTTCTGTTCCACCACAGACTATTGCGGAATCTGAAGAAGTTTCCAATGTTACACCACGCACGCGACGCAGTGTGAGAGCCACTTCTTTGCAGCCAAAGGATAAGATTGATGATAATGAAACGACAACGCCTCGTAAACGTGAGAAATCGCAAGATGTATTAAGCCCTGAAACAAATTCAAGAACAGCACGTGGATCATCTGTTCCTTCAGGTGCGGTGGCTAGTAAAATTTCAACTCGC

>BPA_1297

ACATTATTCATATTTGTATTGAGGGCATTGGAGGTAGCAAGTGTTGTTGTGGTAGCATTAGAAGATTGTTGTGTTGCCGAAGACATATCCAAACCCAATAAATCGAGAAAATCTTGATTATTAGCGGTTGAATCATTATTAGCATTGAGCGTATTTTTCTTTTGCACTGATAATTCTGTAGACGTTGTAGTGCCTGTTAAATCTGAGCCACCCAATAAATCTAACAGAATATTCGTA

>BPA_1328

CTTAACTTTAAAACATGCTTTGCTCTTAATATGGGCCGTTCTTGTAAAGCATACAATAATATGCGCACATTTTGACTGCCCTCCAGTTCTACCACAAAGCTTTCATTCCACATGGGATTCATGGATCGACATATCATTTTGGTGGCAGCTTTACGGAAATAATGTCCATAAGAATCCACTTCAATGGAAATATACAAATCGGCCGGTT

>BPA_1362

GGTTTTGGAAGCTTGTCCTCAACAAACGGCCCATTGTAGTTACCTTTATGAATTATATCATAATAATTTCCCATGTATTCTTTATTCAGGTTAGCTTGGCTGTTCTGCAATCGTTCGTAGTTTTCTCTACTGCAATCTAAATTACGTTCATTGCTATTTAATGGGGAAGAATTTTCATCACGCTTGTCGTGAGATATTACTAAATGTTCATGTTTCAATGAGTTTC

>BPA_1370

GAAGCTTTTAAAACACTTGGGAATGCTATGAATATTCTAACAGATACTAGAAAGCGTAAAGAATATGATTTATCTGGCGGTGACTACATCTCACCAAATGGAAATTCATCTAAATTCTACAAGAATTCAACATCTTATCAACATTATTATAGTCATAGTCACCAACACCATTATCATCATAGCTCTTCTGAAGCATCATTTAGTGGGGGTGAAGAATTTACGGCTG

>BPA_1393

TTTCCAACTGTTCTGCAACAATGCTCACTAATTTTTTCATGCGCGCCTCATAGTCTGTCCAATCACAAACGATTTGTAAACAATGAGCCAAATTGCAATAGGCATCAGGGAAATCTGGTTTCAATTTCAATGCGGTGCGATACGACTGTATGGCTTCGGGAATGTTGCCAGAATCCTTATGAATGCTAGCCAAATTGCTATGGGCATCAGCAAATGCTGGATTTATTTGTATAGCACGCGAGTAACATTGCAGTGCACCTGCCACATCTTGTAATTCCTTTAGCGTGTTGCCCATATTGGAATAGGCATCAGCAAATGTTGGATTGATTCGTATAGCTTCTTTATAGTGCATTAGAGCCTCCTTCAATTTACCCTGCTGTTGTAGTACAGATGCCAAATTAGAATGAGCTGCCGCAAAATCAGGAAAGACTTCTAAAGCCTTCAAATACAAACGAGTGGCTTCC

>BPA_1406

AGTAGTTTACTCAGCTCCCAGTGGTCACACACTCAGTGCCATTGATGTGCATCGTCAGAAAGAAATTGAATTGCAACAAAATGATGCCATGTGGCGCCAACGTATGGCTCAATTAGAACAGACCCTTAAGAAGACCAATGCCATTATGGAAACAGAATATTCATCGGCGGTTGAAGATGTACGCAAACGTTTTGCCACAGCTTCACCCATTCATCAACTGCCACCATGTCAAGATCTTAA

>BPA_1419

CAGCAGCAACAACAACAGCAACAAGTGCAACAACATCATGAACAACAATTCATGCAACAACATCAAGAAATGCATCAACAACAAAGAATTAGCCGCACTGAACATCATGTTCAAAGAAGTCAAGTAACCACCCAACAAAGAGTGGAACAAGTTGGCGGCACTTATGTACAACCAGCTTTGACACACATCTATGCTCAGGGCGATATCTC

>BPA_1425

CGATGGGGACATTGAGCGCCCTCATCTGAACCATCTTCACAGTCGGGCTTGGCATCACATTGCCAACTAGCTGGTATACACATGTTATCACATCTAAATTCATTTGGCTTACACTGCAATTTACAACCAACTTCATCCGAACTGAAATTACCAGCAACATTATCTCCACAATCGTTTTCACCATCACAACGCCATTTTTCATTTATGCAACGACCGTTTGAACATTG

>BPA_1460

TTTAGTTGAAGTTGTGGTAGAGGTAACGGTAATAGTGCGTTTGGCAGCTGTAGGAACTTCAGCATCACCATCTTCCTTAACTGAAGCTTGATCAGCATTATCGTTATCTGTGGCTTCAATAGTTTGTAAAGGTTCTAGATGTATGGTTCCCTCGGGATTAACAATTTTTGAGGTAGCCAATGATATGCGTTGTAATTCAGAGGAAG

>BPA_1472

AATTGAGACAATTTTGCAAATCTTTAAAATTTATTAAACAAAAGTAAGAAAAAATGGCCCGTACCAAGCAGACTGCTCGTAAATCCACTGGTGGTAAAGCCCCCCGTAAACAATTGGCTACTAAAGCCGCACGTAAATCAGCACCCTCAACTGGTGGTGTTAAGAAACCCCATCGTTATCGTCCCGGTACCGTAGCTCTTCGTGAAATCCGTCGTTACCAGAAATCCACAGAATTGCTTATCCGCAAATTGCCTTTCCAACGGTTAGTGCGTGAAATTGCCCAGGATTTCAAGACCGATTTACGTTTCCAATCTGCTGCTATTGGTGCTTTACAGGAAGCCTCTGA

>BPA_1478

CTGAAACTTTAACCACACCCAACAACAGTCCCACACACAAAAGATGTCTATACATTTAAGTATAAATTCGAGTGCCTTGTCTTTAAATGTCGGACCATCTTCATCATCGTCCTCACCTCGGTCACTAGCACGACTATGCCGACCAGCGGCTTGTTCGATTATATCATTTAGGACCATTACATCTTTCATGTCTACCACTGTTTGAG

>BPA_1481

GGGCAACACAATATTCCAATGGTGGCCATTTGGGATAATTCATTGATTCTAATATGGCTGAATTTAAAGCTCTACAAACAGTCTCTCTTTTTGTTGGACATAATTGCCAGCCTAAAGGACTTGCCCAGGGATTGGAATAGGCAATTAAACTAAATGCATCCTCCAACATTTGTCTCTCTTCATCAGTCATTTTATTTTCTTTTTCTAACTGTTGACC

>BPA_1484

CCGTACTCCGAATTATATGTGCGATACATCATTTTATTATTAGTAGTCTCCAATCCATAACCTGTGAAGTTATTAGGACTTTTGATTGTTTCTCTTGTATCCAATTGTTCATATAACTGAACCGTCTGAACTAATGGTTTCCGCTTTAGAAACTTTTGAAACTCAACTTCAGGATTAAAAGTGTCGAAGTAATTACAAGCTTGTTCGC

>BPA_1485

GTTAGTGTATCTAAATCATCTGAAGAAAATGAAAATAATGGTAAGCATGTGGAGGATGATGATAAAGAAGAATTAGACGAAAGTGTTCTTACCGATCACGATCATGATAATGAAGCCGACGATGAAACCGAAAAGGAGACAAGTAAAATTGCCAATATTTCATCGGACATTAGTGAAAGCTCAAAAACTTCGAAACCTAACACAACTAAAGAGACACAAAAGAATGACAAGAACACAAACTCCTCCACCTTATTATCGTCGTCAGCGTCATCTAAAA

>BPA_1506

TCGGTGGTGATCCCTTCAACGGTACCGATTTCATTGATTGTCTTGAAATATTCCTCAAGGATCCCGACACGAAGGGAATTATTTTGATTGGTGAAATTGGCGGTGTTGCCGAAGAACAAGCTGCCGAATATTTGACACAACACAATACCGGCATGAAAGCCAAACCCGTGGTATCCTTCATTGCCGGCATCTCTGCTCCACCCGGC

>BPA_1518

CAAATAATCGATTTAAATGGTAAATTGGAATTATTGCGTAAACAAGAAGAAGAGAATGTTATATTATTGGCACAAACAAAAGCCGCCATACATTCAGAATTGGAGAATAAAGAAAGTGAAGTTAAGAAATTAAACGAAAGTGTTAAACAATTGCAAGGCGAATTGCAACAGGCTACAAAAAAAGAAAAACAAAGTGGAGTGG

>BPA_1535

GCCAAATGTTCTGAAGTTTGTGGCTGGGGGCATGATTCATTTCCAGCAATCAAAAGTAATGCGGCATCCATAACAGCGGCACCGTTCAACATAGTTGCCATAAGAATGTCGTGACCGGGACAATCTACGAAACTTACATGACGTATTAATCTAAAGCGTCCGGAACATGAGGGTCGAGTACATGGTAAACTGTCGTCTTTGCTAGAGCTATCAGATACGAAACATGCAGGTCGTG

>BPA_1537

AAATGATACTATGAGTGTACAAAGTATTACATCTTTAGGTCGTGTTGGACGTATTTTAGCGGGCAGTATTGATCCCTCAGCAATGAGTATTGATCGTGAATATTTTGCAAATACCAGCAATAATAGTCAGCAGCAACAGCAGCTATTACAACAGCAACAACTGCAACAACTACAACTGCAACAGCAGCAACAGCAACAACAACAATATTTACAACAAGCACATTTATCCTCATCATCAGCAACAGTCGGAA

>BPA_1543

CGACGGGTGCCACGGAATGTGGTGGTGTGACAACATGCTCGGGATACACGAGCACCACCACGACCACGAAAACTGCCGCGGGGGAAGCGATTAGTAGTGGAGAGGCCAGGACGATTTGTACGTTTAGACATAACCTTAATTTGACGACCACGAAATAGTGTCTCATTCATGGCCAATGCAGTCTCAACATATTCTTTGGAACCAAATTCAATATAGGCAAAACCCTTAGGATGGCCATCAGCCTTATTGCACAATATAGTCACTCGATTAATTGTGCCACAGCCATGGAAATGAGCCTCCAATTCTTCAGCCGATGCACCATAATCCACATTACCCACATACACTGAACGAGTATCAATTTCTTGTTTCTCCTCCAATGACAAGGGCACTGTAGCTAAGCCCGTTGTAGAACCGGCCATTTGTTTATCTACCTCTGATTGCATTTGTTTAATTTTTTCTGCTTCTTCTTCCATTTCTTTGACGCGAGCTTTTATAGCTTCCAATTCGGGATCAATCTGCATATTGTTATCCTCCTC

>BPA_1544

GTAAGATTGGATTTTCCATATAAGATTGTAAATGATGCAAGTAAAATGTTGGTGGAGGCAAAATTTATAGGAAGCAATATTACTATAACATCGAGCCGCATAAATGTTTTAGATTTTAAACCTCAACCCATATTGGAATTTGTGGAAAATCCGCTTAAATTAAAAGAAGATTTAAGTAAAAACCCTTTAAGATTGAAAGTAACTGAAGAATCGGCCGTTTTAGGATATGGTAGTTTTAAGTGGCCGGAAGAGTTCTTATTCAATTTATTAGAAAGTTCTGGAGAAATAACTCAAACATTTGAAACGGAACTTAAAGAATGTGATGGTACAAAAACTGGCACATTACACGCCATAATACGTCTACAACCGAAATGTGAAGAATATAAAGAAGATGATTCTCCAGATGGCAAATGTCGTAATGCTAATAAAGAAATTAATCCCAATGATGTCCTTTTTATGGTGGGAGAAGATAAAAAATCTTGTTGTTGCTCAGAAGTTGGTTTAATACCGGCTTCAACTTCAGATGAGGATCTCCAACCAAAATGTTTAGACTTATCATATTACCAAGCTGTTAATAGTCGGCTGGTGGATAATACGGACATTTTAAATTCACTCAATCCGTTAAATAAAAACTTTAAAACTGTAACTAAACATTATCAAAGACTAATTGATGCCATAAAATCTAAAAAAACATCTAATACATGCCGCAAAGAAAATGAAAAATTATTGTATACATTAAGTTCTGGTAGTCGTTTGAACATTAATATACATCCAACTAGTTCAGATTCATTTTATTGTAATTTAAATGATACGGCTTTTAGAGCAAATAACAACAAACAAATAGTTCATATAGAGAAACCTCCCGACTTGATTGTAAACGAACCCATATCAATAAAATACTGCCCACTTTGCAAAGAAGATATGAGCTTCTTACCAAAATTGGCTGCCTGTCCCAATTGTTGTTATAAACCTATACCCTGTTTCGAAGAGAAAACCTATAATGAGGAACAAAATGCAGAACAAATATTAAAATCATTTCAGGATAAATTGCAAACTAATGAAAATCCGACTAGTAGTACTAATCTAGCAGAAAAATCTACATGTCGTTGTACCTGCAAATATGGTTCTAATAAACCCTGTGCTCATTGTCGTATACGAAAACTTTGCGAAGATATTTTTCAATCATCCCTACCAGTATCAAAAGCCGAAAATGATTGTGAAAAGAAATCTTCAGATATGCATTGTCCCAGCAGTGCGGATGATCGCCCGAGTGAAAAGAAATCTTCAGATATGCATTGTCCTAGCAGTGCGGATGATCGCCCGTTTTTGACTAAAGTATTTTCAGAATTAAAAGATCTCTATGATATTGAAAAGCCTAAAATGAAAAGATTTGAGCCAGATAAATATTGTGAAAAGATGCTTAACAAAGAACCATGTAAGAAGAAGAAAGCACCTATTAAAAAAGAAATTACAGCAGAAACGCAAAAATGTGTGGAGCCTGAAGAAGATGTTAAAACATATAAATGTAGAAAACGTAGAAAGCGTTCAATTAAACCGGCTACAAATTTAGCTGCTAAAAGTAAATTCTATGATTATAAATTAATTCAACACCGTTTGTCCACTCATATTGGACACAAAACTTGCATTAATGGCTTTGCCGGACGTAAAAATGTTCCTTCCCACATGGGTTGGCTATGGAATGTAAATAATTTTGGCAAATGGAAACCAGGTTACATACGTAAACCTATTAAAGAATTAATGAAATATTTTCTTAAAGATTTTCCTGCCGATACTTTACTGGTTTCTCAATATTCCTATCGTAATAAGTCACGAAATTCTAATAACTTCCCTCCTCTTCTGGTACCACAACAAAAACCTACTTTACATATCCACAAAAGAG

>BPA_1547

TTTCATAGACCCTCTTTGTCATTATTTTTTTACCATCAACAAATATTGTCGAAGTTGTAGTACTTTTAACAGCTCCATTTCTTCCGGCACCGTTAGTGCGGCCACTACCATTCGACATGCTGCTATAGGATGAGAATGAGGTAAAACCATTATTGGGCATAAAAAAGTCATTCATTGAATAATTGAGCATTGGAGAAGTAAAAGGTGAAGACAATTTTAAATTTGTTCCATGATGATGGTGATGACTACGACTACTAGATGTACCATTCGAACGCCGGCTACCTTGCTGTGAACTGTAATGAACTTCTCTAAATAAATCGGCAAATGGTGAGTTTCCGCCGAAAAATTCACGAAAAACTTCTTCTGGCGGTCGGAACACAAATGGAAAATCACCCATTATATTGAAATCATCAAAGTCGTGTGCATGATGTCTTGATGAACGATGGTGTCCACGATCGCCCAATAAACCTTCTTTGCCATATTGATCATATACACGACGTTTTTTCTCGTCTGATAGAACTTCATAAGCTTCGGATAATTCACGGAAACGTTTATTGGCCTCGTCTAAATTATCTGGATTTTTATCCGGATGCCATTTTAAAGCCAATTTTCTGTAAGCCTTTTTAATTTCTGCTTCTGTGGCTTGACGTGGTACTTCTAGAACTTTGTAATAGTCCACCATTTTGTTAGAATTTTCTGTTTAAGCAATTTGTATTGGAAATTATATGATGTTTAAAATTTAAGTACCAATGGCGGATCTTCACACCAGAGTCCTTTTA

>BPA_1549

AAATTTAGAAGTTTTATCATTATTCAGTAATATGATAACGAAATTGGAAAATTTCGATACCCTAGAGAAATTGATTATATTTAGTGTTGGTAATAATTTAATAAATTCCACAGATGGTATTGAACGTTTACGTTTTTTAAAAAATTTGAAAGTTCTTAATTTGGAAGGAAATCCTATATCAAAAGATTCTAAATTTTGTTTAGCTGATTATATTGCTGCCATCTTACCACAACTTAAATACTATGAATATGTAGCTATTAAAGATGAGGATCGTCAGAGGGCTAAAAAACGTTATTATAGGGAACTGCGTGAAATCGAATCTAATGAAGAAATTGAACTTCAAAGTCGAGCTCAAAGAGCTAAAGAAGAACAAGATGCTGAACGTTTATCATCAAGTTTTGTTGAACATTTAAATGAACATCAATTATTTGAATCATTGTGGAAGGGAGATGATGATGGACATATACTTATGATGGTTGGCTCCGCAGCTGACGACTTGGCCGAGGAGTACGATAAAGATATATTTGAATTAACTCAAGAAATATATAAACTTGGATTGCAAAAATTCGAGGAACGTCAATTGGAAATTGAAGAATTTACCACATGCATGGAGGAAGGTCATTTAGAAGTGCAACAAATGGGTCATAAAATATTAGAAGAATTTCAACAATATAAAGACAATATATTCGAGGAAGCAATTGTCTGTCATAAATTTTTAGAAGCCCGAATCATAAGAGGTGAAGAAATAGAAACCGAAGAAAGTATAGAATATTCAGATAAATTGGATCGTTTGACATTGCAATTTGATGACATGGTTAATAAAGTATGGCAAGAATTAATGAGACAAGAGTTGCATTTACACGAGACTACTGAAGAAACTATTATTATTTTTCAACGACGTCTCCAGGAAATGGTAGCAAAATTTGTAGAACAAGCTCAAACCTTTTTCGGTCAATTACGTGATATTGCTGTTAATTTTTCAGAAAATTTAGGTGAAATTGTCAATCGTTATATAGCAACAAAATTGGCTCTACAAGATTTTAAAGGTGTGCCTGAACCTCTGCGTATTTGTATGGAAGATCGTGAAGCTATAGCAAATTTAATAGC

>BPA_1550

CCAACGATTTGAAGGAAGAACCTAAAGTCAAAGAGAAAACGCCCAAGAAAACCAAGGAAAAGGATGGAGAAGAGGATCAGATCAAGTGTTGTGGTTTGTGTAAAAAACGAGCAGAAAACCCAAACGAAAAACCCAAAGAAAAGAAAGAAGAAGAACATGTCAAATTCTGTGGTTTGTATAGAAAACGATCGGAGAAGGCTCCACAAAAACCCAAGGAAAAGAAAGCAGCTGAAGATGAAACGCAATTCTGTGGTTTTTGTAAAAAACGCATGGAAAAACCTCCCGCAGAGCTCAAGGAAAAACCCAAGGAAGCTCCTAAGAAACAAGAAGATGATGATCTAACTGTATGCTGGGGTTTGTGTAAAATACCTGAAGAAGAAAAACCAAAATCTCAGCCTAAAGAAAATCCTCCTAAGGAAAAGTCCAAGCCAGAGATTAAGGAAAAGGTCAAAGCTCCTGAACCACCAGTTACCAAGCCCAAAGTAAGAGAAAAACCACCACCTCCTCCTCCTATTCCGCCCAAACCAAAGGAACCCTCCAAACCGAAACCATTTCTCAGTTGTTTTAAAACCGAAAAAAAGAAAGTTGAGGAAAGAGAACCTTCACCCGAAACGCATATTGTAGATAGAAATGAGGAGGATTATTATCATCCTCGAGTCTTAAGTTATGATGTCAAACCAGACGATTTAGAACAATTTGAGGAAAGAAAACCATCTAGAACATCTTCGAATAATGATTATAAGCCAAGAGTACGTGCTTCGAGACCAAGTCAAGCTTCAACCTCAGCTGCTGTGGAATCTGTTAAATCTTCTCATAGAAAAAATTCAATAAAAAGAATCTCTGGTGGTGGCGCAGAAATGTTATTGTTCTAATGGCAGAAGTCGTTCATTACCCGATATTTGGAATAAAACAAATTATATGAAATCGCCAGATTCTTCTATGTTTTCAGCAGATTGGAATACGGCTAAAGATCGAAATGTTTATGGTTGTCATTGCTCTCAATCAGCTGAAGAGTTAGAACCCAAAAGATCCTATTTTGAAATGGAAAATGGTTGTGAATGTTCGAGATTGTCAAGGGATAAGCAGC

>BPA_1566

CCTTGTTGTTGACGTCCAAATTTATGGGAACGTGTAGACATACTCAAGTCTTTTGTCACCGATGTTTCGTAGTTGCACTTTGGACATGGTATTGGCTTGTTCTTTTCATATTTGAAACCTTTACGCTTTACATGATCCTCACAATAGCATGTCTTGCAACGCAAACAAGAATATTGCCCAAGACGATTACATGATTGACA

>BPA_1572

GAACATTGTGCTCGTACAGAAATAGAAGAAATGGAATTTCCTCGTGGAAATCCACTTTTAGATGCCGTCAATACCATGAGATCTAGAGGTTATAAAATCCATACCGGCCGTTGGCTTGTCGATGGTAATCCTCAATTGATTTTGTTTGACATTGGATCTGGTGCATGGAAGTTGGATCAATTCAAATCGGAGCTATGGGATAAGTGCCATATTGGTATTCCACA

>BPA_1608

CAATTTCTTTTCCAAACCATTTATATTTTCCAATTGCTGTTTATTCGATTTTTGCGTTTCCTCTTTCAAGTGTTTGTGGTCACTTTGCAAAAGACTATGAGATTGTTTTAATTTATCATAACGCATTTGTTGTTCAATTTGTTCTTTTTGATATTTTTCTTCTAATTCTTTGATCTTTTGTTCCAAATTACGCTTTGTTTCCAATTGCTCATTTCTTTCACGTTCTTGATTTCTTTCCAAATGATATTTTTGTTCTACCAAGGTGGTTAAACGTTCATTTAAAGCTTCTTGCTGTTGT

>BPA_1613

ACCAAATCAAAATATCATTGAAAGTGAGAATGAAATTACTCATCAACGGAATTTGACAACAGGGACACCGACCATTATTGTAACAAATAAGACAAATAACAAAAATACAACTGAAATTACGTTACAACACGATGAGTTCTTTAACCGACCTCAGCGGTCACCATCAATCTCGTCATCATTGTCTTTATCTTCAAGTAATACGAATAGATATAAACATAACCCGCCACCCAATATGCATCACTGTAATGGATCGCCTGTTGTTTCAATACGCAGCA

>BPA_1626

CAGATCGAGTTTCGAATTAATGAGGGATATGGTCGAAAGGATGCAATCGCCAGGAGAATCCATAGATTACTTTTTCTTCGTAATGACGCAATTCCAATCACGACTAACTATTTCAATACCAAAGCGTGTCGTCAGTAGAAGAACTTCCGATGAATTGATTATTAGACCACAACAAACAATGGTAAATCGATATTCACGGC

>BPA_1638

ATCTCTGTCACAGGCATTGCAATGTAAAATTATACCAAACCAAGAATATCTGCTTCAAGGATCAATTATTGAGTCTGCAGTCGACCATTTATTGCATAGGCTTCGTGGACTTTGCGACAATGTTGAAACAGCTCCTGAAACGTTCCACGATTTAGAAGTATGTGTGAGCATGAGGCAACCGAATCAACAAATTCCATTAAGTGTGCGTGTTCGTCGTGCACTTGATAGAGACGCTCCGTTTCAATTGCGATATATTGGACAGCCAGAATTAGATCGTTCTAGACCAACACTAGTAAGATCA

>BPA_1667

AATTATCTAGTGTTACACCATCCAGTTGCAATGAGAAACAAGTGCGGTGACAAGTATCTTCACGGTCCATTAATAACTGATGAATTTCTTGTACTAGTTCCATACTCGATAATTGCACAGACAATAATTCTGCTCCAGGACTTTGTATATGTACCGTTATTCCACTGTCACGTAAAGCATCTAAATCAATGTTCTCTTCGTAACTTTC

>BPA_1712

ACAAATGGCAGCACAACATGGAGGTGTACCTCCAAATATGACAGCTGCTATGCAACAAGGTAGTCGTGGTCCTGGTGGTGTTCCCATTGGTATGGGTGGTGGAGGTGTTGGTGGTGACGGTACTGGTGGAGGTTTAATTAGTGGACCGCAACAACAAATGAACAACGCTCAAATGAATCCTAACCAACAGCAAAACCCACAAGGCGGC

>BPA_1738

AGTTGGTTAAAGTTATTCGTCCTTTGGAAAGTAATTCTGCCAACATTAATTCAAAAGATGTAGCCCCATTCGCTCACGAATTATATTTAGTCACTTTGCAGAAACTTAAAGCTATTGATGCTGATCAAGAAGTCAAAGAACGAGCAATTGCTTGCATGGGACAAATTATTGCTTATATGGGTGATTTGCTTCAAACAGAATTGAACACATGCC

>BPA_1842

AAGGCGTAAAGGAGGTCGTTGTACCAAAGGCACAGCGGGCTTTGCCTATGTTGGTGGCGCTTGTGTGGTGAATAAACGTTTGGAAAAAGTTAACAGTGTTGCTATCATTGAAGATACCGGCGGTTTCAGTGGCATCATTGTTGCTGCTCATGAAGTGGGCCATTTACTTGGTGCTGTTCATGATGGTTCACCACCACCCAGC

>BPA_1847

AAACTTATGGCAATAATATTGCTGCTTCAGCAGCATTTATGGCTTTTAACTGGGAACACACTGGTTCCAGTGTGGCAGTATTAGCTTTAGATGATTGTGGTCGTAAAAGTAAAACTATGCCTCTGTTGCATGCTCATACAGATACCGTGACCGATATGAGTTTTTCACCATTTCATGATGGTCTCTTGGCCACAGCCTCAC

>BPA_1855

GCCAATTGTATAGTTTTCTCTTCATTTAAATTGTTCTTATATTTCTTTTCCAAATAACTGTTAGCTTCGACTTGTTTAACACCCACAGAGACAGCGCGAAAACCACAATAGTAACCAGCTGGATCGGTTTTATAAACACAAGGACCATTCTCATCGTCATAGGCGATTAAAACCATACTGCAACCCAAAGGACGCATTTCAGCATTTTGTGTGTATACTTGGTTGATATCGG

>BPA_1893

CCTTGATATCGGTACGACCGGGATCGGAGACACCTTTAACAACGACTACCAAAATGAAAGTAATCAAAGCTTCTATAAGTACAGCTTGACCTTCAGTTATGGTGGGATTATAGTTGGATACACCCAATTCAGCACCACCAATAGTTTCAGAGACACCAACCTTGATAACAGCAGCACCAGCAATAGCTCCTACACATTGTACCGCAATGTAGAGGGCGCTTTTAATTATGCTCATTTCACCAACAATTAGGAAGCCAAGAGTAACAGCTGGATTAATATGACAA

>BPA_1901

CCTGGTGGTCCAAATATTATATATGGCATATTTTGAACTTCGCCACGTAAAATCTCAAATACTGCCTTCTTTTGTATGGGATTAAGCAAATTATTGTACCATTTGTACATTTTATTCTGATAGACGATGTTATTATCTTCATTCAATTGGATATCAAGCTGGGGTTGCTCACGCAAATAAATATTTGAAGGAAAAAGAAACTTTT

>BPA_1904

GCATAGGCGTTCATCTCGTATACATAAGAGTCAACAATTAATGAGCGAAGCAGACATCCAACAAATACAAGAGGATTTACAGGAAGACAAAAATGAATATTTTAACGGTATATTAAAGCGTATATACGAAAAAGAATCGATACATAATGACAGCAGAGATATAAGTCAACAGCCAAGTTTTACAAAATTTCAATTGCCTTACATTATTAAATGCCGTAATCGTTATAATGCTACCAAAAAATTATATGAACAACGTTTTCAATATGAAATTGAACAATTAATTGCCCAGC

>BPA_1931

GGACCATCTCGTATAGCAGAAATAAATATATTATTGGATAAAGAAATAACGCTGTTAAGTGGCATAGATCGTCAGAGACAATTGGTATATGAGGCAATGAAAGATTTTCGCCAAGAACAACTGCTAAAGAAAATGGGTGAACCTATTAAATGGGTGGGTTATGGAGATACTGTTATACATTTAGATCTTTTGCGCACACAAAGG

>BPA_1973

GTCCACTTGATGTTATCGAATCTGTTGTTTTTGAATGTGCTAATTTTGTGCTGGCTATAACATCAGGTTCTTGTAACACAACTTGATTTTCGAATGGCACTTGATTTGAATTACTCAAAACACCAATACCAACTGCGGCTTGTTGAACTGATTTACTGCCACTTGTTTCTGTAAGACTAGATGGTACACTTGACGATTTT

>BPA_1990

GTGGGATGTTCTTCATATTCAGCTTGGGCGTCTTCAATAAGTATTGATTTTTCTTCTGCAGCATATGAACGATTAGAAGAAGCAATGCTTTCGTTTGCAATAACACTAGTTTCTCGTCTAGACAAAACACTGCTCTGATCTTCAAAGGGATTTTCGGGAACAGTAGAAACCGACAAACGACCGCTTGTTGGGGCATTACGAT

>BPA_2026

ACAACTACAGTTTGTGGTTCGTCTGGCGAAGAAGCTGAATTATTTTCGTTTGGTGGCGTTGTGGGCGATGTTAATGCTGAGGATAATTCTTCTGAATTTTGTGTCGATAATAATGTTTGTGAATTATCGTTGTTGTGAGAGTGTTGGTGTTCTTGTTGGTGTTGTTGTTGCTGCTGTTGCTCTTGTGTGTTGTTGTGATGATGGAGATGCGTTTGATGTTGTGTTGACTGTTGACCGGAATCCTCTGTAGCAG

>BPA_2068

CCCGTACCGATCCCGCCGATAACCCGTAGGTGGCGCCGGCGGTGGCGCCATGTTGAACCACTGCTTCGGCGAGTCAACTTGGCCATTCAGCGCCCCAACGGCCAACCGTTGGCACATAGCCGGTTTCGCAATCATTGATAGAGGTTTCTAGATTAATTTTGCCCGTTGATGCGTTAATTGATTGTATAGAAGTTGTTGTTGTCGTCGTTGCAGCAGCTGTATTAG

>BPA_2093

CTCCTTGCTGATCTTCATCATTCATATATGTGGCCAAGGCAGCCAAAGAGCCCAAACGAGCTTCATTGGTCATGTAGGCAGTGGTAAAGGAACCAACACGGAAATTAGGTTTCATTAAATGATTCTCCTCTTCACTTTCATTTGTAACACCACTTTGCACTGTGATCGTTTGATGTATCTGATCCATAGAATCAGATTTTT

>BPA_2101

ATGTAACATTTACAATTTAGCGTCAGCCTGTTTCAATAGAGGAGCCAATTTTTGGGTCAACATAATGTTACCGGCAATCTTAAGTTTACCCTTCATGAAAGCAGCTTGAGGATTCAATTTACCCAAAGCAATATCTACCATATCCTCATCACTAACCGTTAAAGTGGTATCAACTTTTACACCTTGAGCAGGTCCTTCATAGATTTTAGCACTTTTTAAGTCCAAAGTCCATTCCTTAGCAACTTTGCCATCCTTGG

>BPA_2107

TTGGATCGTGTTATAAACAAATTTACGGAAATTAAAGAAATGGCAGAAGCAGAAATTAATGATGTGGCCACATTGCTGGAAGAATTAATGCAGGTTTTGGGTAAGGCAGAACAGGAAATGACTGGAACCATTAATACAGGCACCCAGGTAGATACCGATGACCCAATGGCTGATGAGGCAGAAATGCAAGTGGAAATGGAACCTGCTCCTCCCCGTTTGACGGATAGTCAAATAGC

>BPA_2111

TCAGGTTCCATAGGAATAATGGTATTCAAAGCGAATATGAAGGGATGGACATATTTCATGTACAGATAGTAAGTAGCAACAGCATCTGAAACAGAATAATTAGATAACACTTGTGGCTGTTCCACGGCCATTTTACACATATCCTCTGGATCCAATTCAACGGGATCATAACGTAATTTAGCTTTTGCAACAGCCTTAAGAC

>BPA_2112

AGCATGTTATTAGTAGCAATGCTGTATATGGTAGCAATGGCGGTAGTCATCATATTGTTAATAATTGTGGAAGTGTTAGTGGTTCGACGGGCAGTGGTTATCATCACTCTTCGTCTTCTTCCTCTTCGGGTGGTCAACATCACATTTCATCGCATCATTCTCAATCCCAACATGGACATCAGGCACCACCGCAACATCATGGCGCTATTGTTAAACAACAGACAATTACAATACACGATACACC

>BPA_2146

TGGCATCCACATTCGAGCTCTCCTGCTGTAGATACAAGTGATGGTGATGATGGTGGTGATGATGATGATGTAACAGATGATAGTGTAGTAAATGATGTTGGAATTGTTGCCATTGTTCATAGACATGGTGGTGATGATTATGTTGATTATCTTTTTGCTGTTGTTGCATTTGTTTTTTCTTTTTACGCCTTCTTCGACCCTTTTTCCGCTGTTTACCAGCTTCTGTCACATCTTTGTTCTCCA

>BPA_2149

CAAGAATTTGCTGTAATACCAACTACAGAAGTTGTAAAGGATCATCAACATCAAACATTGGATAATGTAAATGCAAGTACTTCAGTATCTTCTTCGGTTATGTCATCTCCACAACAACAACAGCAACAACATCAAAACAAATCTCCACAAAAGAATAATCATTCCCATCACAATAGTACACAACCAGGTCGTAAAACGCCTGGAAAACATGC

>BPA_2151

TTTATCTAGACTTTGTCTTAAAATTGTTTCCGGACTAATGTATTCTGCTTTCACCACAACTTTATGAATTAAATCATTTATAAATTCTATAGTCTGTTGTTGTAATTCTTTAGCCTCTTCTTCTCTAATTATGGCTTCCCAGTCAACTTCTTCAACAGGTTTTTCCGCCTTTGTTAATTCTAATTCCATAAAAGTTTCAAATAGATCTATAGTTTGTTG

>BPA_2219

CATTTTGGGCGACAGCAGTGTTGGAAAAACATCATTGATGAATCAATATGTAAGCAAACGTTTCTCAAATCAATATAAGGCCACCATAGGTGCTGATTTCTGTACAAAAGAAGTTGTGGTGGATGATCGTGTTGTCACATTGCAAATTTGGGATACTGCGGGTCAAGAGCGCTTCCAGTCATTGGGCGTTGCTTTCTATCGTG

>BPA_2228

TCTTCATATTTCATTTTCCAATCGAGTACTTTATTAACATAGGCATTATTATTTTTGAAATTTTCCCAAGATTGAAAGAATTTTGAATCCTTATGCAACTCTACACCCATTGCCTCCATGTTGGGTTCAACCACTCTTTCATCTGCACTGAATTCAACCTCTTTACGTTTACGCAGACGGGCGGGCGATCTATAAACACGAGAGTTTATGCTTTGAGCATCCATTTCTTGTTTAATCAATTTG

>BPA_2229

CCTGGGCTCTTTTTTGTTTTTCCTTCTTTTTTAATTTTTATAGGCTTATGCTACAGTTGATCTGTGCCTGTGCATAAGAATTCAAGACAGTGACTGGAAGTGAATGTGTGATCATGAAGGTGACGCATAAGAAGATATAAGCAACTTTAACTTTGGCAGTTTTTTTTTTCTTTTCCTATTTTTGTGTTTTTTTCTTGTTCTTCTTGCTTTTTTATTAAT

>BPA_2240

GAGAATTCCACTGAGGGAGATGATAAACCAGAATCTGAATTACGTAGATTCTCACCAGCACGTAGCAGTATTGCAGAAAGAAGACGTTTATACGAATTACATTCGAAGAGTACTACCGAGGAAAGATCACAATCGCCAGTACCATTACGCCGTGAATTATCTAAAGTCGAATCGACTAAATCAAATGCCATTGATATGAAGAGAACTTCGGTGCCCGAG

>BPA_2286

CCCGCCTTGTGAACTGGCCGTACTTAGGGGTCGTGGTGGTTGTGTTAGATTATCATTTATGGGTGGCTTAACGGCAGCCGGTATTGCTGCAGCTGGATTAAACATGGTGGGAGGAACTGAGGGAACACTATTGGCAGCTGGTATAGGTTGAGTAGCAAATGGTGAAGTCATCGGATTATTAACTGGATTTGAATTCCAGTTATTAGTTGGAG

>BPA_2294

GAATAGGCGGTATGCCACCTGGCAGCATTGTGGGCGGTGGACCTCCCATCATCGGTGCTCTTGGTACTCCTCTCCCCATAGGTGCCATATGTTGTTGAGCTGGCCCACCAACACCACGCACAGGCCCTTGTAAGCCTGCCGGAACTGATATATTCACCGGAACACCTCTACCGGAAGTACGTCCCATTCCGGGACCAGGTGCAGCCCCAGAAAGCGGAACTCTTGGCACACCCTCTTCCGATGGCGGTGGCCCTTCTACGGTTAGGGAGACAATATTTTCACCTCTTAGCAAGACGAAACCTAAAACACGTTTCTCCTCCCTTTCCGGAACCTTTGTGCTCTTCGAACGAATTTTTCTGAATTCCTCACAATCTCCAA

>BPA_2350

CTTGATTATTGGGTCCTCCAGGTGCTTGTTGTGGTGGTGGATAACCAGGATATGCTTGGGGTGGTGGAGGCCCAGGCGGTGGTCCTTGTGACAAATACATAGGTATTTGATTGGTCGGATATTGGAGATTATGGTGATAAATTCCTTGACAAAAACCATAAGGATTATACGGAGACAAACCAACAGGAGCCAATGTTGGTACTTGTTGTTGAAAAGCAGGTGG

>BPA_2353

GAAATACTTCACCACAACCATCACAACGTGATGCAAATTGAGCATCATAACAGTTGCCACAATAGATTTTATCGGCCTTAGCACCGAATTGTTTGTCCACCAATGAGAGATGACACTTGAAACATAAGAAGCAGGCTTCATGCCAATGTTTGTCTTTATATGAAAGATCCTTGGAATCGATACCAATGATCTTGTTGCACTCTTCGCAAGTGTTA

>BPA_2356

GCCCTGTCAAGATTTAGTGGATTTGGGTTTAAATTCGACAATTTTAATACATGAAGCCACTATGGAAGATGATTTAATTGAGGAGGCTAAAATTAAAACACACAGTACCATAACACAAGCCATACAACAGGGCCAACTAATGAAGGCACAACACACAATTTTAACACATTTTTCACAACGTTATGCCAAATTGCCACGTCTACAATTGA

>BPA_2369

ATTTTATACAACACACTGGTGGCGGTGTAGGCGCCAGGGGTAATTGTGAAAATCTTGTTATTTTCTGTAAAGCTTGTTGTATGGCCTGATCAATACCAGATGCAGAGGCGGATGATGAAATGGAAGTTGCTGTAGCAGCAGCAGCAGCAGCAGTAGGGCTGGTAGTAGTGGTGTTGGCAGCATTACTGTTATCTCTAGTTAAAGTAGAACATTTTGCAAATGA

>BPA_2380

AACCAATTATGATTTGGGCAACAAAATCATTGAATGCTTTATCAAAGAGAAAATCCAAGCAGGTGATGTGGTTACCATTGATAAAGCTTCGGGAAAAGTTAATAAATTGGGCAGAAGTTTTACCCGTGCACGGGATTACGATGCTACCGGCGCTCAAACCCGTTTTGTCCAGTGTCCTGAGGGAGAATTGCAAAAACGCAA

>BPA_2382

ACAACAGCTGCCGACAATAGGACCGATAAGACATTGCTAACCAATAAACCCTTGATTTGATCCCATACAAAAAAGCCGGGCGTTTGTTTATTAAATCCATGCTTCTGTTCCAATACAAATGTACTATAGATTTTGAAGGGCAGGTCTTTTACATAACCGAATAAACTTAAAATGACTACGAAAAAGCAGCAAACAACAATTTC

>BPA_2396

TTCCTTGGCTTCATTCTTTTTCCGCTTCTCTTCATTTTTCGCCTCTACTTCGGCCAAGCGTTTGCGTTCTTTTTCCTCACGTTCTTTACGTTTTTGTTCTTCTTTTTCTTCACGTTCTTTGCGTTTCTGTTCTTCTTTCTCTTCACGTTCACGTTTACGGGCCAGTTCTTTTTCTTCTTTCTCCTGTTGTTTTTTCAATTTCTCTTCTTGTAATTTTCGTTCTTTCTCTTC

>BPA_2412

ATTGAGAAGATGATAACGGAGCGGGAGACATGAGATTACCAGTTACAGGTGATGGAGAACCTGGCAAATGCTTGTTTGCAAATGGACTATCACTAGAAGCATGAGGATGCTGTGGTGTATGCGGCGAAGGACCTGACGGCGAGGGAGAAGTCAGAGCGTTGCGAGGATCACTAGGAAGCTGATTGGCAGAAGAAAATGCAGGCGGTACATGAGA

>BPA_2415

TATTTTAAAATATTTAAAAATACATGAACGTCCACGTGCTGCTGCTGTTTGTAAATTATGGAATTTGGCGGCAAAGGATACATCATTATGGCAAACAGTGCGCATGAAAAATTCAAAAGTATGTAGTTGGTCCGGTTTTGCAGGAGCTTTACGACGTGGCGAAACGAAACATCTGGATTTACGTAAAATGCTCCTATCTAGCGTTCGTAGCGAAGATATGTGGAATGATTTCTGTG

>BPA_2422

GCCGAACGAAATCAACGATCCGGAGATGTATTTGAAGATCACGATAACGATATGGAAGATGTAACAACTCCCCTACGTTTGGAGGAATATAGTGGTAGATATAAATTAAATATGGATAATAATTCATCAGTTGATCAACGTGGAGATTCATCAATGGTTATAGACGATGATGCTCCTCATTCACCCTACAGCCTGACAGGCTCACCGATTGTCGTTAGTAGTACGCCG

>BPA_2451

TCTCTGACATTTGTAGACGTTGAGCTACGGGTGGTATGCGCGCCAGTGTGGCTGGTAGTCGAGAAACATCGCTCTTCATATCTGAAAGTAATTCCTCCAATTGATTGAGCACTTTATGTAAAACAGCATTTGCTGGCTTATTGCCGGCCAATGATTCTTTGCTCAAATGCTGATGAGACTCGGCTAAACATTCTACTTCAGCAAAACGTGCATTTAACGACATTGCTGGATGATTAG

>BPA_2459

TTTAAGATAGTAAACTAACAATTCATTAAGGGACGGATCAGCATTTAAATTGACTAAATAGTATTTATTCTTTTCCACCATAATGCCACTAGCTTGTACACTAATACCCATTTTTTCTAGAGCCTGTTGGCGCTCATTTTGTATGCGTTCTGTTTTTACTAATTTCTCTTCCCAAGTTTGAGATATTTGTTTCATTAGAT

>BPA_2481

ACTGCTACAATTTGTTGACGAAAATCTCCAGTATCCTGACAAGTGCTCATAAATTGATTATTCAGATCTTGGCTATTTGAACGTGTATGACAACGTAAATCTACATCCATGTGTGAATGAATAGCACGCATACATTGTAAATTTTTAGTAGTCAACATGACGGGGGCTTGCATGACACTGTGAGGTAAAGAAGCACTTGGTAGAGGAGTACCCACTGCTACAATTTGTTGA

>BPA_2485

GCTGTATTTGCCGATGAGGCTAAATCCAAAGTTTGAGGTGTCACACGTTGACCTTCAATAATGTTAATGCCACCTGTTATTGTCGTTAAGGTGCTAGTTGATGTTGTGCCTATTTCCGTTATGGGATTTTCAACAAGTTGTTGTAGATTTCTTAACTCTGATGATCTTAAACTGGTGGATAGACTTTCACTTGAAGCCAAAGATGTTGATAAAGGTGAATTCCTTTCCACAGATGAGG

>BPA_2495

TTTTCAACGATATCTTTTTCCTGTTCGAATTGCCGTTGTCTGGCCTCTTTGGCAGTTTCCTCCGCCGAACGATTAACATTTTGTAACTCACTTACCAAATTACGCATTTCATCACGTTGCGCTCTTAAAGCTTCCAATTTTCGCTGCAACAATTCTTGTTCTGCCATAATTTCCGGACCATCATCACCCATCAAAGACGACAATTCATTTTGATTTTGTATGAA

>BPA_2508

CAAAGAACAACTATTAATGGTCTACTTTCTGCACAAAACATCCATTCCTTGGTCTTTTTATTGTGATGCAACATCCACATGGCCAAATCGTTTTCCAATATTTGCAATAACAATCTTAGAGCCATAAATACTCTCTCAAGTTTTTGATCTTTAGGCAAGGCATCAAACGTTTGTTTTTGCCTCTCCTTTTCCTCATATTCATTTTGATTCCAATAATCACTACATATACTTAACTCAGATGCTAA

>BPA_2510

TGCAATTGCTTGGCAATCTTTTCTTGGGCAGCCTTTTCACGACCAAGTTGATCGCAAGCGGTACGAGTTTGGTTCAACTCATTGTGGCAAGTCTGGCGATCATGTTCAGCCTTAGCCTTCATCTTGTTGAGTTGGTCAACTTGTTCGGCCATTTCAGCGATGGCATCGTTGTGCTTCTTGCGCAAGTTAGCCAAGGTGGATTCGTGTTGGATGTTGGCTTCTTCCAAATCGCGGCGCAATTTGCTGAGTTCAGCTTCACGCTTCTT

>BPA_2571

ATGTTCAAACCGAAGGGACGACCAGCGAATTGATCAGATGGCATGAAGGAGTAAGAAATTGTAGCCTCAAGTCCAGATTTCACTTCACGGTTGTAGGCAACAGCAGAGAAATTTTGAATATAGTAGTTAAAGTCCATGGGATAACGGAAAGAGGCTTCAACAGTTTCGACAACAAAATCGTTGGGCCCCTTGTTGGTAAAACCAATCAAGAATTCTACAGGTTTGCCACCGGGTAAATCTAATTG

>BPA_2574

TGCTTTCTTAGGAGCAGTATTTTCTGATATTGTTATATTTGCCTTGGCGGTATTCAGTTCAGTAGAAGCTTTCTTATTTTTTGGTGTAACTGGTCTAGGTGTTTTCTTAGTGGTTGCGGTTTGAGTTGCAGTAATAGCTGCTTCTTTTTTGATGGGTGTTATAGTAGTAGATCCCTTTGTAATTGAAGCTGTTATAGTTTTCGAATTACT

>BPA_2578

CTTTTGAAACTTGAACTGGTAGACTTAAATCAATACCACCCTTAACATTGAAACCGAAACGACCATGTTCATCGGCTAGTAAACGAATGGTTATTAAATCACTGCCACTTTCAGGAGCAATGGAAGCGCCATTAATGTAGGTGGTTATAGCAGCACCCATTTGAGCACCATTGGGACTGTGAGAACCCGAACTATAGGCAGGAACTGGAGACTCAAAACGTCGACTGCATTGTTCAATATAGCCAGTATCAAGTTGTATATCATATTCATCACTGTGCTGTTCCCAGGCTTTACG

>BPA_2579

AGGAATCCGATAGTGAATTCGATGAATTAGATCCAGATTTCACTGAATATTTATGGATGGAAAATGAAGAGGAATTTGATAAGATTGAGCTACAACGTCTTGAAGATGAAGAACTCATGAAAGAATGCATTGATGCAATGCTGGACGATGAACTCGAAGAACAATTAAACGAATGGGACAAAGCTAAAAACGAAGAACTTAGAGCAGCTCTTTCCTCTTTGGCAGTCAGTGATTGTAATGTGAGCAATTCTTTGCTCAATCCTTTGGCAGCTGAATTTATACCACAAAGTCATTTAATTATCGATTTGGGTACTTCATAAACCAAAAAAAAA

>BPA_2589

AAGAGTTCGTAACTTTAGTTGGGATGACTGGAGAAAGAGATTCCTAAAATACATGAATCACAAGAAATCACGTTTAACCGATTTGTTCCGTAAAATGGATAAGGACAACAATGGTTTAATACCCAGAGGAGAATTCATTGACGGCATTTTAAACACTAAATTCGACACATCCCGCATGGAAATGGGAGCTGTTGCTGATTTGTTTGATA

>BPA_2599

AATCTTTATAAAACAGATTTGAGAGGGCATTTTGGTTGTGTCAATGCAATTGAGTTTTCTCATGGTGGTAAATACTTAGCTTCAGGTGGTGATGATAAACGTGTATTAGTTTGGGATGTTGAAAAGTCTTTAGCTGGAACATCAATGCCTCGTGCTTTGGCCACAGAACAGGCCAGCAATATTTTCTGTTTGGGATTTGATACTAAAAATCGTAAATTAATTTCTGGTGGTAATGACACCTTAGCCATTTCCCACGATTTTGAGACGGGCAAATTAGATAATGTTTT

>BPA_2612

ATCCCAGTACACACAATCGGCAGCACCTCCAGCTAATGTACCCAACAAATATTGATTAATCTCCACAATCTTCTTCATGGTCTGTGAACCAATGAATTGACCACCGGTGGCACGAGAATCTACTGCCAATATAACACCACCTTGATATTTGAAGCCCAATGTGGTGGTACCGTGATCAAAATCAATTTTAATACCTGTCTTATCACTGTTGGCTTGAATCTTG

>BPA_2663

ACAACTACACCAACAACATCAACTACATATGCGTCCAAATGTAGGGCATATGGCTTCAGCTGGTCATCATATGATTATTAGCGCACCCGTTCAACCGCGAATGCCCCAATCGATTGGAAATCTAAGATCAATGATGCAAGGTTCTTATAATGGTTTACCCGGTGTCATACCAACTTCAGTGCCACAATTTAATAGTTTTAATCCCCG

>BPA_2669

ACAGAATCTGAAAATTCCGATTCTGATGATTCCAAGCCGGGCTGCAATACCGATGAACCGTGGATAATGACTGTTAAAGGTTTACATGTAAATAGTGATGCACATAAAATGAGTTCCAATCATTTCAATAGGCTCGAAGAGCAAGAATTAAAAATGGATAAACTTACCCAGGGTATAGCGGAAACATTAACGCCCGCCTCCCTTCTAAGCAGCAGCAAACATTTACAAAGCAG

>BPA_2696

CATCGATACACCAATCCAATCAATCTGAGAACACGTCAAGCATGTTGATTAACATAGAAAACGGAAGTGCTAATGTTATAAACGAACGTAGCATTATATCAGCAGATAATATACTTTCGACAGCTAAAACATTAGCATTAGCATTGCAACAGGTTGGTTTAAGTGCGCCAGCTCCAGATCCTGCTACAGCAGCCATAATA

>BPA_2710

GTTGATGATGTTGCTGTTGTTGCTGTAGATCAGCTGTATTATTGATGTTGCTATTATTACTATTACTATTGATAGTCATGTTGTTGTTATTATTGGCAGATGTTGTAGCATCATTTAAACTCACTTGCTGTTGATGTTGTTGCTGCTGCTGTTGTTGTAAAGAACTGGCAGTTTGTTTACTATTGGTACCACTGGTTGCAGCAACATTACTAGAGGCCACTGACGATGAGGTGGCTGGCAGTGGTGGAAAAGCTGCTGCTTCTAAATCAAATTGCGGCGGTTGCGGTGGTGCA

>BPA_2722

TATTGGAGACATTGAGAGAATTTTCAGATAAACACTTCTATATTACCAACAATTTGCAAGAATATCAAAAAATCTTATCATCTAATTTGGCTGTTTGGATTAATAAATTCATTTCCAATTTAAATATACACATAGAGGATGAACCCCCCGTCCAATATGAAGCAGAATTGCCACAAACTGAAAAACCCACACAAGAATTATCT

>BPA_2753

TTACTACTGAACTCATTGGATTCACCGCCGGAATTTCGATAATTTCTGGCTGTTGGGGTTCCTGGATCATCAATATTAAGCTTTCCGACTTTTCTGAACTTGTTGTAATCGTTATCGTATGATGCACTTGCGACGGTATCGATACAGATTTCTCTATTATTGGTTGTGTGCTTGTTGTTGTTGGTTTGAGGCCGTGATTGGCTTGATTGGGTTTCAGTGATGAATGCCA

>BPA_2759

CACATACCTTTTTGTATTTGACCCGTTTTTTCATGATAAAAAGTACCTTCACCATTTTTATAACCTCTAGCAAAATAGCCCTCATAGCGATTACCATTTGCATAAAACATTACACCCAAACCATGATGCACATCAATTTGCCATTCTCCCATATAAATATCACCATTTTTATACCATTCGATACCCAAACCATGACGACGATTATCCTTCCACCAACCATAATAAACAGCATCCTCATAGAATTGTTTTCCTTCACCCGATTTTAAATCATTCTTCCATTGGCCCACATAAATACGCTGCATTGTGCCATCTTTTAAACGTTTCCTCATCATGCCAAAACCATGACG

>BPA_2770

TTTTCTTATTTTTTAATGAATCGTGAATTATTTTTGCCAAAGGTACTTGAGGTTTTTGATCCATTAATTGTGATTTGTTTCTGTCTGATATTTGCTTATTTTTTTTATTAACTGTTGCATACAAAACCTCAAAATTTTTAACTGGGTTCAAATTCCATACTTCAGCGTTATTATTAAATTTTGGACTTTCAGATATTTGAATGCTGTCATCTTCAATTTCGGAATATATTGGATCAATTATTAGATGATCAACTTTTC

>BPA_2772

AATAGGGGCAACAATTTTAAATTGGCGTGTGCTAACTTTATTGGCGGTAAGAACACCAATTTCTCTTCTGGCCACCTTTTCTTTGTGAATTTGAACCGTTTGGGCGATATGATTCATTTGTGATTCCATTTCACTTAATTGTTGAGCCTGTAACTCCAATAATTGCATATAACTATAGGCCAGTGTATTGATTTGATATGCTACACTGGCTAAAGATTGTGTT

>BPA_2795

GCATACTTTTAGCGAGACGGGTGCAGGAGTGCCGGCATTTTTGGCAAAACTTTGGCGATTAGTTGAAGACCCTGAAACTAATAATCTAATTTGTTGGAGCAAGGATGGTCGTAGCTTCATCATACAAAATCAAGCCCAGTTCGCCCGGGAATTATTGCCACTTAACTACAAGCACAACAATATGGCTTCATTTATTAGACAGCTGAATATGTATGGTTTTCATAAAAT

>BPA_2806

CTATGACAAATACGAATACTTCAAATGGTGGCACCTCTAGCCAAGGAAAAGATAATTCTGATGAATCTGGTGCAGATGTTAAAATTGTGGATAAACGTTCATCACAGGATAAATTGCAGATCGTCAATGAACGTCAGAGTTTAAAAGTACCCAAATCACATAAGAAACATTCCAAAAGTTCAGCTGGTAATCCGGAAATGACTTCCACTGCTACTTCTACTCATGATGAGGGC

>BPA_2810

TTTAATGATGGTTATGAATGAATTTAAGAATTCTATTGAAAAATTTCCCGATTGCGTGGAATGCTATAGCTTAATGGCTCAAGTATTAAGTGATCAACAGCAATTTCAGCAAGCAGATCAATTCTATGAAAAAGCAATTAAAATGGCTCCAGAAAATGCTTCACTTCTGGTACACCGTGGCATCATGATATTACAATGGATAGGGGATATTG

>BPA_2825

ATCAAAATAGACATAACCTTATTGCCTTCTTGATCACCACGGTAAAATTGTTCAGCACGAGTCTTGCCCTGTACAACAGGATCGACAGCTTCCAAGTGAGAGGGATTGGCAACAACAGCCAAACGAATATTCTTGTTTGTTACACGGTTCAAACGTTCAATATAAGTACCCAAATGGTATTTTACATCACCGGAGCCATCATCAGCAGCTTCCAAACCAGCGAATTGTGTAAAGA

>BPA_2830

ACAAGTTTCTTGTAATTCTTGTTCATGTTGTTGCTGTTCTTGTTCGGGAGATGCAAGAGAGTTGTCCATGAAGCTATCAGTGGTCATTAGGGGATTAGTGGTATCAGCCAAAAGACTTTCTTCATGCTGTGTTTCTAATTCATGGTGTTGTTGTTCATGTTGTTCAAACAATTGCTGTTGTCTTTCTTCTTCAGGTTGTTTATCGATTTGTTGTTCAAATTCTTCTAAATGTTGTTGTTGTTGTTCGGCTT

>BPA_2844

AAGTTTAGGTTCTGATACTTCAATTCTTAAGACTACAAGTCAAATGAGTTTCGCTTCGGGTAAGACAGCTGTACCGGTCACTGAACTAAACACTGATAAACCAGCTGATGGTATGGTTATTGTCTCCACTAAAAAGGTTATTACTGTTGTAGGCGGCAAATCAAAGAAGGAACCAGAAACGCAATACATCAGAAGACAGGGCAAGGTAAATGTCATTGAAAATGTTGAAAAGTCATCAACCACTAAGAATATTAAAAATGTGGAAAACATACATAAGGAAGATGTTTCCATTACCCAAAATCGTATGACCACACGAAATAAAACTATTGTGGATGCTACACAGCACGATAGAAGTCATATAACAAATGGTAGCACAACTGTAAGTGGAATCAATAGAGTAACTTCCGCTAATCATCAACATGAACACACTTGTTTGGCCGACAAACTTGTATCTTCTAATAATGAGATACTCGCTGATGGTACAACGATAATCCGAAGAGCCACATGTATCAATCAAAGAAATGGCCATTTGAAGAACGTAATAAATGGTACTGATAGTAAGGGGCATGTAACACATACCCATGACACTGACGGCTCAACTATTACCAATAGAACTACGAATGTTACACAAAGAAATGGACAAAGTTCGAATATGAAGAGTATAATAACTGGAGCAGAAAGTAGTAGTCATGTATCACATACTCATGGAACCGATGGTACAACCATCATAAAAAGAACAACATGTGTTAATCAAAGAAATGGACAAAGTTCGGAAATGAAGAGCATAATCAGTGGTGGTAGTGTAGATAGAAGTGGCTCTGATTCACATACACATGGTACTAGTTCAAAGATAACTAACGTAAATGCCACGAATGGTAGTAGTGGTAGCAATATTATTCGCTCAAGTTCTACCAATTTAAGCTCAAACAGTGTTGAAAGTTCGACAGCAACCTCAACATCAATGTCGACCAGCAAACAGTTCCATCACCGTAAGACAGAATTCTCATCTGAATCTAATGTGGCCAACACAATATTGCAACGTAAAAGTACTCCATCGGAAAACAATCAATATGGTAGTCTCACATCGAATGCCAAGATACAACGCAAGAGTATTATGAACCTCAACGAACAACCTTCGAGCAATTGGTTCCCCAGCGAACGACAAAGTTACACCAGCATTCACCGCCAGAAAGCCGAAGCTGACTCAGTTGCCTTCAATAGTGTTAACGAACGTCGTAGTCTAACAACTAAACATCGCGAACTTAAAGATCAACAAGCAAGTAGCAGTAGCAGCAAGGGAACAACCGTGTACCATACATCCGCTCTGGATTTCCCCTCCTACAGCAGAAGC

>BPA_2847

TATTCTTCAGCTGCTTGAGACATTCTTTGTTTTAATTTTTTCATTTTCTTTTTGTAGCCACGAGTGTGTTCTCGACTGCCCACATCTGACACTGTCGATGAGAGATCACAATCATCAGGTTGTTTCTCATCACTTTCAGCCATTTTCTTCTACATTCCTGGAGGGTATAGTCAGCTCAAGAGCTCTTTGTCGTATATGTTCTATATTTTCTTCATGGCGTCTGGCCGATTCTTTTTGTTTTTGTAATATTTTTCTTTGTAATTCTTCAGTGGTTTGTTGCTGTTGTACTTGTAGTGCCAACAAACGTTCTTCACGATCTCTGGCCTTTTCACGAGCCAATGCTTGTCGTTGTTTTTCCTTTTGTTCTTGCATTTTACCAATTCTTTGCTCTTTTTCCAAACGCGATTCATTCATTTTCTCCAACTTACGTTGTCTTTCCTTTTCCAATTCTTGGCGACGTCTCTCGACTGCTGCCTCTTTAGCTGCCTTTTCTTCTAATCGCTTCTGTCTTTCTTGCTCCAAATCCTGCAAGCGTCCTTCGGTTTCCTTAGAAGACTCAATAAGATCTAACCTCTTGTTTTGTGCTTCTA

>BPA_2848

CAATTAGTTGGTCAAGCGACACAACAAATCGTTTGGCGACAAATTGGATCAACAAATGTTACAGCAACTCCGGCCGGCATGTTGACAGGGACAGCGAATACTGATGGTACGGTAACGGGAACAGGCAATAAAATTGTTTGGACTAGTCGACCGACACAAAAGAGGCAGCTCAATGGCCCGGAGAGTACAGGAGCATCATTTGAAGAGTACAGACATCAACAAGTTATTGTTAAATCGTAAATTTTCACAACGAAAAATTTTACAACAACCACAATTGCTGCAGCAACAACAACAATTACAACAACAGCAGCAGCAACAAACGCAAGATGATGATTTAACAAATGTAAATGTATCAATTAGTGGACAGGATACATCAACTACAACAGCTGTAACATATAATACACAACATCAACAACAACAGCAGCAGCAACAAATCCAAAAATTACCTGGTGGTAAAGTTATTAAAATGCCCTCATTCCCACTCATAGTTTCAGAAGTGAATCTGCAACAACAACAGCAGCAACAACTGCAG

>BPA_2851

CAATTGCCCCAACCCAATTCACCTTATTTGCAAACAGTGACAAAAACTTCTTTGGCTCACAAACAACAAGAGTCCCAACATATCGGCTGTGGCTACAATAATGCCGCCAAACCTTTTGGCAACGGCGTTGATGGCAATGTTAAGAGTATTGTCAATAAACAATACAACAGCCCAGTTGGCATTTATAGCGATGAATCAATTGCAGAAACCCTCTCGGCTCAGGCTGAAGTTTTGGCTGGTGGTGTCCTTGGTGTGAACTTCAAGAAAAACGAAAAGGAATACCAGGCTGATAAATCTGAAGTATTGAAATTCTTGCGTGAAGAAGAATCTGGTCCCTCTACACCAGAGCCACATACACCCTCAAATTATTATTGGACACGAAGTCATGCCATTGGAGGACATCATAAAATTGACAGTGATGATGAACGTATTGGTACACCATTGCAACAAAACACATTGGCACCGGAGGCTTCACATCGCCCCAGCTTACCAACTCCTAAATCACCAGAACCACCTAAACCCATTGTCGCCGATATACCAGATCCCAGACTAATTGTTATGCCCGTTTGTCCGGCATTACAGACACCAGAATACAAGGAAGAATTGGAAGCTGCTGCTGTGGCAGCAGCATTAGGACC

>BPA_2853

CTTTCTTTCGTTATCCTCAGATAATCCAAGATCGCGTCCATGAGCTATAGCAAGTCGGTTTCCTTGAACTAAATTTCCAACCATAACTGAAGCAGGTCCCACGGAAGTGTCCTGATGTATTTCTCTGGGTTTGGGTAAATTTGTTACACACGTATGTGGGCACATTAAAACATTTGCGGGGTGCAATGTACCATCTAAAAACCTACGTCGAGCTTCGCATAAATTAATCCCTCCCAGAGGGGTTTTAGGTAGGTGATTAGGGGGTACAAGTGCCAAGCAGTATATACCGACTTGATGTATTGAATCAACTGCCTGTAAAACTCTTGACATCCATTGAAAACTTTCTTCTTCGGAACAGTCAGGTCGTTGTTCTGCAATTACACAAACGCGTTCGTCTCTTAATACTTTAATACTAAATACTGCTATTCTTCCCCTATATATAAAACGCATGGGTTCTACAGCTAAAACAGTTGCAATTATATCATCAGCATTGTGTTTACGTCCAGTTACAGTCATTAGCCCATCACGAGATCCACAAACGAATACAAGACCACCAGGTCCCAAAAAGCCTAAAAGACCCGAACGAACATAATTATCATCACTGATTGGCTTCATTTTTCCGATCACACCGTCTTTTTCCGGTTCAGGATCCTCTAAAAGTGGTTGCACTTTAAATGTTGTATTAGTTAATCCTTCTAAACCAAAGTAACTAGATCCTGTAGATCCACTTGTAACACATATTTCGCCAACCTGGTCCGTTTTACAGAGTACTGGATGACCTTCGGATCTTACTACTACGATAGTAGCTGTAGGCATTACTTGCCCACAGTCTTGAAGAGTTAAAGATGTTAATGAATCTTCACTATCTACTCTTACAACTCCATGCGATAAAGCTGCCATCGAAAGTACACCTCTACCCGTAGCTGACTGATTAAATCCACTCGAAGATCTTCCAGGACGACGAAGAGATACTGTAAAGACTTCTGAACTACTAGCACAGGGACAAATTGCATCTGCTCGTAAGCCTTTTGCTTGAAAAACATTTAAAAATTGATCACATGACGATAAAGACCAAGGATTTGCTCCATCAGCAACGAGAAGCATTCGCAATGAAGACAGCGATATATCTTTATGATCTTTTGTTGCTAACAGACCCCAATGCAAGTCTCGACTTTTAACTAGGCAACATGAAGCTCTATGCTTAGTAATTAACTGCATCCATGATGATGGCCTAAGTTTCATAAGGGCATAAGGAATAAATAATACATGCATTCCATTCAAAACTGATGTAAAAACAGCATGCCAAAGACCAACTTCCCGTTTAAA

>BPA_2854

ACTCGTGAGCTCGCCGAAGATGGTTACTCTGGTGTTGAAGTACGTGTAACCCCCGCCCGCACTGAAATCATTATTACTCTGGTGTTGAAGTACGTGTAACCCCCGCCAAGGGTCGCCGCATCCGTGAATTGACCGCTATGGTTCAAAAGCGTTTCAACTTCGAACCCGGCCGTATTGAATTGTACGCCGAAAAAGTCGCCACCCGTGGCTTGTGCGCTATTGCCCAAGCTGAATCTCTCCGTTACAAGTTGACCGGAGGTTTGGCCGTCCGTCGTGCCTGCTATGGTGTCTTGCGTTTCATTATGGAATCTGGCGCCAAGGGTTGTGAAGTCGTTGTCTCTGGTAAATTGCGTGGTCAACGTGCCAAATCCATGAAATTCGTAGATGGTCTTATGATCCACTCCGGTGATCCATGCAACGACTACGTTGAAACCGCCACCCGTCACGTCTTGTTGCGTCAAGGTGTTTTGGGTATCAAGGTCAAAATTATGTTGCCCTACGACCCCAAGAACAAGATTGGTCCCAAGAAACCATTGCCCGACAATGTTTCTGTTGTTGAACCCAAAGAAGAGAAAATCTATGAAACCCCTGAAACAGAATACAAAATGCTTGCTGTTGTGCCCATCTATGATGAAGCTGCTGATATGTAAACTTTCAGCGTTCCTGTAATATTTTTTAATTAATATCAACACAATTTTTTATAAGAATCACAAGCGTAATATACAAAATGCTTGCTGTTGTGCCCATCTATGATGAAGCTGCTGATATGTAAACTTTCAGCGTTCCTGTAATATTTTTTAATTAATATCAACACAATTTTTTATAAGAATCACAAGCGTAATATACAAAATGCTTGCTGTTGTGCCCATCTATGATG

>BPA_2856

CATCTTCTTTAGCGTGCATACTGATGAGTTCTTGTTTTTTGTTGGCAATACGCTCTTCACGGCGCTTACGGGCTTCACGTACTTTTTGTCTTCTGGCTTCAGCTTGATCAGCCAACATTTTGCTACGTTGTTTTTCGGCCTTCTTCTTGTGAATGTATTCCATAAGGACACGCTTGTTCTTGAAAACGTTACCTTTGCACTTCATGTACAAATCGTGGTACAAATGACGATCGATTTTCTTGCTGTCACGGTATTTCTTCAACAAACGACGAAGCACACGTTGACGTTGCATCCACAAGAATTTGGTGGGCATACGGGCATTGGCGGTACCTTTACGTTTACCAAAACCACAGTGACGGCCCTTGCGACGAGCTTCAGTGTTCTTACGGACACGGTAGCGGGAGTGTACAACCACGGGTTTTTTAATAATCAAACCATCCTTAATCAATTTGCGGATGTTTTGGCGGGAGTTGGTGTTGGCAATCTCATTTATTTCATTGGGATCCAACCAAACCTTCTTTTTA

>BPA_2858

TTTGTTCAGCAGGAGCATTGTTAGTTACCGGAGCTTCTATTACAGTGGCCAATTGTAAATCGGCTTCCAATTCGTTAATACGATTCTTTAGGTTCATTTCAGAAGTTCTCAATTCTTTCAATTCTGCTTCAGTTTTTTCTATATAAGCAGTATGAACTTCATGTAGATTTTTAATTTCTGTTTCCGCACTTTGAGACATTTTGTAGTATTGATCGGCATGTTGTTTAGCTTTTGCCAAATTTGCTTGCAAAGATTCGATTTCAATCACATGTTGTTTAGCTTTTCCCAAATTTGCTTGCAAAGATTCGATTTCAATCTTCGCTTCCTCGTATTTAACCTCAAATTCACGTGCTTTTTTGTTGGCCAACACAATGGGGTTGTCATTCTTGGAAGGTGTAAGACTCTCCTGTAGTTTCTTACTTAATTCATCTATTTGACCAGCTTTTGTCTGAAGCTCTTCACGTAAGCTAATTAATTCTAAACGTAATTTTTCAGCTTGTGCCTTTTCCTCTTCCATTTGTTTTTTGGCCGAATCTGCTTGACGTTTCAAGTCATTAGTTGTCTCGCGGAAACGATCTTCTTCCTCTTG

>BPA_2862

TTCGTATAGAAAGGTTCATTTCTAAATCGTTACGTGGTAAGCGGTAAATCTGATAATATGATATATCTTCGCATTGTAAATAATTTAAACCACGAAAAAATTTATTTAGGACAACACAACCATCAGTTAGTTTATAAAGTGCTCCTCGTGGACAAATCGCACATTCTTCACTTATAATAAAGACAATTGCTGCAAGTCTTTCCTCTTCTATAAGTTGAATTTCTTCTGGTAAACTTGCAGAGATTACTTGATTAGCATCAATATTGAATATGGGGTCCAATTTAATACGTCTTATGATACTAGGGTCTCCCGAAAAAGGTTCGTGACACAACATTGCGGCTTGAAAAATTTTTGGATTTTGTACAAAAGATAAAAAAAGCCACTGATAACCATCGACGCTAAAAAAGTATTTGCGGTCTCTTAGGCAATCTTTGGTATAACCAAATGCTATATAATAGTCTTTTATTATTCCATTTATTCGTCCCCAAAATAATATTGATTCAAAACGATTTTTATTTTGCAAAACTATTAGA

>BPA_2866

TACCCGAGAGATGGAGCGATTTTGGTGATCGCTTGCGTCAATATTTGGTATCTTACAAACGCTTTGGTGATTTGGGAGCTGATAGCAACTACTATTTCAGACGTGTCAAAGAATCTACCGAAGATGTACCCGTTGATGCAGGTACCGAAGATGAATCTCATCATCATTATGGTGTTGATGAAGTTGAAGCTTCTGGTGAAGGTTATGTAACTGAAACCAGTCCCGTTTACACTAGAGGTCCTGAGGTGGGTTGTCGTGGCGATACCACCTTCACTTGTCCCGAAAGTGGTCGTGTCATTTGTGAGGAACAATTGTGCGACGGCGAAGAACAATGTCCCGATGGCGAAGATGAATTGAACTGTGGTACTGATGGCGATGGTGAAGATGGCGGCGACAATCAATATGAACCTGAACAATGTCAAGATAATGAATTTAGATGCGATAATCGTTGTTTGCCCAAAGAATATCAATGCAATGGGGTTCATGAATGTATTGACAACACTGATGAGCTTAATTGTCCCGAAAAAGAATGCCGACCTGATGAATTCAAATGTCGCTCTGGTGATTGTATTGATGGCAGCAAACGTTGCAACCGTGTCCAGGACTGTCCCGATGGTGATGATGAAGATGAAAGATGCCTCACTACCGTTCTCACTCCCGAAGATTGTGAAGAAAATCAATTCTTCTGTGATGATGATTGTCATCCCATTTCGATCCGTTGCAATGGTCAATATGATTGTGCCGATCGTAGCGATGAAGAGGAATGTTCAAGACCCACTCGTCGTCCACCCACTTATCCTTGTCCCCAGCATACCTGCCCCGATGGTCAATGTTATA

>BPA_2867

CCTTTACCTCCTTTTCCTCCTTTACCTTTGGCTTTTCCAGACTTTCCGGATTTCTTTTTATGTCCACCTCCCTCTTCACCGCTCGAAGAGGTTTCAATACCTTCTATTTCTTCGACTTCTCCCTCCCCCATAGCTTCCTCTTCTTCCACTATTTCTGCTTCACTAGATGATGTTCTCAGTTCCATTTCAAATACATCCGATCTTAGATCTTCATCTTCATAATCACTTATATCAGCTAAAGCTTTAAAGCATATATCTTCGGCCTCTTCAAAGCATATGTGATCATTAAGTGATATTGTTTCACCTTTCGAATCCAATTTCGAATCCTCAATAGCTTTAAGATTTAATTCTTTACGAGTTAATTTCTTTTGTATATTACGGACTTTTATGGCGTGAAGGGAAAAGCTACTGGAGCGATTAGAATCCATTAAAATGTGATTGAAAACATTAAGAAATACATCAAAAGATCGGAACATTCTTAAGGAGGCTGATTTCTCCATTTGTTCATAGACTTGGGATTCAAAAGGTTTGCCCATGATACGATGTCGAAAGGGATCGAAAAGATAAATAAAATCATGTTTTTTCCAAATGGCATACATTTGATAATTGATTTGCATTATCAATTTATGCGCCCCGATCAAAAGCTCTTTTACAATTTTCAGCCAGTTCGCTAAGTTTAAATTCGGGAATACAATGCCATAGGCCCTCATATTGACGGGCTATTATTTCTATGGTAATCTGGAATTGACCTATTGAAAAGTATGTGGGAAATTCTTCCACATCTACCATCTCACTGTCTCCACATTTGGCCCAAACGGATTGACAAAAGTTCACACCGAAACATATAATCTTATCCAACATTTTCATATTCCAGGTGGCAGGATGATCTATTTTGGTACATACAATGGCGGTAATGGCCACCGGTAAAGCACTACGATTTCTTAATAAATCTCTTCTGTTCAGGGTTAAATGTAGTGCTGATCTTAGATAAGCATAATCATCATTAACCACTTGATATTGATGTGATCGTTTAGCATAATCTTGCTGTATAATGCCCCCTGATGGGGTAACAACTTTTACCACCTTAATTTGTCTTATGCTTAAGGGATCTGTCTCTTGTAAACCACTTAAATTCACCACCAAATGTCTTACATTCTCCAAACAACTCAGACATATTAACATGGCCACTCCCTTATCTTTGTCCGATTTAAAATCCGTAACTCGTCTGCCACAGACATCGAATACAAAATACGCTTGCCTGGAATGCCATATTAAAGCGTAAAAATCTGCCACTGTTATCAAAGCATATTTGCGGGAACGTAAAAATTGTCTCATAGCTTTTTTGAAATCTTCCAAAGTTAAAGCCACTGCATCACCATTAGCTCCTGATTCTTTTGTGTTCCCCATTTTCATTTTAAATTCTGGCATTAATTCTACGTGAAAACTGAATCCAGATAACTGAAAATCTCTTTTGATGTTCATTTCATTTGGTGTATAAATATCGCTTGGGGTTTCATAACTATTTTCTTCTTCTTTTTGTTCCTGTGATGTTGTATATAAGTTGAGACCTTCTTTCATTATATCATCGACCATAAGAGGATTCCATAATATTTCCTCATGCAAATGCAGTCGCGCAAAAGATGCAAATGCAGTCGCGCAAAGGCCTTTAAATAGGTAGCAAATTTAGCTTGTGTAATATCGGCAACTTTCGCCTCGGTTGAGAAATTAACATTCGAGGCAACAAGACCAATAGGTGAACCC

>BPA_2869

CGGCATTGCCAGAGATATAGATCAATTCGATAATAATCGTGTACTTTTCACCGATCTCAGCAAGAATGATGTTAGTATACCAAAAATGTATTTGATATGCTATGCCGTGCGCATTGGGTCGATGGAAATGAAAGAGAACGATTCGAAACGTACCAGCATAACGAATACAATGTTAACTGCGGCATCGAAACGTACCAGCATTACGAATACAATGTTAACTGCGGCCAGCAAAAAACATTCACAACTTTCGATAAGTTCAAGTGGTTCCTTTAGTGGTTCCGAATACATTATGAGAAGACCATTTGGCGTAGCCTATAAGGATCTTACTCCCATTATAAATAAACCAGAAGATTTCAAAGGTAACCTAGATATGCCTTTCATATTATGTGAGAAAGATAGTTTGGATGGCACTTTAAGGAAATTAATCGCAAACAAAGATATAGGAAAAATTGATTCACGTATGTGTGTCACAGTGGAGGTGCTACAGGGTGATATAAAACAAATCAAAGAAGAATTTCCCCGACTTGTACATACTAATGTGC

>BPA_2874

AAATCAAAAATTTCTTTTTCTTTGTGATTTATTAAAAAATGGATCACCTGACGGTTTGCATTGTGCATGTTTGGAGGATGTTTGTCACATATCTCACATTCGCAGTAGAATCATATTTCAAAATTATGGCTCAGGCACAGATCAACTGAGTTCCATAATGCTAAAAATCAAAATTTTGTTTTGTTTTTAATGTATGAAAAACATGATCACCTGAGATTGCATAATAGAGTGCACATCAATCACATCTTAAACTTCTTTGTTGTAGAGTAACACCAAGTTTGTTTTTTATTATGGCTCTTGCACTGTTCAACAGAGTCCATTATGCTACAAAAGAACAAAATTTGTTTTGCATTCGGTAAAAAGTGAAAACCTGCCTGTTGTCAACATTTTAATTTCGACATCAAGTTGACTCCACATGTTAGAATCATTAAATAACACTTTCAACCATTTGATCACTGAATTTATTCTCAAATTATTATGGCTCTGGTACCGATCAACGAGTCGCATAATGCTAAAAATCGAAAATTTGTTTGATAATTTTATGAAATTCAAAACCTGCCTGTTGTACGTAAAACATCAATATTTCTTTGATTCGTTCTCAAATTATGGCTCTGGCACCGTTCAACAGAGTCCATAATGCTAAAAATCAAAACAAATTTGCGTTTTGTTGTGTTCAAAATAATACAATTGCCTGTTTTTTCAAGTAGTGACGTCGTTAAGTGTTTTAATAAAGATCCCCAAAACAACCGAGTCATAACGCTAAGACTTCAAATTGATTGGTGCAAAATTATAAACCTGCTGCCGCTTCTTTATAATTTAAATCCATAAGCATGTGAGGATATATTTCAATAGATTGTTCTGTAAAAATCTGATTAGTGATAAAAACAATTTCATTGTTTCCTTACACATCTTCATGTTGTTTCGTGACCCATGGAACAAAAAAACGAGTTCAAGAATTTACGCCAAATGTCAAGGGTACGTAAACAACAGGGATTGTTGTTCCATACTTCTCCAAAAGTACTTCATAATTTTTTTAAATTGTAATTGTCTGCAAAAATTGTATTAATGCTTAAAACAAATTCAAGTTTCCTTACACTTGTGATGTCTGGCTCTTGATTTACTCATATGCAAAAAAAAACCACACCTTTTAAACATTCAAAGCCTCATAAGGGGAAAGTAAGTGTTATATATTATGCTCAGGGACTGATCAACTGAGTCATAATGCTAAAAATTTAGAATTTTTG

>BPA_2898

TGCAGGCTTACCACATCTTTCGATTTATTTCGATGTGGAGCGGATGATATTTTAAATTCTCCCAACCCAATCACTACCAACGATTGTTGGCCTGGTTTGTATTTTCCTCCTTGTGGCAATATTAATATGTTTGGCATTAACGTTATATCAACCTCAACAATTGCGTGACTTTCTATCATATATTGCATTCCGGTAGCAGAACGCTCTTTAAAATCAGAAATTTTTAGAGTAGCTGCACCTTCTAATTCTGAAAGATTAACT

>BPA_2907

TTCAAGTTTAACTTCTTGTTTAATTTCAATATTCTCGGAAGAAGTTTGGGGAAGTGAAATCACGTTTGCTTCTTCTAATTTCAATTCTTCTTGTGGTATCATCTCCTCTTTACGATCTTTGTGTTGTGTTACAACATCATCATCGGCTGTCATACTTTCTTCTTCACGTTCTACATCAATTTTAACATCCTCAGTTCCACTAGTA

>BPA_2913

TCACCCTCAACATCATAATCGGTAGTGATGCCCATGTAAACACGTTTACCGGTACCGTTTTGATTTCTGTATTGTTGCAAAGAGGTGGCGCCAACAGCGGGGAAATTACGTTCATTACCGGGAACAACAGATTCAGCGAAATAACGCACAGCACGCATGGAAGCTTCAACAACATTTTGAGCACCAGCAACACCATCGGTGGGTCCATCAGGATAGA

>BPA_2925

GAAGAAGAAACAGGAAAGATCGTGGAAGAGAAAAAGAAATCCAAAACTGAAGCCGACGAAGTTTGTGAAGGAACACAACAAGATGATGAAATATTGAAAACAGAACCTAGTGCACCAATAGCTACGATCGAGGATGAAGAGGACAAGGATGAAGACTCGGCTATGCATACAGCTGAATGTGAAAGTGCACCATTAGAAGAGA

>BPA_2936

GCCATGTGGAAGAACGTATTGAAGCTTTTCGTGGTAAAATGTTGCAGATATTAGAAGAAATGCCTCAAGATGAATTTGAACATGTTAGAGAATCTCTAATCAAAATCAAACAAGTGGTAGATATGTCCTTAAACGAAGAAGTGGCCCGTAATTGGGGTGAAATAACTTGTGAGGAATACCTATTCGATCGTAAACGTAAAGAAGTTGAAGTTTTAAAAACTTTAACCAAACAAGAAATTATAGATTTTTGC

>BPA_2941

TGAATTGTTGGAAATTCTTGGAAGCATTATTAATGGTTTCGCTTTACCATTAAAGGCCGAACACAAACAGTTTTTAGTTAAGGTTTTGCTTCCTTTGCACAAAGTTAAATGTCTGTCACTGTATCACGCTCAGTTGGCCTATTGTATTGTACAGTTTCTCGAAAAGGATCCATTTTTAACGGAACCCGTAGTAAGGGGCCTTTTAAAATTCTGGCCAAAGACTTGTTCGCAAAAGGAGGTAATGTTTTTGGGTGAA

>BPA_2954

GCCGAAATGTGGTTGACGTGGTGATAATTGGGCGGCATTTCCACTTTGGAACGCCATTTGTTGCTGATGCTGTTGTGCTGACACTTGTTGCGACAACTGTTGTTGTTGTTGAGGTGATAATCGCTGTCCACTTTGAGAATATTGTGGCCCATAACCTACTGAATTAGACTGTGGACTAAACGGAGCAGTTCTTTGTCCTGGACTCA

>BPA_2962

CAAGCATTGTACTAGCTGTTGGGCATTTATGCCGGCTGGCGCTATAAGCAATCGTTCATTTGGTCCAATTGAGGCTGTCGTTTGATTCGCATTTAAGGACGTAATTTGATTATTTAAAGGATTTAAACAGAAATACTGATGTGTGCTGGGATCAATTTTGGGCAATGCAATGTAACGTTGCTGCGATAAGGTTATATTTGAAGGAATTTGAGA

>BPA_2976

TTGTCAATAATGTGGTCGATATTTTCAGCTGAACCAATTGGAGGTTGATTTGAGCTTGATGGTTGATTTGTTGACGATTTGGAATCATTATCGGTTTTCGAGTCTTGCTGTTGTGATTTTTGTTGCTTATTCTTTTCAGAAGGTGGCAGTATGGGTATAGCATTGGTAGGTCTTCTTTGGGGTAGTGAAACAGGTCGTGTGGACGTCTGTGTGGGAGGAGGTACGAAGTAAGTAGTACCTCCTACGGTATTATAGTTTTGA

>BPA_2994

GAAGATGCTGCTACATTCTCAAGTTTACGTGCAATGTTCGCTGCTCGTTGCGAAGAATATGTTACACAAGTTGATGATCTTAATCGTCAATTGGAAGCTGCCGAAGAAGAGAAGAAGACACTTAATCAATTGTTACGCTTGGCCGTACAACAGAAGTTAAATTTAACCCAAAAATTGGAAGAAATTGAAATGGATCGTGAAATGCGCAC

>BPA_3007

CTTTATTAATCGATCACATACACAAAAGGCTAAATGCTTTAGCGTCTACCATAAACATTTTCATCTGAATAGGCAATGTAAAGGAAATAATCTTCCTCATGATGTTCCTGATACAAGGATCCCATTGTGGCGGATGTTGGTGGAATTACATTGTTAACAAAGAAAAAGAGGGCATCTTCTGGTCTCAATTGTATACGTTTACGAATCAAGAAATAAAATTGTCCCACAGTTAAATCAGAAGGTACTAAATACTTTTTCTTATCCAAATCACCGATGCGTGCTTTTGGGGCTTTTTCTACAATAACTGGCACACGATCAGGATATTTTCTGCGAATTTTATCA

>BPA_3043

TATTAATTTAGAAACTTCTGTCGGAAAACTGGCGGCATACATGAAGGTCAAAGCACCGCCTAATGAATGACCCATTAAAGTAACACGCTTCCAGCCATATTTGCGTATAATACGCCGTATAAGGCAAATACCATCCCAAAATAAGAAATATTGCATACCTTTGGGATAATGTGACGATTTTCCATGACCTGGTAAATCGATGCAAAGAACTGGTATATCTGGTGGC

>BPA_3090

AAAATCAGTACTTTCTTGGAACATAACTGAAAGTGATAAAATTCTTTATGTAGCTCCTTTATATCAAAGATTTCACAATGATTTCATATTCAACTTACTTTTTCCCTTGAGTGTGGGCAGTCATGTATATATTGTTGAATCATTTGATGCAAAATCTGTTTGGCGTATAATATTGGGCATAAATGTTGGTCTTAAGGAACGTATCAATGTATTTGCGGC

>BPA_3093

ATTTTCCAAAATCGCTTTATTTGTGCTAGAATTTGGTTTAATATCATTTTCTAACTTTGAATCTTTCTCATTTTTCTCCGACTTTTTTTCTGTTTCTTCAGCACTAGCACCTCCATTTCCATTATTATTACCACCACCTTCGTTGCCACCATTTTCTTCTTCGTTTCCACTTTCATTTTCTTCGTCATCTTTACAATCTTCAGTGCGATTAGCAACTAAATAATGATACTCCAGTTCTTCAAAATCAAAC

>BPA_3121

AAAAAATGTTTATATTAATTATTGGAATAAGACTGGTGAAACATCAAAAGACACTGATTGGTTAGATGAATGGAACAGTCGACCAGATCAACAACCACCCAAAGATTGGAAATTTGAACATCCACAAAATCAACAGAAAAAGAAAACAGCTGGATATTCCATACGTTTAACTCGAGTGGGCAAAAATTCATTATTTTCTCGAGAAATTTTATATTCCC

>BPA_3141

TGCAACTGGCTACTCAGTAAAGTATCCAGTTCGCCATGGTTTGGTAGAAGACTGGGATTTAATGGAACGATTTTTAGAACAATGCATTTTTAAATATTTAAGAGCTGAACCTGAAGATCATCATTTCTTACTTACTGAACCTCCATTAAATACTCCCGAAAATCGCGAGTATACTGCTGAAATTATGTTTGAGACATTTAATGTTCCTGGTCTTTATATTGCTGTGCAGGCCGTTTTAGCTTTGGCTGCCAGTTGGGCTTCCAGACCAGTTGAGGAACGTACCCT

>BPA_3144

AATACCTTCACCCTTGGTATCATACTGAGGACGACCGGCACGACCCAACATTTGCAAAACATCCAAAGCGGACAGTTCTACCCATCTACCTTTTTCAGGATTATAAACTTGGGTCCCTTTAATTATAACAGTATGAGCTGGTAAATTAACACCCCAGGCTAAAGTTGCTGTAGAAACTAAAACCTGTATATGACGATCAGCAAACAGATCTTCAACT

>BPA_3146

CGATTAATAACACCAGCTACAAAACCCAAACGACCACGATTTTGAGCAGCGGCAGCATCATCAACAACTACACCACCAACACCACGATTGCTGGAATCCAAATTCAATACTTCCTGATCGGAGAAGAAACCCTGGGTATTCTCCAAGACCTTACGCAATTCTGTCAATTCCAAATAGTTGGATTTCAAATTGACTTCATTTTGAGCCAATTCAAGAATTT

>BPA_3160

GTATTTAAAATTTGTTTGGAATACTGGAATAGTTTAGTTGAGGACTTGTATACCAGTGAGTCGTATGGCGGTGGATCAAATGTTCATTTAGGTCAAACTTTAGCCCGGCGTAATGCTGCTTTTCCACGACGTCGTTTTTATGCAGAAATATTATCAAAAATTCGATATATTATGATATCTCGTATGGCTAAACCAGAAGAAGTCCTTGTTGTTGAAAATGAAAATGGAGAAGTCGTTCGCGAATTCATGAAAGATACAAATGCC

>BPA_3164

CAATCTTTGGTTCAAGTATTGGCTTCAACAGATGTCAATGTTGTTACATGTGCAGCTGGCATACTCTCGAATTTGACTTGTAACAATCAACGTAATAAGGCAACGGTTTGTCAAGTCGGTGGTGTTGATGCTTTGGTACGTACGATTATAAATGCTGGCGATCGCGAAGAAATCACTGAGCCAGCCGTTTGTGCTCTACGTCATTTGACAACACGTCATGCTGACTCCG

>BPA_3168

CAGCCATAATAACTTGGAAGGCATTGATGACACCATCCAAAACATTCAAGCGACCATTGTCGGCCATAAGACCCAACAAATTGCCAGTGGAAGGAGCCCAACGCAATTTTGCAGCACTTTCTTTCAAGGCGGTGGCCATGACCTTTTTGTTGATGATAGGGCTAGTAACATATTCGCGCAATTTCTTATCGGTCTTAAGGGTAGCTTGGAAAGCAGCTAAATCCTTTTC

>BPA_3177

AGCTCGTCCACGAAGTTTGTTAGCTGCATTTTGAGCTGCTAAAGCGGCAGCCTGCAGATTTCTAGCCTGTTGTTGTTGCTGTATTGAAGCCAACGCTGCCTTTTGTGCAGCTGCTTGCGCTGCCATGGCCTGATAGGCAGTTGCTGTTGCAACCAATCCGTGACGCGGCTTGAGACGTGGTATTTTTGCAGGTGCTTCCCAATCATTAGGTGGTGCCATATTTAATTCGGGTCTGAATTTTTTCGCACAAACAGGTTGATGACGGGCCAATTTTGCTTTTCCATTATCT

>BPA_3187

CTTGTCCTGTGGTACTACTCATACTTTTGTCGTACCTTTGGATTTGGTCAAGTGCCGTTTGCAAGTTGATGCTGCCAAATACAAGAACTTGTTCCACGGTTTCAAGGTTACCGTAGCTGAAGAAGGTGCCCGTGGTTTGGCTAAGGGTTGGGCTCCAACATTCTTCGGTTATTCTGCTCAAGGTCTCTGCAAATTCGGTTTATACGAAGTCTTCAAAGTATACTACTCTAATATTTTGGGTGAAGAGAATGCCTACTTGTACCGTACCTGGTTGTATTTGGC

>BPA_3190

TGGTGCAGCAGTTTGCAGTGTGGCTACTATGACCACAAACCTTGCCGAATGCAGTCTTAACAATCATAAAAAACCACCTGCCATTGCAGCTTTAATTGCCAAAAAAGATACGCCAAAAGATGCTACCAAGCAAAAAAATGCCTCCACATCAACTAGCTCAACATCTACAGAAAATTTAGCTGTCACCGCTACCATTCCCGCCGCTGTAGTATCTAC

>BPA_3227

AGAAACCTGAGAAAATACTTTTTGATATTATACCCGACAAACCTCTTATTGTACCCGAAGATATGCACAAACATAGAAAATCTCATATGAAATTGGTGCCACTAATTGAGGGTTTTGAAGTAAAAGATACCGATAATGAGGAAACTACTTCCAAAGCTGAATTACAGAAAAGGCCCAAAAAATTCCATCGTCGTATTAGACGTGATGCCAGTCATGTTTTGCATAAACGTAATCCTTATTTTAG

>BPA_3233

CAGCAATGAAGGGAGGAAAACGAAGTCAGAAAAAAATAAATCAACAGCTTGCAAATCAATGAATACATCTCTTTCGAGTAATTCAATTAAAAATTTAAAAGAATCGCAATACTGCACAATAGAAAAAAATTCAATAATAGCTGCAAGAGTTAATCCAATTTTTTTGTGGGTGAAGCAAGATGACACACGTATTGTCGAAGTT

>BPA_3243

GTTTGGTGAGAAAAGTTTTGACAGCATTAATAACAATCGATGTACATGCCAAAGATTCCGTTAGAATGCTGATTGATAAAAAAGTTGTGAAATCCACTGATTTTGATTGGTTAAAAATGTTACGTTTCTATTGGTCCCCAGAAACTGATACGGTATATTCGAGAATGACTTCGGCTACCATACCTTATTATTATGAATATTTAGGGGCGGG

>BPA_3280

AAAAATTAAACAAGAAAATAATCCGTTATCAAGCACTATCAAGCAATTGATGCGAGGAGAACTATTTCACGGCACGTATTGTATATGAGTGAGAACTTTAAGCTCTAGATTATTGGCGGCGTTACCAGTGCGTGAGCCTCGGTAGCTGGCAGGATCCTTAGTTAAACGTTGTTTACGTAATTTCGTGGACAAGGGTCTTA

>BPA_3282

TAATGCGCTTCGACTTAATTATTGATGAAAACCTTAAAGTACATTTGATGGAAGCTAATATGTCACCCAATTTGTCGTCAGCACATTTTAAACATAATTCTCTGTTATACGAACAAATTTTATATAGTGTAATGAATTTAGTTGGAATTGGTTCACCGCTGCAAAGTAAATTTCTTCATGAAACATCTGATGAAGTAAATGAAATGATAACTTCCGAAAGGAATCTTGCAACTAATATAAATGAATGCTTCGAAAATGGCTGTGATAGATCTTGTAATCGTCA

>BPA_3302

GATAGTGATTCGGATGATGGTTCAAAGTTGATAGTAGATGAGAAGCCTTTAGTGCCCGAAGAGCAACCATTATCATTGCGTGTACGCTGTACTCCACCACCAGAAGATCAAAGACCCTCTCCTCCTCCCTGTCCCGAACCGGCAGTTAGGTGTAGTGTTATTCAGAGGACACCCGCTAACGAAGCAGAAAACCAAAGACGTTCCCACGAAGTTTTATTGCCTTTGGCCA

>BPA_3315

TGTTGCTGCTGTTGTATTTGCTGTTGTTGTTGCATAGCAGCATTAACAAAACTACTTGGAGAAGATGAGGATGACTTAAAGAGATTTGCGTATGTTTTAGGTTCATTCTGCTGTTGTTGCTGTTGTTTAGCAGCTTCATATTTCTCTTGTTGTTGCTGTTCGTTAATTGTTTTGAAATCTTCAACAACGGGAGCTGGAGTTGGTTCCAATACGGCTGTAG

>BPA_3336

CAACCAGAACACATGCAACAACAACAGCAGCAACAGCAACAACAGCAATTTATACCACCACCACAATATCAACAACAACAGCAGCAATTATTAGCCCAACTCCAGCAACAACATAAATTTCAACAACAACAGCAACAACTCTCCCAATCACCACCAACACAACAACAAAACCTTAACAATAGTCGTAACAATTTAGCTTCTCTTG

>BPA_3342

ATATGTATCATCAGACAAACTCTTCGAACATATCTTCTTCGAATACATATTCACAATCGGCCAGTTCCACAAGTTCAACGATGCCCGTCTACGGTAATCGCAGTAACGATAATATCACCTCAATGCGTCAAGATAACAACAATAGTCAATCTGGTAGTGGTGGACCATTGCCTCAACGTTCAAATGCTAAAGGATTTAATAAAGATTTTGATCAG

>BPA_3344

GAGGAGCAAAAGCATTTTCTATAACATCACTAGCATGGGCCAAAAATATCAGTGCCAACATACGTTTATCCATGCGTTGAGGATCATTAACCCATTTCGATAAAACAGAATCTTGTATCTTTCTAACTAAACGACATTTAACCACATTATCACTTAAGGGATGTGTTGTCATATCAAATAATAAGAAATTTTGTTTTTCTGTTGTTAAAACACCCTTTTCAACCAGATTTT

>BPA_3345

CGAGGCAACAGAAAATAAAGCTTGTCTTAATTGCGCAAAATGTTCTTTTGTGCATTTTTGTAATTCGGCAGATAGATTCTCTATGAGAGTACTATAGGCCTGTGAAGTGCTTCGGGTGCGTTCATCATGTACATCTTTTTTTGGAATATTTGAAGATGCCACCGAATTTGGCACGGGCGTGGAAGGTAGCATATCACGAACATGCTCATCGTTAATATTGAAATAAAA

>BPA_3348

TGCAAAACTCGTTGGAACATTAGACGTGGAGCACTGGAAAGATATTTATCCCAGATTTGTTTGGAGGCAGCCTCATTGCCGCGTGAAATGTGATGAATCCATAAAACATTTATGGGACCTAATTGGTTGTGTTTGGCGTAGTCTTCAGCAAGTTTTTCAAATTGACCCTCCAATTCAGGATGTTTATCTAAAATACCCAAAGCTCCTCCACGTGGGAATTTCTCAGCTAGTTTTG

>BPA_3364

TCCATGTCATCTTCATCATCAATTTCTATGATAGGTACCGGTGGCTGTTCTATAGGTATTACATCATCATCTTTAGACTTATTCTGGGGTTCTGTTTCCTTAGGTTCTTCTTCTGATTTCGCTTCTTTAGTTGATTCGGATGTTTCGGAAGTATTTTCTTTAAGATCCACTTCCATATTTGTATCCTCATCGGTTTTATTTATGTC

>BPA_3388

AATCTCTCAGGTTTTGGTTGTTTTATGCAAGAGCATATTAGTGATCCTAAGTCATACGGTATATACATTGTGGATCGCAGATACATTGGCTTAGAAAGTAGTGTTCAACAATTGTCCAATTATATGATGGAATTTTCAAGACTTAACAGAAGGCAAAGAATTATTCAACGTAATCGTACTGAACGTTTAAGTGATTTACTTGATTGGAGAACACTAGGCATTTATTATAGACAGGCTCGTGTAAAAGCTTTGCAAGCTGTTTACCCAGACTACGTT

>BPA_3391

CTTACCCAACTAGTACCAGATCGGCTCAAATTGCAAAAATTGTAGAATATTTCGAACGTAAGCGACAAGTACCTGATAGTACACCTCCACCGCGAGGTGCAAGCGGTGTTAGTGTTGGTGGTCATGTCTCCACTACTGCTAGTGGATCAACCTCATCATCCTCATCTATGGCCGCTGCTGTAGCTGCATCGGCATTAGGATCTTCTTACTTGTCATCGTATGAAACAAGGCGTTATTCAGATTACA

>BPA_3412

TGGTCACAATATCAGTGTAAAATTGAATTCATTAAACCGAAAACATTGAAATCACGTAAACCAATAAATAATTTCACTTTAATGACACGTAGTTCTCAGGAATTATGTGGTAATAATCGCAAAATAAATTTTAAATTTATTAAAATAAAACGTGGCATGGAAGCAATGATGTTGCAACTACAACGCACCATAGAGATAGCAAATCG

>BPA_3417

CTAGTCCTGGAAACCAACCGAAAACAGACATTAGTTCACATCACCATATCTTTGTGGGCGATCTAAGTCCAGAAATTGAAACAGAAAGCTTACGCGAAGCATTTGCTCCATTTGGAGAGATCTCTAACTGCCGCATTGTACGTGATCCGCAAACAATGAAATCAAGAGGTTATGCCTTCGTTTCGTTTGTAAAAAAGGCAGAAGCGGAAAATGCAATACAAGCAATGAACGGC

>BPA_3428

ATGCCCTCTGTTGGTTTGTGCATGTTGAACGCTTACTTGTCCCATCAGGAACACGCTCACCATCCCCGTCCCGAATTCGTTAAATACGAATATTTGGCTAAGCGTGACAAACGTTTCCCCTGGGGTGATGGCACCAAATCTTTGTTCCACAACCCACACACCAACGCCTTGCCTGATGGTTACGAAGCCTAAGGAGTTGTAAACATTCACGTCAAATTGA

>BPA_3454

CACAAAATGATTTGGATATGGAAAATCTTAATGAATTACCAGAATTACGACGTCATCCCATGAGAACTTATCAGTTACAAACTAGACATTGTAGTCTTATAGACTCTCGTCGTCTGGATAAACCGGCTGGTAGTTGTAATGCCATAAACATGACCAGTAGTGGTCAAATTGAAGAACCTCCACCTTTGCCAATGAAGAAAAAACACAT

>BPA_3455

GTTTTGAAATCACAACAATGTATTGAAAAAGATACCGTTACTCTGGCTTGTGAAATTGACGATGCCCAAGGTGAAGTACAATGGTGGCGTAATGATGAAGAAATCAAACCAGACAAACGTATACAAATTGTCAAAGATGGTCGTAAACGTAAGCTTATTATCAAGGACTGCAAAGTCACAGACGCTGGTATGTTTAAATGTACAACTAATGCCGAC

>BPA_3464

GGAAAATATTAATGCCCTATTGAGATATCTGCATGAGCTTTTGCAGAGTTTGGGTTACCGTGGTAGCAAGCACCAACAACAGCCACCATGGGATGATGATATACAAACGGATCTAGAAGATTTAATGGAAGATTTAGCCAACGATCAGCAAGGTGATGAAGATTCATTTGTAGAGGATTCCGATGAAGATAGTCTCAATAATAAACTTTGCACCTTTTCATTAACCCAAAAAGAGTTTATGAATCAACATTGGTATCATTGTCATACCTGCAATATGGTTAATACCGTCGGAGTATGCTCTGTATGTGCACGAGTATGTCACAAAGGTCATGATGTTAGCTATGCCAAATATGGTAATTTCTTTTGTGATTGTGGTGCCAAAGAAGATAATTCTTGTCAGGCATTACGCCGTCGTCTAGCTACCGGACTTTCAGGCTTAGAAAGTAGACAACAAAATGAATACTCTCAATCTTCAACTAGCTACAACAATTCTTCAAATCTATCACAATCGAAACGTTCC

>BPA_3476

TAAGCGAAGTGGTGCGCACACGTAAGGAAGGTGAGGTGTTGTAGGGCATATCCATGATGCTGGTAGCTGTCACAGATCTTACTAAAGTCGAATGTGGGATGTGCTGGACGACAGCGGCGTTAGCTGTGGAGGAAAGTAGTAGGGAATTTAAGAGGGTCAGTTGGGTTTGTGATTCTTGATTTTTGAGTTTTTGTTTAATTTGCTCAAAAGTTGGATAAGAACAACAGGAATTGGTACAGGTTTCCTCTGCCTCTACTCTGGGAGAGGTAGACAAGCTACCACTACCACATGAGTTCTG

>BPA_3478

TGTGCTACTGTTCGATGATTCCACAGTACAATATTTTGGTTTGTCCCACTTGCCCGGTTTGTTGACTCTGCTGCTAGAGCATTTCCAAAGGAATCTCGCTGACATGTTCAACGATGAAGACAACAATGAGAAACACCTAAAAAATTCTGTTAAAACAGCTAAAAAAGTCAATGACAAACGCACCTGTGCAAAACTTAGACCCTCATCGCGACAACATGACACAAGTTCGGCTTC

>BPA_3492

TATTGTGTTATTCGATACTTTGCTCCTAAACAAAGGTAAAAAAGATGATGCCGATATTAAAGAAGAAGACAAGGGCAAAGGTTGTACCAATGAAGAAGTATTGGCTGTTTTGGGCCATGAATTGGGTCATTGGAAATTGGGTCATGTTACCAAAAATATAATTATTATGCAAGTAAATTTGTTACTACTCTTCTTCGTTTTTAACTACTGCTTCAAATATGCTCCCTTTTATGAAGCTGTTGGTTTTGCTCCTGGTGTGCGACCTATTTTAGTTGGACTTTTCATTGTACT

>BPA_3493

TGAACTTATATCGAGTCCAGCATCACAAGGCAATATATCGAACTATAACGAATGTACCACATCAGCCACTGAATGGTTGGGTATAACAACAAACAGCGATGACTGCAGTTATAGTTCAGAATATGATAATTCAGACAATTATAAGAGTAATCAATATTCAGATGATAATGGTTATGATTTGGATTTCACACCAACCACCATTCTAAATGCCCATGGAACCAGTGATAGAATAAGTTGTA

>BPA_3509

CGCCACAACCGCCAATGTAACCAACTAAGGCGAGAGTCGAAAAGAAGGTTGTTTCAAGCATCACCATCGGCGTATAGGGATGATGTATGCGAATGATGCATGTTGTCTGTTTTACTGAGGAGAAAAATGATTTCGGCACCCAGTGTACATCGATATCGATGGCAGGACGTAAAAGTGTAGACCATAGCAATAGTTGAAGATA

>BPA_3522

ATGAACATTGTCAACTTTCAAAGGACCATTAGGTGGTGAAGGTTTATCAATTACGGTTACTTTGACATCAGCGCTATCTTCACCGCTCATGTTTTCAGCATGTACAGTATATACTCCAGAATCAGCACGAGTTGCATTAGTTACTTTCAGTTTTGTGTTATAATCAACGTGTGTTACTTTAACGTTATCTTTTGTATAAACTTCGCGCTTTTTGAGCAACCATTTTGTTTTTGGGGCTGGTTCACCAGAAACTTTAC

>BPA_3524

AAACAATTGAAACTTAAACAACAGAGTTCGTTACAAAATAACATTAGTAAAATTGCATTAACAGCAGCAACCGAAGGTAATTCTAATGCGGAATCATATGAAAGCGATTCTAAAAACTTTATTCGTGTGAATAGTGTATATCAAGGAGATTTTTCTCAAGAGGCTAATACACCAAAATCTTCACCCACAACGCCTTATGTATGCCAAAAATCGCATGGACATCAAAGTCATCCAAATTCCCAACAACTGCGACAAACTTCTTCTTTG

>BPA_3533

CTAGTATTTGTCAATATATGCCTTCAGCATGTACGTGTATTTTTTATGTGCAAGAAACACGACAGATATATGTTGGCCAAGAAAATGGTACAATTACTGAAAATACATTATCAGAGGATTGTAATCGCCTATCCTTTGTACGAGACTTTTTAGCTCATCAAGCTCGCGTTGTGGCCGTAGTATTTTCTAAAGCTCATAATTGGATTTTATCTTGTAGTAAGGACAAGCTTTTTGCTTATCATTGCACTGAAACTGCGCGTCGATTGGGAACCTACAATTTTG

>BPA_3539

GCCTGAAGCAGAACCGGAACGTGATCTTGATCGTGATTGAGACTTGGCATGAGATTTAGATCTTGAACGCGATTTACGTTCTCGTTGGGTCTGTTCCTCATCCTTACTACCGTCCTCTTGTTCTGATTGCTGTTGTTGTTGCACTGTTTGTGGAACATCATCTTCTTCTTCCTCCTCAATTTCCATTTCTTCTTCATCATCCTCATCACCCGGAGCTTCC

>BPA_3554

ATCATTAAAAATACTCAAGAGTTTATCTTCATCATCCGATTGACATTGGACAGGATTTGGTTCTGGACCATAGTAAATACCAAAACCTTTTTTGCGTGGTTCTTCGGAATCACTAGTGGAATCTGATCCGGGATTATGAGTTTTAAGATCTCGTGACTGTTTGCGCTTGCGTCTTCTTTTCTTAACATGACGCCAAATCTGAGGCAA

>BPA_3563

TTATTATGCAGATCATAACCTCACCAAATGCTGATAACAAACAAAAAGATGGTGCATTACATATGATTGGTACATTGGCCGATGTATTATTGAAAAAACGTATATATCGTGATCAAGTTGAAACAATGCTTACTACCTATGTATTCCCTGAATTCCAAAATCCAGCTGGTCATATGCGTGCCCGTGCCTGTTGGGTATTGCACTATTTCTGTGACATCCAATTGAA

>BPA_3583

GCTAAAATCTTCTCCAGCACGACAAACAATATCACGTACACCACCCAAATCCATACAAGGTTTATTTGGTTGTTCCTCAATAACAATCGGTTGTGATGGTTTGGAAGGATCAGAACGACCGACTTCATTCACGGCGGCAACACGGAAAGTATATTCTTCGCCTTCCTTCAAGTTTGGTATGGTATACACTGTATTGTTAATAGGTAATTCG

>BPA_3595

CAAACGAAGAAAAACTTTAGCCAGACTGTAGAGAGGAAGAATATTGTGTGACTGACACAAAGGCATCTTAACAAGTGTTTAAGATAAAAATTAAATTAAAAACCTTTTGCTCCAAAAGGTGAAAATCGTAGGAGAAGATAAAGCGTTGCACAGCTCTTGAAATTGCCCTGTTTGGTTAGAAAATTTGCAAAAATTTCACCC

>BPA_3596

GTGCAAAGCTTGTACAAGAGAGCTTTGCGTAACTTGGAAGCTTGGTATGATCGCAGAGAAGTTTACCGCTACAAAGCTGTCCAAATGCGTCAACGTTTCGAGGAGAATCGCAAGGTAGCTGATATGGCTAAGGCCGCACATTTGTTGGCCGCTGGCGAGCAAGAATTGTTCAATACCCAACATTATCAGCCCGTTAAACAGGCTAACAGTCCTGGTGGCTGTGCTTTTGAGCGTGATGTGGAACCCCCAGATTGGGTTTTGGATTACTGGCATCCTTTGGAGAAAGCTCAATATCCCGAATTCTTTGCTAAACGCGAACAACGTAAGAAGGAATTTGTGGCCTGGTGGGAGAA

>BPA_3599

AACGAGTGTGCAATCTATAGTACTGAACATTCCTCCCCATACGGCAAAATTGCCCGCAATTACCGGAGATCTAATTTTAATTGCCGATAAGCTTCCAATTATTCTTTTATTCATTCCAGATGGAGCATTTCGAAAACCTTTGATAGCTTGGAATATTCCCCCTCCAATGCAACCCATTGCAAACGCACCACCACAATCATCAACAATTCTGAACG

>BPA_3600

CATCATCAGCATCAATACGCTCATTCGCATATTCATCATCAATATCAACAGGCGGCTGGTGAACATCATGGTATGCCTCCACCACCACATCATTATGGTGGCTCGGGACCACCTCCACCACCATCACATTATGGTCCTCCACCACCAACCGCTCATTGTGATGCTGGTTCGCCACCACCTACTTATCGTGCCAGTAATACATATGGTTCT

>BPA_3612

ATATTTTTTCTCCTTTTTTTTCGTCTTCGATTTTCTCTTCTCTTTACGTTTTTAGATAAAGATTTAACACGCCAAACTAAAATCAGTTCCTCAGTGGAAAATCAAGAAACATTGTTAATGCGTTCCGGCTGGTCTGGCAGTAAAAAATAAGAAATTCATAGAATGAATGTTATTGAAAAAAACCAGAACGCTAAGGGAATCGCTTTTATGATATTTCAACAATATCGTAATACAAGACATAAAGAGAGAGCACGCA

>BPA_3616

CGGGCCATGCCTGAGTTATTATATTCCAACATTTGATTATTTATATAAGTTCTCAAATTATCATAATTTAGATCCTCATAGAATCCCTGGGCACTCATGAAATCCCCAAATATGGGTATCACTTTATTGGGACATAAATTACTTAAAGTTAATTCCATATTTTTACCCAAGACTTCATTCATTTTATTTTGAAACCATAACTGATCATTATCATCTACCAAACGAT

>BPA_3622

TGCCAGAGGAGGTGCTGGACGGCCATATTGCGAATTGGCTGAATTTTGCGCTGGTAAACTTCGTCGTTGCGCCTGAGTTAAAGGCACTTGAGTGGCGGGTTGAGCACTTGCTGTTTGTCCAGGAGCCTCATCCTCATCGGTCAAATCAATAACGGCCTTTTCTTTTGGTTTGGCGGGTGTTATACTTACAGCAGTATTACCTCCGGTAAGATGTGTTAATGTATTGA

>BPA_3631

TTATCACCCATATTTTCGGTCTTTGGTGTTGCTATTCTATCCATAACATTATTGAAAGATTGTAAAGTACTGCCACCATTTGTTAAACTTAATCTATGACTAGGACTTAATTTATTTATATTATTACTACTGTTGTCATTATCACGTGGTGGTGTTATTCTCTTTGTTGGCGATAACTGTTGTTTCAGCGATGTTGTACCACCATTAATCTCTTCTAACTCTAACGTAT

>BPA_3645

CGCGTACCTTCTAGGTTCCATGATAATGTTATAACATGCGAATGACTCACAAATGTTCCAGTTTGAACCCATCTGTAATCCAAAAAATGTGTATATCTTTTATCCGTTTGTAATACTGGTGTTGGTTCGAATATATAAACCTTGTTTTCATAAGCTGCAGCAATCTTTCCGGTGTCTGTGCTGCAATCAAGGGCTGAAATTTTGACATATCCATGTCTTGCTCCTGGTAT

>BPA_3652

CGGGAGCGCTTGCAAATGTTTTGGGCCTTGGACGACCAGGGGCTGACCTTCCATTGGGCCTTCAAGTCACTGTCTTGCCAGGCCTTGCGCACAATGCGGGTGGGAGCAGTGTAGGGGAACTTGATGCGGTATTTGGTCAAGTGAAGATTGTTCAATCTGTATTCTTGACGGGGTACACCAGTCAAGGGACCATCAACAAGAACCCTGTTTTGATCAATAACGTCGACAATGGCGACGAGACGGCCCTTCAAGGGAC

>BPA_3665

ATCATCAGCTCAGCCCATGCAACATAGTATGGTATCTCCTTTGACCAAAAAAACCCATCACAGATCACAGTCAGATGCCACGGTACTCTTAGCCGATTCCAGACGTGCTAGCTCTAATCTGAGCATATCAAATCGTTTGTATCAACAACCCCCCTATACAACACAAACCTTGCCTACCACACCCGCCAAATTATGTCTCGGAGATTTGACTTCTGGTCGTGCTTTCGACAATGGTTGCAC

>BPA_3677

CAAGTAGATTTGTATTTGGTGTTAAATAGGCAACAGTAGCAGTTTGATTATTAAGTATTTGAGGCTGAAGATGTTGTTGCTGGGGTGGAGGCAATGTTATAGGCTGATGAGGTTGAGACGCTACGGTTTGTTGCTGTTGAAGCTGTTGCTGCATTGACAATCCTTGACTTTTAAGTAGATTCATAGCATATTCATTAGCTTCTTTACTTTGGAATCCC

>BPA_3685

TCCAGCTATACTATCAATTTCACTTTGACCTAAAGGAAAACCGACTAATAATTCACCGGTCTTAGTCCACCAATCAATCATATGTTTTATTTTCTCTTGCGTTGGAATATGAGGATCAATTTCGATCGGTCGATATTTATGATACAATTTTTTCGATTCCTCAATAAAACTTGGTGGCAAAGATTTACAAGCATTAAAAATTCCAAAACTACTCAGTACCGGTTTTCCATCGG

>BPA_3692

ACAATACGTTGTGCCGCAGTTAGTTTAGCTTCTTTGGTCATGGGATTATTACACAAATCAATAACTTTGGCATTTTTGAAAGCATTATCACTACACCAAGTCTGGCACCATAACCATTCATCGGGCAGAGATTTAATGGCCACCTGATGTATCATATTATTGGGTAAATCTTGATCTAAATTCGATAAACTGTTAGGATCTTGGCTAAGAGCC

>BPA_3710

ATTATACTCGCTACAAATTCAATAGATGAAGCTGCCGATGTTGGTGATCGTATTGCAATTTTAAATGAGGGCTCTCTAATCGCTTGGGGATCGTCATATTTTTTAAGACATAAATTTGGCAATGGTTTATACTTAATATGCTTCCTACAACCCATGACACCCATTACAGGCATAACGAAAATTCTAGAAAATTATGTCACAAATTTTAACGTTTATCAACATTTCTC

>BPA_3717

AACAACAGCAGCAGCAACAACAACAACAAAATGGTTATCACAATCAACAGTACAATTCTTTGCAACAACAACAGCAACAACATAATCATTTAAATCATCATCAATTGCAACAACATCAGGCTCATCACCAACAGCAGCAGCAACATCATCATCACCAGCAACAACAACAGCAACACCATCAACATTTACATCATCATCACCATCATATGCATCATCACCCACAACACAGTGATAATAGTAATTTATCACTACCCT

>BPA_3720

TTACAGAACAAGGTTTACGTGAACAACTGTTGGCTACTGTTGTGGCTCATGAACGTCCCGATTTACAAGAGAAAAAGGAACAATTGATTATTGAATCGGCAAAAAATCGCGATGCTTTATATACAATCGAATCAAAAATATTGGAGGTACTCTCAACGTCCGAAGGCAATGTATTGGAAGATGAAAATGCCATTAACATTTTGTCATCAAGCAAAATCTTATCCGAAC

>BPA_3721

GAAATGCAAAAAGCAATTCCTTCATTTCAAATATAGAAGTAAACAAAAACCAACAGAATTCATCATAAACTTAAATAAATATAATGAAAATATGAAATTCAATACAATAGAAATCAGCAGATTGTGCTAACGTGTAAATTCTTCAATGTTATCCGATACTGCATTTTATTTCCTGTTTCTTATCTCAAATTGCCACAATCCAAATTGCCCACATTTTGTGGATGTTGCATTTATAAATCCAGCAAACGTTGT

>BPA_3723

CCACATTGAGAGGACGTAAACTTCAGGTGGATTTCGCTTCCAGAGAATGTCAAGATGCGTTTTATGAAAAATTGGAAAAACAATCTTCAGGCACTCGCTTTAATCGATATGAATCACAGTCAAGATCTCGAGCATCATCTTTTAGTCGACATCAAAATTCGAATGATGGCTGTTCTCCCGGTAATACGCCGGGTAGCAGCAGTAGTAGCAGTGTTGGTATGGGCTCATCAGCACCGGCACTATCTTCTGCCATAGTGGC

>BPA_3729

GCTAAATCAAGATATTCATGTGGTTCATCTAAAGCCGGCACTAAATTAGATTTTTGTGCCTCATTATTACGTTCCATAGTTTCGGCAGTATTATTCTCTTCGTTTGATTCAGGTTGTAGAGTTTCTTTGAACCAATGACCTAAATAACTCTCACTATCATCCTCTTCAGAATTATCCGAACAAGACAAAGTATCATTGAAATATGTTGTAGAATCAGAATAACTAATAGGTTCACATTCGGTACGATCTTTAGCATTAGATCGCTTCAAGTTA

>BPA_3762

CTACGATCAAAAGTTTCACATCAAGATAAAATAAGTGAACAATTTAAATTCAACATACAATTGGACGGTGTGGTACTCAATCTAATGACAGATGTGAATGAAGGTCTGGCACGCTTTGGTTTGTATGTTATATCGTTGAAGGGCACAAAACTAATGGATGAGACATTGACGACAAATATTGTTCTCTGTAATATGCAATTGGATGACAC

>BPA_3768

GTGATAATGCTGTAGTACCTTTGCCAAATGTTAATTCAGCTATTTTACGTAAAGTCTTGCACTGGGCCAACTATCACAAAGACGATCCCCAACCACCAGAGGACGATGAAAACAAAGAAAAACGTACCGATGATATTTCATCATGGGATGCTGATTTTCTTAAAGTTGATCAGGGCACACTATTTGAACTGATATTAGCTGCCAATTATTTGGATATCAAAGGCCTATTAGATGTTACCTGCAAAACTGTGGCAAATATGATTAAGGGAAAAACCCCTGAAGAGATACGTAAGACATTTAATATTAAAAATGATTTTACTCCCGCTGAGGAGGAACAGGTGCGCAAAGAAAACGAATGGTGTGAAGAGAAGTAAGGCAACAACAATTTAAATGCAACGAGACAAAGACCAAATTTTAATTACGCAAAATTAAAATGACTTCAA

>BPA_3773

TAAATATTGATATTGCTGGTCCCACAGTGCGTAGTATTGAACGACAAGCTGAAACTACCGGCTGGGCATTAGACAAATTTTTAGATTATGAGACTTTACCCGAAACAAAGGTGCCATGTTACACATACGATGAAATGGTAAAGTTGGTATTAGATGCATATGACGGCTCTGTCGATGAGAGTTCCGTTAGGGTGTTGATGGAACGTGGTATGGAAACGGCACCACAGCATCTACACAAAAATGGCTTTCATTTTGCCAGAGACTTGCGCTTAAAAATC

>BPA_3774

AATTAAATGTGGTAGAAACAAATAATGGTAGTAGTAGCATTCATTTGAACACGGTGAGCACAGTCATTAAAAAGAATGAAACTATAACGTCTACTGATTCAACAGTAACACCTACATCTACATTAAGTACATCTGTAGCTGCCATCCCGAAAGAAAGACAACCAGAAAATAACAAACCTTCTGCGATACAAAATAAACCG

>BPA_3783

GAATTGTTTGATACAAATACGCAACGTTTCTATTACTATAATGCTGCCTCACAGAAGACCGTCTGGCATCGGCCCAGTAAATGTGACATCATACCGTTGGCTAAATTGCAAACGCTTAAACAAAATACTGATCCCAGTGATCGTCGTGAGATTAGTACACAAACATTACAGCAACAACAACAACAGCAGCAGCAGCAACAA

>BPA_3820

GAAAAACCCAAGGCAGCTGATAAACAAGAGAATAAAGCTGTTAATAACAAAAAGGAAGAGGAAGCCTCTGATGGTGCCAAAACCATAGCGGAATTAATGAAAACTAAAGTTCACTTCCATGCTCCTGGCGAGAACTACAAAACAGATGGCTATGTGGTTACCGAAAACACCGAACGTTTGCTTAAGGAACATTTGAAGGCCAC

>BPA_3825

TAAATCATCTTTAAGTTTAAGCTTTTTGTCTTCTAATTTTTCTAAGAATTTTTCAGCGGTATCTTCTTCGGTGAAACGTACATGTCCTACTTTTTCACCCTTACTGAAATCAATGTAGGCAATATCAAAATCTGTTGATACTTTCTCAACGGCTTCGCGTATAACTTCTCGGGTAACATCATCATTTGCACCTTCAAAGTACAAAAC

>BPA_3842

GAATAACTTCATTTTGTGAATGATCATAATTATAAGGATTTAACTGAGATTTTGATTTTTTATAAAGTTCCTTATGAGGATTTTGTGTTATATTGATGGTTTTATAAAGTTCTTCCTCATTGGAATGGGTTTGTTCCAATTTGACTATGTCAATATCATCTAATTGTTGTTGGAAATTGAGATTATCATAATTTGGTTGAAA

>BPA_3845

CAATGGTCTGCAGATTAAAGGTCAAGCTATGACTTATATTCAAGCGAATTTTTGTAATTCAATGGCTGATATGGATTTATTCTTTCTACAAATTTGTGCCACAAATTTACCACAATATTTCTTTTTACAAAATGCTATTGAACTATTTGGTGTAACACAATGGCTGGAGACGGCACCACTTAAGCAACCTCAGAAAATGGAACAGACTTCAATGCTTGAGGGTTTCTTAACA

>BPA_3855

TTTTACTCTTCCTCTTCTATTACTATTTTTTTCAATTGTTGCGGTTTAATTAAAAATCATTCACGCTGGATTTGGGATACTACGAAGCTGTTAAAGCATCACAATATGAACCGGGCGGTTATTCATTACAGACACAAACCAATCGTAATTCAGCCAACATGACACAACGTGATGGAATAATGAAATTTGAAAATGAACGAATTAAAACCCTGCAAGAGGAACGTTTACACATACAAAAGAAAACATTTACAAAATGGATGAATTCATTTCTTGTAA

>BPA_3862

AAATATGATACAGAACCAGGCAAATGGATACGTAATTATACGGGCATTAATTCAGTCACAAAACAACCATTTACCGTTGATGTCGGCTATGAAAGATTTTTAGGTCCAGAAATCTTCTTTCATCCAGAATTTTCAAATCCTGACTTTACCATACCTTTGTCGGAAATCGTTGATAATGTTATACAAAATTGTCCAATTGATGTGAGACGAC

>BPA_3876

TGAAGATTGAGCTGGCAGTGAAGCTCGAGAGGGCCAAGAGAATTTTGCAGATGAGGAAGTGCTACCGCCACTGTTCGGCTCTAAGGATGTGGCATTGAAATCTATGGCATCGTCATCATCCATATTGCCCGCTATGCTGCTATCGCCCACAAAACTTTCCGGATTAATGCCCGAACATTTGTTGGTATTACGGTCCAAATGTTTG

>BPA_3881

TTGTTCAACGTGCCAGTGTGGGAGCTAAAAATGCTCAAAATAATCCTGCTGCAGCTGCACCTGTTACCATACAAGTGCCAGGTCTACCAATGTTAGGTATTTCTGGTCCGCCCACAGAAGTTTTATGTCTTCTCAATATGGTTACACCCGATGAATTGCGCGATGAAGATGAATACGAGGATATTTTGGAAGATATCAAAG

>BPA_3892

TTAGTTCTGGGTTGTGCTGTCAATTGTTCCACCAAACAAGATTATATACGCAAGATTATGTGTTTGGAGGAATCATTGCAGGCAAATATAATGAGGGCCTTACAAGAACTAGAGACGAGTTGGCAAGGAACATCAATAACTAGAAATTCACTTAGTATTGCTAGTTTCGATTATAAAATGCTACAGGAGGAACGTGATCAAGTG

>BPA_3898

CTTTTCCCGGTTTTGGGTCAACTATATTCGATAATTGATTAAGCAGCAATGTTGTTAGAGAATCTGGTTGAGAGACTTGCTTTTCGTCCAGGAAATTACTGATATCGGCAGCAGTTTGTAACAGCATTTGACAAGGACCCAAAGCACTTTGAGATCTATCACGTACTACTAAAACCAAATGCAAAGCACTCGCCCTCAAACAGATTTGTTTAACCAGTAACATATCAC

>BPA_3901

TTGTAAATAAACATATCCTCCACTATCAGAACCGGAAGTGTTGGAACCTGGTATACTAGCAATACCATACTGAGCTGATCCACTGCGTTCTATTGGTTTTTGAGGAAAATTTTCTTCCGGTAAACGCTTCAATTCAAACATATCCAACTCCAAACGAAGATTAGAAACTTCACACAACAAGCGATCACGATCCATTGTAATTTCTTTAAGTTTTTCCCGCAATGTCTGGTTTTCTATTAAAAGCTCCTGCACATTAATTTGCATAATTTTAAAATCAGATGTTGGAATTTT

>BPA_3924

CGCGAATGGACGATGCTGTTGAACCTGTTGTTGACGTTGACGCTGTTGTTGCGTAAGAATTCCAGTATCAGCGGTGGTTGATTGTATTTTTGTATCATTTGTGTGTGGCTCAACAGCAATTTGTTGTTGCTGCTGGTGTTGTATTTGATGATGTTGTTGTTGTTGTTGTTGCTGCTGCTGATGTTGAGTATGTTGCAGAACAGTGGGTGAATGTTGTGAATTATTATTATTATTACTGTTTGAATTACAAATATTAATACCCGTTTGTTTGATTTCTAAACCGGGAGGTAAATTAAATATAAGATTATCACTCATGACAACATTTCCACCAGTTGTTCCAATAATACCAGCAGC

>BPA_3930

ACCAAGTTGTGTAAACACATTTAATCGAAGAGAGCGAAACCCATATCATCATCTTCAGATTCGGATTCTTCCTTCTTCTTTTCTTCTTTCTTGGCTTCTTCCTTTTTGGCACCGGCATCACCACCAGCGGCGGGAGCAGCAACAGCGGCAGCAGCACCACCAACGGGCATAGAAGACAATTTCTCACGACCGGTGGCAATCAATTCTTCAATAGATTTGCCAGTCAATTCGGTGACAACTTTGGTCAAGCGTTCGCTGTCAGCTTCAATACCAACAGAGCTGAGGATCTTTTCGATATCAGCATTTTTGGGGTTTTCCAAACCGCCGAGAACAGCCAACATGTATGCAGCCACGTAACGCATTTTTTAAGCCTTAGTTTTGACACGAAAC

>BPA_3946

CGTCCGGCAGGCGTTCGACTTAAATTAGGACGTTGGACATTTGTTGCATTAATACTGGGACTACGAAACCCCGATGTAGGCACTCTAGATGGTATACCCTTTAGGGGTGTGGTGTCTGTCATCTTACGCGGCATACCACGAGATCTTAGGGGTATGGTAGGAGCTGAAGCCTTTTTAAGTGATGATTGCGCATCAGCCGATTTATGTAGAAGTTCTG

>BPA_3958

AAAGAAATTTGAGCTAGAAGATCGTGTTAAACTACTAGAAGAGGAAGTTGCCCAATTAGAAGCTTTGGAAGAGGTGCATGAACAACTTATAGAAAGTAATCATGATTTGGAAATGGATTTGAGGGAAGAATTAGATTTGGCCCAAGCAGCTAAAAAAGAGGTGTTGAGGGAAAGAGATGCTGCCATAGAAACCATTTATGATAGAGATCAAACTATAACCAAATTTAGAGAAT

>BPA_3972

CCATGAATAGTATGTATCATGCTGAGAAGGATTTTCATTTAATGTATCTTTCGACCGATGCAACGAGTAATGTCAAGGAAGAGCATTTACAAGAAGTGGAATTAACACAACCACCACATCCTTTTTTTAAATATTCAAAAGCTCAAAGTGTGTCGGCCTTATGTTGTGGTTCTCTATTTCTATTGCATGCAATTTTGGCATTCGATC

>BPA_3978

TCGATTTTTGCAAGACGGCTTAATAAATTTTGCCCCATTTTGTACAATTTCGGCTGAAAATGATACAGCACAATCAGCTGATATTCTTATTACGAATCCAGTGGCATCACATCGTCGACCACGAACGCCCGCTTGGAGCTATATGTTTTACCCGAATGAATCGCATGCAGATCTCACTATCATTTTACCAACAGCTATTTTGTTACGAGAGGTGCAAATTCAACCTCATGGTCCCACTTTAGCATCATGCCC

>BPA_4026

CGACGAGGACTCTAGAACTTTTTCTATTGGTTGCGCTTCAGGTGATACCATTAAATTAAGAGCAGCCGATGCTCGTGCCCGGCAGGAATGGGTGGATGGTTTAAGAGCAGTAGTTGAGAGTCATACCAAAGCCATGGATATAAATAATTCAACACCCTTGCCCCCTCGTGAATTATTAGCAGCATCAGATGCTATGGTTTCTGCTAGACAGGCACTTTATTTAACCGAACAATGTAATGCTTCATTGGCC

>BPA_4031

CAGAAAGTGTTCATTTGAAAATGGAAGATAAATTGGTTGTACGCATTGGTCGTGATGGTGGATTGCAACAATTTGAGTTATCCGGTTTGTTAACATTACGCATTACCGATGAAAATTTGGGTCGTATTAAAGTGCAATTGGAAAATAATGATACCCATGGCATACAATTACAAACACATCCCAATGTTGATAAGGAATTATTTAAAACACGCTCAATAATTGGTTTGAAAAATCCAGCAAAA

>BPA_4034

GGTGAGCTCCAATTAGTTTTGCTCTTTATGCTCCTGGAGATAATGACTTTGCTACCATGGAATCTATACAATATGTACAAAATTGTTTGGCTAAAAGTCTTCTCATATTTACTTGCCCCAAGATCATCTACCAGCATTTGTAGTTACAACTGAGGAAGGAACTGAGGAGAATGCCCTTATGACTACAACAACTTTATAGTCCCCTATGAGAACGTTAAACGTTCGAAAATGTATAGAACTCTTCAAAAATTTGATTATCCAATAAA

>BPA_4054

CCCAATCCCAATACTAAAGAACCCAATTCCTGAAGAGCACATACCAAGAAATGTTGGCTAAAAAGAGTTTCTTGATTTGAATCCTTTGCATTCTCTGGATTAAAGTCTATGGAATTCATTTGTTTGGCAATCATATAAACTAGTTCTTTGCAGGCAGATGTTTGGGCCTTTTCACCAAGCATTTTGCCCAAAACAGATCTCAG

>BPA_4064

GGAAAAGGGTCAACTTCTTGGATATTATAAAAAGCTTTCTAAATTTAGGTTGACATCACTTGTGGTTATTACTGCTATGGGAGGTTATGTTATGTCCCCGGCACCTTTTGATTCCGCATCGTTTCTTTTATGTTCATTGGGTACTGGTTTAGTTTCGGCAGCTGCAAATTCAATAAATCAATACCATGAAGTTCCCTTCGATTCACAGATGTCGCGAACAAAAAGTCGTGTTCTTGTCACTGGAAAACTGACACCGTTGCAT

>BPA_4073

TTATTTAAGTTGGTTGAATTATTGTTATGACAAACGTAAAGAGTCTCAAAATCCTACACAAAATCCCACGCCTACTTCTGCCCAAGGGGTAACCACACAACAACCGCAACCTACAGCAGCAGCTTCCACCAGCACATTGGCCAGTACACCTAATTTGTCTTCGGTCCAACAAAAGTCTCAATCTCAGCAGCAGTTGAAAATAAGAAATCAAAAAAGTTCTACCATGACGGGCAGCA

>BPA_4077

GCTGGATATAAAATAGACAAACGCAATTTCTTATTGTTGTCTACTATAAATACAGCTCGACAGGTGAGTGGTATTCCTTCAGCATTTAACTCATCTTTATCCAACATATTAAACATTAAAGCCAATTCACGTTTATCATCAGCAATTATAGGATAATCAAATGTCGCTAACTTGCCATAACTTTTGATATCCTCAATCCAACCTAAATGCGAATCAAC

>BPA_4078

TAAATGTGTTGCAAACATTTCATCCAGCTGATAAAGATTATGGTCATATGAAAATAGACGAACCCAAAACTCCATATAGTTTAGCAGAACCAGACCCACAAAGGGATCAATTAGATGCTGAATTATTAGCTGAAAAATTACGCATTGCTGCCAATACACAAACGCCTTCATTCGATGATGGAGAAGAGTCTGATGAAGAATTTGAAGAGA

>BPA_4095

TAAGGAACGCACAAATGAATTGCTAAAATATGGTCAACAATTTCGTTTGGAATGTGTGGAAAATGATGGAGATTCTTTGTTGTTGTATAGTGCACAAAAATCACCAGATTTAAATAGTATGATTACATCAACATTTAGTTCCAGAAAACATGGTGAACTTAATTTAAAATTGGGTTTATGTTGGAAGAGGAATTGTGGTCCTGGTAAGTTAATACCCTCGGC

>BPA_4118

AGCATGTACAAAGTTCATGGTACCATCGACGGGATCAATAATCCAGGTGGGAGCATCGGTTAACTTTTTGGGTGCACCCTGGGCACTACTCTCTTCCTCGCCTATGAATTTGTGATCTGGAAATTCTTCACGAATTCCATTCATCAACAACTGTTCAACTTCCTTGTCGGTTTCGGTAACTAAATCGATATCGCCCTGTTTACAAACAAATTCCTGGCGCGTCTCATTTCGTCGGGC

>BPA_4155

CTGAAAATCCATAGGTGGTGGCGGTGGTTTTTTACTTTTTAATTTTCTTTTTTCAGCTTCGCGTTTTTTACGTTCCTCCTCTTCTTGCCGTTTTTCGGCTTCAGGATCATATAATGGTTTGGATGATGTGTGATAGGAACGTGCAACAGCTGCTCCACCTGAACTTGATGAGGAATCCTTCACATGACTAACACGATGTCTTGTATGAGAATTTTTCTCTTCAATT

>BPA_4170

CCACGGCAAATAGCCATAGAACCACCTCCAGCTCATAGATCAGGTGCTGTTCATAGTGTGGTAGCACCACCACCTCCTACGGTTGTTGTTCCACATCCTACAGCTGGCAGTAGTAGTCCATTTATGCCAGCTGCACCTCCTGCTACTGCAGTTCCGGCAGCAGCTTCTTCTGCACCGCCTCCTGGCCATCATACCAAAGTAATGGACCGTGAAAGAGAACAACGGG

>BPA_4172

AATTATTTCACAACGAAATACATGTTTTCCATTAGTTCCAAAGGACTATCCCGTACAAATTGTCAACGAACAAAAATACCCAGATTGTTTAGTGTTAGAAGGAAATTTTCTTACGCCTTTAGAGCTACATTTGCCAGGCATAGTACCAAAAGCAGCTCAAAGTGCACATTTTCAGGTTGTATTACCGAATAATTGGAAGGATCGCCA

>BPA_4187

TCTTAAGATGGGAGCAGTAGGTGGAACTGGTAGCTGTGGTATTGGTTGAGATACAATTGTTGGTATAGGCTTAGGCTCTTCTGTGATTGTGGGTGTTGTAGGTGTCGTCGTATTCGTGGTATTTATTGCAGATATTGAGTTAATTGGCTGAGAGCTTGTAGAGCTCAACGTGGTCGCTGTTATGGTACTTGAATTGTTTGAGCCGGAGATCTTTTTATCTTGTTGTTGCTGTTTCTC

>BPA_4192

CTATCTGGCCAATTTTCAGATGCTTTGGGTGAACTTTTGGAATGGTTAAAGAAAGCTAAACACCGCCTCAATGAAGATGGGCCTGTACATGGAGATTTAGAAACTGTTCAAGGACTCTGTGAACAACACAAGCATATCGAACAAGATTTGCAGAAACGTGCTGCCCAGATGCAAGGTGTTCTTAAAACTGGTCGTGAATTGGA

>BPA_4195

CTGGTATTAACTGGAGCAGCGATATTTCCTGCTGCAGATGCTTTTTTACCACCTCTCGGTTGACGTCCCCGTTTGGCTCCTGTTCCTCTGGTACTAAGTCTTCCTTGTACCGGTACTTGTTCAAATGCTGGCATTTGTTGGGTTTCTGTTTCATCTTCGGTATCATGATCTGTTGTAACATTTCCAGCAGATTGTGAATATGTAGATG

>BPA_4223

AAAAGTGAAACCTTTAAAAATGATGAAACGTACTCGCGAAGAAGTACAATATGGAGCACGTCCAGGTCCTGGTGGCGTGGGCGGTAGTGGGGGAGGAGGAGGTGGTGGTGTTCCCACAACGGCTGGTGTTGTAACGGGTACAGCAAATGTCACAACAGGCGCTTTAAATATCGGCACTGTTGTACCCGGTGCACATGCAACAGCC

>BPA_4230

ATACAGGTGTAAAAGAAATTATATATTAAAAAATAGTCAACATACACACACACGCACATACGCAAACGAAAGACGATATTTGAATCGCAAAATGACTGAAAACGTTCAGTGCCCAGTATGCACGTTATACCTGCATGCAGGTATGAATCTTTCGGATCACTTGGAGACACATCCGAAAGAACAAGTGATACGAGCTTTAGTACAAATGACTATATCTGGC

>BPA_4239

CCGGCACCAGCGCCAACATCACCCTTCTTGTCTGCACCGCCAGGGGCACGTCTGTAAGCTGAACGATCTTCACCAGTCTTGCTAACGTCACCAGAACGGGCAGCAGCAGCTGGGCGGGGACGTACAGTTTCGGGTCTGGTGGGACGTTTGAGAGTGGCGGGAACAATCTCAGGAGGCAAATGCAAGTAACTGCGCAAGAATTCAATACCATCGTTGGTCAAATACCAGTAGTAATGTCTCCAAGCGAATTGTTCTTTAACCAAACCACGAGAGTGTAGCGACTGCATTGTTTTGATGACATGCAAGTTTGGAATACTTTCAAGCTCAGGGTGCTTAGGAGCATGGGTATCTTTTTTGGCTACGATAACACCCTCTTTGAAGAGGTATTCGTA

>BPA_4241

TGCAGATCTTGGTGGTAGTAGCAAATATTCAAACGAGAACTTTGAAGGCCGAAGTGGAGAAGGGTTCCATGTGAACAGCAGTTGAACATGGGTCAGTCGGTCCTGAGAGATGGGCGAGCGCCGTTCCGAAGGGACGGGCGATGGCCTCCGTTGCCCTCGGCCGATCGAAAGGGAGTCGGGTTCAGATCCCCGAATCCGGAGTGGCGGAGATGGGCGCCGCGAGGCGTCCAGTGCGGTAACGCGACCGATCCCGGAGAA

>BPA_4253

CAACAATCTGTAGCCCAATCGGCAACAATTTCTTTAGCCTCTGGCACGCAATCTGGCATGGCTTCGGCTTTAATAAGGGCTTCATCAGCCTCCACTTCAGCCAGTTTAACGCAACAGCAACAACAGAATCAGCAATCAACAAATGATAGTAATGGTTCTGGTAATAATTCCTCCAACAATACATCGTCAACTACTAACAGCACCA

>BPA_4257

GGTTGCGGCAGATGCTGCTGCTGCTGCTGATGATGATGTTGAAGGAGATGTTGGTGAAGATGATGACAATTGTATTGGTTGTTTGTTGGTGTTATAAACAATTGTTGAAAATTGATGTTGTTGCTGTTGTTGATGTGCCTGAGTTTTATTAAAACTTTGAGGTTCTAAATCAACAACTTCTCCACGTTTGTGTTCATTGAAC

>BPA_4260

TTACTCTCATCGGCCTCTGCATCAGCTGTTGCTATTCTTCCAAGACGTCGGGCGTGTATAACCGATGAGGGTATTAAATCTAAAGGATCGCCCACAGTTTGTTGTACTAATGCGCCCGTTGTACCAGCACCTGTTGTTGATGTTCTTAATGTTGATCGTGCAACATTGCTTGCTGCATAACGGACACGTTTACTAAGCGAAGATGAATCGTCTGCATAGGCATTATCGTCTTCATCCAGGCACATAAACTCCTCTTTACAGAGGATTTTTTCGAAATTTTGATTTAAATCAGTCGAA

>BPA_4273

TGGTCGATGACATAGAATTGGATCCATCATTATTTGTAGATCCAAAAATTTTAGTCTCCAGTACACCCTTCTCACCGGGACGCAAGAAGCGTGATCATAAATCATTTAGTCCCCTAAAAGATGAAAGTTCACCGAGTCCACGGAAGGAACAATTGAAATTGGCTAATGAAAGAATACCACCTACTGGTTTAAGACGTTTAAATATTGATGAAGAGGATGA

>BPA_4298

GCAGCTTGCTCACGAGCAAATTGTTCACGTTGAGCTTGTTCTCGAGCAGCTTGCAAACGAGCTTCTTCTTCACGCAATGCTCTAAGACGAGCCTCTTCCGCTTCTCTGGCTGCTTGCTCACGAGCAGCTTGTTCTCGAGCTGCTTGCTCTTTAGCAGCCATTAAACGTTGTTCCTCTTCACGCAAAGCTCTTTCACGGGCTGCCAATTCTAAAGCTAATTTCTCACGTTCAAACTGTTCACGTGCTGCCTGCTCTTTTAA

>BPA_4324

ATCAAAAATCTTTAAATTGGAAACAAAAACTTGATGCCAATGAAGAAGAACTCGATAATTTGCGCAAAGAAATTAAAATGTTACGTAAACAAAGTCAAGATATGGAAAAAAATTTCAAGGATCAAATAGAGGCCATAGAAATTGTACGCAAAAAAGATCGTGATGAGACCGTACAAGTAATATCGGAAAAAAATGCTTTACTGGAA

>BPA_4339

GAGGAACCCTTAGCGGCGTTGATCAATTCATCGGCCAAACATTCGGCGATGGTCTTGATGTTTCTAAAAGCAGCTTCGCGAGCACCAGTGCACAACAACCAAATAGCTTGGTTGACACGACGAAGGGGAGATACATCGACGGCTTGACGACGTACAGTACCAGCACGACCAATACGGGTAGAATCTTCACGGGGGCCAGAGTTGATGATGGCGCTGACAAGAATCTGCAAAGGATTTTCGCCAGTCAACAAGTGAATGATTTCGAAGGAATGTTTGACAATACGGCAAGCCATTAGTTTTTTACCGTTATTGCGACCCTTCATCATCAAGGAGCAAGTCAAACGTTCAACAATGGGACATTGTGCCTTACGGAAACGCTTGGCGGCGAAACGACCAGCAGAGTGAGGTAAATAACGAGCAAATTTCTCCTTAACAGAGATGTAATCTTGTAAGGAGATGTCATTGACGGTCACATCATCGCAAGACCAGCGACCGAACAATTTGATTTCGGGCAATTCAGTGGTGGAGACAACATTGGTTTCCAACAAAGTCTCAGCACCCTCA

>BPA_4364

CTGAACTTGAACCATTTGTATTGGAATTATAACCAGGCGGCAGTTGTGGTCCTATTATACGTTGTGATCCACAACCCAATGTATGACCATTCATTTTAATCGATTGTACAGATTCCTGTGCTCCACTCACTTGTGTGCTAGTGACATTTTTTGGAGGTTGTGTATCCATTTCGTAAAATATTATATAAGCATTTGTATTACAAACATTTTGCATAGATATTTG

>BPA_4367

ATTGTCATGATCAACTGGTATGGATGAAACACGTTCGGCACGTAGACAAGCAACAGTTGTGCCAGGACGACCATTTTTACCGGATGCTGTCAATTCCACTGTCATATCCATAGATTTTTGTAAAACCGCATCCATGCGTATGAAGCCTTCGGCTAAAGTTTTATAACCCAATATGGTGCGTGATTTATATTTTTTGCGTCTTTGCAATAAGATAACCAGTCTATTACCA

>BPA_4372

TTAAAATGGGGGAACTGCCAAGCGTATAACAAACCAGCAAGTATCCAAGCTCCGGTGTCAAGACTTCCACAGCAACCTGCCCATCCCATTAACGGGGGAATTGCACCAACAGCCGATCCTACCCATGTATTTAAAATTGACATTCGTTTCATTGGCGTATAAACAGAAGTATAAAGAATCAAATTTCCAAGGCCTAATGCTGCTGTTAGACCATTTACTCCATAGTAAAGCATGGTTAAACCTGAGATAGATGAAACGAC

>BPA_4373

ACATGAAATTCTAAAAGAACAATTCAACAATTATATTCGCATAGCGGAGGCTTTCATGAAAACCCTTAAACGTCCCAAAGATATAGAAATATGTACTAACCTCTTAAGTAAGGTGCAACGTTTAAACGATAGTAAACATTTGGAAGTTAAACGTAATAATAACGCATTTTTTCGTTATTGTTTGAAAGTCTTAAAATGGACAAGTGATAATCAGCCATTAGATTTATATAAATGTTGGTATCCTGCCA

>BPA_4376

TTTAATAGTTCTATGCAGGGCATGCCAAAATGTTCTATAGCTCATGAGATCCATATATTCGTGTAAAGTCTCCTTAAAACCGGGAGATTTATTGCCAAAATAAGCTTGTTTAAGTTTCAAGGCCATTGTCGATAGTTCTTGTGGCGTATGTTTGGAAATGATATCATATGGTAGAAGGTTAACACAATCTGTAAGATCGTTAATCAAATAGGGAGTTTTCAAAACTGTGCTATA

>BPA_4382

GATGTTCAGGCGGAAGCTATACCTTTAATTTTAGGAGGTGGCGATGTGTTAATGGCAGCGGAAACTGGTTCTGGTAAGACGGGAGCATTTTGTTTACCTATATTACAAATTGTATGGGAAACCTTAAGGGATTTGGAGGATGGTAAGGCGTCTAAGGGGGCAGGAGCAGCTGGCAGTGTGCCACATTGGACAATGTCCTTTTTTG

>BPA_4389

TTCATGTTCATGTTCATTTTTAACTTCAAATAACATTCATAATTGCATTTTGACTTCATCTATGGCAACAAATACCTTGGCCCCCTTTGCTCCTTTAACAGAAACTGATACAAAATCAACTAAACCCTCCCCACCATTAACTGGTAATAAATCCTTATCACCATCACATTCCCCCCCAAATAGTGCTGAAGATGCTAAACC

>BPA_4406

TTTTCATCGCCTAATTTAGTTGTACGCCTAAATGGTAAATACTACAGCTGGGCGGCTGCATGTCCAATTGTCATGACCATAATAACATTCCAGAAGCCTTTAACTAATGATGCCGTTGACCAGCTAATGGTTAATGCCAAACAAGATGCTGTAGCAGCAGCTGCTGCTGAAAATAAGGCAGAAGTTGATGGTGGCACTCCTACGGGCACACAAAATCAAGCCAAACGTTC

>BPA_4415

CCAGTATTGTGCATACAATGGTACGGCGAAACCCATTTCACCAGAGGGTAGAAAACTTTTACAAGAACGTTGTGGTTTTCTTTTAGAGAATGATACAACGGATTTTTGCTGTGATAAACAACAGGTGGATATTCTGAATAAGAATATTTTATTAGCCGCTAATTTCCTAGATCGTTGTCCCTCCTGTATGGCAAATTTAGTGCGACATTTGTGCGAATTTACATGT

>BPA_4423

CTCGGGACGCAACTTGCAAGGTCGAGTTAACCAATCATTAACGGAACTTTGTTGTTTCTTAATCCAATCACCCAATTCGGCACGTTGTTTTTGCTGTTGCTCTAATTCACTAACTGAAGCAGTCAATCCTCCTAAATGAGATTGAACCTGATCCAATAAGTCTTCAAGTTTAGCACGTACAATAAGCTCACGTTCGGGTATAACTTGCA

>BPA_4462

ATGAAAGGTTATAAGGGTGATACTGGTTTCCCTGGTTTGGAAGGTGCTAAGGGTGAAAGTGGTCCTAAGGGTTACAAGGGCGAAATGGCTGAACCGGCTGATTTACAATTACAAGAACAAGGTCAGCAGGGCGAAAAAGGTGAACCCGGTGATGCTGAAGAGGGTGAATGGAATCCAATTAGAGTTGAAAAGGGCGAAAGAGGTGATAGAGGTTTTGTTGGTGCACATGGTGTAAGAGGTCCTCCTGGTGAAGCTGGTGAAATTGGTCGTCCTGGTTTGCC

>BPA_4503

CCCCGATCTATTGGAGGTACTGATGAACCACGTAGATTTCTTTGTTTACGTTGAGTAGCTACATTTCGTTCAGTATCTTCCCGATCTTTCGATTGTGACATTTCATTTTTCTCAACATTTTGTTCACTTGGTTCTTCTTCCACAATACTCTCAGATTTTTCTTCAACCTTATTGACTGCTGCTTTCTTTTCGAATTGTTTTTTAATCTTTACATCGGAACT

>BPA_4519

TTCCGATTCCGATAGTTCATCATCATCCTCAAATTCCAGTGACGGTCACACATCACCCGAACCACCCGATCGAGAAGATGATGAAAATGCTAGAGATGGACATCATCAAATTCAAGAGCACCATCAACAACAACAACAGCATCAAGAAAATCATTATGTTACACAACAACAAAAACAAAATACAGAAGCTGGACTTCCACCGAC

>BPA_4555

TGTAAATTTTCCTCCTCAACGGGGCGATTAGAAGGCTGGAATAGATAATTGCCATCATCGGTGTTTGTGGGTGATAAGGGTTGCGTTACTGAAGACTTGGGTTGTTGTGGTTGTGACTGTATCTGGTTTTGTTGAGCTGGTACTGGCACATTTGAGGGCTGCAAAACATAATCATTATTTGCCGTTGTGCTAGAGCAATTCTCCAATAATTGTGAACACTCCTGACTAAATTTATCGGTTT

>BPA_4579

ATAAGTTCACTGCAGATGGCTTGACGAGAGTTACCCACCAAAACGACTTCGTCATGATTGGCCTCATCCTCACTGCTGTCATATTCCAAGGCACATTTGTTCGTTATCAAACTAAAGGCAAATACTGTTAAATCACAATGTCTGGCGGTAATGACTTCATGAACGGTACTCATGCCTACGGCATCTACACCCATGGTACGTAACATTTTCAATTCGGCCACTGTCTCGTAGTTGGGACCACCCAAACACGTGTACACACCCTCATGGACATCACTTTCAATACCCATTTCTTTGGCTACTTTACGGGCAATGTGTATGAGATGAGCATTATAGGCATTTGTCATGGGTGGGAAACGGGGACCAAAACGAGGATCATTAGGTCCTTGCAGAGGTGAATTGCCAGCAAAACCCATTATATTAATATGATCACGTACAATCATAATATCACCAACTTTGAATTTAGGATTGAGACCCCCAGCGGCATTTGTGGCCATTAGAAAACGTACTCCCACTAATTTCATAACGCGTACAGGCATAGAACATTTAGCCAAGGGATAACCTTCATAGTAATGAAAACGTCCCTGCATGGCCATAACGGGGAGTCCTTCCAAATAACCAAAAATCATGCGACCTACATGACCTTCTACGGTGGAAACGGGGAAATTGGGTATATCTTCATAGTCAAACGTTTTAGTATCGGTAATATTATCGGCCAAGGAATTTAAACCGGAACCACAAATAATACCAATGACAGGTCTAACAGAAGTACGTTCTAATAAGTAATCGGCAATATCCTTAATAACTTCATAGGAATAATTATCTTCATTTATCACCATTTCAACTTGTTGTGATGGTGTCAATTCATTTGTCATTGATGAATTTGTTGCTGACGTTTGATGTTCAGAGGCATTTTCACGGGCTGTATAAGAATTGCGTGTGCCATTTGTGGTTTCATTACCATTTGTTGAGGATTTGGCTTTGGTGATGAGACAGGTGGTGCCTTTGATATCGAAACCGGTCATATTTGTTATTAAATTATTGAA

>BPA_4584

CATATTACCTGTAACAATGAAGCCAGCTCCCGTGGAACCCGTCAAACGTTGCTGGGCACCTAACACATCATTAGGTGAAAGATTTGCTACACTACCCGTACTACTAACACCGGCAGAACCAGCTGCTCCCATGGTACCAGCAGAGGCTGTAGCTTGTGCTTGAGCCGTCAACATCAACTGTTGCTGTTGTTCTTTACGTTTCATTAATTGCAGAGA

>BPA_4606

CAAGCATTTTGTGGCATATAACGAGCCCGGTTTACTTCACCCTCATGATTTATTTTTATTTCTATTTCAATCTTACCACACACTGAACCAAAACCACCAAATTCACCCTTTTCGTTGTCATAATGGGACCCATCGAATTGGGCATCTTCACTCGGCAGCTGCACACTGGCTATTAGCAAGTGATTCTGTTCGTCCGATGTATGAGTACCCAAAATTAAACGATGTACG

>BPA_4618

AACGATTGCCCCCAAATGATTTCCATTTGGTAAATATTGTATCCACTTTAAAAGATCGTATGGGTCAGCAGGAAACTGTGGAGGAAAGTGAAGAAATACGTTTGATTTTTATGCAGCAATTGTTAGTGTTAGAAAATCATTTCATTTCAATGGGCAAAAGGAAATCCATTCAGGACTGTTACGATGATATTAATCCTATTATTATAAAAGCTTTAAATGATCCTTACCCAGCAGTTCAAAAAGAGGCATGTGCTTGTGTAACAATTTTAGCAGC

>BPA_4640

GTGGATGAGGACGAAGAATTTGAATACGTTATTACACGTGTTCCAAAAAGAAAACGTCAGAGTGAGCCAATTTATACCGATATTCCAGTATTTAATCGTAAAGTACGTAGACCAGTACCAGAAGCAATACCTATTGAAACCCATTTGAGAGCAAGATCGTGTAACGGCCAACCAATAGTTTTTGCAGCACCGCCACCACCACCACCACCTCCACTTATACCACATCCCCAATTAAGAAGATCTATACCAAATTTGCATAAATTTTCTACAGAATCAGAAGAATACTACTATAA

>BPA_4641

TAATGATTATATAGACGTTTATGATATATACAAACAACGTAGAAAATAGAAAAAATATCAGCCAGCCTTTTGTCCACATACGACCCAATTCACTGATATTTCCTTGTGAAATGTAATATTGCACAGCTATTAAGAGTGTGCCAACAATTCCAAATGCCACCAGTGTTAAAAATGAAAACATTTGACAATCTTCCTGTAGTGCTCTACGGCTTATAGACCGCGAGTACC

>BPA_4643

TTGGCCAAAGAGCCACGAATCTTCAAACGTTCAGATACAACGGAGGGAGTGATCAATTTGTACTGGGGAACTTCTTTGTACAATTTCTCGTATGTGGCTTTGTCGAAAAGTACTTGATTGTTAAGCTTGTCCCTTACTTTTCCTTTGGACCACTTCTTCTTCTTGGCCTTACCGCCGCCACTACCTTCCTTCTTCTTTTGTGTTTTTTGGGGCTGCTTAGCCGAGGATTTTGGGTCCTTCTTGGGTGGCATCTTAAAA

>BPA_4645

CCTCAACCAATTGTTGTAGTAAACGTTCAGGTGTACCCCTTATTACCACATGACCGCGTTTAGCATTACTACCCGCTGTCGCTGCATTACTACCCGCCGTTCCGGGTGCATCATTTGTTCCTATTTGTCTTAATTCTGTGACCATCACTACACGTCCCTCTTCATCTTCATGTCGCCGAATATTTTCCTCTCCTTGATGTTGTATACGATAGTAATCGGTTTGTGTTATACACACAAATTGACAATCATCACATTTTGTACGC

>BPA_4650

GTCCATTCCATAGTATTAATTTCGATCTATTTATAATGGAAGCAAAAAACTCTCTTGATTTTAAACCAATATCTAAACCCATAAAATCATTTGGAATACCCGATTCAATGGTAGCGTAGCAAACATCAGTATCATCCTCAATTTTAACTCCACAGGTAAAATCCATTGGCAAATGAATTTCTACATTATTTTTGCTAGCTTTTTCTATTATGCTCAACACCAACTTTGAACCTTGTGAATCAAACAATGAATTTCCAATATTCATATCACATATAACTTTAAGAAATGTAAATGCCATTCCACCACCTATAATCAATTCATCAACTCTATTCAACATATTTTCGATTAGTGGCAATTTGTCCGATACTTTGGCACCACCTAAAATAGCCAAAAACGGCTTTGTTGGTTTATTTAAGGCCCGGGAAAAGTATTTCAATTCTTTATTTAGTAAAAATCCAGCAGCTCTATATTTATAGCCTTCACCCAACATTGAACTGTGTGCTCTATGGGCAGTACCAAATGCATCATTAACATAAATATCGGCAAGTTGGGCTAAACTAGTACGGAATTTTTGTACATTATTTTTATCAGCCTTTACTTTAGTTCCCTTGGCGTCAATTCCTTTGCCTTCTTCTTCAATATGAAATCGTAGATTCTCTAATAAAATAATGCTACCATTAGCTGGATTTTTGCAAGCATTTTCAACTTCTGACCCCACACAATCATTTAAAAACAGTACATTTCTATTCAATAAACTTTTGAGTTCATCCGCCACTGGTTTTAGGGAGAATTTGACATTCCTTTGCCCATTAGGTCTACCTAAATGAGACATTAAAACT

>BPA_4652

CACCTGTCACTTTGTCCCCGAAGGGAAAGCTCTATCTCTAGAGTGGTCAAAGGATGTCAAGACCTGGTAAGGTTCTTCGCGTTGCTTCGAATTAAACCACATGCTCCACCGCTTGTGCGGGCCCCCGTCAATTCCTTTGAGTTTCAGCCTTGCGGCCGTACTCCCCAGGCGGAGTGCTTAATGCGTTAACTGCAGCACTGAAGGGCGGAAACCCTCCAACACTTAGCACTCATCGTTTACGGCGTGGACTACCAGGGTATCTAATCCTGTTTGCTCCCCACGCTTTCGAGCCTCAGCGTCAGTTACAGACCAGAGAGTCGCCTTCGCCACTGGTGTTCCTCCATATATCTACGCATTTCACCGCTACACATGGAATTCCACTCTCCTCTTCTGCACTCAAGTCCTCCAGTTTCCAATGACCTTCCTCGGTTGAGCCGAGGGCTTTCACATCAGACTTAAAAGACCGCCTGCGCTCGCTTTACGCCCAATAAATCCGGACAACGCTTGCCACCTACGTATTACCGCGGCTGCTGGCACGTAGTTAGCCGTGGCTTTCTGGTTAGATACCGTCAAGGTGATAACAGTTACTCTATCACTTGTTCTTCTCTAACAACAGAGTTTTACGATCCGAAAACCTTCTTCACTCACGCGGCGTTGCTCGGTCAGACTTTCGTCCATTGCCGAAGATTCCCTACTGCTGCCTCCCGTAGGAGTCTGGGCCGTGTCTCAGTCCCAGTGTGGCCGATCACCCTCTCAGGTCGGCTATGCATCACGGTCTTGGTAGGCCGTTACCCCACCAACTAACTAATGCACCGCGGGCCCATCCACCAGTGACACCCGAAAGCGTCTTTTATACTTTCGCCATGCAGCAAAAGCAATTATGCGGTATTAGCACCTGTTTCCAAGTGTTATCCCCCTCTGGTGGGCAGGTTGCCCACGTGTTACTCACCCGTCCGCCACTCATTTCTTTTCGGTGGAGCAAGCTCCGGTGAAAAGAAATGCGTTCGACTTGCATGTATTAGGCACGCCGCCAGCGTTCGTCCTGAGCCAGGATCAAACTCTCCAA

>BPA_4654

ACATTTTAAACAATAATGTCACTTCAATACCAGCATATGACAAAAATATTAAACGTTTACATCCATTAATAAATTATGGGAGCCTAAATTCCACGCCTGTTATTTATAGCAATTGGATTGGAGTGCCAACTAAACCCATTATTGGAACACATTTCAATCATCTGCATCATCATCATTTAATGGGATTGCATGCAAAACGATTCCGGAAACAGGGTATTGATCGAAAACCACGGCAGGCGTACAGTGCAAAACAACTTGAGAGGCTGGAGAATGAATTTAAACAAGACAAATATTTAAGTGTCAGTAAACGTATGGAACTTTCAAAATCACTGAATCTAACGGAAGTTCAAATAAAAACATGGTTTCAAAATAGACGAACGAAATGGAAGAAACAATTAACATCCCGTTTAAAAATTGCTCAACGACAAGGAGTATATGAAAATAACGTTTACTTAGGAAATGTTGTTAATGGTGTCAATATAGGAATGCCAAATAATTCGGTACCACCACCTACCTCCAATATTCCACCAATACTTC

>BPA_4658

CGGATCGTGGTTTGTGCGGTGCTGTCCACACTGGTGTTGCCCGTTACATTCGCGGTGAATTGGCCAAGGATGAATCAAACATTAAGATTATCTGCGTTGGTGACAAGTCTCGTGGTATTTTGTCTCGTTTGTACGGCAAAAATATCTTGATGGTTGCCAATGAAGTTGGTCGTTTGCCTCCTACCTTCTTGGATGCTTCTCGCATTGCTCATGAAGTTCTCAAGTGCGGTTATGATTTCACCGAAGGACAAATTGTATACAACAAATTCAAGTCGGTTGTATCATACCAATGCACCCAATTGCCCATTTTCGGTGCACCCGTCGTAGAAAAGTCCGATAAATTGGCTGTTTACGATTCATTGGACGCTGATGTCATTCAAAGCTATTTGGAATACTCCTTAGCTTCTCTCATCTTTTACACCATGAAGGAGAGTGCATGTTCCGAACAATCTTCCCGTATGACTGCCATGGACAATGCCTCCAAGAATGCCGGTGAAATGATTGAAAAATTGACATTGACATTCAACCGTACCAGACAGGCTGTCATTACCCGTG

>BPA_4662

TTATGGCGTCCACCCAACATTAGTTGTGTATCATAAATCATATAAACACTAAAGACAACAACACCCATTGAACTGTACAACAAGAATATGGGACGTAAAGGGATAAAAATGGTAAATAAACTAAAAATTAAAAATATCACCAGTGACATTAATAACCGCACATCATGGTGAAATCATATTTTGTTTGCAAAGCATAAAGACTCAGTGTCAGACATATGGCAGCCGTTGTGCCCACAGCCAACAGTATTTCAAGTGAATCATATTGACAAGTTACAATAGCCAAGAGCAAAGATTGTGCCAGAGTAAAAATAGCCAAAAATATATAGTTCAAAGGTGTCGTACGTCGTACATCATCACAGCAGGCCATAATTAACATGGTCACCACGAGCACAATAAAGGCTGGCCAAAATATGACTGGATTTGTTATGAAAATTGCCTTTAGTGATTCATTCAGCATGAAAATTGCTATAATTGAAAAGGTCATCAGTAGTTGGGACATT

>BPA_4664

TTTGAGTCTAAAGATAATAAAGCGCCACCTTCAACATTGTGTTCCTTAAAAACGGGTATATAACGTTCCATTTCAATGCCCAAAAGCCAATGACCCACCTGCTCTTTGGTCCATTCATGTACCGGCCCTCCTTTATAGTTATAAGCTGCAGCCTTGCGTTCTGAAAAACTTGAACTAGAGCCAGATAAATCTAAACCTCTGCTAGGTGAATAACCGGGCGATGAGCAGCCTGATGACATGCTCGTTGGCGAGGGAGGACCATTGCTATGAGTCGAAGTTTGCTGTTGCCATGGTACTTTTGGTTGTGCCTCATTTACAGCTTGTCGTATTTCATTTATTAAAGTTTTACAGTTTATAGCTGCCACTGATTGTGATTTATTTATTTCGGTATAATCATCGCTGGAATGTCGTGATGAGCCATCACCCAGACGTCTTTCTCTCTCCGTTAATGCTTGATTTAATTGTTCCGTAAATGATGTTGGTGGTAATTTACCACTTATAATTGCACCCGTTGGTCCGACACGTCGCCGGCTTGGATACATCCAATTTTCTGATGATTGTTCGTTTTCCGATAGTTTATCGTTCGATCCTAAATCGATTTTTGTTTTATTAGCCGAATTATCCAGTAATTCATGTTGTGGCACCGCAGCATCCAATTCATCTTTAACCGGATTTTTACGTTCCACCGTAGCAGTCTTAACTCCTTCACCGTCTGGTGATAAATCAGATATTTCTGTATCTGAAAGTTCAGTTTCTAATTTTTGGAATAATGGTTTGGGTGGTGCTTTACGCATCATTTGTGGTGTCAGTTTTAAAGTGGCACTGTCATACGGTAAACCAACTGGAAAACCACCTTTACGTTGTGTCTCCTGCAATTCATGTTCCAAATTAATGACACGATCTTTAAGTTTTTTCACCAAAGCATTGTATTCCGTATCTTTTTCTTGTAACAATTGCTGATAGAATTCTTCTCGATGACACATGTCCACTTCACGTTGTTGATATTCTCGCACTAAACGTTTGGCTTTATTGTATTTCTTGTCAAGGGCCATACTAAACGTTTGGCTTTATTGTATTTTTTGTCAAGGGCCATATATTGGGCCTGTGACTGTTGTAACATATTTTGTAGATTAGCAGCCTCTTTTTTAATATTGACGAGCTCGCGTTCGCTTTGACGTAAACGTTCGCTGAGTAGTTCATTTTCGTTGCCAGTTGTTTCAAGTTTGACGAGCTTTCTTTTAAGTGTGTCTATTAAGTCATCTTTTAAAAGACAACTCATTTCACTCTCTTGTAGCAGCCTCTTAAGAGACTCTACCTCTTGTGAATGTGTGGCTTCGACCTCCATGGGATGTTCGACTTGTACGGGACTGGTGGGTCCCTCACAAACACTAGAATTAGCCGAAACGGGTTGTGTTGAATCTTCAGAGTAATCCAAAGTACGTCTGAGATACTCTTCTTGTTGTCTTTTTAATCTTTCTTCCTTCTCTTTATCCGCTTGTAAACTTAAACGTATCAATTGAGCCACTTCACTATTTTCGGGATCACGTTCACGTCCTATTTGAAATTTGACTAGACCTGATGTATTACGTAATACAGATGCTGCATATGCCTGAGTAACGCCCACTAAACTTTTACCATCCACCTCGATGATTTGATCATTGACCTGTATGCGTCCGTCACGTGCTGCTGCACCGTTGTCTGTTATGGTTTTTACGAATATACCTAATTTCTCTAGACCGGCATCGGCACCAACGCCCATACCAATAATGCTGAGACCTAAACCCTCGGGTCCTTTCATAAGTTCAACGGGGAAAACGTGCATTTTTTCGACACGTTTCTCCAGTTCATATTCCGCTGAAGCGGCAACAGGATCAACATCTTCATTGCGACGACGGCGATCATAATCGTTGACAGAGAATGTGGAAAAAACTTGTATTGGTCCCGAGCTAAAACGAACTTTGGGATTTTTCTTGACTGGTATGGGTGGATAAGAAAGTTCATCGTCGTCAAAGTCTAGCAGACCGGGTACTTCCATCCAAAAGTTGCCATCTTCAAAGTAATGTACTCCCGATTCAACATAGACTTCTTTACTACGTACCGGCGGATATTCGGGTATAT

>BPA_4665

GTTGAAATGTTTACAAACGGCGACCACGACGACCACCCTTTCTGCGGGTAGAGTCAGAGGGAATGGGAGTAACATCTTCAATGCGGCCAATCTTCATTGAAGAACGGGCCAAAGCACGAAGAGCAGATTGGGCACCGGGTCCAGGAGTTTTGGTCTTGTTACCACCGGTAGCACGCAATTTAATGTGCAAGGCAGTGATGCCCAAGGTTTTGCATTTTTCAGCGACATCTTGAGCAGCCAACATAGCGGCGTAAGGAGAAGCTTCATCACGATCAGCCTTTACCTTCATGCCACCAGTGACACGGGCAATGGTTTCACGGCCAGACAAATCAGTAACGTGAACGAAGGTATCGTTGAAACTGGCATAGATGTGAGCAACACCGAAAACATTTTCACCTTCACGTACTTGGGGACCCAAAGAAACTTGGACTTCTTCTTTTTGAGCTTTAG

>BPA_4666

AAACATTTATAATAAAAATTTCCTTAATTCAGGCCAAAAATTACTTCATCAAGAGATCAAGCCGATATGCGTCTACATAATAAAGGGTGCAATTGTAAACGTTCCGGATGTTTGAAAAACTATTGTGAATGTTATGAAGCTAAAATACCCTGTAGTTCGAATTGTAAATGTGTGGGATGTCGCAATGTTGAAGATCGTCCAGATTTGGATATGGATCCAATAGATCCAAAAATAATGGCTACCATTGCTATGGGTGGTGCAGCTGCCGATAGTATCTCACTTAAGCGTACCTATGACAAAACACAAAGAGATATTGGTGGCACAGGCATTAATATTAAAACTGAAAAGAAAGAACCCATTAAATCTTTTAGTAATTATGACACATTGACTGGACCCGCTTCACCTACATTTGAAAGGCCACAATGTAATTTTATAACACAAGAAGTAATTGATGCCACCATACAATGTATGATAACACAAGCAGATGAATGTGAAAAG

>BPA_4672

GTGAATTCTTTACTCAGGAACATCCCATATCGTTTAATGTTCAGGAAACAGATTTGCTCAAGGAAATTATATCATTTCTACATCATGTCACCAAAGAGCCCGAAGTTGCTGAGAAAATAATTAAAGATGGTGGTTTGGTTCATCTAATGGAATTGTGCAAAATTTTCGCAGACGATAATGAAACTCTATCGACTCTGTGTAAAGTATTAGCAAATATGTCAGTGGTTAAAGATAGTGTAGAACACTTTTTTGCTAGTGGCTGGATTAGTGTTTTAGCCGAATGGCAACAATGTCCAGATTTGCGCCTGCAAGTTATATCCGCCAAAACTATGGCAAATCTCGATCATGATGATCCAAATTATACCGTTTATGCACCAAATGTTTATCCTTTACATCCACGTTTGCGTACCCGTAGTAAACCCAAAGCTGATATTATATTTGTTCATGGCCTTTTGGGTGGTGTGTTCATAACATGGCGTCAAAAAGATCGCAATCCCACCGAATTGGGATTATATGGAAAGAATGCATTTTATACCAGCGAAACCGATGATGTTTTCTTAGTTGGCGAACAGAGACGAATTCAATTGAGAAAGAATGGTAACGGCAATGGCAATGGCAA

>BPA_4681

AGAGCATAACACAATAATAATAAATAAACAAAACTAGAAAGAACTCAGCTTCATCATGGCGGATTTTGATCTAAATGTGGACAGCTTAATACAACGTTTATTAGAAATGCGCAGTTGTCGCACGGGCAAATCGGTACAAATGTCTGAATCGGAAGTACGTGGTCTATGCTTAAAATCCCGTGAAATATTCCTACAACAACCCATTCTTTTAGAGCTCGAAGCACCTCTCATCATATGCGGTGATATTCATGGCCAATATACAGATCTTTTGCGTTTATTCGAATATGGCGGTTTCCCACCAGCCGCCAATTATCTATTCTTAGGTGATTATGTTGACAGAGGCAAACAATCGTTGGAAACAATTTGCCTGCTTTTGGCCTATAAAATCAAATATCCTGAAAATTTCTTTTTATTACGTGGCAATCACGAATGTGCCAGTATTAATCGTATTTATGGCTTTTATGACGAATGCAAACGACGTTACAATGTTAAATTGTGGAAAACTTTTACAGACTGTTTTAATTGTTTACCCGTGGCCGCAATTATTGATGAAAAAATATTCTGTTGTCAGTGGTTTGAGTCCTGATTTACAGGGTATGGAACAAATTAGACGCTTGATGCGTCCAACAGATGTACCCGATACTGGTCTATTGTGTGATCTGTTATGGAGTGATCCAGATAAGGATGTACAGGGTTGGGGTGAAAATGATCGTGGTGTTAGTTTTACATTTGGTGCTGATGTTGTATCGAAATTTTTACATAAACATGAAATGGATCTAATATGTCGTGCTCATCAGGTCGTTGAAGATGGTTATGAATTCTTTGCCC

>BPA_4685

AAAGAAAGTTGGGTGGAAAATTGGCAATCGAAATGGTTACAAATCAAAAAGAGCAGAAGTGATGAAGTAAAGGAAACTCTCAAAGTAACCGAAAAATTAACAGCCGAACGTCGTGCCGCAGAGGCTGAAATTCAAGAACGTTTGCGTAAATTCCAAATTGTTGATCCAGAACTTTTAGTGGTCGATGATTTTGAAGATGTTATTGAGGAGCCAGAATTTGTCGAATTAACACAGGAGCATAGGGCACGTATTAATGCAGCCATTTGTGGTCCATTGGATCAGGTTTTGGTTTCTAAATTCAATTTGAATATCACCAGACGCGATATACACACTTTGTGTGGACACAATTGGCTTAACGATGAAGTCATTAATTTCTATATGAATTTGCTAACCGAACGTGGAGAAAAGAAACACGAATCGCATGGTTTACCCACTGTCTATGCCATGAATACCTTTTTTGTGCCTCGTTTATTGCAAGCGGGTCATTCGGGTGTAAAGCGCTGGACGCGTAAAGTCGATATCTTTGCTAAGGACATTATGCCAGTACCGGTACATGTGGG

>BPA_4686

TCTGGATTAATTTTACGTGTAGAAATTGGTGGATCTGTCATTACCAAACGATCCCACCAATTCATTTTATTAATCTTTTCCAAGGTTATTTGTACAGTTTTACTATCTTGCAACACCCAAAGGCATTCTCCACCTTAACCTCGGCACACAATTGTCCATTGATAATGGGCTCCTGACCTTTAAGGCCCACCTTGAGAGTTTTCTTTTGTATGTCCACCACTAAGTCACGAGCTCTCAAATTGAATGTTACAGTAAATGGAATTTTAATCTCAACTTCACCTAAAGTTTGTGTCCAAATGTATTTGTCAAGAGTACAACCATTGCCAGAATTTGGTAACAATTTACCAACTTCACTCTTCTCAGATTCATCTTCTACGTTTTCAATAGGTTTTGATTTTTCTTCGTCAATAGCTACAGGAGTATTGCCTTCAGCAGCAGCAGCTTCCAAAAGTTTTTGACGTTTTTTGTCTTCTTCC

>BPA_4693

AAAAGTTACACTCAAAGTGTAACAATTTCTCAGAAGGAGGTGGACGAAACCGGGACCGGGACTTACAATGTCTATAGCAAAATTATTTGTTAAAGTATTCAATGAAGATATATTTGAAAAATTAACAACAGAATTAGAAAATCAAATTCAGCAGGAGGCAACCCAACAGGATGTTGTGGCAAAGGATGTGGAGGTGGCAGAAGTTGTACGGTGTTCCGAGGCACCCACCATAAGTTCAGTGGCCTCTCAAAAAATGTCAACTACTCGTACAGAGGATATGTGCTTGCCGGAGCAAATGGTTTTAGTGAAATCTTGGCTAAACGATAAACTAGATAATATCGAGGTTAAATACAAGGAGGATGTGCTAAAGCAACAATTGGAAATGCGGTCAAGGCTAGGACCAGAATTGGTCATATTTGATATTGATTTTTCCTCCACTGTGCGCATAGCCAACGATAGATTATCGGTCAGGTCGCAGGGTAGTTTCAACACTATCAAAGCCAATGTGTGCGTCTATGGGGGAAGATGGATGTATGAGTTACAATTGCACAGCAAAGGGGTGATGCAAGTGGGTTGGTGTTCCAGCAAATGTGTTTTCAATGAGAACAGTGGAGTGGGTGATTCAAAGTTAAGTTATGGTTATGATGGCAGTAAACAACAAAGTTGGCATATAGCAACAAACAAATATGGCGATAAATGGCAAATTGGTGATATAATTGGTATAACCATTGATGTAGACAACGAATTGATAGAGTTCTATCGTAATGGCAAATCTATGGGTCCAGCATTCGAGAAACTAGAAAAGGGACCAAATATAAGTTTTTTTCCTGCCATATCGCTGGGCTATAATCAGGGTGTACAAGCAAATTTTGGCAATGCACCCTTTAAGTATCCGGTACCAGGTTACATGCCATTGCCAAACCTATAATTACATTAGAAAAAGCTGATTTACTTTTGCAATATTTACAAAATTTGGCTGCAGTAGTGGCACGACATAATTG

>BPA_4695

TATAAATTAAGACGAAGCAAATGGGGTTTTTGCAACAAATACTATGGACTCAGAACCAAAGGAAATCCACAGCTTTATTGCAGTCTGTAACGGTTATAATAACAAGAAGCATTAAGAAACATTCACAGCAAGCGAGATTGGCAGAGGTAGGACGTTTGGAGCCGGCAAAATCTGAAGGTAAATATGAAACGGGACAATTATTTTTACATCGAATCTTTGGATATAGAGGAGTAGTTTTATTTCCCTGGACGGCAAGAGTATATGATCGTGATCTACACAATCCTAACAAAGCGAAGGCAACAACAGCAACGTCGTCGCAACACACTGCAAACATTGACAATAATACAACAGGGTCTTCTTTAAAGCATAACTCAATCAATAATTCAACGGATCCTTTGAGCTCAGGTGATAACAACAACAGTATAAGAAATGAAAATAGCTATAAAACCAGCAAATCTAGTTCGGCTGATAGTACAAATGAGAAAAAATCAAACTCAACAAGTCATGCTAACCCGGCTGGACCAGGGCAGGATACTTCAAATAATAAGGAAGTTAAGGGAAAAGTTCATACTTTCTATCAGGTCTTAATTGATTCACGCGATTGTCCTTATATTCGAGCTCAAACTGAAGCTGTTACTTTTTTGGGTAATCAAGATTCTAATCGTAGTTTATATGCCATACCTGGACTAGATTATGTATCTCATGACGATATCATGCCCTACTCTTCAGCGGAAAAGAACCCATTGCACCATGAACTCTTTGACAAGTTCTTAACGCATGTGCCCGATAAACAGCCTTCTTTTGAGGCAAAAGACACTTTAAAAACATGGCAAGAAAAGAATCATCCTTGGCTGGAATTAAGTGATGTTCACAAAGAAACAACAGAAAATATACGTGTTACTGTGATACCATTCTACATGGGTTGTCGAGAGACTCCTTCCTCTTCAGTGTATTGGTGGCGTTACTCCATAAGGTTGGAAAATCTTGGCATGGCAAGTGTACAATTGCGTGAAAGACACTGGCGTATTTTTTCTTTATCTGGTACTTTGGAAACCGTTAGAGGTCGTGGTGTTGTTGGTCAAGAACCTATTTTGAGTCCACGTTTGCCAGCTTTTCAATATAGTAGTCATGTGAGTCTCCAGGCACCCAGCGGTCATATGTGGGGCACTTTCCGATTGGAACGTGAGGATGGTCACATGTTTGATTGTAAAATTCCTCCCTTT

>BPA_4697

CTGTGATATTGTGGCTGGGAAATCGCCTAACACAACAATTGAAGTGGAAACTGAGGAATATGTGATATTTAAAGATATTAAGCCAGCTTCGACTTATCATTACCTGTGTATAACGAAAAAACATATAGAAAGTTTGAAGGTAATGACCAAAGAGGATATACCATTGATAAATCGTATGGCAGAAGGTCTAAAAGGTTTTTTCAAATCGCAACAAATCGATACAAATGATGCTTTATTTGGTTTTCA

>BPA_4705

CTTTTCCGAAAAAAAGTAAAAAGAGAAAAAGTCGTGATCCCGATAAACTTTTCCTCTTATCACTTTATGAGGAAATTAAACGAGTGCCAGAGGACATACGTCTGGATGTAAAAGCCGAACTTATTCAGGTACTTAAGAAGTACCAGAAAAAGGCGCCAGTAAAACAAGAAAAAACTCATCACGCTCATACCCAATCCTCTTCTAGTTCGGGCAAATCATCGGGTAATACAACT

>BPA_4717

CATTCGAGGTTACTGTTATGGAACGTGAAGTTGGTGATGCCAAGAAGGTTAAGGTATCTGGAGCTGGTCTTAAGGAAGGCAAAACTCATACCGAAAATATTTTCTCTGTGGATACCCGTAATGCTGGTTATGGTGGTTTGTCTGTATCCATTGAAGGTCCCAGCAAGGCTGAAATTCAATGTACCGATAAGGATGATGGTACTTTGAACATCTCCTATAAACCTACCGAAC

>BPA_4724

GCGATAATGATTATCAGCGGCTAATATAGTCTTATTATCCGATCTAAAGGCCACGCTATTAATACGTACACCTTCCCAAGATTCCAGCACCGTACCCTTGAGATCGCACAAATAGAATTGTCCTTTTTGACCGCCACACACAAAACGTGTACCATCACGATTGAATGTACCGCACGATAAACTATCCTCTTGATTCATATGATTCATTTTCATCAATAAGCG

>BPA_4729

TCTCATTTGCACTGGCCACAAAACTCAAGTGAAGGTCAGATTTGTTAGTTGCTGCGATTGTGGCATTGCTGGTGTTGATAATGCTGTTACCGTTACCGTTTCTATTGCAGCAATTATTGTCAGTATTATATTGTATTAAGAGTTTTTCATTACTTCCATCATTTTCGCTTGATTGATGCTGTTGCTGTTGATTATGGCCATTTTGT

>BPA_4760

CCACGATCACCGGTGCTGGAGGAAGAAATCATTGATGGTTTTGCTATATTGGAATTTAAAACATATGAAGACTTAGAGTTTGCAATACAATTGGGTCAAAAACGTAAAGAGAAGCGTTTATCTGCACTGGAGGAACTGACGTGTACATACAGCATTGAAGAAATTAAAATGCCAAAGATCATAGACACTGGACAGCCACATCCTGTACGAGGTGTTGGTGGTACAGGAGCTGGTGTTGTTGGTGGTGGAGCTAATGGCGGAACAAATATG

>BPA_4775

ATAGGCAACAACAAGCTACAAATGGTCATACCCAATCGGAATCTGAGACCGAGGGTGAAGTAGAGGATGAAGAACAACGTAAATTAGCAGCAGCCTTGATACAACAAAAAGTGGCCGATATAGATGCCATGAAATCCCAATTAAAGCGTTTGAAAGATATGATGGAAACTGTTAACATTATAGAGGCTCATACTGCTAAAGATCCCAAACTTGTAGGACGTACACCACCACGTGAAGTACCCATTGCATATGATCGTGAAC

>BPA_4807

TGGTGCCGGTGGACCCCAATCAGTGAATGCTGCTGTTGCTTTTGGTCAAGGTTCTAATGTGGCTGCTGTAAATGCTGCAGCTGTCAATGCCGCAGCAGTTAATGCTGCTGCTGTATCAAACAGTACGAATCAACCATCAACACAACAGTTACGTATGTTAGTTCAACAAATACAACTCGCCGTTCATAGCGGCTATTTGTCCAGCCAAATATTAAATCAGCCTTTG

>BPA_4817

GTTTTTCCTGTACCAGGTGGGCCCTGGATAAGACTCAAAGGACGTTGTAGAGCATGTTTGACAGCATAAACTTGTGAACGATTCAAATCGGGTAAATTAGGTGCACTATACAATTTTGGAGGAGGACCTCGAAATACCAACTCATCTAAACCATCTGGACGTCCATGACCCAAAAGTCTAGCATAGATGTAATTCGAAACAGAATTACGATCAAATGCGAAAAG

>BPA_4829

TGCAATGTTGGGTGTGAGCAACGTATGGCAGATATCACAGACACGTGCTGGCCGCTTTCGAGGTCCAGATGGTACAGTTTTGGTTAAACATTTGTCACAGTAGATATGGCCACAATGACGACAGTGTAATTTGCGTACGGTAACTGTAAAATGATTATTACAAGCTGGACAATTCGCTACGTCATCGTCATCTTGCCAGCGAACTTCTGTATCGGCATGTCTTAA

>BPA_4838

TCTTGATGCTGAGAATCTGGTAGATGAAGAAGGAGAAGCTGGTGCCAAAGTACTTGTGGAGGGCGAATCAAAACGACTTATAACTGAAATACTCTTACGTGTTGGAGTACTCATGCTATGTCTTCTGGGAAGGTTTGAACTACTTTTATCTCCAGTTGAAGCTTGTGGAGTAGAGGGGGATCTTAACAAAGCTAGATTACTGCGTAAAGGTATACGATTTATAGGTTCTCTACGCGGCGTAGTAG

>BPA_4840

TTCTTTGTGATCTTCTACTTCCACTTCCACCAATTCCTCATTGGGATCATAGTCACCTTCAACAACATTTTTATATCTCTCTATTCGTTTCGCATCCCATTCTTTATTATATTCAAGAATTTTATCAGATTTCTTGAATTGTTTGAAGCAATTTAAGACATACGGATGCTTTTGACCCCAGGTACCTTCAATTTCTTCCGTTCCCAGATCGCCCAACTCTGGATCATCATATGTGGCATAAAAACGTTCGAAGCGATCATCTAGAAGCGTTAGTTGTTCATTGCGTCTTATAACACTAGAAGACATGGAATATTCGGTAAACTGGGATTTTATTTCTTCGTTTAGGAAACGGCGTTCTCGCATTAGAGGACCTAAACGATCCATCAACTCGC

>BPA_4844

CAGTGGAGCAAGTGGAGGTGGTGCTTCACCAGATGCAAACGCTGGTGGTGGTGGCACCATAGCAGGAGGCGGCGATAGTGGATGTGGGATTATAAACACTTCATCAGGATCTCCCACAGCATACCGTGAATCAAGAGGTCGCAGTGTAGGAGTTGGGCCCATGAGAAGACCTTCAGAACGTAAACAAGATCGTTCTCCTTATGGAAGTACTG

>BPA_4858

TCCTCGGGATCCATACAGTCAGCGAAGAGTTCCTCAACTTGTTCATCTTGCAAATTTTCACCCAAGGAAAGGAGGGCATGTTGCAATTCAGCCAAAAGCATAGTACCGTTTTCGGCTTTGTCGTACAATTTCAAACATTCAATGAAATCTTCGTAGCAACCTTGATCCTTTTCCTTCTTGACTTGAGAGTAGATGGGAAGGAATTCTTC

>BPA_4867

TGTATAAAGAGATTAAGGCTCTTAGTGGATTTCCGGCCAATAGCAAGAAACCCAATGAACGTTGGTGCATTGCAAAGGGGTTAATGAAACGTGAAAACGGTTTAACAGTTTGCAAAAATGGTTCTTTTAGCTCTAATCAACAAAGAAGGATTGCAAATTATTTTGTACCCAATAAAAAAGTAGATCGTTTAATGGCTTTGGATTCAAAGGTTTATATATGTAAATTTAAAGATGATGGATCCCGAC

>BPA_4887

ATATGGTTCCAAGTTTCTTAAAAGTATTTGCCGAAAATGATCCGATAGTTCGACTTCTTATAGATAACAGTCGTAATTTATTATATGTGCTCACTGAAAAAGGATCTATAGAAGCGTGGGGTATGGGACAAGATTGCAATACAATGCGGCGCATAGCAAAAGTTACACAAAATGAGATTTCACAGAGCGCGGGAAATATCGTGAAAAC

>BPA_4891

ATTTATATTATTATAAATAACAATGGTTAGACGTGGACGTTCTGCAAGTCCTCCACCAGCTGGAAGAAGAACTGTTCCAACTTCCGCTCCCACTCAAAATGTTCCAGCCCGTGCTGCTCCTGCATCGCCACCGCCAGTACCAATGCAAGCAGCACCAAGTGCGGTAGGAGCGCCGCAACAACCTTCAATGTTTCAACAAATGGCTGCTACTGCAGGAGGTGTGGCTGTAGGATCTGCTATCGGACATACTGTTGGACATGGAATTACTAGCCTTTTTAGTGGCTCTGACAAAGAAGCACCTGCAGCTGCTGCACCCATTGCTTCTCCCAGTCAAAATACAGTAGGTGCTCAACCCAATGAACCACAAGGTCCATGTAGTTGGGAAATTAAGCAATTTTTGCAATGCGCTCAAGCTCAATCTGATTTAACATTG

>BPA_4901

GTAATAATCAAAATAATAGCAGTACTATACAAGTAATGGGTAATTCAACATCTATGATGAGTGGTGGCAATCAGTATACAACTCCAGCTAAAAAATCACAAATATCACCAGCATCTGGTATTGCATCAACAACAACAACAGATGAACATAGTAAAATAACAACTGTACATGTTGCAGGTACTGATAATGCATATGGTACATGTTTTACACCACCACCAGTATCATCGTCATCATTATCGTCTGCTTCAACATCAC

>BPA_4902

AATTTCGATAAATTAGTTTTGCAATCTGAAGATGATTGGTTAGTAGAGTTCTTTGCTCCTTGGTGCGGTCACTGCAAAAACTTGGCCCCAGAATGGGCCAAGGCTGCCACACAACTCAAAGGCAAAGTGAAATTGGGTGCTCTCGATGCTACTGTCCACCAAAGCAAAGCTGCTGAATATGGTGTAAGAGGCTATCCCACCATTAAATACTTCCCTGCCGGTAAGAAACGCTCTTCAGATGCTCAAGAATATGATGGTGGACG

>BPA_4939

AGTTTTTCTTTATTCAACATTTTATAAATGTTGTCGTAACTAAACCAAAAAGACGCTTTGGCACCCGCAGCACCCAATATCTTGCCAACAATAATGGCAGTAATATAAGTTCACCAACAGCGGCAACATCACCAATCTATACCAGTAAAACCATTGTAACTAAATCCGTTTCCACAACGGGTGTTCATATTGACCAACAACAAGTGCAACAATCGACATCACCACCAACACCACAACCCTCTTCTATAATAAATTCCACAACTCATGTACCACTTGCAGCGC

>BPA_4943

ATTCCGAATCATCATGAAAAAACATTACAAAACGATTAAGATTTTCCACATCGGTGTTATAATTATCTTCCACTTCTTTCAATGAGTCACCATCTGCATACAATTTGAATTCCAAATAAAATCCCTTATTATACTGAAATTCCATATTATGAGCATTATCGTACATTAACATTTCCCCCTCATTGACAACCATAACTACATCCGACAATTCTTTCAATTCCAAAGATTCACTAGTACAAAAAATGATCGTTTTAC

>BPA_4953

GAATCTTTAATTCATTTCTCAATTTGGACATGGTTTCACTTACGACCATCTTCTCATTTTCATATTTCGATTTAAGATTTGTAAGTGCAACTTCAGCTGTTTGCTTATTCGATTTGAGCACTGTTCGTAGGGTGGCAATTTGCTCACGTTTTGTGGACAACAGACTCTTGAGTTTGATAATTTGTTCTTGCAATTCTTCGACTTCTTCGCGATTATATTTGTCGGCTTCTGTAATATCACCGCGTACTTTATTCTTATTAAGTTCAATGGTGTGTTCAACGGCAGTTTTAAGAT

>BPA_4954

GTGAAGAACCTTTACAAGATGGCCAAGACCCCGATATAGGTGTAGGTTTAACCGCAGATATTATTTTAGCTTTGGGTTGGAAAGGTTTAGGAGCAGTAAGAGCTTTACAATGTGATCATGTTATACGTGAAAATAGTGATCCATCTTCGGAGAATGGCGAACAAAAGTTAGCGGTACCTTATGGTTTAACTACTTCAGCGGAAGAATCAGATTCTAAGAATGTTTCTTACACATATACCCTGTGGGTAGGCTCAAATGTA

>BPA_4955

GTCCTGGTGAAAAAATATGATTTTTTTGTACTTGCTGTTGTTGCTGTAGTTGGACAGGTTGTGAACGCTGATGATGATGTTGATTTAGGGAAAAATGTGTTGGGAGTGATTGTTGTTGTTGATGATGTTTTTGTTGTTTCAGTTGCAATTGCAATTGTTGTATTTGTTGCTGATGAAGATTGTGTTGTCTTTGTTCTTCTAATAATTTTTGATGATAACGTTCTCGATAGACATTCGTATGTAATA

>BPA_4963

GAGAGAGCAACAACAGGTTTTTGGGCAGAATCACTGTAATATTGTCTAGCTAGTTCTAATTCTGATTCGAAAAATTTCACTTGCACTTCAAAAGCTGATATTTCCTTAATCACCTCTTTGTCTTCGTCATCATTTGTGAACTGCTTTGCTTTCAATATAAATTTACGCTTGTCTGAATCATATTCACATGGTAAGGTTTTCTGTGCCTTTGCC

>BPA_4965

GTGAGCATAACATCTCCGGTTGTTATTGCCAATCAACAACAACAGCTACAAAATCAGATGCATCATAGAGCTGCTGCAGTTGCAGCAGCAGCTGCTGCTGCTGAACAACATAAACAACAACATCAAAATAATTTAACAACTGCTATGCAAAATGCACAAAATTTGGCCGCTGCTGCTGCAGGAATGCGTCATCATCCATTGTTG

>BPA_4977

GCTGACTTGATCCAGTGTTTACCTGTGCAGGTGGAACAGTGGCAGCTGCTGCAGCATAGGCAGCTTGCGCTTGTATGTTAAAAATAAAATCGTTTAATTGTTGTTGTTGCTGCTGTTGTTGTTGAGGAACCACAGATGGTGTTGGCTGAGGTTGTGGTTGCAAAAATGCTGGACCACCCATTAAAGGATTTGACCAGTGTACAAATTGTG

>BPA_4981

TTTGTATCCAGAACAGTTTGCTGAAAACGTTAGAGATAGCTACCAAAGGCATGAAGTTGATCAACAACAAATAAGTCCAACAGATTTAGCAAAACAATACCTGTGTCCAAAATTCATGACAATTGATGAAATTGTACATGCAGCTAAATTTGTTGTCGCTCGTCAACTATCTAGAGAACCCCTATTAAGAAAAACAGTACGGGAGGTTTATTTTGAAAGGGCTAAGCTTAATGTTCGACC

>BPA_4987

CCTGAGCATACGAGTTTCTATTAATCTCTTCGGTATTTGTTGCTTGAATTTGAGATTTTTCTTTACATTGGACACCTAAAGATTTCTGAGTTGTTACATATGTTTGGGAATATTTTGAAATTTGTTTTAATGTCGGTATTCGACTTTTGGTTTTCCGATTTAGATTAAATCTATCGTTTGTTATAGATTGTTGAGATGTTGAAA

>BPA_5000

GGAGAAATGTGCAATATGAAAAACGAATCGGATGCAGTTTTGCCAGGACATCCGGAAAGTATTCTAGGAGCTTTATTAAATATATTTTGGAATTATTTAGAAGTTGATGAAATGAAATTTCTTTTAAAGAAATTAGCAAATGCCATGTTAAGTGATTTTACTCATACCAATAAAGGTTTGGATTATGAACAACAACGTCATGCCTTACAAATTTTAATATGCCTGTGTAATCATACGCGC

>BPA_5013

AATTCAAATACTACTGGAAGTTTTTTGAACAATAATTCATCAGCTAACACTGCGTCATCCGCTGAAAATGATTTACTGAGTGGCAATAATTCAGATTTGTACAATCCAAATAGTTTTATTACCAATGACATACCGGCTGATATATTTGAGGATACTTCCATACTTCCTGGAGGTGATAGTAGCAATATTTCAGTCAATGATAGTTATAAACAGGCCCAACA

>BPA_5015

ATGCAGAGTACAATATCACGTTTTGCATGTCGTATCTTGGTCAACAGATGTGAACCCTCAAAGGCGAGAATATTTGCAGCCGGTTTTGATTCGAGTAGGAATATATTCTTGGGTGAAAAAGCAACAAAATGGCAAGATAATGTGGAAATAGATGGCCTTACAACAAACGGTGTTTTAATCATGCATCCAAAGGGTCAATTTTGTGGTGGCAATGCCAA

>BPA_5019

AGGTAGGCACCGGTATCAACGTGACGCAAACGCACATGAGCATCTCGTACCCATTCATTGTTACTGCATATCAATTCCCAATGATCACCAGAATCTCCCAAACCATTGTCACCATAAGCGGATACTTCCTGTTCTCCGGACAGAGGTGAGGAGAAGAAATGCGAGTGTAAATTTCTCTTTGTGCTTAAATGTTCCAAGCGTATGGTATCACCA

>BPA_5031

AATCAACATCAAATACTGATATAAACTCAAATGATGTGTCACAAACAAAAATCAGTGATTCTACTTCCAATGAAGTCATAAATAATATTAAAACTTCATTATCCTCAAAACCACAACTGACCCTTCAAGAACATTTAGAAAGAAGTAAACCGAAATTTTTACAACAGTCCAAAGAACGTAAGGCTATACTAAATCAATTGCAAGCTATGCGTCAGGAACGCG

>BPA_5043

TGAAGGAATTAATTGCCCTTCGTGCCAGGATCTTCCGACATAAATGGCTGACCCATCACTGTCATGTCCCGCTAATACAGCATTGTAGGGAATTTCTCCTAACTGCACTGTTTGCCAGTTATCGTTGTTTTCACTTAACAATACTTCATATTGATTTATACTAATTTCTAGTCCTCCAAATGGTATATACAGACAACCATGTGAAGCATGGATTTTACCCACACTTAAACTGTTTGAATGATAACC

>BPA_5055

ACCCTGCAAATCTTCAATAATTTGAGGTTTCGATATAAAGACATCCGATTTGAATTGCGATTGTATAGCAGTTAATGAATCATTTTCAAAACTCATATCATCTGTCTTGTGATCCAATAATACACGTGTTGGCATTTCACCATTAACCGTACAGACTAAGTGATAAAGTTGAGCCAAATCATCACTCAATGCCACCAAAGTAGAGCGTGCTGATCCCAAACTTTGACCAGCATTTTGTGATATATTTG

>BPA_5062

GGGAATAACATTGGTATTGAGTAAGATACCCACAGAGGTCTCTAGGAAATTTTCTTCCACATCTACGGATCCCGCTAGCGAAAGGAAAGTAGTGGCTGTGCGTGATGATATATTGCCTTTATATATATTTCCCATTTCCATTTCTAATTTACCCCAGATATCCATATCCAGCAAGACGTCAAACGGTGTTTTAATATTAATTTGTAAATTATGTTCTTTGGGTTTCAC

>BPA_5117

TAACAAAACGTTTACCACAAATATTGCAAGGGAATGGCAGTTCCTGACCACTATGTTTGAAACTGTGTTTCTTCAAGGCCATGGGACACTTGAATACCTTTTCACAGTCGGGGCAAGGATACGATTTGGGAACATCTTCCTTGGCGATCTTGTGAACTCTCTTCAAATGTACGTTGAGTATGCGACGACGAGTAAACATCATCGTACAACCCTCT

>BPA_5168

AGTAGGAGGACGTGGTAAGTTTTCTTCAGCTGGTGAGGGTTGACGAGAACCGGGTGTACGATTGAATCCTTTGTCATCGTCAATGCCATTGACAACACCATTTGTTGTGGTCACTTGGTCATCCTTCTTTAAGCCATTCGTATCGAATGGAGAATTTGCCTTCTCTTTATCATCGTGTGTCTTATCGTTATCATCGAATTTCAATCCCCTCAGACGAGGTTCCAAACCTGACAAAGGACTTTCTTTATTTG

>BPA_5178

TTCTTGGGTGTGACATTCTTCGTTATTGCTTTCATTTTGGGTTACCATTGGTTGGATGCTGTTATTTTCTTGATTGGTATTATTGTCGCTAACGTACCTGAAGGTCTATTGGCTACCGTAACTGTGTGTTTGACATTGACTGCCAAGCGTATGGCTTCTAAGAATTGTTTGGTTAAGAATTTGGAAGCTGTCGAAACTTTGGGCTCCACCTCTACCATTTGCTCAGATAAGACCGGTACCTTGACCCAAAATCGTATGACTGTCGCTCACATGTGGTTCGATAATCAAATTATTGAGGCCGATACAACAGAAGATCAG

>BPA_5179

TGGCAATTGAAGACGATCTTTTAATGGAAAAACTTTAGCAGGTAAACTGTTGACATAAGTTCTCAATATTTCACTTCCTCTACATTTTTCCACACAAATTGGTTTTTCTTTATTCATTTTTTCCGGAGATTTTGTGCCTTCGATAACTTTTTTTATACAACCTTTGGGATGATCAACTACAATTACTACAGGATCAGCTCTTTGTTTTAATCGTTCATATAATCTTTTATAGATTTCAT

>BPA_5180

CGGCGGAACATCTTATATTCTCCTTTATGACTGGTATGGCATGACGCATTTTGGTAACAAATTTATAACTATGTAATTTGGGTAAATGTTGTAATTCCAAAGTTTCCAAAGTACGCAATGACTGATAGTATTCTTTTTTATTAGCCTGTTCACAGAACTTAATATAACATTCCAATGCAGGCAAACACTCTTGCAGGGCATCTATGGCTGA

>BPA_5181

AAATTGATTGCGGTGAACGTTATCCTGACGAACCTCCTACGATAAAGTTTTTAACAAAAATTAATATTAATTGTATCAATCAAAATGGTGTGGTTGATGCAAGACTAGTACCCATGTTGTCACGGTGGAACCGTGAATATACTATTAAGTCCTTGTTACAGGAAATACGTCGAATTATGACCTGTAAAGAAAATCTTAAATTGGCGCAACCACCAGAGGGCAGCTGTTTTTAGTTGATCAAT

>BPA_5191

ATAACCGTAACCATTGCCACTTGCTCCACTTCCCCTCTGTCCATCAGTACTTCCATAACCAAAACCGCCATTACCACCAGCACCACCATATGGACCATTTGGTCCTATACCACCATAACCAAAACCGCCTTCAGGACCACCGCCGCCGCCTCCTCCACCTCCACCACCACGTTGAGCGCTACAGATTAAACTGCACAATAATAAAATTCCAAGGTAAATGAACGTTTTCA

>BPA_5199

AAATGTTGATGGACTTTATACTGTCAATCAGCCTTCAACAGAAATAGTTACCAAATTGCCTCCCTTGACACAACCTATACCGGATTTACCAGAGGTAGAGTATGCCAAACCTTTGTTGGAAAGTAGTGCTACTAAAGTGACAACACTGCCCAATGGTTTAAGAGTAGCATCAGAGCCACGTTTTGGTCAGTTTTGTACTGTGGGTTTGGTTATCGATT

>BPA_5200

GGGTGAAAAAGAATTTTCCGATAGTGATTCACCAAGTCCTAGAAGAAATTATCGTTACAACGATCGCGCCAGTCCAGCTGATATTGCCACTGAGGTAAAACCAGCCAATAATTTAAACATGAACATACGTCCCAAATCGGCATCATCGATAGGTCCCGTTACAAAACAACCAACTCAACACAAAGCATCTGTGGGTAGTAAAAAAATTGATATGGGTGCTGCA

>BPA_5201

TACGAGGCGAAGCTACCATATTGTGGGCGACTGCGGTATTAGAGGCGGCAGGTGTATGGGTATTCATGGCATTATTCTCTAAAACGGAATCGCATTCATAGTTACGTTTCCTTGAAGCTACCGTGCGTAATTTAGCTCCTATAGCGCCACCATTACCTTCATGTGTAAATTCTGTTGTTTGCTGTTCTTCGTTACCATTGTA

>BPA_5254

GGCCACAGCAGCTGGTGCTTTCTGTTGTTCACCGGCAGCCAATTCATCGGGTGGCAATGGTGCTTCACCCAATTTACCATTTGTAATTTCCCATAAAACTTTATCGACTTCCTTGGCAGCCTCCTCTTCCATTTCCTCAGTTTCCTCTAGGGATTCCATTGTTTCCTCTAGCATTTCTTCTATTATGCCCGCTTTCATCATTTCTTTGGACATATCACGCATTATGCCGGCTAATTCTGGATAGCGGATTAAATTTTGCATTGCTTGCATAACTTCGGTGGATTTTTGTAGAGAACCAGCGACACGTAAAGTTGCCAATTGATTCTTCATTTGTAATTG

>BPA_5262

CGGGGATTGGTGATTGGGGTTAACAAATCCTCGTGGACCCTGCGGACCTTTTTGCGCCAACAAGAGAGAATATTGTTGTGATAACATTTGCTGATGTTGCTGCTGCTGTTGGTGTTGTAGACTTGGATGTTGTATGTGTTGCTGTTGTTGTGGTTGCTGTAGAGGTGTTGGTGTTTCAGATGAGTGCCTTAAAATATCATGTAATATTTTTTGTTGTTTTGCAATCATATCAGCTTCATTTTGGTGCTGTTCTTGCTGATGA

>BPA_5291

ACAGAGACAGAAGTCATGATAAGGAGAAAACGAGAGATAAAGATAAGGAACGAGAACGGGACCGTGAGAGAGTGCGCGAGAAAGAAAAAGATAAAGATCACGAATATGATAGGGAACGCGGCAAAGATAAAGATCATGCAAGGCATAGAGAAAAAGATCTTGAAGATAAAAATAAAGAAAGAGAACATGAAAAGGATAAATACCGTGAACGTAATCGAGAACGAAACGATAATGAGCGCAGTCGTAACCGTAGCAAAAGTAGAGTTCGCAAAATTTTAAGAACTGAT

>BPA_5295

TTTCAGCATTGTTAGGTAGACCCAACCAAGAGGGTGTTTGTCGGTCAGTAAGGTTTTCAATCCATTTTAGGAAATGATCTCTGCGTGTACCGTCTGGCATGGTAATATGACGTTGACCACCACTGGCACCATCCACATTGGCAACCAAAGCAAAATCAGCCTCAAACGATCTTGCGGTAAAGAGTTTCTTCAAGAACGAAGTCAAAAGACGTTGATCGAAATCGT

>BPA_5308

GGAAGTCCTTCAAAGAAAATAACTCAATTCCTCCATCAGCACATACTACTGAAAAAGCACCAATGTTTTCGCTTGTATTACATTCTGCAATGTCCGTTTCCATCACATGGCTCATAAGCTCAATATTTGGCAATATGCAAATATCTTTAGGACACTTTGCTCTACTAAATGTAATAGCTTTTTTATATTCATTAGGAATTATTCCATCTAT

>BPA_5310

GTAACGAAATTAAACTTGTAGTCCACAGGGATAGTAAAATTGCCTATACTCAAGGTCTTAGTGGTGATAGCTCTAGATCAGAATCAGCTTTACTCTCCACTAGCCCAGAATATCCTCATAGAGCTCCTTCTCCTTTTCTACCCGGTCCCGGGCAATATGATCGTGCTCTTCAAGTGCCCGTTGATACATTGCCCCACACAGTATTTCCTAAA

>BPA_5315

TTTAGATATGAATTTATTTTCTTTTGATTCTCACTGCCATCTAGGCTTAATGAATGTGTTCCATTGCCGGTAGCATTGCTGTTATTAGTTCTTAATTGTTCAGCCGAGGATACAAATTCCAATGTTAACCGTTCTACTGATTTTAACATTAAATCTGGATCTTTATGCTTTTCGGTACAGTTATTAAAGGAAACTGAAAACTCAGTTTGTTTGGTTTCCAACGAAAC

>BPA_5359

AATCAATAAAGCCAACCTGCATCATAGGTAATTCGTCCTCTTTTTCACGGTTCATAATGTCAATAGGAGTTATATTTAATTCCTGTTTTTCCATATCACCCTGTTCAAAGAATTCTGAACTAACCAAATCGGCTACACGTTTCTCAATTTCCCAAGGCTTGGTAATGGCCGATAAATCACATACAGTCATGCTCATAGCACGTAATAAAGCACGTGGCTCCTCATCAACCC

>BPA_5361

CTCCACCGAATCAAAATGTCAATCCAATGATGCAACAACAAATACGCATGCAAATGATGAATCAGCAACAGCAAATGCAACAACTTCAACAACAACAACAGCAGCAACAACAAAATGTTATGAATCCTAATATGTCTGGTCCAGGTGGTCCATCTCCACAGCAGCAACAAGTTCCAGTTTCCAATCAGCCAAACACCTCAATGGCCAACCAACAGAATCCTGGTCCTTCATCAGATCAAACTCTTATGCGTGATAAAATTTGGTCAGGTACTCTGGAGTGGGTGGAGAAAAGTAAGCCGGACCAACAAAAAATACCACGTACTCTACAATGTACTGTTACGGCCAACATCAAAGATGG

>BPA_5369

GTCCCTACCAATGTATGGACTGTCCCAAGGCTTTTAAACACAAACATCATTTAACCGAACACAAACGTTTGCATAGTGGAGAAAAACCCTTCCAGTGTTGCAAGTGCTTGAAACGCTTCTCCCATTCCGGCTCCTATTCCCAACACATGAACCACCGTTACTCGTATTGCAAACCCTACAGGGAATAGGTAAGCGAAAATGCACAATCC

>BPA_5391

GTAAGTGTTCAATCATCTTCAACTTCAACTCAGAATGTTGCTCCAATATCACCGAAATGGAATTCGCCACATCATCGTCAATCAAAACTACGTAATAAGGAAGACATAACTGCTTCGCCATTAAGTAAATCGGCAAATGGTGGTATGGATAAGAAGGTGAAATCTCAAAATACAACCCCAAAACAGCATAGTGAAGAACAACGCCATAAATCTCCAACACATGATCAACCAATATCATCTGTTCACATAGGTAGTCCAGCAAT

>BPA_5412

TACGAGAGAATATATCACGACTGTCTTGTTGTAAAGCTTCAAATTCTTCCCAAAAGCCACCCTTAACCAATTGTTCCACTCTGGCATTAATGCCAGCCGCTGTAATACGTGTGGCATTAAATGGCTGACGTAAATGCACCACTGTGCCACAGGTTTCAACCATGGGATTACGTTTATAATGTTCAATTAATTCCGATAAAGTGTTAAAGGGTTCACCACCACCAACA

>BPA_5428

GTTTGCGTGAGCATATTAAAAGAGATCGTAGTAAGGGTGGTGTACATTATTCATCAAAGGATTATGATAGAGAACGTGATCGCGATCGTTCTAAAGATAGAGATAGAGAACGCAGTAGATCATCATCATCATCTTCCTCAAGGGATAAGCGTGATTATCGGGATCGTGAACGTGAAAGGGATAGAGGTCATGACAGACGTCGTGATCATAGAGATCGTGAACGTGATAATCGGGATCGGCG

>BPA_5436

AAAGGTTAAGGATGCTAACAACCAATTGGCTCGTGAGAACAAAAAAATGGGAGATGATCTCCATGAGGCTAAGGGCACCATTAACGAAATGAACCGTCGTTTGCATGAACTTGAATTGGAATTGCGTCGCTTGGAAAATGAACGTGATGAATTGACTGCTGCCTACAAGGAAGCTGAAGCTGGTCGCAAGGCTGAAGAACAACGTGCTCAGCGTTTGTCTGCCGACTTCAACCAATACCGC

>BPA_5453

TTAAGATTTAGTCGCGTGTTTGATCTTAGAGTGAAATATATATTTTTCATATTTAATACGATTTATTAATTAAGATGCCACAATTGCAAAAACCAGCACCTCAATTTAAAGGCACTGCCGTCGTCAACGGAGTTTTCAAGGATATTTCCTTGGCTGATTACAAAGGCAAATATGTTGTTTTGTTCTTCTATCCCTTAGACTTTACATTTGTTTGCCCCACTGAAATTGTTGCTTTTTCCGATCGTGCTGCTGAGTTCCGCAACATTGGCTGCGAAGTGATTGCTTGCTCAACTGACAGTCAATACACTCACTTGGCTTGGATCAATACACCACGCAAACAGGGTGGTTTAGGTGAAATGGACATTCCCTTGTTGGCCGACAAATCTATGAAAATTGCACGTGATTATGGTGTTCTGGA

>BPA_5455

GTTTTAATGAATTCTTCCTTCATTTGCCGTTTATCCATAGGCGGTACATTGTGATATTTATAGTCGTCATAAAAATTGTTTCCAGGATCCTGATTAAAACCAGGAAAATCATAAATTTTTGATATGTTGTATTTATGATTAACAGGATCTATTTCACCCTCATCTTCATCTGGACCTAAAATTTTTGTACCATCTGAATTGAATAAATTTATGCCAGAATGTTCAACTTTAGC

>BPA_5469

ACTCAATAAATTATTAATATCATCTGAATTCAATTGTTGTTGTAGCTGTTGTTGTTCCTGTACGGTTTTTGGTATATCACCCGAACTTAATGAACCAGAATTACTACAAGAACTGCTATTTAATGATGAACATGTTCTTCTACGTTTTCGAGACATCGCCGCATGATTTGGCTTATAACGAAATGATACTGGTGGATAACTTCGTTCATCATCTGAAGGTCTTACCAATTCTAT

>BPA_5488

AAACATTAATAACACAAGTGTCAATTTTAACAATATTGCAAATAATACGGCTTCATTGTCTGTAAAGAATTGGGCCAAAGATCCAGCGGAGAAAAATTTGTCGAATCCACAAAATACAAATACATCATCAACCATAACTACCAACAGTCCAATTGGGATTAATTATAGCATTAACGATTCGAATAATAACAGTGTCAAAG

>BPA_5494

CTGTATCAGGTCCTGTCGTCACTGCCGAGTGCATGGCTGGCTCAGCTATGTACGAATTGGTACGTGTAGGCTACTATGAGTTGGTGGGTGAAATTATTCGTTTGGAAGGTGATATGGCCACTATTCAAGTATACGAAGAAACCTCTGGCGTCACCGTTGGCGATCCCGTCTTGCGTACTGGCAAACCCTTGTCTGTTGAATTGGGTCCC

>BPA_5510

TGGTACTGCGTTGTGACCAATCTCCTTTTTCTGAATTGGCATACGATTTAGGAGTGGGAAAATCCCAGGAAGATTTACGTGACATGCGTTCCCCCGGTTCATCATCATCTTCCCAGGCGCTTCCCGACACAGAAATTGTATTAGGTGTTCGTGGTTCATCTCGAAAACGGGGCGTATTTATACTTCTTTCCGAATCACGATCCCGATCTCGTTCGCTACGATCTCGCCG

>BPA_5511

TTCAAAGTAATTGTACCGGCCCACAACAACATCATCATCATCAAATGATAATAATACAAATACTAATGCAACAACAAATTCATCATCATCCTCCTCTTCAACAGCAAATCCCGCACGTGTTACTACAGCCACTTTGCCAACCACATCGACCCAAACACGTTCTACTTCACGTCCTCAAGTACATTTTGGTAATATACCTGTACTGGGTATGCCAGCCGGCTGGAATGGTCGTGTCCTTCCA

>BPA_5517

GTTATTCTGATGCTGATTGGGGATCGAATGTAAACGATCGTAAGTCGTACAGTGGTTATGTTTTATTTTTCGCAGGTGGGCCGATATCATGGGAGGCGAAAAAGCAAAACATAGTTGCCTTAAGTTAGATGAAGGCCGAATACATTGGCATGTGCCAAGCCGTAAAAGAAATTAGTTTTTATCGTAGTCTGCTAAATGAACTTGCGA

>BPA_5531

GTTTAATTAAATTCTCCAAGTAATATATCAAGAGGCAGTGAATTGCTAGTAGCTTCCACCTGGTTGTCATCATCAAGATTTGTAATCTTGGTCGTAATTGGTCTGATCACCATTTTTAAAATGCTTGGTTTTGTTCCATGCCTGCGAATATATTGAGACTCCATTCGTTTACTTCTCTCTACTGATTGAGGATTTCTTAGGGAGCCC

>BPA_5533

TTCTCTAGCCTTTTTAACCGATTCACTAACAAATGTAGGAGCCTCTGACTTGGCATCATCTTCCACCTCATCAGCTTTTCCCCCAGCGCTTTCAGCTTTCTCAAATGTTTCTTGAGATTTTTGTTCATATTGTGATTTGGGAATTTCATAACCTTCGGGGGCAACATAAAAAGGCAATTTTCCTCTTTGCCAATCATTGAG

>BPA_5542

AGAAAATCTTTGGGAAAACCACAAACTACCGATATGAAATGAGGTCGAGCTTTAGTTACCCCTCCAGAGGCGGTTGCATTTGAAGCTGTTTCAATTAATGTGGAGAAACCATTGCGCAAGGCACGCGAAATAAGTCTCGAAGTCCAACTAAGGGAAGAGTGCATGCTGCGCTTATTTTAGTAATTATTTCTTCTCTTTGTAGTAGAGTATGTATCTG

>BPA_5562

GCCTTATTTAAGCCATTATAATAATATTGATGATAATAAATCGCACTGTGATGGAATGCTGCAATACACAAATGGATTTCAAAATTCACGTATAATAAATGGGGATCATCATAAAGTAGAAAATGGTATGGAAAATGGAATAGATGAAGATTTGGAAGAACATTTAAGTAAATTAAAAGTAAATGATCAAATAAGAAGAAATGATGAGCAACCAATAAGTGCTAGAAGCAGACAAACAATGTGTTCTACTAGTACCAAAAGAAGTGTGCACATATTAGGATCTCGTGATGATGTGCG

>BPA_5568

TCCATATTCAACAATTTCATTTATATCTTTATGGCTATAAGTTTTGCGTGCAGAATATGCTCCCAATTTATAAAGTTCAGGTCTGGCCTTTACTTCCAAAGGAAAACTATGGGAGTCAGTAATGTGCCAATGGAATGTATTAAGTTTTACCATGGCCATGGCATCTAAAGTCCTCTTAATAGATTTTGTACTATAAAAATTTCTTGAAGTATCCAATAACAAACCACGCCATTTAAATGCCGGTTTATCTGTTATAACAGCCTTGGAAACAATTTGCAATTCTCG

>BPA_5581

CAACATTTGCTCCTTTAAGATGTTGCGATTGAGATGCAATACCATTTTGTTGTGCTAGTTGTTGTAAAAATAGTTCTTGTTGCTGTGACGGCATTTGAGGCGTTAGTTGCGTTTGATTAGTAAAAGAGAATTGTCGAAAAGAAAGTATTGCCGGATCCATAGCTGTCCAATCATTCCCAGTATAGCCATTTTGACCAGTACTGCTTTGAATTTGATTACTATTTATTTGACGAACTGGTTCATTCATATGTTGAAAATTTCCTAAATGTGTACCCCATGCATTTC

>BPA_5589

CAATCAGGCATGAGTATGAATAAGCGGGCTTTGGAAATTCTGATTCAATAACAGTAGCTTTTATTTTCTGTTGCTGAGGTTTCACAACAATATTCGTATTTGTTGTAATACCTGGATGGCTGTTGCTGGATAAACTATTCGAATTAAATGTACTTACACCTGCATTACTAACTAAATTTGTGTTATTTATGCTATTATTG

>BPA_5598

GAAGATTGTTGTATTGTTTGTTCGGTGTAAGTTATAAGCCAAGGTGAATACATTTCAATAATTGAACCGAACACTAAATCACAAATTTGATCAGCTAGTAGTTCCTGGTAGGTACATATGGCTGTTACCAATAGGCAATGACCAAAACAGGAAAACCACATGGCCAATGGTTTGCAATATTCTTTGTGTTTAGGATAGAGACCATGTAAACCATGACTCAAACGCTCTTTTTGAAAATGACGAGCA

>BPA_5618

CTCGACAGTAGAGAGCAAAAGTTCTTGGCGGTAAAAGGGTTTACCCAATGGAGGTTGTGTAGGAATTGGAGGTGTAATTTCACTGAGTGTTAATAGCCATGGTCCCCTGAAATAAGATTTATTGTTTTGTTCCCATATCTGGTGGATTTGTGCTATAGACTGTTTGCCCATTTGGGTGGCTACATAAACAAAATCACCCGTCTTTA

>BPA_5628

CTCCATTCATGTGAGACCAAATCGAATTGTCCGTATAACTGACCCATAGTTATGGCTTTGGGATTTATAACGGTGTATATAGCACGATTTTCACCAGCGTTTCTCTTTTCCATGCACTCCAATGTTTCGGCCAAAATTCTATAGCATGTTGTTTTGCCGCCAAAGGGTAAACCCACCAACATTAGGCCATGCCTCACCACAATCATTTCATAAAGCTGTTGCACCTTTTCCAAAAAGAAGTCAGTACACTGTTTA

>BPA_5643

AGACGATCAAATGCATCAGCTACTGGTCCAATTGATAAATTAGCGAAATAGTCTTCGGGAAAACGGCGATTTTGACAAAATGGTGCTGGCATTCTTAAAATGCAGGCAGACATTAGAATTTCAAAGCGTTCAGCGGGTTCATAGAAACGTTTGGTACGTGTTTTCTCCACAAAATCAATTAGACGAAAGAAGGGTGTATACTTTTGTAAATGCATTAAAGGCTCACGATGTGTCC

>BPA_5656

TGAAAGTGTGCGTGAATTTGTTAGTGATGTGATTGGACATCAAGCAGATTTGCGTTTCATTACAATGGCAGCTCAGAAGTTTGTCGATGAAAGCAAAGAGTTCTTAGCAATTTTGAATGATTTTAGAACATCATTACCTGAACGTTTACCACATGTGGAACCTTTATCGAGTGCCGAAAGCCCTATACGTCAAGAAGTTTCTTTAGTATCAGCTCAATATAAA

>BPA_5658

TCTTGGTTTCGAGAAGCTGAAATTTATCAGACTGTCATGTTAAGACATGAAAATATCTTAGGTTTCATAGCAGCCGATAATAAGGATAATGGCACTTGGACTCAACTTTGGTTGGTCACTGATTATCATGAAAATGGTTCTTTATTTGATTATCTAACCTCCCATACGGTTGATACTAAAACAATGCTTAGCATGGCTTTATCGATAGCCAC

>BPA_5667

GTAAGCGAACATGGTTCTATGTATATGGCTCTAGATGTTATTAAACTGATGAAGCAAATGAAAATACCAGAAGCATCTGTAATGGGTCATAGTATGGGAGGGAGAACAATGATGTACTTAGCGTTAAAATATCCAGAATATGTAAGAGATGGCATAATCGTTGATATTTCTCCCCTTTCTATTCCTCGAGACTTTTTCCAAATGGAACAGATTTTTCTTGCAATGAAAAATTTGAATATAC

>BPA_5673

GATCTTAAAGATATGGAATCTGTAGATACAGAATATTATAATTCTTTAATGTGGATAAAAGAGAATGATCCACGCCAGTTAGAGCTAACATTCTGTGTAGATGAAGACACTTTTGGTCAAAAGAGTCAGCATGAACTAAAGGGTGAAGGAGCTAATATAGAAGTTACCAATGAGAACAAAGATGAATACATCAAACTCGTCATTGAATGGCGTTTTGTAGCGCGCGTTAAAGAACAAATGACTGCATTTTTGGATGGTTTCGGATAGCGCGCGT

>BPA_5710

ACCCTTTTGACCGATGGCAGGACAGAAATTTTCTTCGGTTATTAAGTTACGATCACAGCCACGGGCAAGCCAATCTTGTCTTTTACGATCTGTACCGGTGGAACATTTGTTTAAAGTGGGACACCATGTACAATTAAATGCAGTGACATGATTTACACATGAGGCACAATCGGTATATTCAAGACAAGTGGGTAAAGCTTTCAAAGTGATGATTGTAGAATTTGTAATCTCGGCATTGGCAAAATTAAC

>BPA_5729

AACGAACACGACCATTGGGATTTGTCAAACGGATCGACAATTGCTTAGCGCGCTCAACGATTTCCTTGCGTTTCTTTGAGGAGACACCGTGAGCAATTTCACCGCAGTAGACACGGTTTTGCATCATTAAAACTTCGAGCTCCTTAACGTTGTGTACAACGAACTTCTTAAAGCCAGTGGGAAGCATGTGACGGGTACGTTTGTTCGAACCATAACCAATGTTGGGCA

>BPA_5773

ACAAATTTTACCAGATGAAATTTGCTCTTGTTTTCTTTTAAGTGTTCTCAGTATAAGTCAAAATAAAATCTCGAAATTACCCATCAATATGGGAAATCTAACAAATCTCAAAGTTTTAAACATAGTTCAAAATAATATTTCTACATTACCAGTAACACTTTTAAGCCTTAGAAATTTGGCATCTCTATGGATTTGCGATAATCAATCTAAACCTTTAATACCATTA

>BPA_5776

ACCAACATCAAATCCATGTACGAGAATGAATTGGGTGATGCTCGGCGTTTGTTGGACGATACGGCGCGTGAAAAAGCAAAATTGGAAATTGAAATCAAAAGACTTTGGGAAGAAAATGAAGAATTGAAGGCCAAATTGGACAAGAAAACTAAAGAGTGTTCAATAGCTGAAGGAAATGCCCGTATGTATGAATCACGTGCAGCTGATTTAAGCGCCAAATTCAATGCTGCCAATGCTG

>BPA_5787

CCAGTACCACCACCCAAAGAATGAGTCAATTGGAAACCCTGTAAACAATCACAATTTTCACATTCTTTGCGCACCACATCCAAAACATTGTCCACCAATTCGGCACCCTCGGTGTAATGACCCTTGGCCCAGTTATTGCCAGCACCGGATTGACCAAAAACAAAATTATCAGGACGGAAAAGCTGGCCATAGGGACCAGAACGCACAGCCTCCATGGTACCGGGTTCCAAATCCAATA

>BPA_5801

ACCACAAGAGACCAGAGTGGGTTTCAATCAATTTCGCAATTTATATCATTGTCCACATCGGCGTTTGATACATATTACACAGGAGGTGTTTGAAATGAAACCAAATGATTTAGTTGCCCTGTCAATGGTGACCTACTACTTCATTTTGGGTTCCCACATGTTAAGTTATGAATTGAGAATTTCGGATACCCGTATTATTAAATTAAAT

>BPA_5812

TCAATTCGTCCATTGGATGTTGAGACTATATTCAATTCCATTAAAAAGACTCACCACCTAGTTACAGTAGAACAAGGCTGGCCTCAAAGCGGTGTTGGCGCTGAAATCTGTGCTCGAGTTATGGAGGACGAGACTTTCTTCCATCTGGATGCCCCCGTGTGGCGTGTATGTGGTGTTGATGTACCTATGCCATATGCTAAGAATCTAGAAGCCAA

>BPA_5823

GCGCCCATAATCAAAATCAGAATCTTCATCTAAAATTTCATCTACTGCGGAACCGGAAAGTTTATCGAATGTTTTCTTCAATTGCATTTTTATATGTTTCCTGGTTATTTCTTGTTTGGCATCTACAGCCGTAAAAACATCCGTAAGGAAACCTAATGCTGATCGGGCATCCCTTTTCTGTGTTACATAGTTAAAGGCACATACTAAAGACCTATAATCTTCTTGG

>BPA_5851

AAGGTAAACCTCTTAATAAAGCGGCCACAGCCTCCATGCAAGCTTGATCTAAATCTCTAAAAATCAATGAAGTGTTTGTTATTACAGCTGGATCTGTGGAACTAGGTTGTGCTATCTGATGCGATGTACCCATTACCCAATCTGACAAATACTCCACCAATTTATTGCGAAATTTCATTTCTTGTCTAAATGCCAAATCGTCT

>BPA_5884

CGACCAATCGCACAGACAGAGGACCCAATTGGACACGTTCGTAATGGAAATGGGCACATTCTTCAAGTTTGGGAAAACGACACGTTTTCCCAGCTACACTTTGAGCCACCACCGGTTTTCCGGCACCAGCCTTAACCAAAGCCATACTATTTCCGCTTAATGAACCAACATGTGTTGATGCCGTTTGTTGTTGACTCTGTGATTGTGTTGTTGATGTATTTCCTGCCGTCTGTTGATGTTG

>BPA_5891

TAGGCATGGACCATGGTTCCACATATCATGCTGGTGGCGTTGGTGCTGGTGCTGGTGGTGCTGATAGCGCTCATCATAATGTTGGGGGACCTGTTACTACAGCCTTTGAGGATAGCCAATGTGATCATGAGGGTTATTTCCATTGTCGCAATGGTGAAGTGATCGATTGTGCTGAACGTTGTGACAATAAGGCCGATTGTGAAGATGACAGTGATGAAGATGAGGAAATGTGTCGTAAATTCTTTGAGAATGAACCAGAAGAGGAAGATTTGGAAGTTGAAGAAGATTTGGATGGAGATGTAAATGGTCATGATCAAGTTCATGGTCAAGGACACGATCTCGATCAGGGTCATCACTATGTTGATCCAGACACTAATGGTTACGATCCTTATGATTATGATCGTGAACAAGAAATGGAACGTGAAAGACAATTGGAGATTCAAAGAGAACGTGAACGTCAAATTGAATTGGAACGTGAGAGACAAAGACAACATGAATTGGAAAGAGAAAGAGAAGAACAAGAACGTGCTCGTCAATATGAAGAGGAACAAAGAGCACGTGAACAATATGAACGTGAGCGTGCGGAAGAAGAAAAACGTGCTCACGAACAAGAACATGGTTATGATCATGAATCGCAACCTCATGAGGGTGAAGAGGATTCCTATGGTCATGAATATCAACCCGAATCACAACCTGGTTATGG

>BPA_5892

AATTTGCAAGTCATTGGGTACGAAACCAGCATCGACGGCAGCACGGGAAGCGCCAACAGCAGCACCGAATTTGTCAGCCAAATCGTACAACAATTTGAAGTTATCACCCGATTTCAAGCCACGGCCACCGGAGATAATTACCTTGGCACTGGTCAATTCAGGACGATCAGATTTGGTCAACTCTTGGCTAACGAATTCAGACAAATTTTGTGCATAATCACCAGCGGGAGCCTGTTCAATGGCGCCATTACCACCGGTGGCAGCAGCAGGAGCAAAGTTGGTAGCACGTACTGTAATCACCTTAACGGGATCCTTGGAACGTAAAGTCATAATAGCATTACCAGCATAAATGGTGCGCACAAAGGTGTCCTCACTCTTGACATCAATAATTTCCGAAATGGGTGAAACATCCAATTTAGCAGCCACACGGGGTAAAACATTTTTGCCAAAAGCAGTAGCACCAGCCAAAATGTGAGTAAATTTGAATTGTGAATTGATTTTGAGCAGCCAATACCAAAGGAGTCAAAGATTCAGCAGTGAAACCTTTGAAGGCATCATTTTCAGCTACCAAAACTTT

>BPA_5895

AATGAAGTTAGCAAATTTATTCAGTCAAATAAATTTGAATACGTTGAAGATGAAAAAAGGCAACCAGATATTGACAGCAAAGAGAAAGAAAAACGAATTAAACGCGTTGTAGATCATATAGATCAATTGTTAAAGGAAGGCGCTAATGCTGATAGTATCATAGATTACATCAATGGAAACATTGTTAAAGCAGACAAGACATTTATTAAAAATTTAACAACGATATTAACAAGCTTTGCTATAAAAGATCCGAAACTTAGTTTAGATATACCGTGCTTCCAAAAGATATGTATACCAGTACTGCTACGCTACCTCGACAATAAGGAGGATCTTGAATTGGAATGCTTATATGCAATACAATTATTAGTTCATAGCCTGGAACATCCAAGAGGTTTATTGGGAGCTATATTTGCAGAACTGAATGATGCCGATGTTATACCTTCGGAATCTTTTATAGCGTGGAGAGATTCTAA

>BPA_5910

AGCATCTATGGTTATATCGATATCTGTGGCATCCGCAGAACTTCTTAAACTTTCTTGACTCATATGCTCGCTGGAATGTTTCCTGGCAGGCTCTCCTGAATTCCAGGCTTTCTCATATATAGCCACCTTTTGCTTTAAACGCAAGGGAGTGGTAGGAGTTTTAGGTCTCGCCAATGATTCATAACCCTCGCTCATCAAATACTCATGCGAACTGGTGGTAGTGGTCTTTATAGTTTTCGTTATGGTTCTGGAAATGGTTGAGGTTTCCTGGATAGATTCATCAAACATTACATCTTCCATGATCTCAAATTCCGGCTGCCTTTGGCCTTGGCTAACATATGGTTGTGTGTCATCTGTACTAAACGATGACGATTCAACAGCTGTTTGCACTAAACGACGTTTGCGCACTGCCGGTATTGGCCTCTTACCACCACCATCATCCATCCCCATGGGTTGTGGTGGCGGGTCTTCTTCATGTCTCTCATCTAAGGTTTTATCCATGGTCGTCTAGCATTTACTCCACGCGTTTTGTTTGTTCAAACAAATTCTCACACACTTC

>BPA_5916

GAAAAAGTGAATATCTTCCAAATATTGACACCACAAACGGAAGGCTTCAGTACTATTCTGAGTTCTAACCAAACGAGCCGACAAACGATGATATTCATGGAAAGTATCTTGGAAGATTTTCTGCCATAATTCCAATACTTCAATTATAGAATTTTCATCTCGGGTAGGTAAATTCCAATACTTCAATTATAGAAGATTCATCTCGGGTGGGTAATTCCTCAGTGGCAGATTGAATATCACGTTCTCTTAATTGTTTCAGTTGTTCCTCGGTATTGGCCAGTGTTTGTAAATGATTTCTTAAAACCGATAATTGTTCTTCACATTCTTTGCGTGAACGAGATTCCTGGGAAATGTCCTGACGAACATTATTGAAGTATTCCTTGGTTTCGGTACAGTTTTGTTCCACCTCTTTGCGTTCACGTTCTTGGGCCTCAGCCACATGAGCCAATGTGGTAATGGTTTCTTTACAATTCTCCAAACGTTGACCCAATTCCTCTAATTCGCCTTGCATAAAACTGGAGGCATCTAAACCGATATCGGAGGCAATTTTTGTAACAATATCACGTAACTCTAACAGTTTATCATCTTGACTATTTAAATTATTTTGGCAAGTCTTGGCATGTTGTAGTGTATCCAAGGGCTTATTGAAGGTCTTAACGGTATCCATTTGTTGTTCAATGTAGTTTTTAACTTGGGCTGATGCGTTACGATATTTGTGCCAAAGTTCTACAGCACGTTGATAACGTGTACACAAATTTTGCAAATTATCGGAGGTATCATTCCAGGCCACTACAGCTTCTTGAACAGCCGATTCGATTTGTTCAACAATTTGTAAATCACAAGTTTCGATTAATGGTTTTGCAGACGTCTGTAAATCGTCTAACAAAGTCTCGCGATTTTGTAAATCACCAGCGAGTCCCTCTAGACGTTCAGTGGCCTTACGGAGTCGTTCATAATCGACTTGTCCATGTACCTTCAATAGCTCCATCATAAAGTCGGTTTCTTGCACGGAATTGTGTACTAACTCTAATTGTTTCTCATAACGCATCCATAGAGCCAAGCGATTACGCAATATTGTCATAATATTGGTACATTTGCTCTCGAGGAACGCATGATCATCTTGCAGTTGAGATATATCACGTTCGAGTTTTACCGGCGAAACAGACATTTTCAAACGCATTGGTAAAGTGTGTAACATTTGACGGGTGCGCATAATTTCCTGCCACGCCCCTATCAATTCCTCACCAACAGTTTGACAATCTACAACAGCCTTTTCAATAGCATCACTATCCAATTTCTCGGCATTTTCACATTCATCCAAGATAACG

>BPA_5917

AAATACTATGATGATTGTGCTAAAATCTTGGGTAACATGATATTAAGAGAATTAACACCGACCTTGCATATACCCTGGTCAGTGCCCTTTCAATTGGATGATCCACCTATAGTGCCCACTACGTTTGGCAAAATGATGGAATTACGTTGTGTAGTGGCTGTTATAAGACATGGTGATCGTACGCCTAAACAAAAAATGAAAGTTGAAGTTAGACATCCTAAATTCTTTGAAATTTTTGAAAAATATGATGGCTTTAAACATGGTCATGTGAAATTGAAACGTCCCAAACAATTGCAAGAAATTTTAGATATTGCTCGTTTTCTGTTGGCCGAAATTGAAACCAAAGATAATCCCGAAATTGAAGAGAAAAAGAGTAAATTAGAACAATTGAAAAGTGTTCTAGAAATGTATGGACACTTTTCTGGCATAAATCGTAAAGTACAAATGAAATATCAACCTAAAGGTCGTCCACGTGGTTCCAGTTCTGATGACGGTAATAATGTTAAGACTTTAGACGCTCCGACAATTAAGACTTACGCTCCGACAGAACCTTCTTTAGTACTTATATTAAAATGGGGTGGAGAATTGACCCCAGCTGGTCGTATACAAGCCGAAGAATTAGGCAGAATATTTAGATGTATGTATCCGGGAGGACAAGGACGTCAAGATTATTCAGGTACCCAAGGTTTGGGTTTATTAAGATTACACTCCACTTTTCGTCATGATTTAAAAATCTATGCATCTGATGAGGGTAGAGTACAAATGACAGCAGCTGCATTTGCCAAAGGTTTATTAGCTTTAGAAGGTGAATTGACACCCATTTTAG

>BPA_5921

AATTATTACCAGATCTAGCTTGAATCTGAATATGTTGTTGCTGTTGTGTGTGCGGAGATGTAGTAGATTGTTGATGAGTTGATGAAAGTGTCGTTTGTTGACCTAAAATGACCGTTTGTGTTTGTTGTGGGGTCGTTGTTTGAGATGCTTGAGAAGCAGTAATTTGGTTTAAGATGCGTTGATGTTGTTGCTGTTGGGTCTGATGTTGTGAGGATGCTTTAATTGTTTTCAACTGCAATTTTTGAGTCGACATTACAATAGGTGTGGCAGTTAAATTTGCTGAAGAGGTAAGATTTGTATTAGTATTGGCATTAATAGGCGCTGCAGCAGCTATTTTTAAAGTGGATCCACCAATTTGTCTTTGAGTTATCAACTGACGACTACCGGTTAGTTGCTTTGTTCCAACAACTTGTATATTTCGACCACCTTGCTGTATAGTACCCACTAACTGTATAGCCTGATTAGTTTGATTTCCTGTTCCCGTACTACTAACTGTTATTGTAGCTTGTTGAGGTTGGTGAGAAACCTTTTGTTGTTGTTGAACTCCGTGTTGCTGCTGAACGATAATGCTACCACCAGTCGCAGTGTTCGCATTCGTTGTTGTAACACCTACAGATGAGGGCCCGGTGCCCGATGTCCTTAAAGTTGCCGAATTTAACTTTTGTACGCCCGATCCCACCAATTGAGCAATAGGTAACGTTAGCATTGTGGTGCCCGTATTAGTAGTATTGCTGGTATTATTTGTACCTGTTGAATTTCCAACACCTGTAACTGTATTATTAGTTAAAACAACTGTTTGCGTGCCGCCAGTCGTAGTAGCAACCGCTCCTGCAGGTATGTTGACAACGGTACTAGTTGCTGATGAAGCATGATGTTGTTGCAATTGTTGTGGCTGTAGTGTAATCGTATGCTGAGCTTGTCCTTGTTGTTGATTTGCCGAACTTACTGGAACGGATACCAAAAGACTTCTTTGTTGATGTGTCTGTTGCTGAGAATTTGTGTTTTGTGCCGTAACAACAGCACTGCTGCTATTACTTGAATTTGCACTGCCACCAGCAGTCACCACTGAGACGGTTGAATTCCCAGATTGTTGAGGTTGCTGCACAGCGTGTAAGCTACCACTCGTGAGTTGAAGTGATATAGGTTGATTGAATCCTGGTATTTGAACTTGTACATTTTGCAAACCAGGAAAATTTGCCATAGCTCCCTGCAATGTGGAAAAATTAATTCCTTGAAGATTGAGCGTCGCAGTTTGGCTTTGTTGTTGCTGCTGTTGCTGAGGCTGCTGTTGCTGAGGCTGCTGTTGCTGAGGCTGCTGTTGCTGAGGCTGCTGTTGCTGCTGTTGTAGAGGTGGCAAATGGGGAGAGGGTGACGTCTGTGGATGCGGACTAGACATTGGCGAATGTATGGATGATGTGGCGGGAGATTGTACTAAAGACTTTGTAATATACTGCTGTTGCGGTGTAGATGGATGGTGTTGTTGTTGGGGTGACATATTGGGAAGCCCAGTTCCAGATGCTATACCACCACTATTAAGTCGCTCCAGTAGGTTTTGATTTTGTGAAGACTGCGAGTGCTCGGAACCGGATGGTGAAGGTGTTGCAGCGTTTGCTAACAATGCGTTTAAGGTTGTAGAGTTGTTACTATTAACGCTCGTACTCGATGTGGCTTGGGGGACTGCGA

>BPA_5924

ATCACGAAGAAGTCGTAGGAGAAGTAATTTTTTTACTACAAAAGGAAAACCAAAACTGCTTTAGACACACAAATCCCTTAAAAAAGAAAACAAAAAAGAAACTATAACCTAAAAAAAAAACAACCAAAACAGATTATTTAAATAAAAAATGGCCGATAAAAAGAATGAATATTATGCGCCACCACCTAAAATGGGCAAATGGCAGGGCTTCAAAACATTTCTGTGGAACAGTGAAACTAGTCAATGTCTGGGTCGAACTGGTGGAAGTTGGGCAAAAATTCTGATATTTTATGTAATATTTTATGCGGCACTAACTGGATTCTTTGCTGCCATTTTTGCTGTATTTTATCAAACATTGGAAATAAATAAACCAAAATGGACATTAGGCGATGGTTTAATCGGGACAAATCCAGGTCTTGGTTTTCGACCAATGCCTCCAGAGGTAAATGTTGAGAGTACATTAGTTTGGTATGAATCAAATAAACCTGAAAATTTTAAATATTGGGTAGATGAAACTTCCAGTTTCTTGAAATCCTATGAAGATTTACCCAAAAAGAATCAAGTAAACTGCTCTTTTGAAAATCCACCACCCGAGGGCAAAGTATGCGGCATTGATCGTAATCAATTTGCACCATGCACAGAAGAAAATCATTTTGGTTACAACCTTGCCAGACCCTGCATATTCCTAAAGTTGAATAAGATTTACAATTGGGTTCCTCAAATCTACAACGATTCAAAGAATTTGCCCGAAGAAATGCCCAATGAGTTGAAACGACACATCACAGAGAAACACAGCTTAAGACCAAATGAGACCAATGTTGTTTGGATCTCTTGCGAAGGTGAAAATCCCGCTGATGTTGAAAATATTAAAGCCCGTGACTATTATCCTCGTATGGGTTTCCCATCCTACTATTTCCCTTTCAAAAATATTGAGGGTTATATACCACCTATTGTAGCTGTGCAATTTACCGTTGAAACTGGTGTCTTAATCAATATTGAATGCAAAGCTTGGGCTCGTAATATCCATCATGATCGTTCAGAAAGGAGAGGATCCGTCCACTTCGAGTTGATGGTTGATTAAGATCAATCTTTAGAAGATTTAGAAAAAGAGCAGCAACAACAGCAGCAGCAACAAAACCTAACACAACAGCATCAAGAGCAAGAACAACAGCCACAACACCAGAAGAACAGTGAATTAGTAAATAAAACAAAT

>BPA_5928

GGATAAAAGAGCATTATACGATGAACAGGGTATTATTGATGATGACGATGATAATCTAGAGGGTAAATTGAATAATTGGTTGGAATTGTGGCGTAAACTATTTAAACCTTTGACTGAAGAGGATATAAATAATTACGAAAAATCCTATGTGGGATCAGACCTGGAAAAGACCGACATAAAGAAAGCCTATTTGGGTGGCAAAGGATGTATTAATTATATGATTAATTGTGTACCTTTTATGAAAGTAGAAGATGAACCACGTATACAGGAAATTGTTAAGAAAATGATAGCGGACGGTGAAGTGCCCGAATATAAAACCTTTACCGAAGAGCCTGAACAGAAACGTAAGAAACGTTATAAGAAGTATGCCAGAGAATCGAAGGAGGCTGAAGAAATTAAGGCTAAAATTCAAGCAAGAAATAAAAAGCGTGCTGATGCGGCCGGGCCAACAGCGGGTGGAGGTAGTCTAGAACAAGCAATTATGTCTAGACAACAAACACGTGAAAGTGGTTTTAACACTCTAATGGATAAACTAATGGAAAAGTATGGTAATGAGGATGACGAAAATGATACTTTAGATTTCTCAGCCTACGACAAAGTCAATAAAAACAAGAAGAAGGGTCAAAAGAAAGGAAACAACAA

>BPA_5931

AATCCACTAGTATTCTGTTTTAATAGGGTCACTTGTATTGCGGCTGGATTGTGACATGGTTGATATAACGAAAATTTTAAATCAATATGTCCCAAAGATGCAGGTTGCAACAAGTTTATTTCAATGATGTGTTCATCCCATGTTGTAGTATCATTGTGTAAGCGCCATGTGTGCTGTGTTTGCTTACGTTGCTTTTGGGCTTGTACTAAATTATTCCAACAGCTGGGAACTTCTGCTGTATAAGGTATCGATTTTTCATCGAAAAGAGTAAGTGCATATAATGATCTTAAATCATCTAATGACAGATCTCCATTCCGACAAGCCAGTAAATTTGAAACTCCAGCGTTACCGGACATTGAAGATGGGGATGGCGATGGAGGAACAAAAGATCCAAAACCTGTCAATTGATTGGGGCCACAGTCGACTTTTATATTTGAACATATAGGATTTTGTACAAAATTCAATTTCAATGAATCATCATTGGTTTCGGACAGAACATTACCTTCATGATGTTTTGGTTGAAGGTTTGCATTAGCACCATCTGTTACTGAAAATTCAACTAAGTTATTATTTAAAACAACGCTTGCACAACTCTCTTCTTCAATATCAATCATTTCATCTCCAGATTCATCCGTATCATTTAGAGAATAAAAAATCAGTGTTCCTTCTTGAGTGCAACAACATAATCGTTCTAAACTGCGACAATATGTAACAGC

>BPA_5944

ATTATGCTGAACGTGTTGGTGCCGGTGCTCCCGTGTATTTAGCAGCTGTTATGGAATATTTAGCTGCTGAAGTTCTTGAGTTGGCTGGTAATGCTGCTCGTGATAACAAAAAGACAAGAATCATCCCTCGTCATTTGCAACTTGCTATCCGTAATGACGAAGAATTAAACAAATTATTATCTGGCGTTACCATTGCTCAAGGTGGTGTTTTGC

>BPA_5947

GCTGCATCGGACCAGCAGGTGCAGTTTACTGGCACCTCTAAAGCACATTTCTTGGATAGTGGTGGCAATTCTGCAATCTCTTGCGAAATTTCGCGACACTCAGGACTAATGCTGGTATTGCCATAATCTATAAACAAAACCTCATAACCCTCAGGAACTTGTCTCAACAATTTGGCACGATACCACATTTCATCATCGGGAAATAGGGCAGCCACTATTTT

>BPA_5958

CCGCAAAGAACGTTCAAAACGTTCACACCGTAAGTCTCCTACATCGGGCCGTCGTGTCCATAAATATCGTTACAGAGATGATGCCTCCCATTCAAGTTCTCGTCGCCGGCATCGTGACCGTGCCAAAGATGAACGTGATAGTGGACGTAATAATAGGAGATCACATTCTAAGACAGCAAAGCCCGTCATACAAGATGATGCTGATGGCCACTTGATTTATCACGCTGGCGATATACTTCATCACAGATA

>BPA_5993

AAAGTAATAATACCGGACAATTTAGAGAATCAAAAATTTCAGAAACGTACGAAGCATTTACATCCAGCCAAGGACAGCTTTTGGTGGGCGAACAAAAATTCATCATACGGATTTTAAAAATGAATGGCTCTACGATGGTATTCCTAAGTTCAGCTGAACCAGAGTGTTTAGATGAAATCGCATTATCAATGAAAATGCCACAGGATAACCAAGTTGTTGGCACATTTGTATTGGGTTCCCAAATTATAACGGACTCTCAGCGTATGGCAGAAAAATTGTGTAAACGTTTTGGACGTCATTTTATCG

>BPA_6015

GAATAACAGCTGTCGAACGTGAAGACCGGAAAAAAATTGAAAAGCGTGAAAAACGTGAACGAGATGATGAAGAAGAACGTAATCGGTTAGCATTTAATTACATAAAATACACACTTGGTCTAAAGCATCAGGTGTGGAAGCAGAAGGGAGAAGAGTATCGTGTTCATGGTCAATGGGGATGGATGTGGTTATCATCGAGT

>BPA_6040

CTCGTGGATTATGCAATGGGCCAATAAGGGTGGTGTTGATGTTGTTGTACACACTGGCGATCCCATGGCTCTAGTTAATTTAGCTACAAATCTACCACTCTGTCATAACTTACATATCGATCATGCCGAAGAAAAATTGAAACATCAACGTTCAGCCTTAAGTCAAAGTTTACGTTCATTTGGAGCATTTAATGAAGCCATATGTCAACATGAAGCCGCGGAATTGGC

>BPA_6050

GTTATCGCAAACAGGTTGAAGTCGATGGTCAACAATGTATGTTAGAGATTTTAGATACTGCCGGTACCGAACAATTTACCGCGATGCGTGATCTTTATATGAAAAATGGTCAAGGTTTCGTTTTAGTCTATTCGATTACTGCACAATCTACGTTTAACGATTTGCAAGATCTAAGAGAGCAAATTTTGCGTGTTAAGGACACAGATGATGTACCCATGGTTTTGGTGGGCAACAAATGCGATTTGGAAGATGAA

>BPA_6067

CCATATTCACCAGGTGGGAGTTCTGATGATGATGTACCATTATTACCAGTTAAGTTACCAAATACTGTGGACCATATGCAGGCAGATGAAGATGAACTGAAAAGAAAAATGGAAGAAATTAATAGACAAATTGCTGCCCAAGAAATGGAAATTGCGGGACTTTTAACGGGAGAACCTGCGGCTTTTGGTTCGGCTACTGCATCTACATCAAATGTGTTGGCAAATATTTCAATACCTTCAAATTTGTCTCAAATTCTTGCTAGTATAAAAACAACTGGAAAGGATCATTCTGTTGTTCCTCCACCACCGCCCTTAATTGGTTCTCTACTGCCAGCAACATCAACGGCTACAGTAA

>BPA_6082

GTGGCAAAGTAATCACCTCGGCCATGCCAAGTTACTTGACGTATTGGTTTGAAGTGTGTTATTACAACGCGAACACCTTTCTCTTGTTCGGCAGCATTTGCTGAAGACCACTGTACTGCCGTTTTAATTCGGTCACTTTCCAGAGCATCATTTTGAGGGATTTCAGCTAGAAGATCATCAGTCTTCTTTACTAGCATTTT

>BPA_6084

AATATATATTTAAAGCATGAAGTCATACGTAGTGTTTGCGACAACCATATTATTGTTCTATGGCTGTCAGGGACAATATAAAAATCTTGGTATTAAAACCCGTTATCCGCCACCACCACCCAGTTCGGGTAATTTGATATTGGGTAATGCAACCGAACATAATGAAAACACACATTATGTTCATACACAACATCAATATGAAAATCCTCAATCGCAACCCAACACCCACGATCGTTATAATGGTAATTTTAAACCAGCCGAATATTATCCAGGTGGTCCATTACCCACGGTAACAAGGCCTCCTGTGATTTTAGATCCCACTGCTGAATTTATCAATAAAACCAGAGCAGCCATGGCCTCGGGTGTTTGCTATAAAGAAGTACCCACTGCCTCTCTGGTCCATGGCTCTGGCGTTATTCCCGTTGGCAACGGCACTAAACCCG

>BPA_6085

GAGGAGGAATTTATACGCAATCAAGAGCGTCTTAAGCCACAGGATGAGAAAAATGAAGAAGAGCGTTCTAAGGTAGATGATTTGAGAGGCACACCCATGTCGGTGGGTAATCTAGAAGAAATCATTGACGATAATCATGCCATAGTGTCAACTAGTGTAGGTTCTGAACATTATGTTAGCATATTGTCCTTTGTGGACAAAGATCAGCTCGAGCCTGGTTGCTCAGTGTTATTGAATCACAAAGTTCATGCTGTAGTTGGTGTTCTTAGTGATGACACAGATCCAATGGTTACTGTCATGAAATTGGAAAAAGCTCCACAGGAAACCTATGCTGATATTGGAGGCTTGGATACTCAAATTCAGGAAATAAAAGAATCTGTGGAATTACCTTTGACCCACCCTGAGTATTATGAAGAGATGGGTATAAAACCTCCCAAGGGTGT

>BPA_6089

GAAGGATGGAGAATTTCTTTCGCCCGAATACATGGATGAACCTATGGAATCTGCTAGTTTTGGAGGCTCTTGTGATTATCGTCATTTGTCTATAGACTCTTCAAAAGATAGTGGTATATTAGGCGATGCTTCCAATTCAGCTTTGACATCTGCATTTAATAATTTTGTAGATACGGGTGATATGAAAAAATCCCTCAACATGCCCGCATCTACAACTCAGCCCATACCGAAC

>BPA_6102

TGGCTTCTTCAACGGGGTGGTCGACAACTTCATCCATTTTCGGTGGTGGCAGAAGTTCTTTAGCGGCAGCTTCTTCTTTTTCAGCTTCCTCAGGATCACGATAGAAGAACAAATCAACTACAACGGGCCATTCAACGCTTCGGGAGATTGTGCCTCGTAGACGGAGAACTTCTCGAGACAACAACCACCACATTAAACCAATGGAGTGAGGAGATTTGTTATTGCATGGAATGGCAATATCAATATAACGAAGAGGGGAATCGGTATTGGTGAATGCAATAACGGGAATATTGACATATGAAGCTTCCATAATAGGTTGGTGATCAGTCAAGGGATCGGTAACAACCAACAAACGGGGCTCACGAAAAGCAGATTGGATCTGATTGGTGAAAGCACCAGGAGTAAAACGACCAGCAATGGGTGTAGTGTCTGTGTACTTGGCGAATTTTAACACAGCACGTTGTCCAATAGGACGGGAAGAGATAACGAAAACATCTTGAGGATGTTCAATAGCAGCAATAGCACGTGCAGCTAAAACCAACTTTTCCCATGTTTTACCCAAATTGATGATATTGATACCATCAGGGCGGCGTTTGTAAACGTACTGTTCCATCTGGAAGTTAACATTTTCAGAACCCAAATGGGTG

>BPA_6110

CTTTAATAGCCTGCTGGGTGGTTTTGCGTTCAGCCAATGATTGCATAAACAAAGTAATAAGCGCTTCGAATTTTTCACGGCATTCATTTTCCTCGGGTAAATGTTCACTTATAGTCCAATTCAATTCCAAAGCTAATTGTTCCTCTTCCTCAGCATTTTCTTCTTGTTCTGGAGCTCGTGGCACCTCCTCTACATCATTGTCTGAATCTTCATCATCAGAATTTTGAGATTCACTGTTTTTTAAACGATCTTCA

>BPA_6119

AAAACCAATTCTTGTCAGTCCAAAATTTAAGAACGTTTCAAAAATTGATGCAAGAATTGTGGCAGATGTTCAGAGTAAAGGACTAGCATCAAGAATTGTGAAATATCATGAACGTGGAACAAGATTGAATCGAAGTATGTCGGCCCATAATCTATATAGCAAACCTGGACAATTTCCTGCGGTTACACTAAAAAAAACCGAA

>BPA_6122

TTGCATCCACCCAGGGGATCTCCAATGGCAGCATCAAAGCCAGCAGAAACTAAAACTAATTCTGGATCAAATTCATATGCAATGGGCATTATGATGTGCTGGAAAGCCATTACATACTCCAAATCACCCATGCCTTTCTTATTCCAGGGAATGTTAACATTAAATCCAACACCAGCTCCTTTACCTACAGCATCGAAATCAGCATCTTTGCTCTTCG

>BPA_6123

AGCTCTTTCAATGGTTTACGAATGCTTCCTAAAAGAAGAGCGGAATTGCCCACATTAGAATCTGATTCTGAAGAAGAGGATTCCGTAAAAAAGGACAACCTTAATTGTGCTATACTTAATGATTCATTTCTGGTAAGTCCAGGAAAAAGCGATAGAGACGATATCAGTTCAAAAGAATCCTCCGCGGTTAAGGGCAATTCGAATAATTCAACTAATGATATGTCATTA

>BPA_6126

AAAATACCTTAACTACCGGCAGTGGAAGTGATGAAGAAAATTCGGTGATTTTAAGGGCAACAACCATAACAGTGGGGGAAGATCAACGCATATCGGGTAATGTGCAAATTTTAAAGAAAAGTTTTGAGGCGAAGGCGGGCAGTGTAACTGTGGCGGCACAAGGTCAAATGCAAACTACAAATAATCTGGGAAATTCAAGTAATAAACCTAAC

>BPA_6134

GTTTGAGTTTCTGTTTCCGTAGGAATGTTTGGATATTCTGAACTTGTTGTAGGTAAAGTTGCTGTTATGTCCGGTTGTGTATTAGCTGTTTCATTTTCATTGTTACTTAATAATTCTGGGTTAAAATTTGTATTAATTAATCTAGTCTCTGTATTAGTTTCGGGGGTTTGTGTCATTTCCTCTGAATTAACTTCTTTATGTTGAGAATCTTGTCCAATCAATT

>BPA_6155

CTGGTAATCTTTGTGATCAATTGCCCTGTGATGCCCGTTGTTCTGAACATGGTCAATGTAAAAATGGAACATGTGTTTGTTCTCAAGGGTGGAATGGCAGACATTGTACATTGCCTGGTTGTGAAAATGGTTGCTCACGTCATGGTCAATGCACTTTGGAAAATGGCGAATATCGTTGTGATTGTATTGAAGGTTGGGCTGGTACAGAT

>BPA_6160

CGTACAGGCTTACATAACAGCATTACAGTACAATCCTGATTTGTACTGTGTTCGCAGTGATTTGGGAAATCTTTTAAAGGCTTTGGGTCGTCTTGAAGAGGCTAAAGCCTGTTATTTAAAAGCCATTGAGACTTGTCCTAGTTTTGCGGTGGCATGGAGTAATTTGGGTTGTGTATTTAATGCCCAAGGTGAAATATGGTTGGCTATTCATCATTTCGAAAAGGC

>BPA_6172

GTTCCACTGGTACAATTGTTGATTTTTTTCCCGCCAATGTTGTCTCATCTTTGTTAATGTTGTTGCCCTTAACATTGCCAACTTCACGAAAAGCCACTTTATTATGAGCGGCTGTTAAATGGACACGGGTATTGGCATTTGAATTAAGTACGGCAAAACTAGCGCGTCCTTGTTTTGAAGAGACGCCAACACCGCCACCTAAGCTGGTACCCAGTGGCTGTAAAA

>BPA_6185

AAATCCCTTACCAGATGGTTATATTAAATTTGAAGATTCGAAAGTTCTATTGGGACCTAAAGTAGAACGTAACGAATGGGGCGATTTGGTAATACACTATTGGCCCATACTTTTGATAGTTTTATTGGTTGGAGTCTTAGTTGCATCTATGCCTATCATTGGATTATGTTTCTGTTGTTGCCGCTGTGCTGGTGCTTGTG

>BPA_6238

GATTTTTCTTTAGCTAAACGACGATTTTGTGTACGTTGTTGTTGTACTCGATTGGCCAGATTTTGTAATTCTTTGGTTATGAGATCCACCTCTTGAGTTTTTAATATAATCATATCAGCCAATTTTTGTATTTGTTCTTTCATATCGGAGGTCTCAGAATTTAATATTTCTATTGCCTCCTCCACTTCTCGATTATTTTCTATAATTTCATTTAAT

>BPA_6264

CAATTGTTGTTGATGTTTCTCCTCTTGGGCCTTAGAAATTATTTGAATCTGTGACGTTATGGCTGAAGTATGATGATGGGAAGACGACGATGAGGACGAAGTTGTGTTAGCAGACGTAGAATTATTATTGTTATTGTTAGAGTTGGCGTTATGGGAGGGAGACATTAACATATTAGCAATCATATGCTGACGACGTTTATTATCTGGTTGTTGTTGTTGTTGCTGCTGCTGTTGCA

>BPA_6268

GCCAAAACACAAAATCGAGAATGATAAAGATTTGGAAGCGCTAGATTCAGCAAATTTGCCACAAACTGTAGAGGAAATTGAAACAGAACAACAAACAACAAAAGAAAAATCATCATTATCATCAAGTGCGACGAATGCAACAAATTCTTCATCGACGACGATTAATAACAATACAACATCTTCAGCTACATCATCTTTAGCATCGTCGCCGACACCGTCAACAAGTGTAGCTCAC

>BPA_6278

TCGGTGATTTGTTACTACTTAAAGGACTACTTGTTGTTTTCGTTGTCGTTGAGGGTGGCGGCGGTGGTGGTGGTGTTGCATTAGTACCCAAACGAAATTGATCACGTAAAGCTGATACACTATTCGTCGATGATGACGTTGAGGGTGGCTGAGTCGATGAAGGCGATGATGAGGCACTAGCCTGTGATATGAGTGCTGTATTAACAGCACTTATAGGACCACCAACATTAGAAGATGAACCAGTATTAGAGGAGA

>BPA_6317

CCTCTACCAGCTCCAATAGTAAACATAGTGAAGATGAATTTATTGTCGTAAGAGAACAAGATGCCACATCATCCGGACCAGCTACACCACCCTCCAAAGAAGAGCTTAAGGATAAAATAGTGCAATTAGAAAATAGAGTTTCTGAATTAACTTTGGAAAATGGCAGTCTCGCTTTGAAATTGCAAGATCGTGATATGGAAAAAGATCTTTCTATAAATGTGCTTAAAGATTCTATAAAAACTCTACAAACTGAAAATGATGAATTGGCCGAAAA

>BPA_6328

AATGTATTTGATTCTTTCTTCACGGTGGTAAATGTTTGAGAATTAGGGGCTAAAGTAATAGTCTCTTGTTGACTGGTTTCGAGGGTACCCAAATGTTTCTCTTGCTGCTTAAGTTTCTCTTGTTCGATTAAAGTTTTTAAAGTATTTTCAAATTTGTTGAAAGCATTTGTAGTTTCTTCTATGACAGCGGTATGAGCTTGGGTG

>BPA_6345

CGACGATGATGTTTTAATCGTCAAAGAAGATGGTGAAGTTGAAAAACCCAATAGTTCTTTGAATAAATCAACAGTCGATAAGGATCCCAAGGAATCTGTTAGCAAACCCAAGATTGAAGAAAGTTTGGAAGTATTAAAACGTAAATTTATGTTCAACATTGCTGATGGTGGTTTCACCGAACTCCACACTTTGTGGTTGAATGAAGAAAAAGCAGCCGTTCCCGGTCGTG

>BPA_6351

ATTTTGTTGTTGTTGTTGTTGCTGTTGCTGCAACTTTTGACTATTTAATACATGAGCCTTAAGACTTGTAGGTGGTCTTACATTAGTATGTTGGGCATGCATTACCGTAGAGGTCTGTTGTTGCATTATTATGGGTTGTTTTGTAGGTGTGGCAATTTCTTGTCTTGTTTGTTGCTGCTGTGTGTGTTGGAAATGCTGTTGCTGCTGCATCATAACATTACTTGGCGGTGATGCTGTTGGCGGCAAAGTTGATGTTTTCACTACTACACTAACTGATTTATTTAACATAGCAGTAGCCACTGTTGAT

>BPA_6352

AAAAGAAGAAATAATCTAAATATTTCACGTAGTCCACCAATGACACCACAACGTCGAAAATTAAGTCCAGTAAGGGATTCTCCAATACCTGGTTATGCTGCAAGAAAAGCCAGAAAACCGTCATTGGAAGAAATCGAAAATGAAGAAGAAAAAAGTTATGTAGTGCATTTAGCTTTGTTCTTGGGAATTTTAGCTATAGGG

>BPA_6358

TTCGAAACATGTAATTTAAGTCATTATGATTTAGTACTCTAGTTTCCAGTTCCGCAATTTGATTAGTGCAGTCAATAAGAGCAGCAGAATTCGTATAAAATTCACTATCAAAAAATTCCTGAAATAATTGATTATAATGTGTAATGATCTGTGTTTTAAATTCATTACTATCCTTTCCAATAGTGTTAAAAAAGTTATGATCTAAT

>BPA_6366

TGATTTGTATGATCACCTTTTTTGAAACAGTCTCGACATATGGACATACATGGTGATATACCACAGGTGCGACAACGATAGGCAACAACATGAGGCACCCATACTAGACCACATTTAGCATGATTATCATAACTTCGTACAATTTTCACAAAATCACTAATCGTTCTGCCACCTGCAATAAGCCAACGAATCCATGATATACTATCTGGATTATCTATAGGTTTGCTGGGATTGAAAAAT

>BPA_6395

CTTTATAAAGCCACCCAAGGTGGTTATGTGGTTAAACCACGAGATGAAGCATCTAATAAAGCTGAAGGACCCTCACAAGAGGAAATTGAACGTATTGAGCGTATATTAAATGAAAATATTCAAAGACCCAAATTAGCCGGTACAGGTTCAAATAGTTTAGGAAGTGCTACCAGATATCCATCTCTTATATATCGTAGCAGCAGTGATAAGGAACGTCGTAAATCGGCTGGCG

>BPA_6399

ACAGATAGCATCGATCAAAGAAATGCACAAGTAGATAGAAATGTTTTAGCATCAAATACAAAAAATTTAAATACCACTGTAGATTCTCAGTTTGCGACACCGCAGATACGATCGTTTTCAGTTCAAAGACGACCTCGAGATCTGTGTTCGACTCAGTCACAAAAAAATAAATCAGCTATAGTTAATGAATTTCGTACCCAAAAAGTATTGTTTCAAAC

>BPA_6406

CTTTAATCGACTATACTTTGGAACAAGCTTCTATAAGAATATTTGAATCAACAGTAGACAATTTATCAATTAATGAAGACGACATGCGCAATTTTTTGGTATATGTTTCACAAAATGAAGTAGATCTAACACAGGATGCAGAAATGTTATTGAAAGACTACTTTGTCACATCCCGCTCCAATAGACCAGAATGTCTTACAAAGCAATCCTATATCATGC

>BPA_6430

CCACAACAAGCTGCATCACAATTTGGTATGATATCAAGTGGAAATTGGCCTTGGAAGATTGGAGATTTATGTTTAGCCAAGTACTGGGATGATGGAAATTATTATAATGCAAAAATCACCGCAATTTCGGATAAAACGTGCGTTGTTTTATATTTAGGATACGGTAATCACGAGGAAGTATTAAAATCTGACTGCCTACCCATAACAGATACTCAACAC

>BPA_6432

TCAAAACATTCCACCATAGCTTTGGATGCTTGATTCATTAGTTTTTGAGCTAAGCCCAAACGACGATAGGAACGCTTAACCGCTAATGATGTTATATGACCGCGTTTACTATCTTCACCAGATTCAGGTTCTTCCATTTTAGCGAGAACATAACCAACAATATTGCCCTTATCATCGGCGGCCACATAACTTAGTTGGGGCCAGGTAAGGCCATGATAGAAATAATAT

>BPA_6435

GTGAATGTACAGCTGAATGTGGAGAGGGTAGACAATATCGTACACGTGTTTATAAACAGGCGGATATAGCTAAAATCTATAATTGTGATGTAATGGTAGAGAGAAAACAACAAAGAGCATGTTTTGGACAAAAATGTGGACCAATGGATTATACCAATTTTGACACAAATCAATGGTCTGATATGAATAATGAAGAGGAG

>BPA_6444

AAAGCGCAAAAAAATAGTTATTGAAGGCTAAAAAAAAGTGATATAGAGAATCAGAAACTGCTGACAAAGAAAACCTAAAACAAGAAGCAAATAGTCTTAAATAAATAACAACAAGAACAACAAAATGTCTGAAGCTTATTTTGATGAATACGATCATTATAACTATGACCAGGATAAATATATCTTCTCTGGACATAGCGGTAAAAATCGTTCCAAAAAGGAGGCAAGTGAACATAGCAACCATTTTGATCCCAGTGGACATTCCAGAAAACTCTTAACCAAATTTATGAACACTAATAATAATAGAAAAGTTGTCTGTACTGGACCCAAAAATTGATATTGATATAATACCTGGTCTCATTGTCGTCACAAATAAGAAGAAAGTAAAGAAA

>BPA_6449

GTGATGTTTAATCAAGAGACAATGGAAATTGCTAAACCATCGGAGCTACTGGCCTCTGTTAGAGCCTATATGAATGTTTTGCAAACTGTGGAGAATTATGTACACATTGACATGACCCGTGTTTTTAATAATTGTCTATTACAGCAGACACAAGCACTAGATTCTCATGGAGAGAAAACTATAGCAGCTTTATATAATACTTGGTATACGGAAGTTTTATTACGACGTG

>BPA_6484

GCAGCATCTTGTACAAAAGTATCTACACTAGTGGCACCATGTTTAATGGGGAACGATTTACGGCCATCCAACTCATACAGAGTACCATCAACATTAACCAAAGCAATGAAATGATGGTAGACTTTTTCTTCAGGGTTAGCAGCCGTTTGACCTTCCTGAGCCAAAGCTTGATGATCTTCAGTCATAGCATCATCATTTTCC

>BPA_6488

GCCAATTGAAATTTTTTTTGTTGCAATAACTGTTGCCGTTGTGTGGCTATATCCTCCAATCTAATGCACCATAATTCAGAAATTGCTGCTGCAAATATAATCCCTTTTGCGGAACGTATTAAATGTTTGGTTTTATTTAATTCCGGTATAGTTTGTACCAAAGTATCTTTGTCTATGCCATTTGATTCCAAACAACGTACTTCCAAACCATTAGCGACACGACCCACAACATAT

>BPA_6493

ATATCAGTAGAAAACTTCAATTTCTGCAGGATGCTTATAAGTTTGTTTGCCAAGAAGAGAAATTATGGCCAAAACTTCATATTGAGGTTATGTTAATGGATTATCTTTTTACAAGCGAACGTCAAATACCCACCGATGTGGCCGTGTTAATTATCTCATGTCTTATAGATCTTCAAGAAGATTATCGTTATTATGTGAAACGTGC

>BPA_6511

GAGGAAGCCGTGGTCATGCGTAGACCTAAACCCAAAAAACCAGTTGAACCTCAGGTAGAGGAACACGAGGAAGAAGAGGTGAACTTATCATTCAAAAAACCACATAAAATCAATGAAGGTGTTGCTGAAGAAGCCACCGTACTCAAGAAGAGACCACAGAGACCTCTAACCTCCGATGAAGCTGCTGCTGAACTTCACATCAAAC

>BPA_6536

TGGCAAACAACTTTTCTGCCGATACATGGTCAACTATATTTGGAGGAGGAATGAAAAAACTATTAAGGAAAGCAACTTGTGAAAATAATACACCACCAAATAATTGCTCATTAGATGAACCCAATGATGAAAGTAGTGTGTGGAATACTGTTACTTCTATAACAAAACAACGTGTAAAATTAAATATGAAGATACTTGGAAACTTTGGTAGTAAC

>BPA_6538

AGGAATCACCACAATCACGTGTCCTTAAATTGGGTGATAATTTAATCTTAAATACCACTTTACATTTTGTACCTGGCGAAGTGACATTACGGCGAGTACCTACCTCGAAACCGGGAGCTTTATTTGGTGCCAAAATGCAACAAGTTTTAAAACGTGAAAAACGTGACATTCCATTTATAATAAGTGCCTGCATACGAGAAGTGGAAAAACGTGGCATGTCGGAAGTGGGTATTTATCGTGTCAG

>BPA_6557

CTAATATGGGTGATGATATACCCCTACATTTGGCAGCAGCTCATGGTCACAGAGATGTAGTACAAATGCTTTTAAAAGAACGTTCTGATGTTAATGCCGTCAATGAACATGGTAATACTCCCTTGCATTATGCCTGTTTCTGGGGTTATGATATGATTTGTGAGGATTTAGTTAATGCCGGCGCTTGTGTTACTATAGCTAACAAAGATGATGA

>BPA_6559

TGCTAGCTAATGAGGGTAACGTTGTTGCAGTTTCCAATGGTGTGGGAGGTGTGGTGGGCGTTGTTGTTAAACCGCTACTACCACTGTTTGAATCATTGGATGATGATTGTTGCATGCGTTTGCGATATTCATTAATTGATAGTTTACGCTTAGGTTGTGGTGGTGGTTCTGGTGCCGTTAGCTGAGGATTCAAACGTGGATCATGGCTAGCGGTTTTTGTATGTATTTTCATAGCTGTTG

>BPA_6574

GGACGACGTACTGAGACAATTTGTGCTGCTTGTGATTGTGATGCGGATGAACTATTTGTACCGGCATTTTTTATAACAACAACGGTGCCATCAGGTCGTACTACAACCTCATCATCATCATTATCATCATACACATTATCATCATCATCATCATCGTCATCAGCCTCATGACCATCATGATGATATGATTGATGATGACGATTTGTAGTCGAATTAACTTTT

>BPA_6583

AAGAAATGCTGCAGAATATTTATTAAGTTCGGGTATAAATGGTAGTTTTCTGGTAAGAGAAAGTGAAAGTTCACCCGGACAAAGAAGTATTAGTTTAAGATATGAAGGTCGCGTTTATCACTATCGAATATCTGAAGATCCAGATGGTAAAGTCTTTGTAACAGCTGAAGCCAAATTTAATACTCTAGCCGAATTGGTACATCATC

>BPA_6592

GCATGATCGTTTTGAGGAAAACGAAGAATATGCTGGTTATATTATACCACCTTGTCCTCCTAGGCCTGACTTTGATGCTTCCCGCGAAAAATTGCAACGTTTAGGAGAGGGTGAGGGCAATATGACAAAAGAGGAATTTAGGAAAATGAAAACAGAATTGGAAGCTGAATATCTTGCTACCTTTAAAAAAACTGTTGCTATGCATGAAGTATTCCTG

>BPA_6623

CGAAGATATAGGTGAAGAATTTAAGGTTAATGCTTCGTTTTTTGTAAAATTCGAGGCACTAGTGGTTGCTACTGTTGTTCCAGTTGCTGTTGGATGTGTGATTGAGGAAGCTGCGGTATCACTTTCAGTTTTTATAGATGAAGTAGATGCTGCTAAGTTTGATAGGGATGTTGCTGCTGTTGTCGTTGAGATTGTTGTTTGTGTTAAAACTATAGTACATGTGGCTGATGTGCTGCTGCTTATAGTTTCCTGTTTATCCGCTGTCACTTCTAATTTAACTTTATT

>BPA_6625

CGGTGACAGGGCGTGTGGTGGCCAAAGGTATTGGTGGTGGTATTAAGCAAAAATATCATTGGGTTAAGTGGGTAAGAGATGGTCCTTTGGAGGGACCACCACAGGAGGAACGAGTTTTGGAAATTCTAGATGATGGCTGTAGAACAGCAAAAATTGCTTTAGTAGGTGTGGGCGATGAATTGAAATACATTTTGGCTACGGAAAATATGAAACCTGGCG

>BPA_6663

GCTGCCTATCTGAAAGGTATAAATTTTCAGAAAAAAGTTTTTGTTGTCGGTTCAGAGGGTATAACCAAAGAATTGGATGCTGTGGGTATTCAACATAATAAAGTGGGGCCTGATGTTATGCCTGGTCCCTTGGCCGATTATATATCAAAACATTTGAAATTAGATGAAAATGTTGGTGCTGTTATGGTGGGATTTGATGAACATTTTAGTTTTCCTAAAATGACACAGGCGGCTTCATATCTAAAC

>BPA_6678

CGGGTTCCTTCAATGAAAAATTCTGCACCTATATGATAGACGGAGACCAAAGCATGCATATATTCACGAAAAACATCCCAATAAAGTTCATCATTTGAAAATGAACGACGCATAAAAAAGGCACCAGTTTTACGTAACATGGTGCCCATGGCAAACATAGAATGGAAATCCATTCCTGCTGCTATACCTGGTATTTCAATGTCATAATGAAAACATATATAAGACATTAAAATAAAATCCATATAACTTCGATG

>BPA_6709

TTCTGCTATATTTTCCTTTTTATCTGAGTTTCCAGTTGTTGATGGTGGCTTTGCGGTTTCAGTTGGTACTGGTTTATCAGTTGCATCGTCTTTTGATGGCGGCGTTGTTTCAGCTGCAATTGTACTACTGACCTTAGGTGGCGGTAATTCATCGTAAAGTGGTGTAGTGGCAGATTTTGTGTTTTTCTCATTATCCTCATTTGTTGTCGCTGTGTTAGTAT

>BPA_6712

TACATCATGCATGGCAATATAGTCATAAACACCGCTGGTGAAACGAAAACCCACATTAATTAAAGAGGCTCGATTAAAGCGATAGCGATCCACTTGATTTAGTACAAATATATGATGTGTTATACCCTGTTTCTTGAGGAAATTTGTCATATGAGGCACAAATTGCAACAATTCTTCAAAACGATCACGAAACGGTACCAAAATGGCTAGTTTATGAACAATTTGTAATTGATCGGGAGAACGATTCTGCATTTCAGCATTTTCCACTCGCGATTTCCCCCGGCTA

>BPA_6724

GCCACGACCTTTTAGACGTGGCAGAACTCCACCTAATGGTTTAGGTCCCGATCGCTCACCACAAACAGAACTTAGTCCGGAGGAACGTGACGCGCGTACAGTTTTTTGTATGCAACTTTCTCAACGAGTTCGAGCACGTGATCTTGAAGAGTTTTTTTCTAGTGTTGGAAAGGTACGAGACGTTCGCTTAATAACATGTAATAAAACGAAACGTTTCAAAGGAATTGCCTACATAGAATTCAAAGATCCTGAATCGGTTGCTTTGGCTTTAGGCTTATCAGGA

>BPA_6726

TGGATCCATAATAACGCTTTCAAGACTGTCTTCGCCGTTTAGAGTATTAATAATGCTGCCACAACTATCATTTAAATTTTGTAGCTGAGCTGAAGCTTCCAATCTTAATCTGGCCCTTTCCACACGTGCTAATGTTGGACTATCCAATATTGAAGGAGCACGCGATGGGGGACGTGAATTCAAGGTAGATGTACAACCCATTTGTTTCAATTGCTGCGGCTGTTGAAGAGA

>BPA_6759

ATACAATTTGGAAATGAGTTGGTTAATAATGTGAAATCATCTAATGAACAGCACGAGTTGTTGCAAAGCCAAGAGAAACATTTAAAGGAGTTGGAACTTCAAGAAAATCTTCAACAAGAGGCTAAAATATCTTTGGACCAGTTTGAGGAAATCAATAAGAAATGGCGGGAAATGGAAAATCTCAAAGAACCCATGGCCATATTTGAAGATATGCAAAAGCAACGTGAAAACATTTTAAACTTAATGGCCAGTAAGGATGAACTCATCGAACAGTGTCAACAGGAACTAAAGCGTATTAATTTTAAATACTACCACGATCAGCAAAAACAGTCGGAAG

>BPA_6770

TGGAACCAACTTGCACTGTTCATATTGATGAGTTCCAATTTTAAACATCCATTGGCTGCTTTACGCACACCTGTACAAACTATCGGATATTCTAGTTCTGGCGTTATAATCATCTCGAATACTGGTGTATGACCATTTTGTACACAACCATGACCACCGCCCAATATGCTTGTGGCCGGCCACTCGCATTGTTTCAACAGCATAAATT

>BPA_6787

GGATGGCTCATGTAATGGTTTGCAACACTATGCCGCTTTGGGCCGTGACAAGGCTGGTGCAATAAGTGTAAATTTAGCGCCTTCAGATATACCACAGGATGTGTACAGTGCAGTGGCAGCTTTGGTGGAAAAGACACGTAAACATGATGCTGAAAATGGTCTTCATGTAGCGCAGGCGCTAGATGGTTTCGTTAGACGTAAAGTAATCAAACAAACAGTCATGA

>BPA_6796

AGGTAATAGCAGTGGTGGAAATGCGAGTAGTACACAGACTGGTGCAAAATTTCGTCATGGCCTATTGCAGCTTATGATTCACACAATAGATCCGCTACATGATGGTGAGTTCATGATGTTTCCATAGTGTTTTGCACACACTCACACATACACAACCACCACCAACACTCACTACTCACTCACTAACAACACTGCAGAGACC

>BPA_6804

GGTGGTGATGGTTGTGGTGGCGGTGGAGAAGTAGTGGCAAGTTGTAAGGACATGTCATTAGGTGTATCATCACAATAACCAGAAGCGTAACAATTGCGCTGTTGCTGTATGTTATTTAAGTGACCTGGTGCATCGTCCAGCAATGTTGTTGTCACTTGCAGAAAATTATTGGGACTTGTATCAGGTGTTGGAGGGGCCAGCGCCGTCGAATGGCTGTGCATAGCCTGTTCGCAGCGCGTACGTAATTTTGTAGACGATTCAGTTATAGCACTAATATATACTAG

>BPA_6813

TTGGTTATATTCCATGAATATGCAATGCTTCAAAAGTCACACAGTATCTCCAAATGCTGCGGATTTAACACAAAATCTTAAGGAAAATCTTAATAACAATGTTAATATTGCCAATATTAATAACAACAGTAATGTGACTTCCATTGCAGGGCCAGAACAATGTAATGGCCATTTAAAGGATGCCAGCAATGTATTAAATTTTAAGAATAATAAAGCATCGGAAACACCTTCATGTTCTCAAACTCAAACACCTTCAGCTCCTTTACCACCAACTCTAACCCCACCTTCACCGCCTCCGAATATGCGTTCGCT

>BPA_6821

GCACCACACTGATAACTCCAGCATGCGGCAAATGTCTGGGTGAATTCTTAGGTGACATTGGTATGGGCGGTCTAGCAGGTGCAGGTCTTGTAGGTGGCTGCTTTGGTCTTTGCGGTATTTCAGGTGTGGCGTTTAATAACTGTGCCTGTTCTTGAGGATTTGCACAAAGTGCTATAGTGTTAGTGGTACACAATTCGATTTCATTATCAACATATTGATTCCAATTGGG

>BPA_6826

CCTGAACACCCGGTATACTTAAAGTATAGGGTAATGTTTCGTTAGTACCATTAGGTTGTGGTATGGATATGGTGAAATTCTGTGCTGAATTTATAGATTGACTTGCTATGGATTCTTCCGTTGGAGTAGTATCCATGAAATCTTCATCATCTTCATCGGTTATACCACAAAGTTTCATAACATTACCCAAATCTAATTTTCC

>BPA_6833

CATTATTGAGAAAACGCAATAGTGTGCGTAAAGTATTGCGATTTTCATGTGGCAACATTTGGCATATGATGGCCAAAGCTTTGGCTTGATCTGTGGGCGGGAGAGCATGACACTGATAGAATAATTGTATGAGTTCATTGGTGAGCAGGGGTTGGGGTAATTCACGTAGCCAACGTTTGAGCAAAGCACTCAAATCGTGTACACTGGCTGAGCTTAAGAGTTGTTGGA

>BPA_6842

CTATGGTGCCAATGAGGACTATCCTAAACCTAAATACTTTTCTCTAAATCGGGGTCAAATGTTTCCCGAGAAACTTTCTTTTATGGATTATCGTTTTGATGAGCAGGAAAATTGGTGGACTTGGCAAAAATCTAGTGAAGATCCTTCAGCTCTAATTGCATCATTTCCAGAAGGTGCACAAATCAGTGAATTAATTGTACCCACAAATGAATCAGGCTATATGAGTTATTGGCAAGAATTTTGTATAACAAAGTCTTATCCAATGTTAGTTATAGGACCCACCGGT

>BPA_6861

TTTGCATCAAATGCATTATGAAATTTTGGATTCTCATAAGAATAGCCATGTTTGTGGTGTACAATATGTCGGCTATAGTTACGGCAAACAACAATATGTTGCATATGCAGATGACACAAATGCTGATTTATCTAAAACTGGTGGTGATATATTCCAGGGCATCGATGTAAAACATGATGAAACAAATCGTGAAGATGAACGTGGTCGCAGTAGTAAATTCAATGAA

>BPA_6862

TTTATTTCCATTTTTAGATTCTCTTAAATTCAAGCTTGATTTTGAATTGCGATCTGTTGTTTCATGACCATCCTGGCCAATTTCATCTTCTGTAACATCTGCCGAAATGTTAGATTTTGTGGCACCATCAGAATTTGTAGCTGCTTCAAAAAGTGAGGTGGGATCACCACCAGATTCACGATATTTCTCAACATCAGATAGTAAACGTTCCAAATACTCAACATATAATGTTTCCTGTTCCAATTCTTTGGTTAATTCAGCGATTTTTAACTTGTGACGTTCTAAACTAGCACGAAC

>BPA_6866

TTTGGATAACAATAATACCTATTTGAGATGGACCTGGTATCCAAAATTGATATGCAGCATAAGGCGTATGTTTAAGAAAAGGATTATACCTAACAAATTATGGCCTTCAACATTTCTGTGTATAGAGGGTCTCATAAATAGACAACTAAATAATTTAAAACAAAGAACCTTAATGGAACTACTTAAAGTTTGTTCCAACGAACGCAAAATACCCATGTTACGTTTAAATTTAGTTTGTTGCGATGAAG

>BPA_6878

AAATTATGAAAAATTAAATGGGGATTTTTTTCACTCATATGACCAATATCAGGTATGTCATGGCGCATTACGACACCAGATATGTTAAAAAAGGCAGTCGGTCCGTAAGGCATATGGCTTATAACAAGGTTGTCTGGTACTCCTCTATGCTCATGAACAATAATAATATCTGTAACATCATTTGCTTTACAAGCGTTCACTAT

>BPA_6885

AAAAGATCGACGCATAAGAAGTCGTTCTCGTGAAAATCGACGAAGTAGATCTAGAGATCGGAAGAGTAGCAGATCGCGATCGGTTCGCAGAAGCAGCAGATCACGTGATTACAGACGCAGTCGCTCTAGAGATCGTCGATCAAGAGACCGCCGTTCTAGAGATTATCTAAGTCCTATTAAAGAGCGCGGAAGCAGCCGATCATATAGACATTCGCGTTCACGTGAGCGAAGCAAGAGA

>BPA_6899

TGGGTAATGATGTGAATAATGTAGGCCACAATGATTCCAATAAGTTGTCCACTGAACTACAAGAAAAAGAACAGCAAGACAACAAACAACAACAGCAGCAGTTGACACAACCAAAACAAAAGCCACCTCTGCCAGTACGTAACAAAACCATGGAAATTGCCGGTTATGTGGGTTTTGCCAATCTGCCCAATCAGGTATATCGTAAGGCCGTCAAAAGAGGCTTTGAATTCACGCTTATGGTAGTGGGAGCCAGTGGTTTGGGTAAATCCACACTCATTAATTCCATGTTCCTTTCGGA

>BPA_6903

AAGTGGATACTGATGAAAATGTAACAGAAGATTTGAGTAAACCTAAAGATGAGGAAAAATCAATTTCTGAAGATACTAACAAATCAATGGAAAAATCCGAAACTAAAGAAGAAAAACTAGATTCTGAAGCTGAAGCCAAAAAAGTTGATAACAAAGATGTGGATGAAGAAGAAGAAGTTGTGTGTCTGGATAAAGATGATAAGGCAGAAGCAAGTAAAC

>BPA_6917

CAATTTCCTCCATACGAACACCATCTGCATAAGCCGATGGCTGCTGTGAACCAGTAGTGGCCACCGAAGAACGTTGACGCTCAGTGTCTTGGAAAGCACGCATTAAAAGACGTACTAAATGATGTATGGCGCCATGTTCGCGTAGTGGAGCTGCATTTGCTGGGCACAATGCTAAATTTCGTACTAGACCTATGGCGGCCTTTATTAATGGCCAGCGTGATGGTGGATGTAAAAGTTTTACAATCACCGAAAGACCATAATTCAAACGTACTGCATTTTGAGCCATT

>BPA_6931

ATAATGTTTTTGGAATTTGAACCAGATATCGTTTTATTGGCCGAGCACGACGTCCTTTGGGACCAGCACGATGTACTTCAACATCACGAGCTCGTTCTTTCTCGCGTTCTCTTTCACGTTCTCTATCCTCTCGTTCTCGATCTGCTAAATCGCGTTCCTTCTTTCTAGGTTCAGCACGATCACGTTCTTTTTCATTGCGAGCCCCATGACCGCCACG

>BPA_6938

GTTACCACCATTGCCATGAGGCATATTGTGTTGAGATTGTCCAGATTGTTGATGATGTTGAGCTAAAGAGGATGCTGCTGTGGCTGACGATGAGGACGATGATGCCGGTGCCGATGAAGATGTCGTGGCTGCTGATGACGTTTGCTGCAACGATTGATTGAGTAGTGCCAAATTACTGGCCTTCATACGGGCTATAGTGGTATTTGCCACCCCTCCAAAAGCTGGGAGACATGGTGACATACAAGGCGTTGAGCAGAGGCTATTACTGATCTCACACTGGTCAAGTAGGCGGAGTAGCTCGTTGGTATCGGGTCCGCCATTGATTTTCTGCGCCTGATTTTCATCTTTTTTGGAAAACAACGACAGCGCTGTTGTGAGATCCCACATTTGTATATTGCCATTACAATGGCCAGTCAATATAAAACGAGAACCCATACGTGAGCTAACCTCACATTCATGGACGCAAAACGATGTAATCGAGGATCCGTCCAC

>BPA_6966

TTGCAGGTTTAATACGAATGGTATGTCTGGTATGCATGGTATGTACGGTACTCATGGTATGTGTGGTATACATGGTACGTATGTTATTTGGTATGTACGGTACGTATGGTTGGTTTGATGTAAGAAATGAATGGAATGCTGGTATATATGGTATGTATGGTATAAATTGTATATATGATTTGCATTGTATGTACAGTATGTATGGTACGTAAACTATGAATGGTAGGCATAATATGTTTGGTACCTATGGTATGTATGATTTGTACGATATGTACGGTACTTATACGTTTGGTATATATGATACGTTTTGTATG

>BPA_6978

CAATGGTCAAGTTTTGTATCGAGTATAGTCATCTCATCTTCTAAGCGGGAATTATGAATTTTTTGCGTTGACTTATTGCCACCATTAAGCATTTGTATAACAGATTTATAACGTTCCAAAGTTTTGCATGTATCAGCAAACCCCTGTTGGTGTTGGAGTTCAGTAATCCATTCCGTTAGAAGTTTTTCGGCCTCCGTATATTTTTTTAATTTACACAATGCGTGTACCAGCCATCGTCTAGTACGTGAAAC

>BPA_6988

TTCAAATGTTTTTTCAACATTTTCAAAACGTATAAATGCCATATCAAGTCGAGAAACAACTCCTAACTTCGATCAACAACAGCAGGACTTAAATGCGAATAATTTGGCTCCTCAATTAGATATTCAGCCAACTTATACGCAACAGCAACAACAACTTCCTCAACAAAGTAATTTCTCTCAAGCTGCTCCTCCACCATTTTATGCACCAACACAAGGTGGTAATTACTTTTCGGGTTTATCTGATACATCAACTCAACAAGATATACAAACAAATTTACCACCAC

>BPA_7001

TTTGTTCCAAATAATCGGGAGCAAATTCATCAATTTCATCTTCATCACTAGATAATGCTTCTTCGCAATCATCATTTTCCTCCTCTTCTTCCTCCTCTTCTTCTTGAGCACGTGACTCATAGGCTCGTTTCAAACCGTCGAATAGTAAAATTAATGAAGGAATAATTTTACCGGACACTTCGTTCAGTACTGGTGGTTTAGCATCACCCAACGATATCAATGTACACAAACCCAAAACACACAATTTACGATCGTGAATACCTAA

>BPA_7002

TCGGGTGCTATATAACATGTACGTCTTCGGCTAGTGTCAAAGAAATATGTATAATCCGCTGGATTATCTTCTGGTAAATATGTGGGCTTAAACGAAGCAAAATCTGACAATAGAATCCAATTCCAAGATGTTATTAATATATTTTCCAATTTAATGTCACCATGATATATCTTTTGTTTGTGGCACTGATTCAGAGCACAAAGAATTTGAAAAGTAATCCATTTCTTTTCGAGTACCGTGAGAAATGGTCGTGTAGAAACACGGT

>BPA_7007

TGAAAAAGACGAAGAAAAAGAAGAAGAAGGAGAACCCTTAATAAATCTTAAAATTCAAATGACAGAATTTCAACTTCGAGGTGAACAAATGACATCAAATGCTATAAATAAAAAACTTAAAACTTCTTCTTCTTCCTCCTCAAAAACAACAACAAAATTCGATGCTTTGACAGCGGCTAAAATGAAACCACTAAAACAAAAACGCCTCCACCTTCACCACCAACAACAGC

>BPA_7010

ATTTCTCGTTTACGCTCACGATCTTTGAGTTTATCATTTTTTAATACAGCCAAAATTTCATCGGCAGCACCACACAAGATATCTCTAGGCTGATCTCCCAATGCCTCTTGTATAAAACTTAACAACACTTCATAGGTTTGGCGGGTTTCTTGTGTTTTGGGACGATAAACAATACCAACCATTTCATCAATACCTTCAGATAGCAAAGTAGCTCCTTTCATACGTTCAAAATCGTATTGTGCCTCATCACGTTTTTGACGTTTCGCTTTGCGCTCCTCAGTTTTTTCCGGCTTCGAACGTTGGTAGCGGTCACCCATACGTGTACCATCCAATTTGCCCACTAAGGACATAACCTCACCGGTAGCCTCATCGCGTCTAGGAC

>BPA_7015

TCGTCTCTGGCTTTAACTTCGTTAATTAAACGGTGACATAAGCTTGCGTAAGACACAGAAAAATTGGGCTCATCGATAGCTTTTTCAAAAACTAAAATCATTACAGCTTCCATTTTTTCCATAGTATCTATTTTAAGTTTAATAATTTCCTCTACAAGTGGCTCGAACTTTTCTGGAGTTAATTTATTTAATATACCACGAACACGGCGTATCAATTCCTC

>BPA_7025

GTGAGAAATGTTTGGTCACGAAACGTTCAATAGCATGTGCTACTGATGAGGAGAGTAAACAACCAGAGACAGCTAAAACAGTTTCACTTAAGTTATTAGATATGCCGGTACGTAGCAATACATTTAGTTTGGGCGATAATGAGAAACTGAATATAATGAAAAAGAACATAGGAACTCAATATACCCCGGTGGCATTGCAAACTTCTGCCTGTCAAACACTAG

>BPA_7027

CCATTTTTATCGGCTAAACGTCAACTCGAGGAATTATGTTCGCCCACATCCGACTCAGTCGATATACCAACAACACCGCAAACACCACAACTGCCAAGTATGCCGGCTACACCCAGTAGCAATAATAAAATGTCTACATCTCTTATACTCAGCTCTTCAACACCAAATACCACACCTAATCTCATGACAACATCGCAAATACCACAACAAACACAAAAAAC

>BPA_7051

GGGTAAACCAAAATTACCCATTAACCTTTATAACATTATGATACACTCATTGGGTGAAATATTACCAGCAAATCCAAATTTTCATAATGCTCAATGGGTCTACCCTGTTGGATATGTTGCCACACGTATTTATGCTCACCCCAAAGATCCGCAAAGAAAATGCGTTTATACTTGCAAAATATTAAATAATGCTGGAATGCCCCAATTTCAAATTATTCCGGACAATGATTTGGAC

>BPA_7068

ATGCTACTGTAGCAGCTCAAAAACTCAAAAAATCTCTAGAAGAGCTTAAGAAACAAGAAGAAGCCATAGCAGCTTCCGAAAAAGCTGCTGCAGCTGCGGCTGGCAACGTCTATATGCCGGGTCAATCCCAACCTGTTCAATTCGAAGCATTGCAACAACAGCAAATACCTATGCAACAAACCCAACAGGTAGTTATGCAACAACAGCAGCAGCAACATGCTGCCACA

>BPA_7079

GAATCGGAAATTTCTGTTAGTGTCTCATTATCGACACCTTCACCTACATCTCCAAATAATCCAATTCACCACAATCACCAATACACACCAACAGCCATAACAAATCAACAACATCAAACACATTTGCAAACAAAATCACCTATTCGCCATCGTTCAGTCATTACTTTACAACCAGAGCCACCATACACGCGTGATTTTCTTTATACGCCTGCCTCGTTAACATCAACACCAACATCACCTTCTTTGGCAGCGCCTCCGTCAGCTGGTGCAACAGCAGTCGGAACCACAACTATACAAACAACATCATCTTCGTCGGCTACCTCTTCGCCTACAGCTTCTGTGGCCGGTTCACAAAAAAGTTTTACTTCGGTTAATCTCACATTACGTCCGCCCGTCTCTCCCACCGGTCCAAATTCTGCTCTACAGCCCTC

>BPA_7090

TACGAGATCCGAAAGCAATCTCTAATCGCTTTAGGTTCGTTTTGCGTAATGAATGATGAATATTTAATAAGGCCCGATCTAAAACAATTTTACTGTGATATACTAAGTTCAGAAAAATCAGAACCTAGTATAAAAATAATATGTATGCGCAATATATGGATTTACTTGACTGAATCAGAAATGTATATGTATAATAAGGAAAAGGAATGGGAAAAACAGTCAAAAAATGAAGATCTTAAAGAAATGAATGATGTCTCCTCC

>BPA_7093

TGCATTAAGCTATTGACAGAACTTATTGAAGATTTAAAAATTGAAACAACTTCATCACTGGAAGCAACAATCACAACTGGAACAACCGGAGGTCAAGCATCTACTTCACCATCATTACCCGAACCCGCAAGTTTTGATATATTAGAAAATTATTCTACTTTGCAGAGAGTAATGCGTGTTTTAAACACAGCTACCTTACATCAACTGTTCACATTTC

>BPA_7112

CCGATACTCATTGTACGATTGAACAATATTTAAGATTTATACAAAAAAGAGCATCAGGAGAATTAGTAACCACAGCCACCTGGATGAGAGAGCAGGTCTTAAATCATCCAGAATATAAAAAAGATTCCATAGTTACCGATCGTATAAACTATGATATGTTAAAGAAGATACAAGATATCCAAAATGGTAAATTAATTGAACCTTCTCTGCTGGGACAGGAGGGTAATCATACAAAGACAAAAGATTTCATACCACCAGCCATACAAAAACACTTGAATGGTTGTT

>BPA_7129

ATGAAATGTTAAAAGAAAAAGCTTTACAAGAATTAAGAGAAACGCCAGAAAATATTGAAAATGGCCTAAAGGAATTGAGAAAATTATTAGAAGCTGAAAAGAACCTAATTGTACCCATGGACAATGTGGAATGGTTAACGAAATATTTAAGAGTTTGTAAATATTATCCAGAAAGTGCCAGAGATTTGGTCCAACGTTTCTATAAACTACGACAAAAATATGCCGAAATAAGCCAAATTTT

>BPA_7130

CTGTTATTTCTGTTGGGGTTTCAGAAACTGTAGTTTTTGCTGGAGCAGGTGTTGGCGTGGCCAATAATTCATTGGGCATAAATAATTTACGTTTCTTTTTCAAGGTATTCGATTCAGTTGTTGCTTTTGTTGTAACACCATCATTTAAATTTGTACACTCATTAAGAGTTTCACTTGAGGAATGTGGTGTTGTTTGTTGAAGCAAATTATTGGAATTTTTATTATCAACTGGTG

>BPA_7143

ATTGAGATTTTCATTATTATTTTTAATCATTTCTTTTATTTTATGCGGTAACGTTGTCGGTGTTATTTGATTCATGATTTTTTTAAACGCAATGTTTTCTTTTTCGTTTATCTGTTGTTGTTGTTGTTGTTGCCGTTGTGGCTCTGTTGGATGTTGTTGTTGTTGTTGCAGTTCTTTATATTTATTTGTTGTCATTTCATA

>BPA_7155

AAAACAGTCTAGTAATAAGGGACGAGAATTCAAAGGAGTTAATGATGAATTCTCTGCAACCACCAACATGTTTAACGACACCAACAGCAGGATCAATATTAACAACGACGATAACGCCCAACAACAGCAACAACAAGTATGTACATCAAACGAGCGATTGTCTCCCAATAAATTACGATCAATACAATCCCAACCAAACACCTTCATCATTGATCTCCAATCACCACAACCTCCTCCACCACCACCACAACAACA

>BPA_7158

CTTGGCTAAGCGGGCTTGTCGAAATCTTTTGCCGGCGAAAAGATTTAGAAGCACTTGCGGTGAACAATGCCAGGGCCGGATTCATCGTATTCTTGCTTCGAGATCCACATCTGTTGGAAAGTGGACAAGGAAGCCAAGATGGAGCCACCAATCCATACGGAGTACTTTCTTTCTGGTGGAGCAATGATCTTAATCTTAATGGTTGAGGGAGCCAAGGCAGTGATTTCCTTTTGCATAC

>BPA_7173

CCATAATTTGTATTATATTGGCTCAACACACTACATGAGCAATATTGGTTCTTGTTAAAATTGCTAAGTACACCAATGTTTCGACTAAGTTTTGATAATGTATTTTCTTTACTTCCGTTTTTTTTCTTCAGTTGGACATATTTCCTAATTGTTCCAGTATTAATTTATTTTTTTATTGTTTGTTGCTACTATAGATCAACGTAGATGGCAATTATCTAAACTCAAAAACCAGTTTCCCCACTTACTTAAAGTGCGACTTCAGCATTTCGTGTAATTTCTTATTTGAATTTTTAGACTTCCACAGATGTGGCAAGAAAATTTAAAT

>BPA_7174

CTTAAACAACATAGACTGCAAGGAAGGCGATAATGCTCGTTTCGAATGCAATGTTGAACCATCCCGTGATCCTACGATGAAAATCGAATGGTTCTACAATGGCCAACCATTGCAAGCAGCCGCTAAATTCAAGTCCATTTACGACTTTGGTTACTGCGCTTTGGATATGGCTAACCTTTATGCCGAAAACAGTGGCGTATACACTTGCAAGGCTACCAACAGCAAGGGTTCTGCCACTACCTCTGGTACTCTTAAGT

>BPA_7186

AATTTGTCTAGCCTTCACCAGATCATTACCTTGCTTTATCAGTGTAGCACTAATGTCTTGCAGAGATTTATCTAAAATTTGTACTTCAGTAGCCAATTGATGGGCTTGAGTTCTTACCCCCAAAAGTTCATGGATGGACTCAATAAATCCTTGAGAGGAAATATTACAAATTCTTTCAATATCCTTATCATGACTGCGTA

>BPA_7193

GGGGACCTTGCATCACGCCAATATAACTTAGTAAAACTGGTAAAAATATTAAACCGTGTGCAGCACCAATTAAAACAATACCCAAATACATTCTGAAGTAGAAAACTTGAAAGATTTGACTTTTAGCAAATGCCAAAACTAATATTCCACCGAATTTCGTCAGCGTTATGCCAGAAAATATTGAACTGCCCATTTTAGTAAGACAATCAGCAGCACGTCTCTCACGAGTACCTTCAGTTGATATCGAAAAGCTGTGAACCAAATGTGAACAGAATTCCACAGAAATACCAACAGCCATTACAAGATTCACCAGAGAAACAGCATTCAATGAGATATTCCAATAGTACATATTGCCTCCAAGATTTATAACAATCATAGTAATGGTAATGACCACAACCAAAGATGAGTGTATATCAAAACCCATCAATAGGAATGTTACTATGAAAATGGCCAATACTGATATTCCCATACTTTGCAATGTATCTGGCCACATGGTTAGATATTGTTCGTAAAACACATAAAATACAGAATAGGGAAATACTTCAACTTGTATAGCTTCTTCAAATGGAACTCCATGAGACAATAAACGTCCTT

>BPA_7211

CGGCATCTTCCACCGTAACATTTTTAACTTCAAGTGTTGCAAAATATGAGTGACCATCCTTATCGATACTTATCTTATGGCGAGGGGATTCTTTGACTACTGTTCCATTGTGAAACCATGTAATTGTGGGCTTTGGATCAGCATTTACCCGACATTCGAAAAGTAAACGCTTTCCGTCATCCTCTTGTCGAATTGAAGGTTTTTTCGCGAATGTCGGCG

>BPA_7220

TTGGTGTTTGATTCAATATACGATTATGATCACCTTCACTCAAATCGAATTGGAAGGTGTTGTTTAATTCAATCCATTGGTTTTGGTATTCATTGGTTTTATGATTTTCGTTTTTATGTTCGATATCCGGTGTGGGGAGATTTAACTTTAATGGCACTTCCTCATCTTTGGTTTCATTAAATAAACGTATTTCTTTGGAAGTCATAAAATTATTATTTATTAAACAATTT

>BPA_7222

ATATATTTTATTTGTATTGGATGTTTAAATGTGGAACGTATGCTCAATTCAGAAGAACACAATTTACCCGGGAAACAATTAGCAAAATGTAATTTCTCCTGATCGACTTCCAATCTACCCAGTGCGGTACTATATTTTATATTTACTGTTATATTTTCATATTCTGTTGCTATACTAAAGTATGCCATATTATGTTCCGTTGTATAGGGTTGCACATTAACTGTGTAGACTGCAATATCACCTTCG

>BPA_7231

GGTGGAAACATCTTTAGGCGATTTAAGTGGTTTGGCTGATACGGAAACCTCAAATGTGTTACACGATGATACCATTAACACCAGCTTAAGTCCCCATCATAGTGGTGGTGGTGGTGGTGTTGGTGTCGGTGCCAGTGGGAGTAAATTAAATAAATCAAATTCAGCTTCAAATAGTCCGGTGCCTAAGCGTCACATTAATTCCAGTTCTATAACACGTATACGTCCTCAATCTAGTTATTCAGCAAGAGTATTAATATTTGATGACTCCGA

>BPA_7242

GGCAGTGGGAGCAGTTTTTTTGTTATTTCGGCCACCACCAGCTCCACCAGCGGCTCTACGATTTGCGGGACCACCACGACGATTACCACCGGCACCACCTCTACGATTACCACCAATTTGTGGTCGACGTTTTTGCGGACTGGGACCACCAACAACACGTGCTGGTCTTGTTATGCTACTGGCATCAGTTAAGAACTGTATATTCATGGGTCTGCCATCCAAAGGTACACCATTATATTGCTTAATGGCTTTCAAAGCATCTGATCTTCTTTCAAAGACTACATCGGCAGTTCCTAAAGATCTGCCGGAACGATCATAATGTACGGCCGCCTTCTTCAGCGGACCAAATTCTGTGAACAATTCTCTGATATCCGAATCCGATACACCATAATCCAAATTTGACACCAGCAGTTTGGCCGAATCCGATGTTTTCAAAATACCACGTTTAGGTCCATCATACATGTCATGTTTCCAAGCGCTGTTCACATCACCCCTTGAGAATTTTGCTTTTTGTATGCCTCCTGCGCCTCGGCCTTTAAGAACACCACCGCCTGGTTTACGTGGTGATGTTTGAACTCTACGACCTCCTTGACGCTGTGGTCCTTTGCCAGCTGCACCACCACGTCTGTTACCGCCACCACGATTTCCTCCTTGAGGTTTTCTGTTTTGACTACGGTTAGATTTAATGATATCA

>BPA_7246

TGAGCTTCGCTATTGCTAACCCATCCTCTTAACCTTCCAGCACCGGGCAGGCGTCAGCCCCTATACGTCATCTTTCGATTTTGCAGAGACCTGTGTTTTTGATAAACAGTCGCTTGGGCCTATTCACTGCGGCTGAACTTGCGTTCAGCACCCCTTCTCCCGAAGTTACGGGGTCATTTTGCCGAGTTCCTTAACGAGAGTTCTCTCGCTCACCTTAGGATTCTCTCCTCGACTACCTGTGTCGGTTTGCGGTACGGGTCATTTGTTTCTAACTAGAAGCTTTTCTTGACAGTGTGACATTGAGACTTCGGTACTTTATTTCCCTACTCATCACAACTTGTCCTTAGAGATAAAAGCATTTAACTCTTATCAAGACTTGTTGCTTGAACGTGCACTTCCAGTCGCACGATCTCATAGCCTCCTGTGTCCCTCCATTGTTCAAACAAAACAAACGAGTACAGGAATCTCAACCTGTTGTCCATCGCCTACGCCTATCGGCCTCAGCTTAGGTCCCGACTAACCCTGGGAGGACGAGCCTTCCCCAGGAAACCTTAGTCATTCGGTGGACAGGATTCTCACCTGTCTTTCGCTACTCATACCGGCATTCTCACTTCTAAGCGCTCCACCAGTCCTTACGATCTAGCTTCAACGCCCTTAGAACGCTCTCCTACCATAGAACCAAAGGTTCTATCCACAGCTTCGGTAACATGTTTAGCCCCGGTACATTTTCGGCGCAAGGGCACTCGACTAGTGAGCTATTACGCACTCTTTAAATGGTGGCTGCTTCTGAGCCAACATCCTAGTTGTTTGTGCACCCTCACATCCTTTTCCACTTAACATGTATTTTGGGACCTTAGCTGGTGGTCTGGGCTGTTTCCCTTTCGACAATGGATCTTATCACTCACTGTCTGACTCCCGGATATAAATGAATGGCATTCGGAGTTTATCTGAATTCGGTAACCCAAGACGGGCCCCTAGTCCAAACAGTGCTCTACCTCCATCATTCTCAAATCCGAGGCTAGCCCTAAAGCTATTTCGGAGAGAACCAGCTATCTCCAAGTTCGTTTGGAATTTCTCCGCTACCCACACCTCATCCCCGCACTTTTTAACGTACGTGGGTTCGGTCCTCCAGTGCGTTTTACCACACCTTCAACCTGGACATGGGTAGGTCACATGGTTTCGGGTCTACGACAACATACTCATTCGCCCTATTCAGACTCGCTTTCGCTACGGCTCCGCTTCTTCAGCTTAACCTCGCATGCTATCGTAACTCGCCGGTTCATTCTGCAAAAGGCACGCTATCACCCATTAACGGGCTCTAACTTCTTGTAAGCACACGGTTTCAGGTTCTATTTCACTCCCCTTCCGGGGTGCTTTTCACCTTTCCCTCACGGTACTGGTTCACTATCGGTCACTAGAGAGTATTTAGCCTTGGGAGATGGTCCTCCCGGATTCCGACGGAATTTCTCGTGTTCCGCCGTACTCAGGATACTCATAGGTGTGTTGTCAATTTCGTCTACGGGGCTTTTACCCGCTACGGCTGACCTTTCCAAGTCGATTCGACTATCCACAACAGCTACCACAGCTGAGTCCTACAACCCCAACAAGCAAGCTTGTTGGTTTGGGCTGTTTCCGTTTCGCTCGCCGCTACTAAGGAAATCGATTTTTCTTTCTCTTCCTGCAGGTACTTAGATGTTTCAGTTCTCTGCGTCTACCTTCTAATAGCTATGTATTCACTATTAGATAATATCCTATAAAAGATATTGGGTTCC

>BPA_7247

ATGTTTCGTATCAACATCAAAATAGCAAATTATATCAAATTGATAATAGTGTCGATATTACTCTTTGTGAAAGAGAAATAAAATATCAAATAGGAAAAAATAAAGTCAACAATGAAACAAGGAGTAAAATATATAGTTTTTCTTTGGAAGAGAATGAAAAAGAAGTTCGTTCTGTAATGTTTGGTGAAAGAACGATTGATTTTATGCAAAGCGGTACAAATATGAAGGAGGATCATGTAACAATTGGTGATACTAAGGATGGAAAAGGAGTAACAGTATTCAGCGAATATATGGATGAGGAAAATATTTTTATGCCAGTTAAAGATAATTTTGTAAAGGAACAAATTACAATTTATATGAACGATTCTGTAAAAACGAGTGAGAATGAAGAGGGAAAAACATTATTAGATGTAACAATGGAATTTTCTCCAAATAGGGCTAAACAATATAACAAAGAGAATGTAAACCCAATATCAATTCATAATGCAAACGAAACGGTAAACAACTCATGTCAAATGGAAATCGAAGAAGAAATATATTCAGCAACACGGCCAATAGATTTAAATGTTGAAAAGGAATCACTTGCATTTGAAGAAAAATTAAGTCGAT

>BPA_7258

GTTCTTTGGAATTTTGTTCGGGCATAGAAGGCCAACAATCGTGTCTGTAGGGCATAGATTTAGCTTTAATTTCATTAACAGCTTTGGTGGCTAGATTCTTTAAAATTTGTTGCGTCCAGTGTTCTAATTCCTCCACAGGATTCTCAAATACCGAATTGATTTCCTCAGCATTAGGACCCACTAAGGCGGCATGTAAGTGCAAATAATGAACATTTTCACCCCATTCACTAAGCACCATTTTAATATAGTTAATGGCATTTAATATGGTTATACTTTCCACAGAACCACTGCTATGCAATTGAACCAAACGACGACGAAAATTATCTATTAATTCCAATTGCAAAGTTAAAAATTGTAATTGATGCCCGGGTTGGATAAGAGAATAGTAACGATCTTTTATGGCCTCTAAAAGACGTATAAATTGATCAGCACATTTGGGTATTTTTAATTCATCATCATATATATTGGGATCTATTAATTGCCAAGGATTTTC

>BPA_7260

CAAGCCAGGGTCTATGAAACCCGCTATTATCCAGCCTATCAAAGTCACACTTGAGACTACCACTACAAAGGGTACAAAATAGCCCGCAATTTTATCGGCCAATTGTTGTATGGGCGCCTTAGAGGTTTGAGCCTCTTCTACTAAACGGACTATTTGTGCCAGTGTAGTATTCTCACCCGTGTGTGTGGCTGATATTAAAAGTACTCCGTTTTGGTTGATCGAGCCTCCGATGACAACGGCACCCTTGCGTTTAGCCACCGGCATAGATTCTCCAGTAATCAAAGATTCATCACAAGTGGAATGTCCATACAAAACTTTACCATCTACTGGTACTTTAGCTCCTGGTATAACTTTAAGTATATCACCACGTTGCACATAATCCACGGAGATCACCTTTTCCGAGATAATTTCAAAATCTTTGGAAATTTCTACCAAAACCGCTTCGGCTGCTTTTAAAGATAATAATTTTGATAGAGCTTCTGAAGTTTTGCCTTTGGCTATATGTTC

>BPA_7261

CCCAATTTAAATTCATAATTAAGCTGTCTTTCGCCTTTTTTGTTAGCCAATTCATAATTTCGCAAGTAATCAGTGGAATTTTTACGAATAACACACAAATCAATATTTGAACCTGAACCTAAATCATTGAAGACACCAGCGGCAATTGCATCGCGCACTAATTTCTTGCCTTCTTCCTCACTCATATTTGGTTTCCAACGAGCTTCAAAAACTGCCATTGCAGCTAAACTTCCAGAACCCATAGTTACATAAGGTAATTTATCGGTCGAACCATGAGGATGTATGGAATAGATGTATGAACCAGTTTTATCAACACCACCTAATACCAAAGCAGCACTAATATGACCTTGATACCGGAACAGCATTTGTTTCAACAAAGTGTTTGCTGCCACTACAGGTACTTGGCGACCTGTATTAAGACGGTGCAATTCTAATTGTGAAGCAATCAGATCAGTTGTCATTTCAGTATCAGCAGCTGTTCCAGCACCGCAGCAGTACATATTTTTAGCTAAATAATGAATTTTCGAGCAATTTTTATCTGAAACTATAGGTCCTTCCGTTGCTCGTGTATCTGCCCCCAAAATCACACCATCCCGGAAAACAATACCCGCAATGGTTGTACCTGTTTTTACAGTCTTGGGAGGCTGAAATCCACTTTTTAGCAATTCAGCATTTCTTTTGCAGTTATCGAAATTAAA

>BPA_7262

TCTAAACGATCGCCTTTTTCAAATGATAATTCTTGATCATTATTTGAATTGAAGCTATACAAAGCCACCACTATATCTAATACATTTTCGGCCATTGCATATGTATGTATTTCACCATCATTATCACAATCTTCAGTTGTGTAATTGCTTGGAAACCAGCCCACGGCATTACCACTTTGTCCTCGCCACCAGCCATCATTTGATTTCTCCAATATAAGTATACGTGTACCCTTGGTTAGTGATAGCTCATCCGGTTGTTGTGCTTGATAATTATATTTAACAATGGCTGAGCCAATAGCCTCTGATGGATCTGGGGGCAGTCTTCGAGACATTGTTGGACTTTCGATTTGTCTTGATGGAGAACAATTGGGTAATGTTTTTGAACCTGAACCTTTTTTCACTTTTTTCTTTATACTATCGAATAATGATGGTTTTTCTTTTTTTACATAATTACTGGGGACATAACCCGATTGATTGCGATTATTTTGTACACGCCACCAGTTTGGAATCATCTAATAGTAAATAACGTTCATTTTTTCGTAAATCCAATTCTTGGGCTCCCTGAGCAGCATAATCGTATTTGGCTACAACGTAGCAAACATCTTCTTGTGATTTTCCGTGCTTCATATTGCCCGCCATTGATTGTTGACCACCACAACTGGAATTACCGCTACTACTGCCAGCACT

>BPA_7263

TTACTTAGCAGCAAATTTAATTATGAGGAGCTGGAGTCACAGGTGGTGGTAATTGTTCAGAACTACTGCTACCGCCAGAAGGCATTAAGGATACAATAATGATTATCAAAAGTACAACTCCAATAACACCCATTATGATCATCATTTTCATATTGGCCCACCATTGTTTACGTTTCAATTTACCAGCCTGTTGTTCAAATTGTGAGGCTCCTTGTTCCAAGGCATCAGCTCTTTCTCCCAATTCTGTCAATTTTTGATCACGTTCTAATACTTTTTCAACATTCACTCGCATAATGCCGACAACTTCATCGACTTTGGCTTGAGTTTGTTGTAAACGTTTGTTAGCTTGATTCTGTTGATTGTTATTGCTGGGAAACGTTGATGGATTCGAGGCAGATGGTGGTGGAGGTAAAACGGGAAAATCATTATTTCCTGAAGAGCTGGGTTGAACGGGAAAATCATTAGGGGCATCCATTTTTTT

>BPA_7264

GGGTAAATTGCGTTTTGTAGCCAATTGCTTTCCCCACAAGGGTTACATTTGGAATTATGGTGCTTTTCCACAGACTTGGGAAAATCCCGATCATATTGAACCCAGCACTGGTTGCAAGGGTGATAATGATCCCATTGATGTTTTGGAAATTGGTTACCGTGTAGCCAAACGTGGCCAAGTAGTACAAGTTAAAATTTTGGGTACAATTGCTTTAATTGATGAGGGTGAAACTGATTGGAAAATTATTGCCATTGATGTTAATGATCCGTTGGCAGAGAAAATGAATGATATCACTGATGTTGATAAATATTTCCCTGGTTTATTGCGTGCCACAGTTGAATGGTTCAAAATCTATAAGATACCCGATGGTAAACCAGAAAATCAATTTGCTTTCAATGGTGATGCCAAAAACGCTGCTTTCGCCACTAATGTGGTAAGCGAAACTCATAAATTCTGGCAGGGTTTAATCAATAAACAATTGGAAGGAGGCTCTATTTCTTGCTTGAATACTAGCGTTGCTGACTCACCATTCCTTACAAGTCAAGAAGAGGCTGA

>BPA_7266

TTATATTTTTGAAATACTTTTGCTAATACAGAAAAAAGAAAGTCATCGAAATAATCACTGTAAACATTTTGCGTTATAAGATTAAGCCACAATAGAAATATAGACCCATAATCCGAATAAGGAATCATAAATATATTTTGAAAAACTTTCTTGAATTTAGCATTAGCACTCCAAGGTAAACTACTGGTTCCCATCAGCATAATTTTATCTTCCTTTTGGATCGCCTTTAATAATTTTTTTACCAGAACATCTTTTAAAAGTGTTGGTTTTATATCTATTTGATCTTTTGGAACTTTTTTCCAAAAGACTCTGTGAGCATCTTGTATAAAAATAATTGTAGGTTGATAAGCTTTGGCCACTTTAATTACCACATTTATTAAATATTGCAGATTTCCATTAAACTTGTAGGTTTTCTCTGGACTTAGGTTCATAAAAATAGCACCAAGTTCAGAAGCAATAATATTACACAATAACTTTTTTCCACTTTTTTCCGGTCCAATTAAACATAAAGACTTTGGCTTATCCACATTAAATTTTCCCATACCAAACATACAGTCTTGTATAGCGAATTTTATATCGGCTTTTGCCGCATTTACAGTAACAATATGACTCTCATCCCTAGAATCATCTGCCAAAACATTAAAATCTCCTACATAATCACTGAAACTTTTAATTGCATAATCTTCAATTATTTTCGCTTCTCTTAACTCTTGATATAAACATTCATTCGTACGATTTGCCGTTAAATCTTTGGTTGGCTTCACTTTTATTTTTTTCACTTTCTTTCCCTTTTGCTTTATTGGCTTATATTTCTTTTTATTATCAAGACTCCATGCTTTTTTTAATAATTCATATTCAATTCTCATGTATTCATCAACAAGACCTCGTAGTTCTTTATGAATAATAACAAGTTCATCTTCAGAGACCCATTCTTTAACAAGATCATTATTTTTATTTAGATATTCATCAATATATTTCCAGTCGGTTATAAATTGTGAAAAAGTCTCTTCAATTTTCCCCACATTATTATTGTCTCTCAATTCTTGTCCAATATCACATATTCCTGCATTACTCATCTTTTGTTGTCTTAGCTTCTCTTTCTGCATTTCTTTTTTCATTTTTTCCTTCTCTTTCTTCTCTTGGAGTTTCTGTTTTTCCATCAAATTTTTTTTCTCCTCTTTAGATAAAGCTTCATTCATTTTATATTGCCTAAATTCATCCAAATCCATAGTTTCGTCTAATAACATCTGAATCGTTCCAGAATTTGTGGCATCTGGAAAAGGATGGAAATCTCCAGTTGCATCATAGAATTCTTTAAACCAACTTCTTATCTGATCTGATATATCCTCCATAATCTGCGGCTTATGTTCCTCAAGCAATCGTGTATATTCAGTAGCCATTAATTCGAAGTAAATTTTATCGAATTCTTTTTGTTTTAAAT

>BPA_7276

ACTTCGTCTAACAGCATCTGCTCGACGATAGGGCTCACTTTTAAGACCCAATAAAATATCCTCTAAAGCTCCATTATAGACTTCATCCTGTTGCAATAGTTTCTTTTCTCTAACCGAATTTGCTTTACATTTTAATTATTTTTGGCTTTAAACTAAGTCCATCAGGCCAAATTAATTAGCGTTTTATGTAACAAACTGCTTTCCATAGTCACGCAACCAAGTTTCTGTTGTTTTTTCTGGTTGGCTTTATTTCGCATTTGGACTTGTTCATTTTCTTTTTTATTGGCAGCTGCTGCAGCAGCAGCTTGTTCCAATCTTCTTCTCTGCTCATTCTCCTGATCATGGGCCTTAAATGCTCTGGTAAATCTAACGATTAAAGCAAAAAATGCTGCTGCATCTGCATTACGTGATGATTCACCAAAATACTCTACACACTCCCTAAAGGCCTCTTGGGCATTTTTCAATTCGATTTTAATTTTCTTTAATTTCTCTTCAGAATTATTGAGAAAATCTCTTAAGATATGAGTCTGATGTCCTTTGACTCTTAATTCAGCTTCTTTTTTCACCAATTCCATTCCCTTTTCCAAGTCATGCATATCGGTTACTATATTCTCCAAAGAGACTTGTGACGCTTTATCTGTACAGAAAAGTTCTGTTTCGAAATTGAGTAATTCGGGGAATTTTTGTCTTATGGTGGCTACAATGTAGTGTAGCAAAGAAGAACGTTTATCGGTTGATTTGGTATCGATTAATGTATCCAATGATTGCAATTTGAAGCCATAAGCTGGACCACGTTTGCTGCTATTTAAATAGTTGCCAAAGGCTAAGACAATCTCGAGAACAGCCTTGAATTTGCGCGATTGTTTAAGGGAACTGGAGGCGGTGGTGATGGCATGGATTTGCGGACATATTAAATGCAAACAATCGAAAAAGTTTCCCATATAATTCATAATCGCCAATTTCGAAGAAATTCTTTCCACCTTCGACAGCTGCAACATAAATTTATCCTCTTCGGTTAAGAGATTCTGATCTTTACGTTCAATAATATATTCCTTATACGATTTAATTTCCACATCGGTGGGTACCATTTTTTGCAATAATTCAACATTTTCTAGCGATAATTTTTTCAAATCTAAACTATGAATAGCGGCGATAACCTCATCTATGGGCATATCAAGTTTACGGCGTGAGATAGCAATATTTCGTAATC

>BPA_7277

TTGCTCAGCCAAATCACTGAAACAAGTTACCCATCTATTTGATGTCCAATCACCAATACTCATATTAACCAGGAGTCTGTATTTAAAGTTGGGGAATACAGCATTACATTTTTCGCAACGGTAATGACCATTATTTTCATCCACAACTTTTTTATTACATTCTGGTTGTGGGCATGCTTTGTAGTAAGCATTTTGACTTTTAATAAGATGCACAACTGCTTTAGTTTGAAAATAGTCCGGTTTATCTCCAGTTCCCAAATTACGAGCCTCATGGAATGTACGCCATTCTGTGCTATAACCACCTCCACCGGCTCCAGTTCTTGCTGAGACCGAGTTGGTAATGTTTTCGCCACCTCCATTATCGAACCAACCACGCAATTTATGACATTCTGGTATATCAGGATTTATTTTCATGACAGAACCATTTCCTAAACTTAAAGATTTTCCACCATTAAATTCGCTAACTCTGGCTCCCTTAACAAGAATAACAGGCTGAACATGACCATCGAAGTTAACTGCATCATCACCCCATATGGTTAAATTAACAGCTGCATCACTTGTATCAACCAAGACTAATTCGCGTTTTTTGAATTCTTTATTCGTGGTTCTTGATGTAAAGACCTGCAAATCACTGACTTCTTTACAAATTCCAATGCAATCAACAGCTGCTTTTGGTTCTAAA

>BPA_7288

GATATACATGCTCAATTATTGTTATTGCAAAGACAATTTAATGGTAACAAATTCTTAGAACAACAACACCACTTGCAGCAACAACAACAACCACAATATCTCAATTCAAATGTTTTAAACAACAATTGGTCAAATCTATCTTCACCCCTCTGGAGTCCTATTTTGGGAAATGGTTCATCAGCTAATAGTATTCATCATAATTCGACTACAACTTCCTCTATATTTGGTCATACACCACAAGCTCAAAATTCCACTTGGAGTTTAACACCAAATTCCACAACAACCTCCTCTAATAATATTGGTCTAGTTCGTCCACCACCTGGTTTAGAACAAAATTTTCTTATTGGTGGTAATTTGACAACATCTATGAACAGCCATTCAACTCTAACGTCATCTTCAGCTGGATCAGCAGTTTCTGCTGCTGTTAATGTTTCTTCTGCACAATTGGCTTCAGGTTTTAATGACGCCGTGTCATCGTCTACTGTGTCATCGAATAGTTTAACACAACAAACAGCTCCAGATCGTGATA

>BPA_7293

CATTGGTGTTACCGGCCGTGTTGGTGCCGTACGTGCTGCCCACGGACATGAAATCCCTGCCGAAGAATTCGACAAGCGTTATGAAGATTACTTCAATCGCTCCGACATTGATGGCTGGGAGGTACGCAAGGGAATGAACGATTTGTTGGGTATGGATTTGGTACCTGCTCCTAAAATTGTTGATGCTGGCTTGAAAGCCTGCCGTCGCGTAAACGACATTGCTTTGGCTATTAGATGGTTGGAAGGTGTAAAGGATAAGTGTGGTGATAAAACCGATGAAATCTATCCTTACATCATCAATTCTGTACGCCCCACTCTCACTGAATTGGGTATTCCCACACCCGAAGAATTGAACTATGATAAACCAGAATTGGCTTAAATCTGTTTTCGATATGTAAAAAATATCATATATTTAGCAAAAACAAGAACAATACAATGAATGAGCGTGAGAGGCAACGTAGAATTGATGAAAAATTTG

>BPA_7298

CCGCATTCGGTTTTGTTTGATCCAAGTTATTGTATGATGGAGCAGCAGGTTGAACCGCAGATGGCAAAGATTTTTGTGTGGCTTTAGGAGCAACAGCAGCAGCTGGTGTTTGTTGTGGTTGAACAGTTGCAATAGTTTTTGATTGTTGTTGATTATTTTGTATGGCGTTTAATCTTTGTTGTGGTTTCGAATCGAAACTTTGGTCTCTGTAGTTGAAGATTTTTCAACTTTGTTTGTGGTTGCTGATGTTGTGGTAGATGAAGACTTTTGTTGAGATTTCGCTTCCGACTTTTTATTATCAGAAGAATTTTGTTGCTGTTGCTGTTTTGAAGCACCCAATATAGGTCTAGGACCATTACTAGATTGCTTTTGTTGATGCTGCGTTTGTTGTTGATCATTTGCTGATCTCTGTTGAACTTTGCCTTGTTGCTGTTGATTTGGTTGTATAACTTTACTAGCTAAAGGTGATTGTTGTGCCGTTGTTGTTACTGGTTTTTGTTGCAAATTGGTTTTGAGATTTTTCTTGGAATTTAACATTTCACGTAAATCTTTAGCTTGAGTATTATTTATGGCAGCATTATTACTTACTGCTGCCCATTGATTTTTGTCTTTGTAAAGATTGTGAATGATTTTGTTGTTGCTGTGGTTGATTATTAAAGTGCTTTTGTTGAGACATATTACTGCCACCACTATTACGATTATTAT

>BPA_7300

CACGCATAAAAAGTTCGCGTCCTGTAAGTTTCTTGCAATCTGAAGCCTTTTCTCTTTTGGCTGCTATACCCATACTTTCTTCAAATTCTAATTTCCATTTCATAAATGTTTCCACCGTCACACGTGTACCCTCAAATTTCTTACGCTCTGCTTCTTCAGCTTCTAAAAGTTTACGTTCCGCTTCCTCTTCTTTGGCTTTTTTGTATTCATCCCATCTTTCGTTTAACCACTCTTGGGCTGCACTTACTAAACTGAATATCATTTCCATTCCAATATATTCCTCTATAGATTTTCCTATATGTTCCAAAAGTCTTTGCTCATAGTTGTCTTCGAAATTGATGGGGTCTTCAATTTCCACACTAAAGGAGCTTCATCGGGATAAGTGGGGGTATAAGTGAACACTAAATGACATGCCAAACCATTTGTACATTGCTCTTTATCAT

>BPA_7301

AAAAAAACAGCAAAATGGCTTTAAGCGATGCTGATGTACAAAAACAGATCAAACATATGATGGCTTTCATCGAACAAGAAGCCAATGAGAAGGCTGAAGAAATCGATGCTAAAGCCGAAGAAGAATTCAACATTGAAAAGGGTCGTTTGGTTCAACAACAACGTCTCAAGATCATGGAATACTACGAGAAGAAAGAAAAACAAGTTGAATTACAAAAGAAAATTCAATCATCAAACATGCTCAATCAAGCTCGTTTGAAGGTTCTTAAAATTCGTGAAGATCATGTTGCTAGTGTCTTGGAAGATGCCCGTCGCCGTCTTGGTGAGGTAACCAAAAATCCAAGCGAATACAAAAACGTTTTGCAAAAATTAATTTTGCAAGCCTTGTACCAAACCATGGAAAAACAAGTTATCTTACGCTGCCGCCAAGTTGATGTTGGTCTTGTCAATGAAGTTTTGCCCTCCGCCATTGACCAATATAGGGAACAACTTAATCAAGATGTAGTTGTTACCGTTGATACTGATAATTATTTGCCTGCCGATACATGTGGTGGCATTGAATTGATTGCTTTGAATGGTCGTAT

>BPA_7302

CTCAAAAGATCAAAAGCCAAATGTGTGGTGGTCTCTAAATGGCGAACGTCTGGGAACTTACGATGGTCACCAAGGTGCTGTCTGGTGTTTAGATGTGGATTGGACCACTTCAAAATTGATTACTGGTTCTGGTGATATGACAACAAAGATTTGGGATGTTGAACATGGTACTGTTATTGCTTCAATTCCTGCCAAGTCCTCAGTACGTACCTGCAACTTCAGTTATTCGGGAAACCAAGCTGCCTACTCAACTGATAAAGCTATGGGTCAAAATTCCGAATTGTTTATCATTGATGTTCGTACTGCGGACGCAAGTTTAGCTGATCAAAGCCCTATTTTACGTATACCTATGTTGCAATCAAAGATCACTTCTATGCTGTGGGGTCCTTTGGATGAAACTATTATCACTGGTCATGAAAATGGTCAAATTTCAATTTGGGATATTCGTAAAGGTCGTGAAGTAAATTCGGTAAATGATCACACTGCTGGTATTAATGACATGCAAATGTATAAGGATGGTACCATGTTTGTGACCGCCTCCAAGGATACATCCGCCAAATTGTTTGATTCAGAATCGTTGATGTGTCTCAAGACTTACAAAACAGAACGTCCTGTAAATTCCGCCGCTATAAGTCCAATTTTGGACCATGTAGTTTTGGGTGGTGGTCAAGATGCTATGGAAGTAACAACTACTTCAACAAAGGCTGGTAAATTTGATTCCCGTTTCTTCCACTTGATATACGAAGAAGAATTTGCTCGTCTTAAGGGTCACTTCGGTCCCATTAACAGTTTGGCTTTCCATCCAGATGGCAAAAGTTACGCTTCTGGCGGTGAAGATGGTTTTGTGCGTGTGAATACATTTGATAGTACATATTATGAACCCTTATTTT

>BPA_7305

GTGGAATTGCCAACTACGAGTACAAAACTTTCGAAAAACACATCTGTTAACAGACAACAGGAACATGTAAATTCTGCCAACTCAAAATCGACTTCTTGTGTACAAGACTCCAGTTTAAGAGATAACTACATTCCTACTAGTACAGTTTAAGAGATAACTACATTCCCACTAGTGACTATCATGACGAATGTTCCACTACAAATGCTGGAGATTCCGGTTTAGAAGATAATAATATAAATGTGGAATTGCCCAGTACCAGTGACAAACTCTTAAATGTAGCTTCTGCCAATACAAAAACAAGTAGAGAAAATTCCCTCGATGGTTTAATAGAATGTTCCACTACAAATCCTTCTTCAGATTTGAATTATGCTGGAGATTCCGATTTAGAAGGTACAATAGCAACATCAACCTCAACATCTTCCTGCACACGAAAAAGACATTGTAGTAATGATCTGCCCGCAAGTATACAATCTCAATCTCAAACTCAATGTCAACAACAACAGCAGCAACAATTGTGTACTAAAGATCAAACACGATCTCATCGCCATCATCACCATCGACATCATCATCACAATCACCATCATCACAATCACCATAATCCCC

>BPA_7315

CGACGACCATGTGGATAGAAAGTACCTCCGGGTGGTGTAGGACCCATACGGGCTTGTTCTGTGGTAGGTGAAACTTCCTGAGGTAATGGTCTAGGTATTGAGATATTAATATTTGGATGAGTGGCTTTCCAATAGGTCTCTAAATATTCAGCAATATGTTCACAGGCATCTTCTAATTGATTTTCATCTAGGATAACATCAAACATTTCCGGTGGACATTGAGC

>BPA_7319

AACGGCGATCGTAACTGGAGGTAGCTGGAAGTGGTGGACCCACTCCTCTTTTATCGTAAATGGGTGGCCCATAAGTAGTCCGCTCATATGATGAGCGCTCAAAATTACGACCCCTAACCTGTTCAATATCTTGGGACGACCAAGTATGGTGGGGATGTGGAGCACCGTGATGATGATACCAACCAGGAGAGTCCATATCAAAATTATCC

>BPA_7324

GCTTTACTTTTTGGTGAGGATGTAGGTTTTGGTGGTGTTTTTCGATGTTCTATAAATTTACGAGATAAATATGGCAAAGATCGTGTATTTAATACGCCATTATGTGAACAGGGTATTGCAGGTTTTGCAATTGGTGTTGCTAATGTAGGTGCTACAGCGATAGCAGAAATCCAGTTTGCTGACTATATTTTCCCAGCGTTTGATCAAA

>BPA_7328

CCCGTGCTTACTTGAATTTCTTCACTTTGAACTTAAACAAAATAATAAAGATTTGTTGTATTGGAAAAATCTTGCACAAATCTGTTATCATGAATTGGAACAATTCGCTTTAATGCTGATCATGTTTGTGATATTGGTGAAGATCATTTTTGTGGGTATAATTCTCATGACCAATACACACACAATTCATTGGTATGATGAACTGACACAATTGGAAATTAAAAAAAGGTATGCGGACTTTTTGCTGTTGCGCTTGATTGCCGATATTGATAAAGTGGAGGCGAAATATTTAAGATCGGGCAAAACAAGTGATATCATAACCTTGAAAACGTTTATTTTGGCCCATGAGAATATGAAGCAAAGTGTGGAAAATTTTCGTAAAGATGCTC

>BPA_7332

TGGCAGCTCTACGAATTTGAAGAAAGGTGGTAAAATGCTGAAAAAGGTTAAAGATGGTGTAACAGCAAAAAGATCAAAGAAGGCTGAAAAGCTGGCTGCGGCTTCTGCCGCTGGTTTATCTACAACCGGTAAACCTACTCGTAAGGAAACCAAAAAGAATACAAAACGTAAAAAGGCTGGGTCACATGATGGTGTTGGTAGTAC

>BPA_7333

TGTTTTACGTATATCAATATTTAACGAAATATCTGCCATTACGGTCAATGCCCCCAAACGACATTTAGCATCATTACATTCTAAAATATTAACTAAGACCTCTAGGCCACCAATATCCTGAATAGCAAATTGATTTATTTGTGTCGTTAAATCATAATCCTGTAAACAACATAGCGACACTATGGTAGCAGTTTGATTACCAGCCGATTATTATAATATACAAAAATT

>BPA_7351

TCCGCGCATAAATCTCTCTAATTTATCGATGCAGGCATCCTGGTAGTCGTGTATCCACGTCACACCATTAAAATGACATACACTACGCATGTCTTCAGGTAATCTCTCAGGTTCAGGCCAAAAAAATTGACTATCAATTATGGGTATAATATTACAATTTGAATTTAAAGCGGCTACAATTTCTCTATGTACCCAATCTTTGCATTCATTATCATCGACACAACGTTCGAGAGCATTTGGTGTTAATACTAAAACGAAATTTTTCGCTTGACGAATACTGTTTAGCAAACCATTG

>BPA_7352

ACATCACACTGCTACCACAGAAGCTCAATTAATAAGTTTTGATTCTCCTCCAAGTTCTCCTACATTCACTCAGAAATCCAACAGTGATTGTTTGAGCGTAGATAGTTTCAGTTCAGATTCCAATTTTAGTTCACCCAACAATGGCAGTGTCTCACAACCAGAAAGTGGTTTTGAAGATGATTTTGGCCGTTCTAGACCAGCCACCACCAGTCCCCTAGATCCCTGGGAAGCTCTTGATGCTTTTGGTCATACAGATACAACAG

>BPA_7357

ATAAAGATTCATCATCCACAAAGAAGGATAAAAAATCTAAACATGATAAGAAAAATTCCAAATCCGGTACAAAATCGAAAAAGTCATTAAAGAAAGCAAGTAAATCTAGGCAAAAAGAGGAAGAAGAAGAAATGGAGTACTCCGAAAATGAAGATGATTTTCTTAATGAAGAATCTGAAGAAGAGCCAGATGATGAGGACGATGAAAATTTCGAACTAAAGTCCACAAAGAAGGGTAAATCAAAAATGAAAGAAAC

>BPA_7364

ATATTCCTGATCTTATGGGCTTTAATTTGGTATGTAGTAGATGTGGTACCATCGGGTGTAATAGGCATGTTACCCATTATATTTTTACCCATATTGGGAGTAATGGCCTCTCAAGAAGTTTGCATGAGTTATGCTCGAGATACTCAAATTCTCCTTTTATTGTCCATTTTCATAGCTGCTACTTTGGAGCATTGTAGTCTAGCTAAACGTTTGGCATTTT

>BPA_7366

TGTGAAATTTTTTATCGCGAAAATAAATTAAGATACATTTTAATTGCGGAATGTCCAGATTATAGTCGATATCAGGGAATGCCTGAACCAAATTATTATTGTCTGGAAGAATTTTCAGTGGTTTTTTATCCGAAATGTTTTAGTGTGAATTCGTGTCCCAACACCCAGAAGTTGCATGTGATAATACGTGATGATATGTTACCTAGA

>BPA_7383

CTTCAATAAAATCGCACATATTTGGATCATTATTTTTAGAGGCAATAGTATGGGCTTCTAAAAGGGCCTGATTTACTTGTTTCTCAAGATGTAAAGCTGCTAACATGGCATCTTTGGCGGTAGCAATTTTGTCTGGTGGTTTTTCTATTGCTTCAATTTCTATAGTGCCGCCTCTTTTATTCATATATTCCATAAACATTTCAGCATGTTTTCGTTCCTCATGACTAGCATCATTAAAAAATTTAAAAACGCCGGGTGAAGCTACATCACAACGATTG

>BPA_7386

GGCAGTGTCATAACACCTTGCTCTGGACATTGAGCATCCGGACATGAGATCTCATATGCACCTTCCGATATTTCAAATTCCACATATGCTTTCATACACTCTGTGCAAAACTGACAGCCACACTGGTATAATGTAGTTGAATTGCTGGCATTCTCGACATCGATAAGACACAATTTGCACGTGAACATTTCGAATGGACGTGCTGGTGGCGGTGGTGGTGGCTGTGTCTG

>BPA_7403

ATTTGTGGCTTGGTGGCCACATGTACTGGGGGTTTGGGATTATGAGCCACTTGGGCTGCTTGAGGCGTATAATCTGAATTGCCCTTGACAGGAGCTCCACTCACTGACATGGCATTTACATTAACAGGTTGAGTTAATCCCGTACAGTCCTCGGCATCTTTGGCAGGACGTTCCGCATTTTGGGTTTTGTGTTGAACAATACG

>BPA_7410

TTGTCTATAAATTTGATTTATCTTTTGATGTTGCTGGTGCAACGTTAATGGCTGTCGCTACTTCATCTCCCGAGTTTTTTATAAATCTATATGCCACTTTTTTGACCGAGGGAGATATGGGTCTAGGAACTATACAGGGTTCATCGGTTTTTAATACTTTAGCGATTACAGCCTGTTGCTGTTTATACACCGGAGTGGACTTCTCTATTGATTG

>BPA_7417

TGGGTCCAAACGAAAACGACAAGGAATATCAGCGCGAAATACACCCGATTCTAAAGCTTCCACATGACCACCCACATATGTTTCCGAATCTAAAACGTGACCCTCATTTGATAATTTATTTAGTTCAGTTTGTTGTTTATTAGGATAAACAATATTGGCATGATAAGCCTGGACCATAAGTAAAGTTTCACATAAAGTACCAGAGCCTTTACGTAAAATTTCATC

>BPA_7439

CTTGGTGTGGGTTTGAGCTTTTCTCATTTGAGTGAGGATGTTGGACAATTCTTCACGCTTCCTCTTAGCACGAATGTGTGTGCCCAAGCGGCGTTTCAAGAATTTCAAGGCACGCTTATCCTTGGAAACCTTCAACAATTCCATGGTTCTCTTTTCATAAGGAGCGTGACCAACAACTTCACGTACCAAATCACGCATGAACTTGGTGTGGCGGG

>BPA_7450

CGGAGAGGGAGCCTGAGAAACGGCTACCATGGTCAACAAAAAACACGAGGACAATTGAGGCCTATTGGTGGCCGCGGCAAACAAAGTGGGCGAGGCATGTGTAAAATAACGTTCAGACAATAAATTATAAGTTTCTATGGCCGAATCAATATCCTCTCCATGTATACCTATAGCTACTCTCATTAACATATGTTGTGGTCTCTCCACTATCTTACCATTCATTTTCAGCAGATACG

>BPA_7456

CTATATTAGTCTCTTGTAAAGCCAATTCAAAACGATGATGGGCATGTAAATGCTTTGCGGTATCGCCACATTCATCCATTGCAGTAGATGATTTCTTTGGTGACGTAGCTTTTACCATGGAGGATGTACGATTTTCTTGTTGTTTCATAGATTTTGTTGTATGTGTTGTATGTTGTTCCTTTTCATCTAACAATTTATTCTCTTTACTTTTTAAGGGAGATCGAATTATTTCTTT

>BPA_7470

CGTCGTGCTCAAAAGAATGTTGACTCATATCAACGTTTATTTGGTGAACCCGAACGTCCAGTTACTCCTGCCAAAAATCACATGAAGAGCAACATTCCATTTGGTGCTAAGACTGAAGCAGCTCAAGCTTTGATTACTAATGGTAATGGAAATGGTCATTACAATGGTAAAAGTGGTTCTGTGTCATCGGCCTCATCATCG

>BPA_7487

CTTACAGCGTCAGCAACAACAACAACAGCTGCAGCAGCAACAACAACAACAAATGCTACAGAATCAAAATGGTGAAAGGTCTATAGGTCAACAACAGAATTCGCGCTCCCTTCACTCAGATAAAGAAAAACAAGATAGACATCACAAGAGCAGTCGTAAATCTCCCACACGCATGACATCCAGTAGCCGTTCATCAGGCAGTAGTCGTTCAAGCA

>BPA_7490

AGCTTCTTCTTGAACTTCGCGTCTGAAGACAACCTTGTAACCGTAACCAGCAACAGCACCGCCAAGCATGGGACCCAACCAGTAGATCCAGTGGTGTTCGAAATCATAATTCCACAAAGCAGGAGCAAAGGAACGAGCAGGATTCATACTGGCACCAGTGAAGGGAGCAGCAGTGATGGCCAAACAGGCAATAGCCAAACCAAAA

>BPA_7493

GCAAGCTATACAATAGTGTGGTAAGGAAATAATGTTCCGAATAGCGTATACAGGGAACACCATCACGTTGAAATGTTCTCGGACCATGACAATCAATATCGGGAAAGACAGTACATTGTACTTTGGTGTGTTCCACATCTTCATAATTGAGACCACCAAATTTTAAACAACCATAGCCTTTTTCCTCTTTAGCGGTTTTATTAAGCTTATGATCGATTGGTTTATTGCATTCGATGAAGT

>BPA_7494

CTTTGTTGTTGCATATTCTCAATTTTCATTTCCAAATTGAGTTTTTCATTTTCCAACTGGCGATGTGAGTCTTGAACAATGCGCATTTGTTCTTCCATTTGGGAAATTTCATTTAATATACCATTAATTTGAGCTTTTAATTCATTCTTTTCCATAGTGGCCTGTTGTTCTCTTTGTTGTGAGGCTTCCCTTAAGGATTCCATCAGATTTTCTTG

>BPA_7520

AATATCATCTTCATCAATATTTTCTACATCTTCTTCTACGTTTGCGGCAACGGCTGATTCATTGGTTATTTCTTTACTTTCTTGTGTTTCTTCTTTGTCGTTACTTTGTAAAGAAGCTAATAGTTCGGCATGATCTTCAAACTCTATATTATCTTCTTGCTCTTTTGTAAATCTCTCCGCTTCTTTGTTATCGTCTTTTTCTAAAG

>BPA_7526

TCTTTTCGGATACCGAAGTTACCTCATCGGGCGGTAATGGTCCCTTAGGTGCTGGACGTGGTGCTGGCCGCCCCTCTACACCCATACAAAGTGACAGTGAATTAGAGATTTCGATGCGTGAAAAAGAAACGGATAGTGATTTAATTACAAATTCAGCTTCCTGGAAATGGGGTGAACTACCGACCCCTGAACCCAAAGATCAAACTGATAATCAATCTGCTCAGGC

>BPA_7533

CCAAAAATGAATCCGGTACAGACACAGTTGAAGTGGAATTGGAAGTACTATGTAAGCCCAGTAAGCCTAAGGGTCCTTTAGCAGTTTCTGACGTTACGGCTGAATCTGTACATTTGAAATGGGATAAGCCAGATGACGATGGCGGCGAGCCAATTGAACATTATGTAGTTGAACGTATGGATACTGAAACGGGTCGATGGGTACCCGTGCTAACAACCAAGTCACCCGAAGCTGATGTGACTGGTCTCACTGAGGGCAAAGAAT

>BPA_7551

CGACACATTAATGACTTCCATAATGAATTAGCTAAAATAGTTGAAAAAGCAGCCAGAGTTTTAATATTGAGTGGAAGTTTTGGTTTCGAAAAACGCCAGATTGAAGCTTGTCCTTATGAGTACAAGGCTAGTGGTAGTTTTAAAGAATCCCATCAGGAACAATTAAAATTGGTGAAGTGGAAAGAATTTCAAGGCGAAATGATATTTGGTGGTG

>BPA_7564

AAGTTGACGACGAACAAAATGAAAATAATCAAAGCGAAGAAAAATCAACGGAACCTGGCAAAAAGATGGATTTAATTTTACGCACAACAGATCTCAGTAAAGTAGATTTAATAAAACAACGTATTGAAATGTATAAAATGGCGGAAGCAAATGCTAAAACTGATGGCGAGAGTGCCAAAATGCGACGTTTTAATAGAGGTTTAAAAACATTAAACAATCTTTTACGACAAGCAAATTCCGGATATGATATAAA

>BPA_7583

AGGTTTACTAAACACAAACGTCAAATTACACTTGCCGCAGTAATGACGGTCTTCGTGGGCAGCCATGAAAACTCCAGCTCCACAGTTTTCTCCAGGGCATTCACGTCTCAAACGATGTATTTTTCCATTTTCATCGACCTTATAATATTTTAAAACAGCAAGTTTAACCTTCTTCCTCTTGTGTTTCATTTTCTTGGGAGTGGAGTAATTCTTCTTCTTACGTTTCTTTGCACCACCACGGAGACGCAAAACCAAGTGCAACGTCGACTCTTTCTGAATATTGTAATCAGAGAGAGTTCGGCCATCTTCCAGTTGTTTTCCAGCAAAAATTAAACGTTGCTGATCTGGTGGAATTCCCTCTTTGTCCTGAATCTTGGCTTTAACATTTTCGATAGTATCAGAAGGTTCGACCTCAAGCGTGATGGTCTTACCCGTTAGAGTTTTTACAAAAATTTGCATTGCAATCTAGCGC

>BPA_7584

TTGTTGTTTAAACCAGATGCAGATGCCGTTACTGTTGACGTTGTTGAAGGTGAAGATGTTGCTGCTGCTGTTGTTGCCGCCGCCACTGATGGTGTTACCATTTTGTTGGGCGTTAAAGCAGGCTTTCAGCAGCAATATATTTGTGCTGTATCCTTAATATTTTGTTTATTTTTCTATTATTTGCTACTTCTTTTTGGTGGTTGTTCCTTTTATTTTCTGTATTATAATACTGATATCCTTTAAAACCGTATATAAATTG

>BPA_7593

CACGTTTTATTTTTGGAAAAAATCGTATCATGCAGATTGCTTTGGGAAGAACCAAATCTGAAGAAGTAGAAGTTGATTTACATAAATTATCAAAGAGACTTACTGGACAAGTTGGTTTGTTGTTTACCGAAAAAAGTAAAACTGAGGTTTTAGAATGGGCTAACAATTACGGAGCTATAGAATATGCCAGGAGCGGTTTCGTGGCAACAGAAACTGTAACTTTACCAGAAGGACCTTTAGAAGAATTTGCGCACTCAATTGAA

>BPA_7605

AGGACTCTAATGTTATTGATAAGAAAGAAAAATTTGAGGATTCAACTATAGAAAAAGCAAAGAAAACTTCAACTCAACCGGTAAAGAAGAGCAGTAATGATGAAATAAAATCCAATGAATCCCTACAAAAACGACTACTAGATAAGCCTGAAACTATGAAATCTTTAGAAAGTAAAACACGGGTTCCAAAATCTAACAATGTTGAAATTGAAAACAAAGAGAAATCCAAAGAAATGGAAG

>BPA_7625

TCTGTGGTTGAGGGTGAGGGAGAAGGCTCAGTACGTGACTACTGTACCAAAGAAGGTGAACATACGTATCGTTGTAAAGTTTGCTCACGTGTCTACACACACATTAGCAATTTCTGTCGTCACTATGTAACCTCCCATAAACGCAATGTTAAAGTATATCCCTGTCCCTTCTGTTTCAAAGAATTCACCCGTAAAGACAATATGACCGCCCACGTTAAAATCATACATAAAATTGAAAATC

>BPA_7626

AGTATTTACAGCAGCTGGTAAAGCTGCATGAGCTGCAGCCAATGGATGATTCATAACATCGCCATGAAGTTTACCAAATACCTCATGATAGGTAAACAATTGTGATATATCTTCAATGTTAACTTTATACATTTCAATGCCAGAATGAAGTTTTCCGCTTATCACCACACTACCATCATCTTTAATTTCTGTTTTTGGCGGTCCCAATGTAATTGTCGTAGCTCCCGTGGTAGCTGGGCTACCAGATGTTGCTGCTGCC

>BPA_7646

GGGAGGACCATTAGGACCACCAGGACCACCCATCATCATGGCAGGATTGGGACCAGAGGGACCATTAGGATTCATATTCGCAGCAGACATCATTTGATTTGGATTACCAGCCATACCAGGACCACCCATGCCACCCATACCCGGGACATTGGGACCTCCCATACCGGGCACATTGGGACCATTCACATTTGGACCATTAGGTCCACCAGGTCCAG

>BPA_7663

TTTGTCGCCATGTTCAAACACCAGAACTAATGGAAAATTCGGTTTGTATGGAATGTGAAGGTACCGATTCTTTGTGGATTTGTTTAATTTGTGGTCATGTTGGTTGTGGCCGTTATCAAGGAGCCCACGCTGCAGCACATTATCGTGCCACTAATCATACATTTGCCATGCAATTGGGAACATCTAGTGTTTGGGATTATGCGGGTG

>BPA_7673

TTGGAATTGAGATTGAGAATTATTGGCGAATAGATCATTAAAGGAAGATTTACGCCAACCCTCTTCATATCTACGTTGGGTATTTTCATCCTCTAAAGCTTGACGTCTTTGTGAGCGGGAAGAACTGCTCTCAGAGGAATCTTCTCCGGATGTGCCTATGCCTAGATCTTTTTCATCACCATAGGCTTTATAGGGGGCTCCTAA

>BPA_7678

TTTTTGTTGACATCCCAACGTGCGGCCAATTGTTCACGTACCTTTTGTACTTGCGTTTCCTCAAATCGGGCCTTTTTGGCAGCCGATTCGCCCCCGGCTGTATCACCATCAGCTAGTCTTTTCACTTGGGTTGGAATTTCTAATGGCGCACTTTTATCAATGCTGGGACATGTTGTGGTTTCACCATTAAGATAAGCCAAAAGTTCTTTACGATCAGGACGGTTAACAGCGG

>BPA_7680

CCAGACGCATCACTGATGCTAGACTTAAGATTATACAGAAAAATCGAGCTAAAATACGAGATGCTCGCGATAAGTTGGCAGAAATCACCAGAAGTAATGGTGATGTGCGTAAGAAGCTGATGCAGAGACAGGAATTGACCTCGTATAAATACAATAAAATGGGTAGTGGATCGATGAGTGGTAGTAGTGGGCCAGGAGGTAGAAGAGCATTGGGTGTTAGTAAAGTAATACC

>BPA_7681

CTACAATAATCCTAAAGCCCAAATTGGTTTAAGAGTTTTCGGTAATGATTTACGTCATTATTCCTTAGAAGGCAATTTGGAATTTATGGCATTTGCCAAAGAATTTGATCCTTTCCAACAAATTGCCAAAATATTGTCGGGTCAGGAAATAACTTATACCAAATCCAGTGTTTTATTAGATGCTGCCTATAGTGTTCCCTTAGCTATAGGTCTTCC

>BPA_7687

CATAATATGTATGCACATATGCGTCGTATGCATAAATCTGAAAATATAGAAGAGTTTCAGGTACAACGTAAAAGATCAAATGCCGAAAAGAAATATATTTGCCAGCAATGTCCACGAGCTTATACCAATAAATATAAGCTAATTGCTCATGTTAACAATCATCATAATCCAGATAAGAAACCAGAAACTAAAGAACAGGTGGAACAACCTAAAAAACGTTTTCTCTGCACTCTTTGTGGTCTAAAATTCGATAGTGAGGGCAATCTAAAAA

>BPA_7706

CATGGCCATTAGCACTGGTACTGCAGCCACATCATAATCCAAAGCCAATTCGCTGTGTTCATCAATATCAACTTTAGCCAATTTAACTGCACCTGCCTTTTCACCAATAATAGATTCCAAACGAGGTGTCAACATTTTGCAGGGATTACACCATGTTGCAAAAAAATCTACAATCACAACTTTATCGCTTTTCTTGACTTTATTTTCGAAATCTTCAGAGCTTTGTACTTTGAAAATCTCA

>BPA_7714

CGTTAAGGCATTGGGATATGTTGCTTTACATATTGATGCCAACGGAGAAGCTTTGTCTCAATTATTAATGGAAGTGTCACCGCTATTAATGGATTCAATGCCACTTGTACGAAGAGAGTGTGGCGAATTGGGAATACGTCTGCTATTGGATTTACGAGATCGTTATTCGTACTTCGAAAGATTGATTCCATTAGTTTTGTG

>BPA_7724

ATTACTTATGAGTATTTTACCACCAGCATGGGGGACTGTAGTGGCACGATTTTCTAAGCTACCGTTACTGAATTCCTTTAGTATGGGATTTTCGGTATTTAAAATACTTTGTAAAAGTGAAGGCTGTTTGGTTATGGCATGAAGACGGGCCCTCAACTCAGGCGAGTAATGATTTGTTACAGCCAGTTCATAGTTTTCGATCCATTGTCTTAAGTTG

>BPA_7733

AAATTTGTACCATATCGTGATTCTGTATTAACGTGGCTTTTAAAAGACAATTTGGGCGGTAATTCCCGCACAGTAATGGTGGCTACAATTTCACCTTCTGCGGATAACTATGAAGAAACACTATCAACTTTACGTTATGCTGATCGTGCTAAACGTATTGTTAATCATGCTGTGGTCAATGAAGATCCTAATGCTCGTATTATTCG

>BPA_7741

AGATGAATCAAAATTAAATTCTGGTTCATGGGAGGATCCTTTGCCCTCTACAACAACAGCCACAGCACATCACAACAATAATACCAATCAAAATTTATCAATAGCCCCCAGCGCTCAAAGCGCTAAAAACGGCAACATAACGACAACCACAGCACAATTACACAATCTCACAAATTTAAGACGTCCTGATACTTTAGCAACAGCAC

>BPA_7752

ATAAAAGTGATGGTTGTTTGCCTGCCCCTAAATGGGGATGTGGATGGGCAGCTGGTACCGGTGATTGTAGACCTCCATTCAACACCATATTCATATCGTCACCGGAGCATTGAAAGACTTCAATATAACGATATTTCTTTCCAAAAACCATAAATTGATGATGTTTGCGTTGTGCACATAAACGAGCCGATTCTTCTGAATCCATTTGTATAAAAGCTTCACCACTTGGTTGACCCTGTGCATTGATG

>BPA_7786

CCAGACTGAAAAGTTGCTCCTCCAAGGGTAAAGTTAAGCATCCGAATTACTAGTTCCGTTTTATATGATGTTGTTTCCTGTTTCATGCCTGTTATACGTGAATGTGGATATCTCTTGCTAAATTCGTAGTAAAGAGAATATAAAACTGTCATAGGATATTTTCCATTGAACTCTATACCGTTGTCAATCTCTAAAACTACATTCGCAGCTAGG

>BPA_7808

TTTGCTCTCATTAACGGTAAAGGTATAACCTTTCTTCTGCTTAAAACATGTCTATCCTTTTGTTCTTCGTCGATATCTACAACGTCATACTTATTTTGCCTATTTAAAAATTGAACTACCTCCGCCAATATCCAATTTTCTTCGTCTTCTACTCCCTTTACTAAAGCGGCAACATTATCACCCACTTTTGCAATGTATTGACTTTCTGCT

>BPA_7811

TTTGAAAATGCCATCACCCACTTACTATGTCCTGAAGAAGGAAATCGAATTTTGTTTGTTTGAGGAAAAAAAACAAACTCGTTGTGAAATTCGTATCTATTGGATTGACGATGACCAAGATGAAATTGAAATTACTAATCAGTTGGATTATGAAATTTTTATAGCTAAACTCTCCGATCGCAAACATTTGCAAGTAGCTCCCATTAACCAAGATTTGG

>BPA_7817

AAAATGTTCTTCAATACGATGGAACATGTCAAAGATCGTGTTGGTTACTTGGAAGATTATAGTGCTAAATGGAATAATTATAAATCTCGTTTAGCCGAACTCCAAGAATGGGCCAACAAGGTAGCACCAAAAGCCATAGAAACATTGCACTCTGAAGATCTTACTCCAGAGGAGCGTGTCATGAAGGTTAATGCCTTCAAGGGCACTTTGGCCGAAAGAATGAA

>BPA_7823

AACTATATGCGTTTACAGCATCAGTTGACACCCGTTAGTACCAGTAATAATTTGCAAGCAGCAGCATCAACCGGAACTGGTCAAAATGCCAATACAGCAACTGCTGCCAGTAATACAAACAGCAGTACACCTGCATCGAATGAAAAGAGATCTCGTTTATTTAGAACTTCAAGCAGTACACCGGGTAGTGGAGGTAAAAAGAAACAGTCACAGTCACTTAGTTTAAGTGCTGAACCACGCT

>BPA_7833

TGATGATGAGAGTGGTGAGGAATCCTCGGTAGAGACTAGTAAATCAAAATCATCAACCAGCACGAATGATAAAAAACCGAAATCATCTTCGAATCCTGCCAAAAAGAAAGCTCATCCTTACATTCCGCCTAAGAGAAATAAAGAGAAAAATCTAACAAGTGGCATCTCATGTTCAGCGTCAAGTTCACCCAAAACACCAGCAGAAGAGAGAGTTTCTGATATTGAACGTACG

>BPA_7858

TATTAATAGAGGAGTCAGCAGTTTTAGTTCAAGATGAGGCTGTATCTGTGAAAATTTCATTGACTTCTCCCTTGTTACGAGAATCTCCAGATGGTAGTAAATTTGAAAGTACAGCTGCAAATAATAATGGAACTGGTGATTCTGCTTTATTGCCACGAGAACCATGCCTTAAGGCTTTAGCCGAACTGAGACATGCCAAATGGTTCCAGGCTAGAGCAAC

>BPA_7868

CTCAATAACACGTATTGCATCAACTCCAGAATAATTGAGAAAGGCACAAAGTTGATTTTCGGGATGAATTAATGACATGGCTGTTGCTTCGCCTTGTAATAGATAGGCTGTAAAACCACTTTGGTGACCAATAACCAATCGCATGTCAGAAAGAATTTGGACACATTGCACTGTATAGGCCACAGTGAATTCACACATTTTTTGATGTCTCAAGCGATTGCGATTTATTTCATAGACAATTATTTGCGTTTGATTGGTTGGTTTCTTTAAGGCAACCACAAAGCAGTAAAC

>BPA_7869

TGGCTGTGCAAAATTCTTACACATTAAATTTGGTTCAATTTATATGTGGTTTAGGACCCAGAAAAGGTGCTGCTTTAGTGAAAATATTAAAACAATCTAACCAGAGACTAGAAAATCGAACTCAGCTAGTAACGTTGTGTCATTTGGGACCTAAAGTTTTTATAAATTGTTCTGGTTTTATTAAAATTGACACCAGTTCGCTTGGTGAT

>BPA_7872

ATCTCTTATAACAACAACATCAAATACACATTCATCATCCACCTCCTCTTCAGCCACAAATGCAGCCGGTAATCAAACAAATTCCTCATCATCGTCACATCATAAAACAAATTCGAGTGATAATAAACATGCAACAACAGGAGGATCTACAAGTACAGGAAGTGGTGGACATAAATCATCATCAACGACAAGTTCAATAACGGCCAAAGAAAGAGAAGAAAGATATGCG

>BPA_7875

TGCATGAAGCTAATAATTTGCCTTGAGCATCGAATGCAATATCTTGTACAGAATTTGTGTGGCCCTTTAGACTGCGTTCATATTCACCAGTTTCAAAGTCCCAAATTTTTATAACTGCATCTTCTGAAGCTGATACCATTAGACTGAATATTGGATGAAATATTACTCGTGTTATGCTGGCACGGTGACCTGTCAATGAATATTTTTCAGGTGGCCTAGGTATCCATTCTCCCGGACTACGTTTAGCTTTTGATGGAGCACCTTCTATAACTTCTTTTTCTGCTTCAGAAAGTTTAGC

>BPA_7894

TGTTCAAGTAGCGGACCTTTAATAATGGCTTGATGACGTTGTATTAGTGTGGTTAGATGTTCTACATCTAGAGGTTGATTGTACAACGGCAGCAAATAGCGTTCACCACAGCTGTCTAATATGGAATGTTGTGAGGCGGCCGTTTTACGGTTCGCCAAGTATATTTTCTGAGCCAGCGTTTTAGAACGCCACGAATTGGCAGCATCT

>BPA_7905

ATTTATATGGTCGTTGTGCTCAGGTTTCTCTTACCCAATCACCGGCTGCTGATATGCGTGCACCATATGCGACTAGTGAAAATTCTCAAAGTTGTCAAGCCACTTCGGTTATACAACCAGCTACATTGGAAACTAAACATCGTTGGACTTGTATATCGGGTAATGTTACTTTAACACAAAATTGGACTGTGGCCTCACGTTTAACGGGTGCATCAGCTGCTTTATCAAGATGTGTGGTATTCTCGGAGCATCCTTTGAGTGTGGGCTCACCGTTTGAAATAAAACTTGTTTCACATAATCCTTTATTTGCTGGTTGTCTCAATAT

>BPA_7912

GGCATTCGTCAAACGTAATAATTGTTTTTCATCCAGTTCACTGGGTGGTAATTTACCAAGTCTTTGCACTACACGTTTTGCATTGTAAAATTCACGATTTTTGAATGGTTTCATTGCCTCTCGGTATTCTTTCCACTGTTGTTTCTTTTCTTCTTTGCTGATCTCAAGATTTTCTTCGGGTATCTTTGGTTTGGGTATAAAAGGTTTGCCATCGATTTTGGCCTTAGGATTGGGTATATATATTTTCGGTTCAGTGTTTTTGACAACATCTTCACCAGATTTTTTTGTTTCGTTTG

>BPA_7924

GAACCATCGGATTCAACCGACGATTTATTATTAACAGCCGAATTTCGTGGTGGTCTTACTAATATAAATTATGATTTTGATTTATCTGATCACAGCAATGAGAATAGTGTGGCAGAAGATGTTAAAATCAATTTAGCAGAATTTGATCCACTCTTAAGGGCACCAACGCCACCACCTATTGATTATCAAATTCAACCTTGT

>BPA_7926

CACTGGGCACATTATATCACAACAACAACAACAACAACTGAAAAAGAAATTTCCAATCCAAATAATAAATCTGAAGATTTATCCACTAAAGCAGAAACGTCAGCAAAATTGGAAACAAAAACTACAACAGCAACTTCAACATCATCAACGACAACACCAACTGCAACCGTTACAAGTAATACTACAACTCCTCCTAAAAGACGTTCTCAAACCCCAGTTTCTAATGGTCCGGGTGGTAGTG

>BPA_7935

CCTCTTTAGGTCTATTCTCTAAACGTTGATAACTACGACGGCCGGAACCGAATTTAGCTTGATAGGCTTCTTGAACACGCTTATGGCTATCACTCCAAGTAGTGAGTAAATACAATAAAGGCAAGATTACCAAACACACCAGGGCCAAACGGCACATGCGTCCGCTGCGTATATTGCTGATACGCATTTTTTACAACGCACTTGCTCACT

>BPA_7936

GAAAACTAAATATGATATGGGTCCATCTGAAAATGATAAACTTTTGAAATCTACCGATCAACTGCTAAAATTAACTTTAAATAATTTTCAGGATTTATCCTTAAAGTATTCCAAAAAAGCTAAATCTATCAGTGAAGAACTTTTAAAGGATCCAGCAATTTTAAATGCCAATTCACCACAAATAGCAGAATTTAAAACAAATTTAACCAAATTTGAAGAGAAATATTCAGA

>BPA_7937

ATGTTAATTTTAAAAGTGTACTTAAGACCTTCACAAGATCTTAAACGTTTGGAGGGCATTTGCCGCAGTCCGGTATTTTCTCATTTATCTGCCACTTTATCGGGCATTGCAACCATAAGATCACGTAATCTACAAGATACAGTGGCCAAAGAATTTGATAATTTACAAGATGTTCACAGCAGTGTTTGGCAATTGACCATGGCCTCAAATACCGCTTTGGGTTTATGGTTAGACTGTGTT

>BPA_7957

TTTGGTCTTAGAAATTTCAAGTTGATGTTCATGTTTACGTTTCTCTAAAGCTTCCTTCTTGGCCTGTTTCAGTTCACGTTGTCTCTGTTTCTCCAATGCCTTTTCAGTCAATTGATGTTGCGTCTCAGCTAACACCTCCATTTCAATTTCTTCCTCATCATCTTCTTCCTCCACTTCTTCTTCCTCTTCCATTTGGGGCTCTTCTTCATAAC

>BPA_7963

CAACAATATGAATTTTAATAATAGCAAATATTTTGGTAATGTACATAATGGATCCAACCAAAGTTCAGGCAACATAATGCCAACAACCACTATAACTATAACTACAACAACGACTAATACAATATCCTGCCATAATACTCCACCCAGTTATTCATCATTTGCCTCCAACTTATCCTCAACAGCAACAACAACATTATCCAGCTCATCATTATCTTTATTGGCAAACAAACCGACGTTACAACAAAATTCAATAACATTTGGTGGCTTATTAAAGCAAC

>BPA_7982

AATTGTCGGGTGGTGCTAAAATAAATCGTATTTTCCATGAACGTTTACGTTTTGAAATTGTTAAAATGGCCTGTGACGAAAAGGAATTACGTCGTGAAATCTCATTTGCTATACGTAATATTCATGGTATTCGTGTGGGTTTGTTTACACCCGATATGGCTTTTGAGGCCATTGTTAAACGTCAAATTGCACAATTGAAAGAACCTGTCATCAAATGTGTTGATCTAGTCGTACAGGAATTATCAGCA

>BPA_8016

CGTTGTGCTTGTGCCCACTTCCCTCCGTGCCCTCATCTACGCATTTGGGGCAACATTCATCCTCTAACGTTACAGTATTTTTACAATCCAACATTCGACATGATGGTGCAAAACATCTTCTTTCGCCCTTTTCACATCTGCAACTGGTGCAACGTCCCTCTTTCCAAAAAGCTTCATTCATCACCGTACCCCAGTACTCGGAATAACAATTGTGTTTTGTTT

>BPA_8033

CCCAATTCGGACAACACTTGGGAGCCGGAAGAAAACTGTGATTGTCCAAAGCTTATGCAGAAGTTTGAGGAGTCCAGAGCTAAATCCAAGAAACGCGGTGAAAAGAAACCCAAGGTTGAGGAAATACAAAAACCTCGAGGTTATGAGAGAGGATTACCTATAGAACAAATCGTGGGAGCCACTGATACCAAAGGCGATATTTTCTATTTAGTGAAATGGCAATTTTGTGATGAATTCGATTTGGTCCCCGG

>BPA_8045

TATTACGAGGCTGCCATTGGTAATAATCGTGAATTCCGTAATCGTTCTTCGGGTGCCTATATCTTCCGTCCCAAAACCGATAATGTTCGCACAGTAACTGTTGGTCCCGAAATTACAACGTATCGTGGAGATTTAGTTGAGGAAGTGCATCAAAAATTCAACGATTGGATCAGTCAAGTGGTGAGGGTGTATAAACAAAAGAAATATGCTGAGTTTGAATGGTTAGTAGGACCCATACCTATTGAAGATGGA

>BPA_8047

CACGACCCACTTCTGTGGGTATTGTACCCTCAACACCGGTACTCTCTGGCAGAGATCGTACGGCCTCCATAACTGGACCACAACCTGTTGATAGCATCAAACGCCGTGAAATGGAAACGTATAAAATTCAAACTTTACAAAAAATGCTGGAACAGGAAAAATTAAATTTGGAACGTTTAAAAGGTGACACCAATAATCCCAGTTACAAATTATCTGAGGCCAATATTAAAAA

>BPA_8057

AAGTTCCCATTTAGAGGCCTCGGGTAACAGTTTGTTGGTATTATTCTCTTGCAGTTTAAAACTGTTATCATCAAAATCTGTTTCATACTCGTGTATATCAAGAACAGCATCAAAACATTTTATGTCTGATGATGGTGGAGTTTTTTCATCATGTTCGCTTGATGGCGGCTGGTGTTTGGGTGTATTTTCATTTTGAATTGC

>BPA_8058

AAAATAATTCAATAAGAAAGTTTTTTATTAATTGCAAATACAATGGATTTCGATTGTAAATGTATGCGTGTTTTGGGTACTGCCGCCATTTCCATTGTGGGCTATCAATTGGTTACAAAATTTCTACCCTGGTTGTATATTAATATAATTGGTCCCAAACTATTTGGTCCTAAGGTCAATGTTTCACATTTAGGCAAATGGGCTGTTATTACCGGTGCTACAGATGGTATTGGAAAATCATATGCTAAGGCGTTGGCCAAAAAAGGTTTAAATGTTGTGCTTATAAGCCGATCTCTTAGTAAACTAGAAACCGTGGCAAAAGAAATTGCTGATGCTTACAATGTTGAAACTAAAATTATTGATGTTGATTTCACTGGTGGTCAAGAAATTTACACAAAAATCCAACAAAATATTCAAGGCCTCGAAATTGGTGTTCTAGTTAATAACGTTGGCATGAGTTATTCTTATCCAGAATTCTTTATTGAGTTCAGCCAAAATAATCCACAATTCTTAAGAGACATAATATCGGCAAATGTACACTCGGTACTACAAATGACCGCATTAATATTACCCCAAATGGTGGAAAGGAAAAAAGGTGCTGTTATTAATATATCATCTACGGCTGGTGTTATACCGAATCCTTTACTTACTGTATACAGTGCTACAAAGGCATTTGTTGACAAGTTCAGTGCAGATTTACAAACCGAATACCGAGCCCAAGGCATTGTGGTACAAAGTATTCAACCTGGTTTTGTGGCTACCAATATGACAAAAATTCGCAAAACTTCGGCATTTGCTCCCTCAGCCGATACCTATGTTGAATCGGCCCTTAAAACTTTAGGTATAGCCGAACGAACAGCTGGCTATTTACCACATACTCTTATGCAGTCTACGATTAATATCATCTCGTGTATTTTCTGTGAACAATTCGCAAGTCAATTAGTTTTTAAACAATTATTAAA

>BPA_8092

GTACACGCTCCTCATCATCGGGTTCATTACAAGAATCAACAAGTCCCAAATGTTATTGTAACTCTGATACTTGTAAATATTTAAGAGAACCTTTTGATAATTCTCCCTTAAAATCGAACAAAATGGCAAATACACCCGAAACAGATCAGCCACCAAGAGATCTAGAGGAACCCACAGAATCTCAAGTTATACGCGATTATAAAAATGCTATAGGTGAACAATTAGAAAACTCAGTTGTTAAAAATCTCTTAGAAGAAAATCTTCCACAAAAACCTAGC

>BPA_8100

TGCAAACAGACAGTGTATCTTATGGAAAAATTACAAACAGAAAATTTGGTTATGCATCGTGGTTGTCTTAAGTGTCACCATTGTCACACAAATCTACGTTTAGGAGCCTATGCATTCGATCGTGACGATCCCAATGGTCGTTTTTACTGTACACAACACTTTAGATTACCCGCTAAAGCCATAAGGCCTGTAGTGCGCAAACCAGGACAAAGGAAA

>BPA_8139

CATTTTGTGGAGCATTCATATAAGATGGTGTTGGTTGTTGGATTTGCTGTTGTTGTTGATCACCAACTGATTGATTATAGAGATGGGGATTGGTAGAAGGTTGATTAGAGGGACTATTCATGTAGGGTGGTTGTTGGACATGTTGATGTTGTGGTGATATAAATTGATTAGGTGTTGGTTGTTGCTGTTGTGGTTGGGGAATATGTGAGTGTTGTGTAGGAGTTGCCAAATGATTTGATGCGATTGGCGTAGCTGCTGTAGTAGTTACGGTTGGTGCTTGCTGTGGCATATAATGTTGCTGCTGTTGCTGGGAAGGCAACATATGGTTATAAACATGTTGCTGTTGTTGATTTGGTATATGTTGTTGTTGGAGTTGCTGAGATTCAGCGGCTGCATTTAAATTATTTGTAGTCAACGACTGCTGTGGCCCATACAGGCCTTGCTGTTGTGGTGAATGATTTTGTATTTGTTGTTGTTGCGGAGGCATCATTTGATGGCTCATGTGATGTGGTAGTCCTTGCAGCGAGTGGGGTTGTTGAGATGTTGGTTGGCTACCAACGTGACCATTTTGATACTGCATTGGTATGTTTGACAT

>BPA_8143

TTTTGAATGATGAAATTTATTTGGTGGCAGATTTCAAGTAGGCAATGAGGTCGCCACGTTCATTGGGTTTCTTCAAACCGGCGAAGATCATTTTTGTGCCTGGGATGTATTTCTTGGGGTTTTCCAAGTATTCGAAGAGTGTATCCTCATTCCAGGTAATGCCTTTGGCTTTGTTAGCATCTGTGTAGGCAAAGCCGGCAGCTTGGCCAGTCTTGCGACCGAACAAACCGTGCAAGTTAGGACCAACTTT

>BPA_8148

TAGTAGAAGCATCACAGTTTATAGTTAGCACGGTACGTAAAGAATTGGCAAGGAATATTTCCGCATCGAAATTTTCGGAGCCAGCTACAGATTCAACTAACTCTTCGGAGGAAGAAAATTCTGTTGGCAGCAATGAGCGAAGCAGCAGTCGAGATGAAGATTCTGGTCGTAGCCAATCCGAAAATGCTCGTATACGTATGATAAG

>BPA_8165

TGCACGAGTTTTGGCACGTTTTGGACGCCCCTTGACTAGATGCTGCAGTTGAAATGAAGCACTTGGTAGCTCTGTTATTGAATCACAACATTCATCCCCACCACCATCATCTAAAGCCATTCTACTATCGCTTGTAACATTATCACCATTCACCGATGAATCACGTCTCGACATTTGCGTAGAGGATCGATGCGATGATGGTGATTCAAGCAAATCGG

>BPA_8170

GTATAAAGATCCAAATTTGGATCCAATTACAGCAATTTCAGTATATTTAACTCCTAAAACTTCAAGCATTAGTGGTAATTGGATTGAAATTGCTTATGGAACTAAGAGTGGTGCTGTACGAATTATTGTGCAACATCCAGAAACAGCTGGCCATGGTCCACATCTATTTGAAACTTTTACAGTACATCAAAGTCCCGTAACTAAA

>BPA_8181

TAAATGGTGTAAACGTAAATAATCAACCATTAATAAAAGCAATGCATGTTGTAAAAAAAGAAACCTTAAATCTCATATCCGAATGGGTTTCTAGGTCAAATGATAATCAACTTGTAATGGATAGTTTTATACCTCCATTGTTGGATGCTATTCTTTTGGATTATCAGCGATGTAAAGTACCATCAGCTCGAGAACCAAAAGTGCTTAGTTCTATGGGTGTTATTGTATATAAATTGCGAAGCGACATCACAAATGAGATCCCCAAAATATTTGAT

>BPA_8186

GGATGGGCAAACAAAATTTACTTTTTGGAAGCTTGACCAACCTTGGCTTTGGCGGTACCACGGACCTTCTTCATTCTGTTGCGACGTTCCTTACGTTGTTTGCGGGTTTGTTTCTTGATTTCCATAAGACCATGACGAACCAAACGGTATTTGGGTTCGAATTTCTTGGCAAAGTCCAAGGTATCGTAGATCAAGGCGAAGCCAGTAGAACGACCACCACCGAAAGCAGTGCGGAAACCAAAGACAAAGCATACATCAGGGGTAACTTTGTACATGGCAGCAAGTTTCTCACGAATTTCAGTTTTGCTGACCGAAGCCAATCCAGGGTGGATGACATCGCAAA

>BPA_8200

AGTAGAGCCAGCTGACGTGCTACTGGCCATGATGAATCAATTGTATAGCATGTGGTTAATATTTTTTGAACTTGGCTACGCACTACACCCATAGCATCGGGATTTAGGTGACCACTGATCTTAAGATATGGTCCCAAAGAGTCGACGTGTGTAATAAAGAGACGTTTGCGTTGTTCATTGTTGCTGCCGCCTGCGCTTGAGGAGTTGGTGGAGTTGGTAGTAGTGGTGTTGG

>BPA_8207

ACCATGTCCATGATTATGACCATGACTATGACCATGATTGTGTACATGGTGACTACGATGCCGATGATGACTTTGGTTTGATTGTTGTTGATTGTTTGTATTACTGCGATTGTTATTCGATGATGTAGATGTTGTGTGTGTATGGGGATTATGGTGATTGTGATGATGATGGTGATTGTGATGATGTCGATGGTGATGATGGCGATGA

>BPA_8217

ATTAAGGAAGCTCAACAACAACATGGTTTGCGACACGGCGACTATCAGCGATACCGAGGATATTGTACGAGAAGAATTCGTCGTTTGCGTAAGGCTTTAAAGTTTCCCCAGGGAGACAAGAGACATTTCAAGCGCCGAGATGTTACTTTAGCACAATTGATAGGAAAACGGGCCGACGAACGCTTTATTTATATTCCATTGATAAGTGCTGAGCGTGCTTGGGCTTATGCCATGCAGCTAAAGCAGGAGTCAAATACTGAACCACG

>BPA_8236

CAACGGAGAAAAATAGACCTCAAACTAATGGAAATGTATGCAAAACCACTAAAGTGATTAAGCAAACACAAAAGGTAGCTCCAGATAAAAATAATAAAACTTCAAGTTCAACCCTTAGGCCAGAGATAAAAAATGCCAAAGCCAAAGTCAATTCCTTGCCATCACAAAAGACCAAAAATTCAATTAATACCACATCTTCACAATCGAAAAA

>BPA_8237

GCTGCCAATTATTTGTATCAATCGTTGTTAGACGATGCAATTGTGGGTGTCTTTATTGAGATACATCATTTACGCAAGACTGGTAATCTGGCTGCCATCGATGGTGTGCCAGACGATGATGCTGAGACCTCATTTCGCATTGTTGATATGCCAAATTTTGATATATTTGGCATTTCCACCGCTAAGAAACCAATGGACTGCACCTGCCCTAACTGTGATAGGCCC

>BPA_8245

AAGTAAAGAAAGCTGCAAGAATTGACAAAAGAATGTTCCTAGAACGCCTAGCAACGGCAGCTGAGGAAGTAACTAGTTATTGACCGTAGTGACACACTAATAACTAATCATGATGCACAAGCAAAAAGGGCTGTAATTTCGACATAGATGACACAACTGAAGAAAATGAAACCAATAACATTAGCTCTACCAAACATATGTTAAAATACCATGTAATTCTCCTGGCATAATTGAAAAACTCCTTTTTTAACAGCTTTTACGGTCTCACAACACTTCTGTGTGGGAGGAAAAGAGTCTACCTCCAACATGAAAGAAAGGCATCATTGTCAAGCTACCGAAAAGTGAAATAAAACAAAGATCTACGAGATGAACAGGCAGCATTCCGGCCAAACAGAATGCATTAGAATGCGAAGATCATTCTAACACCCATTAGACAAATATTCTACGGCTAGATATC

>BPA_8248

AAAGAACTGGAAGAGGTTAAAGAAGCTATTGATAAAGCTAAATCAGAGTTTTCGGGTGTGCATAAGGAAATAACGGCAATACAAAAGAAAGAGAGCGATTTGAAACTGAAACGTATAGAAATTGATCAACAATTACAAACTGTTTCTGCGAAATTGTCGGATGTAAAAGCTCAAATACCACATTGGCGGGAACAGTTAAAACCTTTACGCTTACATGAAATACCAGGAGATTCGGAACCCCAACCTCCTCTTAAGACATACACGGAAGAGGAACTAGCGACTCATA

>BPA_8255

TTTATTTAAATCTTTAACTTCAATTTGTAATGTTTCTATGGTTAATGAATTACGTTCTAAATCTTTTTTCAAACTCTCGATAAGATTAATGTGTTCTTCATTTTGTTTAGAGGATTTCTGTTGCAGCTCTTCATATTCTTGTGTGGTTTGGTTATGTTTTGTTTCCAATTCTTTTAATTTATTTTGTGTCAATAGTAAATCTTTCTGTACCGTATCATCTTGTTCGGTTATATAGTTAATATGATCTAATTGACGTTGTAATTCTTCTTTTTCTTTTTCCAATTCTAGTGCTA

>BPA_8272

CGATGTTGATCATTATAATCCAAATAGTGCTTTGTATATGCCTGAAATGCAAAGTTTTTATCCTATGTATCCTTATGCCACAGCTGCAGGAGTTCCAGCAGAAGATTATTATGGTTATTGTGGTTTCGATGCTGGTCTACCACCAGGAGCAGCATTTATGCCAGCTGTTAACAATGGCTTTTATTATTTGTGTAATAATGCTATAGCACCACCCGCTAACTATCTTAACTCCAACAATCCTTATTTTCCACCACCGCCGCCACCTCCCACTTTGGGTGTTGC

>BPA_8288

GCATAAAATTACATGAAGGAAGTAGAATTAATGACGTGATTTTTTCGATTTATGATGACCACCATCTCGACTATCATCTCGAGAACGTTTGTGACTACTGCTACTGTGGCTGTTGCTATGATGATGACGACTCTTATCACGATCCTTATCCTTGTGCGAAGAGGAATGATGTTTATCACGACTACGATCGTGATGTTTATCCTTATGGGAGGATTTTGAAGGGCTG

>BPA_8293

GAACTTTGGAGGCGTTTGTATCAATCAGTTTTTGAATATGATTTGCCACTTTTTAATCCTGAGGTTTGCAAGTTTGTCTTTGAGAAACCGGAAGATTCAGAGTATGCCAATGCTTGGAAGGAGAGTTTCCGTCAGCTTTATCGTGGAGTGCACGTTAGACCTGGTTATCAAAATAAAAAATATCCCGGACGTTCCATTGTGTT

>BPA_8294

CCAGGTTTGGAATTATGTTTTAGCAATTGCACTTGGCTTGAGGAGAAAGTGATCAATTTATATCCCTGGGATATTGGCACAGATGGGGGTCCAACATATACGTCCCCCGATCAGCCACAATTTCCACCAGATGTTATAAGACGCATGAGTTCAGAATATCCACCAGATCCTCGGTCACCGTTTTTCGATGAAAATCATGTGCCCATGAAACCTTT

>BPA_8311

AATCAAAATCTTGTGTACCCAATTTACTATATTTCATCTCCACCTTGCGATAACGTCGGGGTATTATTTTAACACCCGTTTCAGACGATTTAACAGCATTACTAAGACCATTACCACTGCCGATAGACATGCCCACACCATTTTCCATTATATAAGGTATTTGATTACGTCTTAAAGTACGTGGATTTGGAGAATATCCTATTGGTCCTCGCATTTTCATATTATTCAAAATTTCTGGTTCGATGGGTTTGGG

>BPA_8320

CCTCCCGTACCACGTAATATATTCGAAAATGCTCCCTTGAAAAATGCTTTAGAACCTTCGGTTCTTAATATAGTTGCCCAACAGTGTGCAGTATTCTTATATACCATTTTCTCTTTCGCTAGACCCGATTGCATCATCATACGACGTCGTACCGTATCAAAAGGATACGATGTTATGCCAGCAATAGTGGTAACCACTTGGGCTATAGCCCAACTTATATAAAATGGGGTATTTTTGGGATTTGGTAGAAAATCTCTGCAGGTATCATAAAAACCAAAGTAAGTAGCCCGATAAATAATGATACCTTGTACCGATACGCCAAAACCACGATATAAACCAATAATACCATCAGTTTTAAATATTTTTGCAATACAATCACCCAAACCCTTGAATTGTCGTTCACCAGCTCCTTTACCAACATCGGCCGCCAAACGTGTCCGACCAAAATCCAAAGGGTATACAAAACATAAAGATGTGGCACCAGCTGCTCCGCCAGATGCTAAATTACCCATAAAATAACGCCAGAACTGTTCATGTTTATCAATGCCACCCAAGAATATTTGTTTATATTTATCCTTAAAGGCAAAATTCAATGCTTGTGTTGGAAAGTAACGTATAACATTGGCCAAATTACCACGCCAATATGATAAGAAGCCCTGTTCCTTTGGCAAGCGCACAAAACAATCAATTATGCCTTTATAACGTTGAGCCTCACTTATTTGCGTTGACACATGTTGAACTTGTAGCAATAGCTTAACACGCTCTATGGGAGCTACTGCTGTTTTGGCCACCGCTGCTGATATACCTCCGGCCATAAAGTCTTTTAGAAAAGACACGGGATCGGCTAAATCTTTACCCATAGTTTATAGTTTCTTGTTACTTTTGTTTTAGTTTTCTTTCAAAAGTTAC

>BPA_8324

AATTTAGAATTTCTTGAAACTCTTCTTGTTACCGTGGCCCTTGGAGACCTTCAATACATTGAAACGAACAGTCTTAGACAACGGACGACATTCACCAATGGTAACAATATCACCGATTTCAACATCTCTGAAGCATGGGGAGCAGTGAGCGCTCATGTTTCTGTGACGTTTTTCAAAACGGCTGTATTTGCGTACAAAATGCAAATAATCACGGCGGATAACAATGGTACGTTGCATTTTAGTTTTACGTACGGCACCAGTTAAGATGCGACCACGGATTCTTACATTACCGGTGAAGGGACATTTCTTGTCAATGTAGTTGCCATCAATGGCTTCACGGGGAGTCTTGAAACCCAAACCGATATCACGGTAACGGCGAAGTACCTTCTTCTTGGTGATACCGG

>BPA_8329

AATAATTAAACCACCGACAGCAGAATCCTCGGAAGGACGGAATGGAGGGGCTGGTGGAGATTCAGCAGTTTTAATACCTTTCTTAGAATCTTTAGGTGTCTTACCACCTTTGACCGGAGAAACATCTTTGCTCTCAGGAACACCTTTTTTGTTTTTACCAAGAGTACCATCCGTTTTAATATGTTTACCTGCAGCCACAGCAATTCTCTTCGCCAGAGGAACATCATCTTCTT

>BPA_8330

CAATTTCTTCTTGATCACGAACAGCTCGTGTACGGTAAGCCGGTTGAGATTGTTGTTGACCAGCTGGACGACCACTACGTCTTCTTCCCACACGCCCCGTACCATTTGCACGTACATTCCTATTCCTCCCTGACCCAGACTGAGAATTTACAATACTTGCCGTATTAATCATAATTGGACGTCTTCGCATTTCACGTTTATATTCTTGCAATTCATATTGTCCTGCTGTATACACCGC

>BPA_8350

ATAGTCGACTTTTCAATTATATGGAATATGAAGTTTTACGCTTTTTATTGTCTAATTTAAGATGGTGGCATGATGAATATAACTTCGATGGCTACAGATTCGATGGCGTTACTTCAATGTTATACCATTCTCGCGGCATTGGAGAAGGATTTAGTGGTGATTACAATGAATATTTCGGTCTCAACGTAGATACAGATGCTCTGAATTATTTAGCATTAGCT

>BPA_8355

CCTTCAATTAATAATTCACCAAAACCAGCTATTGGATTTGGAAATATATTTAAGAAATCATCAAATGAGTGGGAATGTGACGCTTGCATGATTCGTAACAAATCGGATGTGAATAAGTGTGTGGCGTGTGAAACACCACGCAAACAAGCCACCTTAATAAATCATCAGCCACCTACAACTACTGCATCTGGTACCTCTACTTTTGGACAGCAGTTTAAAAAATCTTCCAACGAATGGGAGTGTGATGC

>BPA_8363

TGCTACCAGTCAGTTGTGGTTGGGGTTGGGGAGTTTGTTGCAATTGTTGTAGATTTGTGTTACGTAGTTGTTTACGATGACCACCACTTTGTCCACCTCCACCACCACTGTTGCCAGAATTATTACGATGATTGTTATTATAACGTCCACCGCCTCCGCCACCACCACCGCTACCGCTTTGCGGTTTTGTGCTGCTAGTTAAACCAGGA

>BPA_8364

TTTCACAATCGGATATGTTACAACGAGAAGTTTATCTATTTGAGCGTATTGATTCGGGGCGTTCAAATGAGCGTATGAAATATTTAAAATGTATTGTTTTTATAAGACCAACTAAGCAAAATATCCAGCTGTTGGCTCAGGAGTTAAGAAGTCCCAAATATGGTTCATATTTTTTGTATTTCAGCAATATTATACCCCGCACAGATATTAAATATCTGGCCGAATGTGATGAATCCGAGTCAGTGCGTGAAGTAAAAGAATTGTATGCCGA

>BPA_8383

GGCAACTCCTTCAGCTGGGACATAAGAGCCTCCAATTGTTGACGTAATTTGCGTGGCTGTACAGACAACCAAGGTTTTTCGCGAGTTTCGTCAATCTGGGTCCAAACTTTGGACAATTCACTCCAAACTCCACGCAAATCTTGCAATTCTTCCAAAGCAACATTCATACGCTCCGAACTGTTATTGGGCACGGCAGATTCTTGCAATTCCAAAG

>BPA_8392

TGGGTCACACGGCCAATCATGCCATGGTGGTGGCTCAACTTTACATTGATGACGAACACAAGGGTATACAAATGTTTATTGTACAAGTTCGGGATCTTGAGACTCATATGCCTATGCCGGGTATTGACATTGGTGAGATAGGTAAAAAAGTGGGTATGGCTGCGGTAAATCAAGGATTCTTGGGTTTAAAAAATGTGCGCATACCACGCATGAACATGTTAATGAAAAATGCTAAAGTTATGCCAGATGG

>BPA_8404

GTGTTGTTTCTTTGGTTCAATCGTTTTGGTTGTTGTTATTGTTGTTGATGTGGGTTTTGTAGCCTCTGATTTAGTCCTACGGTGCGTGGTAGAATTTGCATTAACACTGTTACGCTCTGATGTTACCGTACTTTGGGAAATTCCTTTTAGAGCTTGTGGAACATGTGCACGAGAATTTGATTTCAAAACTCGACTTGTTCCTTCCCTTATATCATGATTGGCCTGTACACCTCGTTCTATGGTCTGCACAGCATCATGCGTTTGTGTAGCTTTATACA

>BPA_8405

ACAGCTAACCAGATACAAACAAATAATATATATGGAAATTCTTCAGTATTTGGCAACCAACAGCAGCAGCAACAACAAAATTCCATTTTTGGACAGACAGCAAAACCAGCAAATTCCATATTTGGCGGTGGTGTTAATAATAATCAACAGAACACTTCTATATTTGGTCAACAACAAAGTCAATCTAATCCTTTTACTCAACCACAACAACAGCAGCCGCAACAAAC

>BPA_8424

GCTATAAGGCTCATAAAACACCCGGTCCATTTATATTCTTCTATCAGTTACCAAGCTCAGACGTATCTTCTTCGAATCAGCTAGTGCCACAAACAACGACGACGCTAAATCCAGACGGAACTTATACTACAACTACTTTACCAGCTCAACAAAGCCCGCCTACTTTTCTGAAAGGTCCCGAAATATGTGTTGACACTCGAACGTATGGCAACGAAGCAAGATTTGTACGCAGGTCATGCCGTCC

>BPA_8430

GATTGGCCTCTTCGAGTATAGTTATTAGTTCGGGTGCCATACCCCAATCGGAACGCGTTATATAACTAATAGAAGTACCAGTGCGCCCAGCGCGTCCTGTACGACCAACACGATGAACATATTCTTCAATATTACGGGGAAAATCGAAATTTATAACATGTGTTATATCTTCAATATCTAGACCACGTGAAGCCACATCGGTAGCTATAAGTATTTTTACTTCACCAGAAGTAATATCAGCAA

>BPA_8460

ATGAAACTATAACTTTAACTAAAGGTTCGGGTAACAAGCAGCCTAAGGTACCCAACATACGTAACCAATTTGCGCGAATTTCTGGCATAGTACAATTTTTAACACCATCTAACATGAGCTCTAAATCATTTTGAGTCATTTGATTAAAAAGTTTCTTTTTTGTTTTTAAATGTTCTAGAGCTGCACGCATTAATGAAGTAGATGGCTCAAGTATCGAAGTGTCCTTAGGTCCTTTAAACACTTGTTGCCCTAAATCGACCCAAACACTATAAATGGCATTGT

>BPA_8464

GTTAAACGTTCCAAACATTATATTGTCCAATCACCACAAACATCTAAAGCCATACTCGATGCCAATCAACACTCAATATCATACACATTATCAAGAAATCAAGCTGTTATAGTCGAATATAAAGAAGATACAGAAACTGATATGTTTCAGGTTGGTCGATCCTCAGAGTCGCCCATTGATTTTGTTGTAATGGATACACTGCCTGGTGATAAAAA

>BPA_8470

GCATTAACAATATCATTGTTATTGTTCTTAAGTGCCTTAATAGCCTTAGCGCGGGTAGTATTGGCCTGGTTAATGACAAGCTCAATGTCCTTTTCATCGACACCGGTCTCGTCAACTTCTTCTTCGTCTTCTTCGGCAATGGGTGCAACGGATGATGTGGTACCAATGGTATCAGCAGCACCAGCAGCTTCGGGAGCCTTGAATTTCTCGGCGGCAGCAACTTGAGCTTGTTGTGACAAATCTTCAATTTTAGCTTCACCGAAAACAATGTAAGTATCACTGTGGGGGTTCTTGTATACATCAGGATTGTTGATGACAAACAAAATGTTCTTGGATTTGCGGATGGTGACACGGTTGACACCTTGGATTTGTTTCAAACCCAATTTGAGCATAATCTTGCGGGCTTTCTTTTCACCACGAGATTGCTTGGCCTTGGATACAAGGTCAATGGGTAAACCGGTGGCACCACCACCCAATTGGGTCGTACCGCCACTGGCATCTTCCAACTCGGGCATGGTGTCCTCTGAGTCACTATCACTGCCATCATCCTCTACACGAACAGCTTCAGCGATTTTAGCCTCAGCGGGAGCAGCAGAGGTTGTGGGGGCATCGGCCTTAATTTCAGTAAGTTCTGGC

>BPA_8472

TTCATCCAGCTTTCGTTTATATTTTTCTAAGCGCGGTTGTATTTGGTGTATTTCATCACCCAAAGCATTGATTTCTTTATCGACTTTACCAACAACTTCTGTGTCTAATGCTATCATATTCAGTGATCCATTTGATGAATTGCAACTTTTATTTGCTGGTTTTAAATGAGGTGAAAATCTTTGAGAATTTTCTACTGGATTTTCCGACATGCTTATCCAGAATCTTGG

>BPA_8486

TTTGTTTGCATTGGAAGAATGGATCTCCAGAATAGCCAGCAGGACAAGTACAAGTTGGTACATGATTATCGACTGTACAAATGGCGAATTCACCACATATATTATCACAAGGATCCACACATTTGAAACGAATACAGGCTTGAGCGGGAGAACAATCATCGTGGGCCTCACATTCACGACGACAGCCACGTTCGACATCATAGGGGTTACC

>BPA_8497

AGCGTTGACTAACTGATACTAAACCAGGTGTTAAACTTCCTCTATGATGACCACGACCCACAAACAAAGGTTCACCATTTCGAGATCGTCCAGTAGATATAGCATTGGGTGGAATAATATGATGATGGGTTTTAATCCAAGCATAACCATAACCACATAATACCTCAAAATTGCGTTTATTTATTTCGTAGCCTCCATAACATATATAAGCACATCCCTTACTGGGTATGAATTTACATGGCAGCATTTCACCTTCATGCATTGCTCTACCAACATAAATAACAGCACGATCCGTATCATGCCCCGCAATAACTGCACCGGGAGGTGTGTAGTTAGGTGTTGAAGCAACCCAATTTTCGCGATTTTCAAACACTAAGACTTCAAATGTATTCAAACGTATTTCCTGACCACCGAATGGTATATATAGACAACCATGCGAAGGTACTATTTTACCAACGCACAAACTATTGGCATGATTTCCTCTACCAACATACAAGGATTCTCCAGTGCATGTCATTCCTGTGCGTACCGCATTTGGGGGGACAGCACAAGCATAACAGGGCTGCCAGCTATAACCTTGTCCCACTAATATTTCGTAATGTGTCTTAACGTGTTCAAAACCGCCCCATGCTATATATGCACATCCCTTACTGGGCACCACTTTTGCAGGCATATT

>BPA_8523

TTGATACCTTACCGAAAGGAAGTGTAATAGGCACTTCATCACTGCGGAGAACCGCTCAACTACGTAGGCAGTATCCAAATTTAGTTGTTTGTGACATTCGTGGAAATCTTAATACTCGATTAGCTAAACTAGATGCCGCTGATTCAAAATATGCAGGAATTATTTTGGCCCAAGCAGGTTTGGTGCGTATGGGATGGCAC

>BPA_8524

TACATACGGGACATTTAATTCCATTAAACGACCATATTTGTGATGGAAGTGAAGTTGCTGTATACACACGTCCGATACTATTAGTTGCACCATTTGAGGCTGATTGAGCTGCTAAAGCTATACTTTCTACAAAACTTGCTCCAGCCATCGAAACACCACGTAAGTTATCACCCAATGTATGACGTTGCAAGTGATTGTTGTGACCTTGTTGTTGTGTCGACATTGTCG

>BPA_8525

GTCTGCCCAACCTCCTCCCGGTTCGGCTCCTGGAGGACATCCTGAAAATCTGGCCACTTTATCAGTGGTGAATTCGTATATACCAGGACAACACTTACCTCCACCACCTACACATGGTTTGTTGCATCATCAACAACATGATATTAAGGCTACCATTACGCCAGTCTCTAAAGTGGGCAAAACTACGACTCCTCCTCATGTGGTGGTACCCTCTCCTGATGCCATGTTATATGC

>BPA_8533

AAAGATTTCGCTGATTGTGTTCTCGAGAGTACTGGTGGTGTATCTTCTAATTGTGTTTTTATACTTTCTGATTTACTTTGTAAACTGACCATACTTCCATTGCGTTCAAGTTTTTCTTCCTTCTCCTCAGTTTCTTCCACCACATTGTTTGCATCTACCACATCTTCCAATTGTAAAACTTCTTCTCCTGTTCTCCACTTAGCTGGTGATTCACTTGTAACTTCTGCAATTTCTGC

>BPA_8576

GCCTACAAAGCCAATAATGATGATGACTATCAAAATGAAGATGACTACAATAACAACGATAATAATTATAAGAAACGTTTTAATGGAATTGGTGGAGGCAGAGGACGCAATAGTGGCGGTGGTCGTGGAGGGGGGCGCGGCGGAAATGGTTTCAACCGTTATAACAGAGACAATGACAATGAAGAGAATGGTGAATCCAAC

>BPA_8592

AAAAAACCAAGAAAATCACCATTTCGGATCCCATCCAAATGCCTGAAGTTTACTCATCAACACCCGGTGGTACTTTGTATTCCACCACACCTGGTGGTACCAAATTGATTTATGAACGTGCCTTTTTGAAAAATTTGCGCCAATCACCCTTAAGTCAATCACCACCCGCCAATATTCCCACCTGTTTGATGAGAGGTACACCACGCACACCCTTCCGCAAGTGTGTGCCACCACCCTCCGACCTAAACAAGAAGGTGGAAT

>BPA_8598

CTCCACAAGTGAACCACGTCAATCTGACGTCCTACGCTTAGAAGCTGAATTAGTAGAAATGCGACCTACTTTGGACGCTGTGAATTTAGTTGGACCAAAATTATGCCAGCTTTCCCCAGGCGAAGGAGCATCTACAATTGAAAATATAGTTACTCGTGATAACCGCAGGTTCGATGCTATTGTTGAACAAATACAACGTAAAGCAGAACGCTTGCATTTAAGTAACC

>BPA_8613

TCCGCTTCTTCAACGTCGGCGGCTACTTTGCAACCACCGACAGCACTAGGAAATTGTAGCAGTGGTGGTACAAATAAAAATGATACAAATACAGGAGCCACTTCATCAGTGGTGGCAACAGCTGCAACAACCATGTTGTCAGATGCATATCATCATCAACAAATGCAATTGCAATTGCAACAACAGTTTGAACAACATTATCAAATACAAAACTATTATCATCAGGCTCAACAGCAGCAGCAATTACAACAATATCAAGATTTTTATGACCAACAATTGTATTTA

>BPA_8615

TGAGTTGGTTGTAAATTGTGTTGATTGTCCGGACCATTCTATGCGACCTCCAGCAGTAGCTCGTGATCGATCACCACGGCGAAATTCAGGTTCTGCTTGTTCAAGTTCAGGTAGTTCTAAGTTTCCGCTATTACTTCGCCTTAGTTTACTAAAAATAGTTCGCAAACTTTTTGCAGACCGTTCTTTTATTGTTGGCGAAGGTGTTGAATTGGTATTTAAATTACATGTATAGCTGTTAAACATCGAATCCGAAGAA

>BPA_8629

CGGTGATATGGGCAAATCAAAAGCTTTACTGAGTTTTGCAGATGAAGAGGAAGATGCAGAAGTTTTTCAAGTTCGCAAATCATCACACAGCAAAAAAGTTATGCGTATGTTGGACAAGGAACGTCGCCGTAAAAAGAAGGAAGAACGTAGTTCTGATAATAATATTAATGCCAGCAGTAGCTCCTATAGTAACAGCGGCTATAATGCCAACAACAATGATACTCATAA

>BPA_8634

GAACAATCGGAATTCACTATGTCTAATGAAGATTTCCCTGCGTTACCGGGCACTCAAAACTCGGATGGTACAACGAATGCGGGAAGTGGTCTAACAGATAGTAATAATTTGGATGGTACGGATAAGACAATGAATTCAATTGTAGCCAACAGTGGGTTGGGAGCTGTGGGTAGTGGTGCGGTAGGATCAGGCTCATCAGCAGTTGGTGGTGGTGGTGTCGTTGGTGGTAGTAGTGGTCAAGGTCAAGGTGTTGGTGTAGTTGGTGGCGGAGCGCCTGGTAGTACAGCAGGTGGAAATTTTAGTGGCAG

>BPA_8635

CGCCTAATAGTTACTATGCGTTTGGAGGGATCTGTGGTATTATTTGTGGCTGCGTTTGCTGCCATTGCCGCAACAACATCGGGTGATAACTGTTGGGGATGTTGTGGTTGCTGTTGTTGCGGCTGATGAACTGATGCTACGGATGCAGTCGGTGGTGGAGCATAACACATTGGAGTGGCACGCATTAAACCAAAAGGTGCAGGTGCCGCC

>BPA_8639

TGAAGATGAACCACCATCATGTCACTCGTCTAAGGAAGAGTGTGATCCTACATATTTCAAATGCAACAACTCGAAATGTATACCAGGCAGATGGCGCTGTGATTACGAAAATGATTGTGGAGACGGCAGTGATGAATTAAATTGTCAAATGCGAAATTGTTCGGAAAGTGAATTTAGATGTGGTACCGGCAAATGCATCAAACATGATCATCGT

>BPA_8651

TTGTACGGTTACCTCAACTTTAGAGGAGGGTTGTTCGTAGAGACCTTGTCCCAGACGAAAATATCGACTTGTGTTAAAACTGTAGGGTGACATAAATGTCGAATGTTGGTGTTGTGTTTTATGTTGCACTGTATGATAATCATGGCGTGCTGCCACTGTAACTGCAGCTAAACATTGATGATTTGTTAGAAGTGTATGTCGGCTGACATAAAATTGAG

>BPA_8655

GGTGGAGGAGAATTAATATTAAGACTGCCAGTACTGGCGCTTAGAGCAGATTTTAATTTAGCCGCTTGAGCCACAGCATTGGCAGAATTCAATAAATTGCCCGAAATAAAGTCCATATAGTCGCCTCCTATAAAGGGCTTGGTATCAGGACCTGCACCATTTTGCTTCAATTTAACACGTTTATTTATCTTTGGCTTGTTTTGTTTAGCAATTGGTGCCAATTTCGGTATACTGACCATTGAATTCAACAATGTAACAGTAC

>BPA_8661

GACAAATTGAGGGGTAAATTTGCCAAGGGAATAACATTATGAGCTGCTGTAGTATTAGAGGAGGAAGAGGAGCTTGAAACATTTGCAGGATTTGTAGGTGTTTGCTGACGATGTTGTGTGGGTGTAATGGTAACAATACTTCTATATTGTTGATACTGAGCTGTATTACTATTATTTACAGCAGCTGGAACAGCAGTTGTAT

>BPA_8675

GGCAGACAGAAACTGTATTGACTATAAATGGCGGCGAGTATAAAGGTTTTATTAGGGAAGATGGCTCATTCATTATTAGTTCAGTGCCTTCGGGAAGTTATGTAGTGGAAATACATAATGCTGACTATTTCTATGAACCAATAAGGGTAGAAATTAATCCAAAGGGCAAATTCCGTGCCCGCAAAGTAAATTATGTACAACCCTCACAAATTGTACAAG

>BPA_8678

CTCAAACGTATATTGGATTCGAATAAAAGAGTTCAAGAAGCTGCTTGTTCAGCTTTTGCTACATTGGAAGAAGAAGCTTGCACAGAACTTGTACCATATTTGGAATATATTCTACAAACTTTAGTATTTGCGTTTTCGAAATATCAGCATAAAAATCTCTTAATTCTTTACGATGCTGTGGGCACACTGGCCGACTCTGTGGGTCATCATTTAAATAAACCACAATATATTGAAATTTTAAT

>BPA_8679

ACTGAAATGTTAACGGTACGTTTTAAACTTCCCGAAACTTGTGCTCAATTCTTGGAGGCCATCCAGCAGGCTCAAGCTAAAATGAACAGTGATGGTGAGGAAGCCAACTCAAAAGCCAATGAGAAGAAAACGGAAACTAAAGAAACTAAAGGTTTTGGAGATGCTTTTAAACCTAAAGCGGGTTCTTGGTCTTGTGGAGCTTGTTATATATCAAATACGGCAGATCAATTGTATTGTGTTGCCTGCGATACCCCAAAAGATGATACAGTACCCAAAAAA

>BPA_8681

AAGATCAAATGGAGATGTATGATTTGCCTCCCAACTGGCAGAAATTAGATTCAATTGCTCAAAGGGGAATAGTAATGAAGTTGATGGATCAGTTGGAAGTTTCGAATCGATCATTTCGTATGCAAGCTGCTAGATGTATATTGTATTTGGCACAAGGATGTTGGGCAGAAGTTCAATCGGACGAAGAACAACATCAAAATACTAGAGACAATGTGATTGTGCTGTATGAATTGGGAGTATTTTCGTCATTTATTGATTTACTGAACATGGAAATTGAGAGTGCTTGCTCACCAGATATAGTGGCTGTAAAAATTACTAATGTTACATTGGCTGATTCGGCAGATTTGCGAGTAATTTTGTCAGTCCTTTACACGATTACTGAGACTATTCGTAATGAAAAGGAGAAAGATAGTGAAGACTATCGAAAGATAGTCGAATCCTTTATTCAAGACATAAATAGTCTTTTGCCAGATGGGGAATTATTATCGGTTAAATTGCTTGGTATGATAACTCGTTTCTGCAGTGGAGCGGCTCCACATTTTCCGATGAAAAAGGTAGTACTATTGCTTTGGAAAATATCGCTAATGGGGTTGGGAGGAATGGAAGTTCTTAAAAATCTGAAAAACGAATATCGGATCAAAGCAGGCTTAGAACCGGTATCAGAAGACACGCTGGAAGTTACAAAATGTATGCGAGCCAGTTCTCCACCTGCAACTGCAGCTGATTACTTGGACA

>BPA_8683

ATTTATCCTCTAGTTTTTCATTTCGTTTCTCTAACATATCACCCACAGTTTGCCCATGACCTCCACCCCCATTTCGAGGAGCAGGTAATGGTTTACTTGGAGATAGACTTTGTTCATTGAATCTTTCTAACATTTCTTCCATTTTAGCTACTATATCTTGTAACTTATCGGCTCGAGCCTTTTGTGTGTTTTCATTTTCTTTGGCCATTTCCAAATCAGCTCGCAATTGTTCCTCAATAACTCGTATATCGGTAAGTGAACCGGGTGCTGTTTCATAAGGAGATGTCG

>BPA_8687

AGATTGGATATGAACACTACATCACTTGATACCGATGAAATGGATCGAAGTGGCATTGATTTAGGAGCAACTGGTCGTCTCCAGTTAATGTTTAAATTAGCTGAAGGTGCAGGATTAGCCGTTCCGCAAGCAGCAGCAAATGCTCTACTGGCAACTGCTCCACAGCCGGCTCCAGTACAGACCCAAGCACAAAATCCACCCATTGCTACACAATGCTTTATGTTATCAAATATGTTTGATCCACGTATAGAAACAAATCCCAATTGGGATACTGAGATAAG

>BPA_8691

TGGTACTGCTTCCATCCGCCATAGGTTTAACATCTTTAGTGTCACCACTTGCGGAACCTGCTGCGGAATCACCACCGGTCGCACTCGATGTAGCATTTGATTCTTCAACTTCCATTGGTTCGGTTTTAATCTTAACATCACCATCTTCGCTTTTGATTTCCAATTTAGCGGTACCGTCGTTGTTCACATTTTTACCAACTCCCGCAGAAGACTCTTGTTGAGAATTATCTCCTCCACTACCATCATCCATAAACTCTTTCTT

>BPA_8694

TTCTGAAGCATAGCCATCCAATCTTTGCTACTAATATTGGTCAAGGCTTCTTTTTTTAATTCTTCTGTTTCATATTTAGGCTTGTCTTCATCTTTGCTTTGTGACTTCTCTTCAGTTTTAACATTCTTTTCCATTTCTGACGATCTTCGTTTGGGAGCTACTCTTGCTGCATCTTTTTTTGGCGACTCTTTTCCAGCATCTATTTTCTCTTTA

>BPA_8730

AAAATATCCTCAAAATAGTAAACAACGTAAGATGATCAAAAGACAATATAAAAATCACAATCACTATCAGCAGCCCACACACTATCAACGAAGACATCACGATGGTCATCATTGTAAAGAACAAGGAAAGGAAATTTATTTTAGTCCTGCATCATCATCTGTTATTACAGTACGAACTAGTCCCATATCTCCTATTTTGCAGGCACCAG

>BPA_8739

AGACCGTTCAGTATATTCATTGAGCTCTCCAGTAGCATCCATTTCAAAGTAGACCAATTCACGACCGGATAAAGTAATCACAACTTGTCTTTGATTGACCGCACATTTAGTTATAGATTTTTTGCCAGGAGCTCGCCATTCATTAACACGTTTATCGGAACGTATATGACGTATACCATCAGGATATACTTGCACCAAAGCATCTTCACCCAATGAAGCACAACACAAAGTGGGTGTTGTACCCAAGAAACCACTA

>BPA_8740

CTACACCAGTTACTTATACAACTTGTGCTATGGAGGAGATTTGGGACGAAGAAATCTTAAAAGAGTTGTTGGAACAGGCTAGAACTTACGAAGAACGTCGTAAAATACGATCACGTTTGAGGGAACTTATGGCTGACCGTGAAGAAACTAAACAAAGCCCCAAAGGCGAAGAAAAAGAAGCAGCATCAGCAGCAACATCAGC

>BPA_8761

TTCAAAGATTTCGATTTCTTGGAATATGAAGACAGTATTGCTGGTGAATCTACCGATAACTTCAATTGGGGTGTTAGACGCCATCAATTGTTTGAGGGCGAAGAGGATATGTTTAATTGTGGTAGTGGTATGGGTGGCGGTGGTGGTACCGGTTCTATTAAAGGTGGACACTCCTCAGCCTTAGAGGATAGTTTCAGCGATAAAACACCTATTTTGAGTAAAC

>BPA_8773

GTGAAAAGCCTCATGCCTGTTCCCATTGCGATATGCGTTTCATACAAGTTGTGGCATTAAAACGTCACATGAAAGTCCATCAAGAACGACCCACTTACATACCTCAATCTGCCTTTAAGGCCAAAGAAGAACTGGTTAAACAAGAACGCAAAAAGTATGCTATCAAAATTGAAAAGGAACAGGAACGTGAAGCAATTGCTGAAGAAGCACGCAAACAATTGGAAGAATTAAAACG

>BPA_8777

CGTTAATGCTATTGCCAACAACTTGGTTAGCATGGAAAAGTCATGCAATTTCATTTCTATTCGTTTGTATATTGATGCTATTGCTAAGGCTCACGGTGAAGACTCAGAAGCTGTCAATGAAGCTTTAGAGCTTTTCTCTACTGCTGTAAAACATTTGAACAATGCTGCTCAAAAATCTAGCAACAATGAAGCCTTGGTTGCA

>BPA_8786

TCGCAGGGTGTACAGCCTGCCGCTGATGTTGCATTACCATAACTTCCCTGTCTACAGCGTTCACAACGCTCACCTTCAGTCCACGCTTGGCAGGAGTCACATTTGCCCACCCTTTCATTACATGTCGAATGATTGTGGCAACCACAATTTATGGAACAATCGGCTCCTGTCCATCCCAATTCACAACGACACACATAATCTGG

>BPA_8796

TAAAGATTCTCTATTCCGAGAATAGTGACAATCAAGAAGATAATTCCTCTGCCGATGGTGAGGTTAATAAGACATTCAATCCCGAGGCTCAATGCTTTCGTCCCATCGATGCTGTTAATACGAGCATGAATAATTTGAGCTTAAGTCCCAAGACATCACCTATGGCTGGTTCATCGCCACTATCGGCCTCCTCAAATTCCC

>BPA_8806

TTTTAGCCTTTTTTAATTTTTTTAACTTTTTACGCTCCTCTTTGGCCTTACGTTTACGTTCCAATTCCGCATCTAACTCTCCCGATTGCTTCCTTTTTTCTGCCTCTAGCTTACGTTTTTCCTGCTCTTCTTTATGGCGCTTTGCATCTAATTCTAAAATTGCACGTACTTTTTCTTCACAAGCTTCTGCTTCCAATTGGGAAACTGTTTTTAAAGCTTTGGCTAATGTTTCT

>BPA_8843

AAGCCGGTTCATCCTCTACAGCTTCTATAACCCAACCGTCAACATCTTCAAGTGCATCAGCAGCTTCAGCTCCTCAACAGGCACCTCAACCAGCGGCAGCTGTTCAAATAGATGTTAATCAAATGGCCAATGTTATAAGTGTCAGTTATTTGGCTGCCAACGGTCTTAATAATCAACGTAATCGTCCTGCGGGTGGTGCTATGCCAGGCGTTAATTTTGGTGTTGGGGGTCCGGGTCAGAATAATATTATGAATATGCGTAATCGTTTGTTTCATGCCATTTATTTTAAAGTG

>BPA_8852

CTCTCCATGCACACCAGAGCCAGTTCATTACGTTCCACGTATGGACAAACTCAAATGCGTTTACGTGGCAATACTTTCAATGGCACCACCAGTTACAATCGTGCTTCCCTGAAAGCTACTGTGGCAGCAGTATCAACTAAACCAGAAAGTCATTTGGCAAACCATACAAATGGCACAACAACAACAACAATTCTATTAAATAATTTTGTTAACTCCCACAATGAACATGA

>BPA_8858

TCCGTGAAAGCAGAACCAATATCTAATACAAAAGAAAGTGATTTTTTAGCTCCGAATAATGCAAATACAAGCAACAATGTTAGTAAACGCCCAATGTCTTTAGATTTAAAATGTACATCGAAAAAACAACGTTTAGTAACTCCATCTCCACTAGTTATTGGATCTCCTGATTCTCAAGTTACAAAACCTCTAACTACACCCGACTTGGAAAAAATACTTCATTTGTTACCTACACCTCAACCTGGCTTAATATATCAAACAAAGGCTGTAGTAACATCTGAACAAGAAGCTTTCGGCAAGGGATTTGAAGAAGCTTTGCATAGTTTACGAAATAATGGCAATAAACAAAATCAAAATGATGGTAGCAATAATATCTTGGTTGGATTGACTAACGCGAGTACAACCAACGTTGCAACAGGAATGAGTGGTGGTTCATTCACTTACGCAAATTTAGAGTCATTTAATCCAATTCCTATTAAAGATGAACCGCAAAACTCGTCGGCTTCTCCACCAGTTAGCCCAA

>BPA_8862

CAAACACTTGAACAGGAGCAAGAATGTTTAACTAATGATTTATCTAGAAAATTGGATCAGCTTCGGCAAGAGAAATGTAAACTAGAACAAACACTTGAACAGGAGCAAGAATGTTTAACTAATGATTTATCTAGAAAATTGGATCAGCTTCGGCAAGAGAAATGTAAACTAGAACAAACACTTGAACAGGAGCAAGAATGTTTAGTAAATAAATTGATGAGAAAAATTGAAAAACTACAAGCTGAAACTGATAACAAGCAAACGAATCTTGAACAGTTGCGTAGAGAAATGGTTGAACTAGAGAATACATTGGAACAGGAGCAGGAGGCATTAGTAAATAAATTATGGAAACGTATGGATAAATTAGAAACTGAAAAACGTTTGTTACAAATAAAACTTGATCAACCGGTATCGGATCCAACAACACCTCGTGATATTACTAATGCTAACGGAGACACTGCCTCCAATTTAAGTTCACACATACAAACATTAAGATCTGAAGTTATACGTCTGAGAGCTAATTTAGCAGCTGCCCAAAAAGAAACCACTAAGAAATTGCAACAATTTGCCCAAGAAGAAAAGTCTATACGTGAAGAGAATGCAAGGCTACAACGCAAATTAAAGCAAGAAGTAGAAAGAAGGGAAGCGTTATGTCGCCATTTGTCCGAATCGGAATCATCATTGGAAATGGATGAGGAACGTTTTTATAATGAGAATATATTGGCAAGTGGTGCGCCTTTGACTGGATCTGCATTAAATGCTCATCGTCAACGTACCATTAGTAGTCCAGTATCACACAGTCCATCAAATAGCCGCCCTTTAAGTCCAGGCACTTCACAACAAAATCGTTGTTATGCTTGTGGACAAGTTGTGAATCGTCGTGCTAGTGAACGTTTTATTAAACCAGCACTGCCTACACCCATGTTGGGACTTAACACTTCAGCTCCGAATGTTTTGTCTGGTTCGACTGGAGCTGGCCCCACAGTTGTAGGCGGCTTATTCTCAAGTTCCTCAAATGCTACGGGGCCATCTGCTAGTTTTCTTAATAGTTTGGGTAATGAACGTTTATCCTTTAATTCAGCAACACTGTTAGGTTATAATGCTGCACCACCAGTTCTACCACCTACAAGTTCTTCCGCTCAGTCCACAAATCCCTTAAGTTTCAATTTACAGAATACGTCCAATTCAAACATGAATGTGAGTGGCACAAATTCCGCTTTTACCCCAACAAATCCAAGTGGTGGAGCCACAAATACTTTTGCCCAACCGGCCAGTCCTATGGACACTTCTTCATGCAAAGACTA

>BPA_8863

AAAACGGATCCACAAAATACCGATTATGAAATTGAAGCTGGTGCAACTAGGAATTTTATGGCTCTGAAAATGGCCGAGGAACAAGCACGTCGGGAGGAGGAAGAGCTACGTGAAGAGGAGGCAAATAATCCAATGAAATTATTAGAAAATCGAACGCAACAATCGAGAAATGAAATTGAAATGTTAGAATCATTGGAAGAATTAAGAGACTTGAATAGACGTCAACAAACTGTTGATTATGATAAAATGTTACAGCAATACAACACAATCGAAACAGAAATAGAACGTCTGCAAAGACAAGAACGGGAACTATAAGTTAATTGTTTAATGAAACTATCCTATATCAATTAAATTTCAAAACCAAACGAATCCAACAGACTCCAGTAAGAAGATAATTGCTGAGGAAATA

>BPA_8873

TTTTAGACGTTGCACCAATTCCTCCAAAAACATTAGACTAGATGGACGGTCACGCATTTTAACGCTACGTGGAAATGTTGGTGGTTGACTGCGATCATGTATACGCGGTGAAAATCCAATGGGGTTAGGAGAATCAGCTCCTTCCTTGGGTTGGGTACCCTTGTCAATAGTTTTCTTTATCTGTGGAGTAAGTTCAAGCGATGTATTTAAATGTTTGTAAATCTCATTTATAACCACTTCGTCAATAATATTAACCATCGCACAGTCCAGACTATAAATGGCTTGATAAAAATGACGCATAAAACGCAGACGATACACGACAGCCATAATATCTTCCGGCTGTGATGACATTTTACTTTGTCTTATCAAATCATCTTCAGCTTCTTTGAGCATGGAGGGAACTAATGAAGCCTGGCAAGAATCTTCCGGAGCCAAAAGTGAAGAATTACCATATAGTTGGAAATCTTCTTCTTCATTAACACTGCTAGAAATTATTATATTTTTAATGTGTATAATCAAATGCCGAATGGCATGGCAGAATGCCTTCAACGATTTATCTTCAATTTTGGCTGGAGAATGCAAATAAAAGCAAGTAAATAACACCTGATCCATAGAATTTCCCTCTAGCCAGGATACGATACAGGTGAATAACGCATCAAAAATACCTATAAGTTCTTCATTGGTAAAACCATTTAGTTTCAAAACTCCTGCCTCCACAGCCGACTCAAATGTATGCGGAATAGCGTCATTTTTATTATATCCCATACCCACATCCATTTTGGGATCCATCATTTCAATGGCTGACATGGCCTCGAATAAACCAAATAGCATATCTTGGGCCAACTCTCCTAAATTTAATTCACTGCAGGCTGATTTAAAATCTTGTGTTATATCAACCCAACCGTATTTCGGATATTCACTGGCGTCCTCATTATTCGATCGCATCATACGTTGCACATCAGTGTCCATATAACCGCAATCTTCCATAGGATATTGTCCACCCTCTGAAGTTTCAGAATCTATGGAATCCATAGTTGCAGTTAATTGCATAGTCCCTGTCGAGGCG

>BPA_8878

CGACAATCTTCAGTTTACATTCAGTTTTGTCGGGCTGTCTATCCAATTGGCACGTGTAATGACCGGTATCTTCCAATAGTGAGTCTTTAATGATTAGTTTTAGATTACCATTGATATCTTTCGAGATAAGATAACGTGGATCATCAGATTCAATTTTCTTGCCATCTTTAAACCAGGTTACATTCGCCATACTGCTACTAACAGAACATTCTAACGTTGTTTCATGTTCTGATGTAACCCTCAAGTTTCTTTTTCAGCGGTTTGGTAAAGGTATAGCTGGGATCAGCTTCTTCTACATTAAGGAACGCAGTACTGGAAACGCCACCAATTTCTATAGTATACTTGCCTGTGTCTTCCACCTTTGGCGTTGTAATAATTAGTTGATATGTGTCATTTTCATTCTTAAATTTGTATTTACTGCCCGGGAAACATTCATCTTTGCGGAATAACCACTTTGGTTTGCAATTTGGTTTGCTGAAACGAGCTTCAAAGACTACCTTCTTATCCTTGCCTTCTTTAGCGAATTGATCAATCAGTGGACTTAAGAACGACTCTTGTTTCTCTTCTCGTCGAAATGGGTGCAAAGTTGGCCCGGGCATTGGTTTTTCAGTCTCTTTAAGTTCACCCCAATTGGGATCTTCTTGATCTTTGTCCCATTTTTGATAACGACGTTTCTTTAACATGGCACGGAAATCCATACCGCTTGAATCCGATACAAACAATTGCATTTCGGCAGAATCCTCGCCATGTACATTCGATACCACAACCTTATATTTTCCCTCATCATTAGGTTTACACTTACGCATACATAATGTAATTGTATTTGTTTGACCATCGGTCAAAA

>BPA_8880

GTAAAATTATGTAATACTTGGTTAAGTCTCTGTTTTTTTTGAAGAATGCGGTTATCATGCAAATAACTTAAATCAATACCATGATCAACATTTATTCTAGGTAAATGAATATTTTTCAAATGAATTGTATCTTCAAGAACCCATTCACTTCCACCTGAACTTTCGCATTCGAAAATGCCAACGCAAAGGTTTATGTACCTTGAATCTGGATCTCCGCATTTGTTAGGTCGTGTAAAACTCTTTCCATATTTGTAAGCACAAGCAACACGTCCTGAATAAGCTGCGCTTATGTTTAATGGTTGCCCAGGAATTTCAATAGCCGAATCTTGCTTCTTACTAAACATTTTCCATTCCGACCATACATATTGATTTTGATCACTAAAATTTGTATTGTTTTTTTCAATAGGCGTGGGTTCACACTTCCAAAATCTCGAAACGGAATCAGAACATGCTGTTACTATAATATATGGAGCATAGCATGCTGGATATATGGAGGACGAACTGAGATGTCCAGCAGCTGGTGATGCATGTATTACGTCAACACCGTCAGGTAAAGGTAAATCTTGGGTGCAAACTTTTTCTGTTGTAATAACAATATGAGGAGGTTCAATTTTTTCATCCTTTTGATTGGCATCTGTTGCATCAATATTTATCTCAGACATTCTACGATGTGCATCTATATCTTCGAATTCTTGAGTAATATTGCTGTC

>BPA_8894

CAAGAATTCATTAGTGTTGGTGAAGATTTGTAAAAGAACAAAAGAATAATGTCGTCGCTATCAGCATCGGCTGAAAATGTTTCTACAGGTACGGGAGGTGGTAGCAGTTCAGCTGCCGGAGGTAGTAGTGGTGGTGATGGAAATAATCAAGATGGTGCTGGCTCCAGTATGTGCTTGGAATTGGCTTTAGAAGGTGAACGTTTGTGCAAGGCAGGTGATTGTCGGGCAGGTGTGGCATTTTTTCTGGCTGCCATAAGAGCAACAACGGATGACCTTCGCACCTTGAGTGCAATTTATTCACAGTTGGGCAACGCCTATTTTTATTTAGGCGACTACTCGAAGGCCAAAGTATCACAAATTGGATTTGACTTTGGCTCGTTCCATGAACGATAAACTTGGAGAGGCAAAATCATCGGGCAATCTGGGAAATACCTTTAAGGTAATGGGTCGTTTTGATGAGGCTGTTATGTGTTGTGATCGTCATTTGACATTAGCCCGACAATTGGGCGATAAATTGTCCGAAGGACGTGCTCTATACAATTTAGGCAATGTGTATCATGCAAAGGGTAAACATATGGGTCAACGCAATCCCGGTGATTTTGGTGAAGATGTCAAAGAAGCCTTAACCAAAGCTGTGGAATATTATCAGGAAAATTTGAAATTAATGCGTGAATTAGGAGATCGTGGAGCGCAAGGAAGAGCTTGTGGCAATCTGGGAAACACCTATTACCTGTTAGGTGATTTTCAAGCTGCAATAGAACATCATCAGGAACGTCTACGTATTGCACGAGAGTTTGGCGACAAAGCTGCAGAACGTAGAGCCAATAGTAATTTGGGAAATTCTCATATATTCTTGGGGCAATTCGAAGAAGCTGCAGATCATTACAAACGCACTTTAGCCTTGGCCATTGAGTTGGGTGAAAGAGAAGTCGAGGCTCAA

>BPA_8898

GGGTTTATGGGCCTCCTTTAGGAAGGAAGTGTCTAGTATTTTGTGATGATATTGCCATGCCCAGCAAAGATACTTATGGTTCTCAGCGTTTCTCAGCCTCCTTTGGAACTGGTGCGACAATGGTTAGATCATGGCTATTGGTCGGATTTAACAGATACCACCAAAGTTGAATTAATTGATATGTGTTTCTTAGGTGCCATGGGTTTGCCAGGCGGTAGTAATTATATTTTTCCACGTCTTTACCGTCATACATTCGTTGTGGGTGTTGATTCATTTGAAGATGCTACAATAATCAAAATATTTACAGCAATTGGTGATTGGCATTTTGCCAAAGGTTATCCCGATAAAGTTGCATTACTTTCACGGGGTCTAGCTGAATCTATGGTCAGTGTTTATCGTTCTGCAATGAGAATATTTTTACCAACTCCTGCCAAATCCCATTATACGTTTTCGTTACGTGATATAACCCGTGTTTTTCAGGGTGTTGTTTTGGTGCCAGCAAAACGTTTACACGATCCAGAAAAGCTTGGACGTTTGTGGGCTCATGAAACGTATCGTGTTTTTTATGACCGCC

>BPA_8902

GGCTTGTGAGAGACCTCTATTCTCGCGATGACATGCCCGCAAAGCACTAGCAGCCCAAGCTGTTCTCAAATGTTCTGGTAGATAATCTTCCAAACGAAAGTATTCATTACAGGAGCTGGCAGTTGTGGAAACTGAAGCATTTGTGGAAGCATTACTGGGTCCCCGAGATATCGATGATGACGAGGAAGAGGGAGAGAGGGGAGTTTTAGCAGCTGCACCCAGAACACGTGTTTCAAACCATCCATACCCATAACATTTTCACGCGTTGAGGCCATATAGCTAGATAAACGTAAAACTCTTTCATTTACCATATTAGCTCGTTTACTAATCTTTGGTAATGTTGTTGTCGCTACTCCTGTTGTTGATGATGCCGATGATGTTGATGATAATGTTTTACCAACCTTTTGCTCGCCCGTTGCACTCTCCAATTTACTCTCACCAAGATCTGAGGCCAACTTTGACTCACCCATTTGTAAGCCATTGCTGCCAGTACTACTGCTACTATTACTATTATTTATGCCACTACAGGGTGCATCACCTAAATCTGCTTGCAATGCTAGACCAG

>BPA_8906

CTCAATATGAGATTATTTGTTTTATTGGCTGTAGTGGCCTTTGTTGGCCTGTCTCTGGGAGATGATAAAAAGGAAAAGGATAAGGATATTGGTACAGTCATTGGTATTGATTTGGGTACCACATACTCATGTGTTGGTGTTTACAAAAATGGCCGTGTAGAAATTATTGCCAATGATCAAGGTAATCGTATCACACCCTCTTATGTTGCCTTTACCGCCGATGGTGAACGTTTGATTGGTGATGCTGCCAAAAATCAATTGACTACCAATCCTGAAAATACTGTTTTCGATGCCAAACGTTTGATTGGCCGTGAATGGAGTGATAGCAATGTTCAACATGACATTAAATTCTTCCCCTTCAAGGTTGTTGAAAAGAATTCCAAACCCCATATCTCTGTTGAAACTTCTCAAGGCAAAAAGGTCTTTGCTCCTGAAGAAATTTCTGCTATGGTTTTGGGTAAAATGAAGGAAACCGCTGAAGCCTACTTGGGCAAGAAGGTTACTCATGCTGTTGTCACTGTACCCGCCTACTTTAATGATGCTCAACGTCAAGCTACCAAGGATGCTGGCGTCATTGCTGGTTTGAATGTAATGCGTATCATTAACGAACCCACTGCCGCTGCTATTGCTTACGGTTTGGACAAGAAGGAAGGTGAAAAGAACGTGTTGGTCTTCGATTTGGGTGGTGGTACCTTTGATGTATCATTGTTGACCATTGATAATGGTGTGTTTGAAGTAGTTGCTACCAATGGTGATACTCACTTGGGTGGTGAAGATTTCGATCAACGTGTTATGGATCATTTCATCAAATTGTACAAGAAGAAGAAGGGCAAGGATATCCGCAAAGACAACCGTGCTGTACAAAAATTGCGTCGTGAAGTTGAAAAGGCTAAGCGTGCTTTGTCAAGTGCCCATCAAGTTCGTATTGAAATTGAATCCTTCTTCGAAGGTGATGACTTCTCTGAAACTTTGACTCGTGCCAAATTCGAAGAATTGAACATGGATTTGTTCCGTTCCACATTGAAACCCGTACAAAAAGTAATGGAAGATGCCGACATGAACAAAAAGGATGTTCACGAAATTGTTTTGGTTGGTGGCTCCACCCGTATTCCCAAGGTCCAACAATTGGTAAAAGATTTCTTTGGTGGAAAGGAACCTTCCCGTGGTATTAACCCCGATGAAGCTGTTGCTTATGGTGCTGCCGTCCAAGCTGGTGTTCTCTCCGGTGAACAAGATACCGATGCTATTGTCTTACTCGATGTCAACCCATTGACTATGGGTATCGAAACTGTCGGTGGTGTCATGACCAAATTGATCCCACGTAACACTGTCATTCCCACAAAGAAATCTCAAATTTTCTCCACTGCTTCTGACAACCAACACACTGTCACTATTCAAGTCTTTGAAGGTGAACGTCCCATGACCAAAGACAACCATTTGTTGGGTAAATTCGATCTTACTGGCATTCCCCCAGCACCTCGTGGTATTCCTCAAATCGAAGTCAGTTTCGAAATTGATGCCAACGGTATTCTCCAAGTAAGTGCTGAAGATAAGGGTACCGGTAACAAAGAAAAGATTGTTATCACCAACGACCAAAACCGTCTCACTCCTGAAGATATCGAACGTATGATTCAAGATGCTGAGAAATTCGCCGATGAAGACAAGAAACTCAAAGAAAAGGTTGAGTCTCGCAACGAATTGGAATCTTACGCCTACAGTCTCAAGAACCAAATCGGTGATAAGGAAAAACTTGGTGCTAAATTGAGTGAAGACGAGAAAGTAAAAATGGAATCTGCCATCGATGAATCAATTAAATGGTTGGAACAAAACGCTGATGCCGATCCCGAAGAATACAAGAAACAAAAGAAGGATTTGGAAGCAATTGTACAGCCAATTATTGCAAAATTGTATCAAGGCGCTGGTGGTGCTCCTCCGACAGACGGTGATGACAATGAAGACGAGAAAGTAAAAATGGAATCTGCCATCGATGAATCAATTAAATGGTTGGAACAAAACGCTGATGCCGATCCCGAAGAATACAAGAAACAAAAGAAGGATTTGGAAGCAATTGTACAGCCAATTATTGCAAAATTGTATCAAGGCGCTGGTGGTGCTCCTCCGACAGACGGTGATGACAATGAAGACGAGAAAGTAAAAATGGAATCTGC

>BPA_8922

TGATTGTACATAATTTGAATTTAAAGAAGGCAATTTAGAGCTCTCCGCATTTTTATTATTGTTCAGGGAACATGAGCTTCCAGCAGTAACTCCACTTGCGCTAGTACCAGCAGATGAAATTAGAAAATCATGTAGACGCTTCTCATATTCTTGGTGTTTACCTTGATGCATTTCCTCTTTTGTAAAACTAGCTTCAGGATCACCTAATTCATGTAAATACATACAATCTGCTTTGGGACACTGCTGGTTTTTCATGAAGTGGCTGCAGTATTTAGTTGTGCCGAGACTAGTCTTTATAAGGCGACCGTCAATCATAATATTATTCACACTCTGTATGGCTCTCAATGCATCGGTATTGTGAACGTACGTTACATAAGCTGAAGCGGAAGGACCCTGAACTCCGGCGTATGCTGTACTAGGATTAATCACGACTTTATGAATTTTACCATATTTCCCAAAATATTCGTGCTTTTTTAAAATATCTGCATCAGCGAGTCGTGGTGGTAAGCCGACCACAAAAACTAAATTTTTTTGAACAACACGAACATTTGCCAAACGTTTACGGTTTTCTGTAATTTTCTGCTTTCGTTGCTGATCTCGTTGACGTTTCTGAGACTTAAAAGCAATCATCTCCTCCTGAGTCAAAGGCTTGAAATCTGCAGGATTTTCGGGATACTCTTTTCGACATGCTGGACATAACTCATTTTCGTCGGTCCGAATTCTATGCCAACAGAACCTGCAAATCTGATATCCACAAGTACAAGGGAAAAAATTAAGATCATCTACTTCCAGAGGTTCCATGCACAAGGGGCATTCCACAACATCATCGGTACTATTGTTTA

>BPA_8927

AAAAATCAACCAAAGCAGCTTCAGTACCATCACCAGCTGGTTCATCAAATTCACAAGATTCATTCCCAGCCCCTGGTGGTGCTGCTAATACATCTATGGATGGCTATCCGCAATATCCTGGTAGTTATCCCGGTGGTCCGGGTCCTCAGCCAGATTATGGTGCAGCTGGTCAAATGCCCAGACCACCATCACAAAGTAATGCGCACGCAAAGTCCACATCCAGGTTCTCCATATCCTGGACAACCTGGAGCTTATGGCCAATATGGTTCAGGTGATCAATACAATGCAACAGGGCCTCCTGGCCAATTTGGGCAACAGCAAGGCCAATTTCCACCCCAAAATCGTAACATGTACCCTCCCTATGGACCCGAGGGGGAAGCGCCTCCGTCGGGCTCAAATCAATATGGTCCTTATGGAAATCGTCCATATAGCCAGCCACCACCTGGTGGTCCTCAGACTCCTACTCCTCCTGGTGCTCCTACTGCTGCGGGTGGTCCACCAACAAGTGGTCCTCCAGCCCCTGGTAGCTCACCATATCCCCCTCCTGGTGCTCCTGGTCAACAAGAATATTATAGGCCACCAGATCAGGGACCTCAGCCACGTCGTCATCCAGATTTTGTTAAAGACTCTCAGCCCTACCCTGGCTATAATGCACGACCACAGCTTTACGGTGGGTGGCAAGGTGGTGCGAATCAATATCGTAATCAATATCCTTCATCACCCTCTCCTCAGGCTTGGGGTGGTGCTCCGCCACGAACATCAGCTCCACCATCAGGACCTCTGGGTCCTCCTGGTCAACAACAACCTCCCCCGCAGCTGGTGCTTCACAATGGGATCAACACCGTTATCCGCCACAACAGCAACAGCAGCCGCCACCTCCGCAACAACAACAGCAGCAGCAACAACAGCAGCAACAGCCCCAGCAACAACAACAGCAGCAGCAACAACAGCAGCAACAGCCCCAACCACCCTATCAGCAGCAGCAACAACAGCAACAGGGACCCCAACAGCCGACGCCACCCCAATGGGCTCAAATGGGTGGAGCTGGCACTGGCGCACCTGGAG

>BPA_8941

CGGCTGTTGAGCATTAATTGTCGTTGAGGGCTGTTGTTGTACTGACGCCTGTGCTGCAGCGGCAGCGGCAGCAGTTTGTAAGTCTGACAGCGACGTTGCTACGGAGGCTTGATTTATATTTGGCTGTGAACCACAATCGTATGCCGAGTCCACACTATTGGCCCGAGAATGTGAGACTGTCGGTGATTTGGGACTGGCAGCTGGTGGCGTAAAAAATG

>BPA_8952

AGATGGTAAAACCTGTACACATCCGGTCATGCGACGGCCGAGTCTTATAAAGGCAAGTAATGAAAAACGTTTACCAGCCACAACTGCTGTAGAACCTTATAATGCCAATAATACGCCAAATGTTGTAGTTGGACCCAAACTTTATTCAAGATGGGCTGATAAAAGAGCTATACGTGATTCCTCAAATAGTATGGATACTAATAGCTATCAGCATCATCACAGTCATGGACACCATGGTCAAACTCAACAGCTAACACAAGAGTCAGCTATTAAATTTCAAATTATACCGCCACACAACGAACAGCAACAACAGCAGCAGCAACAACA

>BPA_8961

GCAGCAGCAGCTGATGTGGATGGTCCTGTGCTCGTAGAGGGAACAACATCTGATTCATTATCATTTTCCGATTCAGATGAATGATATACATCAACTAATTGATGATAAACTGCAGCCACATCGATATCATTCGGCTCTCTTATAATACGCAGACAAAGATCCGTTATGATACGTGCATGTTTTACAATATTGGTGTGTCTTCTATTA

>BPA_8963

GGAAAACGCACAAAGCTCTGAGGAACACAAAAAGTTCTACGATGATTCCGAGCAAAGTGATTCCAATAGTAGTGATGATGAAGGCAATAAAAAATCCAAGCACCGGCAAAATAATAAAAAGCAATCATCTAATTCCTCACCCTCTTCAAGTTATCATATTGATCTTGATCACCGTTTGTTGTTAAGGCAAACGAAACCTTTGTTGCAA

>BPA_8966

AGGCATGCGGATCTGAGGCATAAGGGAAGAAATCATCATCTTTTGTGGGCCAAGTAATGCCAGCATCATGAAGAGATTTGAGATAACAGGAGGGTGTCGAGTACAGAAGATTTATATCGGAACCATTAGCTTGACGGGCATTGGAGTATCGTATTAATTTATCCAAATTTTTATAATAAACATTTGCATCCTGATAGGTAAAATCACCACCCATTGTTACTATTATGTTGTTAGTGCGATAATATTTTGCTTGTTTTTTAACAAAATCATAAAAC

>BPA_8968

GGAGAAACCAAATGCTCTGTTTGTAGAGAAACACAATGAACACCGTCATATAGCTATAAAAGCTCCAGATGAAATTAATTTAAACTCAAACGATTCCTTCGTTCAGGAATTTATACAGAATCCATTTCTGGTAGATGGTCATAAATTTGATATTGGAGTTTATGTAGTAATTTCATCCGTTGATCCATTGCGTCTATATATTTATAGCGGCG

>BPA_8978

ACAAATTTAGTAACGTCCTTTGCGATTATCGCCACGATCGCGACTGCGAGAACGACGACGATTCTCACGTGAGCGGGAACGCCTGCGTCTAGGTGAACGAGAGCGAGAACGTGAACGTCGTCTACGGGAATACAGATATCTTCTTAACTCTCGGGAAATCGGTTTCAAGTGCATGAAATTACAAAAGCCCGAACGTGTACATTCTCCCATTTCATATTGACGGCAACAGGCTTCACGAAAATCAGTTACTGGTGATAATTCTGAGTAAACCGGACGTCCACCAAACCATCTATTGTTTAAATCATTTGCGGCCTTTTCAGCATCAGCCTCATGGCGAAATTTTATATAAACATTACCCACCAAATGATCTCCCAAATTATCACACACGTTCATTTCTTCAATTTCTCCGTACTTATCC

>BPA_8984

GTGAAATTCACTTGCATATTGGACAAAGGATCGCCAGTTGAGCACCATCCCATTAAGGGATTTTCCCAACGTTCGCGATTATCGAAATCTATTTGCCAGGTGTGCAAATTATTGGTACCACTTTGCATGGCATTTTTAGGTGGCATAAAAATGCGTACTCGTCTGCCCTTAACATGTTCCTCTGGCACACCAGTGATTGGACTAATATCAACTTTTACGGGTACA

>BPA_8988

TTTGATTATTGACATATTGAACTCTTCAATGAGGGATTTAAAAACGGTGGCTGCTGAGACCTTGGCCAATGTGGCTAAAGTTAGACTAGCTCGGAAATATGTACGGACTTGTGGTGGTATTCCGAAATTAGTTGATTTACTAGATGTGAAATTACAAATTCTGCAAACTCCTAGAGAGGAACTTACGGCAGAAGAGATAGAACAATTAAATATGGC

>BPA_8997

AAAAGAAAAGGATAAAGCTACTTCTAAGTCTCAGTCTAGAGACAAAGATTTCTCTGCCAAACAATTTATAGAGAGTGTTAAGAGAGGTTTAGGTCCTCATTCCCCCTCTAAACTTGTAACCGAATCGGCAGCTATTGCCTGTGTGAAATTATGCAAGGCAGCTACATATGTGAATATCAACGACTCTAATAATGTGATTTTTAAA

>BPA_9001

TGTGACCGCCACATAAGCAACACACTGAGCAGCACAAGAGGGACGAGTATTTTCAGTACCCAAAGCTCCCAGAATGTTTTTCTTAATATATTCCCTTGTCTCTTCGGGAAATTGATGCCATCTTTGTTGGTATTGCAAATTAATGGTCTCATCTTTACTGGTCAAATGATTTTTCAATTGCAGACCAGCTGCCATACGTG

>BPA_9007

AGTCATCTCTAATATGTCTTTTTCTGATGATTATTTTTGGTTAATTAACAAGCTCCCACTTTGCGTATATTCAAAGTACGTCCCAATTTTTTGTATTCCCTTTGAGTGCACTCGAATTCACAGCGATTTTTATAGGTGACACCATTTGAACCACAAATCATTTCTTTATTACTCAAATCGCAAGGACAAAATGCAGCCTCATTAATGCTCACAAATGAGA

>BPA_9013

CACACGCACAAATTCCTGACCACGATAAGAACACGTTAGCAGCACTATAGTAACACCCACAGCATCTGGTTCAGGTATTTTTGTTACATCTGGTGGATCAGCTTGAAATACAAATATATGTCTACCCTCTGGCACAGGACCCACATAAATGGTATCTAATACTTGATCATATTCTTCTGATTCTGCTGAGCCCACATAAA

>BPA_9014

AATTATCGTCATCATCGGGCCAATGATTTTCTAGTGCCCTTAGGACCTCGCGGTTGGACTATACCACCAAATCGTAGTTATGGCATGGTGGTGGTACGTGTATTACGCGGAGCTCTTAAACTACGTTATTTATTATTAGGTGGTGCTATTGGTGGTGGCATGACTTTAAATAAAAAATATGAAGACTGGAAAGATGGTTTACCCGATCTCAAATGGCTGGAAGAAGCTTTGCCTCAGGGTGAGAAATGGAGTCAATTTTCAAAAACTTTAATTGAAAT

>BPA_9017

CAAAAGTTGACGATGTTAAACCAAGTGGCAATGAGTTTGATGACTTCAGTTGTGTTTTCAATGGTTTTTTATTCGAATTTTGATTTTGTTTAGATTGAGAGCTTTCAACAGCGGAAGTATGATGTTGATGCCGTTCCTTTTTGGATTTTTTGGGATTTGCATTATTATCGGGATTAATTGGATGTACTGTTGCTGCTGCTGCTGTTACTATTGTCACATTATTTGTCGATGAAAGAGGTGGAGTTTTTTCTTTGGATTTTTTATTTAAGTTGTTAGTAGAACTGCCTAA

>BPA_9018

GCAGCTGAAAAAGCTAAAGTTAAAAGTGAAGAAATTTGGAATATCGCTTGGAAAATATGGTTAAATATTGGTATGGAGAGTACAAAAATGTCTGCAAAATCTCAAGATTCAACACAAGATGAATTCTATATACCGAGCCAGGCATACTTAACAGCATTAATGCAAATATTTCCAGCCATATTTCAGCAAATACAAAAAAGATTTAAACCAACTGATTTTGATAAATTTTGTACAGT

>BPA_9044

TTCCCATACAATCAACATGTTGCCAAGCACAACATTTATCACAACATATCATGTAACCATCATCGTGTGTTAGATCACATATACAACGCGTAATTGAATCATCTTCCAATTCTGCTTCAGGAGCAGTTTCAGTTTCCTCCCCAGGATCTATATCGCCAACAGCAAGTCTGGAACTATTACTTATAGCTGAATATGAATCATCATCTTGCTGAACGC

>BPA_9066

GTTTTCTCATCATCGTTCGAGTCCTACAGTTTAGGGGCTTTTTAGTTGGTATTTGCGGCACTTGAAGATGATATTGATCTTCACAATATTGACGTAACAGATGAATTCCTTCAATTCGATTATTACCATAACGTGATTCCCATGCATCCAGTAAGCGATTGTAATACCGTGGAGCTTCAGTATATTTTAAATATTTGTAAGTATCTATTGTAGGGAATATACGTTCCAATGGAGTACACCGTGCCAATTCGTCTTCTGCAATGATGAGGCAACGTACATCGTCAGGAGTCAGATGTTCCAAAATAGGATTAATATACTCTTCGCGATCGTCCATACTCTTTCGAGTAAACATATTGTGTTTTATTTTTTC

>BPA_9081

GCTAATTCGGGGTCAACCATAAAACGAACTTCCATGCGAGAATCAGGAGTAACTTGGTTATCATCTAAAAATTTAACATAAACACGTCCCAATGTATTTTCCGGCAACTGACGTAATTTTTCAATATCAACTGTACGTGTATTAATGCGGGGTTTATGTTGCAATATACGTTGACCTTCATCGCAATTTTTCATGCTATACAAAATGTTTTG

>BPA_9109

TTTGGTGCTTATGTCTCACTTGCAGGATACTATCGATGTGGCCGATCCTTCCACTAAGATCCTTTACAACCGTATGATGGCCAATTTGGGTTTGTGTGCATTCCGTCAAGAGAATATCAAAGACGCCCACCATTGTTTGGTCGATTTAATGGTTACTGGCAAACCCAAAGAACTTTTAGCGCAGGGTTTGTTGTCTCAACGTCAACACGAACGTTCTGCCGAGCAAGAAAAGATTGAAAAGC

>BPA_9110

CTGCAACCATGCGCCCTTTAAGTGAATCTACAAATTACAAATCGCCACCGTCTCCACCCCCTAATAATTCTTTGACTTGTCAAATAACACATCCTCAAGCTTTGCAGCATATTAATTTAGTGGAAAATGATGATGTGCCACTGGACATGTCTGTTGGGAATTTAAAGCAACGTAACTCGCCGCCACCACCATATCGGGAACCTTTACCTGGTTCACAATTTTCTTCCACATTAGCAC

>BPA_9117

ACGCATCCAATTTCAAATAGACCTGTTGGAAATATGCCACCAATTAGTGGAGCTTATGTCAGGACAGATGGTCCTAGATTTTATGAACTACCAACTGGAAGTCGCATACCACAATCTCCAGTTCAGCGACATCGAGGACCACCTCAGTTACCACCCAGTTTATTACCACCACATCCTGCCAATCGTCCAACGTATGGTCCACCACCACCTCTCAAAGGTTCAAGTACAGGTTCCCCTCTATCCTCAGCTTCATCCGCAAATACACCCGGATCTATACGAAATCCTTTAC

>BPA_9151

AATAACAACAACTAGCACAGGCGGTGAAAATACAACACTGCCATGCACAGCCAAAACAGAAACACCCGTAACCTCTCCGTCACCTATGACAACCATAGGATCCGATATAATATTACCGCCCATACCAACAGTTTCAGCACGTGTTAGTTTAACAGCGGGTCCAGCTACACCACATATAGGTATACATCACAACAACAATATAAATAATAATAA

>BPA_9160

TCAATTTGAGGATCCGCTTGACTGCATGGAACATTGTTTGGCCGAGAATACCGGTCATGTACAACGTACAGCCCAACAAAATGATTCAGAGTATAGTTGCTTGTGGCGTAATTGCATACGTGTACGTAAAAATATGCAAGCATTTCCCTCACTGGTGCGTCTCATCAAACACGTACGTGAGGTACATTTATCGAAACCGGG

>BPA_9178

ATTCGGTTCAGCCCATATTACAGAAGAAATTCATGCCACCTCAAAGGATGAAACCACTTTAGAAGGTTATCAGAAACATAAAAAGGCATTTGCCGCCCCAGCTCCACTCACAACACGCGAAGAGGAAATGGCCGAGCTAAGAAAAACTGAAGTTAATACCGAAATTAATACGGATACTCGCCATCAAACATTGGGTGGCATATCATGCCCTC

>BPA_9182

GTTAGAGCCAATTGTAGAGACATGCGATCATGATGTCCCTGGGGTGTATACTTGAGTAAATCTTGTAAGAAGAGTATAAATTGTGGAAAACGCTGTACTGGCTTAACCATTAAACCAAAGAAACTTAAACGATCATGAGCACTAATTTGTTTAACTTTGAAGAAATCCGCCAATGCAGATTTACGTTTCTCTTCCATTTTCGCTAATTCCATG

>BPA_9190

CTTGGGTTTCGGTAGCGGAGTTTTCAGGTTCTTTTGTGACATTTGTTTCGTTGGGTTTACTTGATAATTCTTCTGCTGCTTCTTGACTATCAGTGGCCTCTACAACAGGTTCTAAAACAGCATCTGTTTTATCTTCTTCTGGTTTTTCAGTTATTTTTTCCTCTGTCGATTCTTTAGGCTCATTAGTTTCAGTTACCTCCTTTGTTTTTTCTTCAGATTTTGTTTCTTCTATTACCTTTGCTGGTTCTTCAACATTT

>BPA_9194

GAGGAATATGAGTATAAAATTGCTCGTGAATATAATTGGAATGTTAAGAGTAAAGCGTCTAAGGGCTACGAAGAAAATTACTTCTTTGTTATGCGTCATGATGGTGTCTACTACAATGAACTGGAAACACGTGTACGACTTAATAAGAGACGTATGAAGGTGGGCCAACAGCCTAGCAATACAAAATTGGTGGTTAAACATAGACCTTTGGACG

>BPA_9199

CTGCTACCAATGACGATGGAACGCCTGTTGTTCGAAAGAAAGCCAAAGTCAAAATTGGCAACAAGTTTAAAAAGAAGAAGAAGCTTAAAACCACCTCCCGCTTTCCCGATGGTGAAGATGGTGAACATGAACATCAGGACTATTGTGAAGTGTGCCAGCAAGGCGGTGAAATTATACTTTGTGACACTTGTCCACGAGCTTATCACTTGGTATGTTTGGAACCCGAACTCGAAGAACCTCCAGAGGGCAAATGGTCATGTCCACATTGTGAAGCTGATGGTGGGGCCGCTGAAGAGGAAGATGACGATGAACATCAAGAATTTTGTAGGGTATGCAAAGATGGTGGAGAATTATTATGCTGCGACTCATGCCCTTCGGCATACCATACATTCTGTTTGAATCCACCTCTTGACACCATACCAGATGGTGATTGGAGATGTCCACGCTGCAGTTGTCCACCATTAACTGGCAAAGCAGAGAAA

>BPA_9214

ACACTTAGGGGTTGGTGCAAAAATGAACAGAAATTACGCTTTATGTGCCGTCAATTAGGAACAGCAGATCAATTAGCGCCAATGTCAGTCATAGAAAATCCACCAGAAAAGCGTCCTCGTTTCGAAAATCATATCCAGACTCCAAAATATCCAGCTTCCTCTGGTTTCGATGAATTTGGCTACAATCGCTTACCGCTTAATGGT

>BPA_9218

CAAATGTTTAGCTTGAGGTTTACGATCATCATTGTGTAGAATTTTCTCCGTTAATTTGAGACTAGGATCCATTTTCATCTGTTCCCAAGAACCTATACCATACTGATAAATGCCACACAATAGTTTAGAGTCTTCTTCATCGCCCCATTCTACATCGAAATTGGCAGGTCGTGTTTTAATGTTCAATGACCAATTAAGACGTTCAGCTGCTGTGCCGGGTATGACATCA

>BPA_9224

CGCTATTAAATTATTTTTGGTTCTTGTTGCCTTCATCTGCTGTATGCAGTTGGTAGAAATGGCCCGTTTACGCCGTGATGCTCCTGCTGGCAACCCCATTGATGAATTGTCCGCTCTTTTCCAAAAAGCAACCAAGGACTTTACTCAAACTGTTGAAGAAAGCAATGTCTTAAAGGAAATTAAAGAGAAATTTAGCAACATCAACTCCGATGATGTCAAGAAAGCTGTTGAAGAAGCCGGAAAAAGCACTAAGGAAAATTTGGACAAATTTCAAGATTTCTTCAAGAACATCGTTGATGCTGTTGCTAAAGAAATGAAA

>BPA_9233

CTACTAGATGGTTCGATTGGTCTTTAGCCACCTCAATACCATTGATCATGGTTCCACTCTTTTACTTTATAGTTCCTGAAAGTGTCCAATGGCTGATATCGAAACAAAAATATGAGAAAGCAGTACAAAGTTTACAACGTGTAGCGAAAATAAATGGCAAAGCTGTCGACGAGTCGGTGTATAGAGAATTTATAGAGGAATGTAAACGTAATCAAATGCATATAAAAGTTAAGCCCAATTTATGGGGTCTA

>BPA_9251

AGAAAGGGCAAAAAGCTTTTACAGAAATTGAAGCTTCCACTCTAACCTTGGCGGAATGTGTTAATAATATTGTGAATTTTACCGCTATACAAGAGGAAATTAATGAAGCTTCACCCGAAGGCGAGTTAGATGTAGTGTTTAACAAGTACTGTAACAAAAAGGAAGATGCTTTCAAATGTGTTGAGACCTTTAATTCCAAACTTACACCCTGTCTAGATGCCGAAG

>BPA_9280

AGCAAATGTAATTGTTGAGGTCAATACGGAACCCTCTTTGGGTGGTGAATAGTAAACACAATACAAACCATTACCCTTCTCCTCAACACGTAAATCTTCGACGGGAGTTCCTTCAGAATTTTGTATTTTAACCGCAATACGACCGGGACCAGCATCTTGTACATCAATAAAGAAGTGAGTGGGTACACCCTCTCTCACCTCACCG

>BPA_9287

CGCACTATGCTGACGGTTTTATCTGAGCGCACATTGGAGATTTTACTCAATTGTGAGAGTTGCGTTTCTTTGTAACATAATAACATCATATCAAGTTCTTCACTGAGCGTATTTAATTCCTCCTTACTTTCACGTATATGTTTTTGTACCCGATCATATTCCAATTTCCATTCCATAGTATCACTTTCGTCGGCTCTTTGACTTATG

>BPA_9300

CCTCGATTCGCAGAATCGGACGTATTTTTCGTTGAGCCAGATTGTACACCACCGCTACCGCTTTCATTTTGTTGCTGTTCCAATTGTGCTTGGGCTTTTTTCTTTTTGCTTGTACCAAAACAGCCAAGATTTTCACCTTTTGTAGAGGATAAAGCTCTTGTGGGCACAGATATCGGTTGCTTATCATAGTTAGGATCCTCTACTTCCTGACAAC

>BPA_9302

TTTAGAGATATATTTAATTTCGACAACACAACACTGCAGCAGATTGATTTTGTTCCAACACTCGCCACCATTTTGGGTATACCAATTCCATATTCCAATTTGGGGTTAATTAATTTTAATCTCATTCCTGATATTCCGATTCCACATTTAACAAAACTTCAAACACTCATTTTGCATGCCTGGCAAAATGCACAGCAGATTTATAAATATTTCT

>BPA_9312

CAGAGGCGTCACATCTAAAAACGATTTAGATTCTTGTTGGAGATCTGTTAAATTTTAATTCGAACCGTTAAAAAAAAATCACAAATAAATCCAAAAATCACAAATTTTATTTCGGACTAAATTTTCGTCTTCGAACTATTAATTCAGCCATTTCATCTTTAACCAAAAAACAAACAAACATTTTTTCTGTTCAACGTTGTCGTCCAACAGGCG

>BPA_9314

ATTATTAATAACTTCACGGTATTTTTTGGCGTTAGTGCGGAACATTTCGGTAAGTTTATCTGAAGAAATACGTGTCCATTTCTCTTTGAATTGTGAGCGCAATTGATTATCAGAATCACGTTCTTCGTCTAACATGCGTTCAGTTTCATCCAAAATTTCACGATTACGATTCAATAACTCAGGCAATTCCTTCAAATGATTTTGAATAGCTTCAAT

>BPA_9318

AAAATCGAATACGTATCCTCAGCCTGAAGGTCTTTTAGCTGATTGTATGTTGACTTATGGCAAAAAGTTGGGTGAAGACAATAGTGTTTTTGCGCAGGCGCTAGTTGAGTTTGGAGAAGCGCTGCGTCAAATGGCTGATGTTAAGTACTCACTGGATGACAATATTAAGCAAAACTTCTTGGAACCACTGCACCATCTGCAGACGAAAGACCTCAAAGAGGTTATGCATCATCGTAAG

>BPA_9324

CTTTTTCCACTGTAAACTTTGGCGTTTTACCAATCACCCAATCCCATGAGCTAAATTCTTGGCGTAATTCTTCAATACCGGGGAACCATTTCTCTGTAGGGTTGACTAGCTGAAATCCACGTTGTTTCATTGTCTGAGTGCTCCCACCGTCTTCCAATTCTGTCGCAGATGTGCGCAAAAATTCATAGCCTACCGCAGAC

>BPA_9339

CGTGCCTCCGCTGAACTCTCCAAGCGTATTAACGGCCTCGGCTTGCGTTCGAAACATCATCATGGCAATAATAGCAGCAGCAGCAACAACAGCAGTAACAGTAACAGCAGCAACTCCAATAGCAATAATTCGGCGGGGACGGCAGGTGGTGGTGGTGGAGGCGGCGGCTCTTCTTCAACCTCTTCGAATAGTTCCAGCTCAACGAACTCAACATCCTCGTCCTCAACAACGTCCTCATCATCGTCCTCATCCTCTTCG

>BPA_9357

GAGAAATTTTGTCCATAGAAATATATATTCTACAATTTTGTGGACACTAGGCAGTGTCAAGGAACTAGCTATATACTGGAAATGGAAGAGTACTGCTGTTACCCTATAATAAATTAATCAATCGAAAATTTTGGAAACACGTTTCCGAAAAATCCGGGCTGTTCCATTTTGCCCTCGGAAAATAAAGATTTTGGCGCGTTAAGTATTTACCAATTGAAAAATCAACGTCAGGGATGTATAAGTTTGATTTTTGTCAATATTAATTGTGCACAAATGTTATTAACG

>BPA_9387

TTACGAGAATTGGGAGCAGCACGTTCACTACTTAGGCAAACAGATCCAATGATAATGTTAAAGCAACATGAACCGGAACGATATATACATTTGGAAAATATGTTACAAAGAGCATATTTCGATCCGCGCGAGGCATATCCTGAAGGAAGCAGCAAAGAAAAAAGACGCTCCATCATTGCCCAAGAGTTAAGTGGTGAAGTGCATGTAGTA

>BPA_9396

TCAATTTCTTTTGACAATTCTTTTTCTGTATCGATGGATTGTTCTTCGGTCGATGTAACTACTTCTGTGTCGTATACAATTTCTTCTTCGGCAGTAGTTCCTTCCATTGTGAAAGCAATAGAAGCAGATTCCTCAGAATCATTTCTGGCTGATTTTTCGCTTTCTTCGGATTTAATTGAGGAAACTTTTTCAATAGGTTCAGAAGTGCCTTCTTTAACTTCTAAATCAGAAGATGTAATTTCTTCATGCTTTGGCAGTAACAATTTTGAATCTTCG

>BPA_9403

TGCTGCTGTTGTTGTTGTTGCTGTTGCTGATGCTGCTGCTGTTGTTGTTGGCTATTGTTACTGCTGCTACAAATGCTATTAATGTTATTGCGATAGTTATTATTAGGATTTATTACGTTGCCCAATTCTTTGTCCATATCAACGGTGTGAACCGAACGTCTCAAAAACATTTGACTTTGTGGCAGATTTTGTACCACTGAGGGTGACAACGGTGATT

>BPA_9415

CTCACATTCAGCCACGTTAATTGTGGAGGAGGCGGAAGGTGTTAGTACCACTTGTCCTAATGATTGCTCTGGTCGGGGCAGTTGTTATTTGGGTAAATGTGATTGTATTGATGGCTATCAAGGATCGGATTGTTCAAAAAGCGTCTGTCCAGTTTTATGTTCTGCCCATGGTCATTATGGAGGTGGTGTATGCCACTGTGAGGAGGGCTGGAAAGGTTCTGAATGTGATATACCTG

>BPA_9416

TTTTATCTGAAGATGGAGCCATTCGACGTGTATTGGCTTGGGAGCAAATGTCAGCTGATTCATCTGCTCCATCAGCACAATGATCCCAGCCATCACACAAAAGAGCCGCGGATATACAAAGCTTATTAATGGGGCACTGAAATTCACCCGGCCGCTTACAGCAGTCGGCCTCATCGTGACCATTGGCACAATTGGTAGTGCCA

>BPA_9420

TAATTATTAGGTTTTTAGCTGGTTTTTCTTGAACAACTGCTGGAGGGTGCATATGTTGGTTCCAATGCATCGCGGCCCATGGTGCACCACCAGGACCACCAGGCGCTCCAGGAAAACCTCCACCATAACCTGGTGGAGGCATACCGAAAGGCGGTTGATTAAAATTGGGTGGATGCATTCCAGCCCCGAAAAACGGTGGAGGCTGTT

>BPA_9441

ATTTCGCTTTATCGGTTGTTACATCTAATTTATTTAATATATCACTCATCTGCATTGAATATTCACACAAAACAATAATCCTCCCGACTTTGAAAGAACAATAACATAATTGATCAATTGCTATGACCAAAAAACAGGCAGCAAATATAATACAAATCGGTAGAATGTTTTTAGCTTGTTGACATAATTTCTTATAAAATATAGCTCTA

>BPA_9450

TTGTGGATGTGAAGTACCAGTAAGGCATGCAGCCGTGCCAAAACTGCTTGTTCCAGGTTGTGCAGATTTCATAGCATATATTTTACTTTGTGCTCTTGCATTTGGCAATACACAAATAGATGTGACACGAACCCATTCATACGAGCTATCACTATTATTTGACACATCGGTACTCTTTTGGTTGTTATAGCACAATATTTCGTTCATTATAGAAGCAATGTTAAAACATAGCATTTGTTTTAGGTGT

>BPA_9462

GTCAAAGAGCTAACCCCACTATCAGTAGAATCAGTGGACGAGGAATTGCCAGATGTCTTCACCTGCTCACTGTCACGACTCTTAATCTCAGGCAAATCCAAAATAGGCTCCAAAGTGGAAATTGGTATGTGAGGCCTTTCGGGAATAACCAAACCTCTAAGTTTAGATAAGCTGCGACGTCTTTGTTCCAAAATATTGGTGATAGCTGCTTTGCG

>BPA_9473

TATTTTCCAAAAGTGTTTTCGGAGTATTTGGTCGATTAATAATAATGATTAATTCTTTAAGTACCAAATGGATATATTGACGAGTTTCTTCACCTAATTTCATACAAATTTCTCCAATGGCCCAAGTGGCATTATTACATACAGAAATATAATCGGGGTTTAAATTTTGACCCAAAATAGGGAAAAATTCAGCCATGAAAGGGTGTACATGTTGGAAACAAGCTTTAGTTAAATCACCCAATA

>BPA_9475

TCCATTATTTTTAAAACTTCAGCATATGACTTGTTATGGAATGATGGATGTATAATAACTCTCTTAACATACTGCTGTTTTGATCTAACTTGTTTCTGATCGTTTTCTTTTGCATTATCCTTTTCCTCTTGTTCATAATCGTAAAATGGATCTCTTCTGGGACGCCATTCATGATTCTTATCCATTAAATCAGAGCTTTTAGAAGAACAGTCAACAGAAAAAC

>BPA_9493

AAATTCCAATCTATCGAATAGCAACAACAGTACAAGCACAACAACAGCAGGTGGCAATATAACGCCCGATAAACCATCCAGGGCTGGCACATTATCGAGCAACACTGCAACACAACAACAGCAACAGTTACAGCAATCTTTAGTCAGCAATACTACCACCATACAACAGCAACAACAACAGCATTCTTCTGTCACCGTTACCAGTTATCAAATGATGCAGAAATCACAAC

>BPA_9498

ACTCATACCCCAGAACCTGGTCAGTTACAACGAGATTTACAAAATGAAGAGGAGAAAAATCTACGTTTTAGACTTCCTTCGGCAGATGTCAGTAATGCTACACAAATACAAATAGAAGTTACAAAACATGATGATGATGTAATTGAAGAAATGGAAATATCTCATGAGGTGAATGATAAAAGTTCTTGTATTCGTGCAGCTGGTTCTACGAAAATAAAAC

>BPA_9503

TAACCTCCACCGGGACCAACTGAAGCTGCAGCAGCTGAGTAGTTGGGAGCATAACGATTACCCTTATTGCCCTTATAGACGTAGTGGTTTCGACGTCTGATTTGATCGAAGAAAGCAGCTTGTTGTTCATCATAATCTGGTATGTCATTAGGCAGATTAGAGGCATCAGCTGTCACCGTTGCATCACCCAAAGTAAATGTTGAAGC

>BPA_9504

AAAATATTGAAAACCATGTTGGCAAATTTTTCTACAAGATGTTGACCGACAATTTGGGAACAATAGACGTTTAAGAAAATTTATTGAAATTTTTTGCCAAACAATTTTTACTTTTTGTCTTTGCCACAGTTCGTCATTTTTCACTGACTGTCGTCGTCGTTCTCATCGTTTGTTCGTATGTGTGTTGGTGCTACTTTTATGTTGTTGCTGCTGCTGTTGTT

>BPA_9521

CCGTTGTCGGTGTATGGCAAGAAAGAAATGCTGAATCCGCTATTGAGGCCCTGAAAGAATATGAACCTGAAATGGGCAAGATTGTCCGTCAAGACAAATCAGGTGTCCAAAAGGTTAGAGCGAAGGAAATCGTGCCTGGAGATATTGTAGAAGTGTCTGTGGGTGACAAGATCCCAGCAGATATTAGACTTACACATATCTACTCGACCACCTTGCGTATCG

>BPA_9527

TCGAAGGGCATTTCAACATCGACAATATTGCCGAATTGACTGAAATAGGTTTTGATTTCATCATCACTGATTTCGGTGGTCAAACCACCGACAAAGATTTTACCATGACGAGCTTTAGCTTTTTTGGGATCAACTTTCTTGTTGTTAATTACATGATCACCAGCTTCATTAACTTTGTCAATAGCATCTGTAGTTGTGAATACAATAAATGCAAATCCACGTGAACGACCAGTTTGTGGATCTGTTTTAACATTGATGCTTTCAA

>BPA_9535

CGATGAAATGCCAACCGATTTAAGTGTCAGCCATCAGTCTCAACATCAACACCACAATGAACAGAACAGCAATAATCTTCAACAACAACAACATCAACAACAAAACTTAACACCTCTCAATTATCATCATTACCAAAGTTCTCCCTCACCTCAACATCAACATCAACTACAACAACAGCATCAGCAGCAGCAATATCATTATCTCAATACCTC

>BPA_9555

GGCAAATGTTACGTGCATCCAACCAATCGACCTCTAAACTACGAGTAGGAGCATGTTCCCAACTAAAGAAGTAAGAGTGAGAAACACCACGAGCATCACGATAAGAGGCATGTCTTACACGATTAGCACAACTTCTGGGATCAGGTAACGCTAAACGTCTTTGAGCCAAAGTAACGGTGGCACCCAAAGCCAATAACAACATGGTACGTAGCACCGA

>BPA_9563

GGAGCTTTTATATCAAAAGCTGTTACCATTACTTCCTGTAGTGTTATAGAAAAACGTCCCAATTTAAATCTCACGCGTTATAGACGTATAGTCTTTGTGATTTGTAACTTGGCCACTATATTGTTTACGTTGGGTATAATACTGTCCTCCTGCGAACAAAGAATTATGGTGGTTTTATTTACCATTTGTATAGGCATCACCTCGGATATGGCTTTTTCGGGAGTGTATTGGCCTAGTTTATTATATGTGGCACCAGCTTATGCAGGTCTGATATCTGGC

>BPA_9566

TAATCGATAGCAATTTCAGCATAAATAAAGGTTTAATGGTACATTTTACCGCCGAACAAGCTAAAGAATTTTTGAATGAAAATCATCAAGAAGATGTACAAACATCAGTACTTATGAATCAATTGCTAAAGGGACCCTCTATAGGTTTTGAAATAACTGCCGATAACGCTGTGGAAAAGATAAAGAAGTGCTTGGGTTCTTCGCAATATTCCAGTGAAATGCCAAA

>BPA_9578

ATCGTTCTACAAACATTGACTGATTGTTGGGTATAAATCCAAGGAATGCGTTTTTTTCAGCCGTGTAAAGCAAAATCAAAACCTTAATATCACATGCTGGCATATTGGGTGGTGTTGTAAAGTGTACACAGCCAGCAAATATTGTTGTCATATTTTTGGCCAAAGCATCTAGGGCTTCACAAGGTTGTGGCTTAAAGACTACCATTTTGGAG

>BPA_9594

GTCAAAATTTCAGTGCTGAAAAATTATTGGGACAGCATACACCACAACCTCAGCAACAACAACCACCACCACAACAAACGATTATGGGTGGACATAATGTACAACCGCAACAGCAGCAGCAACAACCGCAACATCCATTATCAATTCCCCAACAAGCACAACAACAAAGACCAACACCACCTCCCCACATACAAGCCCAGCAACAACAGCCACCACAGCAGCAGCAACAACAACCACCACCACCACTTCAACA

>BPA_9648

GCCACCACGTCCCCGGCCACCAAAACCACCCAAACGACGTCCATCGTTGAAATTGAATTGGATGTCCAAAACACGTTGTTGTCTACCCACACGTTGGGGATACATGGCAGGATCAAATTCTAATTCTTCTTCGCTTTCGGTACCCTTCTTTTCCTTGGTAGCTTTGCTATTATTCAAAACAATCATTTTCTTCCATTTGGTAGTATCTTCACCTT

>BPA_9661

TCAACGTTAAGAGAATATCAATTGATTATTGATTGGCTGGAAGCTTGTTATGATGGTAAATTAATGGGACCCCAGGTGGGTCATACTACTGATCGCACAGTGGGATGGGAAAATACTTTATTTCAATTGAAAAAACAACAGCATATTGCTTTTGGCTCGGGAGCTGAAATCGTTAAATCTTTAGATCCTGATGCCCCTATAAGGGAGAAAAAACCGTTAC

>BPA_9665

ATAAAGCTTCAGGTCGTGATCGTATAGATTATGAACTGAAATGGTTTATACCCCTAAAAGATGTAGCAGTTTTTGAGGAATCCGATGCAAGTGCAGAACTGAAAGAATCTAGTCCAGCCAATATATTGCAGCTAAAAACACAAGCCTGTACCGTAAGAGATCAATTGTTAATGGAGGAGAAGGATGATAAGTCGCGTAAACCGAATGGCTTAAGAGGAGGAGAAAAATATCGTCGAAAATTAGCCGATTTAGAATCACAATTAGTTTTGGCTTCCCCCAATCTAGTGTTTCGCATAGCAA

>BPA_9680

CGCCTGTATGTATGCGTACATGATTCTTGTAAGATGTGGAATTAGCAAATCCTTTGTCACAATGTTCGCAGGTATAATTTTTCTGTTTTGCAGCATTTTTTCTAGGTTGATTTCCTTTCTTCTGATATTTTCTTTTAGGTCTTTTTTCGAGTCGATGATGTCGCATATGTTCTTTCCAAACTTCTATATTATCCAAAACATTTTTACACTCCTGACATGTTATACTTCCATCTTCGTTTACTTCAGGCGATACATGTTCTTTTTTGATTTTCGTTTTTGTTTTTACAACTTTTTTTGGAG

>BPA_9686

TAGCAAACCAGCGGCGGCTGATAATGGAACAGCGTCGAAGGACGAGGTTCATGGAGACGATGCCTCACCCGCTAAAGAACATGTTAAAGGAACAAAACGGCCGGCTGAAAATAAGAACTCAGAATCAAAGAAGGCCAAGAAAGAAAAAGCCCCCGATGCTGACTCAGATGACGAAGAAGTCATCGACGAAGAAGGTAATGAGGGCGACAGTGATATTGAAAGTGACGAATATGATATCCCCTACGATGGTGAAGAAGATGATA

>BPA_9707

CGGGCCTTATCAATCCATGGATTCCAAATAACTGTATCGGGGAAATTGTATTTGTGTATGCGCATTTTCCGACCCGACACAACATTTGTTATAATATGTTCTTGGGGTGTATGCTGATAAATGCGATCGGTCCATTCATTAACCGTTACCACTTCACGACCCTCTTGATACATAGCACCATCACGAGTCTTATCCATAAAGGTACAACCATGCAGTCCAGTTATTTGACAACGTC

>BPA_9713

GAAGCGTGTACTGAAGAGCAATCGGAGGAAACTTTACCGCCTAAGGCACTAATGCTACTAAAACCACTATGTGACAAAGCCGTATTAGCTGATGTTGTAATAATGCTACTGCCATCTAAACTACAACGTGGCATCCTTGCATTTTCCAATGAACTTGTTTCATTCCAACTTTTCTCTATTTCTTTAACTAAATCGTCACTTAATTTTTGTTGAACATCACGAACACTCTTAACGGATTTTGTATCACTATCGGTATCTGTATCGGCGGCTGGCAGTCGC

>BPA_9724

TTTAATTAAAAATGGCTACGGTTTTGTGGAGTTTGAAGACTATCGCGATGCAGATGACGCGGTATATGAACTAAATGGGAAAGAATTATTAGGTGAAAGAGTTGTGGTAGAACCTGCTCGAGGAACTGCCCGAGGTAGCCATAGGGACCGTTACGATCGCTATGACGGTGGTCGTCGTGGTGGCGGACGTTACAATGACAAAAACAAAAACAACAGATCAAATTCCC

>BPA_9728

ATGGAATGGTGTGATAAATATCAAGCTCAAATTGTGGTTTTGGCAGCACAAATTCTATGGTCCGAAGATGTTGAAGCAGGTTTACAACAAGCCTCTGAGGCCAATAGCACCAAACCCTTGCAACGTGTTTTGGGTACAGTAGAGTCTACACTAAATGTTTTGGCTGATTCGGTTTTGCAAGAACAGCCTCCTTTGCGTAGAAAGAAATTGGAACATTTGATCAATGAATTTGTACACAAACGTACTGTAACCAGACGTTTGATTGCTAATGGCGTCA

>BPA_9733

AGGTAGAGAGCGTGAACGGGAACGTGAACGGGAGAGAGATATTGATAGACAAAAAGGAGGAGATCACAACAGTAGTTTAACGCGAGGTCGACCACGAGTAAGGCGGTCTTTTGAGGAGCGTGATAAAAGGCGCTCTACTTCCGAAAACCACAATGAAGAACGCAACTCAAGTCAAGTAGGTAAAATAAGCACTACAACACCATCTACCACGAATCATGCACGTAGCAGTAGCAAAG

>BPA_9735

CTCCCAATTTAAGCCATTGCGAAGGAGCCCATAAAAACAATTGCATGATATTAGCAGCTATATCGGGACTGACTCTTTTGGAAGGATTGGGTGCTAAAATATTATAGATTAACTGCTGCACTAATGCCGGGCATTCCTCATTTAAGGCGGGTAAATCTTCATGTTTATAATCCGAATTACGTAGAGAAAATGTTTGTTCTCCCTTGGCCACATTGA

>BPA_9737

TAGAATTAGCAGATTGGATGGCTACACGTGGTGCAAGAAATATTGTTTTAAGTTCTAGTAGAGGAATATCTAAAGGCTACCAATCTTATCGCATTGCGTTGTGGAAAACTTATGGGTGTTCTGTAAAAGTGAGTAGAAGTGACATTACTACATATGAAGGTTGTCAAAAATTATTAGAAGAAGCCGAAGAATTAGCTCCAGTTGGAGGCATATTCAATTTAGCTGTTGTTTTGC

>BPA_9746

TTTCTATACGTCTGGCTCGTGTTAAATAACGATTAAAATGAAACTCTTTTTCCAATTCAGTCAACTGTTTATTGGTGAAATTAGTACGTCCCGAATTATTGGTGGCTGCTAGACCACAAGAGATTTGTACCATACTGTTGGGACTGTGGCTGCCGGAAAGTAAAACAGTTGGGGTACCTTGTCCACCCATTTGATGGGCCGCCAAT

>BPA_9762

GGGTTTCATTTGCGGTCGACGAAGAATTTTTGAGAGAACATGGAGCGCCTCTGCAACCATTTCATTTGAAGTGGCATCTAGCATGTTTAGCAACATTTTCATGATGGCGCGGAAGTGTTTATTTGGCAATTCACAGTTGCCGCCTTTTATGCATATACCGAGATTAGTGAGAGCGCTTTGCACGTGAGGCACGGGCATGTCGGGTGTCACATTTAATGCCACCTTAATAATTTCACTCTCAT

>BPA_9773

ATTGTATTTCAGTTTTCGCAGTTTTTCGCCTAGGTAAAGTTGCTGACTTGAGCGTAATAGATGCAGTTGACGTTTTCATAGTACCACCTATTGAATTGTCACCTGGCCCTTGAGAAACTGGCTGAATGGCATTTCCAATAATTGCACGAGCATTCATCAACTTCCGTTCGCGATCGTTTACTGTTTTAGTGGAATGAGATTCACTAACATGAATATTATCAG

>BPA_9789

CACATGAGTTTCCACAATATTCGACATTGCGCGCCACTTCCCTTCGACGTTAATTTCAGGACGTAATATTTTTTTATTTTTTTCTTTCGCCTCTATTCTTTGCTTTTTTTTTTCTTTTTTTGCTCAAACAAGACACACTCAATAGCCGAAGTTCATACAAAAATCGATTTTATTTTGGCCGCCTTGTTATCACACACAAA

>BPA_9804

GGCGAATTGAAAAGCTAAATACAGTCATTGAAAATCTTCAAGAATTGTGCAGCGAGGAATATACAGATGAGCTAGATAGGATATTACATATGTTAAATTTATTAGGGTCACATGCGAAAAATTCAGATTCTTTAAACACAGGTGGTTGTTTTGAATGGGTTGATTCGAAAATAGTTAAATCACTTAAGAATGGTCAATACATTCTGCTCGAACATGTAAATCTTTGCTCGTCCGCAGTCA

>BPA_9810

GCATAGCAAATGTTTACTAGAGGTGTTAAATCATTTTGGCAAATTGGACATTTTGGTAAATAACGCAGGACGTTCTCAACGTGCCAATTGGGAGGATATTGATATCCAGGTGGATAGAGAACTCTTCGAATTGGATGTATTTTCTGTTTTACATCTAAGTCGTCTGGTGGTACATTACTTTGTGGAACAAGCTGGTGGTA

>BPA_9880

CACAACCCGATGTTGAAGTATCTTGGTATAAGAAGGGTAAACCTATTAAACCCAACAAGAAGCACGAAGTGTTTGTGGAGGGTACTGTCAGACGTCTGGTTATTCATGATGCTGGTGACGACGATGCCGGTGAAGTTACTTGTACAGCGGCCAATGTTACTACTGGTACAAACTTGTGTGTAGAAGAACCTAAGACTCCACCAATTA

>BPA_9893

GCAACAACCTCAACACAGAATGGCAACAATCATCTTATGCCTGATATAATTAATGAATTAGAATTAAGACGTCATCAACTTATATTGGATAATGAGCATTTAAAACAATATGAACATGTTACTGCGGTACCTTATATGTATGTACGAGAGATACCTAATAAACCGCCTCCACCATATGTACCGCCAGCTCATGGCAGTCCTATGACTACAATTTTTC

>BPA_9898

TAACAGCTACCTTACGCAATACACGACGCATAATTTTACCCGAACGGGTCTTTGGCAAACCGGGAGCATGTTGAACTACATCAGGCATAGCAAATGGACCAATACGTTCTCGTACTAGTTTCTTTAATTCAGCCACCAATTTGGGATCAAACTTCTCATTTTCATTGGTGGTAATGAAACAATACAAACATTGTCCCTTTACGGAATGTGG

>BPA_9916

TTGCTATTTATGAAAAAGAAGTGGGAGTGCATAAAATATGGGAAGATTTTGTAAGGGAAAGTGAGGATGCATGCACACATCATCATTCCAGATCAAAGAAATCGAAAAAGAATAAAAAGCACAAGAAACGTTTAAGATCAAGTTCAAGGTCTGATATAGAAAATGATCCCATGGAAATTGAAAGAAGTCGTAAAAAGAAATCGAGATCAAGATCGCTCTCGA

>BPA_9927

TTTGTTGTGCAATTATCGGTGTCATGTAAAGTGATTGATTTCACACCACCCAGGATGACATTTTTTGCAATTTCCAAGCCAAGACCATTCAAACCGGATAACAAGATATCTGAATTAGCCATACGACGCATAGCATCATGACCCAACACATACAATTGGCGCGAGTATAAAGATTCATCGATATCATTGCCAGTGGATGATCCGGCAGATCCACC

>BPA_9929

CGACAGAACATTAGCAAACTGGCAAAGGAGATGGCAGAGTACTCTGAAAGGGCGATGGACGCATAGATTAATACCGGATATAAAAACGTGGATAGAACGTGGACATGGGGAGGTGAACTACCACCTAACTCAATTTCTTTCAGGACACGGAGGATACAGAAGCTATCTGTATAAGATAGGATATGACGAATCTCCAAACTGTCCGAT

>BPA_9934

GTACGATCTGTTAGTATCGAATCAAAAGGCGAATATATCGTTTCAGGTTCCGACGATAAAACAGTTAAGATTTGGGAAATTGCCACTGGACGTTGTATACGTACTATTGAAACTGATGACGTTGTACGTTGTGTTGCCTGGTGTCCAAATGCAAAATTATCTATAGTTGCAGTAGCTTCCGGCAATCGTTTGTTACTTATTAATCCAAAAGTTGGC

>BPA_9948

AGGCATGTCATCTACAAAATGTAAAATTCCACTTGGTTCTTTCTTCGGTCATATCGTTATCTTGGAATCGGATGGATATGGCGATATTCTTTTGGATTACATGAATACTAATTCCCAGAACATTGAAGCTCAGATAAATGCTTTACACTCATTATACGAAGATGTACATTGCCGTTATAGTCTTGCAAGTTGTAAACTATTAGATTTGTTAACCCCTCTTCTGAACGCAGATGTATCTAAT

>BPA_9949

AAATTTATAGATCCCTTGTAATAATTCTGGACATGTATTTAAACTCATTTCTTGTTTTTGAATTTCATACTGCGGCTTTAGTTCAATATTATCAAGATTTGTTTTCTCGATAGCCTGAACTAATTGTTTAGTTAAATTGTCATCACGCACAGATAATAAATTTTTTGATTCACTTGCTACCAGAGTGTGAAACAGTAGAGTTCGATTCAATGATGATGTATTTCCAAGGGATTCTGACACTGGCAACTCACTTAGCAAGTCAGATAAGCTTGTTAAGCCTGCTAATGTAGTTACTGGTACACTTGGAATATCACGTTCGCTCATGTTGAACCCACTTTCTATTGTAAATTTCACTGGT

>BPA_9966

TCACAAGCTTTTTGCAAGCGCATACGTCGATATTCAAAGCGATCTTTCCATTCATTTAACTCATCCTGTCCGTGAGCCTCCACACATTGGTTGCCATAGTGACACAACTGTGATTCACTCCACGATTCACATAAACTATATGAATCTAAAGTAAAACCTCGAGGAGGTGGTCGCCACTTCCAATCTCTACCCTCATCGGACATAATTACATTTTGATGGGCTTCGGTCTGACAATGTTGTCTTAACTCATTGCGTGAACTAAAT

>BPA_9990

CATTCCATTATAACTAATTTGCCATTATTTTCTGAAACAATGCGTGGCTTTTCAACAAACGTCGGCCCTTCGCCTTCGGGTTCCGGTTCAGCTTCAATATTTAAGTTTAGATTCGCGTTCGATTCACCATATTCGTTTTTAACATTGCATCTATAAGTGCCACCGTCAGATGCGCCGGGATCCTTGATTTCCAAAGTTAACTCGTAAG

>BPA_9991

AAAAAAATTATGTTGAGGCAGCAAACTAAAACATCACATTTATTGAGGCTTCCTTCTGAAAGAGCAACCTTCTGTGAGCTTAGCACGACCACCGGTTGGCTGGCACAAGATGGTAGCACATCCCGCACATACAACAACGCCTTGAGCATGGCTAAAGACAGTGGTGATTCTGTAGCAGCCAGGGCATTTAACGTCCATGAAGTAGGAGTTGGGATGTTGCACCAAGCGCTTCAACTTGTGTTTACGCTTTTCTTGAGCGGGCAAAGGATGT

>BPA_9995

TAAAGAAACCACCACCTGAGCCAACAGAGGTCTAATAAGCCAATACCTTTTATAGCCTGACCTAAAAGAAGATTCAGATAATCGGAGTTCAGCTTGAATATCAGTAATTTTATAGTTAGTTGGTGCTTCATCGGGACGATAAAATTCATAATCGGGATCACCGTAATCGTAATAATAATGCAAATAAAGCATGACCAAAACAGCTGTGGAGATA

>BPA_9998

GAATATTTAGAAAAAACGTCACCTTCGCTATCACATAAATGTGCAGACAATCTGCTGCAAGATTCAGCAAATGTTAGTACCAATACATCAGTAGTTGCAGCAGCAGAAACTAAGGTAACAGTAACCGAAGTTACCCCTCCACAGACGCTTGTTACGCCACCCACCATAATTACACCACAACAACAACAAATGAATGCAACCACCGACAATTCTGCA

>BPA_10006

ACAACAATTAAACGTTGTGGTAAAGCAGGACGTGATGGTGGTCCTGGTTCTTCAATTTGTTGACGTTGATCACGCGATGGTGGTTTCTGATTACGATTAGCCTGTGGCTGTTGAGCTTGTGCCGCTTGTTGTTGTGCTTGAGCTGCAGCTTGGGCAGCTGCTACTGCGGCCATTTGTTGTTGCTGCTGCTGTTGTTCAGCAGCTATGCGACCCTCTTGTATCTGTTGGAAATTACGACGCAAAGTATCACCGGGTGCTTGAATAATTG

>BPA_10011

TATAACGGCGGCATTTTCTTCGGTCTTAACACCATCCATTTCCATGCGATTTCGAGAGGCTGCCGCTGCTTTAGCCTGTTGATAATCAATATCACCAGTTTGTAAACGTTTGCTTTCGGCCAAATTACTAGTGGCCTGTTCCGAACTGAAACCATCGGTAACAACTTTGGTCTTCGATTCTGTTTTAGTACGTTTCTCTTCGAATTTAACAGTATCAACAATGGTACCCTTCGAACCACTCAAACCATTTGTAGACAATTGATTAGAGGCTTTCACAAGATCCATATTTAAATTCATATGATCGTCATCATGATCATGTTCATCGGGAAATGTTATATGAGGTTCAGTATTGCCATCAACAATATTCTCCAAAGAAGAGCGTAATTTTTTCTTAGTAGCGGCGAGCGAAAAGAA

>BPA_10012

ACGCGTTCTCTAAATAGGAAAAATCAATTTCTAAAATGGCCTGCTGGGAAGGAAAGAAATACAAATTGGACAAGAGCGAAAACTTCGATGAATACATGAAGGAATTGGGTGTTGGTATGGTACTTCGCAAGATGGGCAACTCTGTAACGCCAACCGTTGAATTGAAGAAAGAAGGTGATGTATACACATTCACTACATCCTCCACTTTCAAGACTTCTAGCATTACCTTCAAATTGGGAGAGGAATTCGATGAAGAAACTTTGGATGGCCGTAAAGTTAAATCTATCTGCACTATGGATGGAAACAAATTGGTCCAAGAACAGAAAGGTGACAAACCTTCAACCATTGTGCGTGAATTCACAGATTC

>BPA_10033

CAAAGAACAAACTGATATCTCTGAATCAGATAAGGGTAATGGTACCGGTTCTTGTGGTAACAAAGATATGTCATAAGCACACATATCTTGTGCATACCATAATGGTGGTTCATTCAACCAAATATTGTCTTCATTGTTTTCGTCCTCTTCCACATCGATATTTTCTTCCATGAAGTCTTGTTCGTGAACTTTCTTCGATTGC

>BPA_10036

GTAATTCCGACGGTCGCTGAACCACAATTGCCCGGATAGCCTTTGTCGTTGTCGGGTGGATCGCAATTTTCCTCATTGATAATGGCGTCTAAAACACCATCCCAACCGGCTGAGGTAGACATCTGAAAGAGCAATATCATACTTTGGCCAAATGTCTTAAAATTATACACGTCATTAATGCCACTCTTTTCTTTGACATGCATAAAGAAGGACATCCCAAAGATGGCAAAGATGAAC

>BPA_10042

GGAGGCCGCAGTCGTGACAATAATCCCTTCATAAACCCCTGGGCAAATGATGATGGTGGTTTTGGCAATGGTGGCGGCGGTAATGGAGGCTTTGGAGGTGGTAACTTTGGTGGTGGCGGCGGTTTTGGAGGTGGTAACTTTGGCGGTAACAATGGTTTTGGTGGAAATGGTCCCATGGGTGGTAATAATTTCGGCAATAATGGTGGCGGCTTTGGTAATCAAGGAGGCTTTGGTAACAACGGTTTTGGTGGTGGAGGC

>BPA_10053

TGTCATTATGCCTCATCAAGTAACTAGCCGTAATTTAAGTTTAGCTGTTTCTGATCGTTTTTGGAAAATGGTGAGAGAAACCATAGAACAACAAGCAGATGCTTTTAAGGCCACTAGATTTAATTTAGAAACAGAATGGAAAAACAATTTTCCCAGATTGCGGGAATCTGGTCGTGATGAGTTATTTGATAAAGCTAAAGGAGAAATATTAGATGAAGTTG

>BPA_10057

TGTGCAATATCACCAGCGGAATCAGTTGGATTAAATAGGAATTCTTTTGTTTTGCCGCTCACAAGTATTAAACGTAAATTTATCTTGTCCGAGGGAATAGTACGCGATTTTTGCGTAATCACGGCCGGAGTAACAATATTAGTATTTCCTTGGCCTACACCAGATGCAGTGCCACCACCACCACCGTTGCATCCCCCACCAGCAACACTATTATTGTGGCCACCTACAACAGCAGCAGCAGCTGATACCGAATTAGAACTTGCCGATGAGCAGCAAGATGAGGAGGACGAAGAGC

>BPA_10061

TTTATTCATAAACCCAGTCAACGGCAGCGGGTTTCCAGTCAACGAGTTCTTTCTCATTGAACCACAAGGCAATTTCCTTTTGGGCAGATTCGACAGCATCGGAACCGTGAATAATGTTGCGACCAACTTGGATACAGAAATCACCACGGATGGTACCGGGCAAAGAGTCAGCAGGGTTGGTGGCGCCCAACATTTGACGACCAGTCTTGACAACATTCAAACCCTCCCACACCATGGGAACCACAGGGCCGGAGCTCATGTAGTTGACCAAACCGGGGAAGAAGGGACGAGCAGACAAATCGGCGTAGTGTTTCTCCAACAATTCCTTGGAGGCCCACATGAATTTCATGGCAACCAATTTGAAGCCTTTTTGCTCAAAACGTTCGATGATTTTACCAACGAGTCCACGTTGGACACCATCGGGTTTAACCATGATGAAAGTACGCTCTGTGTTGCCAGCCATTGCGTTTGATATCAAAGAGAAGAAT

>BPA_10069

TTTTGGTGCCGTAGGGTTGCATAAGATTGACAGCTCGACAAATGTAATTTCCTGCATTATCTGCTCTTACCGACACTAAAGTTAATAGTTCACCCATGAAACGAAAGTCTGAAGCTCCTTCTTTCAACCATTCTATAGTTATGGGAGGAGGATTAGCGGTAACATTGCAACGCACCAACACGGTTTCACCCTCTTCGGCTTCATGAGTTTTG

>BPA_10079

GTTGGCCTCAGCTTCACTGCGTCGCATGTACCAAAATTTGCCCGAAGCTCAAATTGTTTTACGCGCAATTGTTGATGTAAATTTGCCAAAATTCTTGGAACAAGATGTTTCATTATTTGTTGGTATTTACACGGATCTCTTTCCCGGCACCGAATTGCCAATGCCATCTCGTGAGGATATAATGAAATGGTTAATTAAAATATTGAACGAAAG

>BPA_10086

TTGTGTTGAGGTTCCTACTGTTAAATTGGATGAAATAGTAGATGTCGAAATTGGTCTTAGAGGATGACTCCGATCTATTCCAACACCACGGCGTCGATCGTCAAACATTTGGCGTACCTTTCCGTTGCCAATGACACTTTTCGTAACCTGAGATATATTCCCTCCAGTCATTGCTAACTTCTTCTGCTCGCGTTCTAACATTTGTTTCTTCTGAAATTTTAACTGCATTTCAGCAAGCCGTGAGTTAGATGTTGTACCTTGTGATGCCATCGCCAAATATGAAGTATGTAAATAAAAATACAATACTGATGATGATCTCTGTAATT

>BPA_10092

ATTTACAAACACGCTCTCTTACCAGCTGTACCAGAGAGGTATACAAGGTTGCTGTGGGGAGACCTTTATCGTTGAGTAAAGAGAAATAACCTGGAAATTCTTGTGGTATCATGACACCCTTTTTTCTATTCGCTGTTGTTGTTTTGGCATTATGTCCATGATAAATGTGACGCTGTCGCACCAATCGATATAGTAAATATAATTCACCCTGTTCCTCATCGTTATGATTAGCATCATTTGGTGATTGATTACCCTCAATTGTCCC

>BPA_10098

CAACTTCTGTACAACGCGTACCTAAGGGATCACGAGCATCTACGACTTCCAAAACGACATCGGCATTTTCCAAAACCTTTTTAAATTCTTTGAAGTATTGTTTTAGTGATTGCTCTTTAGAATTCGATTCTTTGTATTGTTTCTCATTATTTTCGGTATCGTTTTCGTGCATTATTGAATGCACTGTTCCACGCATTCCAGCATCATCTACTAAAGCTTCTAAAGTTTTTAAACGATTTTGTTCTTTCTCCATTTTAAAAGCTTCACGTCTTTTCAGGCGCTCCTCCTCTTTGCGTCTCTTTGCTTCTTCTACTTCTTTCAATATATCTTCTTTGAAAGGACAAATGTTTGGAATCTGTATCAACTTTTGTTTTTTACTGCCCTTTTTGGGATTCTTCTTCGCTTCACGTCTTACTTTTCTATTGTGTTCAGTACACACAGGGACAGCAAAA

>BPA_10102

CTGCATCCATATCGACTAGAGCCAATTTACGTTGTTGTCTTTCCATTTGTTCACGTAATGATTGTTCTCTGGCCTCTTGTAAACGTCGTTTCTTCTCTTCTTCCTCACGTTGTCGTATAATCTCTTGATGTGCCTGTACAAAGGCATCTTTAAAGGTTTTGATGTCAGCAAAGAATTCTTCCATTGTATATTTGCTGGGTTCAAAAGCGAAATATTCACCCAAATCTTTGTACAGTTTTTCCATTTGTACTTGCATTTTGCC

>BPA_10116

CATCTTTATAGTGTTTTTCCAAAAATTCACGTACTGTTTTGGCTGAACGACCGGTAGCATTTGCTTTCCATTCATAGTAAATACCTGAAGGTTCTGTTTGATACAAATGGGGTGTACCATCAAAATCGAAACCACCTATAAGGCAGGAAATACCGAAAGGACGACGACCATTACTTTGAGTATATTTCTGTTTCAACTGAGCAATGTATCTGGTAATGTATTCCAAGGTTACAGGATCTTCTACATTTAACTTATGACTTTG

>BPA_10132

AAAATGCGAAGAAATCACCTACGGGTGATATGAAATGGGCTAGTGATAGTGAAAAGAATCCATTACAAGAAATAAAGGATTATGATATTGACACAATACGACGTATAGTCGAGGAACACACATTCCAAGTGATAGAAGAAGCCCAACAAAATATTGAAAATAACGATATGGTGCGAAAACTTCAAGCCCAAGTTACATCA

>BPA_10134

CAACAATTTAATAACCAGCCAATTCGGCGAATGTGGAGTCCATTTCGTCGGCCAAAGACTTGTATCTGTCCTTGTTGATGCCCAATTCGTCTTCTAGCCTGTCGACTTCCTTTTGGAGACGCTTAACGTGCTTCTCAGCATGTTCGGCGCGTTGTTCGGCTTCCTTCAATTTAACGGTCAAAGTCTTCATTTCACGTTTGAATTCTTCAACACGTTGGTTGGCCTTTTCTTC

>BPA_10136

GTATAAGGGAGCAAATATACAATTGTTGGATTTGCCGGGTATTATTGAAGGTGCTGCACAGGGTAAAGGTCGTGGTCGTCAAGTTATCGCTGTTGCCCGTACGGCCGATTTAGTTGTCATGATGTTAGATGCCACCAAACCCAATGTTCATCGTGAGCTGCTGGAACGTGAATTGGAATCTGTGGGTATACGTTTGAATAAACGTAAACCAAATATTTATTTCAAACAAAAGAAAGGTGGTGGTT

>BPA_10141

TTAGAATCGTTAAACACAAATAATAGAACTTCTCATCAGTCAATAAATACTAATTTAAGTAAATCATTATCTACTGGCCACAACCAAACTACTTCTGAAACAGAGAATATAAATACATTGGATTCGCCTGATAATAAAATTTGTCAAAATCGAATAAGTAATAACTTAATGAAGCCAACAACTTCTGGTCACACAGCAAAGAGTCC

>BPA_10143

CAACCTCCTGCACCAGCTGCTACTAAACCATCATTTATCTGTTCGGTATTGTTACCATACGAAGCATTAGTATTTGTATTCGTATTATTTGTCGTTGGTGTCTGACAATTACGAGATTCAACAGAATTTACACGAATTGCAGTTGTCTCATCGAAACCACAATTTGGTGAAATTTGTCTAGATTGTTCAGTGGTCGGATGTGAAGATGATAAATGTTGTTGCTGCT

>BPA_10157

GCAAATACGGCAATTATTTGTGCATACAATATGTTGTGCTTTATTTTCCATTTGTGGGCTGCTATGCAAAAAGAAATGTGCATCAAGATGCTTTTCACAATAATTGTTCAAAGGCATTTTGGTGTAATTCTTGTTGTTATTGTTATTGTTATTATTTTTGTGCAAATCTTTGTGTCAATTTATGCTCGAAATGCGACTGCTTAATAAACTTTTATTATTATTAAAATAGAC

>BPA_10164

TTTTTCCACTAGTTCACGTTCACCGCTGCGATAGGTTAAAAGTTGGGAAAATTCAAAGGGGGCAGTTCCCTCACTCCACAATTCCAAAAGAGCGCAACCAGCTGAAAATATATCCATAGCAGGTTGTAAATTATTACCCGAATAATCAGGGCCCAATCTTATCAAAGAATCTGTAGGAAACATAGAAGCATTGCCATCGGATGTTTCATCAGATGATAA

>BPA_10184

AACACTTTCAATTACTATTTAAATTCAATAATAAATATCCATTTGATTCGCCAGAAGTAACATTTATTGGCAACAATATACCCGTACATCCACATGTCTATTCAAATGGTCATATCTGCCTTTCAATATTAACTGAAGACTGGTCACCGGCTTTATCCGTTCAGTCGGTATGTTTAAGTATAGCATCAATGTTAAGTAGTT

>BPA_10198

TGTAACTTACGTTGTTGTTGCTGTTGCAACTGCAATTGATGGAATTGTTGGACAGAAACGTTACGAATTTGTTGGGTAGTTGGTTGTTGTAACACTTGAATTGTGGTTAGTTGTTGTTGCGATTGTGGCTGTTGTTGTTGTGTAAGAGGAGCTGCCGCAGTGGGATGATTGGATGTGGATGTTGTTTGTAACTGATGTTGTTGTGTTTGCTGTTGAACACTTAACGGT

>BPA_10202

GTGTTAGAGGAGCAGCAACGTAATGCGGCCAAAGAACGTAAGGCCACATTTGCCGAATGGGAACCGGCACATTTTCAACAAGATCTAATAACGGGCAAGTGGATTTATAAATATGCTGACTTAAGACCCTGGGATCCTCGCAACGATGTTAAACAATATGAATGGGATTATAAAGTTTTAACAAAAACTCGTCATCAAGCACCTATGGTACGTACCGCCAGCATAGTG

>BPA_10212

TTACAAAGATGCCATGGTTTCTCAGAGTAAATCAGCTGATGTATTGCGTATTCTTAAAGATGCTGCAGATGAAAGAGATTGTGAAAATCAATTAGTTTTATTACTTGGTTATGATTGTTTTGATTTTATCAAACAATTAAAACTTAATCGTCAAATGATTTTATATTGTACAATGTTAGCTTCTGCACAGACAGATAGCGAGAGGCAACGAATACGTGAGAAA

>BPA_10214

TTGCTCTCTCAAATTTACAACCACTTCGTATGAGACCCAATGGGATAAAGGAACAATGTAAAATCTTACATAAATCCTTTAAATGACAATCCACTGTTTGCTGAGAACATTTTTCTGTAGTATTGAGATCCAAAATCTTCAAATCATTACCCAAAGCTTTAGCTACAACATGAGCTATAACCTTAGCTCTTTGTGCCAGATTTTCAAAATCTATATTTCGTTTAATTTTTAAAGGATTCTGAGCTAAACCTTGCAGCTCTAACATATTATTAACCTCTTCCAGATACTTGGGCAAAAACTCATCTCCCGGGGAACGACAATAACACCATTCTTCTTTATCTTCTGCAACAACTTTAGTTTCGTTTTCTTGGGAACAACGAACAGGAACTTTAACAACAAGTTCTGTGGGCGTGTCTGGTCTATTGAAA

>BPA_10218

TAGATCGTGCTGAAAAACTTAAGGAATATCTAAAGAAGGGCAAAAAGAAGCCCGTTAAAGAAGGTGAATCAAGTTCAAAAGATGATAAGGACAAGAAAAGTGATAGTGATTCCGATGGTGAGGATCCGGAGAAGAAAAAACTGCAAAGCAAATTAGAGGGAGCAATTGTTATTGAAAAGCCTAAAGTAAAATGGTCCGATGTGGCTGGTTTGGATGCAGC

>BPA_10219

ACAACCACGGTGGAAGAAGTATTCAACCATATCGAAGAAACGGGGATCTTTGGCAGTGGGTACATCCTTTAGGCGTTCAGGAATTTGGTATTCAGAATAACCACGTGCTTGCAAGGCAGCAGTAGGCAAAGCCTTTACCAAAGTAGCAACATCCTGACGGACGGCAGCTTGACGAGCAATATTTTTCAAATGATACATTTTGTATGTCTAAGTCTACACTCTTCTCGCTCTTTACTAAATTACACACTAAAC

>BPA_10222

AATGAACCAAGTAAACGCTTCATTGTAGGAAAATGACGTAGAACAGTATCGGCTATACATGGCGTTTTAACACGATGTCCATCAATATTTAATTGTTCAGCTTTAGTTTCGTCGGTTTGGCAAATATTTGTAAGCTCTTCTTCTTCTGGTATAGTTTCATCTGTCAATTGCCGCGGTAATTCCGTTTGTATTAGAGACGCGCAA

>BPA_10226

GCAATTATACGTGCAATGGGTCATTTAAAAATTGATTTTGCCGATCCCTCAAAAACGGAAATAGCACGACAATTTTTCACATATGCTTCGGCGGCTGAAGAAGGTATTCTATTACCCGAATTAGTATTATTAATGAAAAAACTATGGGCTGATGCCGGTGTTCAACAGTCATTTTCACGTTCTCGAGAATATCAATTAAACGATTCAG

>BPA_10229

GCTACCTTGTGGTAAAAGCACAGAACCCGAGGCTAAGGTAATTGGTTTGAGTGAAGGTAAACCCTACAAATTCCGTGTAAGAGCTGTTAATAAGGAAGGCGAATCTGAAGATTTGGAAACAGAGAAACCAATTATAGCCAAAAATCCATTCGACGAGCCTGATAAACCTGGCCGTCCCGAACCAACCAATTGGGACAAGGACTTTGTGGATTTAGCTTGG

>BPA_10235

CTTCGCCTTCCTCCTCATCTTGTGGGGTACCTTCATTGCTGGGTGGTATACCAATTACACTTTCATCATCCGATTCCTTTTGTATAACCTCAAGATGTGAGGTCAGCTTAAGTATATGCAAAGACAAAGGATTCCAACCTTCTGGTACTGGTGGTGGCCGTACCTCAGATAATGGCATCGTTTCTCCAGTTTCTAAATTTTTTACTGGTACTTGTTCCAATATTTCGAAATCTGTTAATTGTTTGCCCGAATTTGTACGTATACGAACATCAATATTCATTTGTTTTAAAACATCTTCATC

>BPA_10245

CAATTATGCCGTCAGACCACAGAGAATCCCAAACGGGATTCTTTAATACGTGGCTGGGAACTAATGGCAATTTGTTTATCATTTGTACCGCCTTCAATAACATTTCAGCCAACTCTATTAAATTATGTCAATCGTCATAGGGACACTACATATGCTATATCATTTCCCGAAGTTGGCAAATGGCCCATACACGTACAAATCTCACATTATG

>BPA_10263

TTCAGATGTTACAACAAAATCCTCGCCAACAGAACATCTTAAACATTTAATAGTACATCAAAATGTCAATATACCTCCCCTGCAACATCCGCAACAACAGATTTTACAAAAATCTAAAAGTGTTGATAATGATTCCGTTACTACTTCGACAAATGTTTTATCCTCCTCGTCTGCCAGTTATATGGCTTCGTTGAATAGTAACAATTTA

>BPA_10269

AGTACAACATATCAAACGCATTATGATGCTGAGGTGGTGGTGGTGCTAAAGGTGTTTTATGATTGTTGCTGCTGTTACTGCTTGAATTTGATTTAATTGAGTTTGTGGCAGAAGAATCGGATGATTTTGTAATGCCACCACCATTGTTGCTGCTATTTGCAGAAGTATGATGTCTAGATTCTCTAGACATTTGCTGTTGTTGATGTTGTTGTTGTTCTTGAGATGTTGCTGACGT

>BPA_10279

ACCAGGAAATGTGGCTGGTACTAGTACAAGAGCTAAATCATTAGCCCATTCATCAGTGGCAGTTAATCCTAGACCTTTATCAGCCGTAGGAGCAGGAGGTTCGGCTGTTATAAATACTGCAGTAAATGCAAATATGCCAATACATGCCGTCACCGCGGGTTCTGTAAATAGTGGTCTCAATACTTTGTCACAGGATCTAACACAATCTTTGCATATATCTTCCACAAACAGTGAAACTGC

>BPA_10294

TGAAAATATGCTTTTATGTGCACCCACTGGTGCTGGTAAAACAAATGTGGCATTGCTTTGTATGATGCGTGAAATAGGCAAACATATTAATGAGGATGGCACTATTAATGCCGATGATTTTAAAATTATTTATGTAGCTCCTATGAAATCATTGGTACAGGAAATGGTGGGAAATTTCGGCAGGAGATTGGCTTGTTATAATCTTACAGTTTCGGAATTAACTGGAGATCATCAGCTAACTAGAGAACAAATAGCC

>BPA_10302

AGAGACTTGCAATATCTCCTGAGATTTTAACATTTTATCAAATTCTGCTTCCCCAGTGAACATGCGAGACATGCCCATCTTTTTCAATGGTTCTGTTAATTCGACTTCAAATTCAGCTTTAAATTTCGGCATATCAACAATTACCTTTGCTGGATACATACGTCGTGTTAAACTGGGTAAAGAGACATTTTTCAAATTTTCTTC

>BPA_10311

CTTGGATCGCGGAAAATAGATTCAGTGGGTCTTCTAGTAGTACGAGGTGTAGTTTTGCCACTTGCAAAAATAACACGATCATTTTCATCAGCTGCCACACGATTATCAAATGAAATGGGATCATTAGGATCTCTAGCTGGTGCCGAGGTGGGTTTGGGACCAGGTACTGGTAAATCTAAACCATTATGTTTTTTGCCGGGTACAGCATGTTTAACATAAACATATTGAACTTGTCCCTGTACTTGTACATCGCC

>BPA_10312

GGCAATGGTAATAAAAAACCAACTAATAACGAGTTGGATGAAAAACCTGTGCCGAATCTTAATGATAAGGAGCTCAACATTAGCAGTGCTAATAGTAGTAGCAGCAGCCCTAGTGCGAGTTTGAGTAATTCCGCCAGCAATTGTTCAGTGTCAAACTTAAGTGTTAGCAGTGGTCAAAATTCAAACAATGTTTTAAATTTTTCCACAACAAAAATCGAACGACACAATGATAC

>BPA_10335

GAAGATATTAAAACTGAAGTAGGAGAAGAGATACCTCAAAATGTCACTAGATCTCGTAATAAGGAACAACTACCACAAAAATATGATGATCCTCTCGAAACCGTTGAGTGTAATGGCAATGATATTTCAATGGCCGAACAGGAAGAAAATGAAACAGATATGAAATTGAAAAATGAAATACAATTTGATGACGCCGAAATGGATAATATTTCAGAATATGAACATTCCGAAGATGACTCAGATTATGATGGTGCATATTTACC

>BPA_10355

CAATACCAGGACCATAAGCACGTACTCTATTGGGATTAGTTTTTGGTCTTAGAGGAGCTCCTTGCTTCAATTTAGCATTGGGATATTGCGACAAATATGTCATCATTGATTGTTCATCTACATTGGGATTAACCAATTCTTCGGGTTTGATCAATTGGCGCACATTCAACCAATCATCAGCAAGACCCATAGCTTCAGAAGCATTTTGTACAGCATCTTTGGGATCCCACATATCCCAATCGGGACAGAGACCAGGGGCACAGGCATCAACCAAAG

>BPA_10356

TTTATAGAAGTAGAAAATTTACCCCGAGCTGCTGTATTGGATTATCTCTTAACCCATCACAAAGCCTTGGTTATACCCTATCTGGAACATGTTATAACGGTTTGGCAGGACACCAACACATTTATCCATAACATACTTGTCAAGCAATATCTCGAGAAATTGGAAAATATAATGAAAGATTTGGAAAAAGAAGGTTCAAATCAGAAGAAGGAA

>BPA_10384

AGATCCCGTGAAAATGCGATCAACTGCCACAACCACACTTTGGCTACTCGTTTGTCCAATGTGTTCCTTAACACTGGGATTTAGAGAATCTTTCAGAGTAGTCTGTGCGCCCGATAGAAATTTCGGACGAACTCCGGTACCAATCAGTTGTGCCAATTCCAACTGACTTATGCATTTAACAATATCCAACCAGCTGGAACCCAAATAATTGCCATCTGTATGAGCTACCATTATAAGAGTTTTAATG

>BPA_10391

ATAGACAGGAAAATAGACCACGATCTGCTGGTACCAATTGATTACCATTCGGACGACCCTTTCCTCTAAATTTGAGAATGCTCTGAACTGTAACTACGCCATTCGATGTTTCGGTAGGTGTAGTACGTATATCATACATTTTACGTCCTGGCAAAATTTCAGTACCATCTTTAATCCATCTTATAATGGGTGCTGGTTTGGCTTTAGCACTACATGTTACTGCGTAGTCTACATCACCAACTCTTCGTGTCATAT

>BPA_10409

CAGACACCAGAAGAGGAAAGTGTCACCATGACCACTTTAATAAGTGAATTCCAGCCAAAAAGTCGCAACAACCCCTTCGATACCAATCGACCACAGAAGCCCGAAACAGAGACCATATCTCCAGAAATCGAACAGCAAAGAGCTACCAGTATTTTAAAGGACAGCAATTATAAACGGCAGGCCTTGGCCCAGCTGGTGGTGGCTTCTATGGAATTGTTAA

>BPA_10415

GTTTATCCGTATGGTACCTATAAGACCTGTAATATTTGCGCCATTAATGGCCAACAACATTTTGGGTTTGTAGATCCAGGCTGTACGATCTTGCCAACGTTTTAGTTCCATATTTAGATCAAAACTACGTTTACCATTGGTATCAAGGAAAACATCAAGTCGGCGATCATCCGGATGTCTATTATAGTTACAAGTAGCCGAAACATTATTATTGGCATTAACAAATGCCACCGAAGCATTA

>BPA_10416

GTGTTACGGCTACGTGTAAATGTACTTGTATTTGAATGTTGTGCCGCTGCAGCTGCCTCCTGTAAAGCCTTCTGTTTAGCACGTCTTGTCTTTTGACGTGTGTGTTCGATTTGAAAATCTTCAACTCGTAAATTTAAACGTTGCATGTGATTAATAATGAAATCTTCATCTAAAACATTATCGGGAGGATTACGTAATACTTCGAGGGTTTCACATAGACCATTACATTGTTTACGTATTTTATGGCTA

>BPA_10428

GGTCAAGGTGTTATTGTGGGTACTGCCTGGTTCAAGGCCGGCATTTAAATTGTGTTGACGCACTTTTTCTAGGTTGTTTGGATGCAACCAAGACTCCCGGCGACTAGCATCCAAAGACGATACTGGCGCATTTGCATTAGACGCAACAACATTATTGCCCGTATTAGGTGACTGAGTACTTAGCGGTTCAGAAGGTATTTCTCCCCTAAACGA

>BPA_10438

ACCCTTGATGATGCATATGCAGTGCCAGCAAACTTTTTAGAAATAGATGTTGTCAATCCCCAAACTACTATGACGGCAGGCAAAAAGAGATACACGGATTATGAAGTGAAAATGCGGACGAATCTTCCAGTATTTAAAGTAAAGGATTCGAGCGTTCGCCGACGCTACAGCGATTTTGAATGGCTAAGAAATGAATTGGAACGTGATAGCAAAATTGTGGTGCCACCATTACCCGGCAAAGCCTGGAAAAGACAAATGCCATTTCGAAACGACGAGGGAATTTTTGA

>BPA_10447

TTTGGAAGACATTAATAATCTTCTAAATTCCGGAGAAGTTCCAAATCTATTTGAAGGTGATGACTATGAAAAGATTATACTCGATGCTCGAGAAGCATGTAATGAAGCATCAAAAGAGGGATGCAATCGTGATGACATCTATAAGTTCTTTATAAATCGTGTGCGCAACAATTTACATGTAGTTTTATCCATGTCACCAGTAGGAGATGCATTTCGTCGTAGATGCCGCATGTTTCCCTCTTTAGTAAATTG

>BPA_10466

TCATCTAATTCCATTATGGATAAAGCAATAGCACGGGCCAAAGCTTCATCTTCTGATATGTTTCCCTGAATAGTTTGTGTTTGACTAGATCCTGCTGTCAGTCGTTGTGTAGCAGCTGCTCTTACAGCAGCGGTAGTTGGTTTAGGTGCCGTATTAAATAAAGTATTTGGTGGTTTTTGTGTACGTCTTTGTTCTGCGGCTTTAGCTGCCATATTCCTTTTATCATCAATATCCGATTTAGTGGTCTTGGTGGTGGTGTTGCCATTACTACT

>BPA_10475

CCACTTTGCCATTTTCAACTTTCTTTAAACGTGCCGGCATAACAACTGGTGTGGTTGGCACTGGATTTGATTGGCTGGTATTATTTTTACGATTATTACGATTCGGTACAGCACAGAAATCTTCATCACAACTATCATCAGAATCCGAACTACCACCAAATATTTCAACTTGTTCTCGAAATGAAACTGAAAGATTTTTGGATTTATTATTGCGTTTGGCCTGTAA

>BPA_10489

GTTAATAGCACCACTGTTCGATAGGACGGCAGCAGCGGCATGGGCTGCACTAACAACAACGCTAGTAGCAGCAGCCGATGTTTGCTGGATGGTGTAGACGGGCACCGAAGTAGGTGAACCTTCGATACCATCCCCATTGGTCGGTTCTGTTTTGATTATCACATGCGGGTGCAACACAGTTGCATTACCATTCACACTTTTATTCAGTTTAACTGTTAGAGG

>BPA_10498

GACGTGGAGTGTGGCGATTGGAAATACTGACAACCCGATGATGACGAATTTGAGGATGTTGAAGACGAGGAGGAGGCGGAGGACGAATTTGAAGAGGATGAAGAGCCATTACTGCTGGGTGAGACTGATTGTTGGCGTTGGTTAATGGTGGTGGTGGTGATAACGGAATGTCCGGGCCAACGTTGAGCCGCCGGGCTAGCGGAGGGCGGTGAGGCCA

>BPA_10501

AAAATCATCACGTTCAATTTTTGGTTTTTCACCTGGTTGTGGCTTTTTGGCAGCCGGATAATGTGACAATTCTATAGGTTCTTCGTTGTTCATGGCAGGACTTTTAGGTCTGGGCGTTTCTGTACGTATTGCATCTACTAACACTTTCATGGCACTACGTGTGCGTGAGTTAGGACGCGATGGTGGTCGGCTGCCAGCCACCGAACCGGCATT

>BPA_10528

CCCCGCTCAATGGCGGACATGGCAAGTAGTATTAGCGGTGGAGGTGGCGGTAGCGGTAGCGGTAGCGTTGGTATTGGCATGAGTCACGGCAGTTTCGGTAGTAGTTTCACAAGAAGTGCAGGAATTTCTAGTTCATATTTGCGAGAACGTAGAGAAACATTGCCCGGTTGCAGTTCTCACAATAATAACAATCGACGGAACAATAATAATGTTGTTATTGTGGCAG

>BPA_10536

GTTTGGACGATTTTCATTAGAGGTCCATTCATTATTACTAGCATCACTATATGGATTTTCAAAAAATGGTTCTCTCTCTAGTGCTTCCTCCTCAGATATTTCTGTTAAGGGTTCCTCTTCAAATTCTGGCCAATCTTTTTCAGTCGCTTCATCTTCCTCCATTTCATCAAAATTTATTTCTTCAATTTCAATTTCATACTTAGTTATTTC

>BPA_10546

TTCATACGTTATTTACTAGAACGCGATTTTCCTGGTATTCGTATTGGTCCTGAGCCAACTACTGATCGTTTTATTGCTGTTATGTATGATGACAAGGAGGGTGTTATTCCTGGTAATGCTTTAGTAGTTGATCCTAAAAAACAGTTCCGTCCCTTGAGTAAATATGGCAATGCTTTCCTTAATCGTTTCCAATGTTCCTCTGTTAACTCTCCTGTGCTCCAGGCGATTTCTATTGTGGACACACCTGGTATTTTATCAGGTGAAAAGCAACGCATCGATCGTGGTTA

>BPA_10566

AAATGTTTCCACAAGCTATGGATAATATAAATAATGATTTGGAGAGAATGTTGCCGGCAGATGTAAGAGATGCGGATAAAGAAATTTCCTTACTAAAAGCTAAACGCATAATGAGAGCACATTTTGGTGATAAAACACCCAGTAGTGAGTGCATGCAAGAATTTTTGGATTTATATGGCTTTAAAGCTTTTTGGCATGGTTTTCATCGTTCATTACTCTCGCGCCTAACATATGCCAAAGCTCCCACTTATCTTTATCGTTTTG

>BPA_10570

AATGATAGTCAGACACCCTCAACGACAGCTGGTAGAAAATCGCGAAGAGGAACTACTGCAGCTGTCACATCTGCATCAGCCCGTAAATCAAACGCAGCTTCTGCTACGCCTTCAACGTCAGCTGGTAACAAACGTAAACGTGGTGCTAGTTCGACAGCTTCAACATCATCAAGTCCTCGCAAAACGCTGACAAAAAAACGAAAAACATTAGTTAAACGTAAAAAGAAGTTGG

>BPA_10571

TAGCATCAACAGAATTATTATTGGCAATAATTGCAATGGGAGTGGAAGTGGGTGGTTGAGCAGCAACATCGTTTATTTCTACAGAGTTCTGTTTCTCTTCATTCTTTTCCACATTATTTAACTCACAAACTTGTGCTGGTTGTGCTGGGGTTTTTGCTGTTGAGGTTTCTTGTGTTACTGCAGTTGTTTCCTCAGCCACCACATCTTCTTCCATTTTCTCTACGGCATCATTACTTTTTGAGCTGGAATCTACCTCCATAG

>BPA_10591

AGCATTTGCAGAACTTTGTTGAGAATTTTGACCAGGTGCAGAACCAGCTGATGCATAATCTGTCCATTCTGTAGTTCCCGCATTACCGGGTGCCTTATGAGCCGGCGATGTGCCAGGACTTTGACGTACTGAACCAGCAGCTGATGGTGGTGGTGCTATTTTACCCATACCCCCTGGTGGTGGTGGCAATATGCCAGTACCAACCTTTGATTTTGTACGACCAGTACCCTCAGAGCCATCTTTTTTCGTTATTCTCATATTAATTTTAATAGTTTCACCCTCTTTGAAACCTAAATCCAATTCTTGTTTTGGTTGAGTTTTTTCCTTTTCAATTTGTTCCTGATTTTTAACCCATTTAAAATGATCTTGTAATGCTACATTCAAATCAAAAGAATCTGAACGATCACCAAAACCTAAACCCAAAAAGGCAGAGCGACCATTATCATCTTGCACTCGTATAACAAAATAACGCGAACTATCTGATACTGCTTCAATGGCTACGCCAGGATATGTTTCAATGGGACAATTAGCAAATAGAGCACCAGTCGTTTTATCTTCTAATTTAAGATTACAT

>BPA_10593

ATAAAAAGCATCTGACGTAGTGGTGTTTTTTGATCATCAAATTGTTTCGTAACTGTCTGTACTAATGCACGCCTTTGGGGTTTTGTTGTTCGTAGCAATGTATATAAAAAATCGTTCAGTGCTGTTGGCATCGTATCAGAATCACGTTTTACATAACCACGAATTATATTACATTTTGGCTTATTTTTCCACTGTAATACCTCTTGCAACTTGAAACATAATTGCAATCCAAACTGAACTTTCATATTTACAAATCCAGAGTA

>BPA_10595

CTGCAATTCAGCTGATTCGTGGGTATAAATAACAGCATTTTGGTTACTAACACTGGCTCCGGGTTTGCTAGTAACAAACCAACGTTTTGTGGCACTTAATAATGACTTACTAACACCTTTTTTATTTGTGACTGCTTCATTTAATATGCCCACCAAATGTTCAACATATGGTATTAAAGCTCTAACAGCATAATCTTGTACAAAATG

>BPA_10598

GTATACCATAAATATGTTATAATATTAATATAAGTGGCAAGACTATGTTGTTTACTGTTGATAGTCATTAATCCTAGATTTTGTCCGGAAAATGGATATTTTACCGTATTACGACATTTATCATGAGCAAAATTGCAATAGGAAGTATGAAGAAATGCTGTTTTCTCTAATTCTTTATCCCACGTCATAACTGCATGACGACAACC

>BPA_10608

CTGAAGTGCAGAAATGTTGATGAGCATAAAGTGTCTTCATGCATCGCTTATTACGTAAATCCCAAACTCGAATAGTTTTATCATCACTGGCTGATATCAAATATTTACCACCAGGATGGAATGCTAGTTCTCGCACCCAATTGTCGTGGCCGGACAGCACAAATAAGCATAAACCAACACTAACGTCCCAGACGCGTATAGTTTTATCTCTAGATCCAGATGCTAAAAATGGTCCTTGATGATGAC

>BPA_10616

CAAAAAAATAATACTGCTGCACGACGATTGCATATAAGAGGAACCAATGGCAAAATTTATCCCTACTTGGTAGTAATCGATACTGGATTGGGTGACGCTAGAAGAGAGGAGCGAGTTTTACAACTTCTACGCATGCTGAACTATTATTTGGAAAAACAAAAGGAAACAACTAGACGTTTTCTAAACATTACCGTACCACGTGTTGTACCCATATCTCC

>BPA_10617

CACCAAAGCTGTTAAGGCTACCAAAACTGAAAAGGCTGCCGATGTTAAAACTGCACCCAAATACAAACGTCATGGACGTTTGTTTGCCAAGGCTGTCTTCACTGGCTACAAACGTGGTTTGAGAAATCAACACGAAAACCAAGCAATTTTGAAGATTGAGGGTGCCCGCAGAAAGGAACATGGTCAATTCTACATTGGCAAACGTTGTGTTTATGTGTACAAAGCTGAAACTAAAAAGTGTGTACCACAACATCCAGAACGCAAGACCCGCGTCCGTGCCGTCTGGGGTAAAGTAACTCGTTTGCATGGTAACAGCGGTTGTGTACGTGCTCGTTTCAACAGAAATTTACCTGGTCACGCTATGGGACACCGTATTCGCATTATGTTGTACCCCTCCAGAATTTAAGTTGTTTTAGAACTTA

>BPA_10625

TCTTCCTAATACTTCGTTGTCGTCATCGTCTACTGGTCCTAAGACCGTTACTTCTTCGTTGATTTTAAGTAATTTACCCACTACCACATCGACTCAATCGATTTTACAACAACATCCAACTAATTTGAATGTTGCTACGCGTGCTCCACAATCTGTAACAGCATCGGTTTTCTCACCATTACCGCCATCTACGGCAGCAGCAGCAGCAACAACAACAAAATTACCTTCAACTACGGCTACAGCTGCTATTTTA

>BPA_10638

TCCAATATATGTGGAAGTGGATCCTGTTCTACGATAGCTTTTTTTACATCCAATGATGCTCTGGCAGTTTTAATATTATGGGCCATCTGGATGTCGGGACAAGCAGCTATGTTGTTGAAATATGATGAGTCATTTCATCAGCTTTGCGAATTACTTAGAAGCAAAAAATCAAGGCTTATAATAACACCCCATAAATATGAGAACTTGAGTAAACAATTGGCTCTGAA

>BPA_10657

CTTCCCATGAGAATTATAAAGAAAATTACGAAAATGATAGATTTTTTGAAATAAAACCCTTAAGATTACGTTGTAATATGCCAGCACCAAAATATCGAGAACCTACCTTAACACAACATCCACGTAAACCTAGAGATTATCAGCAATTGAGAGTACCTTCAGCCGTGTTTCAGCAAATAGTGGCCACTAGCAATTTACCATTTTTGCATAAATTAGATGAAGATAAAATAGAATTCGCTAA

>BPA_10670

TTTTGCCATTATCACATTCATCCACTTCCATTTTCTCGACTTTATGGTGCTTTCTTTTATATTCTCTCTCTAATCTAGCAGTCTCCTCCTCATATTCACTACTTGATCGTCCCAAAAGTGCTAATATTAAAATTATAGCAGTTGTGGGTTTTCCTTGATACCTTCTCACCATTGGTTGTTGTACCTCACTCAATTCCTCTTCGCGATCAAAT

>BPA_10694

ATTGGCGGCTGAATTGCCAAAACCAAAACGATTGTAATAGTTATTTTGTGCCGCTGCATTAGAGACAGCTTGAGATTGACCAAACTGACGTTTAACACGCTTATGTTCCACCAATGTTTTAAGATTGAAATTATTTAAATCATTTAAAATGGATCTACGAACTCTACGCAATTGAGGGCGTTGTTGTTTGGGTTTAATATACAAAGATGATTCAACCACAACTTCATCGACTGCTACACCACTGGAGGGCAGTTGAACATTTTGTTGAGTGTTTTCCTGATTGCCCACATTTAAATCGGCTTCGCATAAATTATTAGCTGCAGCGGCACTCATGCAGTCATACTTACACACGCACGCACGCACGCTTCTTAGTAC

>BPA_10706

GCGGTTGTCATAATGGCATTATTAAACTTTGGGATCTAAGATGTTCGAGTAGTAGTAGTTCACCACGTTTTACCAAAGCCAGTAGTGTCTTTCTGGGTCACTTGGATAGTGTCACCTATATTGATCCAAGAAATGATGGTCATTATATTCTAAGTAACTCCAAAGATCAAAGTATTAAAATTTGGGATTTAAGGCAACCAACACCAAAAAATAAAGTACGAACTAATCCAAATACTCCCTTAATTGATTGGGATTATCGTGTCAATCAAGTACCTAGAGAATATTATAATCCCACCAAAGTGTTAGATATGGTGATGTTAGTGTAATGACCTATCGTGGTCATCGTGTCACAAAAACATTATTACGTGCAAAATTTTCACCAG

>BPA_10708

GGCGATCGCATTGAAGTTTGGACTCCAACTAACTGCATTTACTGCCGAGATATGTCCCTTTAATTCAGCCTCTAATTTACCCATAATATTCCAAACATAAATGGCACCATCTGCTGAACCACAAGCAATTTTTGTGGCATTCATATTAAAAGAGGCACGAGCCCAATCACAGCTAAGTTTAAAGTGTTCGTCAGAAAATGTGGTTATAATTTGATTTTTACGTAAATCTAAAAGTTTTATAGTATCATCACGTGTTGAACAAATGAGGTAATTGCCATCTTTTGACAAATCCAATGAGGTAATTTTAGCATTCAATAAAACATCGTCTGCTTGCTTTTCTGTACGTATATCCCAGAAACGTATCTTTTTATCGTAATGACCACTAATGATGGTTGAACCTAAACTATCGGTGGTAACGAGATCATTACAACTGGAACCAGCAAATTTTGTTTCTATACATGCTATGCTACGTAAGTCCCAGATTTTTAAGGTTCTATCATAACTGCCGGTAACAACTTTAACCGGTTCTTGGACGTATTTAGCAGCCATAACTTTACCACTATGACCTGTTAGGGTGTGCCTTAATCTATTATCAGCAGTTGACCAAATACGAGCGCCATAATCATTTGATGTGCCAATTATAAAGGCACCATTGGAATCAAAATCAACGGAATTAATACCAGCACTGCTGCCACCTAGAACAGCTCGAGGCTCTGTAGAACCTTTCCCCACATCCCACAATTTCACTCTACGATCAGCACCACCTGTTGCTATGACACGTTCTATAGGACTCCAACGTACAGCATGTGACTCTGTTTCATGAGCTTCAAATTTCATATGAACTGTTGTGGGGCAGGCAGTTTTATAATAGCCACCACCATAACAACCAACATTACCCATACCACCATATTGATCACTTCGTGTCTCACCATACATTTCGGCCTCACTACCAGCCTCTACACAATGATCACCACTGCTAGTATCACGATCGTCGTCGTCGTATGGATCACCACCATATTGATCACTTCGTGTCTCACCATACATTTCGGCCTCACTACCAGCCTCTACACAATGATCACCACTGCTAGTATCACGATCGTCGTCGTCGTATGGATCACCACCATATTGATCACTTCGTGTCTCAC

>BPA_10713

ATTTTCTTTCGACAAGAACAATATTTTTTAGCCCCACCATCGGATACACATTCGGAATCGGCACTATATGTATTAAGACGGCTTATTTCTCCCGTTACTTTCAGATAATTCTCCAAAGGATCATATATGGCAGTTGCCTCAAAGAGTCCTTTATTTGGTTTTGTGTAGAAAGTTATGCGATAAATATTTATAGTTTTGATACTTTTGCCGTCATCGCTTACTTGCAGTTGGGTGGTGCGATAGGCACTTTCCACACTATCGTATGATAAAGGAACACATAGTTTGGCCAAAGAACGATTATGAAATGTGGCCACATAATCATTAATATAATCAATTAATAAATGTGTTAAATTGACAACGGTTTTTGAATTTTTATAAATACTTTCGTACAACAAACAAGTGCACCAATGTGGTTCAATCGCTGCATCCAGGCAGGAACGATTTTGTGGTACTGTTTCTAATAACGACTGGCACTTGGGACAAGCATCTGCTCCATTTAAAACTGTTGCATTTTGTACTCTTCCTGTTAATTTTAAAATATGTTTAAGTGTCATGTGCGTGTCATAGGGATTTGTCAAACGATTCTTATTGACATTTAAGGCATTCACAAAGCTGGGATAATTTTGTTTAAGATATGGTGGTAACCACAGGAAAACAAAGGGTAATCTCTCTTCCAGATGACCCGAATAGGTTTTTCGTATAGGTCCGAAGCGCATGCCATGATCGCTAAAAAATATCACTATTGTATTATCTAAAGTACCCATTTCCTTAAATTTCTTCAGATAATCCATAATATGTGTATCCATGGCCGAACAGTCACTTATGTCATTGTGACTAAACGTATTTGCCCAAAAAAGACCAAAATATGAATCATTCTTATAACGCTTTGCCAGTTCGATGGCGTAATCGAAAACAAAATCAGCTGAATGTTTATAACCCAGACAGTCGATTAAGCTTGATTTTAATTTAGTTTCCAAATGCTTCTCGGCTCCAATTAGAAATGGTCGTAAATAATAATCTACGGGTGGTTGTTGAAAACCGACTTTTAAGTAATTAAAAGTATTTATGCTGGCTTCATCTTCGGCATAGGCCGTTACATAACCATGTTCGCGAAATGTTTTCCAAATAAAATCACAATTTAATGGCCGTACTTACGGGTATGAATCGCACAACTGTTATGCAAAAATGTTCTCCGTAC

>BPA_10715

AATTTACGCACTTCAAAAGGTAAATTCTTTTTAGCTGCATTTAGGATTTCTACAGTCTCTGCCTCAATTTCAATATGTCCGGTTTTCATTTTAGGATTGATGGTTGCTGCTGGTCTGGGTATAACTTTACCCTCTACTCTTACTATAGATTCCAAGGTGATACCTTGATCTTGTTTCTCTAGACCAGTACTGTCTGTTATGAGAACTTGGGTTTGTCCATAACCATCACGAAGTATGAAAAATTTACCCATACGTTGAAATTCTAACCAACCACATATTGTGACTTTTTCGCCAATATTATCGGTAGTCAACTCGCCACAAGTATGGGTACGATTCGCAAATTTGTTTAAATCAGCCACTTTAATAACATTGCCAGTTCCATCGGATTTTGATGTGCCATCTTCTTCCATGGCTTCACCATTTTTACCTTCACCTTCGCCACTCTCATCAATGTCGTTAATGGAAAGTTTCTTGACCTTCTCCTTAGCCTTAATGGGACCATCATAAGGATCTTCCGGATTGAGTACTTTAACTGTATCAACCATAACCTCAATTTCACCTGTATCGTATTTCATGTTGATATTTTGTCTTGGTCTTCCTAAAACTTTTCCAGTTATCTGTATAACAGTTCCCTCAGGAGCACTAACAACAGTATCTTGCATAGTTTGATCTTCAATAATAATTTGC

>BPA_10723

ATTGCCAAACCATCAGTATTCGGTACATTTGGTATGACGAATACGGCATCAAATAATCAACACCAAAATTCTATTTTTGGTGCCCCCACTGTAGGAGCAGCATCATCTGGATCATCATCTTTAGCTAATTCAGCCCCAGGCTCGACAAATACTAGTGCAAATCTGTTTGGAACCAATTCAACGCCTGCTGCCACAAGTATATTCGGAGCTAGTTCAAGTGGAAATGCAGCAACACCCACACCTGCTTTTGGTTCATCACCATTTGGTGGAGCTGCTTCATCGACTAGTGCCACACCAACTTTTGGAAGTACTGCAGCTTCAGGAACCACAACTTTTAGTGGTTTTGGTGCTACAGCCAATAATACTATAACAAACACAACAGAGGTGAAAAAACCTGAAGCGGCTTTCAATTTTGGTGCTGCGCCAGTTACACAGAGCACTAGTGGAGGTTTTAATTTTGGATCATCATCTGCAACAAAGCCAGCTTTCAATTTCAGTGGTACAACAACAACTCAACAACCTTCTTTCAATTTTACTGGTACTGCGGAGGGCACGCCGAATGCACCTTTCCAATTTAATGCAGGTACCGCGCCTGTATCAAGTAATATATTCGCGCCAACACCAACACCAGGAGCCCAAACAGCACAACAGGCAAAGAGGAAAATACGTGCACCAATACGACGAGTAACACAGCGGTAGAGAACATTATAAAAACAAACGCTTTCATCAGCGGGGACCGATCACAACAAAATATACATGAACAGACAGGACAGAATATAAATAGCAGCTGTATTATTAAGAAAGAGAAAAAACATCAATATCAACATTTATCACAACCGCAAAAACAAGAACAAAAACAAAACCAACAAAAAGACTACATCATCTTCAGTAAGACAGACGACAACTTACGCCGCGTTAGAAACTGCACCGGCATTAATACTGTAGATGTTGCCAGCAACAATAGACTTTCGAAAGATTTGCAACAAGAGNNNNNNNNNNNNNAAAACAAAAGAATAAAATTCTTTTAAACAAATACAAACTGATTAGGAAAGTATCATCATTATCAACGGCGTCGTCATTATCATCTTCAACAAATAGATATAATGTTGATGATATTATT

>BPA_10724

TAATTTTGTCCTTGGTGAAGATACTATTAATGAAGTTGAAGCACCATGTGTTTCTTGTTTGTTTGGAATGGCAGAAGTGGAAGCTTGGTTTTCTAAAGATATTGTTGCTTCTAGGTCTATTGGTGTTTTGGATATTACTTCTGCCGAAGTCTTTAATTGTCATTTAACTAGTTCAACTGTCAAAAACCTTTTAAGCCGTTCAAAGTATTGTGAATGTAGTTCCACATCATTATGTCCAGCCCCCTCTACCCACAATGGTTCCACTGCCTTTGGACATCTTTCGTAAATACCTATACCATGGGAAAAGTCTATTACCTCATCTTCCGTACCATGAATGACCAATACAGGTGATTTTACTTTTGAAACTTTATCAATGCTTGGAAAAGCATCGAAGANNNNNNNNNTTTTTGTATTCCTAAAAACAACTCTTAAACCAGACATTAATGGCGAATGTAAAATAACAGCTCCTACTTCGTGTCTAGTTGCTAGATCTACGGTAGGAACAGTACCAATACTTTGTCCATAAAGAATTATAGTTTCCGGATTAATATTTAATCTTGTTCGCATTGCTTGCCAAGCTGCTTCAATATCAGCATAAAGATTTTTTTCTGATGGCTTCCCTCCACTCATTCCATAACCAGAGTAATCATAACCAAAT

>BPA_10733

AAAAGCTTAAAACACAAGTTCGAAGATTGCATCAAGAAAATGCTTGGCTAAGAGATGAATTGGCTAATACACAGCAAAAATTTCAAGCTTCCGAACAATTAGTTGCGCAGTTAGAAGAAGAAAAAAAACATTTAGAATTTATGACATCTGTCAAAAAGTATGATGAAAATCAGGACCAAGATGATGTTAGTGAAAAGCCAAGACCAGATCCCGTTGTTGAATTATTTCCAGATGAGGAAAATGACGATAGAAATAATATATCTCCAACTCCTCCAAGTCAGTTTTCGAACCAGTCATCAGGATATGAGATTCCAGCACGATTGAGAACCCTTCATAACTTGGTTATTCAATATGCATCACAAGGAAGATATGAAGTTGCAGTACCCCTTTGTAAACAAGCTCTGGAAGATCTAGAAAAAACCAGTGGACACGATCACCCTGATGTCGCTACAATGCTAAATATTTTAGCATTAGTTTATCGTGACCAGAACAAGTACAAGGAAG

>BPA_10734

CGTTCATCCATGGGCGGATATTGACGACCCTTTGATTTGCCCAAACATTTATTGCGTTTCTCACTCACCACCTGACAAAAGAAACCTTTTTTCACATCGTAACGCAATTGATTTGAGTAATCAAAGGCCGGTTGTATTTTCAAAAAACGTTGCAAATCATTCATCACATCAACTGGATTGAGACGCAATTGTTCACCATCAATGATGTGCAATTGTTGGGCCGAATAGGAGAGCCAACGTTCCAAGTGTTGTGCATATTTACCAGGATTAAGGCAACGATTACGTAAATCCCTGAGTGCTTTTGGTGCCGAATCAGAAGCCGTTATGACCTGATAAAAACTATAATTATTAGCAATAGTATCACCATGAGCCCTTTGATGCTGATACCAGGAGTAAGCTCTCTTCGCAGGTGATATTAAAATCGTAACAATTTTTGAATGTGGTAGTAAAGCATGAGCTCTCTTCGGCACCATTTCGCCATCAAAATAGGTGGCACTCTTTTCGAAGATATAACGTGGCGTGGAATTGGGTGCTACTGTCGT

>BPA_10735

CCTAGTGGTGGTGGCGGTAAAGGTTTAAAAGGCATTATTGCTGGTGGTATTACGGGCGGAATTGAAATTTGCATCACTTACCCCACGGAATATGTCAAAACACAATTACAATTGGATGAGAAAGGTGCCAATAAACAATACAACGGAATTGCTGATTGTGTAAAAAAGACTATTAAACAGAGAGGATTTTTTGGTTTATACAGAGGTTTGAGTGTTTTGTTGTACGGAAGTATACCAAAATCTGCAGCGAGATTCGGTGCATTTGAAACTCTACGTGGTCATTTGGTTGATGACAATGGTCAGTTGAGTAATTCTGGAAAATTGTTAGCTGGTTTGGGGGCTGGTGTCTGTGAAGCTGTATTAGCAGTAACTCCCATGGAGACCATTAAGGTTAAATTCATTAACGATCAACGTAGTGCTAAACCCCAGTACAAAGGTTTTGCTCACGGTGTTGGATGTATTATAAAATCGGAAGGTATTAGTGGCATATACAAGGGTTTAACACCTACTATCTTAAAACAAGGTTCCAATCAAGCAATTCGCTTCTTTGTAATGGAATCTCTTAAACTTAAAGATCTATATAAAGGAGACGATAAAAATAAACAAGTCCCTAAATTATTGGTTGGTGCATTCGGT

>BPA_10739

TAGTCTTTGCATGAAGAAGTGTCCATAGGACTGGCCGGTTGGGCAAAAGTATTTGTGGCTCCACCACTTGGATTTGTTGGGGTAAAAGCGGAATTTGTGCCACTCACATTCATGTTTGAATTGGACGTATTCTGTAAATTGAAACTTAAGGGATTTGTGGACTGAGCGGAAGAACTTGTAGGTGGTAGAACTGGTGGTGCAGCATTATAACCTAACAGTGTTGCTGAATTAAAGGATAAACGTTCATTACCCAAACTATTAAGAAAACTAGCAGATGGCCCCGTAGCATTTGAGGAACTTGAGAATAAGCCGCCTACAACTGTGGGGCCAGCTCCAGTCGAACCAGACAAAACATTCGGAGCTGAAGTGTTAAGTCCCAACATGGGTGTAGGCAGTGCTGGTTTAATAAAACGTTCACTAGCACGACGATTCACAACTTGTCCACAAGCATAACAACGATTTTGTTGTGAAGTGCCTGGACTTAAAGGGCGGCTATTTGATGGACTGTGTGATACTGGACTACTAATGGTACGTTGACGATGAGCATTTAATGCAGATCCAGTCAAAGGCGCACCACTTGCCAATATATTCTCATTATAAAAACGTTCCTCATCCATTTCCAATGATGATTCCGATTCGGACAAATGGCGACATAACGCTTCCCTTCTTTCTACTTCTTGCTTTAATTTGCGTTGTAGCCTTGCATTCTCTTCACGTATAGACTTTTCTTCTTGGGCAAATTGTTGCAATTTCTTAGTGGTTTCTTTTTGGGCAGCTGCTAAATTAGCTCTCAGACGTATAACTTCAGATCTTAATGTTTGTATGTGTGAACTTAAATTGGAGGCAGTGTCTCCGTTAGCATTAGTAATATCACGAGGTGTTGTTGGATCCGATACCGGTTGATCAAGTTTTATTTGTAACAAACGTTTTTCAGTTTCTAATTTATCCATACGTTTCCATAATTTATTTACTAATGCCTCCTGCTCCTGTTCCAATGTATTCTCTAGTTCAACCATTTCTCTACGCAACTGTTCAAGATTCGTTTGCTTGTTATCAGTTTCAGCTTGTAGTTTTTCAATTTTTCTCATCAATTTATTTACTAAACATTCTTGCTCCTGTTCAAGTGTTTGTTCTAGTTTACATTTCTCTTGCCGAAGCTGATCCAATTTTCTAGATAAATCATTAGTTAAACATTCTTCCTCTCTTTCATAGTGATGTGCTAGAGTTTCCTTTTCTTTTTTAAGAGCTTGAATTTTTTTCAAAAGAGTATTAGAAATGTATTCCTCCTCCTGTTCGGCTTTAGCTTGTATAATAACCGAAGCTTGTTTAAGAGTTCTATTTTCTTCTTGGACAACTTTGCATTTTATTTTCATGGTATCCAATTCCGCTTTAAGAACTTTATTCTGTTGGGTTAATGATTCTATGCGTTTT

>BPA_10742

TTTTTCTCCGTGGTTTTTGCGATTAAACATGGCACCAACCCCTAAAAACCAAAAGGGTGACAAACCCGCTGCCGCCAAAAAGGCCGAACCTGCTAAGGCTGCTCCAGCTGCCGCTAAGGGCAAAGTTGAAAAGCCTAAAGTTGAAGCTGCCAAACCTGCTGCTGCTGCTAAAAATGTAAAGAAGGTCCCAGAAGCCGCTAAGGATGTTAAGGCTACCGCTGCCAAGGCTGCTCCAGTAAAGAAAGACGCTAAGGCTGCTGCTCCCGCTAAAAAGGATGCTAAAGCCGCCGCTCCAGCCAAGGAAGCCCCCAAAAAAGATGCTAAGGCAGCTGCTAAGCCAGCTGCTGCCGCTGCTCCTGCCAAGAAGGCCGAACCAGCCAAGACTGAAAAGCCCGCTGCTGAAAAGACTGCTAAAGCTGTCGTTAGCAAACCCGCTGCTCCCAAGCCCAAGAAGGCTGCCGTTGCTGCTGGTAAAGTAGGTAAGAAGGCTGTTTTGCGTGGCAAGGGTCTTAAAAAGAAGAAGGTGTCTTTGCGTTATGCCATCGACTGCACCAACATTGCTGAAGACAATATTTTGGATGTTGTCGACTTCGAAAAATACATCAAGGCTCGCATGAAGGTTAACGGTAAAGTCAACAACTTGGGTAACAATGTCACCTTCGAACGTG

>BPA_10748

GTAAAACTTCGTTAATTCTTTCCCTGGTAAGTGAAGAATTTCCGGAAGATGTTCCCCCTAAAGCAGAAGAGATAACTATACCAGCTAATGTTACACCGGAACAAGTACCCACAAATATTGTGGATTACTCAAGCGTTGAACAATCGGAGGAAATTTTAAATGAAGAAATTGTTAAATCTCATGTTGTATGTATTGTTTATGCTGTTGACGATGATGATACCTTGGATCGTATTACATCGCATTGGTTACCATTGATACGTACGGCAGTGGGTGAAGATCAACCTAGAAAACCGGTTGTATTGGTGGGCAATAAAGTTGATTTGATTGAATACTCAACTATTAATAGTGTTCTTTGCATTATGGAGGAATTTCCTGAAGTTGAAAGTTGTGTTGAATGTTCTGCCAAGACTTTGCACAATATATCCGAAATGTTTTATTACGCACAAAAGGCTGTATTACATCCCACATCACCACTTTATATAATGGAAGAACAAGATCTAACACCTGCTTGTAAAAAATCTCTGGTGCGAATTTTTAAAATCTGCGATATTGATGGCGATAATCTTCTCAACGACTACGAACTAAACTTATTCCAAAGACGTTGTTTCAACACTCCTTTACAGCCACAAATATTAGATGAAGTCAAGTCGGTAATACAAAAGAATGTAGCCGATGGCATTTACAAAGATTCCGCCTCAAGGGTTTCTTATTTCTTCATTGCCTTTTCATACAAAGGGGACGTAATGAGACCACTTGGGCGGTGCTAAGACGTTTCGGTTACAATGAACATTTGGAAATGTGTAAAGATTATTTGAAGCCAACTTTAAAAATACCACCTG

>BPA_10752

GAATATTCGATATTAGATTGTAAAACATCTCTGTTATCAAAAGGTATACGGAATAATAGCATAACATCATCTGCAAAAAAAGTATTATGACCACGTTGAAATGAGTGTTGGGAAGAAGTAATATAGTCCAAACAAAATGAAGCATTTGATCTTTCGTCAAATGTTCAACAACCTCTGACATGAAAGGAAGTATGGATATTGGTTTTAAAAAGATGACGAGGTTAATATAAAGTTAACTTGATGCTTAAGACAATGTGATATATGAGGAAAAATAATTATAAAATGTATTGGAATAAAAAATCTTTCACATGCCCGATATATCCAACCCAGAAAATAATATCGAATAAAGCACAAATAATGTAATTATTCTTTGCCTTATTTCTATATCTGCAATCGATAAGCCAACTTTCAGAAGAATCGCTAGATCGGAAAACTAAATTAGCCAAATCCCTGAGAGAATAAACATATACAACTGGAC

>BPA_10753

AACTTGCACAGCACAATCCCAGGTACGTGAGAACATTTGCAATTGAATGAAGGACATAAAAGGTTTGGTACGACCACCTTCTTCTTTTGCCAAAATATAAACTTGAGCTTCCAACTGATCAAGAGCTTTAACACTGCCTGGTTTGCACATAACCATACCACGCTTGATTTCATCACGTTTTACACCTCTAACTAAAGCACCCAATTGATCACCAGCCTGAGCTTCATCCAAAATTTGATGGAACATTTCAACGCCAGTAACGGTGGATTTCAATACTTTGTTGTAGCCCACAAATTCACATTCCATACCCTTCTTGACAACACCTCTCTCCAAACGACCAGTGACTACAGTACCACGACCAGGAATGGAGTAAACATTTTCTACAGGCAACAAGAAGGGTTTATCCAATTCACGAACTGGTGTGGGAATAAAGTTATCGACTTCGTGCAATAATTTCAATATGGCTTCAGAACCAATTTCGGGCCTCTTGTCTTCTAAAGCACACAAAGCTGAACCCTTGACAACGGGAATATTATCACCATCATAACCCATTTCCGTCAACAATTCACGGATTTCCATTTCTACTAAATCTACCATTTCTTGATCGGCAGCATCGACTTTGTTAATGAACACTACAATATGA

>BPA_10759

AGCGTTTTCCAATGAATTTTGATCTATTGATGTTAGATGATCCGAGGCTGGACGTTTTTCAGCATTTGATTTTATTGTCGATGGACCACTGCTGGTTGTTGGTTTAAAGGCGTTTGTTTTCTTAGCAGGACCCAAAGTTGTTATCAAAGAATACATATCGTAGGGTATATATATTCTTGTATTCGGTAGAAAACCTTCAATATCGGGAGGAGGTTTTTGGTAATACAATGGTGGTAATTTAATGGGTACATTGAATGTTTTGTTTGGCGTTAAATCACATTGTCCCATTTTGGAACAAATTATATAATTAGCGAGATCACAACAAGCCCACATTTTCAAATTATTAATTTGAGGATCTTCATGCGGCGTTACCACATAATGATCCTTCATTAATTCAATTAAGTTCTTATAGAAACTATATGAAAAGGCCTCCGATTTAGCCATTAAAGGTTCTAATATGAAGCGTAAACATTTCTCAACTTGTTTTAATTGAGTACGATCCTCGTGATCGGTGAATTTTGGATCGTGTACTAAAATGGGTATAGCAAAAGCTAATATAAAATCAGGTAGTAGGTGTAAACGTTGTTCGGCAGAAATATCAGGATTTGTAGATTGTATTGTTTTCAGATATTCTCGTCTTCGATTGATATCTGCTTCTAAAAATTGACGTTTCAAGTCTAATAATCTTTTATCTGTTTCATTACCCGCAAAAGCGTAAAAACCCATAAAATCCAAGGGTAAACAATTA

>BPA_10770

CCTGGAAGAAAATTAATGCAAAATTATCGAAATTATGGAATTATGAGAAATACGGTTGTCCCATAAAACATGGAGACTATTATTATTTCTACAAGAATACCGGCTTACAAAATCAAAGTGTGTTATACCAGCAGGATAAGTTAGACGGAGAGCCTCGATTGTTTTTCGATCCTAATGCGCTATCCACCGATGGCACAATTGCATTAGCTCAGAAATCCTTTTCAGAAGATGGCAAATATATGGCATATGGTTTGAGTGAAAGTGGTTCCGATTGGGTAAAAATCTATGTGCGTGATGTAGAAACTGGCAAAGATTTGGACGAGGTTTTAGAAAAAGTTAAATTTTCTGATATATCATGGACTAAAGATAACAAGGGTTTCTTCTATGGTCGTTATCCCGGGCAAGAAGGAAAAACTGATGGTTCGGAGACTAAACAAAATGAATTTCAAAAGCTTTACTACCACTATGTGGGTCAACCTCAGGAAAAAGACATTCTTATAGCCGAATTTCCCGAAGAGCCAACTTGGCGTATACAATCAGAGGTATCGGACTGTGGCAAATATCTGATAATGCCCATAGTAAAGGACTGTCGAGATAACATTGTTTACTATGCAAATCTAGAAGAAGCCGGTGAAATAACAGGCAAGTTAAAGGTAAACAAGATTGTGGAAAAATTTGAGTCCGATTACTATTATGTAACAAATGTTGGCTCAAAAGTATTCTTTAGAACAAATAAGAATGCTCCAAACTATCGTGTTATTGCCATTGACTTTGATAACTATGCCGAAGAAAACTGGGAAACCTTGGTGCCAGAGCATGAAACTGATGTACTCGATTGGGCCCGTTGTGTTGATGATGACAAACTTGTGCTCTGTTATATACAAGATGTAAAGAGTGCCCTGCAAGTGAATTCATTAAAGGATGGTAAATTGATTAGTAAATTTGAAT

>BPA_10773

TCCACCACCAAATGAGCCTCTGGGCATCATAGGTGTCATGGGTGTCATAATATCGGGACGACCACGCGGCATAAAAGACGGCGGTGCAGCACGTTTACGTAATTCCTTATCTTCATCAATCAAAGTTCCGTGTATTTTACCCATAATTATTTCTTCATCGCTAAGTTTCGGTTTAGTTTTGCCACTGCGTAAGAACTTATTATCATTGCGTGGCAAAATATTGCCATGAGCCTCATTTTCCGGTATATGCTTAAGGACCTCTCCCTCACCATTGGCAAAACGTTGTATTAGAGAATGTAATTGTTCCGGAAATATTTCTCCATCCAAATCAGTATCGAAATATTGTTTCTTTTGTTTTTCAAATTCTTTAATTATTTCACTTTCAAAACCTGGTTTTAAATATTCTTCCTTAAGATTTTTCTCTACAGCAATGCCATTTTCTCCTTCCATTTCACTAACAGCTTCTAGAATTTGTTGCATTTGCCTTTCAAAGTGTTTTTGCAATTCCATGGGATTAGTGAAAATTTGGAATGCAAATTTACGATCATCANNNNNNNNNNNNNCATCATCAAATAGTTCATCATCAGTTTCAGCCTCTTCAAACCACTGAGGTTTACGGAATTCATCCTTGGCCGACGCTGTCGAATCCATATTCTTCTTGGCCTCCTCTTCGTTTTGTTGGAACATTTTCT

>BPA_10776

CTTGTTGTTCTCTTTATTGGATTCCCTCAACATTAGAGTAGATATTCTCCATAAAAGGAGTGTTTGTCGCTTTTAAGTGATTTTAATTAAAATTAAAAGAAATAACTTCTGAAAGTTTAAATTTTTGTTGTCTTGTGTGTGTGTAAAGGTTTGGGGTAAACCGCAGTAATAACTCAAGGAAGCAGCCGTTAACAACAATAACTACAACTTAAGACTAAAGTCAGCTATAAAAAAGGAAACTAAAGAAAAAAATTGCCTAAAAACCAAACAGCTAAAGAAGGAAACAACTTGAAGAAAACGTCAGTAACATGTTGAGGAAAATGGGAGGCGTAGGCTTGTTAGCCTGCGGTATTTTACTGTTATGTGCTGTCAGCAGCGTCAACTCTTATTCAAAGTATGGGCGTGGCTGCAATGACATTGGCTGTTTGCCCAATGAGGAGTGTGTGATTAACACTGATTCTTGCAGTTATAGTCAACGTGATGGCAAGGATTGTGGCAGTTATCCCACTTGTAGAAAGAAGTCTGGCGTCAGTACCTCTTCTTCGTCATCGTCAGTTAACCCTTCAGTAAGCGGTGGTAATACGCACAATTCATATACGCCTAGTGCACCACAAACACCAAGTGCTCCCGTGGCCGATTTTAATGGTGGTGCCGGTGGCGGAGGCAGTAGTGGTGGACATGGTGGTGGTTTTTCGGCTGGTGGCCATAGTTTATATCCTAGTTTACCCTCTGACAATAATAATGGCGGTGTAATGGGTGGTGGCTATAATCCTTATAGTGGTGGCTATCAACCAGGCGGTTATAATCCCGGTGGTTATGTACCACCTGGCTATCAACCTGGCGGTGCTGGTGGTTATCAACCTAGACCCGGTTATACTCCTCCGGCTCCTGGTTATAGTGGTGGTGGTGGTAGCAGTAGTGGCGGCAGTGGTGGCTACACGCCCTCAAAACCTAAAGACAAGGAAAGTGGCGGTGGCTTCTTCTCAAACTTTTTCTCCAATCCTGCTGTAAGTCAAGCTGTATCTGGCATCATTGCAGGACAAATAGCCAATACATTGCGTGGTGGTGGTAATAATCCTCCAGCTCATCAGCCTAGTGGTGGTTATCAGCCCAGTGGAGGTTATAGCAGTGGTGGTTCTTCTTCTGGCAGCAATGCTGGTAGCAATATTTTGGGTGGAATATTAGGCAGCGTTTTAACTGGTGGTGGCGGCAGCAGCAGCAGCAGTAGTAGCAGTGCAAGCAACTTTTTAAGCAGTGTTTTATCCGGAGGCGGTGGCAGCAACAGTGGTGGTAGCAGTGCTAGCAGCTTTTTGGGTGGCCTTTTGAGCGGTGGCGGTAGTTCGGCCGGCGCTAATCGTGGCGGTTCCGGTGGTTTCGGAGATATTTTATCTTCTCGCAACTTTGGTGGACTTTTCAGCGAAAATCCCTCATCCAAATC

>BPA_10777

AACAATTTGTACTTGTATTTTAATTTCGGGTTCCTTTTGATTTAAACTATTTACATAAGCTTCTCGGTGCGTAAAAGGATTTGGAGGTACTAAAGGCAAAGGTAAAGCAGGAGGCACTATTGGTTCGGGTAAAATAGTTTTATGTTTTATTGGCGTTGTATTAATTGCATCCATTTGGTTTGTGGAGGAGGAATTCACATTTAATTCATGTATTGAAGACATTTTTGGTCTTTTAGCTGAATCCGTACTTTTACTTTTCTTTTCGCCCTTCTCATTTTCATCCTTATGTTTGCGTTTCTCTTTTTTCTCCTTGCGAGCACTAGAAGTTTTAGTCGTATTATTATCACCTGTACCACTGCTACTGCTGCCTTTTGCGCATATCAATTTTAGGTTCTTCTTTTGTTAATTTCTTTTTGGCCGGTGACACACTAACACTTCCTCCTCCATCATCCTTAGAAGCGGCATTCGATATTGATTCATCAGTGCCACTTTCACTGGAACGTCTACTTAAACCCTCAGCCTCTAGCTGTTTCTTTAA

>BPA_10783

TTGGTGTTGCTATTATCATCTTTCTTTTTATTGTTTTGCTGTTGCTGCTGTTGTTGTTTTGGTGCTGGAGCAAGTTGAACTGTTTGTGATTCTTCAACAACTGGACTACTATTACGTGTATTTTGTTGTTTGTTCTTCTTTTTATTTTTACTTTTTCCTCCTCCGCCGCCACCAACACTATTACTACTAGCTCCANNNNCAGTAGATTTATCATTGTTTTGATTTCCTGTAGAAGCCTTAATGTCAGTACTGGAACAAGATCCTTGATCAATACCAGAATCTCCTTCTTCACGGTAATTTTGTTGATTTTGTTGATCATCATCTTCGTCGGCAGCAGTGTTGTCTTCATGATCGGATTCCTCATCTTTGTCGGAATCTTTATCATCTTGCTCCTGTGGATTACTGGAACCACCTGTGGCCTCCTCCATTTGTCTGCGTTTCTCCTCTTTCTTTTCCATTTTCTTTTTCTTTTTACGTTCGCGGCGACGAGCAGCAGCTGCTCTTCTACTTTCCTCACGCGTTCTCTCCATATCTAATTCCTCCAATAATATTGAAGCATTTTTGTTAGCCTTAACTGCTTGCGCCTCCTTTGCGGCACGAATGATTTTCATGCAATCGTAACATTTTTCCGACAGTTCTTTATCACTTATGGTACTAATAAAACGGTTCATTTCTTGATCAGATGGAAACTGGGTTACATATTGAACCATCCATCTCACGGTCTTCGTGTGTCCTTTGCGGAAGGCTGCCATTAAACAAGAAACCCTCCTGTTGTCTTGAGAATCTATATCGGCATTATGCTGGTACAATATATCAACAACGCTTAGGTGACCACCATGAGCCGCCAACCATAGTGGAGAATTTCCTTTTTTATTTTTAACCTCCACCGCTGCTCCACGAGATAACAACAATTCAACAAACTTCAAATGACCCTTATCAGCGGCAATAGTCAAAGCAGTATCTCTGGATGTGGGAACGGGTGCTGCATTAACATCAGCCCCTTTATCAAGAAGAACACGGCCAACTTCTATATAACCTCCAGACGCAGCTTCCATTAGCGGTGTTAAACCGGTCTTAGCACGATGTTCGACATTTGCTTTACGATCTAACAATAAACTAACTACTTCATGTCTACCTTGAAAACAGGCCAATGTTAAAGCAGTATTGCGATTAGTTTCGATCTGAGCATTAATATCAGATCCTTGATCTAAAAGCAATTTCACCGCAGCCGTATGACCATTCATAGCGGCCAACATTAACGGTGATATTCCCAATTTACTGCCGGTACGTGAATTTATCTCAGCCCCATTACTTAAGAGTAATTTTATAATGTTCACATAACCACCACTAGCAGCCAAACTTAAAGGTGTATAATCGGAAACATTACGATGCTCCTTATTTGCTCCAATACCCAATAGTAACTCGACAACTTCATAGCGTCCACCAGAACAGGCCAACGAGAGTGGTGTGTCTTTAGTACGTTCCGA

>BPA_10793

TTTAATACCAGCCGAACGGCACTTGGCTACAGCATCGGGTACGGCAGCACGAGGAGGATCAATCATGGACATAAGACCTACGAAACGCAAATTATCAATGGGGAAGTTAACATCATCAGTATTGAATTTGAAGCCAGTGGGATATTTGTCGGAGGGCAACATGAAGTCACAGAAACCTAATACACGTTCACCCAAACCTCCCAATTCCATGTAGGCATTGTTGAAAGCTTCTTTCATCTCTTCGTCCAAGACCTTCTCCTTGCCGTTAATGAAAATGGTAGCGCAACGTTCCAAGATACGTTCGGGGGCACCCTTCATGACGAGCAAATAACGTGGATCGGAGGGATCTTCAGTTTCGTGAATTGAGACCTGATACTTGTTGGTGGAGTTGAATGGAATTTCAGCGATTTTCTTGTTGCGTTTACGAATATTCATTACATCACCGAGAGCCAATTCCATACACTTCAACAAAGCAGCTTCTGAGGCGTCACCGCTGACTTCTTTCTTCAAAATTGGAACACCTTCT

>BPA_10794

TCTTTAATGTCAGGCAGGGTAGCAATATCACCGCGAGCTGCCAATGAAACAGGAGTTGATAATATACGTTTGGCGACTCTTTGAATATCAGCAGCAGTAATTTTGTCAATTTCGTTAATAAAATGTTCGGGTCTCTTACGATGTCCCGTAGCCAATACTTGACGTCCCACATCTTCGAAAACCACTGGTCTGGATTCCAAGTTCATTAGCAACATAGATTGAAGTTGAATTTTCGAACGACTTAATTCTTCACGTCCAGGTATTGAGGTCATATTAACTAATTCTCTAGTTATTACTTCGACCATATCACGTACATTATTTGGTGGAGCACTTGCATGTATACAGAATAGACCAGTATCGGCATAAGCATGATTGTATGCTGTGGCACTATACATCCAATGATAACGATTCAAAACATTTGTATAAAGGCGAGAATACATGCCTTTGCCAGGACCACCAGCCGAAAAGGAACCACCACCGCCCATCATAATATTCAAAACACACAATGCTACAAAATCAGGATCTTGATGAGAACAACCCTCTAGACCAATAACCACATGGGCTAGTTCAGGTAAACCAGCAGCGGCATAAATTGGTATTTCGCATTCTTCTTTACGTAAACCGCCCGTGTATTGGGCAATAGATTTATCTACTTCTTGAGCACCAGTACCCGAAATTTGCTCTTTTTCCCAAATAGCAGCATCCTCTACAAAGAATCGTTCTACATTGCGGACTAATTCGTCGTGGTTTACTCCTACACCGGCAATAACCATGCGCTCTGGTGTATGATGATATTTCAAATAGTTCATTAGAACATCACGATTTATAGTATCTAGATTTTGGGAAGGGCATAATTTAGGTAACCCCAGAGTATTTTCTCTATAGGCAGCTGCATGTATCATATCCATAAGTATGGGTTCTTGTTCTGGACGCATACCCAATGTTTCCAATTCAAATTGTA

>BPA_10795

ACGACACCGTATCCGTTAAAGTTTGTAACATGGTTAAAGCCAGGGGGAGGCATGCGAGCGCGTTGCATATATTCCATCATATAATGTTGTTGCAGATGTTGTTGGTTATTTGGAGCTTTATTATTTGACTCTTTCGAAGAAATTAGAACAGAATTTTGTTCTTCTTCATTTTCTATTAACTCAGCTAATGCCTTTTGGGTTTCAATGAATGGATCAAAACCCAATTCCATTTGCACTGTGAATAAACTGGTTTTGTTGTGTTGTTAAAGGTCCAATTTGAGGATTTGCAATCTGCTGAACTGATTGATGGTTTAGCATTTGATCAATTTTGCCATATATTTTTTGTTTCTGATGATTTTGGGATGTCAACAAACAGTTTTGATCAACCAACTGAGAATTAAGCTGATTATTATCAATTATTGGCATCATAAGTAACTTGTCATTCATATTGCTTGTGCTGTTTATTTGTAAACAGTGTTGTTGTAATTTTTGTTGTTGTTTATGAAAGTCGAAAAACTTTGACATATTAGTCCCAAACAATGATTTCTGTAGACCA

>BPA_10797

TTACAAATGTGTGGAAGAGCATGATCCATTACATGTGATTACCACCGATATCACAGTCGAAAATGAAATGAAACCTTTAGGAATCCATTATGTACATACCAAAGATTTGGCCAAACCGGAAGGGGCTTTAGACATCAAACACATACAATTATTTGAATTGAAAAATGTTGAGAACTTTAACTTAACTGGTGAATTGTATACAACAACGAAGATAAACGGCCAGGAATTCAAAATTGTGGCCATACATCCAAATCGTACTGTGATATTAACCACCAACTATGAGGAGGAAAGTGAAAAGATGTTTAAACATCGCAGCAAATTGGAATTATCGGAAACAGCTTGGGTTGGTTACAATCTCGAAATGAGTAATTATACTGTGTCTGGCAATGAGTCCCGCAAATTCATTATTGAGCTGTCTTATCCCAAACGCACTTTATCCACTGAAGGTTGGTACTACAATACCGAAAGCAAATTCTTCTCCGACATTGAATTCAAATGGACCAATCCTGAATCATTTGATGATATTCTAAAAGTAATACGTACATCTGTTTTATGGCAGGATGAACCTTTG

>BPA_10803

CTTTGGTAGAGTATTGTCTTTAGACGGTGGCGTTTTGATTTGCGATCTGAATCATCATCCTTAGGTGTTGAAGGTGCTGAGTCTGATGTAATCGAATTATCCGACTTAAGTTTTCTTTTGACTCGGGCACTAGTAGATACTGGTTTATCTTCTTGTGATTCTGGTGTTTTATTTTCAACAAGAGTCGATGGTGCTGTAGATTTGCTTTTCGCTCCTTTGCCAAGACGTCCTACTTTTTTGGTTGGTGTCGCCGTTGAAGCTTCTGCCGCTGTTTCTTTTTGTGCACTTTCATCGGAATCCTTAGAATAATCGTCAACATTCGATTTGTTAGGGACAGCTGNNNNNNTGAAGAACTACTTTTTACAGACGTATTATTTTCAGCCTTTTCAGTCGATTTTGTAGATTTATCTGATTCATCATTCTCCTTAGGAGCTGCTTCTTTTTTATTTTCTGTCGCTTTAGCATCAAGTGTTACTTTTGCCAATTCTGTATCTGCTTTAGTTGCAACTTCCTCTCTCACATCAGGTTTTGGTGGCAAAGCTTCCGTTTTTTCTTTTTTATTCGCCTCCAAGGAATTTT

>BPA_10809

CATCATACACCTCCTTGGAGGATTTAGAACATGAGGGAAAAAATTTAAATGCCGTTACCTCAACACCAGTGGTGACCAGTGATGCCACCATTAGCTGTGATGTTAACTCTACACCGAGAGGACAACAAAGAGCAGATGCCACAGAGCAGCAACAACAAAGATTGCAAGAATCCGATGAGGCCAATGATTATGATGAAGATGAATCGGCTGATGAGAAATCCGCACAACCACGTCAGCTTATGCATTATAAACGTCGCCTTAGTGGTCGTAGTCTGGCGGATAAAACAGCTAGTGTTTTGTTGGAATATAATCCAGATAATCCCAATTCTTTGCGCAAAAAATTTCGCTTCAATCGTTATGTTCAAAACAATGGCGATGAATCGGGTTTTGTAGATGCCAGCAATAACAGCCAAACCATGAATAATTCTTCGTCGCTATTAACATCCAATAATACAAATCAAAGTAATACAAATAATAATACAAAATCGAATAGTTCTTCGGATACCTCTTCACCGCCACAAAGTAATGGTAATTCTAGTCCAGAATCGGGTATAGGAGAACGGGAAGATATGAAATATATGTGTCCCATCTGTGATGTAGTCTCACAAACTGCCCATCAATTCACAAATCATATACGTTGTCACAATTATGCCCCTGGTCACACGGAGAATTTCACGTGCCGTATTTGCTCTAAGGTGCTCTCATCTGCCTCATCATTGGATCGTCATGTTCTAGTGCATACGGGGAGAACGACCATTTAATTGTAAATATTGTCATCTTACATTTACCACAAATGGAAATATGCATCGTCATATGCGCACTCATAAACAGCATCAGCATACTCATCATCGACGTAACTCGAATGGAACAAAGTTCAACTCAAACAATAATAATAACAACAACAACAATAGCAGCAACAACAACAACAATAACAATAATGTAATAGAACAAAACGACAGCGTTAACAGTACTAGTAGCTCACCGCAAACGACAAACGCCAATAAT

>BPA_10816

AAGATTTAAGTCCTTCACCAGCAGAACAGACTGCTGTAGGTAATTTAGTATCAAAAGTGCAATCAGTACTCGATAACTTAATTGTGGCACCTGGAGATTTCAATAAATGCCAATTGGAGGAAGTACGTCAGGTTGGCTCATTCAAGAAGAAGGGCACCATGTTAACTGGAAACAATGTTGCCGATATTGTAGTCATACTAAAATCGGTGCCCTCAAAGGAATCTTGCGAAGAATTAGGCAAAAAAGTGGAATCAGACCTAAAGAATGCTATGAAAACGGAAGTTCTTACTAAAGCCGATCATATTAATACCACTTTTCACGATCGCGGCTTTGACATTTTCAATACACAGGCCAAAGTTCGCATACTTATTACAACAATACCACAAAATATGAGAAAAAGTGAACCGAATATGTTCCCCGATCCCAAAGTAATGCAGGCACATTTAGCGGCTATAAGACACACTCGTTGGTTTGAAGAGAATGCAGCACATCATTCGTCTATTAAAGTTTTAATACGCATTCTTAAAGATCTATCGAAACGTTTTGAAGCCTTCTCCCCACTCTCACCTTGGATGTTGGATTTAATGGCTCATTTAACAATTATGAATAATCCTTCCAGACAAGCTCTACCTATCAATTTAGCTTTCAGGCGTGTATTCCAATTGCTCTCAGCAGGTCTATTTTTACCTGGTTCTGCTGGCATAACAGATCCCTGTGAACCAGGACATAATCGTGTTCATACCGCTATGACTC

>BPA_10817

GTACATTCGGCCTTACTAGTAGGATTTGATAAAGCAAACACAACTGGACGATCGTTATTTGCAGCCATAGTTTGTAAGATTTTAGGAGTAAAAATACCAGCGGCAGCGGAGGCACCAATTAATACATTAGGTTTGACTTTTTCAACAATTTCTTCCAAACTCTGCATTGGTTCTACATCTTTAGCATAGTTTAGTTTGTGGCCTTCCAAGCTACCCTCTTTGCGACTGGTTGTTAACAAACCATGAACATCAACCATCCAGATTTTGCTACGAGCAACATCAATTGGCACACCCTCAGCTACCATGGCCTTAACTGTCAAATCAGCAATACCAATAGCAGCTTCACCAGCACCAGCAAATAGGAAAGTGTAATCAGCAAAACTCTTGCCTGTAATACGTTTCGAAGCGTATAAACCAGCTACCGCTACCCGCTACCGATGCTGTACCCTGAATGTCGTCATTAAAGGTACAATATGTATTACGATACTT

>BPA_10822

ATTGCTATTAGCTCCCCGACCTCGTCTCTTTTTAGTACGTCTACTGGGTGTAGCAGTAGAATCTCCAGCTGAATCCGGTATAGAATCTATTGTTTCATCTCCTACTTCATGATGGCCAGCACTGTCATCTCCCTTGGGAATTTTCGATACTGGTATGTCATCGTAATCGGGAGATTCTCCATCTACAGTGGAACATTTTAAGTCAATATCCATTGTGGTAGTCT

>BPA_10828

GCGACGAGGAGGAAATTGTTTTAAAATATTCACAATAACAATACTTAAGCGTACTGGATCAAAAACTTGCATCAAATCGGTAGCTATAATATGTAAATTTTTCAATAATGCGCCTTCTGATTTTAAAAGATCCGTAGGACAAGCTATCATTTTAATAAACATTTCCAAAAGTTCATGTAATTTATTGGCAAAATCATCATGATCTGAAT

>BPA_10840

ATTTAGCTTTATCAGCTCAAGAAAAGAAGATAGCCCATGTCGTTTTATCCGGATTTAGCTATTCGGTCGATGCAGCACCATCAAAATCTAATGACAAGCAACTTCAAATCGAAGATTCCATAAGTTCCGTAGAAAGGCAATTGTACTTTACTACAGCACATGCTCATTTTAAAGCAGGTTGCCCTGCGTTAGCATTAGAAGTATTAAATAAACTTCCAACAAAGATTGTAGATTCAAA

>BPA_10841

AATGGCAAGATGGCCTATTGGGTTTAGCAGTTAGAACGGCTGTTAATGTAGAAGACGAAATACATCAATGGATTGTTAACGATGGTCCTGTGGATGCTGTTTGGATAGAAAATTTAAATACTGTTTTGGATGACAATAAAATGTTATGTTTGGCCAATTCTGAACGTATTAAACTCACTGCCTGGGTTCACATGGTATTCGAAGTACAAGATCTAGTACAAGCTTCTCC

>BPA_10865

CTACTTCCTTACAAAAAGCAGTTTATAAAGGAGATTTAACGGATTCCGATGTTTTGCTGGATTATCTTATGAATCAGCCGCATGTTATGCCACGCTTAAATCAACGTGTTTTGGGTAATAATGAAAATGCTAAATTCCTTGATTTATCAGGAGAGCCTCACAAAGATCTCAATAATGTTAAAGCTTTGGCTCAACTGTCCAATCG

>BPA_10869

TTCACACGTGTACTTTTGGTCAACACTGTTGGTACTTTCAACGTGATTCGTCTTTCGGCTGGTCTTATGGGCACCAACGAACCAAACCAAGACGGTCAACGTGGTGTCATTGTTAACACTGCTTCCGTAGCTGCTTTTGATGGTCAAATCGGTCAAGCTGCCTACTCTGCCTCCAAGGCAGCTGTTGTTGGTATGACTTTACCAATTGCT

>BPA_10874

TATTAATACAAAATCAAAATCAAGCTGCTATTGTTAGTCTACAACAGACATCGGCTGGTAATTATATACCCGTGTCTGGTGTACAAACAGTAACCACAAGTGTAGCGGCGGGTGGAGCACAACATCAGCAGCAACAACAACAGACAACCATTGTTACAGCCGACGATTCGGATTCGGATAAACCTTCTTTGCAAAAGATTTGTAAAACTGAAGCCGTGTCTTCAG

>BPA_10882

TGTGTTGGCATGTTTATGGCCTATTGCAACGTAGTGATAAGAAATATGATGAAGCCATTAAATGTTATCGTAATGCCTTGAAATGGGAAAAGGATAATTTGCAAATTTTAAAAGATTTGTCTTTGTTGCAAATACAAATGAGAGATTTGGAGGGTTATAAAGAGACCAGACATCATTTATTCAAATTGCGTCCATCACAGCACGCATCGT

>BPA_10890

CCACAGTTAGATCTCCAGAAAGTACGGCTACCGAATGTCCGTCTGAAGATAATTTTCCAGCTAACCAGCCAGCCGTTTTACGAGTATGACAGAAAATAATAGCTTGTCCAATTGTAATACTGCCATATATGTTTTGAATTGCCTGATATTTTTCATCTTCCGAACGACATCTTACATAATATTGTTTAATGTTGTCAAGTGATTCTTGTTCTCTTTTGAGTCTAA

>BPA_10909

GAAATACCACCGACGCCACTGCCAGTTGTTGTTGGCTGAGGCGCTGGTCGTTTATTGTGACGTAAAGGTGCTGTTGGCGGCATTTGATGCCCAGTTGATTGCAAAAACCATGGATCGCCACCGGTATTGGAGGCAGCCACTGTTACGGTTGGTTGTGAGAATCCTCGATGATGACCCACGACAGTTGTTTGTCCGTTTTGTGCAGCTGTAACGTGACTATTCATAACTGTATTATTTGCACGTATATCCGCA

>BPA_10912

TCTACCGAAGAAGACAAAAAACCAAATGCAGAAAAGTCGGGAGAAGAAAAACAAGAAGAAGAAGAAAAAGAAAAGGAAGTTAAAGAACCAAGCTCTGTTGAAGAAACTAAAGAGGATAAAAAGAAAGATTCAGATGTTGAAGTTGAGGGTGAAAAGAAACCAGAAGAAAGTGAAGATAAAACTAAACCAGAACCTGTATATATAGAAGTAGAAGAATATCTTGTTAAGTATCGCAATTTCTCCTATTTGCACTGTGAATGGCGTACCGAAG

>BPA_10937

GTAGTGGAAGAGGTTAAACCCAAAAAGAAAGGAAAAGGTGGCAAAAAAAGGACGGTCGGCCCGCAAATCAGCTGACAAGGAATCCACAAGCCCAGGTGGAACCTCGGAAAATGAGGGTGGTGAAGATGGTATGGATCGGGAGGATAGTGTTGATGGCGACGAAGGCGATGAAGTTGCCGAGAAGGGCAAAAAGGGTAAGAAGAAAGCCGCTCCCAAGAAGAA

>BPA_10953

GGGATTTCTGGCAATTTGGGAGCATCTAAATAACTGGGAGTAAGAGATTTTTCTTCCTCGAAGAAGTTCTTTTCCATATCGCTGATTTTAGCTTCATTGCTTTTGTCATTTATGACACAAACTTCTTGTAATGCGTGATTTATTTCTAAGTCCTTGTGTTCTTCATTTGTTATATCAATTTCTGCGGAATTCTCAACTTCAATTTGTTCTTCCATTTCCGTCTCCGC

>BPA_10960

AAGAAATTGCAGTCCATTCGATCGGAATTACAAAAAGAAAAATCTAATAAATCGGGAGAACTTATTGAATGGAAGGAAAAGTACAATATTTTGGAATTGGAAAACAAGCGTTTACATGATTCCCTCTCAACGGATAATCAGCAATTACACACACATATTAAGGAATTGGAACAAACTGTTAAAGATTATCAGTCTCATTGTGATTATAAAGTTGCGGAG

>BPA_10987

TGAGACCATTGGTATGCATTTCATCTATAAGCTGACGAGCTGAGGCAGAATTATCCTCATCACGATAAGTGCTATCACGACTTGAAGACAGGAGACTGGTTCTTTTGGGTGGTGCTGGTGCTTGTTTGCCTTGTTTATTGGTAGTTCTACGCATTTGTACATTACCCTTGCCTTGACTGGAAAACGTTAAAAGCTTTGTGGAGGAGGAACCAGTTTCTGACATGG

>BPA_10990

TTTGGGACAAAAATATACAAAATTTGCTAAAAATCTGGCAGAAAATCATCGCAATGAAAAGAATCTTAATAATGCCTGGCATAATAATCAAAATCATATTAATAATCAAAGAAGTAACGGTAATATTTACGATAATGTACCACCGCCCAAACAATTGAACAGTCAAGAGAGCAGTTCCAGAAATGGAGAATATCTGACAAAATTAAAACATGCCAATGAGGCTCATAGGCAAGCAGTGAC

>BPA_11004

CTGGAGCATTGTTAGTAGTTAATTGTTTTTGTTTGGGTAAATTCATGCTTTCAGCCAATTCGTTAACTAATAGGGATACTGGATCTACACCACTAGAGTTACCATTTTGTATATTGGCGGTGGCCGAGGAATTCTCTTTGGCTTGTCTGTCTTTTATTTCTGCCATTAACTTTTCCTTCATGGTTAATTCTTGATTTTGC

>BPA_11009

GAGAGCCAGTTTTGAGCTTAAAGTGCCAAAATCGAAGATTGATTTTAAAGTTTCATTAAAACACGAAGAACGAAGCAAAAATGGCACTGAGCACAATGTTCTATTGGGTGTACGTTACGCTCCAGAAAAAGAAGCCACTGGTTTATTCTCCATACATCTGCCTAGAAGAAGCCTTTTCGCCATAGATGTTTATGTCAACATAAC

>BPA_11011

ATTTCTTGTTGTTGTTGCTGTAAAAGTTCCTGTTCATGTTGTTCTTGACATTCCTGCTCATGACGATTGAACGAATCATCAACCAGCATTTGTTCATGTTGTTGAGTTTGTTCAAATTCCTTATGATGTTGCTGCTGTTGTTCTTGATGTTGTTCCTCGTATTGGTGGTGATATTGATCATGATAAACAGCTTCATCGGGAGAAACGAGATGTTCGGATAT

>BPA_11025

TTATAATGCATCTTTTGTGATTTGGTCTAACTCTGAGACCTTCTTCGTCTACTTGCACATTCGGTGATTCCCTAAGCACTTCAGTTATAAGTTTTATATCTTTAGTTAATTTTTTAACTAAATTAAAATTGGCCACTGTCCATATAGGTACATATTGATCACTATCCATTTGTGATAGCAGCCAAGAATCGTTTGCTAAATTTTCTC

>BPA_11042

CGCAGCTCAATTAAGTTGGCTGAAAAATATGATACGGAGACCTTAAATGTATTTTTTCCAAAGTACAGCTCCGATCAACCCAATACATACACCTTTACTAAAAGTTTAGCTGAGCACATTATAAATGATTATCGTCATCAATTACCCATTATGGTATTTAGACCATCCATAGTTGTTTCAAGTATCGAAGAGCCAGTACCCGGTTGGGTAGATAACTTCAATGGTCCACTTGGTATGTTG

>BPA_11072

CTTCGCTGAAATATTGTAACTTGAATAGTGTTGAACCACCCTCCACTTGTGTTTGTACCAATGTAGGAGGAGTAACCTCGCAATAGCCGCGATCAAAATAATGACCACGGAATGCTTGCATCACCACAGAACGCATTTTTAAAATTTTCGAAGTATTTTCACCTCTTATCATTATATGGCGATTATCCAATTGTACATCAGGATGAG

>BPA_11081

CCAAATGGTGATACCACAACAACAGAAGCTGATAAAGGTAGTGCTGAACCACTATCACGCAATATGGGTAGCCATTCAAGTTTGGAAGATAAAAATCCTGATGTTATACCACAGGAAACGAATAGTGAAGATGAATTTCATTTAGAGGAAAAAGCATTCGATCGTTTGAATATGGAATCACAACGTATTTTATATACGCCAACAGCTAGAATAAATACACAATCACCACCACCACCCTCCCTATCACCAACCTTTGGTAAACAGTATGGGGAA

>BPA_11086

ATATCACAGATCCCAGTGTAACTGATTTAACTTGGGAAAAGAATGGCACAGATGTGAGAAAAGTTAAAGAACTTGAAGGGCGCTTTCGTATAATAGCAGCCGAAAGAAAATTCATCATTGATAAAACAGAGGTGAATGATGATGGTCTCTACTCATGTGTGGCCAATAATCAAAAGAAAGATATTAATGTAGTTGCTAATGTTGTTGTTAGAGTGCCTTCAAATAGTGGCGTTGTTGAGGGTGAAAAATTGACAATTATCTGTACAGTTGTCGGTACTGATCCAAAATTGTCATGGGCTTT

>BPA_11091

TTAACAAGGAAATTGTTAAACTTTCATTATCGCAGTTTGCAGGAAAATATGTAGTACTACTTTTCTATCCATTGGATTTCACATTTGTTTGTCCCACCGAAATTATTGCCTTTTCGGATCGCCTTAAGGAATTCCATGAAATAAACACAGAAGTTATTGCTTGCAGTGTAGACTCTCATTTCACCCATTTGGCTTGGATTAATACACCGCGCAAAGAAGGTGGTTTGGGA

>BPA_11094

CGATAACCCAAGGCTTCCAATGTTTGTAAAAGTAACCAGCTTAAACGATAATCCATAGGATCAGATGTGTGAGTAGCTGGATTGAGTAAACTCTCTAAAGGATGACTACGTTTGGAAAATAACTGCAGCAAGTGATAACGTAAATCATAAATATTGTCTTTTTCATTGTAATGGGGGACATTCTCCATATAAACAGGTGTTGGAGGAAGAGAATAAAATTCCTCCGACTTGAA

>BPA_11103

TTCAATTTTATTTGGTATTTCTCTATTTAATTTGTTGTGGGGTGCAAAATCGTGACTTTCTTTTAACTGTTTTTCATATTCTGTTGGACTGATAAGTGCTATTTCCGTCAGCCACACGGCAGATGCTTCAAAAAATTTTAAAATGGCAGTATCATTTTCAGGTTCCATTAGACAATTACGTAAACATAGATATTGTTGCATTTGTT

>BPA_11107

TAAATAGTCCCTACTTAGCTGGGTTCAAACTTATGACTATTGAAAGAATTCAAGGTTCTCCAGAGGGTCTAGTAGAGAGTATTAAATCAACTCTAGATTCACCACATCAAACAAATCGTTGTGCTCAGGGTTTAAATCTAACAAAACATCCCACACAAATGGATGTTTATTATGTTTTAACCGATGAAGGTTGTGTTCACAAATGTTCCAC

>BPA_11109

AAGTGATACTAGTGGTGTGGTTATGAGACGCAAATATACAGCTTCACCCATAATGGTACCATATCCCACACACAATTTACATGGATCACCTCTCAATAGTAGATCCCAGAAATGTCGCAGTCATTCTTTAGATACCTCCTCATTACCCAATTCGTATCATCATACCACCCATCAACCAGCACAATTTCACAATCATCAACATCATCATTCGCAGG

>BPA_11116

CTCTGCACATACAATGGGGAACACCATCGCCTTCTTCACATATACCGCCATTTTTGCAGGGATTAGGTGAACAAAATTTTCCGTATTCACATCTTTTGCCAGAAAGATTCATTGGACATGCACACGAATAATTATTAGGTCCAAAATGTTCACAGTGGCCACCAAATAAACATGGTGATGAGGCACATGGATCTAAATCAATTTCGCATAATTTTCCAGTAAACCTTTCAGG

>BPA_11144

AGCTAATGGTGATCGTATCTTATGGGGTAATCATCATTTTTTCCGTGTAAATTGTCCCAAATCAGCTAATGCCAATATGAATTCTGAACCGCAAACGCCGGCTCAGTTAATAGATTATAATTTTGCAAGAGATGAAATAATGCAAAATGAATTAAGCAATGATCCTATACAAACTGCCATTGCTCGATTAGAGCGCCAGCATGAAGAGGACAAACAAG

>BPA_11150

CTTTCCCTAACGGAACCCGTTGAGCCTTTAGATTATGAAGAATTTTTAAATTCCCACATAAATACCATAAATCGTGATCCTTTAAAGAATATATTGGATTTTCCACCAGGAGATGTTACCGTCAAAACCATACCACGGAAAATAAGAACCATAAAGCATATTGTACCAAAAGAAAATTTTGCTGAACTCCCACAACATGTACAAGAATGTGTTAGTTGTTATACGCGACCTTGGAAAGTGGTGGAATATGCCCAACGTCATTATTCTAGTTCATGTTGTGCCCGGGAACGTATTGATCGTGGCACCATCAGTCCATCGGCTTATCAACAGGAATTCGAAGTAGATAAAGATTTTGCATCTTTTGACGAATCATCGCTAACCGATCAAAGTAACAGTTGCACTACTAGCTCTCGGCAATCTATAGCCAGTTTATCATCCGTAAGTTCTTG

>BPA_11151

ATAACCAGATACGAAACATGGAGACCAATATTGAGTTGAAAAAATTGCAACAATTGAAGGATGAAACTGGTGAATTGTCATCCGCTGATGAAAAACGTTATCGTATATTGAAGCGAACGGCCGAAAATCAACTTTTGGAAGCAGCTGACGTAATTTGTTGCACTTGTGTAGGAGCTGGTGATGCTCGATTGCAACGCATTAAATTTACCTCGATTTTGATAGATGAATCGATGCAATCTACCGAACCAG

>BPA_11152

CCGCCGATCTTGCCATAGAAAACGAGTTGCATGTCTGTCCCAGCGAAATGCCCGATGAATTACGTAAACTTTTACCTCCCACCTCGGAGACTGTGATGACTAAACCATTTCCTATACGAGGCGAACGTGCCATATCTGACGTGTCCACTCCTCACGTTATTGCCATGGTCGGTTTGCCAGCCAGAGGTAAAACTTTCATCTCCAAGAAA

>BPA_11160

GTGGGATTGAGGCCAGTTGATACCAACGAAGCATATTTTTTGCCATCTATATCAGTAACAATTATTTCATTGTGTCTGGAATCGATTATATACACTAGCTGTGTAACCCATTCAATGGCCAACGATTTGGGATCATCTAAAGCGGGTATATCAAAAGCCTTTAATTCCTTGAGATCTCTACGTACTCTTACAGATGTTGTTTCTGTATTGGGTGTAGCAATTTTCATAGTGTGCACCTTACCATGATGAGAATCTATCCAGAATAGAACTTGATC

>BPA_11171

TTCAATATTTTGTAGTTGAGCATAAATTTCACTTAGTTCTGAACTTAAAGCTGCATCTTGGGTGCCACTATTTAAAAGTGCCATAATTTCTTTTTCTCGTGCTAACAGACGAGTTCGCTCTGTATCGCATTCAAGGACACTATCAACAGCAGGAGTATCATCACCCACGACTTCTTGTTCTACATGCAACACCGTGATGTGTGAGGGTATTTGAAGTTGACGTTCAGAAATCATACGTAAGAGAGTAGTTTTTCCTAAACCATTGCGACCCACA

>BPA_11174

GTGCTTTCTTTTCTGCAGCAGTTAGAACAGTAGTTGTTGAGGTAGCAGTTGGTGTTGCTGCTTGAGCGGAGGCTTGTAACTGTTCTTGCTGTTGTTGTTGCTGTAACAACAAATTTCTGGGAGGTCTACCTTTACGTCCTCGTGTGGCTGGAGTTGTAGCATTCGGTTTGCGTAAACGTGTTTGCACACCTCCTGCTGTCACATTACCAACCATTTCTGTTTGTAGTTGTTGTGTATTAACA

>BPA_11185

TGTTATTATTATTGTTGTTGTTGTTGTTATTATTGTTATTGGGCAATTTATGAGGCGGCTCAAAGTTTTCGGCACCATTGTCGGCATCGTCTGCGGGTCGTTTTAAGAATTTTTGTTGCACATGTACGCTGCTCTGTAAATTGCTCGATAGCATTGTCGTTGCCGTTTCTGTGGGTTTCTTTTCCGTCTTCTTGGCGGTCCTTTTATTTTTACTCTCGAGAAAACGTTTTTGC

>BPA_11187

TTAATTCACTATGTTCTACTTTAATTTGTAGATTTGGTGTTGGTGAAGTAGAAGTAGTAGTATCATTATATTTGTTGCTGCTTACCGATGACAATAAAATGGTATCGGTAATTTGTTTATCGCAAATAGATGCCACTTGTTGTTGTTGATGCTGCTGATTTTGCTGTTGTTCGACATCTCCGGGTGTCATATCTGACATACAACAAGTGGCAGCAACTTCTAAGATATTTGTACT

>BPA_11199

CCAGATCGGTCGGCACATTCACCTGACCAATATCGTCTAAACAATTGAAAATATATTTACGCGCTTCTTTACTTTTGGTATTTGTTTCAGCTTCATATTCTTCTATGGTCTTTAAACGCCAAAATGGATATGTTAAATCCGTGTCGCGATAAAAGAATTTCGATGTCTTTGAAGCACTGCTTAACATATGTTGCGTTGGTAAACCTTGAGT

>BPA_11214

GCTAATTCTATTTTTAGAACAGAAGGGTTAGTAGTAAACCTACCTCCGAATTCGCCAGATAACAGTGAGCATAACGAACGCAATACTATATTAGAAGAGTGTAATTGTTCGGATGACGATGAAATTATAGCTTCATTTAATGATCGTACTCGTTCTCCCAAGTCAGAGTTAGAAATACAACAAAATTTAGGCAAAAACATGTGCGGTCCATCAACTTCTACCAGTACCTCAAAAGCTCCTTCACAGTATTCAAAA

>BPA_11227

GCGGTTGATGCTGATGCTGCTGTGGATGATGATGATGATGAGGTGTTTGAAGATTTGTTACAGCTGATGATGTTGAGGACGATGAAGAGCCAGTTGTGGTTCCAATTGCTGCTGTTGCAGATTGTGGTGTTGGTGGTAATGGTGGTAGAGCTGAGACTGCATTTGAGGCAACAGCAGGTGTTGTAAAATGATGCTGTTGCAACATTAAACTATG

>BPA_11250

GGAGGTGTTAAATGTCCTTCCCAAGTATCGATGAGAAAACTAATGGCAGGTGCACCGATTTCCATGGCCTGTCCAATAATCCACGAAACATGAGAAGAGTAAGTACGAGACAACCAATTTGGACTAACACAATTGTGTAAACCTAAAGCATAGAGACCCAATTGAAAGGCGCACATGTGCAAACCACGGTGTGGTCCATGGTGG

>BPA_11256

GTTTGCAAACATAAAATTATTTAAGGGAGGAAAATGATCAACACCGGTAAATTAGCAGGTAGAACTTTGTTCGTTACTGGAGCTTCTCGGGGTATTGGTAAAGCTATTGCTCTTAAGGCTGCTCGTGATGGTGCTAATATTGTGGTAGCTGCTAAAACAACTGAACCTCATCCTAAGTTGCCGGGCACAATTTACACAGCTGCAGAGGAAATTGAAAAGGCTGGTGGTAAGGCTCATCCTTGTGTTGTCGATGTAAGAGATGAAAATGCTGTACGTTCAGCTGTGCAAGAGG

>BPA_11257

GGGGCCATTGTCATATGATCTAGGGTATGGGGATCTTGATGTATAATTTTATTGCAATTATTATGCTCATAACAATAGTTTTCTAAACTACTAACAATATCACCGGTTCGCTCGAAGGTTTTAGCATAATAATAGTAAAGAGATGTAAATTCTTCTATAGTTTCATCCGCTACATTGGAATTATTCATTTGATTAGCATCTGCCGTAAATTCATCTTCCCATTCTAAACAATCCATACCATCGGGTGTGGTATCAACTTCTTGTTCATTATTAGTG

>BPA_11266

GTTTATTGGTTAGACCTGTTATGCAACAAGGCGTAAACAAAATTGATGTGTATTTCCCTGCTATTGATGATAAGAAAACTGGTGATTTATGGTATGATGTAGATACTTTACAAAAATATGACAGAGTTGGAAAGGAAACTGTTGCAGTTGATGAGTATAAGATTCCTGTTTTCCAACGTGGAGGTTCCATTGTACCTAAAAAAGAACGCATACGCC

>BPA_11284

TTTAAATCCGAATGTGTGGTTAAATCCACATCGTTCTATGTTGGGGTTAGGCAACTATGACATCAATGTTATTATGCAGGCGTTACAACTACGCAATTGTGAGGCAGCCTGGTTTGATAAGCGCAAAGATCCCGAATGCATTGATTTAAGTGTTATAGTTGGTTTCATTTTAAATGTTCCCTCGGATTATAAATTTGGTTTTATAACACTACCTTTGCGTAAAA

>BPA_11331

GTTAAATTGCAAGCTGGCGAATATGTTTCGTTGGGCAAAGTGGAATCTGAATTGAAAACTTGTCCCATAATTGAGAATATTTGTGTTTATGGTGATCCTACCAAACAATATACAGTTGCTTTGGTGGTGCCCAATCAAAAACATTTGGAAGAATTAGCACAACGTCAAGGTTTAGAGGGTAAAACCTTTGAGGAACTTTGCTCTAATCCTGTAATGGAAAAGGCG

>BPA_11345

AAGCAGCGAAACACGGAAACTTGGATGCTGTTAAAGAGTTATTGGCCGTTAAAGCTCCACTCTTGCCTCGAACATCTATGGGTGAATTTCCAATAGATTTGGCTAAAGAAGCCAACCATGTTGATGTTGTTTGCTATTTGGAAGCTTATAAATTATGCCCAGCAAATACGTTGAAATCACAGTGGTATCACGGAACATTAACAAGAGATGAAGCTGTCGATGTTGTGAAGAATTTTGCTACACAATTAAAAACTGATACATCAGATTTAGATACGTCTGGCTGTTTTTTGGTGCGGTTTTCAGAAAGAAAGGTATCTGGTTCA

>BPA_11354

GTCACCAACATATTACGATTACGCATGGCCACCATCATTTTACGATAAACTGGTAGTGGCACATTAACCATTGTAACCTCGTCTTTATAACGTCTCCATAATTCTACCAAATATAATTCCTCCCAAGAATTCCAATAGCGTTGTTTCTTATGATTACTCAAATGTTCTTCTGCACTCTTATCCATGGGTGTTTGATGACGCCTGGGAGGTATACGAACTTGTTCCCCATAGTAACCTCTTGAAGATCTCCACGGTATGGACGATGCA

>BPA_11361

GAAACAAATATATACGTATACAAATTTGGGGTATCATGGAGCCCCTTTGGAAGCGGCTAATTTTTGCCGCTGTTTTAGCTGGAGCGTTAGTGGGAATTAATGCTCAATTTTGGAAAACTGCCGATACTGGAGCTATTTATAACTCTGCCAAACATTATAGACCTGAAGTTGAGGCTCCGAGAAATCCAATTGATGATAGTTATGCTATAATTGATAGTGCTACAACTACACGTACTGGTCAAGTGCCATCGAA

>BPA_11365

TGCCAACCAAGTATCGCAGATATGAGGCCCTAGGGGCCAGTATCCAACTATAGTAGAAACTGCAAACAAAGGCATTGAAATTAAACCAATAGCAAAATCAGCAATAGCCAAAGAAAACAAGAAGTAATTGCTTATTGTTTGGAGTTGCTTGTCAATTTTAAATGAAATCATCACCATTACATTTCCGACAACTGTGAGTAAACTAAGTATCCCAGCAATAACTCCCATGAGGATCATTGAAGACAGCGAGTACCGAGGTCCTCGAGAATCTATAATATTTGTAATATTTGTTTGGCTTAATACAGACTCATTTCCAAAAACTGTTTCTGTAGTTAGATTTTCT

>BPA_11374

AAGACCTCGTAAGGGCAATTTCATACCACCAACAAAGTTTCTGCTGGGTGGCAATATATCGGATCCTTTAAATTTAAGCTCTTTACAAAATGAGGCTTCGAATACCTCCTCGAACAATAATACCCCAGCAACTACACCGAGGCAATCGCCAATAACAACACCTCCCAAAGTAGAGGTTATCATACCACCCAACATTCATGATCCACTACATCTTTTGGATCCCGTTG

>BPA_11376

CCACCATCTGCCCCCACAGTGAGTTCTTGTTGATAGAAAGGCAAAATCTCACCCTCTTTAGTGACCTTTTTCCTTATTATTTGTGCCCTCACATGTGCCTCTATTATATGCGATTTACGCATATCACCCACTCGAAACATTAAACATGGTACACCATCCCGATGACATATCACCGCATTGCGGGAGAATAATAAAGTTTGAGCACGTTTTTT

>BPA_11383

ACGCTCTAGTTAGAGGTATAAATAATAAGAAAAAAGAAATTAACAACTCAGCTGATATTGCTGTTGGACGTCGTATTGCCAACGAAGAAGATAACAACAAGGACGTAGAAGGTGAAACAAATAACGACGGCGACGACGAAGATAGTGATCTACCACAATCAGAGATCGAAGTTAGTTCTTTACCATCACCATTGTTTGATTTAGATGACGAAGCAAAAGAAGAACTAGCTGATTTAAGTATGATGTTACATTCCTCAGCTCCGTTTGCTCCGC

>BPA_11393

GTTTTGAAAGAACTTTGGCTGGCACACAATGATTTAGATTGTAATGATGCCGATACAATTGCAGCAATGTTGAAAAGCAATCATTACATAGAACTAATCGATATAAGCAATAATAATATAAGAGACGAAGGTTTACGGCATATAGTTAAAGCTTTGATTATGCAATCAAAAGAGTTAGAGAGAAGAACAACTTTTCAGCGAACAGCTG

>BPA_11394

GTGATATCGGACTGCAATTACCAAAGTAAGCATCGGCTGATGAGATGATTGTTGTAATTGCTTAGCTGTTGATGTTGTTGTTGTTGCAGCATTTGTGATGAAAATGGTCCTGTATCATTTTGTTGTTCTTAGCAGTAGCAGCATGGAGCAACAATGTATTTCGACTAGGAACAGGTCCTGTGGTGACAACAGTAGCACCTTAACTATATAGACACGAGATGCAAACGGTTCTGGCAAAC

>BPA_11417

GATTTTAAATATTGAGCACTTGGTGAAGTGGTAGGTAGAGGTGCCATGGGCTTATTGTCTTCATAATCAGCTGAATCTCCTTCACTTAAACGACGTTGCAGAGCTCGTTGCTTACGAGCACGTCTCTTTTGATTCTTTTTACGTTGTCCCAAGATACTGGAATCTAATCTAAACACCTGTTTACGTATGAACTCACTGAAACGTACAGTCTTTTTACAGCTTTCCGATAGGCCTACAACATTTTCTTCCGGTATATCACTGAATGACTGTGAAAA

>BPA_11424

ATTAAAAGAAAAACAACAAGAACAACAAATAAAAGTATTTGAACATCCCTTCATTGCCAGTATAACAGACCTACTTATGAAAAGTAAAGCTTTAGGTGAGAAAAATCATCAAAAACGTCATCAATTAAATAATGTACGTGAATTACAAAAAAAATTTCAAGCTGCCCTAGATGAGAACAAAGAAAATCAAACGTATTCTACAAT

>BPA_11426

TTTGTTCTTGTTCAAGCAGCCAATATCTTGGAATCATCAAACACTGTACAGTAGTTCGAGCCATTATAACACGATGTTCAAATTTTTCACCAAGTCCAAAAATTCCACCAAACGTTAAAGAACCCACATCAATAAAACGATTTTCAATTTTATCTTTAACATCATCGAAATCTTTAGATGGTCTCTCATGCGTTTCATTGTCAAAATCTATGTTGGCACCAAATTTATTAACTGTATTCATCTTTAAGATTTTTGACATTTTATTGTCGGAAAAATATTGAAATATTGAATCAGATGCCT

>BPA_11450

ATTCTGCTAAACAATTCAGTATGAAAGTAGGCGTTTATCCGTCCTTCAACTCATTTCCCCATTCCTTGGCAATCATATAAATAGCATGCTGATTGAGTTTAGGTCCAATTAGTGGATTCATCTGAGCCATGTAACAACAGAGCCAGAAGAGCCAACACGTAGCTCCCGTTAGTATTAATGTACAACGTATGAGATTTTGATGAGGTCCCTTTGGTGTCATCAATGGTAGACCAATTC

>BPA_11462

CTTAATCTAAATTCCTTAAATATAGTTACGGAAAATGGGTTGCCTTTACCATCGGCGCAACAACTTTTGGAGCCAATATATTCTAATTTACTTATTCCTTTCCCCAGTAATTATTATCCCACGTATGCCTTAATAAAATCTCTGCAATCATTTGACTTTGCATCGACGCTATTGGAATTACACACACATTTTCATTGCAACGGTGTTGACTGCGGTAAATATGCAG

>BPA_11470

ATTTAATATATAGACATTTTGATATGTATTTGCAGGAGGTCCTTCAGGTTGAGAATATTGCTGTTGATGCGTGTAATAAAATTGTGGTTGATCTTGTTGATATTGAGGTTCTTGGAGTTCCTGTTGTTGGTACGGTTGATGTTCTTGTTGTTGATATTGTGTTTGATGCTGATACTGCTGTTGATCCTGCTGTTCTCCATTCTTTTGTGATGTATAATATTGCGGCGC

>BPA_11474

GTGTCCAAAAATTTATCAAACGACTCGACTGTGGTTATATTGTAATTATGTGTTTCTATTGTGACATCAGGATTGATAAAGGTTAAAGTTTTTGCAGCTGCCTCCACTTTAGACAGACCCGCCTGATCTGGGGTAAAAAACAAACGATTCATATTGGCCAACTCAACTTTGTCATAATCGAAAAGTATTAACTTCCCCACACCACAACGTGTTAACATATCAGCCGTGACACTGCCTACACCACCAACACCCACAACAGCAA

>BPA_11476

TGTTACAACCTCAGGGGCCATATAGTGCGGACAGCCAACGCGTCCATGTGTTTCTAAAGCTTCTCTACCACCGGGCAATTGTACTGCTGAACCAAATCCACCCAACTTGACAGGTGCTGAATTATCTACTGTTGCTAAAAGTGCACAAGCTGGTCTAACATCACGGTGTAAAATATCATTTTCATGACAGTACCGCAAAGCTTCCAATATCTGACGCATATAGTGACAGGCAACAGCTTCACTGTACACAAAACCCGCCACTGCTCTTCG

>BPA_11477

AATTGCATGAAATGCTTGTGCATAAGTTCTTCGGTAATCACATTAGGGGGAAAGCCTCCACAATAGACTGTTGTATTCATAGGACTTGACTGATTGTAGACCTCTTCGTAAGTTTGTCTTGTGTTTGTTTTTACTCCACCACCGCCGCCACTGGCATTTGTTTTACTGCTTTCACGAGGTGGCGCCAACTTACGTGTCGACCAATTGGTACGTATTGATCGTGAACCAATC

>BPA_11493

AACAGTTTCGGGTATAGAACTTTTAATGGATTTTAAGCTGTGTGATATGGACAGACCACCAGATTTGGAGGATTTAGAACGTGATGAAGAAGAGGAGGAGGATGAAGAAGATGAGGATGAACTGGAGCTAGAATCAGCTGAACTGGCATGTACCGGTTTAGTAGCTATCCAAAATTTCAAAGCGAATAAAGTTTCCACTTTAAG

>BPA_11499

ATCCATATCCTTGGGATCTGGCCTGGCTGGTTTTGTTTCCGGATTGGGGTCAATTTCTCCGGGCTTCAATTTGCGCGGATCGTCAGTGGCATCTTCACCATCTTCTTTACGCTGAGCTTGATCTAATAAGTATTCATAACGTTCTAAGCACTGAGCCGCTGTACGGCCTATAATTGGTGCTATGGTACGCCATTGGGTTGGCATAAGTTTGGCCAAATGCAAAAGCTTCTCATCTTCTTCACGCGACCATTCGGTTTTCTTAATA

>BPA_11503

CTACACCATCAACAACACACTCCATTAATAAATGGTTATACAAGTATAGAGTCTTTAGGAAAATTGACAACAGCTGACGAATCCCGACATATTAATATTCCAACAACACAAAGCATCACAAGTACACCAGTATCTAATAATATGCCATTGAATAATATACCAACAACAAATGTCTCAACAAATATATTATCATCACTACAAACATCACCAGTCATCACATATCTAAATGAATCTCCAAGTACTGAGTACAACAAGGACATCATTCAATCAAATGGAACCACTGTCTTAGC

>BPA_11504

CGATAGTAGTGGAAATGTGATCGTATCACATGAGCAACAGCATTTCGAAAGAAACGGTAGAAATGAAATTAATGGCAGCTGCAATAATAATTTAATGACAATATCATCATCAAGTTTACAACAAGCCACAACATCTTCTTCATCTTCATCTGCCTCATCACCATTGACATCGACTACTTTATCTTCGTCATCCTCCTCATCATCAA

>BPA_11514

CTAGTGGAACTAAGTGAACAAAAGGCTTTGCAGTTAGATGAAACTGTTAATCAGAATACGGTTTTACAAAATAAAATCACACAATTGGAAAACACACTTAGTGCACGCGAACATGAACTTGCATTATTCGATACTAAATACCGCAAATGTGTAGAAAGAGCTAAGGAGATTATTAAGAATCTCGATCCACGCATATCGAATGTGGTCG

>BPA_11540

GGTGGTTATACAACTTTATGTCGATCCCTTAATACGTGTCATGACTGTGGGCATTGCGGTACTCATCGGTTATGTTTGCATTAAATGTTTAGGGGAAAATACAGTTGTAGCCTATCAACGTGCCAAAGTAAGCAGCATTAATATAGTCACAAAATCTGAAATGGAAAAATTACACACGATCTGTGAGGATGTCAGTACGGCGGGATCCAATAGCTCCTCATAAAATGCAATAAAATACTAAAAAAC

>BPA_11542

TCGAACTGCTGACCACACATTGGACAAAAATGGGAGGTGGCTACAATGGCGGGATCCATACTTAGATTCGGTAAAAATGATGTCTTCAGTGCGTCCATGGTGGCGGGCGACTGGGCTCGACTATGCTCGGGTCGTGGTAAGCGTATTTCCTGGGCATTGGAAGGAGATTTAAGCATGACGTCTCACAACTTTCAACAAAT

>BPA_11547

CCAATGAGAATATCTCAACAGCTGTTAGTCTAGTACCACCAGCAGTCTTAGTAAAACCATCTTCAAATATTGTCAGCAACAATAAAAGACCGGAGAATAAATTTCCGCAAACAATTGCAGTGGTAAAAGGCAAGAAATACGTTATGGTGGCTAAACCTTTGGCATTACCCACCGAACATGTTAATGATATTGAAAACGATTTGAA

>BPA_11552

ATGCTGCCCAACTTTATACTTTAGGAGAATCTATTGATAATTTAAGCATTTACATAGCTTTAACAGGTCTCCAACATGCGGGATCTCTTATGGGAGATACTATACGCATGCAACGTAATCCTCTTTTCAATCCTGGTCATATATTCTCTAAATTTATGGAAAGATCTAATATTGATAATCCCCGAACAGAAATGAATCTTAAGGAAAATGTTCATCCCACCTTAGAGCCAGCTGCCTTGTGTCTTGAACATTCTGTGGCTCGAT

>BPA_11561

AGAAACTCAACAACAAAGGATAGCAAATACTGGCCCTCCTGGGCCTGCAAGTGCTGGTAATACTCGTCGTGGTTGCGTACAAAAAATAACACCAATTCGTCCCGTTCCTGGCGCCAACCAATCGGCGTCAGCTGGCCCGACAAGTACTGCCCCAACGGCAGCTTATCGTAACACTCAAAGTCACTCGACTGCAGCTCAACCACGTATATTGAATAAAAATGCAGCGCC

>BPA_11571

CTCCATTTATCAAATTTATATGCTTTTCGAATTCTTCTGTAATAAAATCATTGACTTCCTGCAAAGCTTCTATGGGTGTATTATAAAAGTCTTCTATATGTTTATGTGTTGACTCCAAACTATTTATTATTTCTGTATTATCAATTAACAAGGAATGTAATTTCTTTTCAGGGACCTTAATTAGTTTATGTTGTTTTTGCAAATGCATACGAAATTGTTGTAATTGATTTTCAATGT

>BPA_11599

GAATTAAGAGCTTTAGCTGAGTTGGTGGGTCCCTATGGTATTAAAACATTGAACGAAACTCTTATGTGGCATATAGCTAATCAAGTACAAGAGTTGAAATCTCTTGTAGGAGTAAATAAAGAAGTTTTGATTACTTTACGCACCAGTTTCGATAAACCAGAAGTGATGAAAGAACAATTTAAACGTTTACAAGATGTAGATAGAGTATTACAAAGAATGACCATAATTGGTGTCATTTTATGTTTCAGAAATTTGGTACATGAAGCTTTGGTAGATGTTTTAGAAAAACGTATACCTTTCCTATTGAGTTCGGTGAAAGATTTCCAAGAACATTTACCGGGAGGTGATCAAATACGTGTGGCTTCTGAAATGGCAGCAGCTGCTGGTCTCATGTGTAAAGTTGATCCTACATTGGCTGCAACTTTAAAGTCGAAGAAACCAGAATTTGATGAAGGAGAACATTTAATAGCTTGTCTATTAATGGTATTTGTTGCCG

>BPA_11604

CAAACCAACTTGTGGTATAAATGGTTCTCTGGGATTATATTTAGTACCGTAAGGTTTTGTTGGCTTTACGGTTATCAAAAAACACACATCATGTTTGCGCAAATTTTCCCATTCTTCTTTTATCTCCCTACGCACCGATAAGGTGACTCCAATATCTGCTCTTACTCTAGAAGGTTTCTTTTCTCCTATATGTGGCTTAGCCACTTCTACAACGGCAAATGAGGC

>BPA_11627

TATCGAATTCTGAATCCGAATCTGAACAAGTTGGCGACACCATCAAGGGATGATTATCAGCCAAAGTGCGTGCAATGTTACTACGTTTCTGCTGCTGTTGTTGTTGATGAGTTTGGGAATCATCAGTCGATTCAATTGAAAGATTGTGAAAAGGTTTTAAAATGTGGTTGTTATCTAAGGATATTTTATTATTCAAAACTTCGTTGTCATTGTACATCATCTCTAGAGCACTCGAATTGGTATTTGTTTTATCCTCCACAAGTAATTTGTTATTGTGATGATTGTTGCAATT

>BPA_11631

GAATTTTTAAAAGCTATTCGCATTCTTCAAGACGGTTCTACAAGTGATGAAGAAAAGTGTGATGCACTGGACATAATTCGAGATTACATAGATAATATTGATTTTGCAAACAGTTTTGTTAAAATAGAAGGAGCAGATATTCTAATAAATTGCGCAAAACATTCTAGTAAATTGGTTTGCCAAAGTGCGCTCTCCATAATT

>BPA_11655

AGTGGCACGGTGCCAATAACCACAGGCGCTGAAATGTCCGTATCATAGTGAAAAGCACCCGTTTTCAGTTTTACTTGCAGTGTATAATGTATGAAAACTATATAGTTGAGATTTAAAGTTGACCTTGGTGTATCGGTTGGCACATACACATTACCCTCATACAAACGTTTGGTCAAGCGCAATGTTCGCTCTTCGATTGAAC

>BPA_11657

GAGAGATATCAAATATCGAAAATGTGAGTAATTTAAGTTTATGCTCTCATAAAGTGGAAAGTAATGAAGGTGATATTGTTGTCATTAATGAGAAAAGATTTCAGCGTCAGCTGGTTTTAAAGAAATGGGAGGAATTCAATGAGAAAATAAAATCCTCTGATTATTATGTTCAGCTGCGTAGACGTCAGTTTGGCTTTTATTA

>BPA_11661

ACCGGGTGTATAAGTGGGTTTGGTAAACAGTAGAATAGTGTCGGCATAGGGAGATGTTGATTGTGTTGAGTCTGAATCTTCTTCTGCTTCTACATCCTCTCCAGTTACAGCACCATCTTCACCATCTGCTTCAACAATGTCTTCCTCAACGGCATCTTCAGCAAAAGCTGAGAATTTGTTGGGTGCATTAAAAGTGCAAATAATCGAATGAGACCTTGGAGG

>BPA_11665

GGGTACCTCGTAAAGATTTTCATCTGCTAAAACCATTGCCTGCCATTTGTCTTTAGTAGCATCAAAAGAAATGATGGCTGCATTTTGCCATTTGTATTGCAATAAACTTTGTTGTACTGGTAAATAAGCCTTGGCTGGTAAGGGATACCTTTCGCCTCCTTCCACCTCCCCCAATTTTAACCATGATTCTGAAGAATGGCAATCATAA

>BPA_11671

CCAGTCCATCCTTGTTGACAAATACAGGTATAGGCCATTTCGGTTTGACTTTCCAAACAAATGCCACCATTTTGACAGGGACTATCTTGACAAGGTTGACATGGTGTAATGCCATCCTTAACCTTAGCATCCTTCATCAACTCAATAATGGATCCTTGCAATGTCAAACGGCTAACGCAACCCACAAAACCAACAGTTTGATCTACAGCATCTTTAGGTAAA

>BPA_11680

GTTCAACAGGCACATCAACAGGGTCTTCAACTTAATCCAATAAATCAATTGCGCCATAATCATCAACATAGCAGTACCAGTACATTGGTATCTATAGGGGGTCAATCTAAACATCAAAACCATCCCCAACACTTGGCTCAACAGCAACACCAACAACAACAACAACAGCCACAACAATTATTAAACGATAAGACTATTCCAAAAAACGGCGTTAACTCGGGGTCGATTTTTCACTGGCATACGCTGCTGCCGC

>BPA_11696

TTCCAATAACACTAAATTCACTACTATGAACGTGAATTCTAGTGATACTGAATATTATGAATCCACTATTATGAATTTGAAATTTAGTAATAATGAATACGATGATTTTAACACTATCAATGTAAATTCCAAAGATAATGAATACATTGAGACTTCCACCATGATGGTGAATACTACAACTTTAAATTCCAATGATGTAGGCTATATTGAAATGC

>BPA_11703

GGAAAATTGAATTTGGAAGAATTTGAAAATCTTTGTTTGGATCTTAAATCAAAAGATGTGGCCAGTACCTTTAAGACAGTGGTATCTAAGAAAGAAAATCTCGAAACATTGGGCGGTATGTCTAGTATCTCCTCAGAAGGTACCACACATTCCGTACGTCTCGAAGAACAATTGGCCTTTTCAGATTGGATTAATTCCAATTTGGGTCATGATCAAGATTTAAAACACTTGTTGCCCATTGATAATGAGGGCAAGCGTTTATATCAATCCATTAAGGATGGTATTTTATTGTGTAAAATCATTAATCATTCCTGCCCCGACACCATTGACGAAAGAGCCA

>BPA_11713

GTTTTCCTCAGGAGGCTTGCAAAAAAGCAGTATTTCATACAAAAAACGCTGGATTGGATGTTGCTTCCAACTGGCTAATGGAACACATAGGCGATGCAGATTTTTCCGATCCATTTGTAATTCCTAATGAGAATACAGTTGGTGGAGTTGGAGCTGCATTCGTTGCCAATCCCGAAAGTCTGGCCATGTTAATGAGCTTGGGCTTCGATGAACGTCAAGCTACAAAAGCCCTT

>BPA_11730

CTCTCTATAAAGGTACCATCTGGTAGGACTTGTTGATTTTTCATCAACATATTCATACGAGGAACACGAACATTTTTCAAACCCAAAAATCCATTATTTATACCATTAATACCGATCTTTTTACCAATCTCACCAATATGTATACCCGGCAAAGGCATATGGGTCTCTTCATCACGAACTTGCAGCACAAACATTTGAATGCCTTTGCTGTCGCCATCAATGAACAATTGAGCCACAACAATACAATAATTACAAATGTG

>BPA_11740

GCTGGAGTTCTTGAAGAAGAAGTACCAGTTCCATTCGAACTGATGCCAATCCACCACCACTTGCACCATGTAAAGAACAATAACTTAGTAAAGTTCGTAAAAGCGTTTCATTTGCTTTCAACCAACGCTTTCGCTTAGCTGCTCTCATGACTTTAATTTCAAAATCATGTTTTTCATGCATCAATATTTCATGTAGAGTAGGTTTCTCTGACAAAATATAAGAAGTAGACATGTCTTTTTCCTTAATAACATCAGTACTATTTAAGTCAGTAGGATCAGAATTACAGTAATTGCACA

>BPA_11742

GAGTTGCATTTATTTACGTTTAATTTTCAGAGATATTCAATCGTTACGTTTAAATGAACCCAAAGAGATCAAAGATTTAAGACGTGAAATACGTGTTTGGCAAAAGGCCGCCAATTCAATTTCTTCATTTTCCAAAGATGCCGATTTAGTACGCGAAACGTTGCTCAAAAAAGTTCGTATTTTAAAACATGCCCTCAAGCGTAAATTAA

>BPA_11745

GAATTATGCCGTCAAACAGCCGAAGATGGAGTCTCATTGGTGGCATTAGAAAAATCGAATGGTAAACCGGTAGCTGTACTTTTTAATAAACTTCTATTTTTACCTTCTCCCGGTGAAAAAGATTTCTATACAAAATTCCGTGAGGAAAATACTCATTCACCACAAGCTCAAGCTTTAATGGATTTCATGTTATATGTCGAGGAGTTGCATGATGTTTTTGAGAAATTCCATTTAAATTGTATTTATGAAATGTTATTTATGTCCACTTCACCCGAATGGGGACGCCAGG

>BPA_11747

GACGCAATGGCTTTAGTTATTTACACTCTATTAATTCTTCAAATAATAACAGTAACAATATCACCATTAATGGTACATGTTTTACCACCGAAAAAGTCCTCAACAATTTCTGTGAGTCGCACTGGCAGTGTGGTGCCAGAAATTTTCAAGAAAATGAAACGCATGTATGATGGGGATGATGTTGAAAGATGCAACGAAATTCCTGTGGTTTTTGGTTTGGGAACGGTATA

>BPA_11755

CCACATTTGGTGAAGTAGATTCAGCTTGGAATGTAACGGATGAAAGTGTAGTATTACAGCAACAAACAGCACAATTATGCAGTAGCAATATATATAATAATCAAGGTGACGCGGGGAGTGATAATGAAGCATTGGCGTCTCCCATTCCTAGAGTGGTCGTAAGTCCACGTCATGAAGCTCATTCCACAAGCAACAGATCTCCATTCCACAGTGCAGCAACATTTAACAAAAC

>BPA_11765

CTCTTTCGAAGCCTTCATTAGGATACATTTTATATTAAACATCATGTTCACGAAATTGGTCATTTTGTCTTTGGCTGCTTTGGCTTGGGCCAAACCACAAGCTGCGCAATACCCTGCCGGCGTTAACCCTCAGGACTGTCCCGGCTTCCCCATCTGTGATAATGCTCGTCTCCACAATCCCAACAAATGGCAACAACCTGCCTGGCAACCACAACCCGCATGGCAACCACAACCCCAATGGGGAGCCCCTGCCCCAGCCTGGCAACCAC

>BPA_11774

TCCTGCTTAACTGAAATAATATTCGTTTTGCTCTCAGTGTTTTCAGTTTCTAATTTAGTTGTCTCTTTTATGTTTGAATTTTCATTTAATATAGTTTGCTTGGGACTTTGAACTTCTTGTTTTATTTTTATCTCTTTCAAAGGCGTAATCGATCGACGTTCAGCAATATTTTCAAAAGTGCTACTTCTACGCTTACTGACAATACGCGGTTTAGCCTCTTCTTGTTCTTTTTC

>BPA_11792

TAATTCTTCACCTAAGCCAGCAGTAACAGCAGAGGTTTTATTGACTTCACTGACATTTCCTCCAGCCAAGGCGGTTAATTTTCTCCTTAAAGCTGTTGCCTCAGCTCTGGCTTCCTCATAGCGTCTGTCAGCTTGTCGGGCAGCTAACCACGTTTCATGAGCCCGATTTTCTAAAGTGCCAGTTTGAGCCTTATGAGCTGCTACTTGTGCCTCA

>BPA_11799

ATAAAAGAAGCTGCTTCGGGAGCAGTGGTGGATACTAAAGCCGTTGAGACCACACAGAAACAAGTGGCCATCAAGAAACCTTTGAAGACTCGTATTTGGGAAGAAATTGTGCATTATTATCATGGTTTCCGTTTGTTATTTATTGATATTAATATTTGCCGTAAATTATTATGGCGAGTCCTAAATGGCAAGACTTTAACAAGGAGAG

>BPA_11812

ATGTATGAGCTTTCCTCATGTATGAGCTTGGAATAGCTTTTGTTTCAGTGTATTATTATTCCTATCATATCGTTAATATGTCTGAGAATGTGTTGTCGATACATAGTGACTTCTTCGGAACACTTATTGTTTACACCTCATAACAGGAAATTATATTTTTGTAATTTATTGCGGGGAAGATGTTGCTCTATTCTTAATTGGAGAATATTAAAAATGTTTGTCTGCCACAGTTTACGTTG

>BPA_11841

TAGAAATTAAAGAAGCAGTAGTTGAAGGTATTATAGTATTTGGGGAAGAATTGGAGTTTAAGAAATCTGTTACTTCATCATTGTCACTATCGTCTGCCGCTGCTGTTGAATTAATGCTTTGTACAGCATTGTTGTCCATATTAAAGTAACAAACAAATTTTTATTTGTGTCAAAATTCAAGGTAAATGATTGTTCGTTGATTGGGGAGTTGTTTGTTTCCTGTTTGTTTCTTCTTTTGTTATATTATAATATTCTA

>BPA_11856

GTGATGTTAGCAATCATATACCTATTTGCACCTGTCCTAAAGGTTATACCGGTGATCCATTCATCAATTGCCGTCAAGTAATACGAAATGATATGGTACCAAATAAAGATCCTTGTCAACCAAATCCATGCGGACCCAATAGCATTTGCCAAGTTTCTTCACAAGGTCCCAGATGTGCTTGTCAACCGGGTATGTTGGGCT

>BPA_11864

AGACAAAATATATTTGAAAACAAAGAAAAAAATAAATTTTAAACATCCTTGCAAAATTAAATAAAAGCTAAAAATGCCACGTGGTAAATATGTAAACCATAAAGGACGTAGCCGCCACTTTACCTCTCCTGAAGAGCTACAACAAGAAAGTGAAGAAGAATCGAGCAGCAGTGAAGAGTCATCGGGTGGAGAAGATGAACAGCCAGGTGGCAGTAATAAAGCACGAGGTGCCAAGCCTAAATCTGCTCCTGCTGGTAAAATGCCGACTAGCAGTGAGGAATCCGACGAAGAATCAGAAGATGATCGTGGTGCTAAAAAGGGTGTTGCCGGCCTAATTGAAATTGAAAATCCCAATCGTGCTGTCAAAAAGGCCACACAAAAGGTATCAAAATTAAGTTTAGATCCTGATGCACCTGCACCCAAACCCGAATTATCAAGACGAGAA

>BPA_11867

CAATATCGCAAGACATTGATATTGGAATGTAAAAAACTAACATATTTGGATTCTAGACCTGTATTTCCCAAAGATAGGGCTTGTGCTGAAGCATGGAAACGTGGAGGATATGAAGAAGAACGTCGTGAAAATGAACGTTGGAATCGTAAAGAGCGCAAGAAGATGAGAGACGGCGTAAATGCCACCATTCAATTACGAAATAAATATCG

>BPA_11870

TTCACGTGTTCTTTTCTTCTTTTTCTTTTTAATAGAATGATCATCATTATTGCCACCACTGCCTATATCACCCAAGGAGGTATCACGCTCCATGGATGGCGTACGCACTAATGCATAACGTTTTGATGTTTCTTTCTCTTTTGCCGCTGCGGCGGCTGCCTGTTCTCGTTGAGCGTCCAGTTCTTTTTGTAAAGCCAAACTTTTGGCCAGTTCTTCTTTAGCCTTACGATCTGAATCAAAACTGGAGCCTTCAACAAAA

>BPA_11875

AACAATGACTTGCGGTGGGGTGGTGGTATTGGTGGCATTATTATTGTTATTAACATTATTATGTAAGGCTGGCGATGAATTACCAGTCGAACTTAAAGCTGGACTGGCATAAACGGGATATTGTTGTTGGGTGGTGGTCGATGAAGCAGGACTAGGATGTTGTTGTTCTATTTGCAACACCTGTTGTTGTTGTTGCGGTTGCTGCTGTTGTTGTTGCTGTTGTTG

>BPA_11880

GATACATTTCTCACCATTAGGACATTCCCAAAACCAACCATATTTGGATTTTTCAACGGCCTCAATAAAGTATTTGCAAATAATATCTGTTGTGGGCCTTTTCTTTTCACCAGCATGTTTCTTATCCACCACTTCCTTAAGTTTGGCATCATCCCAATTGCTCATGTCATCCTCGGTTTCATCACGCATATCCACATAAATGGAACGTTTCTCCACTTTATTTTCCACCGCCAAATCGTGCGAGAATTTACATTTGTCTCCTTTGGTGCAA

>BPA_11882

CGCCAATCCCGAAGAACTGCCATTTTCTAGGTTACCATTTATAGTAATACACATGGACTTAATACTCTTCCCTACCGTATTAACACTGCCACCATTTTCCGAGCCCCTTAATCTACCATTTTCTATATCATGCAAAGTGGAATTACTTAAATACAATTCACATACCAAATTCCAAGCGTCCTGCAAACAAAATAAATATCTCACAAAATTCTCCTTTTTCTTAACTTTGGTGGTATTTAAACGCGAACAGGATTCTAAGATGAAATT

>BPA_11886

GCCACTATTATGAACACCTCTGGTATAAATCATTTTATTTGCAGCTGCGGATGCTCTGACACTTCGACGTGTGCCAATTTTTTCATCACTAGGGCTGCTGCCGGTGTTGCTAATACTACTATGATTTATGGTTGCGTTAGGTGTTGCTGCATTAGAATTTGTGGCAGAATGACTTGCTCCGCGTGTAGCATGTGTTGGTGTAGCAGTTTCAGTTATGTTTGATTTCGGCGTTAAATTCGATGCCAAGACAGATGAGCTATTGGAACCCGTAGTAGTT

>BPA_11897

CTGCTAAGAGTTTTTCATATTGTCGGTATAATTGTTGAGCATTTGCTGCTGTTGCATGTTTTAAATATGAATCTGAGACATTTAATACATCTCTGGGTAATATACCAAAGTTATTCATAGGTGTAGCTATGCCCACTAGAGCAGACATAATAGGGGCCATTTGTGCTTGCTCCATTTCATATAAGGAGGATATATTAAGTGGTGAATTGTAGGATTTG

>BPA_11904

GAAAGAACTGCGTGCCAAAGAGCCGTCACCGCATACAACACAAAGCATTATTAGTTTGCTTATGGGCATGAAATTCTTTAGAGTTAAGATGGTACCCATAGAAGAATTTGAGGCATCATTTCAATTCATGCATGAATGTGGCCAGTATTTTTTAGAGTTAAAAGACAAAGATATAAAACATGCTTTGGCAGGCTTATTTGTAG

>BPA_11905

GCCTGTTTGTTTTGTGCTTCAGTCAATTGCTTCTTCAAATCTAAAAGCTTGTTAACTTCTGGTTGCCATACAGCTTTGTCTTTTGTAGAAGCCTTAAGTTGTCTCACTTTATCACCTTGTTCCTGAATAGCCTTTTCCAAATCAGCTATTGATACATTGCCGATTTGTGAATCTGCGGAATTAACAACCTGAGGACCGGCATATTTCTTTTTCATTTCGT

>BPA_11920

TTTAGAACACTTCTCTGTTGTTGATTTTTTAGTCGCGGAAATTTCATCATTTTCCTCTTTAACCAAATTTTGAATTTCATCTTCGTCGTCTTCAAGCCGTTCTAAAGTTTCTTTTTGCTCATTTTCATGCTCTAACGAACTTGTTATCCATTCGGGTTCCGTAGCCGATTCATCATCCATTTGGGCATCATCTTGCTGCTGGTTCC

>BPA_11924

ACGTGGTTTGGAACACGTTGAGACCCTTGAGGTTCAACAAGATGCTAACATTGCTGCCGTTAAGGCTCAAATTGCCCAACTCCATGGTTACAATACTGAGGAAATCACCTTGAACTGTGAAGGCAATGCTTTAAGCAATGAAACTCTTGTTTCTGCTTTGTCCTCTTCCGAATTGGATATCACCATCCCCATGTTGGGTGGTAAAGTACACGGTTCTTTGGCTCGTGCCGGTAAGGTAAAGGGTCAAACCCCCAAGGTAGACAAGCAAGAAAAGAAAAAGAAGAAGACCGGTCGTGCTAAGAGACGTATCCAATACAACCGTCGTTTCGTCAACACCGTATCTGG

>BPA_11926

AAGGTTCGTCTTCCCTAGATTTTGGAACAACGAATATAATAACGCAGGTGCAACCACCTCCACGGGCTATGCAATTGAAGAAACAGTTTAGCTTGGACCAAGGCAAACAGCAACAACCTCCACCGATTCCACCAACTACACGTATATCATGCAACGAAACGCCTGCCACACTTCCAGACGATTTGGCGCCATCATCATCCTCAATATCTG

>BPA_11927

CGCTGCCTCGGTTACATTAATAACAAAAAGGGGCAAATTCCGTTATAATACCGAACTCACTGGTCCCCGCAATATATGTGAAGCTATCGAACAATTGGGCTTTGAAGCCCATCTACTCTCTGGAAAAGATAAAATGGCCCATAGTTACTTAGAACACAAAGAGGAAATACGTAAATGGCGTACTGCCTTCCTCATCTCTCTAATATTTGGCGG

>BPA_11937

AAATGGAGTCAATGCCATGCAATTGGATCCGAACACTGGTAATCTATATTCGGCCGGCAGAGATGCTATAATACGGGTTTGGAATTCACGTTTGGATTCGAATGAAAAATATATACAATCAATGGAGCACCACAATGACTGGGTCAATGATATTGTCTTATGCTGTAATGGCAGAAATTTAATTAGTGCTAGCTGTGATACTACTGTAAAATTATGGAATGCCCATAAGGGATTTTGTATGTCAA

>BPA_11949

TGCTGATCCGCGATGGGGTGATATCGGACGACTCGACATTCGAGCAGCTGAAGATGATGTAGACGCTGCCGCAGCCCCACGTTGATATTGCAAAGAACGTGACGAAGATGATTTTGGAGGTGGTGATCGTGGACCACCTCTCCCGCGATGTAATGAAAGAGAACGAGATCGATGTCGATGACTAGGTGAGCAAACCGAACACGAATCATCCGAACAACTACTAACCG

>BPA_11959

TGGCAATCTATCATCTACTACCACGTCTATCCATTTGCCATATTGCCAAAAGCAAAAATGAAACACTCCGGCATAATTTTCTTCAAAACTTTGATCTGGTGGCACTACACGGAAGAAAAGACTCGAATCTTGTGTAAGATTAGCGGTAGCAGCCAAAAGCCAACAATCGCCCAATTCACCTTGTTGAACATCAAATCTAGAATAA

>BPA_11966

GGAGGGCCAAGACGACTATTTATTGAACCAGCCCCACTAAAAGCCCCTGATGGTCCCGCCATTATATTCGTGCCTGCCGATGATGGAGGCATTTGAGCACCAGGTGGTAACTGCATACTTGCCGGAGAATTAGCTGTCACTTGCGAAGAAGGATGACCCGGCTTAGGATGTATACCTTGCAAGGGTGATGCATTCGATTGAGAAGATG

>BPA_12002

GTAAATTTGTAAAATTTACTTTTTGGCAGCGCCCTTCTTGATGCCTTGAGCCTTCAATTCACGAATGCGTTCACGTTTGTCTTTCTTGAGTTTACGGACGGTACCATCCTCCTTGGTGCGCTTCTTCAATGCGTTGAAAGCAATGGTGAGCAACTTGTTGCGTTGACGTTTGGCATAACGCAATTTGAAACGATCAAAATCATTCAAACGGGAGCGCTTGCAAATGTTTTG

>BPA_12006

CCACTATTTACTGACCCACCTATTGGTCTATCCTTTACGTCCAAAGACCAAACGCTCGGATCCATTTTGACTGTATCGTCCAGAATTCCACTTATGCTTTCGTTGTCTCCATAGTTCAGATCTACATCGTCCAATTTCATACCTGACAAATTTAATTCGAGGTCCGAGTCCGCTAATCGTTGAGAGCGATTGCTTTTCATTCCGGACAGAGCGCTATATCCACCACCAACCATACCAAGAGCTCCACTGTCCAAAGCATCGCCATCGCCCTCAACATCACCTGTCAAGCG

>BPA_12010

TATGAGAGTGGAAGTCTTCTTAAGATTACCAGTCTTTTGAGTGGGTGTAGAGTCTTTTGGTGTTCCTCCAGTTTTGACACGTATACGTTTATTGCACTCTGAATCAGGTTGCATAGATTTGACACGCTTCATTTTTGCATACAATTCAGGATTTTCATAACGATCTAAATTAGATATGGGATAATCTTTGGGATCACATATACGACGTTGATTTTTACAGGCTTCAATCCATAAAATTGAAACGATTGGAATATTCATTTGTGTAGCCTTTTTAAAAAGAAAATATATCAGATCAA

>BPA_12022

TTTTTTGATCCATTAAATGTGCGTTTGATTTATTGAAAGCTCCTGGTATACAGTTGGCTGCCATTGAAGACATTGTTTTAAAGGAAGGTGGCCCTAAACCAGGATGTGGATGTATACTATTGTGTTGTTTGTAAGCATCATTATTATTTGAGCCGTGTGTTAACAAAGTTGAACTATTAATTGTTGATAATGACGAAGAAGTCGTCGCTATTGTTGTTATTACTACTATTATTATTGTGCA

>BPA_12028

CTGGGTGTTGGCGAAGGCATCTGAGAAGAATGATAATTGCGTGGTGAAGACATTGGCGAACGAGCAGGACTTGGACAACGGGGCGAACCGCCTTGTGGTACACTTAATGTATTACTACGCACAACCAAAGAACTGGCCGAATGATGACCGTTTGAAATGTTTGGCGATATTCCAAAATCACATTCAGCATTTGAATTACGATCTCCA

>BPA_12029

CTGTTGCATTTGCATTTTGTGGTACCACAATGGAATTGAAAGATGGTTTTGAACGTGAGGGGAGAGATATTAAAAACTTGTTGGTTTATCAAATGCGCGACGATAAACCTTTGGCTTTGGGAGCACATTATGAAAAATCTGTTTCCTCTCAATTAACACATCGTAAACCACCAAAATCACCGAATGCTTTATTGACACCAGCACCACTAACACCCAAAAAATCACATTCTCCTTCACGACATTCCAG

>BPA_12033

CCGGGATTGCAGCGACATTTGCCAGATTCACAAACGCCATTTGGACCACAATCTAGACTGCATACAGCTTGTGAACAATCTGGTCCTGTCCAATGCCTTTCGCAAACACACTGACCTGTCTCCAAATCGTAGGTACCATGTTCTGAACAACCGGGTAAACATTGATATACTTGCTGATCAATGGTGCCACAATCTTCACCTTGCCATCCAGCCTTACAATAGCATTGACCCGCTACACAGGTGCCATGACTCGAACATAAAGGATCTAAACAATCATGTTGATCACAAAAAGGTCCCTTCC

>BPA_12037

ATCCGATGAAATTAAACAAAAACATTTAATTGAACTTGAAGAAGCTAATCAAGAGAGTGATAATCCACTAACAGAAGAATTTGACAGTGAAAAATTTACCCAAGAATCTAAAGAGAAAACTGAAGAAAGTATTCCCAGTGGAAGTTTTGACAAAAATCAGCAAGGGGTATCAACTCAATCAAACATTCTTTCTAAAGAAACTGA

>BPA_12050

TGCAAAATACAAATATGTATCAATCACCACCTAACCAATCTTCAGTTACAACGCCGCAACATTCACCAACAAAAGCAACATCAACAGGTGCTTCAACCGTTTCAATGACAGTCACAGCTTCTCCGTATTCTGCAGCAAATGAACAAAGGAAGCAACAAGGTTTATACCTTTTGCATGTGAGGCTACCTCCTGGATATACACCTAATACAACTATTAATAAGC

>BPA_12054

GCGGACTTTATGCCCAATCATGCAACTTCTGATATGTACATCGATGAAGGAGAGACATTATTACGTGAAACCGAGCATTTATTAAAGAATGTTGGTAGCGATGCCAAACTCGCTTTGACGGAAGCCAAAACTGAATTGACCCATTGTGTAGATGAGTTGCGAGAAGTGTTTCTAGGACTGCGTGTTTCATATCGCATTTTAAAAATTGATGATTTGTTTCAATGTGTAGAGGAAGCTAATGCCACTAAAGACTATTTGATTGTTTTGGATTTATTGGGGAA

>BPA_12066

GAACTGATGCAACGTTGCTGGGAAGTAATCGATGCTCAAGCCGAAATGGCCATTAAATCCGAAGATTTTGTTGATATCGATTTAAAAACATTCGAATCAATTTTATGGCGCGAAACTTTGAATTGCAAAGAGATTCATCTCTTTGAGGCCGCATTAAATTGGGCTCAAAATTGTTGTCAAAAAATGTCCATTGACAATACACCACAAAATATGCGTAAAGTTT

>BPA_12070

TTTTGGTGTAGAAGGTAAATGATTTTCACGATCTTCATATATTTGTTGTCGCGATAGACGACGTTTTGGTTTACGATGTGTAGCAGCTTTAGGAGAGTTTTTCTTACCAGCTCTAACACGATCCAAATGTTTATTCGTAGGTTTGTTTACCACGTGCAAAACGTTGTTATCATTAAACTTTACATTAGCATTTTGTGTGGCCATAGATAC

>BPA_12075

AGGGTCCTGAAGATGTTATTAACCGTTTATGATGCAAAGCTGAACTCATGCCCGAGGAAGCGGACGATTCAGAAGATAATAAAGAAACAGTGTCACTAAAAGCTGAATCATTATCGGGAGATTCAGTACGTGGCTCTCTCAAACTGGGCACACCACCGCCACCTATTCCTCCCCCGCCTCCTCCGCCATTTAGTTCCCCCCTTTGCATATGCTGCTGAAGATGATGTTGT

>BPA_12091

AGATCTTACAACCAAATTGATAATGCAAGTTCTGATGATGGAAGTGAAACTAAAGCTAAGAAATCCAAAACTGTATGTGATGAAGAGCCTAGTAAACGAATTACGAAACCAGTAAAACGTTTTGATGACACGAATTTAGAAATTGCAAGTACTAATCTGAAGCAGAGGGAAGTCATTGTAATGAAGCCTGTGCCTTGGATTCGTTTAAATGGATCCATCAATCGACGAGTCTTAGACAGATGGTTGGGATCTATACTCTCTGAATGTATCGCCCGTAATGGTTGTAGTAGTCACGATATATGCAAAAGATTTTCAC

>BPA_12102

GAACTCCCCGTTCAGTGAAACCCGCCATACTGGCCTCTTGAGCAAATTGATAACGTTGTGTCATAATACGTTGATGTTGATGATGATGTGTTTGAGCACCAGCAGCTGTTGCATATTGTGCAGCATGTCCACGTCCTGCCACAACATGTAACTGCATGCCCGGCAAAGCATAACCAATTGGAACACGTATAGTTTGTTGAGTTACTTGTGTCAT

>BPA_12113

CTCGGACTGATCATCTATTCGACGTATATTATCCATTACTATACCCAACCAGGGTACATATAATAAAGCAATCCTTGACAGTTGACCTCGATTTTGATAACGATCATCCAATTCATGTTTGGCTAAAAGATTTTTCAACACCGTCAAAGCGTGCCTGCGAACAACACTCACTTCATTTAAACTACTTTTCAGTTCTTGTAGCAATAAGCCCGACAAGAA

>BPA_12120

GCCACAGCTCCATTTGTATGGCACAGTCCAATTGGAATAGCCTGGTATCAAGGCACAATTGTTGAGTTTCCAATTTAGTAATTGACACACCGGTTGTTTGATTGGTGCTCTTACAAATATCTTCTAAATGTTTGCGCAATTTATCGCACAAACGACGGAATTCACTCTTACGATTGTATTTCAAGCAGAAATTGAAAGCCATACGAGCAATGTCATGATACAAAGCCTCACAATGAGTGTTAATACGCAACAACTCCAAACACTGACAGTAAGATTCCCACAAGAATTTTACCCATGGCAATAAAATGGTGCGATCCGAACGATCTTGAGCATCTTCACCACACACAGCACTCATTAAAATACTTTCTGGTGTAGCAATATT

>BPA_12142

TATTCTCTATAGAGGTATTGGAGTGGACTTCCACAAAAGTAAATCGAGTTGTTGTGGCCATTTCTAAAGTTTCGGATGATGATCGGGAGAGTTCGCTACTTGGAACAATGTTGGGATTCTTTATAATCGATATTTCAGGTGAAAATGTTTCTTGGGATTCGGTTGGTAGGTCCCTCAAGTTTTCTACAAGAGATTTATCCATTTGAGAGTTCTTAGGAGAAATGGAAGCTTCTTTTTCTCTCTCCACATCGCTAAATTCATAATGCTCCGAAATGGTTTCAATATTCTTAAGATCCTTAAGAGATTCTGAATTGTTTAAGGAGCTTTTATTAAATCCAGTGTCTTCCTCATAAAAACCTGGATTTCCTAAACTGTTATCCAAAGTAAATTCTCCCAACGCTGTTGGAAATTCCACATATTGTTTATCCGATAAATTTTGTTTTCTCGAACGATGACCACTCTCTGTATAAGAAATTGAAGACCCTGAAGCTTCTGTACTTGAAGCATCTGGAGATTTAGATTCATTGGACTTAGAGGCCTTAGGTTTATCACTAGATTTAAGATTTTCT

>BPA_12143

CAAACGTTCTCTTTCCATTTGAGATATGGCCGCTGGATTTGCATACATAGGAAATTGTGAAGGATGTAAGCCCCTTCGATAGTATTCCATCCAATGAGGATCTAAACGTGATTGACCCGCTGCAGCAGCTTGTGCGTTTTTAAGTTCCTCCAATTCTCTTTCTCTACTATACATGGGCCCCATTGGATGATGATATGGTACCATCTG

>BPA_12147

GAATCTAATAGTGATGAAGAAAACAATCATTCAAACTCAATACCTAAAGAGCCGACGACGGCGGCGACGGCGCCACCAAGAACGACGCCGGAATCCGTAAAAAATAACAGTGATTCTGATGAAGAGGAAGAATATGCTTCTGATTCAGGTGGATCTTCAAAATCTTCAAATTCTTCTGCTCAATCCGCATCTGAATCAAGTAGCA

>BPA_12161

CACGTAGTATAGTGGGTGGTAGACCACGCCACAGACCCAATATGCCTTGTGTTTGTACCACTGTCCGTACAGCTGAAGTTATTTGAGCATAAGTCATTTTTTCCGACTGCATTTTTGTGCGTATCAATTCGACGGGACTAACAAAAGTGACGGCACAAACGCGAGCGGTTACACCGGCTAGCAAAGGTATAAGAAATGGAATATCACGA

>BPA_12181

TTGATCCAAAAACAGTGAAATGTATAATCGATTTTAAAGACATGCTTGATGGTGTCTTCAATCCACCAGCAAAAGGTGCAAATGATGATTTGGATACTGAAGATGAAGATGATGAACCAGAAACAAATTCCTCTGAGAATAATGTGGAGACTGAAATAGTTAATATTGTGGACGACGTAAATATTGAAGCCATTACAAATTCAAGTACACCACATGACAAAGAAAAATCAGAAGAAACACTTAATTTAC

>BPA_12187

ACCATCAGTACCATCGGGACCACGATCACCACGACTACCCTTAGTACCTTTAGAATTTATACCACCTTCACCAGCATCACCAGGTGGACCTGCAGGACCAGGTTTACCAATCTCACCACGACCACCATGACGACCTGGCAAACCGGGAACACCTTGTTGACCGGTCTTACCATCACAGCCATCAATACCACGAGGACCTTGGGGACCATCTTCACCGGGACGACCACGTGGACCAGGAGCTC

>BPA_12196

ATTTAAGGAACGTATTCGTATGTATCCATCATTGATAAACTGTTGCACAATCGATTGGTACATGCCATGGCCGGTAGACGCTTTAGAACGTGTTGCGGAATATTTTATTAGCTCAATGAAATTGGGTCAATCACAAGAAACAGAAGATGCTGATGCCAATAAGTCACTTGAATCATATGAATCGAAAGAAAGTAGAAAAAG

>BPA_12198

TTATAAGATCATTTCCAAATTTTGTCCATGATGGTGGAGATTGTTTACGCATTGGTACTAGACTTGTTTGTGACTTTTGTTGTTGTGCAACTACAACTGCATATGTTGGTGGTGACTGACTTAATGGAGTGCCCGACATAGTTGTTGTGGATGTTGCCACTGGTTTTGTTTCCGGCGATGAACTACGCTGAAATATGGTAGTTG

>BPA_12199

TTTGAAACGATGATTGTATTGCTGACAAAACAGGACGATAAATATTCATTGAAATATTTACATGCAGCCCGTAATACAGATGTTAATAATAAACCAGTTAGTCCCATTATGAGTATCGATTCGGAAACATTGGTGCGTCAGGAAGCCGCAGATAAAAATTCATTTTTCTTAATTAAAACAAAAACATCACAAATGTTGGAATTACGTGCACCAAGTAGTTCAGAA

>BPA_12201

ATTTTGAATCTAATGAAATTAGAGCTTTCTGATGTCGTATGGGTGGTCTAGGAGAAAGAGAACGTGTTGACCGGAGCAATTTTTGTGATTTAGATTCTTTTAAAGAGTCGTGTTTCTTCATTCCGTTTTTACGATTTTCATTACCGTCAATATTTCCAAAATTTTCGGTTTGCAATTGGAATGTACTATTTTTGCTTTTGGTCGATTTCAAGCAAATATCATTTGTTAGCGATTGCGAAGGAGTAAGCCTTCCCAATTT

>BPA_12205

TTCTTTTTAACAACCTTTGCGGTTTCCTTAACTTCTTCTTCCTCTTCTTCGACAGGCACAATTTCATCTTTCTCAACAACTTCCTCAGGTTCTGGTTGTGGTTCCTCTACTGGTTTTTCAGGTTTCTTCTTACCACGTTGCCACAAAACAGGTTGAGGTTGTTCAACTTTTTCCTCTTTAGGTTCTTCTGGCGACTCATCAACCACTTCTTCGAAA

>BPA_12206

AAGCTTCTTTAAAATCGATGGACTTATCTTCATTATCTCTAACAGTACCACGGTGACTATCAACAATGCCAACCTGTGCAGCTTTTTCAAATGGCAATACCTCGCCTGCTACATTCACAATGGTCGAGTCCGGATCCAAAATTCCTCTACGAATAGCACGTTCAGTTGAGAGTTTCTCACGAGTAATAGTGTTACTGAATTCCCCAGTTTTAGGATCATAAAGACCACGGTACACAGCATCTGGTAAGCTTCGTTTTCGTT

>BPA_12221

GAGATATTCAATTCGAAGACTATGACGTCTTCTCCTACTTCTCCAAGTGGTGGAAATTTTGTGAAACCTTTAACTCCTACTATGACCACAAAGAAAATACAAATGCCCTACATTAATGCGCCAAAGACTACGCAATCCAACAATATTGTATTACCGACACAACATAATATGACACAGCAACAAGATGCAACCACAAAATCATTTACAGTTATTGAACCGTTGTCAACAACAACTGCAACAACTGCAACAACAACAGCAGCAGCAGAAACAACTGCAGCAGCTGCAGCTG

>BPA_12227

CTCGGGTGTCACTTGAGTCGGTGCCGTGTGATCACATTCGATAATTCCTTGAGGTGTTGAAGGTTCACTCTCAATAATTAAATCTTTTACCTGAGGTGTTGTGGGTGTTGGTGCTGGATTTTCATTATCCTCCTCAACATAACTTTCTTGTGGCGTACAAGGCATAATAATGTTATTATCAAATTCACCGCATACATCTT

>BPA_12241

AAATCAACGTGGTCACGGTCCCAATCAGTAACGACTGGTTTACCGGGTGCGTCGGGTTCATCAAATTCGTTTTTAGCAATTATACTTCTGGCCGCCTCTAACGGTTCTGATTCACCAATTGCATTTACAGCCTTCACACGGAACAAATATTCTTTACGGTGTGTAAGACGAGTAACATCATGAACAGGATGTGTACTCATACCAGCATCTGACCAGGTACCACGAGATAAGTCCATCTTTTCTATTATATAATGTAAAATTGGCGATCCACCATCATCTTCGGGTACTTG

>BPA_12257

TAGAGCTATAACACGTAAAATTGTTTTTCATGCTGGACCTACAAATTCGGGCAAAACTTATCATGCAATGGAAAGATTTTTAAAAGCAAAATCAGGTGTTTATTGTGGTCCTTTAAAACTATTGGCTACTGAAGTTTTTAATAAAGCAAATGGAAGGGGAACTCCCTGTGATTTAGTAACTGGTGAAGAACGGAAATTTGGCATTAATGAACAAACACCAGCAGCTCATGTGGCCTG

>BPA_12258

TTGGTTCCAAATGTGTCATCACTGAAAAATTCATTGGTTCTGTTTCAAAATCGGGTATAGTGGAGTCTGTATTGTCTTGGGCTAAATTTAAAGCTGAAACCGATATAGCCAAGACGGGTGGTAAGAAATCTACCAAAATCAAGGGTATACCCAAGTTGGAAGATGCCAACGAAGCCGGTACCAAAAACTCTTACATGTGCACTTTGATTCTAACGGAGGGAGACTCAG

>BPA_12268

CACTATACTCGACGTAATTGTGAAATGGAATGTGATGCTCATTTTATGTTGGACGAATGTAATTGTATTCCTTATTACATGCCTCCAATAACCCCAAATACCACTATTTGTAGTATACGACATTTTGATTGTCTGTTAGAGGCCGAGAAAGATTATATCGATCCAAAACGTTTGGCCTGTAAAGATGAATGCTTACCAAG

>BPA_12270

TCACGATTCAAATGTTGACGCATTTCTTTGGTTTCTAATTTCAAATAATGTTGCGGTACTAAAGCTTCAATGACACAACAATGTTGAAAATGTTGTAGAATTGGTGAAAATTGAACTTTTAAAGGATTCATTATACGATCCAGGAGACAAAGTAATTTGGAAAAGTTACAGGTGCGTACGGTACAGATAATAAACACTTTTTGTTTTAATGTCATTTGAAT

>BPA_12277

AAAAACAAGTCTTCGAATTCGTTGTTCATAGAAAGTTCCATTAGACCGGTTTTGTAATTAACACCTAAATTGGTGAGAATTTTAAAGAGATCTGTTAAGGCTTTACGTTTTTGCTGCAATATTTGCTTTGCTTCTAACTTCTGTTTAGGTCGTTCCTTAGATCTATCTACTGTTAAGTTGCGCAAATGATCACATCTCTCTAATTGTTGTGATAGTAAGGAATCCAAAGCAATT

>BPA_12281

AAAGCAAATGGCATTGAAGATCATCGTTTTGTCAAAGAAATGGACTATCAAGAGGTCTTAAATAGTCAAGAGATATTCGGTGATGAATTAGAGATATTCGCCAAAAAGGAATTACAAAAGGAAGTAGTAGTGCCCAAGGCTAAAGGGGAAATATTGGGTGTGGTTATAGTAGAATCTGGTTGGGGTTCTATGTTACCAACAGTAGTCATAGCAAATCTTATGTCAACAGGAGCGGCAGCCAGA

>BPA_12290

TTGGCCCTGGGCAGTGAATCTCATGATTTACATGAAATGTTTGGTGATGTCAAGTTTGTTTGCATGGGCGGGACACCAAAGCGTATGGAAAACTTTGCACATTTCATTATGAATGAAATCGGTTATAAATTACCAGCGGGCACTATGTTGCAAGATATTAGCGCCTACTCGTACCGTTATTCCATGTACAAAGTTGGTCCAGTACTTTGTGTTAGTCACGG

>BPA_12295

AAATCATTGCCAATTCCGTTACCAGTTAAATTCAAATAATCATTGCCGCCAATCCATTTATGTAAATTCCTTGCCAACCTCATATTTTCCTGTGTAGCATTTGCATAAGTTGCCGTTGTCGTAGCCCCTATTGTCGAGGATCTCTTTATGGGTGGTTCATCCTTAGCCGAAGTACCATTTATTTTATCCGCTGTCGTACGATTAATAGGACGCAAAAAATGCAATGGCGGTGGT

>BPA_12311

CAGTAAAGTGATTGGTGAGAAAATGAAGAAAAAAGAACCTCAAGGTGATGAAGCTGCCGAGGTGCCGGCTGAAGTGCAAAAAGATTTAACAGAGTTGAATGCAGAGAAATTGGGTGAACTTACGGTGAATCAAATTAAAAAAGTACGTGTGCTCATTGACCAAGCTATGGTGGAGAATGAGAGACTTTTACAGGAGGCTGAACAAAAACGTAATACAGCTTTGCGTGAGGTAGGAAATCATTTGCATGAATCTGTACCCGTATCCAATGACGAAGAAGAGAAC

>BPA_12320

CGTATTGGCCGATGCCTTGAAATGCATTAACAACGCTGAAAAGCGTGGCAAGCGTCAAGTCCTTCTCCGCCCTTGCTCAAAAGTCATTATCAAGTTCCTGACTGTCATGATGAAACATGGTTATATTGGCGAATTTGAAATTGTCGATGACCATCGTTCAGGCAAGATTGTTGTCAATCTTACCGGTCGCTTGAACAAGTGCGGTGTGATCTCTCCCCGTTTTGACGTTCCCATTAACGACATTGAAAAGTGGACTAACAATTTGTTGCCTTCTCGTCAATTCGGTTATGTTGTATTAACCACATCCGGCGGTATCATGGATC

>BPA_12322

AACAAAGAGCAGCAGCTCCACCAGCTGCCGCCTATATATATCAACAGAATGCAGCAGCCGCTGCGGGCTTCCCTACACAATTAATATCACTACATCAAATTCGTAACTATGCTCATCAACCGGGAGCAGCTGCAGCAGCTGGTCTGCTGGCCGGTGATCATTTATTGGGTCTAAGTGTAACTGCCGGTGGTGCGGGTAAAGATAAAACGCAATAGTATCATGCAAAGC

>BPA_12329

GCCAGTGTCAGTTCGGGCGGTAGGACTGATTCTGATACAATGTCTCTTTCAAGCTGTTCTATGGATGGACGCCCATATATTCAACGAAGACATAGCTCTACTGAAACTAAAGCTATACGACAGAGTGCCTTGGCTAATAAGGAAACAAATTCCTTTCAGGTAATACCTCGCACTCAACGTCTTCATTCGAACGAACATCGTCCCCTAAAAGAGAATGAATTAATAGCGTTACTAATACCGAAGTTAGAGGAGGTTAAACGTAAACAGGACCTCGAAGAAAGAGCCCGTTTGGAGCATTTTCCTGAAGAATCTTTGCCGACAAATGAAAGAATTGCCAGTGATCGTGCGTTTGCACAAGCCATACGTGAAAAATTTGCTATCGATGAAGACAATGATCAGGACATACTTGATCAGCATGTTTCCCGCGTTTGGAATGATCAGACACCACATCGTTCTCCTGGTACAATGTCACCCTGCCCACCTTTACCATCCAGAAGGCG

>BPA_12342

CCACCACCCAATAAATCAACATTAGTAGGAGCAGCAGCAGGCATTGATGGCGTATTAATGTCCATTGATAATAAATCGCCAATTAGAGATTCCTGATTGGGTATAACCATAGCTTCACCACCAGCGCCAGCAGCTGCAGTAGTTTCAGCATCACCAGTAGCGGCAGCACGATTGGGCAAAGATTTACGCACACCAGCACCTCGTCCTTCAACGAAAGCAGTAGGAGGCTT

>BPA_12344

ATTAGGTCAATCCCTCAATCCAAATAAATCCAAACAAAATGCCTCTCAATCATCATCTGCCAACAATTCAAAACGTACATCACCCACCTCATCATCTGCGATTAATAAAACAGCTCAAATAAATCCTCAAGTTATGCGTCAATCACAAACTTCCATTGATAAGGCTATTGAAGCTACAGAAAAACCCATATTTACTGTAGGACGTCAACCCAAATACTCAAGTGG

>BPA_12355

TCTCGGTCGAACGGGGGAATCTATAGAGGCTGGTGGTGTTCTCGGCCATTTTGGAGGTTCTTCTACATCAGAACGTGAACGTTCTGAACGTTTCCGTCTCGAGTGTCTGGAGTACACTGGACTTCTGGACCAATCTCTTAAAGGTTTCTCACGATCTCTTTCACGTTCTCTTTCTCTATTTCTCATATACTCATTACTCTCTGAGTGGCAGCGATTACGTGCTGAAGGTGATCTTTCCGAAGAACGA

>BPA_12369

ATTAACATTCTCTTCTAGATTTTCAGTCTTAAATATTTTTGCACTATCTTCATTACACTCTTTCAATATCATCATGGCCTCCTCACTAGGCCTTCGGTATTTATCAGCACATGTTGTTGTAGTTACACTTGAACTATCTTGTCTCTTCACCGATTGCTGATGATCGGAAAAATCTTCAAATGATTTTCTGCGATTCAGTTTCTTTTGTGCATTTGTTCCGCTGACAATCGACACATTTGAT

>BPA_12375

TTTTACCTGAATATTGTTGATTGTACGTGCCACTAACTTCTCAACATAAGATGGATCTGCTTTATTTTCGTCTTTTGCTAACTCTTTTTGATATGCCTCATCTATGGCCTTTAAAGCAATTTGTTTTTGTTCTAATTCTTGTCTTTGTTCTTTCTCCGCATCATATGGCACACTTTGTTTAGGTGAGACAAGTAAAAACAAATCCTCAATTTG

>BPA_12381

GCTGCCACATGTAATAAATTCTTAACATCTTCATAAGTAAGTTTAGCACTGGCAGCTCTTAGACCCTGTGAACGTTTGTACCATTTCAACTGTTTAAGACGCATATTCAACTGTTGCATTTGTGGCAATTCAATGCGCAACGAATTGCCTTCGCTTATAATTGCTTCCAATTCGGATTCCTCTAAGGCATCCAAGGGATGTTTTAAAATTTCATTGGTGCGTTCCACGAACTGTTTTCCTAAAACTAAAA

>BPA_12391

ACATAAAAAATTAGTGGAAACTGGTGGCCTACAAATACAAAAAACCTTGGAAACGGCTAATAAATTTACACTTCAACAACAAGTTCAAGGAACTCCTGAAAGGCCACTTACCTCCGTTAACAATATGATTATGGCACGTATACAAAGGGATCTTGATAGTCCCTCAGCCGCAGCCCGTATGAAGGCTTTAAGAGCTCTTAAATCTCCCACTAAATCTGCTTATACGCAATTTGACATTCCTGAGGCAGAACAAAGTCTTCATTTGCCCGAACCTCCTA

>BPA_12401

CCGCCACCACCGCCAGCAGCGACAGCGGCTGGTGTATTTTTAACACCACCAGCTATTGACAAAGAGCCTGATGCAGTAGGTAATTCTCCCAATTGTTCACCCAATTGCAAGCCCAATTCATCCAAAACCTGTGAAACAACTGCATCAGTTTCCTCTTCATCACCCTCATCTTCCATGGCATCGTCTATAGCATCATTAATCATTTCTTCCTTCATATCCATAATCTCTGATTGTTTTTCGAAATCATGTAAAATCTTTTGGATTTGGGGTAAA

>BPA_12409

TGCTAACACCACAGCTGCCACAGTGACAGCCACCACAACAGCAGGAGGTGCAGCCACAACAACCGGTTTGCTTATGCCAAAAATGGAAAGTATAACCAATATGGAACAAAGTACGGTAACTTTTGCCCCAGATGGTACGGTGGTCTCCACGGGGGAACATGTTTGCGATATTTGTGGTAAAATGTTTGATTTCCGCTATCAATTA

>BPA_12438

TGCAAGTCAGCACTTGCAGCAATAATTCGTCAGTTAAGAAACGTTGGTTAAGGCAAGCAATCAGCGAGGAAACACATGAAGAGTTCCAAAACAACTTCTCGTTGACTCCATCTCCATCTGTTTCTGTCTCTGTCAATAACAATTTTGCAACTGCTCAAATACAGCCAACGGTTGTTGCTTGTTCGTCAGCTTCAGTAACAAATCACGTACCAAATGGTTTTACCACTCCACTAAAAAAGAGGCGTTTGTTATTAACTGGCAAGGATGAGTTAGATGAAAGTGTGCAGCAAACTCCTGGAG

>BPA_12465

GTTTGCTGTTGCAGATTATTTTGCGGTTGCTGTTGCATTGGAGCTGTAACTCCCACAAGTTTAGTGCCTGTAGATGTTTGCGTTTGAAATGGCTGAATTGTTACTACATTGGAAGTTGTGGTAGTCGTAGTTGAGGTGGTTGATGTTGATACCACTGGTTTGCCACTTGCTGTATTAGGCTGCACTATTTGAACTGGATTGTTATTAGGTTTTTGGGATAGAGGATCCAATTTCGGCATTGGTTTAACCACTGAAGCATTTTTCATAAGAGCTACATCAGCTACTGTAGTTGTTGAGGCTTTTGGTTTT

>BPA_12468

AATTCGTATTGGATCATCATTATGTGGAACTGGTGTTTGCAAAACGGTGTCTAGCAATAAAACCCCGTTATAATCTCCTCGGAAACCAATAACATGGCCATCTGTTATAATAAGTTTCTCTTGCAGTTGCATAAAATATCCAGATATTTTATGGTCTGTATCATTTTCCAGATGATATGTCTGCAATATGATACCAGAGTGGACATCGAGAACCTTAATTTGATTGATACTATCGAATAC

>BPA_12475

ATTGTGCTTGAACAGGTAAAATATAGGGCAAACTGGCAAAAACATCAGGAGGCACAGCGTCGTCGTGAAGAGGAGAAAATTGAAAAGGAAAGAGTCGCATATGCGCAAATCGATTGGCACGACTTTGTTGTAGTGGAGACGGTAGATTATCAACCTTTTGAAACAGGTAATTTCCCACCTCCAACAACTCCTGAGGAAGTTGGTG

>BPA_12485

TTCCGTCAATTCCTTTAAGTTTCAGCTAGTTTAGATGATACCCATGGTCATGTACATATGCAAATTAAACCCGAAGTTGATATGAGCGGAGTAGCACAATCTATGCCATTGGATATATCGGGTGCCACAACTCCCTCTGAACATGATGCTCCCAATTCCCAATCTGCACATCCAGGTTTACAATGGACGGTTGTTGAACCAAATTATTCACGCTTTTCGCTGTCATCCTGTCCCAC

>BPA_12490

CTGGTAATGGTGGCGGCAGTATTGGTGGTGGGGCAAATGCCAATGGGGATTCAATTGATCGTCGTGCGGATGGTATGCCACAAAATTCACCACCACCCATGGAAAGACCTAATTTGGCAGGTAAATCATGGTATTATGGAGCTATAACACGTAGTCAATGTGATACGGTCTTAAACCAACACGGTCATGATGGTGATTTTCTAATACGTGACAGTGAAACTAATATGGGTGACTATTCTGTTTCTCTTAAGGCTCCGGGACGTAATA

>BPA_12499

CATACTTGATCGTTTGGTAACGTTATCACAAAAAACTAAACCTTTGGATTTGCGTAAATTATGTGTACGTTTTCGCAATCGTGCTAAATTTAGTTCACCTGAACTGTTAAAGAAATATCAACGTACTGTGGGTACCTGTGAAGTTTGCAAAAACTGTGATCAATTTTTCTTTAATTGTTTATGCATGGAATCACCTTTTGTAA

>BPA_12500

TGTCCTTGGCATAGTTATCGTGATTTGACTGAATGGTTTGCTTCGCCATGTTCGCGATGGATTGTTATTGAATATTATTTGGAAAGAGTAAGAGGCACATTATCTTTATTGGATCAATTAGACTTAATAGGACGTACTAGTGAAATGTCTAAACTAATAGGCATACAATTCTATGAAATTTTATCGAGAGGTTCACAATTTCGCGTAGAAAGTATGATGTTAAGAATAGCTAAACCTAAAAATTTAGTACCTTTATCGCCAAGTGTGCAACAGAGAGCTCATATGCGT

>BPA_12505

GATTGCATTAAAATAATGTCCAGTCGTATTATCAAAATGCGTGAAATGTTGAGAAAACATTTGGAAGAACTACAAACACCAGGTAACTGGGAACACATTACACGGCAAATTGGCATGTTCTCATATACTGGTCTCAATGAAAAACAAGTGCGTATTTTGATTGATGAATATCATATTTATCTTCTAAAGTCTGGACGTATTAACATGTGTGGTCTTAATGAAAATAATGTTAAATATGTTGCTGAATCTATA

>BPA_12517

TTATTTCTTGTACCAATTTCATAGCTTGTGGTTTATTTTCAGCCAAGTAACGCAAGGTTTCAATCTTACGTTTAATATCGGATTTTTCGCCATATGCATCATCACATTCCAAAAGTTTGGCCTTCTCACCAGAAATCCAGGTTTTGGCCGCCGATATTTGATTGTTCAAATCATTGTGTTTTTGCACACAATCATCCAAATTGTGCAAAATTT

>BPA_12521

GGTCCACAACACAAGGGAGCTCAAGAATTGGCGAAATTATATAGTCCAGGAAAACGTGCTCAGGAATTTCTTTGTGTTTACACATGCATCGCATTAATGATAATTGATTTCATTTTAATATTAAAACATTTTCATTTCGATCGTATAATTGTCACCTGCATATCGGCTATATGTGGTATAATAACAGCAGATTTTGGTTCGGGT

>BPA_12532

ACCACCAGCATTACTAGAACCATTACCACCTCCACCACCACTACCACCATTCTTTTTGCCTGATTCCGAAGGATTCAATACATACATAAACTCTTGTACATCTGCCTTACGACCAACTTCATCCTTACTCAATGTTATATTACCACCCAACCAGGCGCAATTATTGGGTAAATTGGCTTTTGTGAGTTTCTCTTGTGATAACGGTGACACGTACAATACTTTAGGACAATCCTGTTTTACAGTAGAAAAA

>BPA_12533

TTATCTCAAAATCACATACGAAAAATTGAAAATTGTGGTTTTGAGGTATTACCACTATTGAATACACTTAATCTTTCCTCAAACTATTTAAAGGACAGCGATGGTTTAAAGGAATTGGAAAATTGTAAAAATCTATCAGTATTGGATTTATCCAATAATCGCATTGATGATATACTGGTTGTTAAGATTTTTTCGCGTATGCCTGAATTAAAAGTATTAGTATTGCAAGGAAACCCTGTA

>BPA_12543

TTTCACTTAAATGTAAATATTGCAAAAGTGAAGCAACATCTACACCACTTTTTGATAAATAATAAAAACCACACATCCATCCCGCTGAGGTTACAACATGTACGGGTATCAACACATACCAATATTTCTTATACATAAGTTTAAACTTTGCAAACAAACCCAATTTTGAGGCTTCACCAAACAAATCCACCTCTGAGATGGCTGCAGATTTTGCATCAG

>BPA_12558

TGTTAGCCACCACCTCTTTAATAGGATTTGTTGATGTTTCGGTGGTTTTATCACTTTCGGATAAATCAATTAATTGTTCTGTATTACCTCCCGCTAAAGCAGTTGCTATATTATCAGGATTTTCACCAAGTAATTTAAGTTTTGTTTGATAGGCCTGCATTTTACATTGCATATCATCCATTTCGATACGTAAAGCTTCCTCTAAATGAG

>BPA_12564

CGCAAAAAGTACGAAGACTTTGAAAAATATTTATTGCCCATTGGTCGTCTCTCAGAATACTCATCACTACGAAAATTACTTATGGAAAAATACAAGATACATGATACGTCATTCGATCGCCGTCAAGCTGCTCCTCGCTTTGTTATTCGTCCCACTTCACAATTCTGTTATGAAGGCCAAAGTGTCAAGTTCTATTGCCGCTGTATAGCCATTGCTACACCCACCCTAACCTGGACCCATAACAATGTTGAATTGCGAC

>BPA_12571

ATATTATTGCAAGTTAATTTTCTTCTCTGGTCTTGTGACAGCGTCTACATTGACGTTGTTGATACGGTAACAGCGGCTGTGAAGGAGCCGCAAAGACATTGCAAAACTGGAGGTGGATCAGCAAAATCAGACAAGTTTTCGAAAATTAACAAATATTACAAATTTATCTATTGCTGATGAAGAATTTATTTACAATGTTAATTGTGATATTGAACATTTTCTAAAGCAGCGCCACACTAACGGTGTTTTACTTTTTCCTCCTGTTAACAACTTTCGACGTTTTCTTATTCATC

>BPA_12582

TCCTGACATAACATTACTGTCGAATGCTATCAATAAACCCGATATCATTGCGGGTACACCCCAAGCAAATGCAATCATGTATGGCCATAACTTCAACACAAAACAAAGACTGCGGCATTGTAAGAATAATAATGATATGGCCAATATGCCAGTCCATAAACGGCTGCTGTAAGTACCGATATTGAATAATATGAATTGTATAAAC

>BPA_12591

TATAAACCTACATCAGAGGTAGATGTAAGCTCAATGGCGGTGGGAGCATTAACAAAAAATCAAAGCACTATGGAATCTTTGGCGGATTCTATTTTATCCATGAGTCTTTCACAAGTTGTGTCCCAAAGTCAAGCAAAATTGTCACAAGATAAGCTAAATGCCATACGGAATTTTACACAAAGACAACAGAAGATAAATATTATAAAACCCAAACGTCCGGATCGTATTGGTTTAAAATCAG

>BPA_12598

TGCCGCTGTCGTAATAATGTTTGCCTTTAATGACTTGGCAAATGTAAACGGATATTGAGCCTTCAATTCAGATGATGATTCAATGTTTTGTATGAGCAGTAAATGAGCAGCCAAATGTTTGGGATGTGCACCAATTATCTCTTCATAGATTTTTTCAGCTTTTTCAAGTTCACATTTGGTTAACATTGAACATTGGAAATCAC

>BPA_12607

CTACTAATGCTCCATTTGAGCAAACATTTCGCATAACTGTATTACTGCCAAAAGATCAGTTATATGTAACCAGATTAGGGGCTCGGGTACCCTTAAGTAAACTATTGGAGTTGGTGTGCGATAATAAATTATTAGATTCTGAAAAATATGAATTTCGTAATCCGGTTGATCCTAGTCAAGTCTACAGTTGCGATTTAACCATTGGTGCTGTGGGTCTCTCCGAAATACGTTTATGTCACAAAACCGAGTCATACGATAGTTTCAATAGTGACGAAATCATTAAGCTTCATCGCACTTCT

>BPA_12617

CGATAAATCATTATAACCAAAATTTGTATTTAAAGGATAATAACATGAGGCCGTTGAAGATGAGGAGGAGGAAGAGGGCGTGGTGGTATTATTTAATGGCCTTGGTAATTGCATTGTTATGGCACTTTGTTGTATGGGCTCCCAAACTGGTGTAGATGATGGTGATTTTCTCGAACCAATAGGTCCTAAATCTGTTGCAGTATTCTTCGGACGTTT

>BPA_12622

CATGCCGCCAGTATTTGGTGATTGTAGATTGGGTGATTTACTACCTAATTGATTCATGCCACCGGCACCCATAGGATTACCAACCATGCCAGGATTTTTATTACCCTGTTGCTGTAACAAGTGTTGTAAATGTTGATGATGTTGCATTTGTCTTAAAGGATTACCACCGCCAGGACCTTGATTTCCAACGCTACCATCATCTACGGAACCATTTAATTGAGGACCTGGTCCACCGGGACCTGGACCTTG

>BPA_12623

GCGCATAGCACGATGTCTGGCAGCTTCAAAAGTAATATCTGGTCCCGTTTCATCAGAATTTTCTCCTACTATTGTTTGATCATCATCATTTCCTAATGGCGTTAGGGTATCTTTACTTTCGGTACTTGCACCTGTATCCTGTCGCACAGTGACATATGGCGTTCGTTTAACTGTCTCACATGTAGAAGGACGTTGTGAAAGGGGCACATAAACATCTGAATCAGATTCAGATTGTACAGATATTTTACGCACAACTGGTTCATTAAGAGCTTCACGCGATTTTACAATTTCGTCAGATTCTTCCGGCACTGGTACTACTTCAAAATGTGTGCCATCGTCAAAGATATATGCATCTGGTCGGACTTCAGTTAGTGAACGATTAAACCTTCGACGGTTTGTAACCTGTAAACTATTTGATACCGATCCAGAACCGCCGCCTCCTCCACCAAGTCCGCCACCACCATTATTTCCACTACCCGGACCACCAGCTTCATTATCTGATACGGAAATTGTTCTTAAACGTTGCAAGTCTG

>BPA_12625

TCGCTTTTCTTCTTCTTGAATTTGTCGTCGTTTTTCTTCTTCAGCTTGTACAAGACGCCGCTTCTCTTCTTCCATCTGTTGACGCTTACGTTCTTCTTTTTGACGCCGCTTTTCTTCGTTTTGCTCACGTCGACGATCTTCCTCAGTTTTCTTTGATATCTCTTCCACATTTTCCATTTTCGCTTTACTCGATAAGGTTTTTCCAATGGCGACATTGTTATTGTTGTTAGTGCAATTTCGC

>BPA_12634

GTTGTTTGCATAACTTTGGAAATGTTGTTCATATTGCCAAACATAAATATCGCCATCAAAAGTAGAACCAGCAAACATATTTTTATTGAATGGATTTGTACATAAACTCTTTAGACAAGCTTTAATTGTAATGCTCTTAACTTCATTGTATGTTACGAAGTTTCCATTCGGTATACGTTTCGGTATAAAAATTTTTAAATACTGATCAAC

>BPA_12659

ATTTTATCCTTTTTTTTTAAAACTACGCCACCCGCGTTATCTGTTGATATGGTATTCTCTAATTTTCGTTTAGTTTGAACTGGACTTTGTGAAGGTGTGATGGTACGTGTGGGTGATGGCATTAGAGCTGCTTCTCCGCCAGCTGCGACCATATTTAATATTCTACGTTCTGCTTCCGTATTTGGAACGACTTCGCGTATTGACCC

>BPA_12664

AGCAACTACATGTTCTCTCATGGATTCAGGAGGGCCTACAAGCGATTGACGTTCAGATGAGCGCAATTGTTGGTAGAAGGTCTTACTAATCATGCGACGACGAGCATCAAATTCATGAGCAGCAATATAGGGAATTTCGATAAGCATGGCGGATACCAAATAAACACATTCCAACAATTCCAAGTTAATATGCATGTGGAAGGGCATT

>BPA_12665

CTCGCTATCTTGACAAACAGCATCACCATTATCGTCTTCGCCATCTTTGGGCCTTGTTACAAATTGTATATCTTGAGAGGGATCTTCAGAGGCCAAATGGGCGCGAAATTGTTTGTTTCTTAATTCCAATTGTTGCATTTGTTGAAAATGTTTCAGTTTTTGATTGGCAACACGTAATTTCTCTTCCAAAGCAGCATTTTCAACACACTTAATGGAATATTTTTCGGAGAATGATAGAATTTCTTGACGCAATTCTTCCAATTCTTCTTCCTTCGCCTTGGTTGAATCTAAAGAACGTTCGCCTTTTTGTTGAAATTGTTTAAGAAATTCTTGTTTAAAGCGTGCAACTTCACGTTGAACTTC

>BPA_12672

ACCTACACGATGACGGGTGATACAAGACATCGTGGTATAATGCCTAGATGTTTAGATGTTCTATTTCGTACCATATCTGATTATCAGACTAAAAAATACATATTCAAACCGGATCGTTTAAATGGTTTTGAAATTTTATCAGAAACAGATGCTTTGCTGGAGAGACAACAAGAAATGAATCAGCGTTTTGCTGGCAGGGGGCTCCTAAAAC

>BPA_12675

GTAATTATTGAAAAACTTCAAGTAGAACCACATACAAATGGAAATGAACTGGTGCAGCCTGATTTGAAGGAAACTATAAAAGAACCCTCTTTAATACCTAGACTTACAGAGACTAAAAGCTTTATTTTGAAAATGAAACGTTTATCACACGAGCAAAGAAAATCACAAATATCTTTGGCAAATGCTTTACTACAAAAAGGCGAAAATGTTGAATATGGCGCAAATACTCTAACTATAAATGATTTCTCTTTAACATCCAC

>BPA_12676

AAAGTAATTGTACCGGCCCACAACAACGAAATTAAAAAATGGCCCCACCACAGAGAATGCGCGTTGCTAATGAAAAAGCCAGCAAATATATTACAATGCGTGGCAATGTACCAAAATCTTCGAAAGCTAAAGAAGGACAATATCCTGTTGGACCCTGGCTCTTAGCACTTTTCATTTTTGTTGTTTGTGGCTCAGCAATTTTCCAAATTA

>BPA_12702

AAAGCATACTTAGGTATAAGTAAGATACTTTGGATAGGAATTCGTATATTATAGTTTTTGAAGGACATACGGGGAGACTTCAAAAGGTAATTTATCGTATGAATGACAATGAACAGAATTTGTCGCGGTCTACAAAAATAGTTCTGGCAAAACTGACATCGAGATGGAGCAATAGGCTCTATGCATAGTGGAATCGTATTGTCTGTGTCGTTCCAAACCTACGTCCTGAATGTGGCCAATGACCACATGATACACGACATTTATTTAACTGCCAGA

>BPA_12705

GGACCATCAGGTCGTGGTCCTATATTGGGATATTATATAGAAGCTAAGAAACGAGAAAGAGGAGAACCATCATTTATTTACGACTCCCGCTGGGAGACAATAACAAAAACGACCACAGGTACGATACAGGACTTTACTGTCAGTTATCAAAGTTTGTTGCCATCTACTGCCTACACATTCCGAGTTATTGCATACAATCGTTA

>BPA_12709

TTTGAGTGCCGGGCTCTTCTTTCATTTGATGTTGTGCATAAAAGGCGGCGGCAGGATTTCTCATGGTCATTTGCAGGAAATTAAGATTGGCACCATTGTTATTGTTATTATTACTATTAGCACTATTGGGTGATAAAGCTGAAGCTGTGTTATTGTTATTGCTGTTGCTATTGGCATTAGAAGATGAAGAGTTTTGGGTTTGAGCAGGTGGC

>BPA_12722

AAAAGGGTCGTCTTTAAACAATCACGAGGGCTTAACGCATACGCCTTATGTCGCGACATAAGCTCTTGCATAGGCTCCAGTATCACGCACAAACGCAAATAATTTAGGGTTGAGTTCGTGATTCCTGCCCTCGTGATATTCTTCGTTATTTGTTCTAAAATCATGGGATCTGATGGTAGAGAAGTACCAATTACTGACCGTGGTATAAGTTCTCGGTGGCCTTTGATTGTCATGTGCCACG

>BPA_12732

GTTGCATTGAAAAGAAAATTACTTGAACTTAGGGAAAACAATCGCCAAGCCATGGAAAGATTTACAAACGATATACAAAAATTACATGCCAGTATAAGTGAGCTAAAAACAAAAGCTGAACATTTCCGGAAAAATAATAAATCAAAATTGGTCAAAGTTTGGCACATAAACTTTAAGGACATTAAACATTTAATTACACGAGTATTTGAGATTGATCGTAT

>BPA_12739

ATTATACCAATGGGACGAAAATAATCCTTATGGGATTTCTTTTCAGCAGCTTTTGAATCTGTTGTTGTTGTTGTTTGGGGAACCGTTGTTATTACATCTGTTGTGTCTGATGGACTTCTGATGGTCAAGGTCGTCGTGGATTTAGTACTACTGCCGATGTTTGTTTCATTTTGATTATGATTATTTTCCAATTTACGACAACCTT

>BPA_12740

TTTTGGGCATTTCTGGATCGGCCTTGCCGGCAATCATCCAGCGACTATTATGAAACTTATAACGATAATCATCGGCAGCCACAATATCCAAAAGAAGTATATATTTCGCCTTGGCATCGAGACCCGAAACTCGAAATTTCATTTGGGGAAACATTTGTTTGTAATTCTATATTTTTTGCGCTGCTCCTTTTCTAGAATCCTTTTTTG

>BPA_12741

GTAAAATATTCGAAAGCCAATTGTTGGGATGTGAAGGTGTTGAATTATCGTTGACATGATTCTGATCTGCTTCATTTTGTTGTTGCTGTTGTTGTTGTTCTTTTTGCTGTTGTTGCATTTGGTGTTGTTGTGCCATCACTTGAGGTGATGAATTTATAGTTCTAGGCGTATTTGAACGTCTATGTTGTTACGGATGTTTCGCAAAAGTTG

>BPA_12743

AAGTTCGGTTGTTACGAAAGCAAGAAATTTGGGCGAAAAGCGTTTATATTCTTCTTCTTTGTTTTAAGTTATATTTTGTATTGCAAACCAAAGGAAATTTAGAGAAAAAACCTAAGAAAACAAATTAAATTCGGAAATGTCTCTTGTACCATTATTAGTAAATTTGGCTCGTGGCTTGGAAAGCGATTATCCTCATGGAATGTTGGATGATTGGGATCGTATGTTGGATGATGATTTTGGTTTTGGTATAAATCCAGTCGATATATTTCGTCCACGTTTAGCTCCTGGTCACCATCTTCATCATCATCATCATCATCTTTTACCTAGACGTTCGGCATTATATGCGCCATATTTGTTAGGCAGACGCCAACGTCAGCAACGTGACAGAAATGATTCAACATCTGGTAGCACTGCTTTGATGCCAACCATTGGCAAAGATGGTTTTCAAGTGTGCATGGATGTTTCACAATTCAAACCTAGCGAGTTGACTGTAAAAACTGTCGATAATATTGTCATTGTAGAGGGTAAACATGAGGAACGCGAAGATGAACATGGTCTTATACAACGTCATTTTGTACGCAAATACTCACTGCCCAAAGATTATGATCCAAAAGATGTTGTTTCTACCATTTCTTCAGATGGTGTTCTCACCGTTAAAGCTCCTCCACCACCCAATAAAGCCATAGCTGGCAATGAACGTGTCGTTCAAATTCAACAGACTGGTCCGGCACATTTGAATGTCAAACAACCTGAACAAATTCAGTCCAATAAAATGCAACAAACTCAAGCTACTAATGGTCCAGCTACGGCT

>BPA_12759

TTCAGACAATCGAAATGGCCAAATTAGATGAAGAGTACAATGTGGAATTGTGTACCACACCGCCATCGCATACAGATTTAGAATTGGCTTTAATGGATAATGATAATATAGACACTAGTATATACTCTTGGAAATTAGTCAAACGCAGACGACCCCGCCGACGTTATACAGTGTCACATGGTGGTGGTGGAAGCGTTAGTAATGGAGCTGGCTTATCATTAAATGGTCATACAGCTTTAAATATATGTGCAAATGGTGGCGGTGGTGGTGGTGGGGGAGGAGGTATTAATAGTGGTGGTTTACCTCCAGCCCCACCCTCAACACCCACTTCTCTTACCCCCACTAATACCAGNNNNNNNNNCCAAATTCCGGCATTGCCGGCATTGTAACCGAAGAAGAGCAACAAAGACTCGATAGTCTACTCTACATTCTCGACTATGCACCCAAGTACAAAAAGAAACTAAAGGAACAACAACGTACCGATGCTGAGTTGGCCGAACTATTTAATGTTGTCTCCATAACCGCCTCAAATGGTTCTTCATCATCATCGTCCTC

>BPA_12760

CTTTGCTTTAGGTCTATCGCTGATTGCAGTACTTTGAGACACTTCTGAAGGTGTTGTAATACCACCAGTCTTTTGTGTCGTTTTACCAGCAACAATACGTTGCTGCTGCTGCAACTGTTGTTGTGAAGCTTGTTGTTGTGACTGCGTCTGTTGCAGTTGCTGTTGTTGTTGCTGCTGAGGTGGGGGTGCAGGACCTAATACAAATTGTGTAGTGGTTGCCAAAGTTAAAGGTGTCTGAGGATTATTAGGATCGAAAAGTTCTTGCATCATTAGTGCCTGGTTGGGTGCAAAGTTCAAAGGTTCTGGACTCGCTATAATTACCGGTTGCATTTCTGAACCATCACCGATAGCAGGAGCAACTGCTAATAAATTTTGATTCATCGTTAAAGCGTCTGCGCCATCTCCAACATTTTGATTTTGGTTATTAACATTGCCGCCAGTCATATCCAAGTAGGAGGCAGGCAACAAAGTAGCAGGAATATTTTTTTGATCATTTATCTTATTGAAATCCCTTAAAACTCGACGTCTAATAAAACGTGAATCAT

>BPA_12762

AAAACGATCCGAATTGTTCAAGTAATGTTACAACAGGTAGTGTGAAAATATTAAAACCCAGTGTCATAAGACGTGATCTCTATAAGGATAGATTTAAAACTCAACCTGTGACCAGTGATGAAAAGAATTTCTTTAAAAATAACGTGTCATCTGTCAACAACTCGTTGGCAAGTGGTTCTGCTTTTAAACCAGCCCAATCATTAGAAGGTCTTGGTTTTGCACATTTATCCTCACACGGCAATCAATCGCCTAACGCTCCACCCTCAGCATTTACTTTAACGCGTTCATTTACTCAACCTCTATCAAGTTCTATCCATTCACCACCTCAATCGTCGTCTTCATTTAAGGCTGGTGTACCAGTGCCTGTACCGCGTAAATCACCACCCCGTTCTAATTTACTTAGTTCACAACAAGATAGTTCAATCGCTCATAATTTAACTCTGTCATTTACGGGCGATTCGGCGTGTAATAATAGTAATAGTGGTGAACGTTTATTCGAAATGTCTAGTCTTGAACAGGATGTTGATATGGCTGGTGAAAATCAAGAGCCAAAAACTCCTCTAAATAAAGACTCATCCGAATCGTCAGCATCGTCGTCGGGTGTGGTGCGTACGAATAGTGTACGTGCTAGAGCGAATATGTTCCAACAAATGCAAGAGAAGACGAATAATGGCGGCGCGGCTTTGAATCGGGAGGAACGTACATCACCGAAAAGAGTACCGCGTCGTTTATCACCTTCTCCTGCTGTTAATACGGATGAACCGAAAACACCAAATAATCCACCTGTTAATCCAGATGATGAAATCGAACCCTCATCCTTGCCAGTATCGGAACGTTTAAGATTTTTCAGCAGCCTAAGTGAAGTGGGTAAACGTAATTCGTGTTCTTTTACACGCAGCCCACCATTTAATTCCATCGAACGCAGTTCAAGTGTAAGTTCCTATCACTACTATATGGCAACAACACCACGCAGTAGTACCAGCCTAAGCAGTGCCAGTGTTACACCCACACCCATGGAATGT

>BPA_12764

TTTTGACCGATGGTCAAACAAATACAATTACATTATGTATGCGTAAGTGTAAACCTAATGATGAGGGAAAATATAAGGTTGTGGTATCGAATGTACATGGCGAGGATTCTGCCGAAATGCAATTGTTTGTATCGGATTCAAGCGGTATGGATTTCCGTGCCATGTTAAAGAAACGTCGTTATCAAAAATGGGACAAAGATCAAGAAGATCCCAATTGGGGTGAACTTAAAGAGACTGAAAAACCAATGCCCGCACTTAAAAAAGTTGAAAGGGCACCTACATCACGTAGACCATCGCTGGCTGAATTGATTCCAGATTGGCCAACTTTGCACCCATTTCGACGAGAAGAGAAACAAGAGTCGTTCTTAAGTCCACTGATTGATCAATTCGCTAAAGAAGGCAAGGATAAGAAGGTAGTCTTTGAAGCTCGTTTCAGCAAACCAAATTGCAAACCAAAGTGGTTATTCCGCAAAGATGAATGTTTCCCGGGCAGTAAATACAAATTTAAGAATGAAAATGACACATATCAACTAATTATTACAACGCCAAAGGTGGAAGACACAGGCAAGTATACTATAGAAATTGGTGGCGTTTCCAGTACTGCGTTCCTTAATGTAGAAGAAGCTGATCCCAGCTATACCTTTACCAAACCGCTGAAAAAGAAACTTGAGGGTTACAACAGAACATGAAACAACGTTAGAATGTTCTGTTAGTAGCAGTATGGCGAATGTAACCTGGTTTAAAGATGGCAAGAAAATTGAATCTGATGATCCACGTTATCTTATCTCGAAAGATATCAATGGTAATCTAAAACTAATCATTAAAGACTCACTATTGGAAGATACCGGTCATTACACGTGCCAATTGGATAGACAGCCCGACAAAACTGAATGTAAACTGAAGATTGTCG

>BPA_12765

GCGGCTTCTGTGGCAAATTTCAAAGATTTAATCTTAGACATGGCAGGTTCTAAAACACCTGCTTTTTTGTTATCACGCACAACACCTTCAATCAAATCCAAACCAGTCCATTTCAATAAAGCATGTTCAGCTTTAGTTTGACTTGAATTGTGGTAGGCACGCAATTTAGCCACTAAATCGGTTGCATCTTTTGCAGCATTAACAGCCAATGTCTTTGGAATGACCAAAAGTGATTTGGCAAATTCAGCAATAGCCAATTGTTCGCGGGAGCTCAAAGAAGTAGCAAAGTTTTCAAGATAAATTGACAAGGCAGCTTCAACACAGCCACCACCAGCTACTACCTTTTTGCTTTCCAAAACACGCTTAACAACACACAAAGCATCATGAATAGACCTTTCCATTTCATCACAATAGAAATAATTGGGACCACGCAAAATAATAGAAGCGGCAGCTCTAGCTTTGGTACCCCTAATGATAATCAATTCATCATCACAAATACGTTCTTGTGATACTTCAGCAGCTTCACCCACCATAGAA

>BPA_12772

TTACGGCTCACTGACAAGGGAATACATTTACGTTTGCTGAGTACCGATTGTATGGCGGAGAATACAAAACTCAAGAGAAACATAGCGGCATAAGGTAAAGCTGAGAGTAAGGCATTGCTCTTAATATCCATATTGAGAATATTTTTCATATATGAGGGAATCTCAGTTAACAAAGTCCAAAAGCCCCANNCCCAGTTATGAGTACAATGTGTGAGTGTCAAAACCATGAAGGGCAAAGAGGTAAAGAATTTAAGCCAAGGAGTCTTGGGAGTAGGTTTATGAGCATCTTCCTCATTTTGTGAGCCCAAAGATGTTTCAATTAATTTTTTTTCCTCAGCAGAAATATTTTTATATTCACTGGGCGAACTGGCACCCCAGAAAAACATGGCCACCGACCATACCAAACCTACACCTCCAGAAATATAGAATATACTAGGCCATCCCATGGAAGAAGATGCCATAACACCACTAGAAGCCAACATTACAACAGTGCCAAATTGAGTGCCCGAATAACTGTATGTACCCAAAGTACCTCTTTCCTCCACAGGAGCCCATTTGGAGAGTATAGTATGCGTAGAGGGAAAAAGAAAACCTTGGCTTAAACCCTGAAT

>BPA_12774

GCTAAGATACGGATTATGGCAGAAATTGTAATTTTTTTTGGTGCTATATTATATTTATTATCGGCCTTAAGGGAAGCTCGATTTTTGGGCTATAAAATGTTTGTTGAAAACCTTATGACAGCACCGAGAGTTATGTTTTTATTTTCATGTTGTTTAATGATGACAATACCATGGTTGAGATTATCTTGTCTAACGGAATTAGATGATCATGTAGCGGTGTGTATTATGCTGACAACTGCGCCTTATTTTCTTTTCTTTTGTCGTGGTTTTAAAACAGTCGGACCTTTTGTAGTAATGATATATAGAATGGTTATGGGGGATTTAATTCGCTTTGTCTCCATCTATTTAGTGTTTGTCATGGGATTTTCCCAAGCATATTATATAATATTTTTAACATTTGATAATCCAACAACGCCTGAAGAAGTAGATGATTCAGAATCTAATCCCATGCCTTCTCCGATGGAATCTGTCGTAGCTATGTTTTTAATGTCACTTACTAACTTCGGCGACTATTATGGTTCAATGTCTTCAACACAACATGAAGCTGAAGCAAAAATACTGTTCTTTCTCTTTATGGTGATAGTCAGTGTGTTATTGGTCAACATGTTAATTGCTATGATGGGTAATACATATCAAAAAATAGCTGAGATTCGTAATGAATGGCAAAGACAATGGGCTCGAATTGTGTTAGTAGTAGAACGTAGTGTACCGCCTGCAGAAAGGCTAAACAATTTTATACAATATTCCCAACCCATGTCAGACGGGAGACGAGCCCTAGTTCTACGATTAAATATGAGTGATGAAGACAAAGAAGAAATGAAAGAAGTTCAGGAAATGAAAAGAATTCATGAACGTTTTTCGAAAAAACGCCAACAAGAGAGGGAAGCGCGCATTAAACGGAGAAATGAAGAATACGAAAAATTCTTTGGCACCAGTTCTTTAAATAATGAAACTAAGTAAT

>BPA_12777

ATGGCAAAAAGAAAAAACGCATCAAATTATGCGAAGACATTAATCGGAATGTGGTACAGGAACGGCGTTTTTTCTTTAGTCGTAAATCATTTAAACTTCGTTATTTATGGAAACGTAAACCGCTGATCTACCTGATAGCAATATTATTGTTAATACTTTTAGTTAATTTAACCATAAATCATGATGTTGAGGAACGAGAAGATGAAAATTTATATTTTTTAAGCAATTTACATTCTCATGTTCCGGGAATGAAAGGTTATTTAGTATGGAGTGAATATTGTAAAATGCCCAACTTAGATCCGTATCTGCCAGAGGTTATGAAAAATTTTCGGCGTGAAAAATATAAACCCTGCAAATTGTTATCACCTTTAACCCGGGTGGATTTCAATAGAACAACAAAACGTTATACTCTTTCTGTGGATCAGGATGTAATACTTTCATATAGTAAAACTGGCAAAGTGGATTGTTGTTATCAGTCCATAGTTCGTAATGGTACGGCGGAAAGAGCCGACAGAGATGTGAGTCTTTCCGAGTGCATGAGTTTTGTTGATAGAACTTCTTTATCGCCTACAATCGATAATATACTGGTACGATGTCGCTCCAATAATCGTCAGGTATACATAAATGGTTATCCATTGATGCCGGAGCGCAAAGAAATACGCCAACGTTTGGATACATGGAGAAAATATGATGAGCAATTGAAAAGGAAAGAGACCCCTCCTAGTGTGTTAATGCTGGGCATTGATAGTATATCCAGATTAAATCTGGTGAGAGCGATGCCGAAGACAGCAAAATATTTGTATAATAATGAGTGGTTTGAAATGAGCGGTTTTAATAAGATTGATGATAATACATTCCCCAATTTAATGGCCGTACTTACGGGTATGAATCGCACAACTGTTATGCAAAAATGTTCTCCGTACACCCTTAATGCTTTAGATAATTGTGATTTTATTTGGAAAACATTTCGCGAACATGGTTATGTAACGGCCTATGCCGAAGATGAAGCCAGCATAAATACTTTTAATTACTTAAAAGTCGGTTTTCAACAACCACCCGTAGATTATTATTTACGACCATTTCTAATTGGAGCCGAGAAGCATTTGGAAACTAAATTAAAATCAAGCTTAATCGACTGTCTGGGTTATAAACATTCAGCTGATTTTGTTTTCGATTACGCCATCGAACTGGCAAAGCGTTATAAGAATGATTCATATTTTGGTCTTTTTTGGGCAAATACGTTTAGTCACAATGACATAAGTGACTGTTCGGCCATGGATACACATATTATGGATTATCTGAAGAAATTTAAGGAAATGGGTACTTTAGATAATACAATAGTGATATTTTTTAGCGATCATGGCATGCGCTTCGGACCTATACGAAAAACCTATTCGGGTCATCTGGAAGAGAGATTACCCTTTGTTTTCCTGTGGTTACCACCATATCTTAAACAAAATTATCCCAGCTTTGTGAATGCCTTAAATGTCAATAAGAATCGTTTGACAAATCCCTATGACACGCACATGACACTTAAACATATTTTAAAATTAACAGGAAGAGTACAAAATGCAACAGTTTTAAATGGAGCAGATGCTTGTCCCAAGTGCCAGTCGTTATTAGAAACAGTACCACAAAATCGTTCCTGCCTGGATGCAGCGATTGAACCACATTGGTGCACTTGTTTGTTGTACGAAAGTATTTATAAAAATTCAAAAACCGTTGTCAATTTAACACATTTATTAATTGATTATATTAATGATTATGTGGCCACATTTCATAATCGTTCTTTGGCCAAACTATGTGTTCCTTTATCATACGATAGTGTGGAAAGTGCCTATCGCACCACCCAACTGCAAGTAAGCGATGACGGCAAAAGTATCAAAACTATAAATATTTATCGCATAACTTTCTACACAAAACCAAATAAAGGACTCTTTGAGGCAACTGCCATATATGATCCTTTGGAGAATTATCTGAAAGTAACGGGAGAAATAAGCCGTCTTAATACATATAGTGCCGATTCCGAATGTGTATCCGATGGTGGGGCTAAAAAATATTGTTCTTGTCGAAAGAAAAT

>BPA_12778

TATTCAGGGTCACGATTTCAGCATAACATGTGAAGCTACTGGTACTCCATATCCTAGCATTAAATGGACCAAGGTTCATGAATCTTTGGGTGATAATGTTCATCAATCTGGCAATGTTTTGCGTATCATAAATGCCCGCCCCGATAATCGTGGCATTTACTTGTGTATTGTGGAAAATGAAGCTGGCAGGCAATGATCAGACTAGCACTTTTGTTGATATTGAACCTCGTGAACATCCAATTGTTGATATTGATCCCAAAGAACCACAAGTCATCTCAGTTGGCGGTCAAGGTTTATTATATTGTTCGGCTACTGGTATTCCACAGCCTCGTGTACAATGGTTACGTGTTAATGGTCAACCTTTGTCTCACCGTCATCAGGCTCAACATGGTGAACCTGGTTACATTGTCATTGAAGATGTTAAATTGGAAGATGCTGGTGATTACAAGTGTGTGGCTGAAAATGAAGTTGGCAATGCTACTGCTGTGGCTACTATCCGTGTTATTGAGTCTCCGGTTATTACCTTGGAACCCAATCAAGAAGTTCTTACCGTTACCGAAGGTGATGAGGTTAAAGTTAGNATTTAAAGTTTGCTGGATTGCCACTGGTATTCCCAATCCCAGTGTACGTTGGGTAGAAGATAGTTCTGCTGATGTATACAATTATTCTCCCGATGCTGATAATTATAATGAAGCTTTCTTGGAATTCGCTCGTGTTTCCATTTCCAATGGCAAGGCTTATAAATGTGTGGCTACCAATGAGGCTGGCACTGATGAACGTTATGTTGTTTTGGATGTTAAACCAAGACGTGGTGATGCTCCTGAGGATAGCGATGTTGATAGATATCCTTATGACAGACAACCCTCAAGACCTTATCCACCACAACCCAGCTATCCACCACAACCCAGCTATCCACCACAACCCAGCTATCCATCACAACCCAGCTATCCACCACAACCCAGCTATCCATCACAGCCAGGCTATCCCACACAACCAGGATACAATTATCCCCAATATCCTTATGGTCAATCTCAGCCACATCCCGAAAACGTCTATCAATCAAAACCTGGTGACAATGTTACTCTCAACTGTGACTTAAGTGCTGCTTATAAGACCATGTGGGTTCGTGAAGATGGTCGTCCTTTGCCTCCCAATTCTCAATTTGAACGTAACAGTTTGATTATCCATCATATGCAAGAGAACAATGCTGGCAAATATAGATGTAACGCCTATGACAGTCGTGGTGAAATTATCACTTACATTTTAGCTGAATTGGTGTACATTCCCATTCCTCACATTACCTTGAATCCACGCATGCCAATTCATGTTAATGCCAACGACAATATTGATATCTCTTGTGATGTTGAGGGTGCCCAACCCATTATTGTTTCCTGGCATACCGATAATAACAGACCTTTGCCACCTTCCGTAAGCATTGAAGGCAAATACTTGCGTTTCATTTCCATTACACCCGCTGCTGCTGGCCGTTACTATTGTTCTGCCTCGAATAGCTATGGCAACACTACTGAAATGGCTGAAGTTATTGTTAATCGTGGTCACACCTATGAAGCTCGTCCTCAAGCCAAAAACTACGATTTGAGCGAAGGTGAATCTGTTCGCATAGCTTGTGATGTTGATCACTTGCCCATACGTGGTGATGTACATTACACTTGGACTCGTGAA

>BPA_12783

GACACTTGTGACAATGGCTACTGCTATTGCAAGGCCAATGTTCAAGGCACCTATTGTGATCAATGCCGTCCCGGAACGTATGATCTTTCTGCCAGCAATCCTGATGGCTGCACAGAATGTTATTGTTCCCACAAATCTACCACCTGTCGCTCCGCTTCTCTCTACCGTCAATTGATCCCTGTCGACTTCCTCAGCNNNNNNNCACTCTTCACCGATGAAGAGGGCAACATTGCTGATACCCAGAACTTGAACTTCGATATTGAAAGCAACGAATATACCTACAGTCACTCCTCTTACACACCCAAATTCTGGAGCATTCGTGGTTCCGTATTGGGCAACCAATTGTACTCTTATGGTGGTATTTTATCCTACAAATTGTCAGTACAATCTTATGGCAACTACGAACCTGGTCATGATGTTGTACTCATTGGTAATGGCCAGAAATTGTTGTGGTCTCGTCCCAGCAATGAACAAGATAATACCGAATACAAGGTACGTCTCCATGAAGATGAAAACTGGCAATCCATGCAATTGGGCTCTATGCAACGTG

>BPA_12786

CATTTGTTGAAGCCGTCTGAGGTGGTTGTGAACCAAAGTATAAACGTGTTGAAGTTACTGGTGGAAAAATGGTTAAAAATAAAAAGGGCAAGAAAGAGATAGTTTCAGCTGGAGCTGATGCATCGTCCGGAGAAGAAATCGAAACGGAGACTCAACAAACTGTCATTGACTCTGACGGTCAGGAAGAAAAAAATGTTAGTGCGAAAAACAAAAAGAATAAACAAAAGAAAGGCAAAAAGAAGAAGAATGATTCTGATGATGATGAGGACATTGATGTAGTCGAAGATGTAACTGCTGGGGTAGAAAAACTTAAAATTAAATCAAAAGCTAAAAAATCTGCATTTGAACTTCTAATGGAGGATGACGAAGATGATGACAATGTACAACAACAAGTAGTACAATCTGAGAGCGAGGGTGAAAGCAATGAACCCGTTAAGCCTTCTAAAGGAAATGTTGGCAAAAAAGGAAAAAAAGCAAAACGTAAAGGTAA

>BPA_12788

CCACACTAAACTGGGAGCCGGACTAGATGAAGAATAGTCTGTAGTATAACTTACATCACTGTGAGCCCCGGTTTGATTGTGTTGATTTGACGTTGCCACATGAGTGTTCAGAAGTTTTGTGTGTTGTAAAGGTAGAGCCTGATACGTAGAAGGATTTTGTGTATGATATGAAGAGAAATTTGTAAATTTTCTATCTGCTATGGGATGGCGCATTTTAAACAAATCCGAGGAGTCTGAAGAATCTACATCATGTGCCAATGACACCGAGGCTTCGGTAGGATCATCATTGACGTTAATTAGTCCATTTTTATGGTTTTCCATCAATTTATTTTCTGTTTCACAGGGATTATTATGATCCCTTTTCCACAGCTTCTCTTCATCCGACAAATTGCTCACTGATTCATTTGACAACTGTGTATCATTTTTATGTCCATTTTCTCGAAAAACACGATGACGATGACTTTGGTCGGGCTGGTGGTGAACTAATGTTCATTTCATCGACAAAATCCTTGGATTTTACTTTGCACCGGGCAAAG

>BPA_12792

CTTTCTCGCATAATGATTTTTCTAAAACAGAATTGTCTTCATCATCATCTACCTTAAGGCTTTCAGTAGCTAAATCATCATCATCTACATTTTCTTCATTTTTCTGTTCGTCACACCTTCATCATCACATTCCAGTATTGAGGGCGGTTCTGCTTGATCGAGTGTCTCAATCCATTCTTCTACGTCCGTTTTTGGCAAATCTGATAAAACATCTGCCGCAGTAAATGGGGATTGTGCTTGGGGTGTTTGTAAAGCAAACGATTTTCGTGGATAAACTTTACGTATAGGAGTTTGAATTTGTGTTTCTTGTCCTACAAGTTTATCAAAAGCTTCTTCGGCAGATTTACTTAGAGTGTCTTGTAAAGTTTCATTTTTGTTTTCATTGAATTTTTCTAATGTTTCATCATGTTTTTCGTCGTCAATAGTAAGAAGCTGTAATACGCTCGGCAAAACGTGATGCCACAGGTGTTTTAAAAAGATCTTCCA

>BPA_12800

GTCATATATATTGGGAAAATAAATGTGCAATAAATCAAAAAAATCACTTTCGTCAGCTGGAAGATTTTGATCGGTTAAAAGCTTTAGCAAATAACCAAAATCATAGCCAGAATGAAAGCAAAGCCACTTAATATTATCCATCAAAACAATGCCAGATGACATTAAAAGTTCGGCAAAATCTAAAGGATCAATGCCATCATCTTCATGTTTACGAAATTGTATACCAGAATTTGTCAGCAAATCTATAGAATCCTGGGCGTACATGTCTTCACTTAAATTAAATTTAAAATTAAATTGCCAAGTACTATAACCAGGTGGTGTTTTACCCTCATCATCCATAAAAGTCAAACCCAATTGAATGATAATCTGCAGTTGAACGAAAATCACCCACAGGACGTGCGACTACGCCAGGAAACTCTGTATCCATGGCAACATAATGATATTTTTGCACTATTTTACGTATAGTACGGAATTCTTCCTCTAGATTATGTTTCCAAACATCACGTATGCCACACTCCTCATTGCTG

>BPA_12805

CAATTGGATGAACAATTGGAACGTTTCGATAATTTGGATAAAGATGATCTTCAAGCATTAAGAGAACAACGTATAAGAGAGATGAAGGAATTTAACACCAAAAAACAAGAATGGTTGAAAAATGGTCACGGCACCTACAGTGAGTTGGCTGATGAAAAAGAATTCTTTGAAGTCTCAAAAAAATCTCCCAATATCGTGTGTCATTTTTATCGGGATTCAACAGAACGTTGTCGCATTGTTGATATGCATTTGAAAATTTTGGCTGGCAAACATATCGAAGCAAAATTTTGTAAAGTTAATGCAGAAAAATCTCCTTTTCTTACACAACGTCTGCGCATCAAAGTAATACCCACCATAGCTTTGATAAAGGATAGTAAAACAAAAGATTTTATTGTTGGTTTTACCGATTTGGGTAATTGTGATGATTTCTCAACCGATATGCTCGAATGGAGAATAGCACAATCGGGCGCAATCGATTATAAAGGTGATCTTATGACCCCACCAGATGTT

>BPA_12811
[truncated: 2,335,935 more chars]
